# Comparative genomic analysis and molecular examination of the diversity of enterotoxigenic *Escherichia coli* isolates from Chile

**DOI:** 10.1371/journal.pntd.0007828

**Published:** 2019-11-20

**Authors:** David A. Rasko, Felipe Del Canto, Qingwei Luo, James M. Fleckenstein, Roberto Vidal, Tracy H. Hazen

**Affiliations:** 1 Institute for Genome Sciences, University of Maryland School of Medicine, Baltimore, Maryland, United States of America; 2 Department of Microbiology and Immunology, University of Maryland School of Medicine, Baltimore, Maryland, United States of America; 3 Programa de Microbiología y Micología, Instituto de Ciencias Biomédicas, Facultad de Medicina, Universidad de Chile, Santiago, Chile; 4 Department of Medicine, Division of Infectious Diseases, Washington University School of Medicine, Saint Louis, Missouri, United States of America; 5 Veterans Affairs Medical Center, Saint Louis, Missouri, United States of America; 6 Instituto Milenio de Inmunología e Inmunoterapia, Facultad de Medicina, Universidad de Chile, Santiago, Chile; University of Texas Medical Branch, UNITED STATES

## Abstract

Enterotoxigenic *Escherichia coli* (ETEC) is one of the most common diarrheal pathogens in the low- and middle-income regions of the world, however a systematic examination of the genomic content of isolates from Chile has not yet been undertaken. Whole genome sequencing and comparative analysis of a collection of 125 ETEC isolates from three geographic locations in Chile, allowed the interrogation of phylogenomic groups, sequence types and genes specific to isolates from the different geographic locations. A total of 80.8% (101/125) of the ETEC isolates were identified in *E*. *coli* phylogroup A, 15.2% (19/125) in phylogroup B, and 4.0% (5/125) in phylogroup E. The over-representation of genomes in phylogroup A was significantly different from other global ETEC genomic studies. The Chilean ETEC isolates could be further subdivided into sub-clades similar to previously defined global ETEC reference lineages that had conserved multi-locus sequence types and toxin profiles. Comparison of the gene content of the Chilean ETEC identified genes that were unique based on geographic location within Chile, phylogenomic classifications or sequence type. Completion of a limited number of genomes provided insight into the ETEC plasmid content, which is conserved in some phylogenomic groups and not conserved in others. These findings suggest that the Chilean ETEC isolates contain unique virulence factor combinations and genomic content compared to global reference ETEC isolates.

## Author summary

The study of enteric pathogens has progressed from examining single isolates to being able to integrate large amounts of genomic and epidemiological data. These types of analyses have allowed the identification of trends in the genomic data that would have remained unexamined with technologies that have less resolution. In the current study the genomic content of enterotoxigenic *E*. *coli* (ETEC) isolates from Chile was compared to that of previously sequenced isolates that represent the global distribution of ETEC. We identified genomic content and virulence factor combinations that are common and unique to ETEC in Chile compared to a global collection of ETEC. Completion of the extrachromosomal plasmids, which contained the majority of the virulence factors, also identified common genetic themes among ETEC from Chile. This study highlights the diversity of ETEC in Chile and provides additional avenues of research to examine these important human pathogens.

## Introduction

The pathogenic variant (pathovar) of *Escherichia coli* known as enterotoxigenic *E*. *coli* (ETEC) has been implicated in 1 billion cases of diarrhea annually [1], and recent studies, such as the Global Enteric Multicenter Study (GEMS), has further confirmed that ETEC is a significant global pathogen [2]. ETEC are defined on a molecular basis by the presence of genes that encode the heat-stable (ST) and/or heat-labile (LT) enterotoxin [3, 4]. Although a number of virulence factors identified in ETEC isolates have been investigated as potential vaccine antigens, to date no effective vaccine has been developed most likely due to antigenic variation of key virulence factors [5–8]. One goal of microbial genomic studies is to identify novel antigens that may be used as alternate vaccine targets [9, 10]; however characterization of the genomic and antigenic diversity of these pathogens is required prior to selecting these novel antigenic targets. Genome sequencing of ETEC isolates to date has revealed significant diversity not previously observed in the other *E*. *coli* pathovars [11–17]; however, previous studies have not extensively characterized the genomic diversity of Chilean ETEC isolates.

While the enterotoxins are the defining molecular and virulence features of ETEC, the majority of isolates also express one or more colonization factors (CFs), a heterologous group of antigens that promote attachment to intestinal epithelia [18, 19]. Most ETEC-specific virulence factors, including the CFs are plasmid-encoded. At least 27 known or putative CFs have been described in the literature [18, 19] and more CFs have been identified in genomic studies [20], but have yet to be functionally characterized [13, 21]. In addition to the canonical ETEC virulence factors and CFs, additional virulence factors have been identified with variable presence in ETEC isolates. Two plasmid-borne loci encode EatA, a secreted serine protease autotransporter [22], and EtpA, a glycoprotein that acts as an adhesive bridge between ETEC flagella and host surface structures [23], seem to be more broadly conserved in ETEC than previously identified putative virulence factors [23, 24]. While functional characterization of the contribution of EatA and EtpA ETEC virulence is ongoing, EatA appears to accelerate toxin delivery by degrading MUC2, the major mucin secreted by gastrointestinal goblet cells [25], and EtpA modulates adhesion to blood group antigens [26, 27]. Additionally, both EtpA and EatA are immunogenic in humans [28] and have been demonstrated to be protective antigens in animal models [25, 29, 30].

The advent of whole genome sequencing has opened the possibility of examining large numbers of ETEC genomes from isolates collected over time from different geographic locations to increase our understanding of the dynamic nature of these organisms. Until 2014, there were fewer than 10 sequenced and assembled human-associated ETEC isolates available in Genbank, and all were from symptomatic patients, [11, 12, 14]. However, recent studies by von Mentzer *et al*. [15], Del Canto *et al*. [21] and Sahl *et al*. [13] as well as others [16, 17, 31] have greatly expanded the genomic knowledge of ETEC as a pathovar, by including ETEC from various global geographic locations and clinical presentations. While there have been a number of molecular studies of the ETEC in Chile [24, 32–36], only a limited number of genomes of Chilean ETEC isolates have been examined [21]. The current study examines a collection of 125 diarrhea associated ETEC isolates from three geographic locations in Chile to begin to address the gap in our knowledge of the genomic and virulence factor diversity of ETEC in Chile. This study also takes advantage of the ability to compare traditional PCR and other typing assays with *in silico* analyses based on the whole genome sequencing.

## Methods

### Bacterial isolates and media

The ETEC isolates examined in this study were obtained via targeted ETEC surveillance at three locations in Chile: Santiago (88 isolates), Antofagasta (31 isolates) and Calama (6 isolates). All isolates were determined by PCR to be either ETEC LT or ST positive using a previously validated PCR assays [37]. The isolates were grown, with minimal passage, in Lysogeny Broth (LB)[38](Difco) for genomic DNA isolation and propagation.

### Genome sequences

The genomes of the isolates were generated, sequenced and analyzed as previously described [39]. The 150bp sequencing reads from the Illumina platform [39] were assembled using spades v.3.7.1 with careful mismatch correction [40] and the assemblies were filtered to contain only contigs ≥500bp with ≥5X k-mer coverage [41]. The assemblies were further examined for characteristics that would suggest the genome was of high quality (<400 contigs) and potentially *E*. *coli* (%GC ~ 50% and genome size between 4.7–5.4 Mb). The assembly metrics and corresponding GenBank accession numbers are provided in S1 Table. Four isolates were further sequenced with Pacific Biosciences (PacBio) to complete the genomes as representatives of isolates from Chile, as well as to completement the existing genome references. PacBio raw data was corrected and assembled as previously described [42, 43] The final assembly statistics for these genomes are included in S1 Table.

### Multilocus sequence typing and serotype identification

The seven loci (*adk*, *gyrB*, *fumC*, *icd*, *mdh*, *purA*, and *recA*) of the multilocus sequence typing (MLST) scheme developed by Wirth *et al*. [44] were identified and compared with the database maintained by the University of Warwick (http://mlst.warwick.ac.uk/mlst/dbs/Ecoli). The MLST gene sequences extracted from each genome were used to query the BIGSdb database [45] to obtain the allele numbers and sequence type of each ETEC genome analyzed. *In silico* serotype identification was performed on the assembled genomes using the online SerotypeFinder 1.1 (https://cge.cbs.dtu.dk/services/SerotypeFinder/).

### Phylogenomic analysis

The genomes of the ETEC isolates analyzed in this study were compared with 73 previously sequenced *E*. *coli* and *Shigella* genomes (S2 Table) using the *In Silico* Genotyper (ISG) [46, 47] as previously described [41–43]. Single nucleotide polymorphisms (SNPs) were detected relative to the completed genome sequence of the phylogroup F laboratory isolate *E*. *coli* IAI39 (NC_011750.1). A total of 221,978 conserved SNP sites, which were present in all of the genomes analyzed, were concatenated into a representative sequence for each genome. A maximum-likelihood phylogeny was inferred using the GTR model of nucleotide substitution with the GAMMA model of rate heterogeneity, and 100 bootstrap replicates, and visualized using FigTree v1.4.2 (http://tree.bio.ed.ac.uk/software/figtree/).

### Comparisons of genome content

The genomes of the 73 reference *E*. *coli* isolates and the 125 Chilean ETEC isolates (S1 Table) were compared *de novo* using Large Scale-BLAST Score Ratio (LS-BSR) analysis [39, 48]. The resulting output was used to identify genes that exhibited an altered distribution among the isolates that were examined. The LS-BSR values and the nucleotide sequences of each gene cluster for the 125 new ETEC Chilean isolates are included in Supplemental Data Set 1.

The presence or absence of the ETEC virulence genes were examined using BLAST Score Ratio [49] as previously described [13, 39, 41, 50, 51]. The genes that were detected with a BSR ≥0.8 were considered to be present in a genome as previously described [13, 39, 41, 50, 51].

### Phylogenetic analysis of ST genes

The ST genes from each ETEC genome were compared with previously described *estA* reference sequences [52]. The *estA* nucleotide sequences were aligned using ClustalW and a phylogeny was constructed using the maximum-likelihood method with the Kimura 2-parameter model and 1,000 bootstraps using MEGA7 [53].

### In silico detection of ETEC virulence plasmids

Plasmids in each of the complete genomes were annotated using an in-house annotation pipeline with gene prediction using Prodigal [54–56]. The predicted protein-coding genes of select plasmids were detected in each of the ETEC genomes using BLASTN LS-BSR. Heat maps were generated using the heatmap2 function of gplots v. 3.0.1 in R v.3.3.2 and were clustered using the complete linkage method with Euclidean distance estimation.

### Identification of EtpA and EatA by immunoblotting

Supernatants of overnight bacterial cultures were precipitated with trichloroacetic acid (TCA) as previously described [23] and detected via Western blot as previously described for EatA [22] or EtpA [30].

### Identification of *etpA*, *etpB* and *eatA* by polymerase chain reaction

Isolates encoding *eatA*, *etpA* or *etpB* were identified by PCR as previous described [23, 33]. The resulting amplicon was electrophoresed on a 1% gel and visualized. A strain was positive when an amplicon of the appropriate size was visualized.

## Results

### ETEC isolates

The ETEC isolates were obtained from three hospitals in Chile and were determined to be ETEC based on the presence of at least one of the heat-labile (LT) or heat-sable toxins (STh or STp) at the location of isolation. Of the examined isolates, 37 were obtained from a recent ETEC outbreak in the Antofagasta region of Chile in the cities of Antofagasta and Calama [24] [57]. The remaining 88 isolates are from an ETEC collection maintained in the Vidal Lab that was obtained from an ETEC targeted surveillance study in the community of Santa Julia (Santiago city).

### Genome characteristics of isolates

The assembled ETEC genomes on average contained 203 ± 53 contigs. The mean genome size and %GC were 5,036,883 ± 140,015 bp (range 4,708,441–5,479,008 bp) and 50.56 ± 0.13 (range 50.02–50.83), respectively, which are both well within the normal variation of *E*. *coli* genome size and %GC. Further analysis was carried out on these assembled genomes, with the exception of four isolates that underwent additional PacBio sequencing to complete their genome assemblies, in which case the complete genomes were used in the analysis (Table 1).

**Table 1:** In silico determined characteristics of the ETEC genomes selected for complete genome sequencing.

Strain	Contig Description	Sequence Length (bp)	GC-content (%)	Completion Level	Contig Name	Plasmid Replicon	Virulence Genes	Accession Nos.
10754_a_1	chromosome	4,897,493	50.72	not circular	10754a1_chromosome	NA	T6SS, *fyuA*, *irp2*, *tibA*, *tia*	CP025976
	plasmid 1	92,477	46.77	circular	10754a1_p10754a1_92	IncFII	STh, *rns* (2 copies), CFA/I, *eatA*, *etpA*	CP025977
	plasmid 2	46,623	45.9	circular	10754a1_p10754a1_46	IncFIB(AP001918)	CS21-like	CP025978
10802_a	chromosome	4,872,344	50.71	circular	10802a_chromosome	NA	T2SS, T6SS, *tia*, *tibA*, *irp2*, *fyuA*	CP025973
	plasmid 1	92,479	46.77	circular	10802a_p10802a_92	IncFII	STh, CFA/I, *eatA*, *rns*	CP025974
	plasmid 2	46,623	45.9	circular	10802a_p10802a_46	IncFIB(AP001918)	CS21-like	CP025975
11573_a_1	chromosome	4,902,738	50.72	not circular	11573a1_chromosome	NA	T2SS, *tia*, *irp2*, *fyuA*, *tibA*	CP025970
	plasmid 1	92,481	46.77	circular	11573a1_p11573a1_92	IncFII	STh, *rns*, CFA/I, *eatA*, *etpA*	CP025971
	plasmid 2	46,623	45.9	circular	11573a1_p11573a1_46	IncFIB(AP001918)	CS21-like	CP025972
2407_a	chromosome	4,941,120	50.89	circular	2407a_chromosome	NA	T2SS, T6SS	CP025967
	plasmid 1	135,437	48.57	circular	2407a_p2407a_135	IncFII	STh, CS6, CS5, *eatA*, *peaR*	CP025968
	plasmid 2	9,863	49.15	circular	2407a_p2407a_9	unknown	none	CP025969

### Phylogenomic analysis of Chilean ETEC isolates

**Fig 1:**
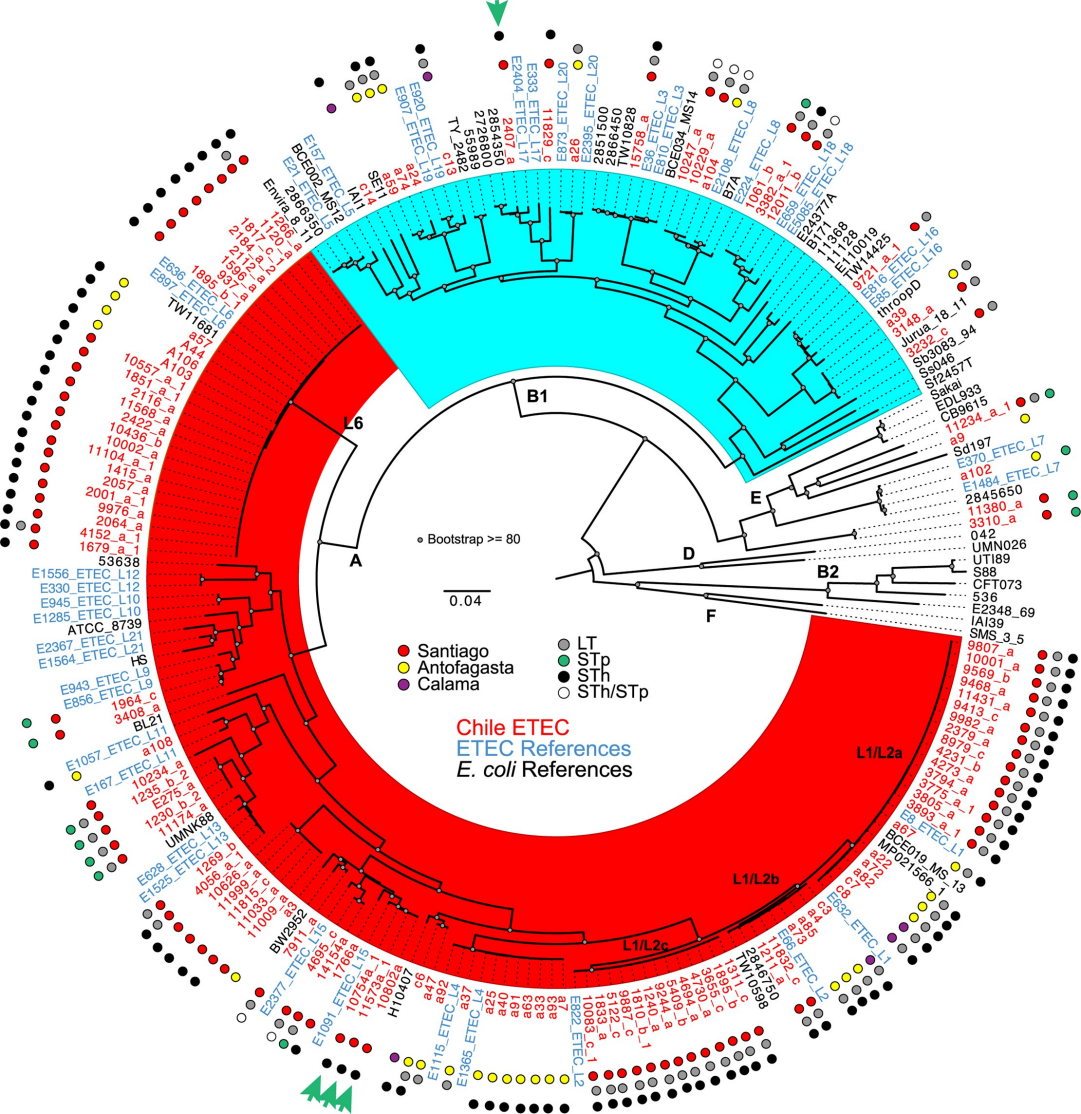
Phylogenomic analysis of the Chilean ETEC isolates. The whole-genome sequences of the Chilean ETEC isolates were compared with previously sequenced *E*. *coli* and *Shigella* genomes listed in S2 Table using a single nucleotide polymorphism (SNP)-based approach as previously described [46, 47]. SNPs were detected relative to the completed genome sequence of the laboratory isolate *E*. *coli* IAI39 using the *I**n* *S**ilico* Genotyper (ISG) [47]. A total of 221,978 conserved SNP sites, which were present in all of the genomes analyzed, were concatenated into a representative sequence for each genome. A maximum-likelihood phylogeny with 100 bootstrap replicates was inferred using RAxML v.7.2.8 [72]. Isolates designated in red are from Chile, isolates designated in blue are the ETEC lineage references identified in von Mentzer et al [15], and isolates designated in black are reference *E*. *coli* and *Shigella* isolates representing other pathotypes and phylogenomic groups. The letters (A, B1, B2, D, E, and F) designate the *E*. *coli* and *Shigella* phylogroups that were previously defined [73, 74]. Colored circles indicate the country of origin on the inner ring and the heat labile toxin (LT) and heat stable toxin (ST) status on the middle and outer rings respectively. The green arrows indicate the genomes that were completed using Pacific Biosciences sequencing. The scale bar represents the distance of 0.04 nucleotide substitutions per site.

The genomes of the Chilean ETEC isolates were compared to selected draft and complete reference ETEC genomes of ETEC from a global collection representing defined abundant ETEC lineages L21L10 [15], a collection of previously sequenced ETEC from Bangladesh [13], and reference *E*. *coli* that are commonly used to define the *E*. *coli* pathovars and phylogenomic groups [39, 50]. The ETEC L1 to L21 lineage reference isolates were selected for comparison based on the dominant MLST sequence types, serotypes and colonization factor profiles from von Mentzer, et. al. [15]. The phylogenomic analysis revealed that 80.8% (101/125) of the Chilean ETEC isolates are in phylogroup A (red highlighting), 15.2% (19/125) in phylogroup B1 (blue highlighting) and 4.0% (5/125) are in phylogroup E (Fig 1). Of the 101 Chilean ETEC isolates in phylogroup A, 27 are in Lineage L6, 40 are in lineage L1/L2 and the remaining 34 are distributed in smaller groups some with lineage references and others as phylogenomic singletons (Fig 1). When further molecular studies of these ETEC genomic lineages are undertaken, it is clear that these lineages represent expansions of successful clones, as all the isolates in Lineage L6 are also ST3223, and 25/27 (92.6%) contain only the STh toxin, with the remaining isolates (2/27) contain both LT and STh (Fig 1 and S1 Table). This relative expansion of the phylogroup A isolates in Chile is not due to the examination of a localized outbreak as there are isolates from each of the three geographic sites in Lineage L6. In other genomic studies ETEC from phylogroup B1 and A are in similar proportions [13–15, 58]. Additionally, the phylogroup A Lineage L1/L2 also contains isolates from each of the three geographic sites, which were all determined to be LT and STh positive (Fig 1, S1 Table). By examining the phylogeny in greater detail, it becomes evident that the L1/L2 isolates in phylogroup A can be split into three subclades named L1/L2a, L1/L2b and L1/L2c (Fig 1). Within these subclades L1/L2c isolates were all ST3854, L1/L2b were all ST4 and L1/L2a were a mixture of ST4, ST2353, and undetermined sequence types. In phylogroup B1 (blue highlighting) there was limited clustering of Chilean ETEC isolates within any one lineage, with between one to four isolates in seven different lineages (L3, L8, L16, L17, L18, L19, and L20) (Fig 1).

### Complete genome sequencing of Chilean ETEC isolates

**Fig 2:**
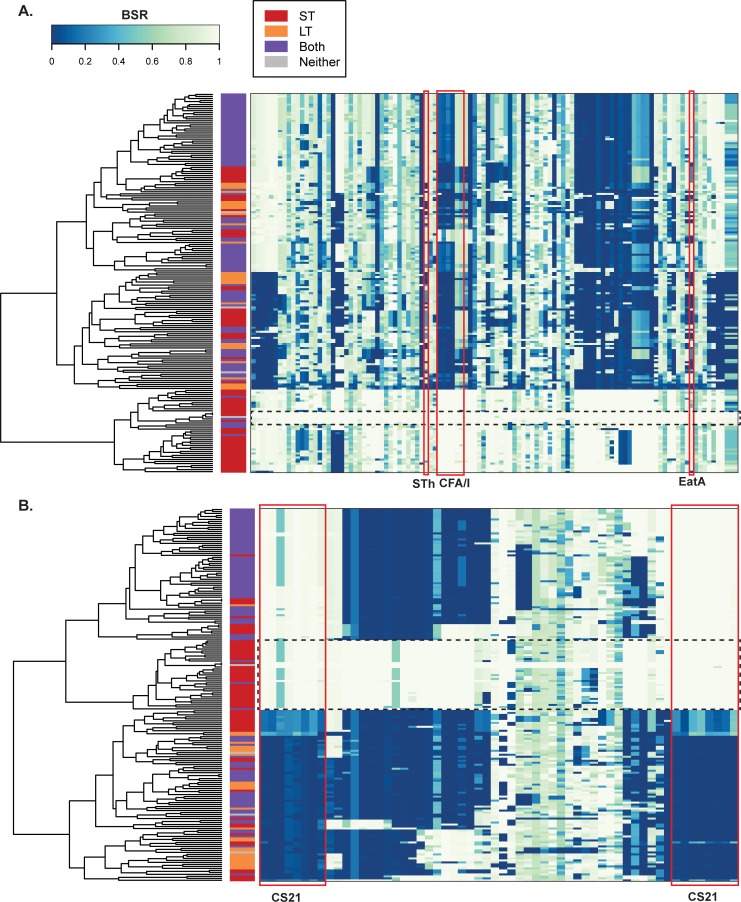
Distribution of virulence plasmids among the Chilean ETEC isolates. Heat maps indicate the presence of the virulence plasmids **A)** p10802a_92 and **B)** p10802a_46 among the Chilean ETEC and reference ETEC isolates analyzed in this study. The predicted protein-coding genes of each plasmid were identified using BLASTN LS-BSR [48] as previously described [43]. Each row represents an individual genome that is labeled on the left side by its ETEC toxin content as having the heat labile toxin (LT), heat stable toxin (ST), both LT and ST, or neither LT nor ST. Each column represents a different protein-coding gene of the reference plasmid being compared. Virulence factors of interest are indicated by a red box. A dashed line box indicates a group of genomes that contain all or nearly all of the plasmid genes.

In addition to the draft sequences generated for the aforementioned 125 Chilean isolates, four isolates were selected for complete genome sequencing with Pacific Biosciences. Each of these isolates were selected as they are heat stable toxin containing only, which was determined in the GEMS analysis to contribute significantly to severe diarrheal disease in children under five years of age [2, 31]. Three of these isolates in phylogroup A, 10754a-1, 10802a and 11573a-1 can be considered clonal (Fig 1, green arrow), as the isolates are most closely related in the inferred phylogeny, whereas the isolate 2407-a is in phylogroup B1. For each isolate examined there is a single chromosome and 2 plasmids that contain many of the canonical ETEC virulence factors for each isolate (Table 1). The average chromosome size is 4,905,574 ±32,283 bp and the plasmids range in size from 9863 bp to 135,437 bp, with the phylogroup A isolates each containing a conserved 46,623 bp plasmid and 92,479±2 bp plasmid (Table 1) encoding the CS21-like colonization factor gene cluster, as well as the ETEC regulator, *rns* [59], or CFA/I, STh, *eatA* and *etpA* genes respectively. These two plasmids from ETEC isolate 10802-a were examined for the plasmid gene distribution among the Chilean ETEC isolates (Fig 2). These studies identified that there was a limited number of isolates that appeared to contain the majority of the plasmid genes and additional isolates contained components of the plasmids including the virulence factors, but not the complete plasmid (Fig 2). In the fourth isolate, 2407-a, a plasmid of 135,437 bp contains the STh gene, CS5 and CS6 colonization factor gene clusters, as well as *eatA* and the previously identified plasmid encoded regulator, *csvR* [60] (Table 1). An additional plasmid of 9,863 bp was identified in the 2407-a isolate but contains no known virulence genes. These plasmids were less conserved in the examined isolates suggesting that these plasmids are not common. These plasmid findings highlight the variability of the ETEC plasmids as previously described [11, 43, 61].

### Detection of toxin genes

Previous molecular studies have described a range of ETEC toxin profiles among both the global ETEC isolates as well as isolates from Chile [33, 35, 36, 62]. The *in silico* analysis failed to identify any isolates encoding STb (aka STII from K88) or variant forms of LT (IIa, IIb, IIc) [63, 64]. Of the 125 Chilean ETEC isolates, four genomes (4/125, 3.2%) were identified that contained no toxins, which is not uncommon when one considers the observed instability of the ETEC plasmids [61, 65–67]. Of the isolates with toxin genes, 10 isolates (8.3%) contained LT only, 43 isolates (35.5%) contained STh only and five additional isolates (4.1%) had STp only, 48 isolates (39.7%) contained both LT and STh, seven isolates (5.8%) contained LT and STp, and six isolates (5.0%) contained LT, STh, STp (Table 2). There were also two isolates that in the *in silico* analysis contained STh and the *eltB* gene for the LT binding subunit, but were lacking the *eltA* gene, suggesting that the LT toxin is non-functional. Comparison of the traditional PCR with the *in silico* analysis identified a 77.6% (97/125 isolates) concordance between genomic and traditional PCR-based analyses. Among the discordant isolates were 15 toxin-positive strains identified by *in silico* analysis not identified by the traditional PCR, and 13 toxin-positive isolates identified by PCR not identified in the genome assemblies. Plasmid loss is one reason why there may be an inconsistency with the PCR, but also variation of the toxins or variation of the genes and surrounding genomic regions targeted by the PCR assay.

**Table 2:** Virulence factor prevalence^a^.

	Traditional PCR		Genomics^a^	
Gene	number	%	number	%
LT-I_*eltA*_H10407	NT	NT	71	56.8
LT-I_*eltB*_H10407	71	55.5	69	55.2
STIa_STp_H10407	23	18.0	19	15.2
STIb_STh	98	76.6	99	79.2
*eatA*	97^c^	75.2	85	68.0
*etpA*	105^c^	81.4	2	1.6
*etpB*	92	71.3	92	73.6
*tia*	18	14.0	9	7.2

^a^ Prevalence as calculated by LS-BSR in genome data

NT = not tested

^c^ These samples were also tested with western blots for EatA/EtpA were performed and 86 and 91 if the isolates were positive

In addition to the presence or absence of the ST encoding gene we examined the distribution of the different ST alleles as described in Joffre et al [52]. There was no geographic variation associated with the ST alleles in the examined isolates (S1 Fig). Overall, we do not observe a geographic component to the distribution of any of the ETEC toxin genes.

### Detection of colonization factors

In addition to the toxin gene profile, the other factors that identify ETEC isolates are the colonization factors (CF), which have been the target of intense study as vaccine and therapeutic targets. As with the ETEC toxins, there are traditional PCR tests for the majority of the well characterized CFs [21, 24, 33, 37], additionally, the *in silico* analysis has been expanded by including novel CF gene clusters that have been recently identified but are not yet included in the traditional PCR assay [13]. The CF detection rates are included in Table 3, and for the majority of the CF examined by both genome analysis and PCR-based approaches there is excellent concordance between the two assays. There were no Chilean ETEC isolates that contained CS4, CS13, CS14, CS15, CS18, CS22, CS26, CS27a, CS28a, CS28b, CS31a, CS30 or the novel CF identified from TW11833 [13], nor were the CFs identified that are traditionally associated with livestock (ETEC_K88_ab, ETEC_K88ac, ETEC_K99, ETEC_f41a, ETEC_f987P, ETEC_f17a). The distribution of the CFs from the Chilean isolates was not significantly different when compared to the global distributions of the CFs in others studies [13, 15, 31, 62].

**Table 3:** Colonization factor prevalence^a^.

	Traditional PCR		Genomics^a^	
Colonization factor	number	%	number	%
CFAI	30	23.4	32	25.6
CS1	23	18.0	23	18.4
CS2	19	14.8	19	15.2
CS3	38	29.7	40	32.0
CS5	2	1.6	4	3.2
CS6	14	10.9	13	10.4
CS7	0	0.0	4	3.2
CS8	6	4.7	60	48.0
CS12	5	3.9	7	5.6
CS15	5	3.9	ND	0.0
CS17	2	1.6	3	2.4
CS19	1	0.8	3	2.4
CS20	17	13.3	4	3.2
CS21	106	82.8	81	64.8
CS23	1	0.8	6	4.8
CS27b	0	0.0	2	1.6
NT	5	3.9	6	4.8
Novel_CF_TW10509	NT	NT	4	3.2
Novel_CF_TW11786	NT	NT	1	0.8
Novel_CF_PCFO71	NT	NT	23	18.4
CFAI_variant^b^	NT	NT	14	11.2

^a^ Prevalence as calculated by LS-BSR in genome data

^b^Protein ID EMV36291.1

ND—Not detected

NT—Not tested

### Detection of non-canonical secreted antigens

In addition to whole genome sequencing the isolates were also interrogated by gene specific PCR and Western blotting for the putative vaccine candidates, EtpA [68] and EatA [25]. By PCR, the *eatA* gene was identified in 76% of isolates consistent with the genome data, which identified the gene in 68% of the isolates, and immunoblotting confirming EatA protein secretion in 72% of the isolates tested. The *etpB*, *and etpA* genes were identified in 71.9%, and 82% of isolates, respectively, with EtpA protein secretion verified in 75% of isolates tested (S1 Table). However, *etpA* was identified in the genomes of only two isolates, likely relating to difficulty in assembling the multiple repeat modules comprising the 5’ end of the gene using short read technologies [13].

### Functional characteristics of isolates

Traditional serotyping of ETEC has demonstrated that this pathovar is associated with many serotypes [69]. In the current study we have the opportunity to compare serotyping by traditional methods with an *in silico* serotyping method (https://cge.cbs.dtu.dk/services/SerotypeFinder/) (S3 Table). Of the isolates that were examined by both serotyping methodologies (n = 89), the *in silico* and traditional methods were generally congruent for 66.3% (59/89). The majority of the inconsistencies were common in the trend of discordance, in that there were common mis-identifications between the two methodologies. There were 13 isolates (14.6%) identified as O114/O127 by traditional methods, but were predicted to be O128ab/ac by *in silico* serotyping, an additional eight isolates that were identified as O153 by traditional methods, but were predicted to be non-typeable by *in silico* methods, and nine other isolates with unique predicted *in silico* serotypes with discordant serotyping results. Of the identified serotypes, the O6 was the most prevalent O serotype (n = 39, 31.2%), H16 (n = 39, 31.2%) was the most prevalent H serotype, and the O6:H16 (n = 25, 20%) was the most common combination (S3 Table). The distributions of the O and H antigens identified in the current study were similar to that of a global examination of ETEC isolates from the literature that had identified ETEC isolates of the O6:H16 serotypes as among the most prevalent [69].

### Comparison of total genome content

The total gene content of the Chilean ETEC isolates were compared using *de novo* LS-BSR. We identified 18,719 gene clusters, of which 3,806 were identified in all 125 isolates, comprising the core genome. This number of predicted genes was similar to previous estimations of the genome core of *E*. *coli* [12, 15, 70]. The LS-BSR analysis allows us to integrate the clinical parameters, location of isolation, as well as genomic factors (MLST or phylogroup) to identify genomic features that may be associated with these clinical parameters. There are no genes that are exclusive to isolates from one of the Chilean geographic locations when compared to the isolates from the other locations in Chile; however there were genes that were identified among a greater number of the ETEC isolates from certain geographic locations compared to the other geographic locations in Chile (S4 Table). We could identify 496, 456, or 94 gene clusters that were more or less prevalent among the isolates from Santiago, Antofagasta, or Calama, respectively (chi-squared test, p-value <0.0001) (S4 Table). Additionally, the examination of the Chilean ETEC isolates, as in previous studies [13–15, 58], did not identify any genes that are exclusively detected in the genomes of phylogroups A or B1; however phylogroup E had 30 genes that were identified among all members of that phylogroup and absent in all other Chilean ETEC genomes of the other phylogroups (S5 Table). Additionally, there were 1190, 978, and 896 genes that were detected in in a greater proportion among the genomes of phylogroups A, B1 or E; respectively, compared to isolates in the other phylogroups (chi-squared test, p-value <0.0001, S5 Table). These genes represent multiple biological functions, including secretion systems, as well as potential virulence factors.

## Discussion

This study describes the genomic content of ETEC isolates from three geographic locations within Chile. While other molecular studies have focused on the virulence and/or colonization factor distribution among the ETEC isolates in Chile [33, 36, 37], this is the first study to incorporate large-scale comparative genomics analyses. The distribution of the canonical virulence factors, including the CF types was not significantly different when compared to previous global studies [13, 15, 31, 62]. However, the most striking finding from the study is that the majority of the Chilean ETEC isolates are within phylogroup A, and within that phylogroup multiple ETEC lineages could be readily identified that have similar toxin, MLST and phenotypic profiles, suggesting that these clones have been successful and have expanded within Chile (Fig 1). In each of the sub-lineages within phylogroup A there is representation from each of the three geographic locations, suggesting that one geographic location or clinical presentation is not driving the observed difference. Additionally, we were able to compare the Chilean isolates to reference ETEC lineages identified by von Mentzer et al. [15], and in each case where Chilean isolates formed a group with the lineage reference isolate(s) they shared similar molecular attributes such as toxin profile, colonization factor or MLST sequence type, suggesting that these are successful global ETEC lineages, which have expanded in Chile. For example, ETEC lineage L6 was identified by von Mentzer et al. [15], which contained only seven L6 isolates from a collection of 462 isolates (7/462, 1.5%), none of which were from Chile or South America, whereas in the current collection of 125 Chilean ETEC isolates, 27 isolates were identified in Lineage L6 (27/125, 21.6%). This represents a 14.4 fold greater detection of the ETEC L6 isolates in Chile compared to the rest of the world (Chi squared p-value < 2.2e-16). The reasons behind the increased occurrence of this particular ETEC lineage in Chile is not clear and the current study focuses only on the pathogen attributes; however there are many factors to consider when thinking about the host pathogen interaction, including the host genome, as well as nutritional status and the microbiota of the individual, all of which may play a role in the clinical outcome.

The representative ETEC isolates selected for genome completion by PacBio sequencing in the current study were from the major ETEC phylogroups (B1 and A), as well as containing the ST toxin genes, and they complement the complete genomes of the ETEC prototypes [11, 12] and recent isolates [16, 17, 31, 43]. Interestingly, the three phylogroup A isolates selected for complete genome sequencing were very closely related based on the phylogeny (Fig 1), and each isolate has the same plasmid profile, and a very similar virulence factor profile (Table 1). While the early studies of ETEC plasmid diversity indicated that the plasmid content was extremely variable [13, 14, 60, 61, 67, 71], more studies [31], including the current study indicate the presence of stable combinations of ETEC chromosome and plasmids. The reasons for the stability of these combinations and instability of other chromosome:plasmid combinations is not clear and will require further functional analyses to examine in detail.

Overall, this study demonstrates the utility of combining whole genome sequencing with the isolate and laboratory molecular data to provide a detailed view of the pathogen distribution within a country or geographic region. We have determined that the Chilean ETEC isolates primarily are present in phylogroup A at an almost four fold greater proportion when compared to a global study of ETEC [15], whereas ETEC from other geographic locations are more distributed between phylogroups A and B1. How this impacts clinical outcomes or ETEC transmission is not yet clear, but provides a foundation to build upon for future studies to examine host genomics or other environmental factors that may be important in the study of this human pathogen.

## Supporting information

S1 Fig Phylogenetic analysis of STh and STp. Nucleotide sequences of STh and STp genes were aligned with the *estA1-estA7* reference sequences and used to infer a maximum likelihood phylogeny as previously described [43]. Labels of the Chilean ETEC isolates are colored by their location (see inset legend), while labels of reference ETEC are indicated in black. The *estA1-estA7* reference sequences [52] are indicated in bold. The tree scale indicates the distance of 0.04 nucleotide substitutions per site. Bootstrap values ≥50 are indicated by gray circles.
(EPS)

S1 Table Isolate and genome information. (PDF)

S2 Table Reference genomes and corresponding pathotypes. (PDF)

S3 Table O and H antigen determination in Chile ETEC isolates. (PDF)

S4 Table Distribution by geography. (PDF)

S5 Table Distribution by phylogroup. (PDF)
