# Supplementary material for: Comparative genomic analysis and molecular examination of the diversity of enterotoxigenic Escherichia coli isolates from Chile
Source: PLoS Negl Trop Dis. 2019 Nov 20;13(11):e0007828. doi: 10.1371/journal.pntd.0007828 (PMC6901236; doi:10.1371/journal.pntd.0007828)
Supplement: S1 Table — (PDF) [file pntd.0007828.s002.pdf]

Isolate		Molecular defined data			Genomically defined data					PCR amplification			WesternBlot					
Original Code	***Origin***	***Serogroup***	***Toxins***	***CFs***	***Phylogroup***	***MLST (CG)***	***Serogroup***	***Toxin***	***CFs***	***eatA***	***etpA***	***etpB***	**EtpA**	**EatA**	**No. Contigs**	**Genome size**	**GC**	**N50**
10001 a	Santiago	O6	LT, STh	CS1, CS3	A	ND	O6:H-	LT, STh	CS1, CS3, pfc071	1	1	1	1	1	220	4891845	50.52	72429
10002 a	Santiago	O104,O127	STh	CFAI, CS21	A	2332	O128ab/ac:H45	STh	CFAI, CS21	1	1	1	1	1	218	5116806	50.7	100075
10083 c-1	Santiago	O6	LT, STh	CS2, CS3, CS21	A	3854	O6:H16	LT, STh	CS2, CS3, CS21	1	1	0	0	1	218	4864323	50.45	81249
10229 a	Santiago	O148	LT, STp, STh	CS6, CS20, CS21	B1	1988	O148:H28	LT, STp, STh	CS6, CS21	1	0	0	0	0	154	4945065	50.69	97611
10234 a	Santiago	ONT	LT	ND	A	6335	ONT:H2	LT, STh	nCF-TW11786, CFAI-var	1	0	0	0	1	216	5297795	50.57	127346
10247 a	Santiago	O148	LT, STp, STh	CS6, CS21	B1	1988	O148:H28	LT, STp, STh	CS6, CS21	0	0	0	ND	ND	188	5023977	50.54	111110
10436 b	Santiago	O104,O127	STh	CFAI, CS21	A	2332	O128ab/ac:H45	STh	CFAI, CS21	1	1	1	1	1	316	5286180	50.6	86511
10557 a-1	Santiago	O49	ND	CFAI, CS21	A	2332	O128ab/ac:H45	STh	CFAI, CS21	1	1	1	1	1	206	5167107	50.64	102633
1061 b	Santiago	O23	LT, STp	CS20, CS21	B1	453(86)	O23:H16	LT, STp	CS20, TW10509, CFAI-var	1	0	0	0	1	176	5023865	50.52	98097
10626 a	Santiago	ONT	STh	CS21	A	1564	ONT:H21	STh	CFAI	0	1	1	1	0	235	4955724	50.66	86727
10754 a-1	Santiago	O49	STh	CFAI, CS21	A	10(10)	O49:H12	STh	CFAI,CS21	1	1	1	1	1	3	5,036,593	50.6	4897493
10802 a	Santiago	O49	STh	CFAI, CS21	A	10(10)	O49:H12	STh	CFAI,CS21	1	1	1	1	1	3	5,011,446	50.6	4872344
11009 a	Santiago	ONT	STh	CS21	A	1564	ONT:H21	ND	NT	0	1	1	1	0	170	4760038	50.78	90765
11033 a	Santiago	ONT	STh	CS21	A	1564	ONT:H21	STh	NT	0	1	1	1	0	215	4924288	50.66	87067
11104 a-1	Santiago	O104,O127	STh	CFAI, CS21	A	2332	O128ab/ac:H45	STh	CFAI, CS21	1	1	1	1	1	193	5131549	50.71	102633
11174 a	Santiago	O80	LT, STp	CS20, CS21	A	4121	O80:H9	LT, STp	CS12, CFAI-var	1	1	0	0	0	157	5049466	50.59	121254
1120 a	Santiago	O153	STh	CFAI,CS21	A	2332	ONT:H45	LT*, STh	CFAI, CS21	1	1	1	1	1	173	5119763	50.79	118108
11234 a-1	Santiago	O27,O103	LT, STp	CS20, CS21	E	57(350)	O27:H10	LT, STp	CS20, TW10509, CFAI-var	1	1	0	0	1	180	5217811	50.5	152021
11380 a	Santiago	O169	STp	CS6, CS20, CS21	E	6591	O169:H25	STp	CFAI, CS6	1	1	0	0	0	269	5186122	50.19	75800
11431 a	Santiago	O6	LT, STh	CS1, CS3, CS21	A	ND	O6:H-	LT, STh	CS1, CS3, CS21, pfc071	1	1	1	1	1	233	4973536	50.41	69545
11568 a	Santiago	O104,O127	STh	CFAI, CS21	A	2332	O128ab/ac:H45	STh	CFAI, CS21	1	1	1	1	1	199	5122821	50.69	102726
11573 a-1	Santiago	O49	STh	CFAI, CS20, CS21	A	10(10)	O49:H12	STh	CFAI, CS21	1	1	1	1	1	3	5,041,842	50.6	4902738
11815 c	Santiago	O104,O127	STh	CS21	A	1564	ONT:H21	STh	CS5, CS6, CS7	0	1	1	1	0	222	4850642	50.76	87026
11829 c	Santiago	O104,O127	STh	CS5, CS6, CS21	B1	7	O128ab:H21	STh	CS5, CS6, CS7	1	1	0	0	1	222	5050917	50.61	97832
11832 c	Santiago	O6	LT, STh	CS2, CS3, CS21	A	4(10)	O6:H16	LT, STh	CS2, CS3, CS21	1	1	0	1	1	196	4789762	50.52	85449
11999 a	Santiago	ONT	STh	CS21	A	1564	ONT:H21	STh	NT	0	1	1	1	0	231	4902187	50.7	87062
12011 b	Santiago	O23	STp	CS20, CS21	B1	453(86)	O23:H16	LT*, STh	CS20, CS31a, CFAI-var	1	1	1	1	0	216	5110856	50.34	93629
1211 a	Santiago	O6	LT, STh	CS2, CS3, CS20, CS21	A	4(10)	O6:H16	LT, STh	CS2, CS3, CS21	1	1	1	1	1	207	4794935	50.51	71911
1230 b-2	Santiago	O80	LT, STp	CS12, CS20	A	4121	O80:H9	LT, STp	CS12, CFAI-var	0	1	0	0	0	155	5000858	50.59	127670
1235 b 2	Santiago	O80	LT, STp	CS12, CS20	A	4121	O80:H9	LT, STp	CS12, CFAI-var	0	0	0	ND	ND	183	5071970	50.53	100327
1240 a	Santiago	O6	LT, STh	CS2, CS3, CS21	A	3854	O6:H16	LT, STh	CS2, CS3, CS21	1	0	0	0	1	236	4939026	50.58	81075
1266 a	Santiago	O153	STh	CFAI,CS21	A	2332	ONT:H45	STh	CFAI, CS21	1	1	1	1	1	207	5220469	50.79	102633
1269 b	Santiago	O141	LT	ND	A	750	O78:H32	LT	CS1, CS27a, pfc071, CFAI-var	0	1	0	0	0	370	5473241	50.26	55549
1311 c	Santiago	O6	LT, STh	CS2, CS3, CS21	A	3854	O6:H16	LT, STh	CS2, CS3, CS21	1	1	1	1	1	237	4968497	50.55	81075
1415 a	Santiago	O104,O127	STh	CFAI,CS21	A	2332	O128ab/ac:H45	STh	CFAI, CS21	1	1	1	1	1	220	5125279	50.73	100956
14154a	Santiago	O159	LT, STp	CS12, CS20	A	10(10)	O159:H4	LT, STp	CS12, CFAI-var	0	0	0	ND	ND	156	4923342	50.59	94616
15758 a	Santiago	O78	LT	CS5	B1	173	O78:H10	LT, STh	CS5, CS7	1	1	1	1	1	233	5262813	50.52	164438
1596 a	Santiago	O153	STh	CFAI,CS21	A	2332	ONT:H45	STh	CFAI, CS21	1	1	1	1	1	188	5151107	50.75	118628
1679 a-1	Santiago	O104,O127	STh	CFAI,CS21	A	2332	O128ab/ac:H45	STh	CFAI, CS21	1	1	1	1	1	206	5129139	50.73	100093
1766a	Santiago	O4	LT, STh	CS23	A	10(10)	O4:H16	LT, STh	CS23	0	0	0	ND	ND	216	5057783	50.42	147479
1810 b-1	Santiago	O6	LT, STh	CS2, CS3	A	3854	O6:H16	LT, STh	CS2, CS3	1	1	1	1	1	239	4973442	50.61	71730
1817 c-1	Santiago	O153	STh	CFAI,CS21	A	2332	ONT:H45	STh	CFAI, CS21	1	1	1	1	1	203	5167839	50.77	118182
1833 a	Santiago	O6	LT, STh	CS2, CS3, CS20	A	3854	O6:H16	LT, STh	CS2, CS3, CS21	1	1	1	1	1	238	5017244	50.57	71662
1851 a-1	Santiago	O104,O127	STh	CFAI,CS21	A	2332	O128ab/ac:H45	STh	CFAI, CS21	1	1	1	1	1	213	5120737	50.7	102726
1895 b	Santiago	O6	LT, STh	CS2, CS3, CS21	A	3854	O6:H16	LT, STh	CS2, CS3, CS21	1	1	0	1	1	232	5010379	50.57	71156
1895 b-1	Santiago	O153	STh	CFAI, CS21	A	2332	ONT:H45	STh	CFAI, CS21	1	1	1	1	1	235	5119282	50.62	126044
1964 c	Santiago	O27	STp	CS6, CS21	A	398(398)	O27:H20	STp	CS6	1	1	0	0	0	204	5085070	50.56	118423
2001 a-1	Santiago	ONT	STh	CFAI, CS21	A	2332	O128ab/ac:H45	STh	CFAI, CS21	1	1	1	1	1	203	5125503	50.7	102632
2057 a	Santiago	O104,O127	STh	CFAI, CS21	A	2332	O128ab/ac:H45	STh	CFAI, CS21	1	1	1	1	1	219	5112784	50.7	118394
2064 a	Santiago	O104,O127	STh	CFAI, CS21	A	2332	O128ab/ac:H45	STh	CFAI, CS21	0	1	1	1	1	237	5110625	50.69	102633
2112 a	Santiago	O153	STh	CFAI, CS21	A	2332	ONT:H45	STh	CFAI, CS21	1	1	1	1	1	197	5242107	50.62	118254
2116 a	Santiago	O115,O152	STh	CFAI, CS21	A	2332	O128ab/ac:H45	STh	CFAI, CS21	1	1	1	1	1	231	5140957	50.65	102633
2184 a-2	Santiago	O153	STp, STh	CFAI, CS21	A	2332	ONT:H45	STh	CFAI, CS21	1	1	1	1	1	194	5256980	50.7	118852
2379 a	Santiago	O6	LT, STh	CS1, CS3, CS21	A	ND	ND	LT, STh	CS1, CS3, CS21, pfc071	1	1	1	1	1	226	4911126	50.5	68753
2407 a	Santiago	ONT	STh	CS6, CS21	B1	278(278)	O115:H40	STh	CS5, CS6, CS7	1	0	0	0	1	3	5,086,420	50.83	ND
2422 a	Santiago	O104,O127	STh	CFAI, CS21	A	2332	O128ab/ac:H45	STh	CFAI, CS21	1	1	1	1	1	236	5167601	50.66	102632
3148 a	Santiago	O8,O127	LT	CS17	B1	423(23)	O8:H9	LT	CS17, CS19	1	1	1	1	1	160	5029132	50.57	135437
3232 c	Santiago	O8,O127	LT	CS19	B1	423(23)	O8:H9	LT	CS17, CS19	1	1	1	1	1	209	5133675	50.57	111389
3310 a	Santiago	O169	STp	CS6, CS21	E	6591	O169:H25	STp	CS6	1	1	0	0	0	205	4894394	50.34	92506
3382 a-1	Santiago	O23	LT, STp	CS20, CS21	B1	453(86)	O23:H16	LT, STp, STh	CS20, TW10509, CFAI-var	1	1	1	1	0	183	4959162	50.43	95993

3408 a	Santiago	O27	STp	CS6, CS21	A	398(398)	O27:H20	STp	CS6	0	0	0	ND	ND	195	5073550	50.56	118423
3655 c	Santiago	O6	LT, STh	CS2, CS3, CS21	A	3854	O6:H16	LT, STh	CS2, CS3, CS21	1	1	1	1	1	231	5001450	50.57	73708
3775 a-1	Santiago	ONT	LT, STh	CS1, CS21	A	ND	O6:H-	LT, STh	CS1, CS21, pcf071	1	1	1	1	1	208	4844591	50.52	72280
3794 a	Santiago	O6	LT, STp, STh	CS1, CS3, CS20, CS21	A	ND	O6:H-	LT, STh	CS1, CS3, CS21, pcf071	1	1	1	1	1	232	4915037	50.46	69545
3805 a	Santiago	O6	LT, STh	CS1, CS3, CS21	A	ND	O6:H-	LT, STh	CS1, CS3, CS21, pcf071	1	1	1	1	1	220	4915860	50.47	80373
3893 a-1	Santiago	O6	LT, STh	CS1, CS3, CS20, CS21	A	ND	O6:H-	LT, STh	CS1, CS3, CS21, pcf071	1	1	1	1	1	222	4915030	50.48	72164
4056 a-1	Santiago	O141	LT	ND	A	750	ONT:H32	LT	CS27b, CFA-var	0	1	0	0	0	180	5073162	50.45	106493
4152 a-1	Santiago	O104,O127	STh	CFAI, CS21	A	2332	O128ab/ac:H45	LT, STh	CFAI,CS21	1	1	1	1	1	197	5103264	50.71	102633
4231 b	Santiago	O6	LT, STh	CS1, CS3, CS21	A	ND	O6:H-	LT, STh	CS1, CS3, CS21, pcf071	1	1	1	1	1	208	4909767	50.49	69531
4273 a	Santiago	O6	LT, STh	CS1, CS3, CS21	A	ND	O6:H-	LT, STh	CS1, CS3, CS21, pcf071	1	1	1	1	1	217	4912107	50.5	67928
4694 a	Santiago	O6	LT, STh	CS2, CS3, CS21	A	3854	O6:H16	LT, STh	CS2, CS3, CS21	1	1	0	0	1	204	4888247	50.52	73708
4695 c	Santiago	O159	LT, STp	CS12, CS21	A	10(10)	O159:H4	LT, STp, STh	CS12, CFAI-var	0	0	0	ND	ND	158	4980666	50.56	94686
4730 a	Santiago	O6	LT, STh	CS2, CS3, CS21	A	3854	O6:H16	LT, STh	CS2, CS3, CS21	1	1	1	1	1	223	5008346	50.57	73951
5122 c	Santiago	O6	LT, STh	CS2, CS3, CS21	A	3854	O6:H16	LT, STh	CS2, CS3, CS21	1	1	1	0	1	222	4977009	50.48	71822
5409 b	Santiago	O6	LT, STh	CS2, CS3, CS21	A	3854	O6:H16	LT, STh	CS2, CS3, CS21	1	1	1	1	1	244	5169962	50.45	67396
7911a	Santiago	O16-O44	STp	CS20, CS21	A	10(10)	O16:H48	LT, STp, STh	CS21, CS23	0	0	0	ND	ND	235	4985721	50.39	71740
8979 c	Santiago	O6	LT, STh	CS1, CS3, CS21	A	ND	O6:H-	LT, STh	CS1, CS3, CS21, pcf071	1	1	1	1	1	210	4925299	50.48	69545
9244 a	Santiago	O6	LT, STh	CS2, CS3, CS21	A	3854	O6:H16	LT, STh	CS2, CS3, CS21	1	1	1	1	1	230	5007353	50.57	74228
937 a	Santiago	O153	STh	CFAI,CS21	A	2332	ONT:H45	STh	CFAI, CS21	1	1	1	1	1	206	5191363	50.81	118444
9413 c	Santiago	O6	LT, STh	CS1, CS3, CS21	A	ND	O6:H-	LT, STh	CS1, CS3, CS21, pfc071	1	1	1	1	1	204	4878912	50.46	69545
9468 a	Santiago	O6	LT, STh	CS1, CS3	A	ND	O6:H-	LT, STh	CS1, CS3, pfc071	1	1	1	1	1	212	4894323	50.51	69545
9569 b	Santiago	O6	LT, STh	CS1, CS3, CS21	A	ND	O6:H-	LT, STh	CS1, CS3, CS21, pfc071	1	1	1	1	1	234	4918801	50.46	69545
9721 a-1	Santiago	O78	LT	CS1	B1	23(23)	O78:H10	LT	CS1, pfc071	1	1	1	1	1	140	4796064	50.61	95194
9807 a	Santiago	O6	LT, STh	CS1, CS3, CS21	A	ND	O6:H-	LT, STh	CS1, CS3, CS21, pfc071	1	1	1	1	1	214	4922478	50.48	70353
9887 c	Santiago	O6	LT, STh	CS1, CS3, CS21	A	3854	O6:H16	LT, STh	CS2, CS3, CS21	1	1	1	1	1	227	4968463	50.56	71730
9976 a	Santiago	O104,O127	STh	CFAI, CS21	A	2332	O128ac:H45	STh	CFAI, CS21	1	1	1	1	1	201	5116807	50.71	102633
9982 a	Santiago	O6	LT, STh	CS1, CS3, CS21	A	ND	O6:H-	LT, STh	CS1, CS3, CS21, pfc071	1	1	1	1	1	216	4927557	50.48	72321
E275 a	Santiago	O80	LT, STp	CS12, CS20, CS21	A	4121	O80:H9	LT, STp	CS12, CFAI-var	1	1	1	1	1	178	5231721	50.67	112115
a102	Antofagasta	ND	STp	CS15, CS21	E	182	O169:H41	STp	CS6	0	0	0	0	0	141	5002381	50.35	175866
a104	Antofagasta	ND	STp-STh	CS6-CS21	B1	1988	O148:H28	LT, STp, STh	CS6, CS21	0	0	0	0	0	154	4900733	50.66	117588
a108	Antofagasta	ND	LT, STp, STh	negative	A	226(226)	ONT:H10	STh	CFAI-var	0	1	1	1	0	134	4708441	50.67	121677
a22	Antofagasta	ND	LT, STh	CS1-CS2-CS3-CS21	A	4(10)	O6:H16	LT, STh	CS1, CS3, CS21, pfc071	1	1	1	1	0	217	5019328	50.45	81609
a24	Antofagasta	ND	LT, STh	CS8-CS21	B1	1049	O114:H10	LT, STh	CS23	0	0	0	0	1	181	5235772	50.52	130594
a25	Antofagasta	ND	STh	CS21	A	1312	O25:H16	STh	CS21, CFAI-var	0	1	1	1	1	182	4877670	50.6	83906
a26	Antofagasta	ND	LT, STh	CS6-CS8	B1	940	O64:H5	LT	CFAI-var	0	0	0	0	0	355	5478860	50.02	106601
a3	Antofagasta	ND	STp	CS21	A	10(10)	ND	ND	NT	1	0	0	0	0	318	5197389	50.7	57166
a33	Antofagasta	ND	STh	CS21	A	1312	O25:H16	STh	CS21	0	1	1	1	0	210	5021836	50.6	84272
a37	Antofagasta	ND	LT, STh	CS6-CS21	A	1491	O25:H16	LT	CS6, CS21	1	0	0	0	0	216	4974455	50.58	65140
a39	Antofagasta	ND	LT	CS8-CS17	B1	423(23)	O8:H9	LT	CS17, CS19	1	0	1	1	0	195	5089190	50.61	113952
a4	Antofagasta	ND	LT, STh	CS2-CS3-CS21	A	4(10)	O6:H16	LT, STh	CS2, CS3, CS21	1	1	1	1	0	253	4969120	50.56	67721
a40	Antofagasta	ND	STh	CS21	A	1312	O25:H16	STh	CS21	0	1	1	1	1	185	4882900	50.62	86360
a47	Antofagasta	ND	STh	negative	A	5276	O25:H16	LT, STh	CS3, CS21	0	1	1	1	0	266	5412624	50.3	80605
a57	Antofagasta	ND	LT, STh	CFA/I-CS15-CS21	A	2332	O128ab/ac:H45	STh	CFAI, CS21	1	1	1	1	1	168	5157561	50.65	136596
a59	Antofagasta	ND	LT	CS8-CS21	B1	155(155)	O21:H12	LT	CS23	0	0	0	0	1	157	5202080	50.55	138000
a63	Antofagasta	ND	LT, STh	CS6-CS21	A	1312	O25:H16	STh	CS21	0	1	1	1	0	205	5026316	50.62	77228
a67	Antofagasta	ND	LT, STh	CS1-CS3-CS21	A	2353	O6:H16	LT, STh	CS1, CS3, CS21, pfc071	1	1	1	1	1	263	5034944	50.46	71921
a7	Antofagasta	ND	LT, STh	CS3-CS21	A	1312	O25:H16	STh	CS21	1	1	1	1	1	203	4995307	50.64	84190
a72	Antofagasta	ND	LT	CS1-CS2-CS3-CS21	A	4(10)	O6:H16	LT, STh	CS1, CS3, CS21, pfc071	1	1	1	0	1	219	5020491	50.46	81609
a73	Antofagasta	ND	LT, STh	CS2-CS3-CS21	A	4(10)	O6:H16	LT, STh	CS2, CS3, CS21	1	1	1	1	1	235	4936600	50.49	85610
a74	Antofagasta	ND	LT, STh	CS8-CS21	B1	155(155)	O15:H12	LT, STh	CS23	1	1	0	0	1	207	5238386	50.53	184917
a82	Antofagasta	ND	STh	CS21	A	4(10)	O6:H16	LT, STh	CS1, CS3, CS21, pfc071	1	1	1	1	1	214	5018579	50.46	81609
a85	Antofagasta	ND	STh	CS21	A	4(10)	O6:H16	LT, STh	CS2, CS3, CS21	1	1	1	1	1	219	4828880	50.57	82778
a9	Antofagasta	ND	LT, STh	CS21	E	3036	O97:H45	ND	NT	0	0	0	0	0	239	5042464	50.78	64145
a91	Antofagasta	ND	STh	CS21	A	1312	O25:H16	STh	CS21	0	1	1	1	0	204	5009571	50.63	81565
a92	Antofagasta	ND	LT	CS6-CS8-CS21	A	1312	O25:H16	LT	CS6, CS8	1	0	0	0	0	208	4957769	50.55	81565
a93	Antofagasta	ND	LT, STh	CFA/I-CS21	A	1312	O25:H16	STh	CS21	0	1	1	1	0	192	4997274	50.65	81565
c13	Calama	ND	LT, STh	negative	B1	58(155)	O25:H51	LT, STh	CS23	0	0	0	1	0	130	5090623	50.4	133471
c14	Calama	ND	STh	negative	B1	101(101)	O21:H21	STh	NT	1	1	1	0	1	83	4830195	50.46	196723
c3	Calama	ND	STh	CS2-CS3-CS21	A	4(10)	O6:H16	LT, STh	CS2, CS3, CS21	1	1	1	1	1	278	5021898	50.51	73580
c6	Calama	ND	STh	CS21	A	5276	O15:H11	STh	cfaD-var	1	1	1	1	1	157	4769758	50.77	98777
c7	Calama	ND	LT, STh	CS1-CS3-CS21	A	4(10)	O6:H16	LT, STh	CS1, CS3, CS21, pfc071	1	1	1	1	1	235	5087210	50.46	81609
c8	Calama	ND	LT, STh	CS1-CS3-CS21	A	4(10)	O6:H16	LT, STh	CS1, CS3, CS21, pfc071	1	1	1	1	1	239	5152628	50.17	79838
A103	Antofagasta	ND	STh	CFA/I-CS15-CS21	A	2332	O128ab/ac:H45	STh	CFAI, CS21	1	1	1	1	1	172	5086241	50.69	118635
A106	Antofagasta	ND	STh	CFA/I-CS15-CS21	A	2332	O128ab/ac:H45	STh	CFAI, CS21	1	1	1	0	0	175	5072311	50.7	118629

A44	Antofagasta	ND	STh	CFA/I-CS15-CS21	A	2332	O128ab/ac:H45	STh	CFAI, CS21	1	1	1	1	1	174	5084136	50.69	121396

*PCR for LT-I, ST1a and ST1b
**PCR for CFA/I, CS1, CS2, CS3, CS4, CS5, CS6, CS6, CS7, CS12, CS14, CS17, CS19, CS21
**additional CF genes by PCR for a- and c- strains (Antofagasta and Calama): CS8, CS13, CS15, CS18, CS20, CS22, CS23
ONT: nontypeable O serogroup
ND: not detected

Table S2: Reference genomes and corresponding pathotypes			
Isolate	Pathotype	Phylogroup	GenBank Accession
BL21	lab adapted	A	NC_012947.1
BW2952	lab adapted	A	NC_012759.1
SE11	fecal isolate	B1	NC_011415.1
IAI1	fecal isolate	B1	NC_011741.1
SMS_3_5	other	F	CP000970.1
HS	commensal	A	NC_009800.1
ATCC 8739	lab adapted	A	NC_010468.1
536	ExPEC	B2	NC_008253.1
S88	ExPEC	B2	NC_011742.1
UTI89	ExPEC	B2	NC_007946.1
CFT073	ExPEC	B2	AE014075.1
IA139	ExPEC	F	NC_011750.1
UMN026	ExPEC	D	NC_011751.1
042	EAEC	D	FN554766.1
53638	EIEC	A	AAKB00000000.2
55989	EAEC	B1	NC_011748.1
TY-2482	EAEC/STEC	B1	AFOG00000000.1
S. flexneri 2a 2457T	Shigella	B1	NC_004741.1
S. boydii 3083-94	Shigella	B1	NC_010658.1
S. sonnei 046	Shigella	B1	NC_007384.1
S. dysenteriae 197	Shigella	E	NC_007606.1
EDL933	EHEC	E	NC_002655.2
Sakai	EHEC	E	NC_002695.1
CB9615	EPEC	E	NC_013941.1
11368	EHEC	B1	NC_013361.1
11128	EHEC	B1	NC_013364.1
32/73	EPEC	B1	LAFA00000000
702324	EPEC	B2	JHRR00000000
B171	EPEC	B1	AAJX00000000.2
E110019	AEEC	B1	AAJW00000000.2
E2348/69	EPEC	B2	NC_011601.1
ETEC References			
TW11681	ETEC	A	AELD00000000
H10407	ETEC	A	FN649414.1
TW10598	ETEC	A	AELA00000000
UMNK88	ETEC	A	NC_017641.1
2846750	ETEC	A	AQGG00000000
MP021566.1	ETEC	A	AQEU00000000
BCE019_MS-13	ETEC	A	AQCZ00000000
B7A	ETEC	B1	AAJT00000000.2
TW14425	ETEC	B1	AELE00000000
E24377A	ETEC	B1	NC_009801.1
TW10828	ETEC	B1	AELC00000000
Jurua 18/11	ETEC	B1	AQFB00000000

ThroopD	ETEC	B1	AQEJ00000000
2726800	ETEC	B1	AQFE00000000
2854350	ETEC	B1	APZL00000000
BCE002_MS-12	ETEC	B1	AQDA00000000
Envira 8/11	ETEC	B1	AQFC00000000
2866350	ETEC	B1	APXJ00000000
BCE034_MS-14	ETEC	B1	AQCY00000000
2851500	ETEC	B1	AQDN00000000
2866450	ETEC	B1	AQDI00000000
2845650	ETEC	E	AQDR00000000
ETEC Lineage References			
E8_ETEC_L1	ETEC_lineage 1	A	ERS038927
E632_ETEC_L1	ETEC_lineage 1	A	ERS038942
E66_ETEC_L2	ETEC_lineage 2	A	ERS044460
E822_ETEC_L2	ETEC_lineage 2	A	ERS044484
E36_ETEC_L3	ETEC_lineage 3	B1	ERS044458
E810_ETEC_L3	ETEC_lineage 3	B1	ERS044481
E1115_ETEC_L4	ETEC_lineage 4	A	ERS206737
E1365_ETEC_L4	ETEC_lineage 4	A	ERS077686
E157_ETEC_L5	ETEC_lineage 5	B1	ERS077703
E21_ETEC_L5	ETEC_lineage 5	B1	ERS044456
E636_ETEC_L6	ETEC_lineage 6	A	ERS038943
E897_ETEC_L6	ETEC_lineage 6	A	ERS038948
E1484_ETEC_L7	ETEC_lineage 7	E	ERS206752
E370_ETEC_L7	ETEC_lineage 7	E	ERS038935
E2108_ETEC_L8	ETEC_lineage 8	B1	ERS077758
E224_ETEC_L8	ETEC_lineage 8	B1	ERS044476
E856_ETEC_L9	ETEC_lineage 9	A	ERS044486
E943_ETEC_L9	ETEC_lineage 9	A	ERS044499
E1285_ETEC_L10	ETEC_lineage 10	A	ERS077680
E945_ETEC_L10	ETEC_lineage 10	A	ERS077657
E167_ETEC_L11	ETEC_lineage 11	A	ERS077743
E1057_ETEC_L11	ETEC_lineage 11	A	ERS077667
E330_ETEC_L12	ETEC_lineage 12	A	ERS077581
E1556_ETEC_L12	ETEC_lineage 12	A	ERS077700
E628_ETEC_L13	ETEC_lineage 13	A	ERS077614
E1525_ETEC_L13	ETEC_lineage 13	A	ERS077692
E1091_ETEC_L15	ETEC_lineage 15	A	ERS038960
E2377_ETEC_L15	ETEC_lineage 15	A	ERS077765
E85_ETEC_L16	ETEC_lineage 16	A	ERS044463
E816_ETEC_L16	ETEC_lineage 16	A	ERS044483
E333_ETEC_L17	ETEC_lineage 17	B1	ERS038933
E2404_ETEC_L17	ETEC_lineage 17	B1	ERS077770
E659_ETEC_L18	ETEC_lineage 18	B1	ERS077617
E5085_ETEC_L18	ETEC_lineage 18	B1	ERS055670
E907_ETEC_L19	ETEC_lineage 19	B1	ERS044496

E920_ETEC_L19	ETEC_lineage 19	B1	ERS044497
E873_ETEC_L20	ETEC_lineage 20	B1	ERS077633
E2395_ETEC_L20	ETEC_lineage 20	B1	ERS077769
E1564_ETEC_L21	ETEC_lineage 21	A	ERS077702
E2367_ETEC_L21	ETEC_lineage 21	A	ERS077762

Table S3. O and H antigen prevalence in Chile ETEC isolates

O-antigen			H-antigen			O and H antigen		
O-antigen	number	percent	H-antigen	number	Percent	O and H serotype	number	Percent
O6	39	31.2	H16	39	31.2	O6:H16	25	20
O128ab/ac	20	16	H45	28	22.4	O128ab/ac:H 45	19	15.2
ONT	16	12.8	H-	14	11.2	O6:H-	14	11.2
O25	11	8.8	H9	7	5.6	O25:H16	10	8
O80	4	3.2	H21	7	5.6	ONT:H45	8	6.4
O148	3	2.4	H10	5	4	ONT:H21	5	4
O169	3	2.4	H12	5	4	O80:H9	4	3.2
O23	3	2.4	H28	3	2.4	O148:H28	3	2.4
O27	3	2.4	H4	2	1.6	O23:H16	3	2.4
O49	3	2.4	H20	2	1.6	O49:H12	3	2.4
O78	3	2.4	H25	2	1.6	O8:H9	3	2.4
O8	3	2.4	H32	2	1.6	ND	2	1.6
ND	2	1.6	ND	2	1.6	O159:H4	2	1.6
O15	2	1.6	H2	1	0.8	O169:H25	2	1.6
O159	2	1.6	H5	1	0.8	O27:H20	2	1.6
O21	2	1.6	H11	1	0.8	O78:H10	2	1.6
O114	1	0.8	H40	1	0.8	O16:H48	1	0.8
O115	1	0.8	H41	1	0.8	O114:H10	1	0.8
O16	1	0.8	H48	1	0.8	O115:H40	1	0.8
O4	1	0.8	H51	1	0.8	O128ab:H21	1	0.8
O64	1	0.8				O15:H11	1	0.8
O97	1	0.8				O15:H12	1	0.8
						O169:H41	1	0.8
						O21:H12	1	0.8
						O21:H21	1	0.8
						O25:H51	1	0.8
						O27:H10	1	0.8
						O4:H16	1	0.8
						O64:H5	1	0.8
						O78:H32	1	0.8
						O97:H45	1	0.8
						ONT:H10	1	0.8
						ONT:H2	1	0.8
						ONT:H32	1	0.8

Table S4. Distribution by Geography - Santiago

Gene_ID	Annotation	antiago_prese	Other_present	antiago_Abse	Other_Absent	Gene_ID	chisq-stats	pvalues	Gene_ID	pvalues
centroid_144	gram-negative pili assembly chaperone, N-terminal domain protein	88	23	0	14	centroid_144	33.788978	6.14E-09	centroid_144	4.99E-09
centroid_15982	glutamate decarboxylase	88	23	0	14	centroid_15982	33.788978	6.14E-09	centroid_15982	4.99E-09
centroid_17423	gram-negative pili assembly chaperone, N-terminal domain protein	88	23	0	14	centroid_17423	33.788978	6.14E-09	centroid_17423	4.99E-09
centroid_145	type VII secretion system (T7SS), usher family protein	88	24	0	13	centroid_145	30.840207	2.80E-08	centroid_145	2.33E-08
centroid_7651	type VII secretion system (T7SS), usher family protein	88	24	0	13	centroid_7651	30.840207	2.80E-08	centroid_7651	2.33E-08
centroid_9887	hypothetical protein	88	26	0	11	centroid_9887	25.101807	5.44E-07	centroid_9887	4.61E-07
centroid_12405	ABC transporter family protein	88	27	0	10	centroid_12405	22.310192	2.32E-06	centroid_12405	1.96E-06
centroid_1287	helix-turn-helix family protein	88	27	0	10	centroid_1287	22.310192	2.32E-06	centroid_1287	1.96E-06
centroid_1288	outer membrane protein G	88	27	0	10	centroid_1288	22.310192	2.32E-06	centroid_1288	1.96E-06
centroid_1289	ABC transporter family protein	88	27	0	10	centroid_1289	22.310192	2.32E-06	centroid_1289	1.96E-06
centroid_1290	beta-phosphoglucomutase	88	27	0	10	centroid_1290	22.310192	2.32E-06	centroid_1290	1.96E-06
centroid_13072	beta-phosphoglucomutase	88	27	0	10	centroid_13072	22.310192	2.32E-06	centroid_13072	1.96E-06
centroid_17428	haloacid dehalogenase-like hydrolase family protein	88	27	0	10	centroid_17428	22.310192	2.32E-06	centroid_17428	1.96E-06
centroid_1916	conserved hypothetical protein	88	27	0	10	centroid_1916	22.310192	2.32E-06	centroid_1916	1.96E-06
centroid_687	transcriptional activator CadC	88	27	0	10	centroid_687	22.310192	2.32E-06	centroid_687	1.96E-06
centroid_692	conserved hypothetical protein	88	27	0	10	centroid_692	22.310192	2.32E-06	centroid_692	1.96E-06
centroid_8199	ABC transporter family protein	88	27	0	10	centroid_8199	22.310192	2.32E-06	centroid_8199	1.96E-06
centroid_11295	fimbrial family protein	88	28	0	9	centroid_11295	19.569319	9.70E-06	centroid_11295	8.14E-06
centroid_12429	phosphate transporter family protein	88	28	0	9	centroid_12429	19.569319	9.70E-06	centroid_12429	8.14E-06
centroid_12430	phosphate transporter family protein	88	28	0	9	centroid_12430	19.569319	9.70E-06	centroid_12430	8.14E-06
centroid_1277	EAL domain protein	88	28	0	9	centroid_1277	19.569319	9.70E-06	centroid_1277	8.14E-06
centroid_146	fimbrial family protein	88	28	0	9	centroid_146	19.569319	9.70E-06	centroid_146	8.14E-06
centroid_17105	type VII secretion system (T7SS), usher family protein	88	28	0	9	centroid_17105	19.569319	9.70E-06	centroid_17105	8.14E-06
centroid_17641	conserved hypothetical protein	88	28	0	9	centroid_17641	19.569319	9.70E-06	centroid_17641	8.14E-06
centroid_2664	conserved hypothetical protein	88	28	0	9	centroid_2664	19.569319	9.70E-06	centroid_2664	8.14E-06
centroid_2717	phosphate transporter family protein	88	28	0	9	centroid_2717	19.569319	9.70E-06	centroid_2717	8.14E-06
centroid_2989	PTS system, cellobiose-specific IIC component	88	28	0	9	centroid_2989	19.569319	9.70E-06	centroid_2989	8.14E-06
centroid_2990	-diacetylchitobiose-specific phosphotransferase enzyme IIB compo	88	28	0	9	centroid_2990	19.569319	9.70E-06	centroid_2990	8.14E-06
centroid_3080	oxygen sensor protein DosP	88	28	0	9	centroid_3080	19.569319	9.70E-06	centroid_3080	8.14E-06
centroid_3180	inner membrane transport protein YeaN	88	28	0	9	centroid_3180	19.569319	9.70E-06	centroid_3180	8.14E-06
centroid_4400	glycine zipper 2TM domain protein	88	28	0	9	centroid_4400	19.569319	9.70E-06	centroid_4400	8.14E-06
centroid_4457	conserved hypothetical protein	88	28	0	9	centroid_4457	19.569319	9.70E-06	centroid_4457	8.14E-06
centroid_4668	haemolysin E family protein	88	28	0	9	centroid_4668	19.569319	9.70E-06	centroid_4668	8.14E-06
centroid_492	taurine ABC transporter, periplasmic binding protein	88	28	0	9	centroid_492	19.569319	9.70E-06	centroid_492	8.14E-06
centroid_5570	haemolysin E family protein	88	28	0	9	centroid_5570	19.569319	9.70E-06	centroid_5570	8.14E-06
centroid_602	glycine zipper 2TM domain protein	88	28	0	9	centroid_602	19.569319	9.70E-06	centroid_602	8.14E-06
centroid_688	transporter, basic amino acid/polyamine antiporter family protein	88	28	0	9	centroid_688	19.569319	9.70E-06	centroid_688	8.14E-06
centroid_689	lysine decarboxylase, inducible	88	28	0	9	centroid_689	19.569319	9.70E-06	centroid_689	8.14E-06
centroid_690	H+ symporter) family protein	88	28	0	9	centroid_690	19.569319	9.70E-06	centroid_690	8.14E-06
centroid_691	lysine--tRNA ligase	88	28	0	9	centroid_691	19.569319	9.70E-06	centroid_691	8.14E-06
centroid_693	conserved hypothetical protein	88	28	0	9	centroid_693	19.569319	9.70E-06	centroid_693	8.14E-06
centroid_7650	type VII secretion system (T7SS), usher family protein	88	28	0	9	centroid_7650	19.569319	9.70E-06	centroid_7650	8.14E-06
centroid_8096	conserved hypothetical protein	88	28	0	9	centroid_8096	19.569319	9.70E-06	centroid_8096	8.14E-06
centroid_8220	major Facilitator Superfamily protein	88	28	0	9	centroid_8220	19.569319	9.70E-06	centroid_8220	8.14E-06
centroid_8446	taurine ABC transporter, periplasmic binding protein	88	28	0	9	centroid_8446	19.569319	9.70E-06	centroid_8446	8.14E-06
centroid_8926	putative pTS system, cellobiose-specific, IIC component	88	28	0	9	centroid_8926	19.569319	9.70E-06	centroid_8926	8.14E-06
centroid_893	fimbrial assembly family protein	88	28	0	9	centroid_893	19.569319	9.70E-06	centroid_893	8.14E-06
centroid_9097	major Facilitator Superfamily protein	88	28	0	9	centroid_9097	19.569319	9.70E-06	centroid_9097	8.14E-06
centroid_9909	fimbrial family protein	88	28	0	9	centroid_9909	19.569319	9.70E-06	centroid_9909	8.14E-06
centroid_9910	conserved hypothetical protein	88	28	0	9	centroid_9910	19.569319	9.70E-06	centroid_9910	8.14E-06
centroid_3011	CTP pyrophosphohydrolase	88	29	0	8	centroid_3011	16.878859	3.98E-05	centroid_3011	3.28E-05
centroid_7219	conserved hypothetical protein	87	24	1	13	centroid_7219	26.952031	2.09E-07	centroid_7219	2.61E-07
centroid_2311	bacterial regulatory, gntR family protein	87	26	1	11	centroid_2311	21.355295	3.82E-06	centroid_2311	4.38E-06
centroid_2312	D binding domain of 6-phosphogluconate dehydrogenase family pro	87	26	1	11	centroid_2312	21.355295	3.82E-06	centroid_2312	4.38E-06
centroid_2313	conserved hypothetical protein	87	26	1	11	centroid_2313	21.355295	3.82E-06	centroid_2313	4.38E-06
centroid_17045	conserved hypothetical protein	87	27	1	10	centroid_17045	18.649788	1.57E-05	centroid_17045	1.70E-05
centroid_4351	conserved hypothetical protein	87	27	1	10	centroid_4351	18.649788	1.57E-05	centroid_4351	1.70E-05
centroid_13909	EAL domain protein	87	28	1	9	centroid_13909	16.009115	6.30E-05	centroid_13909	6.37E-05
centroid_13964	sensors of blue-light using FAD family protein	87	28	1	9	centroid_13964	16.009115	6.30E-05	centroid_13964	6.37E-05
centroid_14633	conserved hypothetical protein	87	28	1	9	centroid_14633	16.009115	6.30E-05	centroid_14633	6.37E-05
centroid_147	fimbrial family protein	87	28	1	9	centroid_147	16.009115	6.30E-05	centroid_147	6.37E-05
centroid_16436	major Facilitator Superfamily protein	87	28	1	9	centroid_16436	16.009115	6.30E-05	centroid_16436	6.37E-05
centroid_17685	conserved hypothetical protein	87	28	1	9	centroid_17685	16.009115	6.30E-05	centroid_17685	6.37E-05
centroid_1915	HTH-type transcriptional repressor YcgE	87	28	1	9	centroid_1915	16.009115	6.30E-05	centroid_1915	6.37E-05
centroid_2521	inner membrane transport protein YdiM	87	28	1	9	centroid_2521	16.009115	6.30E-05	centroid_2521	6.37E-05
centroid_2715	molybdopterin guanine dinucleotide synthesis B family protein	87	28	1	9	centroid_2715	16.009115	6.30E-05	centroid_2715	6.37E-05
centroid_2716	conserved hypothetical protein	87	28	1	9	centroid_2716	16.009115	6.30E-05	centroid_2716	6.37E-05
centroid_3182	putative diguanylate cyclase YeaP	87	28	1	9	centroid_3182	16.009115	6.30E-05	centroid_3182	6.37E-05
centroid_13336	shikimate transporter domain protein	86	25	2	12	centroid_13336	20.887096	4.87E-06	centroid_13336	6.05E-06
centroid_5486	putative aminopeptidase	86	25	2	12	centroid_5486	20.887096	4.87E-06	centroid_5486	6.05E-06
centroid_9791	papC C-terminal domain protein	86	27	2	10	centroid_9791	15.650488	7.62E-05	centroid_9791	8.01E-05
centroid_10606	helix-turn-helix domain protein	85	18	3	19	centroid_10606	38.04339	6.92E-10	centroid_10606	1.18E-09
centroid_10599	putative predicted inner membrane protein	85	24	3	13	centroid_10599	20.733385	5.28E-06	centroid_10599	6.66E-06
centroid_17355	conserved hypothetical protein	85	24	3	13	centroid_17355	20.733385	5.28E-06	centroid_17355	6.66E-06
centroid_3651	conserved hypothetical protein	85	24	3	13	centroid_3651	20.733385	5.28E-06	centroid_3651	6.66E-06
centroid_5438	conserved hypothetical protein	85	24	3	13	centroid_5438	20.733385	5.28E-06	centroid_5438	6.66E-06
centroid_6147	conserved hypothetical protein	85	26	3	11	centroid_6147	15.594175	7.85E-05	centroid_6147	8.26E-05
centroid_10357	type VI secretion system effector, Hcp1 family protein	84	24	4	13	centroid_10357	18.221393	1.97E-05	centroid_10357	2.26E-05
centroid_3649	conserved hypothetical protein	84	24	4	13	centroid_3649	18.221393	1.97E-05	centroid_3649	2.26E-05
centroid_3650	putative inner membrane protein yafU	84	24	4	13	centroid_3650	18.221393	1.97E-05	centroid_3650	2.26E-05
centroid_9099	-dependent phosphotransferase enzyme II for cellobiose domain pr	83	19	5	18	centroid_9099	29.230452	6.43E-08	centroid_9099	9.71E-08
centroid_12699	putative transposase	83	21	5	16	centroid_12699	23.673544	1.14E-06	centroid_12699	1.52E-06
centroid_3648	type VI secretion system effector, Hcp1 family protein	83	23	5	14	centroid_3648	18.475559	1.72E-05	centroid_3648	1.94E-05
centroid_16558	type VI secretion system effector, Hcp1 family protein	83	24	5	13	centroid_16558	16.020273	6.27E-05	centroid_16558	6.46E-05
centroid_4905	putative inner membrane protein	83	24	5	13	centroid_4905	16.020273	6.27E-05	centroid_4905	6.46E-05
centroid_12314	transposase, IS605 OrfB family	82	16	6	21	centroid_12314	35.467579	2.59E-09	centroid_12314	4.01E-09
centroid_9182	transglycosylase SLT domain protein	82	22	6	15	centroid_9182	18.848344	1.42E-05	centroid_9182	1.57E-05
centroid_12686	transposase, IS605 OrfB family	81	16	7	21	centroid_12686	32.937412	9.52E-09	centroid_12686	1.36E-08
centroid_4208	transposase, IS605 OrfB family	81	19	7	18	centroid_4208	24.476447	7.52E-07	centroid_4208	9.41E-07
centroid_8226	transposase, IS605 OrfB family	81	19	7	18	centroid_8226	24.476447	7.52E-07	centroid_8226	9.41E-07
centroid_10607	transposase, IS605 OrfB family	80	15	8	22	centroid_10607	33.521195	7.05E-09	centroid_10607	9.35E-09
centroid_3998	prophage CP4-57 regulatory family protein	80	16	8	21	centroid_3998	30.59408	3.18E-08	centroid_3998	4.15E-08
centroid_10758	conserved hypothetical protein	80	21	8	16	centroid_10758	17.444474	2.96E-05	centroid_10758	2.92E-05
centroid_12941	conserved hypothetical protein	80	21	8	16	centroid_12941	17.444474	2.96E-05	centroid_12941	2.92E-05
centroid_2389	conserved hypothetical protein	80	21	8	16	centroid_2389	17.444474	2.96E-05	centroid_2389	2.92E-05
centroid_5185	conserved hypothetical protein	80	21	8	16	centroid_5185	17.444474	2.96E-05	centroid_5185	2.92E-05
centroid_11498	conserved hypothetical protein	79	13	9	24	centroid_11498	37.257307	1.04E-09	centroid_11498	1.24E-09
centroid_2391	conserved hypothetical protein	79	21	9	16	centroid_2391	15.742571	7.26E-05	centroid_2391	0.0001117
centroid_8205	type VII secretion system (T7SS), usher family protein	79	21	9	16	centroid_8205	15.742571	7.26E-05	centroid_8205	0.0001117
centroid_13621	transglycosylase SLT domain protein	78	20	10	17	centroid_13621	16.410083	5.10E-05	centroid_13621	6.36E-05
centroid_13091	mRNA interferase MqsR	77	19	11	18	centroid_13091	17.12839	3.49E-05	centroid_13091	3.76E-05
centroid_2748	mRNA interferase MqsR	77	19	11	18	centroid_2748	17.12839	3.49E-05	centroid_2748	3.76E-05
centroid_8297	transglycosylase SLT domain protein	76	19	12	18	centroid_8297	15.639228	7.66E-05	centroid_8297	6.65E-05
centroid_8076	glutamate decarboxylase	75	12	13	25	centroid_8076	31.864393	1.65E-08	centroid_8076	1.41E-08

centroid_17627	fimbrial subunit ElfA	75	15	13	22	centroid_17627	23.632272	1.17E-06	centroid_17627	1.37E-06
centroid_11069	conserved hypothetical protein	75	17	13	20	centroid_11069	18.713201	1.52E-05	centroid_11069	1.36E-05
centroid_10219	phage tail sheath family protein	74	11	14	26	centroid_10219	32.920629	9.60E-09	centroid_10219	7.26E-09
centroid_18677	phage tail sheath family protein	74	12	14	25	centroid_18677	30.020952	4.27E-08	centroid_18677	3.42E-08
centroid_13199	phage late control gene D family protein	74	13	14	24	centroid_13199	27.236843	1.80E-07	centroid_13199	2.17E-07
centroid_3638	ogr/Delta-like zinc finger family protein	74	13	14	24	centroid_3638	27.236843	1.80E-07	centroid_3638	2.17E-07
centroid_10205	phage late control gene D family protein	74	14	14	23	centroid_10205	24.568282	7.17E-07	centroid_10205	7.14E-07
centroid_13559	putative phage tail tape measure domain protein	74	14	14	23	centroid_13559	24.568282	7.17E-07	centroid_13559	7.14E-07
centroid_3637	hok/gef family protein	74	15	14	22	centroid_3637	22.015682	2.70E-06	centroid_3637	2.43E-06
centroid_3640	phage tail sheath family protein	73	11	15	26	centroid_3640	31.106807	2.44E-08	centroid_3640	3.00E-08
centroid_16096	phage late control gene D family protein	73	13	15	24	centroid_16096	25.565506	4.28E-07	centroid_16096	3.70E-07
centroid_7906	phage late control gene D family protein	73	13	15	24	centroid_7906	25.565506	4.28E-07	centroid_7906	3.70E-07
centroid_10197	phage P2 GpU family protein	73	15	15	22	centroid_10197	20.497528	5.97E-06	centroid_10197	7.84E-06
centroid_11465	hypothetical protein	73	15	15	22	centroid_11465	20.497528	5.97E-06	centroid_11465	7.84E-06
centroid_16146	conserved hypothetical protein	73	15	15	22	centroid_16146	20.497528	5.97E-06	centroid_16146	7.84E-06
centroid_7095	phage P2 GpU family protein	73	15	15	22	centroid_7095	20.497528	5.97E-06	centroid_7095	7.84E-06
centroid_14165	conserved hypothetical protein	73	17	15	20	centroid_14165	15.908436	6.65E-05	centroid_14165	5.75E-05
centroid_5538	conserved hypothetical protein	73	17	15	20	centroid_5538	15.908436	6.65E-05	centroid_5538	5.75E-05
centroid_4244	conserved hypothetical protein	72	12	16	25	centroid_4244	26.625667	2.47E-07	centroid_4244	1.88E-07
centroid_10421	conserved predicted domain protein	72	13	16	24	centroid_10421	23.986331	9.70E-07	centroid_10421	7.31E-07
centroid_8326	putative conserved predicted protein	72	13	16	24	centroid_8326	23.986331	9.70E-07	centroid_8326	7.31E-07
centroid_3641	phage major tail tube protein	71	9	17	28	centroid_3641	33.503919	7.11E-09	centroid_3641	4.91E-09
centroid_13238	phage tail tube FII family protein	71	10	17	27	centroid_13238	30.565391	3.23E-08	centroid_13238	2.12E-08
centroid_3639	phage late control gene D family protein	71	11	17	26	centroid_3639	27.751161	1.38E-07	centroid_3639	9.22E-08
centroid_5227	conserved hypothetical protein	71	12	17	25	centroid_5227	25.06045	5.56E-07	centroid_5227	6.15E-07
centroid_3835	putative conserved predicted protein	71	13	17	24	centroid_3835	22.492874	2.11E-06	centroid_3835	1.87E-06
centroid_3643	phage P2 GpU family protein	71	15	17	22	centroid_3643	17.727699	2.55E-05	centroid_3643	1.89E-05
centroid_17429	conserved hypothetical protein	70	14	18	23	centroid_17429	18.708429	1.52E-05	centroid_17429	1.64E-05
centroid_3256	conserved hypothetical protein	70	15	18	22	centroid_3256	16.463455	4.96E-05	centroid_3256	4.31E-05
centroid_3257	conserved hypothetical protein	70	15	18	22	centroid_3257	16.463455	4.96E-05	centroid_3257	4.31E-05
centroid_3258	putative cPS-53 (KpLE1) prophage, predicted protein	70	15	18	22	centroid_3258	16.463455	4.96E-05	centroid_3258	4.31E-05
centroid_4195	plasmid stability family protein	70	15	18	22	centroid_4195	16.463455	4.96E-05	centroid_4195	4.31E-05
centroid_7397	putative yfdP	70	15	18	22	centroid_7397	16.463455	4.96E-05	centroid_7397	4.31E-05
centroid_7753	conserved hypothetical protein	70	15	18	22	centroid_7753	16.463455	4.96E-05	centroid_7753	4.31E-05
centroid_10247	hypothetical protein	69	15	19	22	centroid_10247	15.27233	9.31E-05	centroid_10247	6.56E-05
centroid_13239	phage tail tube FII family protein	69	15	19	22	centroid_13239	15.27233	9.31E-05	centroid_13239	6.56E-05
centroid_16143	hypothetical protein	69	15	19	22	centroid_16143	15.27233	9.31E-05	centroid_16143	6.56E-05
centroid_7843	phage tail tape measure protein, TP901 family, core region	68	13	20	24	centroid_7843	18.471362	1.72E-05	centroid_7843	1.19E-05
centroid_3834	terminase-like family protein	68	14	20	23	centroid_3834	16.245394	5.56E-05	centroid_3834	5.83E-05
centroid_7081	putative phage tail protein	68	14	20	23	centroid_7081	16.245394	5.56E-05	centroid_7081	5.83E-05
centroid_16147	baseplate J-like family protein	66	12	22	25	centroid_16147	18.343451	1.84E-05	centroid_16147	1.37E-05
centroid_13561	phage integrase family protein	66	13	22	24	centroid_13561	16.125983	5.93E-05	centroid_13561	3.95E-05
centroid_1940	conserved hypothetical protein	66	13	22	24	centroid_1940	16.125983	5.93E-05	centroid_1940	3.95E-05
centroid_11068	conserved hypothetical protein	65	11	23	26	centroid_11068	19.476254	1.02E-05	centroid_11068	6.48E-06
centroid_10953	baseplate J-like family protein	65	12	23	25	centroid_10953	17.191479	3.38E-05	centroid_10953	2.04E-05
centroid_10126	conserved hypothetical protein	64	10	24	27	centroid_10126	20.670842	5.45E-06	centroid_10126	2.98E-06
centroid_5033	conserved hypothetical protein	64	10	24	27	centroid_5033	20.670842	5.45E-06	centroid_5033	2.98E-06
centroid_10127	cold shock-like protein CspG	64	11	24	26	centroid_10127	18.313946	1.87E-05	centroid_10127	1.65E-05
centroid_10226	baseplate J-like family protein	64	11	24	26	centroid_10226	18.313946	1.87E-05	centroid_10226	1.65E-05
centroid_12178	putative ynfN	64	11	24	26	centroid_12178	18.313946	1.87E-05	centroid_12178	1.65E-05
centroid_5031	conserved hypothetical protein	64	11	24	26	centroid_5031	18.313946	1.87E-05	centroid_5031	1.65E-05
centroid_8212	conserved hypothetical protein	64	11	24	26	centroid_8212	18.313946	1.87E-05	centroid_8212	1.65E-05
centroid_5030	gnsA/GnsB family protein	62	11	26	26	centroid_5030	16.145441	5.87E-05	centroid_5030	4.90E-05
centroid_10248	bacteriophage replication gene A family protein	57	5	31	32	centroid_10248	25.366166	4.74E-07	centroid_10248	1.34E-07
centroid_5110	PIN domain protein	56	8	32	29	centroid_5110	16.759828	4.24E-05	centroid_5110	2.64E-05
centroid_4328	electron transport complex, RnfABCDGE type, C subunit	51	37	37	0	centroid_4328	20.12612	7.25E-06	centroid_4328	1.81E-07
centroid_10643	putative dNA primase competative inhibitor domain protein	47	4	41	33	centroid_10643	17.845456	2.40E-05	centroid_10643	6.84E-06
centroid_12376	hypothetical protein	47	5	41	32	centroid_12376	15.462783	8.41E-05	centroid_12376	2.51E-05
centroid_10359	conserved hypothetical protein	44	34	44	3	centroid_10359	17.738688	2.53E-05	centroid_10359	4.96E-06
centroid_14162	initiator Replication family protein	43	0	45	37	centroid_14162	25.437486	4.57E-07	centroid_14162	4.56E-09
centroid_4143	initiator Replication family protein	42	0	46	37	centroid_4143	24.498796	7.44E-07	centroid_4143	1.06E-08
centroid_4684	plasmid stability family protein	42	2	46	35	centroid_4684	18.641018	1.58E-05	centroid_4684	1.58E-06
centroid_5169	putative membrane protein	42	2	46	35	centroid_5169	18.641018	1.58E-05	centroid_5169	1.58E-06
centroid_10625	putative mobA domain protein	42	3	46	34	centroid_10625	16.068159	6.11E-05	centroid_10625	1.23E-05
centroid_3982	hypothetical protein	41	34	47	3	centroid_3982	20.425433	6.20E-06	centroid_3982	8.87E-07
centroid_3983	conserved hypothetical protein	41	34	47	3	centroid_3983	20.425433	6.20E-06	centroid_3983	8.87E-07
centroid_4242	conserved hypothetical protein	41	34	47	3	centroid_4242	20.425433	6.20E-06	centroid_4242	8.87E-07
centroid_11088	putative integrase core domain protein	41	33	47	4	centroid_11088	17.845456	2.40E-05	centroid_11088	6.84E-06
centroid_16011	conserved hypothetical protein	41	33	47	4	centroid_16011	17.845456	2.40E-05	centroid_16011	6.84E-06
centroid_10641	hypothetical protein	39	0	49	37	centroid_10641	21.814004	3.00E-06	centroid_10641	7.34E-08
centroid_3978	R.Ecl18kI	39	0	49	37	centroid_3978	21.814004	3.00E-06	centroid_3978	7.34E-08
centroid_3980	conserved hypothetical protein	39	0	49	37	centroid_3980	21.814004	3.00E-06	centroid_3980	7.34E-08
centroid_4038	helix-turn-helix domain protein	39	33	49	4	centroid_4038	19.676244	9.17E-06	centroid_4038	2.69E-06
centroid_4687	bacteriophage replication gene A family protein	38	1	50	36	centroid_4687	18.04247	2.16E-05	centroid_4687	1.50E-06
centroid_14139	conserved hypothetical protein	38	2	50	35	centroid_14139	15.390775	8.74E-05	centroid_14139	1.55E-05
centroid_8561	putative transposase	37	33	51	4	centroid_8561	21.620986	3.32E-06	centroid_8561	1.02E-06
centroid_3979	DNA (cytosine-5-)-methyltransferase family protein	37	0	51	37	centroid_3979	20.12612	7.25E-06	centroid_3979	1.81E-07
centroid_13566	conserved hypothetical protein	37	1	51	36	centroid_13566	17.241452	3.29E-05	centroid_13566	3.23E-06
centroid_5446	conserved hypothetical protein	36	34	52	3	centroid_5446	25.447588	4.55E-07	centroid_5446	4.59E-08
centroid_6892	conserved hypothetical protein	36	34	52	3	centroid_6892	25.447588	4.55E-07	centroid_6892	4.59E-08
centroid_4940	thiol peroxidase	36	0	52	37	centroid_4940	19.310722	1.11E-05	centroid_4940	2.28E-07
centroid_3981	rop family protein	35	0	53	37	centroid_3981	18.513517	1.69E-05	centroid_3981	4.55E-07
centroid_6825	hypothetical protein	32	0	56	37	centroid_6825	16.225244	5.62E-05	centroid_6825	2.34E-06
centroid_10415	hypothetical protein	31	28	57	9	centroid_10415	15.515687	8.18E-05	centroid_10415	6.30E-05
centroid_16109	putative parB-like partitioning protein	31	28	57	9	centroid_16109	15.515687	8.18E-05	centroid_16109	6.30E-05
centroid_13545	hypothetical protein	23	26	65	11	centroid_13545	19.476254	1.02E-05	centroid_13545	6.48E-06
centroid_12243	conserved hypothetical protein	18	25	70	12	centroid_12243	23.575659	1.20E-06	centroid_12243	9.11E-07
centroid_8121	conserved hypothetical protein	16	26	72	11	centroid_8121	29.385732	5.93E-08	centroid_8121	4.70E-08
centroid_7857	putative yceA protein	15	23	73	14	centroid_7857	22.972182	1.64E-06	centroid_7857	1.35E-06
centroid_16743	integrase core domain protein	15	20	73	17	centroid_16743	15.908436	6.65E-05	centroid_16743	5.75E-05
centroid_10644	phage portal protein, lambda family	14	22	74	15	centroid_10644	22.015682	2.70E-06	centroid_10644	2.43E-06
centroid_15283	conserved hypothetical protein	14	20	74	17	centroid_15283	17.262401	3.26E-05	centroid_15283	3.92E-05
centroid_9698	conserved hypothetical protein	13	22	75	15	centroid_9698	23.632272	1.17E-06	centroid_9698	1.37E-06
centroid_11141	haemolysin expression modulating family protein	13	20	75	17	centroid_11141	18.713201	1.52E-05	centroid_11141	1.36E-05
centroid_17734	caudovirales tail fibre assembly family protein	13	19	75	18	centroid_17734	16.428421	5.05E-05	centroid_17734	7.52E-05
centroid_12696	resolvase, N terminal domain protein	12	22	76	15	centroid_12696	25.355585	4.77E-07	centroid_12696	4.53E-07
centroid_17368	conserved hypothetical protein	12	20	76	17	centroid_17368	20.269422	6.73E-06	centroid_17368	7.62E-06
centroid_5345	phage Tail Collar domain protein	12	18	76	19	centroid_5345	15.639228	7.66E-05	centroid_5345	6.65E-05
centroid_13202	HNH endonuclease family protein	11	18	77	19	centroid_13202	17.12839	3.49E-05	centroid_13202	3.76E-05
centroid_16015	resolvase, N terminal domain protein	10	23	78	14	centroid_16015	32.02854	1.52E-08	centroid_16015	1.71E-08
centroid_5993	resolvase, N terminal domain protein	10	23	78	14	centroid_5993	32.02854	1.52E-08	centroid_5993	1.71E-08
centroid_15181	resolvase, N terminal domain protein	10	22	78	15	centroid_15181	29.160808	6.66E-08	centroid_15181	7.44E-08
centroid_15371	putative regulatory domain protein	10	19	78	18	centroid_15371	21.186023	4.17E-06	centroid_15371	4.18E-06
centroid_6754	prophage CP4-57 regulatory family protein	10	19	78	18	centroid_6754	21.186023	4.17E-06	centroid_6754	4.18E-06
centroid_11766	putative antigen 43 domain protein	10	18	78	19	centroid_11766	18.742337	1.50E-05	centroid_11766	2.56E-05
centroid_12617	phage-related baseplate assembly family protein	10	18	78	19	centroid_12617	18.742337	1.50E-05	centroid_12617	2.56E-05

centroid_15154	rhs element Vgr family protein	10	18	78	19	centroid_15154	18.742337	1.50E-05	centroid_15154	2.56E-05
centroid_9125	HNH endonuclease family protein	10	18	78	19	centroid_9125	18.742337	1.50E-05	centroid_9125	2.56E-05
centroid_9597	hok/gef family protein	8	22	80	15	centroid_9597	33.521195	7.05E-09	centroid_9597	9.35E-09
centroid_15103	hok/gef family protein	8	21	80	16	centroid_15103	30.59408	3.18E-08	centroid_15103	4.15E-08
centroid_5795	phage terminase large subunit family protein	8	20	80	17	centroid_5795	27.764001	1.37E-07	centroid_5795	1.73E-07
centroid_15117	conserved hypothetical protein	8	16	80	21	centroid_15117	17.444474	2.96E-05	centroid_15117	2.92E-05
centroid_14826	conserved hypothetical protein	7	18	81	19	centroid_14826	24.476447	7.52E-07	centroid_14826	9.41E-07
centroid_9752	conserved hypothetical protein	7	18	81	19	centroid_9752	24.476447	7.52E-07	centroid_9752	9.41E-07
centroid_12267	conserved hypothetical protein	7	16	81	21	centroid_12267	19.317774	1.11E-05	centroid_12267	1.20E-05
centroid_11622	putative dLP12 prophage; DNA base-flipping protein	7	15	81	22	centroid_11622	16.891252	3.96E-05	centroid_11622	3.97E-05
centroid_6120	replication regulatory RepB family protein	7	15	81	22	centroid_6120	16.891252	3.96E-05	centroid_6120	3.97E-05
centroid_10579	RHS repeat-associated core domain protein	6	17	82	20	centroid_10579	24.018421	9.54E-07	centroid_10579	1.24E-06
centroid_14606	integron integrase family protein	6	16	82	21	centroid_14606	21.385129	3.76E-06	centroid_14606	4.52E-06
centroid_15131	antirestriction family protein	6	15	82	22	centroid_15131	18.848344	1.42E-05	centroid_15131	1.57E-05
centroid_15999	istB-like ATP binding family protein	5	17	83	20	centroid_15999	26.408443	2.76E-07	centroid_15999	3.94E-07
centroid_8682	integrase core domain protein	5	16	83	21	centroid_8682	23.673544	1.14E-06	centroid_8682	1.52E-06
centroid_13428	putative transposase subunit 1	5	15	83	22	centroid_13428	21.028143	4.53E-06	centroid_13428	5.57E-06
centroid_15643	RHS repeat-associated core domain protein	5	15	83	22	centroid_15643	21.028143	4.53E-06	centroid_15643	5.57E-06
centroid_8522	bacterial dnaA family protein	5	15	83	22	centroid_8522	21.028143	4.53E-06	centroid_8522	5.57E-06
centroid_15941	putative gifsy-1 prophage VtiI	5	14	83	23	centroid_15941	18.475559	1.72E-05	centroid_15941	1.94E-05
centroid_6210	integrase	5	13	83	24	centroid_6210	16.020273	6.27E-05	centroid_6210	6.46E-05
centroid_18684	putative transposase subunit	4	15	84	22	centroid_18684	23.465011	1.27E-06	centroid_18684	1.73E-06
centroid_8521	putative transposase	4	15	84	22	centroid_8521	23.465011	1.27E-06	centroid_8521	1.73E-06
centroid_10383	regulatory protein rop	4	13	84	24	centroid_10383	18.221393	1.97E-05	centroid_10383	2.26E-05
centroid_12799	transposase family protein	4	13	84	24	centroid_12799	18.221393	1.97E-05	centroid_12799	2.26E-05
centroid_12811	putative tail length tape measure domain protein	4	13	84	24	centroid_12811	18.221393	1.97E-05	centroid_12811	2.26E-05
centroid_15841	endodeoxyribonuclease RusA family protein	4	13	84	24	centroid_15841	18.221393	1.97E-05	centroid_15841	2.26E-05
centroid_9697	antitermination family protein	4	13	84	24	centroid_9697	18.221393	1.97E-05	centroid_9697	2.26E-05
centroid_10378	hypothetical protein	4	12	84	25	centroid_10378	15.736435	7.28E-05	centroid_10378	7.60E-05
centroid_10379	conserved hypothetical protein	4	12	84	25	centroid_10379	15.736435	7.28E-05	centroid_10379	7.60E-05
centroid_10380	conserved hypothetical protein	4	12	84	25	centroid_10380	15.736435	7.28E-05	centroid_10380	7.60E-05
centroid_10602	hypothetical protein	4	12	84	25	centroid_10602	15.736435	7.28E-05	centroid_10602	7.60E-05
centroid_15173	transposase family protein	4	12	84	25	centroid_15173	15.736435	7.28E-05	centroid_15173	7.60E-05
centroid_8523	cytoskeleton-binding toxin CbtA	4	12	84	25	centroid_8523	15.736435	7.28E-05	centroid_8523	7.60E-05
centroid_10382	exc1 domain protein	3	13	85	24	centroid_10382	20.733385	5.28E-06	centroid_10382	6.66E-06
centroid_11463	conserved hypothetical protein	3	13	85	24	centroid_11463	20.733385	5.28E-06	centroid_11463	6.66E-06
centroid_15687	helix-turn-helix domain protein	3	13	85	24	centroid_15687	20.733385	5.28E-06	centroid_15687	6.66E-06
centroid_6060	putative transposase	3	13	85	24	centroid_6060	20.733385	5.28E-06	centroid_6060	6.66E-06
centroid_15820	ead/Ea22-like family protein	3	12	85	25	centroid_15820	18.120539	2.07E-05	centroid_15820	2.40E-05
centroid_10381	homeo-like domain protein	3	11	85	26	centroid_10381	15.594175	7.85E-05	centroid_10381	8.26E-05
centroid_10877	tail needle protein gp26	3	11	85	26	centroid_10877	15.594175	7.85E-05	centroid_10877	8.26E-05
centroid_16664	yafA domain protein	3	11	85	26	centroid_16664	15.594175	7.85E-05	centroid_16664	8.26E-05
centroid_13441	conserved hypothetical protein	2	34	86	3	centroid_13441	97.700647	4.87E-23	centroid_13441	9.72E-25
centroid_13442	conserved hypothetical protein	2	34	86	3	centroid_13442	97.700647	4.87E-23	centroid_13442	9.72E-25
centroid_13443	hindVP restriction endonuclease family protein	2	34	86	3	centroid_13443	97.700647	4.87E-23	centroid_13443	9.72E-25
centroid_13444	DNA (cytosine-5-)-methyltransferase family protein	2	34	86	3	centroid_13444	97.700647	4.87E-23	centroid_13444	9.72E-25
centroid_17138	hypothetical protein	2	34	86	3	centroid_17138	97.700647	4.87E-23	centroid_17138	9.72E-25
centroid_12233	putative entry exclusion protein 1	2	15	86	22	centroid_12233	29.287983	6.24E-08	centroid_12233	9.26E-08
centroid_15716	putative entry exclusion protein 1	2	15	86	22	centroid_15716	29.287983	6.24E-08	centroid_15716	9.26E-08
centroid_11770	conserved hypothetical protein	2	13	86	24	centroid_11770	23.617384	1.18E-06	centroid_11770	1.56E-06
centroid_12234	putative membrane protein	2	13	86	24	centroid_12234	23.617384	1.18E-06	centroid_12234	1.56E-06
centroid_12235	conserved hypothetical protein	2	13	86	24	centroid_12235	23.617384	1.18E-06	centroid_12235	1.56E-06
centroid_15176	conserved hypothetical protein	2	13	86	24	centroid_15176	23.617384	1.18E-06	centroid_15176	1.56E-06
centroid_15717	conserved hypothetical protein	2	13	86	24	centroid_15717	23.617384	1.18E-06	centroid_15717	1.56E-06
centroid_17514	hsdM N-terminal domain protein	2	13	86	24	centroid_17514	23.617384	1.18E-06	centroid_17514	1.56E-06
centroid_6005	CFA/I fimbrial subunit D	2	13	86	24	centroid_6005	23.617384	1.18E-06	centroid_6005	1.56E-06
centroid_12232	putative entry exclusion protein 2	2	12	86	25	centroid_12232	20.887096	4.87E-06	centroid_12232	6.05E-06
centroid_18634	type VII secretion system (T7SS), usher family protein	2	12	86	25	centroid_18634	20.887096	4.87E-06	centroid_18634	6.05E-06
centroid_5998	putative transposase domain protein	2	12	86	25	centroid_5998	20.887096	4.87E-06	centroid_5998	6.05E-06
centroid_6061	SEF14-like adhesin family protein	2	12	86	25	centroid_6061	20.887096	4.87E-06	centroid_6061	6.05E-06
centroid_6067	hypothetical protein	2	12	86	25	centroid_6067	20.887096	4.87E-06	centroid_6067	6.05E-06
centroid_6357	type I restriction-modification system, M subunit	2	12	86	25	centroid_6357	20.887096	4.87E-06	centroid_6357	6.05E-06
centroid_10337	T5orf172 domain protein	2	11	86	26	centroid_10337	18.230087	1.96E-05	centroid_10337	2.25E-05
centroid_13223	conserved hypothetical protein	2	11	86	26	centroid_13223	18.230087	1.96E-05	centroid_13223	2.25E-05
centroid_14810	transposase IS116/IS110/IS902 family protein	2	11	86	26	centroid_14810	18.230087	1.96E-05	centroid_14810	2.25E-05
centroid_15411	protein CexE	2	11	86	26	centroid_15411	18.230087	1.96E-05	centroid_15411	2.25E-05
centroid_15927	putative membrane protein	2	11	86	26	centroid_15927	18.230087	1.96E-05	centroid_15927	2.25E-05
centroid_16102	conserved hypothetical protein	2	11	86	26	centroid_16102	18.230087	1.96E-05	centroid_16102	2.25E-05
centroid_17015	gram-negative pili assembly chaperone, N-terminal domain protein	2	11	86	26	centroid_17015	18.230087	1.96E-05	centroid_17015	2.25E-05
centroid_5793	conserved hypothetical protein	2	11	86	26	centroid_5793	18.230087	1.96E-05	centroid_5793	2.25E-05
centroid_5999	transposase family protein	2	11	86	26	centroid_5999	18.230087	1.96E-05	centroid_5999	2.25E-05
centroid_6000	ABC transporter family protein	2	11	86	26	centroid_6000	18.230087	1.96E-05	centroid_6000	2.25E-05
centroid_6001	putative transporter protein AatB	2	11	86	26	centroid_6001	18.230087	1.96E-05	centroid_6001	2.25E-05
centroid_6002	outer membrane efflux family protein	2	11	86	26	centroid_6002	18.230087	1.96E-05	centroid_6002	2.25E-05
centroid_6003	permease family protein	2	11	86	26	centroid_6003	18.230087	1.96E-05	centroid_6003	2.25E-05
centroid_6059	serine protease SepA autotransporter	2	11	86	26	centroid_6059	18.230087	1.96E-05	centroid_6059	2.25E-05
centroid_6062	type VII secretion system (T7SS), usher family protein	2	11	86	26	centroid_6062	18.230087	1.96E-05	centroid_6062	2.25E-05
centroid_6066	putative membrane protein	2	11	86	26	centroid_6066	18.230087	1.96E-05	centroid_6066	2.25E-05
centroid_6068	hypothetical protein	2	11	86	26	centroid_6068	18.230087	1.96E-05	centroid_6068	2.25E-05
centroid_6894	integrase core domain protein	2	11	86	26	centroid_6894	18.230087	1.96E-05	centroid_6894	2.25E-05
centroid_15906	N-6 DNA Methylase family protein	2	10	86	27	centroid_15906	15.650488	7.62E-05	centroid_15906	8.01E-05
centroid_5615	resolvase, N terminal domain protein	2	10	86	27	centroid_5615	15.650488	7.62E-05	centroid_5615	8.01E-05
centroid_6014	conserved hypothetical protein	2	10	86	27	centroid_6014	15.650488	7.62E-05	centroid_6014	8.01E-05
centroid_6015	conserved hypothetical protein	2	10	86	27	centroid_6015	15.650488	7.62E-05	centroid_6015	8.01E-05
centroid_6016	conserved hypothetical protein	2	10	86	27	centroid_6016	15.650488	7.62E-05	centroid_6016	8.01E-05
centroid_6017	conserved hypothetical protein	2	10	86	27	centroid_6017	15.650488	7.62E-05	centroid_6017	8.01E-05
centroid_6018	putative translation elongation factor P domain protein	2	10	86	27	centroid_6018	15.650488	7.62E-05	centroid_6018	8.01E-05
centroid_6019	conserved hypothetical protein	2	10	86	27	centroid_6019	15.650488	7.62E-05	centroid_6019	8.01E-05
centroid_9613	csp231I C protein	2	10	86	27	centroid_9613	15.650488	7.62E-05	centroid_9613	8.01E-05
centroid_9614	putative dNA-directed RNA polymerase beta subunit	2	10	86	27	centroid_9614	15.650488	7.62E-05	centroid_9614	8.01E-05
centroid_13240	C-5 cytosine-specific DNA methylase family protein	1	25	87	12	centroid_13240	65.805538	4.98E-16	centroid_13240	3.28E-16
centroid_13241	putative r.SnaBI endonuclease	1	25	87	12	centroid_13241	65.805538	4.98E-16	centroid_13241	3.28E-16
centroid_13242	helix-turn-helix family protein	1	25	87	12	centroid_13242	65.805538	4.98E-16	centroid_13242	3.28E-16
centroid_13418	integrase core domain protein	1	16	87	21	centroid_13418	35.801432	2.18E-09	centroid_13418	2.88E-09
centroid_11972	putative gp55	1	14	87	23	centroid_11972	29.841324	4.69E-08	centroid_11972	6.03E-08
centroid_11974	conserved hypothetical protein	1	14	87	23	centroid_11974	29.841324	4.69E-08	centroid_11974	6.03E-08
centroid_11975	conserved hypothetical protein	1	14	87	23	centroid_11975	29.841324	4.69E-08	centroid_11975	6.03E-08
centroid_11976	conserved hypothetical protein	1	14	87	23	centroid_11976	29.841324	4.69E-08	centroid_11976	6.03E-08
centroid_11984	conserved hypothetical protein	1	14	87	23	centroid_11984	29.841324	4.69E-08	centroid_11984	6.03E-08
centroid_11985	hypothetical protein	1	14	87	23	centroid_11985	29.841324	4.69E-08	centroid_11985	6.03E-08
centroid_14867	conserved hypothetical protein	1	14	87	23	centroid_14867	29.841324	4.69E-08	centroid_14867	6.03E-08
centroid_11980	putative gp16	1	13	87	24	centroid_11980	26.952031	2.09E-07	centroid_11980	2.61E-07
centroid_11981	marR family protein	1	13	87	24	centroid_11981	26.952031	2.09E-07	centroid_11981	2.61E-07
centroid_12299	conserved hypothetical protein	1	13	87	24	centroid_12299	26.952031	2.09E-07	centroid_12299	2.61E-07
centroid_13167	conserved hypothetical protein	1	12	87	25	centroid_13167	24.12316	9.04E-07	centroid_13167	1.09E-06
centroid_17716	conserved hypothetical protein	1	12	87	25	centroid_17716	24.12316	9.04E-07	centroid_17716	1.09E-06

centroid_18633	putative transposase	1	12	87	25	centroid_18633	24.12316	9.04E-07	centroid_18633	1.09E-06
centroid_9090	conserved hypothetical protein	1	12	87	25	centroid_9090	24.12316	9.04E-07	centroid_9090	1.09E-06
centroid_11693	bacteriophage lysis family protein	1	11	87	26	centroid_11693	21.355295	3.82E-06	centroid_11693	4.38E-06
centroid_11732	HNH endonuclease family protein	1	11	87	26	centroid_11732	21.355295	3.82E-06	centroid_11732	4.38E-06
centroid_11739	conserved hypothetical protein	1	11	87	26	centroid_11739	21.355295	3.82E-06	centroid_11739	4.38E-06
centroid_11740	conserved hypothetical protein	1	11	87	26	centroid_11740	21.355295	3.82E-06	centroid_11740	4.38E-06
centroid_11741	immunoglobulin domain protein	1	11	87	26	centroid_11741	21.355295	3.82E-06	centroid_11741	4.38E-06
centroid_11742	phage tail assembly chaperone family protein	1	11	87	26	centroid_11742	21.355295	3.82E-06	centroid_11742	4.38E-06
centroid_11743	conserved hypothetical protein	1	11	87	26	centroid_11743	21.355295	3.82E-06	centroid_11743	4.38E-06
centroid_11744	phage tail tape measure protein, lambda family	1	11	87	26	centroid_11744	21.355295	3.82E-06	centroid_11744	4.38E-06
centroid_11745	phage minor tail family protein	1	11	87	26	centroid_11745	21.355295	3.82E-06	centroid_11745	4.38E-06
centroid_11872	cupin fold metallo, WbuC family protein	1	11	87	26	centroid_11872	21.355295	3.82E-06	centroid_11872	4.38E-06
centroid_11873	glycosyl transferases group 1 family protein	1	11	87	26	centroid_11873	21.355295	3.82E-06	centroid_11873	4.38E-06
centroid_11874	UDP-N-acetylglucosamine 2-epimerase	1	11	87	26	centroid_11874	21.355295	3.82E-06	centroid_11874	4.38E-06
centroid_11875	NAD dependent epimerase/dehydratase family protein	1	11	87	26	centroid_11875	21.355295	3.82E-06	centroid_11875	4.38E-06
centroid_11877	glycosyl transferases group 1 family protein	1	11	87	26	centroid_11877	21.355295	3.82E-06	centroid_11877	4.38E-06
centroid_12067	methyltransferase domain protein	1	11	87	26	centroid_12067	21.355295	3.82E-06	centroid_12067	4.38E-06
centroid_13168	conserved hypothetical protein	1	11	87	26	centroid_13168	21.355295	3.82E-06	centroid_13168	4.38E-06
centroid_15976	conserved hypothetical protein	1	11	87	26	centroid_15976	21.355295	3.82E-06	centroid_15976	4.38E-06
centroid_16037	conserved hypothetical protein	1	11	87	26	centroid_16037	21.355295	3.82E-06	centroid_16037	4.38E-06
centroid_6658	HNH endonuclease family protein	1	11	87	26	centroid_6658	21.355295	3.82E-06	centroid_6658	4.38E-06
centroid_6659	sensory box protein	1	11	87	26	centroid_6659	21.355295	3.82E-06	centroid_6659	4.38E-06
centroid_6699	bacterial regulatory, tetR family protein	1	11	87	26	centroid_6699	21.355295	3.82E-06	centroid_6699	4.38E-06
centroid_12069	ncrBC 5-methylcytosine restriction system component family protei	1	10	87	27	centroid_12069	18.649788	1.57E-05	centroid_12069	1.70E-05
centroid_12907	putative transposase	1	10	87	27	centroid_12907	18.649788	1.57E-05	centroid_12907	1.70E-05
centroid_15239	integrase core domain protein	1	10	87	27	centroid_15239	18.649788	1.57E-05	centroid_15239	1.70E-05
centroid_16486	AAA domain family protein	1	10	87	27	centroid_16486	18.649788	1.57E-05	centroid_16486	1.70E-05
centroid_17513	methyltransferase domain protein	1	10	87	27	centroid_17513	18.649788	1.57E-05	centroid_17513	1.70E-05
centroid_17573	nucleotidyltransferase domain protein	1	10	87	27	centroid_17573	18.649788	1.57E-05	centroid_17573	1.70E-05
centroid_6660	coA-transferase III family protein	1	10	87	27	centroid_6660	18.649788	1.57E-05	centroid_6660	1.70E-05
centroid_6661	HMGL-like family protein	1	10	87	27	centroid_6661	18.649788	1.57E-05	centroid_6661	1.70E-05
centroid_6662	sugar (and other) transporter family protein	1	10	87	27	centroid_6662	18.649788	1.57E-05	centroid_6662	1.70E-05
centroid_8929	integrase core domain protein	1	10	87	27	centroid_8929	18.649788	1.57E-05	centroid_8929	1.70E-05
centroid_9093	ead/Ea22-like family protein	1	10	87	27	centroid_9093	18.649788	1.57E-05	centroid_9093	1.70E-05
centroid_10121	conserved hypothetical protein	1	9	87	28	centroid_10121	16.009115	6.30E-05	centroid_10121	6.37E-05
centroid_11855	ninB family protein	1	9	87	28	centroid_11855	16.009115	6.30E-05	centroid_11855	6.37E-05
centroid_12068	ATPase associated with various cellular activities family protein	1	9	87	28	centroid_12068	16.009115	6.30E-05	centroid_12068	6.37E-05
centroid_12109	conserved hypothetical protein	1	9	87	28	centroid_12109	16.009115	6.30E-05	centroid_12109	6.37E-05
centroid_13971	conserved hypothetical protein	1	9	87	28	centroid_13971	16.009115	6.30E-05	centroid_13971	6.37E-05
centroid_13972	ERF superfamily protein	1	9	87	28	centroid_13972	16.009115	6.30E-05	centroid_13972	6.37E-05
centroid_13978	regulatory protein cro	1	9	87	28	centroid_13978	16.009115	6.30E-05	centroid_13978	6.37E-05
centroid_13980	dnaB-like helicase C terminal domain protein	1	9	87	28	centroid_13980	16.009115	6.30E-05	centroid_13980	6.37E-05
centroid_15260	phage tail fibre repeat family protein	1	9	87	28	centroid_15260	16.009115	6.30E-05	centroid_15260	6.37E-05
centroid_15548	hypothetical protein	1	9	87	28	centroid_15548	16.009115	6.30E-05	centroid_15548	6.37E-05
centroid_17625	conserved hypothetical protein	1	9	87	28	centroid_17625	16.009115	6.30E-05	centroid_17625	6.37E-05
centroid_14499	hypothetical protein	0	20	88	17	centroid_14499	52.677589	3.93E-13	centroid_14499	2.22E-13
centroid_14496	hypothetical protein	0	19	88	18	centroid_14496	49.379662	2.11E-12	centroid_14496	1.31E-12
centroid_15928	hypothetical protein	0	19	88	18	centroid_15928	49.379662	2.11E-12	centroid_15928	1.31E-12
centroid_17181	hypothetical protein	0	19	88	18	centroid_17181	49.379662	2.11E-12	centroid_17181	1.31E-12
centroid_17394	hypothetical protein	0	19	88	18	centroid_17394	49.379662	2.11E-12	centroid_17394	1.31E-12
centroid_14498	hypothetical protein	0	18	88	19	centroid_14498	46.143788	1.10E-11	centroid_14498	7.37E-12
centroid_14915	mbeD/MobD like family protein	0	18	88	19	centroid_14915	46.143788	1.10E-11	centroid_14915	7.37E-12
centroid_14916	mbeB-like, N-term conserved region family protein	0	18	88	19	centroid_14916	46.143788	1.10E-11	centroid_14916	7.37E-12
centroid_14495	replication family protein	0	17	88	20	centroid_14495	42.968316	5.56E-11	centroid_14495	3.98E-11
centroid_14640	conserved hypothetical protein	0	17	88	20	centroid_14640	42.968316	5.56E-11	centroid_14640	3.98E-11
centroid_14641	DNA relaxase MbeA	0	17	88	20	centroid_14641	42.968316	5.56E-11	centroid_14641	3.98E-11
centroid_14497	mobA/MobL family protein	0	16	88	21	centroid_14497	39.851671	2.74E-10	centroid_14497	2.07E-10
centroid_14827	integrase core domain protein	0	16	88	21	centroid_14827	39.851671	2.74E-10	centroid_14827	2.07E-10
centroid_14908	mobA/MobL family protein	0	16	88	21	centroid_14908	39.851671	2.74E-10	centroid_14908	2.07E-10
centroid_15762	bacterial regulatory , Fis family protein	0	15	88	22	centroid_15762	36.792361	1.31E-09	centroid_15762	1.03E-09
centroid_17401	pentapeptide repeats family protein	0	15	88	22	centroid_17401	36.792361	1.31E-09	centroid_17401	1.03E-09
centroid_14863	phage tail sheath family protein	0	14	88	23	centroid_14863	33.788978	6.14E-09	centroid_14863	4.99E-09
centroid_15760	conserved hypothetical protein	0	14	88	23	centroid_15760	33.788978	6.14E-09	centroid_15760	4.99E-09
centroid_15761	qnrB1	0	14	88	23	centroid_15761	33.788978	6.14E-09	centroid_15761	4.99E-09
centroid_16135	hypothetical protein	0	14	88	23	centroid_16135	33.788978	6.14E-09	centroid_16135	4.99E-09
centroid_14864	conserved hypothetical protein	0	13	88	24	centroid_14864	30.840207	2.80E-08	centroid_14864	2.33E-08
centroid_14865	putative lipoprotein	0	13	88	24	centroid_14865	30.840207	2.80E-08	centroid_14865	2.33E-08
centroid_14868	DNA adenine methylase family protein	0	13	88	24	centroid_14868	30.840207	2.80E-08	centroid_14868	2.33E-08
centroid_14642	mobilization protein MbeC	0	12	88	25	centroid_14642	27.944843	1.25E-07	centroid_14642	1.05E-07
centroid_14917	firmicute plasmid replication family protein	0	12	88	25	centroid_14917	27.944843	1.25E-07	centroid_14917	1.05E-07
centroid_14918	putative oRF3	0	12	88	25	centroid_14918	27.944843	1.25E-07	centroid_14918	1.05E-07
centroid_18675	putative variable tail fiber protein	0	12	88	25	centroid_18675	27.944843	1.25E-07	centroid_18675	1.05E-07
centroid_14919	putative taxA	0	11	88	26	centroid_14919	25.101807	5.44E-07	centroid_14919	4.61E-07
centroid_15183	type-2 restriction enzyme Cfr10I	0	11	88	26	centroid_15183	25.101807	5.44E-07	centroid_15183	4.61E-07
centroid_15226	integrase	0	11	88	26	centroid_15226	25.101807	5.44E-07	centroid_15226	4.61E-07
centroid_15228	DEAD/DEAH box helicase family protein	0	11	88	26	centroid_15228	25.101807	5.44E-07	centroid_15228	4.61E-07
centroid_15229	conserved hypothetical protein	0	11	88	26	centroid_15229	25.101807	5.44E-07	centroid_15229	4.61E-07
centroid_15272	dTDP-4-dehydrorhamnose 3,5-epimerase	0	11	88	26	centroid_15272	25.101807	5.44E-07	centroid_15272	4.61E-07
centroid_15273	glycosyl transferase 2 family protein	0	11	88	26	centroid_15273	25.101807	5.44E-07	centroid_15273	4.61E-07
centroid_15274	glycosyl transferases group 1 family protein	0	11	88	26	centroid_15274	25.101807	5.44E-07	centroid_15274	4.61E-07
centroid_15275	putative membrane protein	0	11	88	26	centroid_15275	25.101807	5.44E-07	centroid_15275	4.61E-07
centroid_15276	bacterial transferase hexapeptide family protein	0	11	88	26	centroid_15276	25.101807	5.44E-07	centroid_15276	4.61E-07
centroid_15277	UDP-N-acetylglucosamine 4,6-dehydratase/5-epimerase	0	11	88	26	centroid_15277	25.101807	5.44E-07	centroid_15277	4.61E-07
centroid_15363	phage gp6-like head-tail connector family protein	0	11	88	26	centroid_15363	25.101807	5.44E-07	centroid_15363	4.61E-07
centroid_15364	putative head-tail adaptor	0	11	88	26	centroid_15364	25.101807	5.44E-07	centroid_15364	4.61E-07
centroid_15372	phage portal protein, HK97 family	0	11	88	26	centroid_15372	25.101807	5.44E-07	centroid_15372	4.61E-07
centroid_15373	phage prohead protease, HK97 family	0	11	88	26	centroid_15373	25.101807	5.44E-07	centroid_15373	4.61E-07
centroid_15374	phage major capsid protein, HK97 family	0	11	88	26	centroid_15374	25.101807	5.44E-07	centroid_15374	4.61E-07
centroid_15547	bacteriophage lysis family protein	0	11	88	26	centroid_15547	25.101807	5.44E-07	centroid_15547	4.61E-07
centroid_15738	putative mobilization protein 1	0	11	88	26	centroid_15738	25.101807	5.44E-07	centroid_15738	4.61E-07
centroid_15874	DEAD/DEAH box helicase family protein	0	11	88	26	centroid_15874	25.101807	5.44E-07	centroid_15874	4.61E-07
centroid_16784	putative dead/deah box helicase domain protein	0	11	88	26	centroid_16784	25.101807	5.44E-07	centroid_16784	4.61E-07
centroid_16817	prophage CP4-57 regulatory family protein	0	11	88	26	centroid_16817	25.101807	5.44E-07	centroid_16817	4.61E-07
centroid_18276	polysaccharide biosynthesis family protein	0	11	88	26	centroid_18276	25.101807	5.44E-07	centroid_18276	4.61E-07
centroid_14339	conserved hypothetical protein	0	10	88	27	centroid_14339	22.310192	2.32E-06	centroid_14339	1.96E-06
centroid_14531	mobA/MobL family protein	0	10	88	27	centroid_14531	22.310192	2.32E-06	centroid_14531	1.96E-06
centroid_14954	conserved hypothetical protein	0	10	88	27	centroid_14954	22.310192	2.32E-06	centroid_14954	1.96E-06
centroid_15121	conserved hypothetical protein	0	10	88	27	centroid_15121	22.310192	2.32E-06	centroid_15121	1.96E-06
centroid_15122	conserved hypothetical protein	0	10	88	27	centroid_15122	22.310192	2.32E-06	centroid_15122	1.96E-06
centroid_15162	colicin pore forming domain protein	0	10	88	27	centroid_15162	22.310192	2.32E-06	centroid_15162	1.96E-06
centroid_15164	colicin E1 (microcin) immunity family protein	0	10	88	27	centroid_15164	22.310192	2.32E-06	centroid_15164	1.96E-06
centroid_15227	putative dead/deah box helicase domain protein	0	10	88	27	centroid_15227	22.310192	2.32E-06	centroid_15227	1.96E-06
centroid_15281	phage tail tape measure protein, lambda family	0	10	88	27	centroid_15281	22.310192	2.32E-06	centroid_15281	1.96E-06
centroid_15286	conserved hypothetical protein	0	10	88	27	centroid_15286	22.310192	2.32E-06	centroid_15286	1.96E-06
centroid_15313	type I restriction enzyme, S subunit domain protein	0	10	88	27	centroid_15313	22.310192	2.32E-06	centroid_15313	1.96E-06
centroid_15314	putative membrane protein	0	10	88	27	centroid_15314	22.310192	2.32E-06	centroid_15314	1.96E-06

centroid_15322	polysaccharide biosynthesis/export family protein	0	10	88	27	centroid_15322	22.310192	2.32E-06	centroid_15322	1.96E-06
centroid_15323	ABC-2 type transporter family protein	0	10	88	27	centroid_15323	22.310192	2.32E-06	centroid_15323	1.96E-06
centroid_15324	ABC transporter family protein	0	10	88	27	centroid_15324	22.310192	2.32E-06	centroid_15324	1.96E-06
centroid_15325	lysaccharide export inner-membrane , BexC/CtrB/KpsE family prot	0	10	88	27	centroid_15325	22.310192	2.32E-06	centroid_15325	1.96E-06
centroid_15326	methyltransferase domain protein	0	10	88	27	centroid_15326	22.310192	2.32E-06	centroid_15326	1.96E-06
centroid_15327	glycosyltransferase WbsX family protein	0	10	88	27	centroid_15327	22.310192	2.32E-06	centroid_15327	1.96E-06
centroid_15328	glycosyl transferase 2 family protein	0	10	88	27	centroid_15328	22.310192	2.32E-06	centroid_15328	1.96E-06
centroid_15329	nucleotide sugar dehydrogenase family protein	0	10	88	27	centroid_15329	22.310192	2.32E-06	centroid_15329	1.96E-06
centroid_15330	hypothetical protein	0	10	88	27	centroid_15330	22.310192	2.32E-06	centroid_15330	1.96E-06
centroid_15331	glycosyl transferase 2 family protein	0	10	88	27	centroid_15331	22.310192	2.32E-06	centroid_15331	1.96E-06
centroid_15332	glycosyl transferase 2 family protein	0	10	88	27	centroid_15332	22.310192	2.32E-06	centroid_15332	1.96E-06
centroid_15333	glycosyl transferases group 1 family protein	0	10	88	27	centroid_15333	22.310192	2.32E-06	centroid_15333	1.96E-06
centroid_15334	dTDP-glucose 4,6-dehydratase	0	10	88	27	centroid_15334	22.310192	2.32E-06	centroid_15334	1.96E-06
centroid_15335	dTDP-4-dehydrorhamnose reductase	0	10	88	27	centroid_15335	22.310192	2.32E-06	centroid_15335	1.96E-06
centroid_15336	glucose-1-phosphate thymidylyltransferase	0	10	88	27	centroid_15336	22.310192	2.32E-06	centroid_15336	1.96E-06
centroid_15337	dTDP-4-dehydrorhamnose 3,5-epimerase	0	10	88	27	centroid_15337	22.310192	2.32E-06	centroid_15337	1.96E-06
centroid_15338	capsule polysaccharide biosynthesis family protein	0	10	88	27	centroid_15338	22.310192	2.32E-06	centroid_15338	1.96E-06
centroid_15339	capsule polysaccharide biosynthesis family protein	0	10	88	27	centroid_15339	22.310192	2.32E-06	centroid_15339	1.96E-06
centroid_15340	putative pH01	0	10	88	27	centroid_15340	22.310192	2.32E-06	centroid_15340	1.96E-06
centroid_15347	DNA transfer protein gp7	0	10	88	27	centroid_15347	22.310192	2.32E-06	centroid_15347	1.96E-06
centroid_15353	type I restriction modification DNA specificity domain protein	0	10	88	27	centroid_15353	22.310192	2.32E-06	centroid_15353	1.96E-06
centroid_15354	yecA family protein	0	10	88	27	centroid_15354	22.310192	2.32E-06	centroid_15354	1.96E-06
centroid_15355	'Cold-shock' DNA-binding domain protein	0	10	88	27	centroid_15355	22.310192	2.32E-06	centroid_15355	1.96E-06
centroid_15381	putative transposase	0	10	88	27	centroid_15381	22.310192	2.32E-06	centroid_15381	1.96E-06
centroid_15392	putative outer membrane lipoprotein SlyB	0	10	88	27	centroid_15392	22.310192	2.32E-06	centroid_15392	1.96E-06
centroid_15789	adenine-specific methyltransferase EcoRI family protein	0	10	88	27	centroid_15789	22.310192	2.32E-06	centroid_15789	1.96E-06
centroid_15790	type-2 restriction enzyme EcoRI	0	10	88	27	centroid_15790	22.310192	2.32E-06	centroid_15790	1.96E-06
centroid_15882	tail spike protein	0	10	88	27	centroid_15882	22.310192	2.32E-06	centroid_15882	1.96E-06
centroid_17479	putative yacA	0	10	88	27	centroid_17479	22.310192	2.32E-06	centroid_17479	1.96E-06
centroid_17652	conserved hypothetical protein	0	10	88	27	centroid_17652	22.310192	2.32E-06	centroid_17652	1.96E-06
centroid_18682	conserved hypothetical protein	0	10	88	27	centroid_18682	22.310192	2.32E-06	centroid_18682	1.96E-06
centroid_14835	recT family protein	0	9	88	28	centroid_14835	19.569319	9.70E-06	centroid_14835	8.14E-06
centroid_14839	putative transmembrane anchored domain protein	0	9	88	28	centroid_14839	19.569319	9.70E-06	centroid_14839	8.14E-06
centroid_14840	conserved hypothetical protein	0	9	88	28	centroid_14840	19.569319	9.70E-06	centroid_14840	8.14E-06
centroid_14841	conserved hypothetical protein	0	9	88	28	centroid_14841	19.569319	9.70E-06	centroid_14841	8.14E-06
centroid_14842	putative predicted protein	0	9	88	28	centroid_14842	19.569319	9.70E-06	centroid_14842	8.14E-06
centroid_14844	conserved hypothetical protein	0	9	88	28	centroid_14844	19.569319	9.70E-06	centroid_14844	8.14E-06
centroid_14845	conserved hypothetical protein	0	9	88	28	centroid_14845	19.569319	9.70E-06	centroid_14845	8.14E-06
centroid_14847	putative membrane protein	0	9	88	28	centroid_14847	19.569319	9.70E-06	centroid_14847	8.14E-06
centroid_14848	conserved hypothetical protein	0	9	88	28	centroid_14848	19.569319	9.70E-06	centroid_14848	8.14E-06
centroid_15123	conserved hypothetical protein	0	9	88	28	centroid_15123	19.569319	9.70E-06	centroid_15123	8.14E-06
centroid_15218	prophage tail fibre N-terminal family protein	0	9	88	28	centroid_15218	19.569319	9.70E-06	centroid_15218	8.14E-06
centroid_15223	conserved hypothetical protein	0	9	88	28	centroid_15223	19.569319	9.70E-06	centroid_15223	8.14E-06
centroid_15224	putative transcriptional regulator	0	9	88	28	centroid_15224	19.569319	9.70E-06	centroid_15224	8.14E-06
centroid_15231	lysR family regulatory helix-turn-helix protein	0	9	88	28	centroid_15231	19.569319	9.70E-06	centroid_15231	8.14E-06
centroid_15262	hypothetical protein	0	9	88	28	centroid_15262	19.569319	9.70E-06	centroid_15262	8.14E-06
centroid_15285	conserved hypothetical protein	0	9	88	28	centroid_15285	19.569319	9.70E-06	centroid_15285	8.14E-06
centroid_15357	hypothetical protein	0	9	88	28	centroid_15357	19.569319	9.70E-06	centroid_15357	8.14E-06
centroid_15365	phage minor tail protein L	0	9	88	28	centroid_15365	19.569319	9.70E-06	centroid_15365	8.14E-06
centroid_15376	conserved hypothetical protein	0	9	88	28	centroid_15376	19.569319	9.70E-06	centroid_15376	8.14E-06
centroid_15377	P63C domain protein	0	9	88	28	centroid_15377	19.569319	9.70E-06	centroid_15377	8.14E-06
centroid_15379	DNA transfer gp20 domain protein	0	9	88	28	centroid_15379	19.569319	9.70E-06	centroid_15379	8.14E-06
centroid_15398	putative sel1	0	9	88	28	centroid_15398	19.569319	9.70E-06	centroid_15398	8.14E-06
centroid_15893	peptidase S24-like family protein	0	9	88	28	centroid_15893	19.569319	9.70E-06	centroid_15893	8.14E-06
centroid_15923	dihydrofolate reductase type 1	0	9	88	28	centroid_15923	19.569319	9.70E-06	centroid_15923	8.14E-06
centroid_17083	conserved hypothetical protein	0	9	88	28	centroid_17083	19.569319	9.70E-06	centroid_17083	8.14E-06
centroid_17084	conserved hypothetical protein	0	9	88	28	centroid_17084	19.569319	9.70E-06	centroid_17084	8.14E-06
centroid_17085	phage T7 tail fibre family protein	0	9	88	28	centroid_17085	19.569319	9.70E-06	centroid_17085	8.14E-06
centroid_17111	hypothetical protein	0	9	88	28	centroid_17111	19.569319	9.70E-06	centroid_17111	8.14E-06
centroid_18676	colicin pore forming domain protein	0	9	88	28	centroid_18676	19.569319	9.70E-06	centroid_18676	8.14E-06
centroid_14645	putative transposase	0	8	88	29	centroid_14645	16.878859	3.98E-05	centroid_14645	3.28E-05
centroid_14812	transposase IS66 family protein	0	8	88	29	centroid_14812	16.878859	3.98E-05	centroid_14812	3.28E-05
centroid_14858	conserved hypothetical protein	0	8	88	29	centroid_14858	16.878859	3.98E-05	centroid_14858	3.28E-05
centroid_14926	conserved hypothetical protein	0	8	88	29	centroid_14926	16.878859	3.98E-05	centroid_14926	3.28E-05
centroid_14986	conserved hypothetical protein	0	8	88	29	centroid_14986	16.878859	3.98E-05	centroid_14986	3.28E-05
centroid_15184	DNA (cytosine-5-)-methyltransferase family protein	0	8	88	29	centroid_15184	16.878859	3.98E-05	centroid_15184	3.28E-05
centroid_15225	phage integrase family protein	0	8	88	29	centroid_15225	16.878859	3.98E-05	centroid_15225	3.28E-05
centroid_15375	conserved hypothetical protein	0	8	88	29	centroid_15375	16.878859	3.98E-05	centroid_15375	3.28E-05
centroid_15397	putative entry exclusion protein 2	0	8	88	29	centroid_15397	16.878859	3.98E-05	centroid_15397	3.28E-05
centroid_15883	putative dNA transfer protein gp20	0	8	88	29	centroid_15883	16.878859	3.98E-05	centroid_15883	3.28E-05
centroid_15901	conserved hypothetical protein	0	8	88	29	centroid_15901	16.878859	3.98E-05	centroid_15901	3.28E-05
centroid_15929	phage tail fibre repeat family protein	0	8	88	29	centroid_15929	16.878859	3.98E-05	centroid_15929	3.28E-05
centroid_15961	conserved hypothetical protein	0	8	88	29	centroid_15961	16.878859	3.98E-05	centroid_15961	3.28E-05
centroid_15968	conserved hypothetical protein	0	8	88	29	centroid_15968	16.878859	3.98E-05	centroid_15968	3.28E-05

Table S4. Distribut+A939ion by Geography - Antogasta

Gene_ID	Annotation	ogasta_pres	Other_presen	togasta_Abs	Other_Absent	Gene_ID	chisq-stats	pvalues	Gene_ID	pvalues
centroid_4328	electron transport complex, RnfABCDGE type, C subunit	31	57	0	37	centroid_4328	15.495128	8.27E-05	centroid_4328	5.36E-06
centroid_13441	conserved hypothetical protein	28	8	3	86	centroid_13441	72.154818	1.99E-17	centroid_13441	1.65E-17
centroid_13442	conserved hypothetical protein	28	8	3	86	centroid_13442	72.154818	1.99E-17	centroid_13442	1.65E-17
centroid_13443	hindVP restriction endonuclease family protein	28	8	3	86	centroid_13443	72.154818	1.99E-17	centroid_13443	1.65E-17
centroid_13444	DNA (cytosine-5-)-methyltransferase family protein	28	8	3	86	centroid_13444	72.154818	1.99E-17	centroid_13444	1.65E-17
centroid_17138	hypothetical protein	28	8	3	86	centroid_17138	72.154818	1.99E-17	centroid_17138	1.65E-17
centroid_5446	conserved hypothetical protein	28	42	3	52	centroid_5446	17.900109	2.33E-05	centroid_5446	5.93E-06
centroid_6892	conserved hypothetical protein	28	42	3	52	centroid_6892	17.900109	2.33E-05	centroid_6892	5.93E-06
centroid_8561	putative transposase	28	42	3	52	centroid_8561	17.900109	2.33E-05	centroid_8561	5.93E-06
centroid_10415	hypothetical protein	25	34	6	60	centroid_10415	16.761112	4.24E-05	centroid_10415	2.08E-05
centroid_16109	putative parB-like partitioning protein	25	34	6	60	centroid_16109	16.761112	4.24E-05	centroid_16109	2.08E-05
centroid_10311	serine/threonine-phosphatase 2 domain protein	24	94	7	0	centroid_10311	18.416367	1.78E-05	centroid_10311	3.30E-05
centroid_1273	voltage gated chloride channel family protein	24	94	7	0	centroid_1273	18.416367	1.78E-05	centroid_1273	3.30E-05
centroid_5247	putative parB-like partitioning protein	24	31	7	63	centroid_5247	16.925192	3.89E-05	centroid_5247	2.01E-05
centroid_6902	conserved hypothetical protein	24	32	7	62	centroid_6902	16.026213	6.25E-05	centroid_6902	4.78E-05
centroid_3011	CTP pyrophosphohydrolase	23	94	8	0	centroid_3011	21.787789	3.05E-06	centroid_3011	6.71E-06
centroid_15442	conserved hypothetical protein	23	93	8	1	centroid_15442	17.816875	2.43E-05	centroid_15442	4.98E-05
centroid_17170	putative two-component-system connector protein AriR	23	93	8	1	centroid_17170	17.816875	2.43E-05	centroid_17170	4.98E-05
centroid_4815	biofilm development YmgB/AriR family protein	23	93	8	1	centroid_4815	17.816875	2.43E-05	centroid_4815	4.98E-05
centroid_11295	fimbrial family protein	22	94	9	0	centroid_11295	25.223071	5.11E-07	centroid_11295	1.32E-06
centroid_1277	EAL domain protein	22	94	9	0	centroid_1277	25.223071	5.11E-07	centroid_1277	1.32E-06
centroid_146	fimbrial family protein	22	94	9	0	centroid_146	25.223071	5.11E-07	centroid_146	1.32E-06
centroid_17105	type VII secretion system (T7SS), usher family protein	22	94	9	0	centroid_17105	25.223071	5.11E-07	centroid_17105	1.32E-06
centroid_17641	conserved hypothetical protein	22	94	9	0	centroid_17641	25.223071	5.11E-07	centroid_17641	1.32E-06
centroid_2664	conserved hypothetical protein	22	94	9	0	centroid_2664	25.223071	5.11E-07	centroid_2664	1.32E-06
centroid_2989	PTS system, cellobiose-specific IIC component	22	94	9	0	centroid_2989	25.223071	5.11E-07	centroid_2989	1.32E-06
centroid_2990	-diacetylchitobiose-specific phosphotransferase enzyme IIB compo	22	94	9	0	centroid_2990	25.223071	5.11E-07	centroid_2990	1.32E-06
centroid_3080	oxygen sensor protein DosP	22	94	9	0	centroid_3080	25.223071	5.11E-07	centroid_3080	1.32E-06

centroid_3180	inner membrane transport protein YeaN	22	94	9	0	centroid_3180	25.223071	5.11E-07	centroid_3180	1.32E-06
centroid_4400	glycine zipper 2TM domain protein	22	94	9	0	centroid_4400	25.223071	5.11E-07	centroid_4400	1.32E-06
centroid_4457	conserved hypothetical protein	22	94	9	0	centroid_4457	25.223071	5.11E-07	centroid_4457	1.32E-06
centroid_4668	haemolysin E family protein	22	94	9	0	centroid_4668	25.223071	5.11E-07	centroid_4668	1.32E-06
centroid_492	taurine ABC transporter, periplasmic binding protein	22	94	9	0	centroid_492	25.223071	5.11E-07	centroid_492	1.32E-06
centroid_5570	haemolysin E family protein	22	94	9	0	centroid_5570	25.223071	5.11E-07	centroid_5570	1.32E-06
centroid_602	glycine zipper 2TM domain protein	22	94	9	0	centroid_602	25.223071	5.11E-07	centroid_602	1.32E-06
centroid_688	transporter, basic amino acid/polyamine antiporter family protein	22	94	9	0	centroid_688	25.223071	5.11E-07	centroid_688	1.32E-06
centroid_689	lysine decarboxylase, inducible	22	94	9	0	centroid_689	25.223071	5.11E-07	centroid_689	1.32E-06
centroid_690	H+ symporter) family protein	22	94	9	0	centroid_690	25.223071	5.11E-07	centroid_690	1.32E-06
centroid_691	lysine--tRNA ligase	22	94	9	0	centroid_691	25.223071	5.11E-07	centroid_691	1.32E-06
centroid_693	conserved hypothetical protein	22	94	9	0	centroid_693	25.223071	5.11E-07	centroid_693	1.32E-06
centroid_7650	type VII secretion system (T7SS), usher family protein	22	94	9	0	centroid_7650	25.223071	5.11E-07	centroid_7650	1.32E-06
centroid_8096	conserved hypothetical protein	22	94	9	0	centroid_8096	25.223071	5.11E-07	centroid_8096	1.32E-06
centroid_8220	major Facilitator Superfamily protein	22	94	9	0	centroid_8220	25.223071	5.11E-07	centroid_8220	1.32E-06
centroid_8446	taurine ABC transporter, periplasmic binding protein	22	94	9	0	centroid_8446	25.223071	5.11E-07	centroid_8446	1.32E-06
centroid_8926	putative pTS system, cellobiose-specific, IIC component	22	94	9	0	centroid_8926	25.223071	5.11E-07	centroid_8926	1.32E-06
centroid_893	fimbrial assembly family protein	22	94	9	0	centroid_893	25.223071	5.11E-07	centroid_893	1.32E-06
centroid_9097	major Facilitator Superfamily protein	22	94	9	0	centroid_9097	25.223071	5.11E-07	centroid_9097	1.32E-06
centroid_9909	fimbrial family protein	22	94	9	0	centroid_9909	25.223071	5.11E-07	centroid_9909	1.32E-06
centroid_9910	conserved hypothetical protein	22	94	9	0	centroid_9910	25.223071	5.11E-07	centroid_9910	1.32E-06
centroid_8121	conserved hypothetical protein	22	20	9	74	centroid_8121	23.621428	1.17E-06	centroid_8121	1.18E-06
centroid_13909	EAL domain protein	22	93	9	1	centroid_13909	21.122029	4.31E-06	centroid_13909	1.09E-05
centroid_13964	sensors of blue-light using FAD family protein	22	93	9	1	centroid_13964	21.122029	4.31E-06	centroid_13964	1.09E-05
centroid_147	fimbrial family protein	22	93	9	1	centroid_147	21.122029	4.31E-06	centroid_147	1.09E-05
centroid_16436	major Facilitator Superfamily protein	22	93	9	1	centroid_16436	21.122029	4.31E-06	centroid_16436	1.09E-05
centroid_1915	HTH-type transcriptional repressor YcgE	22	93	9	1	centroid_1915	21.122029	4.31E-06	centroid_1915	1.09E-05
centroid_2521	inner membrane transport protein YdiM	22	93	9	1	centroid_2521	21.122029	4.31E-06	centroid_2521	1.09E-05
centroid_3182	putative diguanylate cyclase YeaP	22	93	9	1	centroid_3182	21.122029	4.31E-06	centroid_3182	1.09E-05
centroid_687	transcriptional activator CadC	22	93	9	1	centroid_687	21.122029	4.31E-06	centroid_687	1.09E-05
centroid_10816	fimH, mannose binding family protein	22	92	9	2	centroid_10816	17.807199	2.44E-05	centroid_10816	4.98E-05
centroid_17045	conserved hypothetical protein	22	92	9	2	centroid_17045	17.807199	2.44E-05	centroid_17045	4.98E-05
centroid_8893	blue light- and temperature-regulated antirepressor YcgF	22	92	9	2	centroid_8893	17.807199	2.44E-05	centroid_8893	4.98E-05
centroid_9571	fimbrial family protein	22	92	9	2	centroid_9571	17.807199	2.44E-05	centroid_9571	4.98E-05
centroid_13545	hypothetical protein	22	27	9	67	centroid_13545	15.727809	7.31E-05	centroid_13545	4.45E-05
centroid_12405	ABC transporter family protein	21	94	10	0	centroid_12405	28.722145	8.35E-08	centroid_12405	2.50E-07
centroid_1287	helix-turn-helix family protein	21	94	10	0	centroid_1287	28.722145	8.35E-08	centroid_1287	2.50E-07
centroid_1288	outer membrane protein G	21	94	10	0	centroid_1288	28.722145	8.35E-08	centroid_1288	2.50E-07
centroid_1289	ABC transporter family protein	21	94	10	0	centroid_1289	28.722145	8.35E-08	centroid_1289	2.50E-07
centroid_1290	beta-phosphoglucomutase	21	94	10	0	centroid_1290	28.722145	8.35E-08	centroid_1290	2.50E-07
centroid_13072	beta-phosphoglucomutase	21	94	10	0	centroid_13072	28.722145	8.35E-08	centroid_13072	2.50E-07
centroid_17428	haloacid dehalogenase-like hydrolase family protein	21	94	10	0	centroid_17428	28.722145	8.35E-08	centroid_17428	2.50E-07
centroid_1916	conserved hypothetical protein	21	94	10	0	centroid_1916	28.722145	8.35E-08	centroid_1916	2.50E-07
centroid_692	conserved hypothetical protein	21	94	10	0	centroid_692	28.722145	8.35E-08	centroid_692	2.50E-07
centroid_8199	ABC transporter family protein	21	94	10	0	centroid_8199	28.722145	8.35E-08	centroid_8199	2.50E-07
centroid_2311	bacterial regulatory, gntR family protein	21	92	10	2	centroid_2311	21.038206	4.50E-06	centroid_2311	1.15E-05
centroid_2312	D binding domain of 6-phosphogluconate dehydrogenase family prc	21	92	10	2	centroid_2312	21.038206	4.50E-06	centroid_2312	1.15E-05
centroid_2313	conserved hypothetical protein	21	92	10	2	centroid_2313	21.038206	4.50E-06	centroid_2313	1.15E-05
centroid_9791	papC C-terminal domain protein	21	92	10	2	centroid_9791	21.038206	4.50E-06	centroid_9791	1.15E-05
centroid_12243	conserved hypothetical protein	21	22	10	72	centroid_12243	18.390571	1.80E-05	centroid_12243	1.70E-05
centroid_10727	xylose isomerase-like TIM barrel family protein	21	91	10	3	centroid_10727	18.13197	2.06E-05	centroid_10727	4.13E-05
centroid_1291	glycosyl hydrolase family 65 central catalytic domain protein	21	91	10	3	centroid_1291	18.13197	2.06E-05	centroid_1291	4.13E-05
centroid_1292	conserved hypothetical protein	21	91	10	3	centroid_1292	18.13197	2.06E-05	centroid_1292	4.13E-05
centroid_1293	xylose isomerase-like TIM barrel family protein	21	91	10	3	centroid_1293	18.13197	2.06E-05	centroid_1293	4.13E-05
centroid_14310	conserved hypothetical protein	21	91	10	3	centroid_14310	18.13197	2.06E-05	centroid_14310	4.13E-05
centroid_14311	oxidoreductase , NAD-binding Rossmann fold family protein	21	91	10	3	centroid_14311	18.13197	2.06E-05	centroid_14311	4.13E-05
centroid_9336	conserved hypothetical protein	21	91	10	3	centroid_9336	18.13197	2.06E-05	centroid_9336	4.13E-05
centroid_13240	C-5 cytosine-specific DNA methylase family protein	20	6	11	88	centroid_13240	44.359442	2.73E-11	centroid_13240	1.43E-10
centroid_13241	putative r.SnaBI endonuclease	20	6	11	88	centroid_13241	44.359442	2.73E-11	centroid_13241	1.43E-10
centroid_13242	helix-turn-helix family protein	20	6	11	88	centroid_13242	44.359442	2.73E-11	centroid_13242	1.43E-10
centroid_9597	hok/gef family protein	20	10	11	84	centroid_9597	34.205051	4.96E-09	centroid_9597	1.19E-08
centroid_15181	resolvase, N terminal domain protein	20	12	11	82	centroid_15181	30.117834	4.07E-08	centroid_15181	7.06E-08
centroid_16015	resolvase, N terminal domain protein	20	13	11	81	centroid_16015	28.269921	1.06E-07	centroid_16015	1.59E-07
centroid_5993	resolvase, N terminal domain protein	20	13	11	81	centroid_5993	28.269921	1.06E-07	centroid_5993	1.59E-07
centroid_12696	resolvase, N terminal domain protein	20	14	11	80	centroid_12696	26.537407	2.58E-07	centroid_12696	3.43E-07
centroid_13336	shikimate transporter domain protein	20	91	11	3	centroid_13336	21.303599	3.92E-06	centroid_13336	9.78E-06
centroid_5486	putative aminopeptidase	20	91	11	3	centroid_5486	21.303599	3.92E-06	centroid_5486	9.78E-06
centroid_6147	conserved hypothetical protein	20	91	11	3	centroid_6147	21.303599	3.92E-06	centroid_6147	9.78E-06
centroid_17050	putative parB-like nuclease	20	20	11	74	centroid_17050	18.092251	2.10E-05	centroid_17050	1.69E-05
centroid_1247	conserved hypothetical protein	20	89	11	5	centroid_1247	16.397829	5.13E-05	centroid_1247	8.19E-05
centroid_145	type VII secretion system (T7SS), usher family protein	19	93	12	1	centroid_145	31.529718	1.96E-08	centroid_145	8.80E-08
centroid_7651	type VII secretion system (T7SS), usher family protein	19	93	12	1	centroid_7651	31.529718	1.96E-08	centroid_7651	8.80E-08
centroid_15103	hok/gef family protein	19	10	12	84	centroid_15103	30.785262	2.88E-08	centroid_15103	6.39E-08
centroid_15982	glutamate decarboxylase	19	92	12	2	centroid_15982	27.797402	1.35E-07	centroid_15982	5.20E-07
centroid_7219	conserved hypothetical protein	19	92	12	2	centroid_7219	27.797402	1.35E-07	centroid_7219	5.20E-07
centroid_10599	putative predicted inner membrane protein	19	90	12	4	centroid_10599	21.802919	3.02E-06	centroid_10599	7.37E-06
centroid_17355	conserved hypothetical protein	19	90	12	4	centroid_17355	21.802919	3.02E-06	centroid_17355	7.37E-06
centroid_3651	conserved hypothetical protein	19	90	12	4	centroid_3651	21.802919	3.02E-06	centroid_3651	7.37E-06
centroid_5438	conserved hypothetical protein	19	90	12	4	centroid_5438	21.802919	3.02E-06	centroid_5438	7.37E-06
centroid_9698	conserved hypothetical protein	19	16	12	78	centroid_9698	20.518845	5.90E-06	centroid_9698	5.99E-06
centroid_10357	type VI secretion system effector, Hcp1 family protein	19	89	12	5	centroid_10357	19.369022	1.08E-05	centroid_10357	2.11E-05
centroid_3649	conserved hypothetical protein	19	89	12	5	centroid_3649	19.369022	1.08E-05	centroid_3649	2.11E-05
centroid_3650	putative inner membrane protein yafU	19	89	12	5	centroid_3650	19.369022	1.08E-05	centroid_3650	2.11E-05
centroid_10644	phage portal protein, lambda family	19	17	12	77	centroid_10644	19.166966	1.20E-05	centroid_10644	1.52E-05
centroid_16558	type VI secretion system effector, Hcp1 family protein	19	88	12	6	centroid_16558	17.22804	3.32E-05	centroid_16558	5.30E-05
centroid_3721	bacterial regulatory, gntR family protein	19	88	12	6	centroid_3721	17.22804	3.32E-05	centroid_3721	5.30E-05
centroid_4905	putative inner membrane protein	19	88	12	6	centroid_4905	17.22804	3.32E-05	centroid_4905	5.30E-05
centroid_17492	conjugative transfer relaxase protein TraI	19	19	12	75	centroid_17492	16.70039	4.38E-05	centroid_17492	3.66E-05
centroid_7857	putative yceA protein	19	19	12	75	centroid_7857	16.70039	4.38E-05	centroid_7857	3.66E-05
centroid_16045	putative parB-like nuclease	19	20	12	74	centroid_16045	15.574069	7.93E-05	centroid_16045	8.41E-05
centroid_144	gram-negative pili assembly chaperone, N-terminal domain protein	18	93	13	1	centroid_144	35.153824	3.05E-09	centroid_144	1.60E-08
centroid_17423	gram-negative pili assembly chaperone, N-terminal domain protein	18	93	13	1	centroid_17423	35.153824	3.05E-09	centroid_17423	1.60E-08
centroid_3648	type VI secretion system effector, Hcp1 family protein	18	88	13	6	centroid_3648	20.185193	7.03E-06	centroid_3648	1.36E-05
centroid_11141	haemolysin expression modulating family protein	18	15	13	79	centroid_11141	19.160096	1.20E-05	centroid_11141	1.26E-05
centroid_5795	phage terminase large subunit family protein	17	11	14	83	centroid_5795	22.535283	2.06E-06	centroid_5795	3.17E-06
centroid_12699	putative transposase	17	87	14	7	centroid_12699	21.101166	4.36E-06	centroid_12699	8.32E-06
centroid_15371	putative regulatory domain protein	17	12	14	82	centroid_15371	20.858545	4.94E-06	centroid_15371	6.62E-06
centroid_6754	prophage CP4-57 regulatory family protein	17	12	14	82	centroid_6754	20.858545	4.94E-06	centroid_6754	6.62E-06
centroid_17368	conserved hypothetical protein	17	15	14	79	centroid_17368	16.51813	4.82E-05	centroid_17368	6.76E-05
centroid_6095	transposase family protein	17	16	14	78	centroid_6095	15.267491	9.33E-05	centroid_6095	9.65E-05
centroid_17394	hypothetical protein	16	3	15	91	centroid_17394	38.731374	4.86E-10	centroid_17394	3.07E-09
centroid_14499	hypothetical protein	16	4	15	90	centroid_14499	35.457035	2.61E-09	centroid_14499	1.33E-08
centroid_14826	conserved hypothetical protein	16	9	15	85	centroid_14826	23.188165	1.47E-06	centroid_14826	2.70E-06
centroid_9752	conserved hypothetical protein	16	9	15	85	centroid_9752	23.188165	1.47E-06	centroid_9752	2.70E-06
centroid_13672	poly-beta-1,6 N-acetyl-D-glucosamine export porin PgaA	16	85	15	9	centroid_13672	20.203979	6.96E-06	centroid_13672	1.11E-05
centroid_2895	poly-beta-1,6 N-acetyl-D-glucosamine export porin PgaA	16	84	15	10	centroid_2895	18.469565	1.73E-05	centroid_2895	2.36E-05

centroid_4208	transposase, IS605 OrfB family	16	84	15	10	centroid_4208	18.469565	1.73E-05	centroid_4208	2.36E-05
centroid_8226	transposase, IS605 OrfB family	16	84	15	10	centroid_8226	18.469565	1.73E-05	centroid_8226	2.36E-05
centroid_12617	phage-related baseplate assembly family protein	16	12	15	82	centroid_12617	18.065595	2.13E-05	centroid_12617	2.55E-05
centroid_15154	rhs element Vgr family protein	16	12	15	82	centroid_15154	18.065595	2.13E-05	centroid_15154	2.55E-05
centroid_9125	HNH endonuclease family protein	16	12	15	82	centroid_9125	18.065595	2.13E-05	centroid_9125	2.55E-05
centroid_13202	HNH endonuclease family protein	16	13	15	81	centroid_13202	16.617444	4.57E-05	centroid_13202	4.88E-05
centroid_14498	hypothetical protein	15	3	16	91	centroid_14498	35.051412	3.21E-09	centroid_14498	1.74E-08
centroid_14496	hypothetical protein	15	4	16	90	centroid_14496	31.883717	1.64E-08	centroid_14496	7.10E-08
centroid_15928	hypothetical protein	15	4	16	90	centroid_15928	31.883717	1.64E-08	centroid_15928	7.10E-08
centroid_17181	hypothetical protein	15	4	16	90	centroid_17181	31.883717	1.64E-08	centroid_17181	7.10E-08
centroid_9099	dependent phosphotransferase enzyme II for cellobiose domain pr	15	87	16	7	centroid_9099	27.416375	1.64E-07	centroid_9099	4.36E-07
centroid_14606	integron integrase family protein	15	7	16	87	centroid_14606	24.193667	8.71E-07	centroid_14606	1.97E-06
centroid_10579	RHS repeat-associated core domain protein	15	8	16	86	centroid_10579	22.104613	2.58E-06	centroid_10579	4.85E-06
centroid_15117	conserved hypothetical protein	15	9	16	85	centroid_15117	20.203979	6.96E-06	centroid_15117	1.11E-05
centroid_6792	phage tail tape measure protein, lambda family	15	9	16	85	centroid_6792	20.203979	6.96E-06	centroid_6792	1.11E-05
centroid_12632	mu-like prophage major head subunit gpT family protein	15	11	16	83	centroid_12632	16.882606	3.98E-05	centroid_12632	4.75E-05
centroid_5997	tn3 transposase DDE domain protein	15	11	16	83	centroid_5997	16.882606	3.98E-05	centroid_5997	4.75E-05
centroid_13091	mRNA interferase MqsR	15	81	16	13	centroid_13091	16.617444	4.57E-05	centroid_13091	4.88E-05
centroid_2748	mRNA interferase MqsR	15	81	16	13	centroid_2748	16.617444	4.57E-05	centroid_2748	4.88E-05
centroid_13516	putative tail length tape measure domain protein	15	12	16	82	centroid_13516	15.427131	8.57E-05	centroid_13516	9.10E-05
centroid_6535	conserved hypothetical protein	15	12	16	82	centroid_6535	15.427131	8.57E-05	centroid_6535	9.10E-05
centroid_10606	helix-turn-helix domain protein	14	89	17	5	centroid_10606	36.077246	1.90E-09	centroid_10606	8.99E-09
centroid_14495	replication family protein	14	3	17	91	centroid_14495	31.465749	2.03E-08	centroid_14495	9.23E-08
centroid_14640	conserved hypothetical protein	14	3	17	91	centroid_14640	31.465749	2.03E-08	centroid_14640	9.23E-08
centroid_14915	mbeD/MobD like family protein	14	4	17	90	centroid_14915	28.41428	9.79E-08	centroid_14915	3.55E-07
centroid_14916	mbeB-like, N-term conserved region family protein	14	4	17	90	centroid_14916	28.41428	9.79E-08	centroid_14916	3.55E-07
centroid_15941	putative gifsy-1 prophage VtiI	14	5	17	89	centroid_15941	25.701657	3.98E-07	centroid_15941	1.15E-06
centroid_15643	RHS repeat-associated core domain protein	14	6	17	88	centroid_15643	23.277532	1.40E-06	centroid_15643	3.26E-06
centroid_15131	antirestriction family protein	14	7	17	87	centroid_15131	21.101166	4.36E-06	centroid_15131	8.32E-06
centroid_11622	putative dLP12 prophage; DNA base-flipping protein	14	8	17	86	centroid_11622	19.139241	1.22E-05	centroid_11622	1.94E-05
centroid_6120	replication regulatory RepB family protein	14	8	17	86	centroid_6120	19.139241	1.22E-05	centroid_6120	1.94E-05
centroid_5798	conserved hypothetical protein	14	10	17	84	centroid_5798	15.753304	7.22E-05	centroid_5798	8.55E-05
centroid_5799	ATP-binding sugar transporter from pro-phage family protein	14	10	17	84	centroid_5799	15.753304	7.22E-05	centroid_5799	8.55E-05
centroid_11069	conserved hypothetical protein	14	78	17	16	centroid_11069	15.267491	9.33E-05	centroid_11069	9.65E-05
centroid_17514	hsdM N-terminal domain protein	13	2	18	92	centroid_17514	31.314507	2.19E-08	centroid_17514	1.02E-07
centroid_14827	integrase core domain protein	13	3	18	91	centroid_14827	27.97665	1.23E-07	centroid_14827	4.63E-07
centroid_3998	prophage CP4-57 regulatory family protein	13	83	18	11	centroid_3998	25.581151	4.24E-07	centroid_3998	7.27E-07
centroid_12799	transposase family protein	13	4	18	90	centroid_12799	25.052323	5.58E-07	centroid_12799	1.67E-06
centroid_12811	putative tail length tape measure domain protein	13	4	18	90	centroid_12811	25.052323	5.58E-07	centroid_12811	1.67E-06
centroid_13418	integrase core domain protein	13	4	18	90	centroid_13418	25.052323	5.58E-07	centroid_13418	1.67E-06
centroid_14641	DNA relaxase MbeA	13	4	18	90	centroid_14641	25.052323	5.58E-07	centroid_14641	1.67E-06
centroid_15841	endodeoxyribonuclease RusA family protein	13	4	18	90	centroid_15841	25.052323	5.58E-07	centroid_15841	1.67E-06
centroid_9697	antitermination family protein	13	4	18	90	centroid_9697	25.052323	5.58E-07	centroid_9697	1.67E-06
centroid_6210	integrase	13	5	18	89	centroid_6210	22.473156	2.13E-06	centroid_6210	5.08E-06
centroid_5797	clp protease family protein	13	7	18	87	centroid_5797	18.145289	2.05E-05	centroid_5797	3.28E-05
centroid_5800	prophage minor tail Z family protein	13	7	18	87	centroid_5800	18.145289	2.05E-05	centroid_5800	3.28E-05
centroid_12674	phage integrase family protein	13	77	18	17	centroid_12674	16.552634	4.73E-05	centroid_12674	4.76E-05
centroid_5965	CFA/III pilin	13	8	18	86	centroid_5965	16.318535	5.35E-05	centroid_5965	7.25E-05
centroid_6747	lysis S family protein	13	8	18	86	centroid_6747	16.318535	5.35E-05	centroid_6747	7.25E-05
centroid_8682	integrase core domain protein	13	8	18	86	centroid_8682	16.318535	5.35E-05	centroid_8682	7.25E-05
centroid_12314	transposase, IS605 OrfB family	12	86	19	8	centroid_12314	35.294664	2.83E-09	centroid_12314	9.06E-09
centroid_12686	transposase, IS605 OrfB family	12	85	19	9	centroid_12686	32.95534	9.43E-09	centroid_12686	2.49E-08
centroid_13167	conserved hypothetical protein	12	1	19	93	centroid_13167	31.529718	1.96E-08	centroid_13167	8.80E-08
centroid_17716	conserved hypothetical protein	12	1	19	93	centroid_17716	31.529718	1.96E-08	centroid_17716	8.80E-08
centroid_9090	conserved hypothetical protein	12	1	19	93	centroid_9090	31.529718	1.96E-08	centroid_9090	8.80E-08
centroid_10607	transposase, IS605 OrfB family	12	83	19	11	centroid_10607	28.767749	8.16E-08	centroid_10607	1.54E-07
centroid_12299	conserved hypothetical protein	12	2	19	92	centroid_12299	27.797402	1.35E-07	centroid_12299	5.20E-07
centroid_14863	phage tail sheath family protein	12	2	19	92	centroid_14863	27.797402	1.35E-07	centroid_14863	5.20E-07
centroid_6357	type I restriction-modification system, M subunit	12	2	19	92	centroid_6357	27.797402	1.35E-07	centroid_6357	5.20E-07
centroid_11770	conserved hypothetical protein	12	3	19	91	centroid_11770	24.587577	7.10E-07	centroid_11770	2.19E-06
centroid_11972	putative gp55	12	3	19	91	centroid_11972	24.587577	7.10E-07	centroid_11972	2.19E-06
centroid_11974	conserved hypothetical protein	12	3	19	91	centroid_11974	24.587577	7.10E-07	centroid_11974	2.19E-06
centroid_11975	conserved hypothetical protein	12	3	19	91	centroid_11975	24.587577	7.10E-07	centroid_11975	2.19E-06
centroid_11976	conserved hypothetical protein	12	3	19	91	centroid_11976	24.587577	7.10E-07	centroid_11976	2.19E-06
centroid_11984	conserved hypothetical protein	12	3	19	91	centroid_11984	24.587577	7.10E-07	centroid_11984	2.19E-06
centroid_11985	hypothetical protein	12	3	19	91	centroid_11985	24.587577	7.10E-07	centroid_11985	2.19E-06
centroid_14867	conserved hypothetical protein	12	3	19	91	centroid_14867	24.587577	7.10E-07	centroid_14867	2.19E-06
centroid_10382	exc1 domain protein	12	4	19	90	centroid_10382	21.802919	3.02E-06	centroid_10382	7.37E-06
centroid_14497	mobA/MobL family protein	12	4	19	90	centroid_14497	21.802919	3.02E-06	centroid_14497	7.37E-06
centroid_14908	mobA/MobL family protein	12	4	19	90	centroid_14908	21.802919	3.02E-06	centroid_14908	7.37E-06
centroid_15687	helix-turn-helix domain protein	12	4	19	90	centroid_15687	21.802919	3.02E-06	centroid_15687	7.37E-06
centroid_8523	cytoskeleton-binding toxin CbtA	12	4	19	90	centroid_8523	21.802919	3.02E-06	centroid_8523	7.37E-06
centroid_17627	fimbrial subunit ElfA	12	78	19	16	centroid_17627	20.518845	5.90E-06	centroid_17627	5.99E-06
centroid_10383	regulatory protein rop	12	5	19	89	centroid_10383	19.369022	1.08E-05	centroid_10383	2.11E-05
centroid_3637	hok/gef family protein	12	77	19	17	centroid_3637	19.166966	1.20E-05	centroid_3637	1.52E-05
centroid_10197	phage P2 GpU family protein	12	76	19	18	centroid_10197	17.896192	2.33E-05	centroid_10197	2.26E-05
centroid_16146	conserved hypothetical protein	12	76	19	18	centroid_16146	17.896192	2.33E-05	centroid_16146	2.26E-05
centroid_7095	phage P2 GpU family protein	12	76	19	18	centroid_7095	17.896192	2.33E-05	centroid_7095	2.26E-05
centroid_7450	conserved hypothetical protein	12	6	19	88	centroid_7450	17.22804	3.32E-05	centroid_7450	5.30E-05
centroid_3643	phage P2 GpU family protein	12	74	19	20	centroid_3643	15.574069	7.93E-05	centroid_3643	8.41E-05
centroid_13385	phage tail tape measure protein, lambda family	12	7	19	87	centroid_13385	15.334326	9.01E-05	centroid_13385	0.0001206
centroid_13425	putative phage domain protein	12	7	19	87	centroid_13425	15.334326	9.01E-05	centroid_13425	0.0001206
centroid_15710	conserved hypothetical protein	12	7	19	87	centroid_15710	15.334326	9.01E-05	centroid_15710	0.0001206
centroid_7452	phage stabilisation family protein	12	7	19	87	centroid_7452	15.334326	9.01E-05	centroid_7452	0.0001206
centroid_9750	putative yheA protein	12	7	19	87	centroid_9750	15.334326	9.01E-05	centroid_9750	0.0001206
centroid_9751	putative phage protein	12	7	19	87	centroid_9751	15.334326	9.01E-05	centroid_9751	0.0001206
centroid_15183	type-2 restriction enzyme Cfr10I	11	0	20	94	centroid_15183	32.285576	1.33E-08	centroid_15183	4.57E-08
centroid_15226	integrase	11	0	20	94	centroid_15226	32.285576	1.33E-08	centroid_15226	4.57E-08
centroid_15228	DEAD/DEAH box helicase family protein	11	0	20	94	centroid_15228	32.285576	1.33E-08	centroid_15228	4.57E-08
centroid_15229	conserved hypothetical protein	11	0	20	94	centroid_15229	32.285576	1.33E-08	centroid_15229	4.57E-08
centroid_15363	phage gp6-like head-tail connector family protein	11	0	20	94	centroid_15363	32.285576	1.33E-08	centroid_15363	4.57E-08
centroid_15364	putative head-tail adaptor	11	0	20	94	centroid_15364	32.285576	1.33E-08	centroid_15364	4.57E-08
centroid_15372	phage portal protein, HK97 family	11	0	20	94	centroid_15372	32.285576	1.33E-08	centroid_15372	4.57E-08
centroid_15373	phage prohead protease, HK97 family	11	0	20	94	centroid_15373	32.285576	1.33E-08	centroid_15373	4.57E-08
centroid_15374	phage major capsid protein, HK97 family	11	0	20	94	centroid_15374	32.285576	1.33E-08	centroid_15374	4.57E-08
centroid_15547	bacteriophage lysis family protein	11	0	20	94	centroid_15547	32.285576	1.33E-08	centroid_15547	4.57E-08
centroid_15874	DEAD/DEAH box helicase family protein	11	0	20	94	centroid_15874	32.285576	1.33E-08	centroid_15874	4.57E-08
centroid_16784	putative dead/deah box helicase domain protein	11	0	20	94	centroid_16784	32.285576	1.33E-08	centroid_16784	4.57E-08
centroid_11498	conserved hypothetical protein	11	81	20	13	centroid_11498	28.269921	1.06E-07	centroid_11498	1.59E-07
centroid_11732	HNH endonuclease family protein	11	1	20	93	centroid_11732	27.981976	1.22E-07	centroid_11732	4.60E-07
centroid_11739	conserved hypothetical protein	11	1	20	93	centroid_11739	27.981976	1.22E-07	centroid_11739	4.60E-07
centroid_11740	conserved hypothetical protein	11	1	20	93	centroid_11740	27.981976	1.22E-07	centroid_11740	4.60E-07
centroid_11741	immunoglobulin domain protein	11	1	20	93	centroid_11741	27.981976	1.22E-07	centroid_11741	4.60E-07
centroid_11742	phage tail assembly chaperone family protein	11	1	20	93	centroid_11742	27.981976	1.22E-07	centroid_11742	4.60E-07
centroid_11743	conserved hypothetical protein	11	1	20	93	centroid_11743	27.981976	1.22E-07	centroid_11743	4.60E-07
centroid_11744	phage tail tape measure protein, lambda family	11	1	20	93	centroid_11744	27.981976	1.22E-07	centroid_11744	4.60E-07

centroid_11745	phage minor tail family protein	11	1	20	93	centroid_11745	27.981976	1.22E-07	centroid_11745	4.60E-07
centroid_12067	methyltransferase domain protein	11	1	20	93	centroid_12067	27.981976	1.22E-07	centroid_12067	4.60E-07
centroid_13168	conserved hypothetical protein	11	1	20	93	centroid_13168	27.981976	1.22E-07	centroid_13168	4.60E-07
centroid_14642	mobilization protein MbeC	11	1	20	93	centroid_14642	27.981976	1.22E-07	centroid_14642	4.60E-07
centroid_15976	conserved hypothetical protein	11	1	20	93	centroid_15976	27.981976	1.22E-07	centroid_15976	4.60E-07
centroid_16037	conserved hypothetical protein	11	1	20	93	centroid_16037	27.981976	1.22E-07	centroid_16037	4.60E-07
centroid_6658	HNH endonuclease family protein	11	1	20	93	centroid_6658	27.981976	1.22E-07	centroid_6658	4.60E-07
centroid_6659	sensory box protein	11	1	20	93	centroid_6659	27.981976	1.22E-07	centroid_6659	4.60E-07
centroid_6699	bacterial regulatory, tetR family protein	11	1	20	93	centroid_6699	27.981976	1.22E-07	centroid_6699	4.60E-07
centroid_14864	conserved hypothetical protein	11	2	20	92	centroid_14864	24.370503	7.95E-07	centroid_14864	2.51E-06
centroid_14865	putative lipoprotein	11	2	20	92	centroid_14865	24.370503	7.95E-07	centroid_14865	2.51E-06
centroid_14868	DNA adenine methylase family protein	11	2	20	92	centroid_14868	24.370503	7.95E-07	centroid_14868	2.51E-06
centroid_5793	conserved hypothetical protein	11	2	20	92	centroid_5793	24.370503	7.95E-07	centroid_5793	2.51E-06
centroid_10205	phage late control gene D family protein	11	77	20	17	centroid_10205	21.940781	2.81E-06	centroid_10205	2.72E-06
centroid_11465	hypothetical protein	11	77	20	17	centroid_11465	21.940781	2.81E-06	centroid_11465	2.72E-06
centroid_13559	putative phage tail tape measure domain protein	11	77	20	17	centroid_13559	21.940781	2.81E-06	centroid_13559	2.72E-06
centroid_10877	tail needle protein gp26	11	3	20	91	centroid_10877	21.303599	3.92E-06	centroid_10877	9.78E-06
centroid_11980	putative gp16	11	3	20	91	centroid_11980	21.303599	3.92E-06	centroid_11980	9.78E-06
centroid_11981	marR family protein	11	3	20	91	centroid_11981	21.303599	3.92E-06	centroid_11981	9.78E-06
centroid_5998	putative transposase domain protein	11	3	20	91	centroid_5998	21.303599	3.92E-06	centroid_5998	9.78E-06
centroid_3638	ogr/Delta-like zinc finger family protein	11	76	20	18	centroid_3638	20.583251	5.71E-06	centroid_3638	6.78E-06
centroid_14456	conserved hypothetical protein	11	4	20	90	centroid_14456	18.673079	1.55E-05	centroid_14456	3.07E-05
centroid_15341	conserved hypothetical protein	11	4	20	90	centroid_15341	18.673079	1.55E-05	centroid_15341	3.07E-05
centroid_15762	bacterial regulatory , Fis family protein	11	4	20	90	centroid_15762	18.673079	1.55E-05	centroid_15762	3.07E-05
centroid_15820	ead/Ea22-like family protein	11	4	20	90	centroid_15820	18.673079	1.55E-05	centroid_15820	3.07E-05
centroid_17401	pentapeptide repeats family protein	11	4	20	90	centroid_17401	18.673079	1.55E-05	centroid_17401	3.07E-05
centroid_6005	CFA/I fimbrial subunit D	11	4	20	90	centroid_6005	18.673079	1.55E-05	centroid_6005	3.07E-05
centroid_17429	conserved hypothetical protein	11	73	20	21	centroid_17429	16.948326	3.84E-05	centroid_17429	3.87E-05
centroid_10378	hypothetical protein	11	5	20	89	centroid_10378	16.397829	5.13E-05	centroid_10378	8.19E-05
centroid_10379	conserved hypothetical protein	11	5	20	89	centroid_10379	16.397829	5.13E-05	centroid_10379	8.19E-05
centroid_10380	conserved hypothetical protein	11	5	20	89	centroid_10380	16.397829	5.13E-05	centroid_10380	8.19E-05
centroid_10602	hypothetical protein	11	5	20	89	centroid_10602	16.397829	5.13E-05	centroid_10602	8.19E-05
centroid_10886	conserved hypothetical protein	11	5	20	89	centroid_10886	16.397829	5.13E-05	centroid_10886	8.19E-05
centroid_11463	conserved hypothetical protein	11	5	20	89	centroid_11463	16.397829	5.13E-05	centroid_11463	8.19E-05
centroid_15173	transposase family protein	11	5	20	89	centroid_15173	16.397829	5.13E-05	centroid_15173	8.19E-05
centroid_6060	putative transposase	11	5	20	89	centroid_6060	16.397829	5.13E-05	centroid_6060	8.19E-05
centroid_14954	conserved hypothetical protein	10	0	21	94	centroid_14954	28.722145	8.35E-08	centroid_14954	2.50E-07
centroid_15227	putative dead/deah box helicase domain protein	10	0	21	94	centroid_15227	28.722145	8.35E-08	centroid_15227	2.50E-07
centroid_15281	phage tail tape measure protein, lambda family	10	0	21	94	centroid_15281	28.722145	8.35E-08	centroid_15281	2.50E-07
centroid_15286	conserved hypothetical protein	10	0	21	94	centroid_15286	28.722145	8.35E-08	centroid_15286	2.50E-07
centroid_15313	type I restriction enzyme, S subunit domain protein	10	0	21	94	centroid_15313	28.722145	8.35E-08	centroid_15313	2.50E-07
centroid_15314	putative membrane protein	10	0	21	94	centroid_15314	28.722145	8.35E-08	centroid_15314	2.50E-07
centroid_15322	polysaccharide biosynthesis/export family protein	10	0	21	94	centroid_15322	28.722145	8.35E-08	centroid_15322	2.50E-07
centroid_15323	ABC-2 type transporter family protein	10	0	21	94	centroid_15323	28.722145	8.35E-08	centroid_15323	2.50E-07
centroid_15324	ABC transporter family protein	10	0	21	94	centroid_15324	28.722145	8.35E-08	centroid_15324	2.50E-07
centroid_15325	lysaccharide export inner-membrane , BexC/CtrB/KpsE family prot	10	0	21	94	centroid_15325	28.722145	8.35E-08	centroid_15325	2.50E-07
centroid_15326	methyltransferase domain protein	10	0	21	94	centroid_15326	28.722145	8.35E-08	centroid_15326	2.50E-07
centroid_15327	glycosyltransferase WbsX family protein	10	0	21	94	centroid_15327	28.722145	8.35E-08	centroid_15327	2.50E-07
centroid_15328	glycosyl transferase 2 family protein	10	0	21	94	centroid_15328	28.722145	8.35E-08	centroid_15328	2.50E-07
centroid_15329	nucleotide sugar dehydrogenase family protein	10	0	21	94	centroid_15329	28.722145	8.35E-08	centroid_15329	2.50E-07
centroid_15330	hypothetical protein	10	0	21	94	centroid_15330	28.722145	8.35E-08	centroid_15330	2.50E-07
centroid_15331	glycosyl transferase 2 family protein	10	0	21	94	centroid_15331	28.722145	8.35E-08	centroid_15331	2.50E-07
centroid_15332	glycosyl transferase 2 family protein	10	0	21	94	centroid_15332	28.722145	8.35E-08	centroid_15332	2.50E-07
centroid_15333	glycosyl transferases group 1 family protein	10	0	21	94	centroid_15333	28.722145	8.35E-08	centroid_15333	2.50E-07
centroid_15334	dTDP-glucose 4,6-dehydratase	10	0	21	94	centroid_15334	28.722145	8.35E-08	centroid_15334	2.50E-07
centroid_15335	dTDP-4-dehydrorhamnose reductase	10	0	21	94	centroid_15335	28.722145	8.35E-08	centroid_15335	2.50E-07
centroid_15336	glucose-1-phosphate thymidylyltransferase	10	0	21	94	centroid_15336	28.722145	8.35E-08	centroid_15336	2.50E-07
centroid_15337	dTDP-4-dehydrorhamnose 3,5-epimerase	10	0	21	94	centroid_15337	28.722145	8.35E-08	centroid_15337	2.50E-07
centroid_15338	capsule polysaccharide biosynthesis family protein	10	0	21	94	centroid_15338	28.722145	8.35E-08	centroid_15338	2.50E-07
centroid_15339	capsule polysaccharide biosynthesis family protein	10	0	21	94	centroid_15339	28.722145	8.35E-08	centroid_15339	2.50E-07
centroid_15340	putative pH01	10	0	21	94	centroid_15340	28.722145	8.35E-08	centroid_15340	2.50E-07
centroid_15347	DNA transfer protein gp7	10	0	21	94	centroid_15347	28.722145	8.35E-08	centroid_15347	2.50E-07
centroid_15353	type I restriction modification DNA specificity domain protein	10	0	21	94	centroid_15353	28.722145	8.35E-08	centroid_15353	2.50E-07
centroid_15354	yecA family protein	10	0	21	94	centroid_15354	28.722145	8.35E-08	centroid_15354	2.50E-07
centroid_15355	'Cold-shock' DNA-binding domain protein	10	0	21	94	centroid_15355	28.722145	8.35E-08	centroid_15355	2.50E-07
centroid_15392	putative outer membrane lipoprotein SlyB	10	0	21	94	centroid_15392	28.722145	8.35E-08	centroid_15392	2.50E-07
centroid_15789	adenine-specific methyltransferase EcoRI family protein	10	0	21	94	centroid_15789	28.722145	8.35E-08	centroid_15789	2.50E-07
centroid_15790	type-2 restriction enzyme EcoRI	10	0	21	94	centroid_15790	28.722145	8.35E-08	centroid_15790	2.50E-07
centroid_15882	tail spike protein	10	0	21	94	centroid_15882	28.722145	8.35E-08	centroid_15882	2.50E-07
centroid_17652	conserved hypothetical protein	10	0	21	94	centroid_17652	28.722145	8.35E-08	centroid_17652	2.50E-07
centroid_13199	phage late control gene D family protein	10	77	21	17	centroid_13199	24.871589	6.13E-07	centroid_13199	6.06E-07
centroid_8076	glutamate decarboxylase	10	77	21	17	centroid_8076	24.871589	6.13E-07	centroid_8076	6.06E-07
centroid_12069	ncrBC 5-methylcytosine restriction system component family protei	10	1	21	93	centroid_12069	24.511893	7.39E-07	centroid_12069	2.29E-06
centroid_12907	putative transposase	10	1	21	93	centroid_12907	24.511893	7.39E-07	centroid_12907	2.29E-06
centroid_15239	integrase core domain protein	10	1	21	93	centroid_15239	24.511893	7.39E-07	centroid_15239	2.29E-06
centroid_15272	dTDP-4-dehydrorhamnose 3,5-epimerase	10	1	21	93	centroid_15272	24.511893	7.39E-07	centroid_15272	2.29E-06
centroid_15273	glycosyl transferase 2 family protein	10	1	21	93	centroid_15273	24.511893	7.39E-07	centroid_15273	2.29E-06
centroid_15274	glycosyl transferases group 1 family protein	10	1	21	93	centroid_15274	24.511893	7.39E-07	centroid_15274	2.29E-06
centroid_15275	putative membrane protein	10	1	21	93	centroid_15275	24.511893	7.39E-07	centroid_15275	2.29E-06
centroid_15276	bacterial transferase hexapeptide family protein	10	1	21	93	centroid_15276	24.511893	7.39E-07	centroid_15276	2.29E-06
centroid_15277	UDP-N-acetylglucosamine 4,6-dehydratase/5-epimerase	10	1	21	93	centroid_15277	24.511893	7.39E-07	centroid_15277	2.29E-06
centroid_15738	putative mobilization protein 1	10	1	21	93	centroid_15738	24.511893	7.39E-07	centroid_15738	2.29E-06
centroid_16486	AAA domain family protein	10	1	21	93	centroid_16486	24.511893	7.39E-07	centroid_16486	2.29E-06
centroid_17513	methyltransferase domain protein	10	1	21	93	centroid_17513	24.511893	7.39E-07	centroid_17513	2.29E-06
centroid_18276	polysaccharide biosynthesis family protein	10	1	21	93	centroid_18276	24.511893	7.39E-07	centroid_18276	2.29E-06
centroid_6660	coA-transferase III family protein	10	1	21	93	centroid_6660	24.511893	7.39E-07	centroid_6660	2.29E-06
centroid_6661	HMGL-like family protein	10	1	21	93	centroid_6661	24.511893	7.39E-07	centroid_6661	2.29E-06
centroid_6662	sugar (and other) transporter family protein	10	1	21	93	centroid_6662	24.511893	7.39E-07	centroid_6662	2.29E-06
centroid_8929	integrase core domain protein	10	1	21	93	centroid_8929	24.511893	7.39E-07	centroid_8929	2.29E-06
centroid_9093	ead/Ea22-like family protein	10	1	21	93	centroid_9093	24.511893	7.39E-07	centroid_9093	2.29E-06
centroid_16096	phage late control gene D family protein	10	76	21	18	centroid_16096	23.430088	1.30E-06	centroid_16096	1.17E-06
centroid_18677	phage tail sheath family protein	10	76	21	18	centroid_18677	23.430088	1.30E-06	centroid_18677	1.17E-06
centroid_7906	phage late control gene D family protein	10	76	21	18	centroid_7906	23.430088	1.30E-06	centroid_7906	1.17E-06
centroid_10219	phage tail sheath family protein	10	75	21	19	centroid_10219	22.066472	2.63E-06	centroid_10219	2.90E-06
centroid_10421	conserved predicted domain protein	10	75	21	19	centroid_10421	22.066472	2.63E-06	centroid_10421	2.90E-06
centroid_8326	putative conserved predicted protein	10	75	21	19	centroid_8326	22.066472	2.63E-06	centroid_8326	2.90E-06
centroid_11693	bacteriophage lysis family protein	10	2	21	92	centroid_11693	21.038206	4.50E-06	centroid_11693	1.15E-05
centroid_11872	cupin fold metallo, WbuC family protein	10	2	21	92	centroid_11872	21.038206	4.50E-06	centroid_11872	1.15E-05
centroid_11873	glycosyl transferases group 1 family protein	10	2	21	92	centroid_11873	21.038206	4.50E-06	centroid_11873	1.15E-05
centroid_11874	UDP-N-acetylglucosamine 2-epimerase	10	2	21	92	centroid_11874	21.038206	4.50E-06	centroid_11874	1.15E-05
centroid_11875	NAD dependent epimerase/dehydratase family protein	10	2	21	92	centroid_11875	21.038206	4.50E-06	centroid_11875	1.15E-05
centroid_11877	glycosyl transferases group 1 family protein	10	2	21	92	centroid_11877	21.038206	4.50E-06	centroid_11877	1.15E-05
centroid_15906	N-6 DNA Methylase family protein	10	2	21	92	centroid_15906	21.038206	4.50E-06	centroid_15906	1.15E-05
centroid_18675	putative variable tail fiber protein	10	2	21	92	centroid_18675	21.038206	4.50E-06	centroid_18675	1.15E-05
centroid_5615	resolvase, N terminal domain protein	10	2	21	92	centroid_5615	21.038206	4.50E-06	centroid_5615	1.15E-05
centroid_6014	conserved hypothetical protein	10	2	21	92	centroid_6014	21.038206	4.50E-06	centroid_6014	1.15E-05

centroid_6015	conserved hypothetical protein	10	2	21	92	centroid_6015	21.038206	4.50E-06	centroid_6015	1.15E-05
centroid_6016	conserved hypothetical protein	10	2	21	92	centroid_6016	21.038206	4.50E-06	centroid_6016	1.15E-05
centroid_6017	conserved hypothetical protein	10	2	21	92	centroid_6017	21.038206	4.50E-06	centroid_6017	1.15E-05
centroid_6018	putative translation elongation factor P domain protein	10	2	21	92	centroid_6018	21.038206	4.50E-06	centroid_6018	1.15E-05
centroid_6019	conserved hypothetical protein	10	2	21	92	centroid_6019	21.038206	4.50E-06	centroid_6019	1.15E-05
centroid_3640	phage tail sheath family protein	10	74	21	20	centroid_3640	20.775245	5.16E-06	centroid_3640	4.45E-06
centroid_3835	putative conserved predicted protein	10	74	21	20	centroid_3835	20.775245	5.16E-06	centroid_3835	4.45E-06
centroid_13223	conserved hypothetical protein	10	3	21	91	centroid_13223	18.13197	2.06E-05	centroid_13223	4.13E-05
centroid_14810	transposase IS116/IS110/IS902 family protein	10	3	21	91	centroid_14810	18.13197	2.06E-05	centroid_14810	4.13E-05
centroid_15411	protein CexE	10	3	21	91	centroid_15411	18.13197	2.06E-05	centroid_15411	4.13E-05
centroid_15980	phage terminase, large subunit, PBSX family	10	3	21	91	centroid_15980	18.13197	2.06E-05	centroid_15980	4.13E-05
centroid_17015	gram-negative pili assembly chaperone, N-terminal domain protein	10	3	21	91	centroid_17015	18.13197	2.06E-05	centroid_17015	4.13E-05
centroid_18633	putative transposase	10	3	21	91	centroid_18633	18.13197	2.06E-05	centroid_18633	4.13E-05
centroid_5999	transposase family protein	10	3	21	91	centroid_5999	18.13197	2.06E-05	centroid_5999	4.13E-05
centroid_6000	ABC transporter family protein	10	3	21	91	centroid_6000	18.13197	2.06E-05	centroid_6000	4.13E-05
centroid_6001	putative transporter protein AatB	10	3	21	91	centroid_6001	18.13197	2.06E-05	centroid_6001	4.13E-05
centroid_6002	outer membrane efflux family protein	10	3	21	91	centroid_6002	18.13197	2.06E-05	centroid_6002	4.13E-05
centroid_6003	permease family protein	10	3	21	91	centroid_6003	18.13197	2.06E-05	centroid_6003	4.13E-05
centroid_6059	serine protease SepA autotransporter	10	3	21	91	centroid_6059	18.13197	2.06E-05	centroid_6059	4.13E-05
centroid_6062	type VII secretion system (T7SS), usher family protein	10	3	21	91	centroid_6062	18.13197	2.06E-05	centroid_6062	4.13E-05
centroid_6066	putative membrane protein	10	3	21	91	centroid_6066	18.13197	2.06E-05	centroid_6066	4.13E-05
centroid_6068	hypothetical protein	10	3	21	91	centroid_6068	18.13197	2.06E-05	centroid_6068	4.13E-05
centroid_6894	integrase core domain protein	10	3	21	91	centroid_6894	18.13197	2.06E-05	centroid_6894	4.13E-05
centroid_9784	ead/Ea22-like family protein	10	3	21	91	centroid_9784	18.13197	2.06E-05	centroid_9784	4.13E-05
centroid_7843	phage tail tape measure protein, TP901 family, core region	10	71	21	23	centroid_7843	17.288561	3.21E-05	centroid_7843	2.40E-05
centroid_10381	homeo-like domain protein	10	4	21	90	centroid_10381	15.672416	7.53E-05	centroid_10381	0.0001202
centroid_12479	conserved hypothetical protein	10	4	21	90	centroid_12479	15.672416	7.53E-05	centroid_12479	0.0001202
centroid_15760	conserved hypothetical protein	10	4	21	90	centroid_15760	15.672416	7.53E-05	centroid_15760	0.0001202
centroid_15761	qnrB1	10	4	21	90	centroid_15761	15.672416	7.53E-05	centroid_15761	0.0001202
centroid_16135	hypothetical protein	10	4	21	90	centroid_16135	15.672416	7.53E-05	centroid_16135	0.0001202
centroid_18634	type VII secretion system (T7SS), usher family protein	10	4	21	90	centroid_18634	15.672416	7.53E-05	centroid_18634	0.0001202
centroid_6061	SEF14-like adhesin family protein	10	4	21	90	centroid_6061	15.672416	7.53E-05	centroid_6061	0.0001202
centroid_6067	hypothetical protein	10	4	21	90	centroid_6067	15.672416	7.53E-05	centroid_6067	0.0001202
centroid_13561	phage integrase family protein	10	69	21	25	centroid_13561	15.24665	9.43E-05	centroid_13561	7.40E-05
centroid_15218	prophage tail fibre N-terminal family protein	9	0	22	94	centroid_15218	25.223071	5.11E-07	centroid_15218	1.32E-06
centroid_15223	conserved hypothetical protein	9	0	22	94	centroid_15223	25.223071	5.11E-07	centroid_15223	1.32E-06
centroid_15224	putative transcriptional regulator	9	0	22	94	centroid_15224	25.223071	5.11E-07	centroid_15224	1.32E-06
centroid_15231	lysR family regulatory helix-turn-helix protein	9	0	22	94	centroid_15231	25.223071	5.11E-07	centroid_15231	1.32E-06
centroid_15262	hypothetical protein	9	0	22	94	centroid_15262	25.223071	5.11E-07	centroid_15262	1.32E-06
centroid_15285	conserved hypothetical protein	9	0	22	94	centroid_15285	25.223071	5.11E-07	centroid_15285	1.32E-06
centroid_15357	hypothetical protein	9	0	22	94	centroid_15357	25.223071	5.11E-07	centroid_15357	1.32E-06
centroid_15365	phage minor tail protein L	9	0	22	94	centroid_15365	25.223071	5.11E-07	centroid_15365	1.32E-06
centroid_15376	conserved hypothetical protein	9	0	22	94	centroid_15376	25.223071	5.11E-07	centroid_15376	1.32E-06
centroid_15377	P63C domain protein	9	0	22	94	centroid_15377	25.223071	5.11E-07	centroid_15377	1.32E-06
centroid_15379	DNA transfer gp20 domain protein	9	0	22	94	centroid_15379	25.223071	5.11E-07	centroid_15379	1.32E-06
centroid_3639	phage late control gene D family protein	9	73	22	21	centroid_3639	22.320102	2.31E-06	centroid_3639	1.82E-06
centroid_11855	ninB family protein	9	1	22	93	centroid_11855	21.122029	4.31E-06	centroid_11855	1.09E-05
centroid_12068	ATPase associated with various cellular activities family protein	9	1	22	93	centroid_12068	21.122029	4.31E-06	centroid_12068	1.09E-05
centroid_12109	conserved hypothetical protein	9	1	22	93	centroid_12109	21.122029	4.31E-06	centroid_12109	1.09E-05
centroid_13971	conserved hypothetical protein	9	1	22	93	centroid_13971	21.122029	4.31E-06	centroid_13971	1.09E-05
centroid_13972	ERF superfamily protein	9	1	22	93	centroid_13972	21.122029	4.31E-06	centroid_13972	1.09E-05
centroid_13980	dnaB-like helicase C terminal domain protein	9	1	22	93	centroid_13980	21.122029	4.31E-06	centroid_13980	1.09E-05
centroid_15260	phage tail fibre repeat family protein	9	1	22	93	centroid_15260	21.122029	4.31E-06	centroid_15260	1.09E-05
centroid_15381	putative transposase	9	1	22	93	centroid_15381	21.122029	4.31E-06	centroid_15381	1.09E-05
centroid_15548	hypothetical protein	9	1	22	93	centroid_15548	21.122029	4.31E-06	centroid_15548	1.09E-05
centroid_17479	putative yacA	9	1	22	93	centroid_17479	21.122029	4.31E-06	centroid_17479	1.09E-05
centroid_17625	conserved hypothetical protein	9	1	22	93	centroid_17625	21.122029	4.31E-06	centroid_17625	1.09E-05
centroid_13238	phage tail tube FII family protein	9	72	22	22	centroid_13238	21.082915	4.40E-06	centroid_13238	3.06E-06
centroid_10882	phage terminase, large subunit, PBSX family	9	2	22	92	centroid_10882	17.807199	2.44E-05	centroid_10882	4.98E-05
centroid_14636	conserved hypothetical protein	9	2	22	92	centroid_14636	17.807199	2.44E-05	centroid_14636	4.98E-05
centroid_14649	conserved hypothetical protein	9	2	22	92	centroid_14649	17.807199	2.44E-05	centroid_14649	4.98E-05
centroid_16817	prophage CP4-57 regulatory family protein	9	2	22	92	centroid_16817	17.807199	2.44E-05	centroid_16817	4.98E-05
centroid_17573	nucleotidyltransferase domain protein	9	2	22	92	centroid_17573	17.807199	2.44E-05	centroid_17573	4.98E-05
centroid_5614	hypothetical protein	9	2	22	92	centroid_5614	17.807199	2.44E-05	centroid_5614	4.98E-05
centroid_6012	conserved hypothetical protein	9	2	22	92	centroid_6012	17.807199	2.44E-05	centroid_6012	4.98E-05
centroid_6013	conserved hypothetical protein	9	2	22	92	centroid_6013	17.807199	2.44E-05	centroid_6013	4.98E-05
centroid_11068	conserved hypothetical protein	9	67	22	27	centroid_11068	15.727809	7.31E-05	centroid_11068	4.45E-05
centroid_3641	phage major tail tube protein	8	72	23	22	centroid_3641	23.942203	9.93E-07	centroid_3641	6.96E-07
centroid_14812	transposase IS66 family protein	8	0	23	94	centroid_14812	21.787789	3.05E-06	centroid_14812	6.71E-06
centroid_15184	DNA (cytosine-5-)-methyltransferase family protein	8	0	23	94	centroid_15184	21.787789	3.05E-06	centroid_15184	6.71E-06
centroid_15225	phage integrase family protein	8	0	23	94	centroid_15225	21.787789	3.05E-06	centroid_15225	6.71E-06
centroid_15375	conserved hypothetical protein	8	0	23	94	centroid_15375	21.787789	3.05E-06	centroid_15375	6.71E-06
centroid_15883	putative dNA transfer protein gp20	8	0	23	94	centroid_15883	21.787789	3.05E-06	centroid_15883	6.71E-06
centroid_15929	phage tail fibre repeat family protein	8	0	23	94	centroid_15929	21.787789	3.05E-06	centroid_15929	6.71E-06
centroid_15961	conserved hypothetical protein	8	0	23	94	centroid_15961	21.787789	3.05E-06	centroid_15961	6.71E-06
centroid_15968	conserved hypothetical protein	8	0	23	94	centroid_15968	21.787789	3.05E-06	centroid_15968	6.71E-06
centroid_11979	helix-turn-helix family protein	8	1	23	93	centroid_11979	17.816875	2.43E-05	centroid_11979	4.98E-05
centroid_11982	conserved hypothetical protein	8	1	23	93	centroid_11982	17.816875	2.43E-05	centroid_11982	4.98E-05
centroid_13974	conserved hypothetical protein	8	1	23	93	centroid_13974	17.816875	2.43E-05	centroid_13974	4.98E-05
centroid_15123	conserved hypothetical protein	8	1	23	93	centroid_15123	17.816875	2.43E-05	centroid_15123	4.98E-05
centroid_15893	peptidase S24-like family protein	8	1	23	93	centroid_15893	17.816875	2.43E-05	centroid_15893	4.98E-05
centroid_15923	dihydrofolate reductase type 1	8	1	23	93	centroid_15923	17.816875	2.43E-05	centroid_15923	4.98E-05
centroid_6230	helix-turn-helix domain protein	8	1	23	93	centroid_6230	17.816875	2.43E-05	centroid_6230	4.98E-05
centroid_6231	hypothetical protein	8	1	23	93	centroid_6231	17.816875	2.43E-05	centroid_6231	4.98E-05
centroid_10126	conserved hypothetical protein	8	66	23	28	centroid_10126	17.238021	3.30E-05	centroid_10126	1.83E-05
centroid_5033	conserved hypothetical protein	8	66	23	28	centroid_5033	17.238021	3.30E-05	centroid_5033	1.83E-05
centroid_4244	conserved hypothetical protein	7	77	24	17	centroid_4244	34.591386	4.07E-09	centroid_4244	3.47E-09
centroid_5227	conserved hypothetical protein	7	76	24	18	centroid_5227	32.915024	9.63E-09	centroid_5227	7.45E-09
centroid_14501	regulatory protein rop	7	0	24	94	centroid_14501	18.416367	1.78E-05	centroid_14501	3.30E-05
centroid_15880	conserved hypothetical protein	7	0	24	94	centroid_15880	18.416367	1.78E-05	centroid_15880	3.30E-05
centroid_15889	putative membrane protein	7	0	24	94	centroid_15889	18.416367	1.78E-05	centroid_15889	3.30E-05
centroid_15890	conserved hypothetical protein	7	0	24	94	centroid_15890	18.416367	1.78E-05	centroid_15890	3.30E-05
centroid_15891	bacteriophage replication O family protein	7	0	24	94	centroid_15891	18.416367	1.78E-05	centroid_15891	3.30E-05
centroid_15892	conserved hypothetical protein	7	0	24	94	centroid_15892	18.416367	1.78E-05	centroid_15892	3.30E-05
centroid_15895	conserved hypothetical protein	7	0	24	94	centroid_15895	18.416367	1.78E-05	centroid_15895	3.30E-05
centroid_15896	ead/Ea22-like family protein	7	0	24	94	centroid_15896	18.416367	1.78E-05	centroid_15896	3.30E-05
centroid_15909	conserved hypothetical protein	7	0	24	94	centroid_15909	18.416367	1.78E-05	centroid_15909	3.30E-05
centroid_15910	conserved hypothetical protein	7	0	24	94	centroid_15910	18.416367	1.78E-05	centroid_15910	3.30E-05
centroid_15911	putative korC protein	7	0	24	94	centroid_15911	18.416367	1.78E-05	centroid_15911	3.30E-05
centroid_16923	repressor protein C2	7	0	24	94	centroid_16923	18.416367	1.78E-05	centroid_16923	3.30E-05
centroid_17409	hypothetical protein	7	0	24	94	centroid_17409	18.416367	1.78E-05	centroid_17409	3.30E-05
centroid_5110	PIN domain protein	5	59	26	35	centroid_5110	18.469524	1.73E-05	centroid_5110	7.23E-06
centroid_10248	bacteriophage replication gene A family protein	5	57	26	37	centroid_10248	16.736722	4.29E-05	centroid_10248	2.03E-05
centroid_5169	putative membrane protein	1	43	30	51	centroid_5169	16.659679	4.47E-05	centroid_5169	6.00E-06
centroid_14162	initiator Replication family protein	0	43	31	51	centroid_14162	19.637559	9.36E-06	centroid_14162	3.55E-07
centroid_4143	initiator Replication family protein	0	42	31	52	centroid_4143	18.905412	1.37E-05	centroid_4143	3.74E-07

centroid_10641	hypothetical protein	0	39	31	55	centroid_10641	16.811464	4.13E-05	centroid_10641	2.27E-06
centroid_3978	R.Ecl18kI	0	39	31	55	centroid_3978	16.811464	4.13E-05	centroid_3978	2.27E-06
centroid_3980	conserved hypothetical protein	0	39	31	55	centroid_3980	16.811464	4.13E-05	centroid_3980	2.27E-06
centroid_3979	DNA (cytosine-5-)-methyltransferase family protein	0	37	31	57	centroid_3979	15.495128	8.27E-05	centroid_3979	5.36E-06

Table S4. Distribution by Geography - Calama

Gene_ID	Annotation	alama_prese	Other_preser	alama_Abse	Other_Absent	Gene_ID	chisq-stats	pvalues	Gene_ID	pvalues
centroid_17039	hypothetical protein	4	119	2	0	centroid_17039	21.919515	2.84E-06	centroid_17039	0.0019355
centroid_14919	putative taxA	4	7	2	112	centroid_14919	19.26779	1.14E-05	centroid_14919	0.0004645
centroid_14917	firmicute plasmid replication family protein	4	8	2	111	centroid_14917	17.247529	3.28E-05	centroid_14917	0.0006871
centroid_14918	putative oRF3	4	8	2	111	centroid_14918	17.247529	3.28E-05	centroid_14918	0.0006871
centroid_15409	plasmid mobilization domain protein	3	1	3	118	centroid_15409	30.106348	4.09E-08	centroid_15409	0.0002471
centroid_14646	putative membrane protein	3	4	3	115	centroid_14646	15.508375	8.21E-05	centroid_14646	0.0020437
centroid_14860	conserved hypothetical protein	3	4	3	115	centroid_14860	15.508375	8.21E-05	centroid_14860	0.0020437
centroid_14920	conserved hypothetical protein	3	4	3	115	centroid_14920	15.508375	8.21E-05	centroid_14920	0.0020437
centroid_15649	putative mobC	3	4	3	115	centroid_15649	15.508375	8.21E-05	centroid_15649	0.0020437
centroid_15650	mobA/MobL family protein	3	4	3	115	centroid_15650	15.508375	8.21E-05	centroid_15650	0.0020437
centroid_15651	mobilization A domain protein	3	4	3	115	centroid_15651	15.508375	8.21E-05	centroid_15651	0.0020437
centroid_15652	hypothetical protein	3	4	3	115	centroid_15652	15.508375	8.21E-05	centroid_15652	0.0020437
centroid_15653	putative repF	3	4	3	115	centroid_15653	15.508375	8.21E-05	centroid_15653	0.0020437
centroid_15654	regulatory protein RepA	3	4	3	115	centroid_15654	15.508375	8.21E-05	centroid_15654	0.0020437
centroid_15655	replication C family protein	3	4	3	115	centroid_15655	15.508375	8.21E-05	centroid_15655	0.0020437
centroid_16122	mobilization protein A	3	4	3	115	centroid_16122	15.508375	8.21E-05	centroid_16122	0.0020437
centroid_18288	phage tail protein I	2	0	4	119	centroid_18288	21.919515	2.84E-06	centroid_18288	0.0019355
centroid_18289	phage baseplate assembly V family protein	2	0	4	119	centroid_18289	21.919515	2.84E-06	centroid_18289	0.0019355
centroid_18290	phage late control gene D family protein	2	0	4	119	centroid_18290	21.919515	2.84E-06	centroid_18290	0.0019355
centroid_18291	phage P2 GpU family protein	2	0	4	119	centroid_18291	21.919515	2.84E-06	centroid_18291	0.0019355
centroid_18292	phage tail tape measure protein, TP901 family, core region	2	0	4	119	centroid_18292	21.919515	2.84E-06	centroid_18292	0.0019355
centroid_18294	conserved hypothetical protein	2	0	4	119	centroid_18294	21.919515	2.84E-06	centroid_18294	0.0019355
centroid_18295	phage major tail tube protein	2	0	4	119	centroid_18295	21.919515	2.84E-06	centroid_18295	0.0019355
centroid_18296	gp37 family protein	2	0	4	119	centroid_18296	21.919515	2.84E-06	centroid_18296	0.0019355
centroid_18297	conserved hypothetical protein	2	0	4	119	centroid_18297	21.919515	2.84E-06	centroid_18297	0.0019355
centroid_18298	putative dihydrolipoamide acyltransferase	2	0	4	119	centroid_18298	21.919515	2.84E-06	centroid_18298	0.0019355
centroid_18299	conserved hypothetical protein	2	0	4	119	centroid_18299	21.919515	2.84E-06	centroid_18299	0.0019355
centroid_18300	putative protease	2	0	4	119	centroid_18300	21.919515	2.84E-06	centroid_18300	0.0019355
centroid_18301	conserved hypothetical protein	2	0	4	119	centroid_18301	21.919515	2.84E-06	centroid_18301	0.0019355
centroid_18302	terminase-like family protein	2	0	4	119	centroid_18302	21.919515	2.84E-06	centroid_18302	0.0019355
centroid_18303	conserved hypothetical protein	2	0	4	119	centroid_18303	21.919515	2.84E-06	centroid_18303	0.0019355
centroid_18304	putative phage membrane protein	2	0	4	119	centroid_18304	21.919515	2.84E-06	centroid_18304	0.0019355
centroid_18307	conserved hypothetical protein	2	0	4	119	centroid_18307	21.919515	2.84E-06	centroid_18307	0.0019355
centroid_18308	DNA adenine methylase family protein	2	0	4	119	centroid_18308	21.919515	2.84E-06	centroid_18308	0.0019355
centroid_18309	conserved hypothetical protein	2	0	4	119	centroid_18309	21.919515	2.84E-06	centroid_18309	0.0019355
centroid_18310	helix-turn-helix family protein	2	0	4	119	centroid_18310	21.919515	2.84E-06	centroid_18310	0.0019355
centroid_18311	iclR helix-turn-helix domain protein	2	0	4	119	centroid_18311	21.919515	2.84E-06	centroid_18311	0.0019355
centroid_18313	integrase core domain protein	2	0	4	119	centroid_18313	21.919515	2.84E-06	centroid_18313	0.0019355
centroid_18314	AAA domain protein	2	0	4	119	centroid_18314	21.919515	2.84E-06	centroid_18314	0.0019355
centroid_18315	putative prophage exported protein	2	0	4	119	centroid_18315	21.919515	2.84E-06	centroid_18315	0.0019355
centroid_18316	conserved hypothetical protein	2	0	4	119	centroid_18316	21.919515	2.84E-06	centroid_18316	0.0019355
centroid_18317	conserved hypothetical protein	2	0	4	119	centroid_18317	21.919515	2.84E-06	centroid_18317	0.0019355
centroid_18318	conserved hypothetical protein	2	0	4	119	centroid_18318	21.919515	2.84E-06	centroid_18318	0.0019355
centroid_18319	conserved hypothetical protein	2	0	4	119	centroid_18319	21.919515	2.84E-06	centroid_18319	0.0019355
centroid_18320	putative membrane protein	2	0	4	119	centroid_18320	21.919515	2.84E-06	centroid_18320	0.0019355
centroid_18364	conserved hypothetical protein	2	0	4	119	centroid_18364	21.919515	2.84E-06	centroid_18364	0.0019355
centroid_18366	putative membrane protein	2	0	4	119	centroid_18366	21.919515	2.84E-06	centroid_18366	0.0019355
centroid_18367	hypothetical protein	2	0	4	119	centroid_18367	21.919515	2.84E-06	centroid_18367	0.0019355
centroid_18368	conjugal transfer/type IV secretion DotA/TraY family protein	2	0	4	119	centroid_18368	21.919515	2.84E-06	centroid_18368	0.0019355
centroid_18369	hypothetical protein	2	0	4	119	centroid_18369	21.919515	2.84E-06	centroid_18369	0.0019355
centroid_18370	putative membrane protein	2	0	4	119	centroid_18370	21.919515	2.84E-06	centroid_18370	0.0019355
centroid_18371	hypothetical protein	2	0	4	119	centroid_18371	21.919515	2.84E-06	centroid_18371	0.0019355
centroid_18372	hypothetical protein	2	0	4	119	centroid_18372	21.919515	2.84E-06	centroid_18372	0.0019355
centroid_18373	hypothetical protein	2	0	4	119	centroid_18373	21.919515	2.84E-06	centroid_18373	0.0019355
centroid_18374	AAA domain protein	2	0	4	119	centroid_18374	21.919515	2.84E-06	centroid_18374	0.0019355
centroid_18375	conserved hypothetical protein	2	0	4	119	centroid_18375	21.919515	2.84E-06	centroid_18375	0.0019355
centroid_18376	type III restriction-modification system EcoPI enzyme mod	2	0	4	119	centroid_18376	21.919515	2.84E-06	centroid_18376	0.0019355
centroid_18377	type III restriction enzyme, res subunit	2	0	4	119	centroid_18377	21.919515	2.84E-06	centroid_18377	0.0019355
centroid_18378	hypothetical protein	2	0	4	119	centroid_18378	21.919515	2.84E-06	centroid_18378	0.0019355
centroid_18379	hypothetical protein	2	0	4	119	centroid_18379	21.919515	2.84E-06	centroid_18379	0.0019355
centroid_18380	putative membrane protein	2	0	4	119	centroid_18380	21.919515	2.84E-06	centroid_18380	0.0019355
centroid_18381	hypothetical protein	2	0	4	119	centroid_18381	21.919515	2.84E-06	centroid_18381	0.0019355
centroid_18384	NACHT domain protein	2	0	4	119	centroid_18384	21.919515	2.84E-06	centroid_18384	0.0019355
centroid_18385	hypothetical protein	2	0	4	119	centroid_18385	21.919515	2.84E-06	centroid_18385	0.0019355
centroid_18386	conserved hypothetical protein	2	0	4	119	centroid_18386	21.919515	2.84E-06	centroid_18386	0.0019355
centroid_18387	transposase IS116/IS110/IS902 family protein	2	0	4	119	centroid_18387	21.919515	2.84E-06	centroid_18387	0.0019355
centroid_18388	plasmid partitioning protein ParF	2	0	4	119	centroid_18388	21.919515	2.84E-06	centroid_18388	0.0019355
centroid_18389	plasmid partition protein ParG	2	0	4	119	centroid_18389	21.919515	2.84E-06	centroid_18389	0.0019355
centroid_18390	ribbon-helix-helix , copG family protein	2	0	4	119	centroid_18390	21.919515	2.84E-06	centroid_18390	0.0019355
centroid_18391	conserved hypothetical protein	2	0	4	119	centroid_18391	21.919515	2.84E-06	centroid_18391	0.0019355
centroid_18392	conserved hypothetical protein	2	0	4	119	centroid_18392	21.919515	2.84E-06	centroid_18392	0.0019355
centroid_18393	conserved hypothetical protein	2	0	4	119	centroid_18393	21.919515	2.84E-06	centroid_18393	0.0019355
centroid_18394	initiator Replication family protein	2	0	4	119	centroid_18394	21.919515	2.84E-06	centroid_18394	0.0019355
centroid_18395	PI protein	2	0	4	119	centroid_18395	21.919515	2.84E-06	centroid_18395	0.0019355
centroid_18396	conserved hypothetical protein	2	0	4	119	centroid_18396	21.919515	2.84E-06	centroid_18396	0.0019355
centroid_18431	putative membrane protein	2	0	4	119	centroid_18431	21.919515	2.84E-06	centroid_18431	0.0019355
centroid_18433	conserved hypothetical protein	2	0	4	119	centroid_18433	21.919515	2.84E-06	centroid_18433	0.0019355
centroid_18434	putative membrane protein	2	0	4	119	centroid_18434	21.919515	2.84E-06	centroid_18434	0.0019355
centroid_18435	sigma-54 interaction domain protein	2	0	4	119	centroid_18435	21.919515	2.84E-06	centroid_18435	0.0019355
centroid_18436	conserved hypothetical protein	2	0	4	119	centroid_18436	21.919515	2.84E-06	centroid_18436	0.0019355
centroid_18439	trbL/VirB6 plasmid conjugal transfer family protein	2	0	4	119	centroid_18439	21.919515	2.84E-06	centroid_18439	0.0019355
centroid_18453	putative eex protein	2	0	4	119	centroid_18453	21.919515	2.84E-06	centroid_18453	0.0019355
centroid_18454	bacterial regulatory, luxR family protein	2	0	4	119	centroid_18454	21.919515	2.84E-06	centroid_18454	0.0019355
centroid_18455	arc-like DNA binding domain protein	2	0	4	119	centroid_18455	21.919515	2.84E-06	centroid_18455	0.0019355
centroid_18456	conserved hypothetical protein	2	0	4	119	centroid_18456	21.919515	2.84E-06	centroid_18456	0.0019355
centroid_18458	hypothetical protein	2	0	4	119	centroid_18458	21.919515	2.84E-06	centroid_18458	0.0019355
centroid_18459	conserved hypothetical protein	2	0	4	119	centroid_18459	21.919515	2.84E-06	centroid_18459	0.0019355
centroid_18471	putative plasmid mobilization protein	2	0	4	119	centroid_18471	21.919515	2.84E-06	centroid_18471	0.0019355
centroid_18589	baseplate J-like family protein	2	0	4	119	centroid_18589	21.919515	2.84E-06	centroid_18589	0.0019355
centroid_18593	conserved hypothetical protein	2	0	4	119	centroid_18593	21.919515	2.84E-06	centroid_18593	0.0019355
centroid_18678	type III restriction enzyme, res subunit	2	0	4	119	centroid_18678	21.919515	2.84E-06	centroid_18678	0.0019355
centroid_18679	putative type III restriction-modification system enzyme, res subuni	2	0	4	119	centroid_18679	21.919515	2.84E-06	centroid_18679	0.0019355
centroid_18681	putative taxA	2	0	4	119	centroid_18681	21.919515	2.84E-06	centroid_18681	0.0019355
centroid_11791	conserved hypothetical protein	2	111	4	8	centroid_11791	17.247529	3.28E-05	centroid_11791	0.0006871

Table S5. Distribution by Phylogroup A

Gene_ID	Annotation	Phylogroup A_present	Other_present	Phylogroup A_Absent	Other_Absent	Gene_ID	chisq-stats	pvalues	Gene_ID	pvalues
centroid_17467	type II secretion system protein L	101	1	0	23	centroid_1746	112.320341	3.04001E-26	centroid_1746	3.1685E-24
centroid_3084	BFD-like [2Fe-2S] binding domain protein	101	1	0	23	centroid_3084	112.320341	3.04001E-26	centroid_3084	3.1685E-24
centroid_3085	bacterioferritin	101	1	0	23	centroid_3085	112.320341	3.04001E-26	centroid_3085	3.1685E-24
centroid_3086	e 4 prepilin-like proteins leader peptide-processing enzy	101	1	0	23	centroid_3086	112.320341	3.04001E-26	centroid_3086	3.1685E-24
centroid_3087	type II secretion system (T2SS), M family protein	101	1	0	23	centroid_3087	112.320341	3.04001E-26	centroid_3087	3.1685E-24
centroid_3098	AAA domain protein	101	1	0	23	centroid_3098	112.320341	3.04001E-26	centroid_3098	3.1685E-24
centroid_3099	putative peptidoglycan binding domain protein	101	1	0	23	centroid_3099	112.320341	3.04001E-26	centroid_3099	3.1685E-24
centroid_3100	lcium-binding protein required for initiation of chromosome	101	1	0	23	centroid_3100	112.320341	3.04001E-26	centroid_3100	3.1685E-24
centroid_4710	gntP permease family protein	101	1	0	23	centroid_4710	112.320341	3.04001E-26	centroid_4710	3.1685E-24
centroid_5500	gspL periplasmic domain protein	101	1	0	23	centroid_5500	112.320341	3.04001E-26	centroid_5500	3.1685E-24
centroid_8581	putative general secretion pathway protein A	101	1	0	23	centroid_8581	112.320341	3.04001E-26	centroid_8581	3.1685E-24
centroid_16783	hypothetical protein	101	3	0	21	centroid_1678	100.052169	1.48435E-23	centroid_1678	5.6568E-21
centroid_9217	conserved hypothetical protein	101	3	0	21	centroid_9217	100.052169	1.48435E-23	centroid_9217	5.6568E-21
centroid_1035	2-keto-3-deoxy-L-rhamnonate aldolase	101	4	0	20	centroid_1035	94.0939379	3.00899E-22	centroid_1035	1.4849E-19
centroid_1036	major Facilitator Superfamily protein	101	4	0	20	centroid_1036	94.0939379	3.00899E-22	centroid_1036	1.4849E-19
centroid_1037	major Facilitator Superfamily protein	101	4	0	20	centroid_1037	94.0939379	3.00899E-22	centroid_1037	1.4849E-19
centroid_1038	L-rhamnonate dehydratase	101	4	0	20	centroid_1038	94.0939379	3.00899E-22	centroid_1038	1.4849E-19
centroid_1039	conserved hypothetical protein	101	4	0	20	centroid_1039	94.0939379	3.00899E-22	centroid_1039	1.4849E-19
centroid_14832	putative bifunctional chitinase/lysozyme domain protein	101	4	0	20	centroid_1483	94.0939379	3.00899E-22	centroid_1483	1.4849E-19
centroid_16153	bacterial transcriptional regulator family protein	101	4	0	20	centroid_1615	94.0939379	3.00899E-22	centroid_1615	1.4849E-19
centroid_16154	iclR helix-turn-helix domain protein	101	4	0	20	centroid_1615	94.0939379	3.00899E-22	centroid_1615	1.4849E-19
centroid_2637	conserved hypothetical protein	101	4	0	20	centroid_2637	94.0939379	3.00899E-22	centroid_2637	1.4849E-19
centroid_2747	antitoxin MqsA	101	4	0	20	centroid_2747	94.0939379	3.00899E-22	centroid_2747	1.4849E-19
centroid_4273	inner membrane transport protein RhmT	101	4	0	20	centroid_4273	94.0939379	3.00899E-22	centroid_4273	1.4849E-19
centroid_7695	bacterial transcriptional regulator family protein	101	4	0	20	centroid_7695	94.0939379	3.00899E-22	centroid_7695	1.4849E-19
centroid_7696	iclR helix-turn-helix domain protein	101	4	0	20	centroid_7696	94.0939379	3.00899E-22	centroid_7696	1.4849E-19
centroid_8579	putative bifunctional chitinase/lysozyme domain protein	101	4	0	20	centroid_8579	94.0939379	3.00899E-22	centroid_8579	1.4849E-19
centroid_1113	inner membrane protein YhaI	101	5	0	19	centroid_1113	88.2486029	5.7724E-21	centroid_1113	3.148E-18
centroid_1340	conserved hypothetical protein	101	5	0	19	centroid_1340	88.2486029	5.7724E-21	centroid_1340	3.148E-18
centroid_14713	conserved hypothetical protein	101	5	0	19	centroid_1471	88.2486029	5.7724E-21	centroid_1471	3.148E-18
centroid_14714	conserved hypothetical protein	101	5	0	19	centroid_1471	88.2486029	5.7724E-21	centroid_1471	3.148E-18
centroid_2947	papC N-terminal domain protein	101	6	0	18	centroid_2947	82.5130769	1.04973E-19	centroid_2947	5.614E-17
centroid_9257	protein DedA	101	6	0	18	centroid_9257	82.5130769	1.04973E-19	centroid_9257	5.614E-17
centroid_9965	biquinone/plastoquinone (complex I), various chains fami	101	6	0	18	centroid_9965	82.5130769	1.04973E-19	centroid_9965	5.614E-17
centroid_3231	conserved hypothetical protein	101	7	0	17	centroid_3231	76.884406	1.81263E-18	centroid_3231	8.6616E-16
centroid_5319	conserved hypothetical protein	101	7	0	17	centroid_5319	76.884406	1.81263E-18	centroid_5319	8.6616E-16
centroid_1012	sensory box protein	101	8	0	16	centroid_1012	71.3597684	2.9768E-17	centroid_1012	1.1801E-14
centroid_1013	response regulator	101	8	0	16	centroid_1013	71.3597684	2.9768E-17	centroid_1013	1.1801E-14
centroid_1014	acetate CoA-transferase subunit alpha	101	8	0	16	centroid_1014	71.3597684	2.9768E-17	centroid_1014	1.1801E-14
centroid_1015	acetate CoA-transferase subunit beta	101	8	0	16	centroid_1015	71.3597684	2.9768E-17	centroid_1015	1.1801E-14
centroid_1016	short-chain fatty acids transporter	101	8	0	16	centroid_1016	71.3597684	2.9768E-17	centroid_1016	1.1801E-14
centroid_1017	acetyl-CoA C-acetyltransferase family protein	101	8	0	16	centroid_1017	71.3597684	2.9768E-17	centroid_1017	1.1801E-14
centroid_17737	acterial extracellular solute-binding, 5 Middle family protei	101	8	0	16	centroid_1773	71.3597684	2.9768E-17	centroid_1773	1.1801E-14
centroid_17738	acterial extracellular solute-binding, 5 Middle family protei	101	8	0	16	centroid_1773	71.3597684	2.9768E-17	centroid_1773	1.1801E-14
centroid_2684	conserved hypothetical protein	101	8	0	16	centroid_2684	71.3597684	2.9768E-17	centroid_2684	1.1801E-14
centroid_2685	inner membrane protein YigG	101	8	0	16	centroid_2685	71.3597684	2.9768E-17	centroid_2685	1.1801E-14
centroid_2746	putative binding protein YgiS	101	8	0	16	centroid_2746	71.3597684	2.9768E-17	centroid_2746	1.1801E-14
centroid_7693	ative signal transduction histidine-kinase atoS domain pro	101	8	0	16	centroid_7693	71.3597684	2.9768E-17	centroid_7693	1.1801E-14
centroid_7694	sensory box protein	101	8	0	16	centroid_7694	71.3597684	2.9768E-17	centroid_7694	1.1801E-14
centroid_8264	thiolase, N-terminal domain protein	101	8	0	16	centroid_8264	71.3597684	2.9768E-17	centroid_8264	1.1801E-14
centroid_8265	acetyl-CoA C-acetyltransferase family protein	101	8	0	16	centroid_8265	71.3597684	2.9768E-17	centroid_8265	1.1801E-14
centroid_14303	cyclic di-GMP phosphodiesterase YahA	101	9	0	15	centroid_1430	65.9364765	4.65693E-16	centroid_1430	1.4424E-13
centroid_8383	protein YoaG	101	9	0	15	centroid_8383	65.9364765	4.65693E-16	centroid_8383	1.4424E-13
centroid_17674	conserved hypothetical protein	101	10	0	14	centroid_1767	60.6119817	6.95096E-15	centroid_1767	1.6011E-12
centroid_2839	conserved hypothetical protein	101	10	0	14	centroid_2839	60.6119817	6.95096E-15	centroid_2839	1.6011E-12
centroid_2840	repair family protein	101	10	0	14	centroid_2840	60.6119817	6.95096E-15	centroid_2840	1.6011E-12
centroid_4506	conserved hypothetical protein	101	10	0	14	centroid_4506	60.6119817	6.95096E-15	centroid_4506	1.6011E-12
centroid_4916	conserved hypothetical protein	101	10	0	14	centroid_4916	60.6119817	6.95096E-15	centroid_4916	1.6011E-12
centroid_4431	conserved hypothetical protein	101	12	0	12	centroid_4431	50.2499529	1.35358E-12	centroid_4431	1.5351E-10
centroid_958	pfkB carbohydrate kinase family protein	101	12	0	12	centroid_958	50.2499529	1.35358E-12	centroid_958	1.5351E-10
centroid_2828	FAD dependent oxidoreductase family protein	101	14	0	10	centroid_2828	40.2566836	2.22692E-10	centroid_2828	1.1058E-08
centroid_2829	putative 4Fe-4S binding protein	101	14	0	10	centroid_2829	40.2566836	2.22692E-10	centroid_2829	1.1058E-08
centroid_2830	conserved hypothetical protein	101	14	0	10	centroid_2830	40.2566836	2.22692E-10	centroid_2830	1.1058E-08
centroid_4505	carbohydrate kinase, FGGY family	101	14	0	10	centroid_4505	40.2566836	2.22692E-10	centroid_4505	1.1058E-08
centroid_470	conserved hypothetical protein	101	14	0	10	centroid_470	40.2566836	2.22692E-10	centroid_470	1.1058E-08
centroid_13071	conserved hypothetical protein	101	15	0	9	centroid_1307	35.3940959	2.69302E-09	centroid_1307	8.5512E-08
centroid_18532	type VI secretion system effector, Hcp1 family protein	101	16	0	8	centroid_1853	30.6194119	3.13937E-08	centroid_1853	6.2531E-07
centroid_3606	putative mRNA interferase HicA	101	16	0	8	centroid_3606	30.6194119	3.13937E-08	centroid_3606	6.2531E-07
centroid_848	type VI secretion system effector, Hcp1 family protein	101	16	0	8	centroid_848	30.6194119	3.13937E-08	centroid_848	6.2531E-07
centroid_6124	yhaC domain protein	101	17	0	7	centroid_6124	25.9324288	3.53579E-07	centroid_6124	4.3404E-06
centroid_961	putative pseudouridine transporter	101	17	0	7	centroid_961	25.9324288	3.53579E-07	centroid_961	4.3404E-06
centroid_18274	gram-negative porin family protein	101	18	0	6	centroid_1827	21.3342943	3.85768E-06	centroid_1827	2.8695E-05
centroid_846	conserved hypothetical protein	101	18	0	6	centroid_846	21.3342943	3.85768E-06	centroid_846	2.8695E-05
centroid_847	conserved hypothetical protein	101	18	0	6	centroid_847	21.3342943	3.85768E-06	centroid_847	2.8695E-05
centroid_9306	type VI secretion system effector, Hcp1 family protein	101	18	0	6	centroid_9306	21.3342943	3.85768E-06	centroid_9306	2.8695E-05
centroid_1060	fimbrial family protein	101	19	0	5	centroid_1060	16.8287825	4.09081E-05	centroid_1060	0.00018123
centroid_10769	amino acid permease family protein	101	19	0	5	centroid_1076	16.8287825	4.09081E-05	centroid_1076	0.00018123
centroid_10770	amino acid permease family protein	101	19	0	5	centroid_1077	16.8287825	4.09081E-05	centroid_1077	0.00018123
centroid_11086	o-L-gulonate-6-phosphate decarboxylase UlaD domain p	101	19	0	5	centroid_1108	16.8287825	4.09081E-05	centroid_1108	0.00018123
centroid_11948	GY family of carbohydrate kinase, N-terminal domain pro	101	19	0	5	centroid_1194	16.8287825	4.09081E-05	centroid_1194	0.00018123
centroid_1240	type VII secretion system (T7SS), usher family protein	101	19	0	5	centroid_1240	16.8287825	4.09081E-05	centroid_1240	0.00018123
centroid_1241	fimbrial family protein	101	19	0	5	centroid_1241	16.8287825	4.09081E-05	centroid_1241	0.00018123
centroid_13580	type VII secretion system (T7SS), usher family protein	101	19	0	5	centroid_1358	16.8287825	4.09081E-05	centroid_1358	0.00018123
centroid_13867	type VII secretion system (T7SS), usher family protein	101	19	0	5	centroid_1386	16.8287825	4.09081E-05	centroid_1386	0.00018123
centroid_14011	class II Aldolase and Adducin N-terminal domain protein	101	19	0	5	centroid_1401	16.8287825	4.09081E-05	centroid_1401	0.00018123
centroid_14613	conserved hypothetical protein	101	19	0	5	centroid_1461	16.8287825	4.09081E-05	centroid_1461	0.00018123
centroid_15451	conserved hypothetical protein	101	19	0	5	centroid_1545	16.8287825	4.09081E-05	centroid_1545	0.00018123
centroid_1582	conserved hypothetical protein	101	19	0	5	centroid_1582	16.8287825	4.09081E-05	centroid_1582	0.00018123
centroid_1666	conserved hypothetical protein	101	19	0	5	centroid_1666	16.8287825	4.09081E-05	centroid_1666	0.00018123
centroid_16905	major MR/P fimbria domain protein	101	19	0	5	centroid_1690	16.8287825	4.09081E-05	centroid_1690	0.00018123
centroid_17640	conserved hypothetical protein	101	19	0	5	centroid_1764	16.8287825	4.09081E-05	centroid_1764	0.00018123
centroid_1885	conserved hypothetical protein	101	19	0	5	centroid_1885	16.8287825	4.09081E-05	centroid_1885	0.00018123
centroid_2634	putative 4-phosphopantetheinyl transferase EntD	101	19	0	5	centroid_2634	16.8287825	4.09081E-05	centroid_2634	0.00018123
centroid_2767	conserved hypothetical protein	101	19	0	5	centroid_2767	16.8287825	4.09081E-05	centroid_2767	0.00018123
centroid_3536	conserved hypothetical protein	101	19	0	5	centroid_3536	16.8287825	4.09081E-05	centroid_3536	0.00018123
centroid_3537	2,3-diketo-L-gulonate reductase	101	19	0	5	centroid_3537	16.8287825	4.09081E-05	centroid_3537	0.00018123
centroid_3538	HTH-type transcriptional regulator YiaJ	101	19	0	5	centroid_3538	16.8287825	4.09081E-05	centroid_3538	0.00018123
centroid_3815	TRAP transporter solute receptor, DctP family protein	101	19	0	5	centroid_3815	16.8287825	4.09081E-05	centroid_3815	0.00018123
centroid_3816	3-keto-L-gulonate-6-phosphate decarboxylase SgbH	101	19	0	5	centroid_3816	16.8287825	4.09081E-05	centroid_3816	0.00018123
centroid_3817	xylose isomerase-like TIM barrel family protein	101	19	0	5	centroid_3817	16.8287825	4.09081E-05	centroid_3817	0.00018123
centroid_4604	inner membrane protein YgiZ	101	19	0	5	centroid_4604	16.8287825	4.09081E-05	centroid_4604	0.00018123
centroid_5298	xylose isomerase-like TIM barrel family protein	101	19	0	5	centroid_5298	16.8287825	4.09081E-05	centroid_5298	0.00018123
centroid_5299	xylose isomerase-like TIM barrel family protein	101	19	0	5	centroid_5299	16.8287825	4.09081E-05	centroid_5299	0.00018123
centroid_6197	maltose acetyltransferase family protein	101	19	0	5	centroid_6197	16.8287825	4.09081E-05	centroid_6197	0.00018123
centroid_6935	hypothetical protein	101	19	0	5	centroid_6935	16.8287825	4.09081E-05	centroid_6935	0.00018123

centroid_7184	papC N-terminal domain protein	101	19	0	5	centroid_7184	16.8287825	4.09081E-05	centroid_7184	0.00018123
centroid_7185	type VII secretion system (T7SS), usher family protein	101	19	0	5	centroid_7185	16.8287825	4.09081E-05	centroid_7185	0.00018123
centroid_7218	galactoside O-acetyltransferase	101	19	0	5	centroid_7218	16.8287825	4.09081E-05	centroid_7218	0.00018123
centroid_77	conserved hypothetical protein	101	19	0	5	centroid_77	16.8287825	4.09081E-05	centroid_77	0.00018123
centroid_845	conserved hypothetical protein	101	19	0	5	centroid_845	16.8287825	4.09081E-05	centroid_845	0.00018123
centroid_911	HTH-type transcriptional regulator FrlR	101	19	0	5	centroid_911	16.8287825	4.09081E-05	centroid_911	0.00018123
centroid_912	pfkB carbohydrate kinase family protein	101	19	0	5	centroid_912	16.8287825	4.09081E-05	centroid_912	0.00018123
centroid_913	xylose isomerase-like TIM barrel family protein	101	19	0	5	centroid_913	16.8287825	4.09081E-05	centroid_913	0.00018123
centroid_914	SIS domain protein	101	19	0	5	centroid_914	16.8287825	4.09081E-05	centroid_914	0.00018123
centroid_915	amino acid permease family protein	101	19	0	5	centroid_915	16.8287825	4.09081E-05	centroid_915	0.00018123
centroid_9912	type VII secretion system (T7SS), usher family protein	101	19	0	5	centroid_9912	16.8287825	4.09081E-05	centroid_9912	0.00018123
centroid_9913	type VII secretion system (T7SS), usher family protein	101	19	0	5	centroid_9913	16.8287825	4.09081E-05	centroid_9913	0.00018123
centroid_9962	gamma-glutamyltranspeptidase domain protein	101	19	0	5	centroid_9962	16.8287825	4.09081E-05	centroid_9962	0.00018123
centroid_10869	type II secretion system protein H	100	1	1	23	centroid_1086	106.410035	5.99534E-25	centroid_1086	7.533E-23
centroid_12074	type II secretion system protein H	100	1	1	23	centroid_1207	106.410035	5.99534E-25	centroid_1207	7.533E-23
centroid_17465	type II secretion system (T2SS), F family protein	100	1	1	23	centroid_1746	106.410035	5.99534E-25	centroid_1746	7.533E-23
centroid_17466	type II secretion system (T2SS), F family protein	100	1	1	23	centroid_1746	106.410035	5.99534E-25	centroid_1746	7.533E-23
centroid_3083	putative bifunctional chitinase/lysozyme	100	1	1	23	centroid_3083	106.410035	5.99534E-25	centroid_3083	7.533E-23
centroid_3089	type II secretion system (T2SS), K family protein	100	1	1	23	centroid_3089	106.410035	5.99534E-25	centroid_3089	7.533E-23
centroid_3090	repilin-type N-terminal cleavage/methylation domain prote	100	1	1	23	centroid_3090	106.410035	5.99534E-25	centroid_3090	7.533E-23
centroid_3091	type II secretion system protein I	100	1	1	23	centroid_3091	106.410035	5.99534E-25	centroid_3091	7.533E-23
centroid_3092	type II secretion system protein H	100	1	1	23	centroid_3092	106.410035	5.99534E-25	centroid_3092	7.533E-23
centroid_3093	type II secretion system protein G	100	1	1	23	centroid_3093	106.410035	5.99534E-25	centroid_3093	7.533E-23
centroid_3094	type II secretion system protein F	100	1	1	23	centroid_3094	106.410035	5.99534E-25	centroid_3094	7.533E-23
centroid_3095	type II secretion system protein E	100	1	1	23	centroid_3095	106.410035	5.99534E-25	centroid_3095	7.533E-23
centroid_4742	repilin-type N-terminal cleavage/methylation domain prote	100	1	1	23	centroid_4742	106.410035	5.99534E-25	centroid_4742	7.533E-23
centroid_7722	type II secretion system protein F	100	1	1	23	centroid_7722	106.410035	5.99534E-25	centroid_7722	7.533E-23
centroid_7723	putative type II secretion system F domain protein	100	1	1	23	centroid_7723	106.410035	5.99534E-25	centroid_7723	7.533E-23
centroid_8069	type II secretion system protein D	100	1	1	23	centroid_8069	106.410035	5.99534E-25	centroid_8069	7.533E-23
centroid_8070	type II secretion system protein L	100	1	1	23	centroid_8070	106.410035	5.99534E-25	centroid_8070	7.533E-23
centroid_8580	carbohydrate binding domain protein	100	1	1	23	centroid_8580	106.410035	5.99534E-25	centroid_8580	7.533E-23
centroid_9733	conserved hypothetical protein	100	2	1	22	centroid_9733	100.241727	1.34888E-23	centroid_9733	3.6834E-21
centroid_9734	hypothetical protein	100	2	1	22	centroid_9734	100.241727	1.34888E-23	centroid_9734	3.6834E-21
centroid_5432	conserved hypothetical protein	100	6	1	18	centroid_5432	76.7649415	1.92566E-18	centroid_5432	1.01E-15
centroid_11950	FHIPEP family protein	100	9	1	15	centroid_1195	60.3380689	7.98873E-15	centroid_1195	2.1308E-12
centroid_17686	putative domain protein	100	10	1	14	centroid_1768	55.0760154	1.15957E-13	centroid_1768	2.1997E-11
centroid_11278	aldo/keto reductase family protein	100	12	1	12	centroid_1127	44.8649328	2.11105E-11	centroid_1127	1.8E-09
centroid_11408	zinc-binding dehydrogenase family protein	100	12	1	12	centroid_1140	44.8649328	2.11105E-11	centroid_1140	1.8E-09
centroid_13575	pfkB carbohydrate kinase family protein	100	12	1	12	centroid_1357	44.8649328	2.11105E-11	centroid_1357	1.8E-09
centroid_3166	major Facilitator Superfamily protein	100	12	1	12	centroid_3166	44.8649328	2.11105E-11	centroid_3166	1.8E-09
centroid_3167	alcohol dehydrogenase GroES-like domain protein	100	12	1	12	centroid_3167	44.8649328	2.11105E-11	centroid_3167	1.8E-09
centroid_3844	aldo/keto reductase family protein	100	12	1	12	centroid_3844	44.8649328	2.11105E-11	centroid_3844	1.8E-09
centroid_3845	deoR-like helix-turn-helix domain protein	100	12	1	12	centroid_3845	44.8649328	2.11105E-11	centroid_3845	1.8E-09
centroid_3846	inner membrane metabolite transport protein YdjE	100	12	1	12	centroid_3846	44.8649328	2.11105E-11	centroid_3846	1.8E-09
centroid_4236	major Facilitator Superfamily protein	100	12	1	12	centroid_4236	44.8649328	2.11105E-11	centroid_4236	1.8E-09
centroid_5297	pfkB carbohydrate kinase family protein	100	12	1	12	centroid_5297	44.8649328	2.11105E-11	centroid_5297	1.8E-09
centroid_5512	sugar (and other) transporter family protein	100	12	1	12	centroid_5512	44.8649328	2.11105E-11	centroid_5512	1.8E-09
centroid_7566	zinc-binding dehydrogenase family protein	100	12	1	12	centroid_7566	44.8649328	2.11105E-11	centroid_7566	1.8E-09
centroid_7567	alcohol dehydrogenase GroES-like domain protein	100	12	1	12	centroid_7567	44.8649328	2.11105E-11	centroid_7567	1.8E-09
centroid_9282	sugar (and other) transporter family protein	100	12	1	12	centroid_9282	44.8649328	2.11105E-11	centroid_9282	1.8E-09
centroid_9283	sugar (and other) transporter family protein	100	12	1	12	centroid_9283	44.8649328	2.11105E-11	centroid_9283	1.8E-09
centroid_13866	type VII secretion system (T7SS), usher family protein	100	14	1	10	centroid_1386	35.0714451	3.17827E-09	centroid_1386	1.0817E-07
centroid_7792	thanolamine utilization - propanediol utilization family prote	100	14	1	10	centroid_7792	35.0714451	3.17827E-09	centroid_7792	1.0817E-07
centroid_8736	thanolamine utilization - propanediol utilization family prote	100	14	1	10	centroid_8736	35.0714451	3.17827E-09	centroid_8736	1.0817E-07
centroid_17241	conserved hypothetical protein	100	17	1	7	centroid_1724	21.212171	4.11145E-06	centroid_1724	3.0346E-05
centroid_10278	type VII secretion system (T7SS), usher family protein	100	18	1	6	centroid_1027	16.8487798	4.04793E-05	centroid_1027	0.00017482
centroid_10331	type VII secretion system (T7SS), usher family protein	100	18	1	6	centroid_1033	16.8487798	4.04793E-05	centroid_1033	0.00017482
centroid_11121	hypothetical protein	100	18	1	6	centroid_1112	16.8487798	4.04793E-05	centroid_1112	0.00017482
centroid_11884	papC N-terminal domain protein	100	18	1	6	centroid_1188	16.8487798	4.04793E-05	centroid_1188	0.00017482
centroid_13001	tonB-dependent siderophore receptor family protein	100	18	1	6	centroid_1300	16.8487798	4.04793E-05	centroid_1300	0.00017482
centroid_13002	tonB-dependent Receptor Plug domain protein	100	18	1	6	centroid_1300	16.8487798	4.04793E-05	centroid_1300	0.00017482
centroid_1513	putative ybl100	100	18	1	6	centroid_1513	16.8487798	4.04793E-05	centroid_1513	0.00017482
centroid_240	ferrichrome-iron receptor	100	18	1	6	centroid_240	16.8487798	4.04793E-05	centroid_240	0.00017482
centroid_9275	tonB-dependent Receptor Plug domain protein	100	18	1	6	centroid_9275	16.8487798	4.04793E-05	centroid_9275	0.00017482
centroid_9957	ferrichrome-iron receptor	100	18	1	6	centroid_9957	16.8487798	4.04793E-05	centroid_9957	0.00017482
centroid_13907	conserved hypothetical protein	99	0	2	24	centroid_1390	107.227915	3.96808E-25	centroid_1390	1.0096E-23
centroid_1481	putative lipoprotein	99	0	2	24	centroid_1481	107.227915	3.96808E-25	centroid_1481	1.0096E-23
centroid_1482	conserved hypothetical protein	99	0	2	24	centroid_1482	107.227915	3.96808E-25	centroid_1482	1.0096E-23
centroid_8471	conserved hypothetical protein	99	0	2	24	centroid_8471	107.227915	3.96808E-25	centroid_8471	1.0096E-23
centroid_17801	putative membrane protein	99	3	2	21	centroid_1780	88.850023	4.25909E-21	centroid_1780	1.3531E-18
centroid_1781	inner membrane protein YmfA	99	3	2	21	centroid_1781	88.850023	4.25909E-21	centroid_1781	1.3531E-18
centroid_15706	hypothetical protein	99	11	2	13	centroid_1570	45.1922145	1.78614E-11	centroid_1570	1.4126E-09
centroid_3381	alpha amylase, catalytic domain protein	99	11	2	13	centroid_3381	45.1922145	1.78614E-11	centroid_3381	1.4126E-09
centroid_12827	pfkB carbohydrate kinase family protein	99	12	2	12	centroid_1282	40.2620038	2.22087E-10	centroid_1282	1.1355E-08
centroid_3841	alcohol dehydrogenase GroES-like domain protein	99	12	2	12	centroid_3841	40.2620038	2.22087E-10	centroid_3841	1.1355E-08
centroid_3842	ketose-bisphosphate aldolase family protein	99	12	2	12	centroid_3842	40.2620038	2.22087E-10	centroid_3842	1.1355E-08
centroid_4320	zinc-binding dehydrogenase family protein	99	12	2	12	centroid_4320	40.2620038	2.22087E-10	centroid_4320	1.1355E-08
centroid_4319	alcohol dehydrogenase GroES-like domain protein	99	13	2	11	centroid_4319	35.4527729	2.6131E-09	centroid_4319	8.4124E-08
centroid_4773	conserved hypothetical protein	99	14	2	10	centroid_4773	30.769472	2.90573E-08	centroid_4773	5.7671E-07
centroid_6916	conserved hypothetical protein	99	15	2	9	centroid_6916	26.2198198	3.04679E-07	centroid_6916	3.669E-06
centroid_2768	putative type-1 fimbrial protein, A chain	99	16	2	8	centroid_2768	21.8156411	3.00143E-06	centroid_2768	2.1696E-05
centroid_7818	conserved hypothetical protein	99	16	2	8	centroid_7818	21.8156411	3.00143E-06	centroid_7818	2.1696E-05
centroid_7097	conserved hypothetical protein	99	17	2	7	centroid_7097	17.5750996	2.76181E-05	centroid_7097	0.00011925
centroid_3096	type II secretion system protein D	98	1	3	23	centroid_3096	95.953751	1.17599E-22	centroid_3096	8.0059E-21
centroid_8068	type II secretion system D domain protein	98	1	3	23	centroid_8068	95.953751	1.17599E-22	centroid_8068	8.0059E-21
centroid_2542	helix-turn-helix domain protein	98	4	3	20	centroid_2542	78.1452281	9.57366E-19	centroid_2542	2.3514E-16
centroid_3555	integrase core domain protein	98	4	3	20	centroid_3555	78.1452281	9.57366E-19	centroid_3555	2.3514E-16
centroid_4665	conserved hypothetical protein	98	4	3	20	centroid_4665	78.1452281	9.57366E-19	centroid_4665	2.3514E-16
centroid_4814	conserved hypothetical protein	98	4	3	20	centroid_4814	78.1452281	9.57366E-19	centroid_4814	2.3514E-16
centroid_16928	yiaA/B two helix domain protein	98	5	3	19	centroid_1692	72.468721	1.697E-17	centroid_1692	4.2197E-15
centroid_17238	alpha amylase, catalytic domain protein	98	10	3	14	centroid_1723	45.9817294	1.19361E-11	centroid_1723	8.3467E-10
centroid_10622	putative transposase DNA-binding domain protein	98	11	3	13	centroid_1062	41.066727	1.4712E-10	centroid_1062	6.8359E-09
centroid_5520	integrase core domain protein	98	11	3	13	centroid_5520	41.066727	1.4712E-10	centroid_5520	6.8359E-09
centroid_5511	conserved hypothetical protein	98	12	3	12	centroid_5511	36.2850738	1.70464E-09	centroid_5511	5.1127E-08
centroid_3097	type II secretion system protein C	97	1	4	23	centroid_3097	91.3067192	1.23042E-21	centroid_3097	5.3518E-20
centroid_17796	outer membrane autotransporter barrel domain protein	97	4	4	20	centroid_1779	73.7175449	9.01342E-18	centroid_1779	1.3582E-15
centroid_4666	outer membrane autotransporter barrel domain protein	97	4	4	20	centroid_4666	73.7175449	9.01342E-18	centroid_4666	1.3582E-15
centroid_5353	integrase core domain protein	97	4	4	20	centroid_5353	73.7175449	9.01342E-18	centroid_5353	1.3582E-15
centroid_8894	outer membrane autotransporter barrel domain protein	97	4	4	20	centroid_8894	73.7175449	9.01342E-18	centroid_8894	1.3582E-15
centroid_14132	integrase core domain protein	97	13	4	11	centroid_1413	28.3545933	1.01007E-07	centroid_1413	1.1754E-06
centroid_5513	integrase core domain protein	97	14	4	10	centroid_5513	24.0600036	9.33799E-07	centroid_5513	6.8879E-06
centroid_15977	transposase, IS605 OrfB family	97	15	4	9	centroid_1597	19.9488294	7.95426E-06	centroid_1597	3.7083E-05
centroid_10180	integrase core domain protein	96	11	5	13	centroid_1018	34.2186009	4.92559E-09	centroid_1018	8.59E-08
centroid_14811	integrase core domain protein	96	11	5	13	centroid_1481	34.2186009	4.92559E-09	centroid_1481	8.59E-08
centroid_11719	integrase core domain protein	96	13	5	11	centroid_1171	25.4914614	4.44345E-07	centroid_1171	3.3566E-06
centroid_13637	integrase core domain protein	96	13	5	11	centroid_1363	25.4914614	4.44345E-07	centroid_1363	3.3566E-06
centroid_4246	integrase core domain protein	96	13	5	11	centroid_4246	25.4914614	4.44345E-07	centroid_4246	3.3566E-06

centroid_10137	caudovirales tail fibre assembly family protein	96	14	5	10	centroid_1013	21.4007729	3.72621E-06	centroid_1013	1.8313E-05
centroid_7172	istB-like ATP binding family protein	96	15	5	9	centroid_7172	17.514495	2.85126E-05	centroid_7172	9.1433E-05
centroid_14088	integrase core domain protein	95	11	6	13	centroid_1408	31.3487889	2.15593E-08	centroid_1408	2.4621E-07
centroid_12444	uvate-dependent sugar phosphotransferase system, EIIA	95	15	6	9	centroid_1244	15.4236127	8.59081E-05	centroid_1244	0.00020111
centroid_2249	corbate-specific phosphotransferase enzyme IIA compon	95	15	6	9	centroid_2249	15.4236127	8.59081E-05	centroid_2249	0.00020111
centroid_4739	WGR domain protein	94	0	7	24	centroid_4739	85.1457799	2.77159E-20	centroid_4739	8.1685E-20
centroid_526	conserved hypothetical protein	94	0	7	24	centroid_526	85.1457799	2.77159E-20	centroid_526	8.1685E-20
centroid_15925	reverse transcriptase family protein	94	13	7	11	centroid_1592	20.7577289	5.21208E-06	centroid_1592	1.9423E-05
centroid_4181	reverse transcriptase family protein	94	13	7	11	centroid_4181	20.7577289	5.21208E-06	centroid_4181	1.9423E-05
centroid_7880	reverse transcriptase family protein	94	13	7	11	centroid_7880	20.7577289	5.21208E-06	centroid_7880	1.9423E-05
centroid_7881	reverse transcriptase family protein	94	13	7	11	centroid_7881	20.7577289	5.21208E-06	centroid_7881	1.9423E-05
centroid_7943	group II intron, maturase-specific domain protein	94	13	7	11	centroid_7943	20.7577289	5.21208E-06	centroid_7943	1.9423E-05
centroid_8104	reverse transcriptase family protein	94	13	7	11	centroid_8104	20.7577289	5.21208E-06	centroid_8104	1.9423E-05
centroid_8276	reverse transcriptase family protein	94	13	7	11	centroid_8276	20.7577289	5.21208E-06	centroid_8276	1.9423E-05
centroid_18647	istB-like ATP binding family protein	93	12	8	12	centroid_1864	22.5131191	2.08713E-06	centroid_1864	8.2732E-06
centroid_5141	integrase core domain protein	93	13	8	11	centroid_5141	18.7833333	1.46441E-05	centroid_5141	4.1053E-05
centroid_2894	oly-beta-1,6-N-acetyl-D-glucosamine N-deacetylase Pga	93	14	8	10	centroid_2894	15.2823468	9.25777E-05	centroid_2894	0.00018474
centroid_13091	mRNA interferase MqsR	92	4	9	20	centroid_1309	56.1765578	6.62467E-14	centroid_1309	1.0527E-12
centroid_2748	mRNA interferase MqsR	92	4	9	20	centroid_2748	56.1765578	6.62467E-14	centroid_2748	1.0527E-12
centroid_9316	protein PhnP	92	5	9	19	centroid_9316	51.0975528	8.78878E-13	centroid_9316	1.4167E-11
centroid_10926	gyrI-like small molecule binding domain protein	92	6	9	18	centroid_1092	46.1898917	1.07329E-11	centroid_1092	1.5804E-10
centroid_17190	bacterial dnaA family protein	92	12	9	12	centroid_1719	20.5756426	5.73208E-06	centroid_1719	1.7292E-05
centroid_9797	phage DNA packaging Nu1 family protein	92	13	9	11	centroid_9797	17.018708	3.70133E-05	centroid_9797	8.1118E-05
centroid_5142	integrase core domain protein	91	11	10	13	centroid_5142	22.4451248	2.16234E-06	centroid_5142	6.9087E-06
centroid_15969	CRISPR-associated endonuclease/helicase Cas3	90	2	11	22	centroid_1596	61.0271667	5.62926E-15	centroid_1596	2.5432E-14
centroid_7749	hypothetical protein	90	9	11	15	centroid_7749	28.2987483	1.03963E-07	centroid_7749	4.452E-07
centroid_6945	transposase DDE domain protein	90	10	11	14	centroid_6945	24.3947246	7.84833E-07	centroid_6945	2.6024E-06
centroid_8205	type VII secretion system (T7SS), usher family protein	90	10	11	14	centroid_8205	24.3947246	7.84833E-07	centroid_8205	2.6024E-06
centroid_9929	transposase family protein	90	10	11	14	centroid_9929	24.3947246	7.84833E-07	centroid_9929	2.6024E-06
centroid_15610	transposase family protein	90	11	11	13	centroid_1561	20.7032707	5.36243E-06	centroid_1561	1.3584E-05
centroid_6531	conserved hypothetical protein	90	11	11	13	centroid_6531	20.7032707	5.36243E-06	centroid_6531	1.3584E-05
centroid_10582	SPR-associated protein Cas6/Cse3/CasE, subtype I-E/E(	89	1	12	23	centroid_1058	63.6943438	1.45301E-15	centroid_1058	2.3459E-15
centroid_15998	SPR-associated endonuclease/helicase Cas3 domain pro	89	2	12	22	centroid_1599	58.376325	2.1648E-14	centroid_1599	7.0567E-14
centroid_2331	CRISPR-associated endonuclease Cas1	89	3	12	21	centroid_2331	53.2436099	2.94644E-13	centroid_2331	1.4096E-12
centroid_4611	SPR-associated endoribonuclease Cas2, subtype I-E/EC	89	3	12	21	centroid_4611	53.2436099	2.94644E-13	centroid_4611	1.4096E-12
centroid_9502	SPR-associated endoribonuclease Cas2, subtype I-E/EC	89	3	12	21	centroid_9502	53.2436099	2.94644E-13	centroid_9502	1.4096E-12
centroid_13465	conserved hypothetical protein	89	5	12	19	centroid_1346	43.5368392	4.16053E-11	centroid_1346	2.4884E-10
centroid_9932	rhs core with extension domain protein	89	5	12	19	centroid_9932	43.5368392	4.16053E-11	centroid_9932	2.4884E-10
centroid_5521	POTRA domain, ShlB-type family protein	89	10	12	14	centroid_5521	22.6591619	1.93436E-06	centroid_5521	5.1191E-06
centroid_17493	hypothetical protein	88	1	13	23	centroid_1749	61.106261	5.40759E-15	centroid_1749	6.4275E-15
centroid_17627	fimbrial subunit ElfA	88	2	13	22	centroid_1762	55.8773418	7.71365E-14	centroid_1762	1.8602E-13
centroid_9313	CRISPR-associated endonuclease/helicase Cas3	88	2	13	22	centroid_9313	55.8773418	7.71365E-14	centroid_9313	1.8602E-13
centroid_11130	conserved hypothetical protein	88	5	13	19	centroid_1113	41.335224	1.28237E-10	centroid_1113	5.8182E-10
centroid_3520	putative predicted protein	88	6	13	18	centroid_3520	36.8740998	1.26009E-09	centroid_3520	5.4655E-09
centroid_3521	conserved hypothetical protein	88	6	13	18	centroid_3521	36.8740998	1.26009E-09	centroid_3521	5.4655E-09
centroid_5163	putative domain protein	88	6	13	18	centroid_5163	36.8740998	1.26009E-09	centroid_5163	5.4655E-09
centroid_10619	conserved hypothetical protein	88	7	13	17	centroid_1061	32.6107755	1.12589E-08	centroid_1061	4.3545E-08
centroid_11183	transposase DDE domain protein	88	9	13	15	centroid_1118	24.6966593	6.71004E-07	centroid_1118	1.8078E-06
centroid_7910	hypothetical protein	88	9	13	15	centroid_7910	24.6966593	6.71004E-07	centroid_7910	1.8078E-06
centroid_3088	type II secretion system protein L	87	1	14	23	centroid_3088	58.6582135	1.87583E-14	centroid_3088	1.6805E-14
centroid_12408	RISPR-associated protein Cas5/CasD, subtype I-E/ECO	87	2	14	22	centroid_1240	53.5175749	2.5629E-13	centroid_1240	4.6823E-13
centroid_13690	CT1975-like family protein	87	2	14	22	centroid_1369	53.5175749	2.5629E-13	centroid_1369	4.6823E-13
centroid_16204	CRISPR type I-E/ECOLI-associated protein CasA/Cse1	87	2	14	22	centroid_1620	53.5175749	2.5629E-13	centroid_1620	4.6823E-13
centroid_2335	CRISPR type I-E/ECOLI-associated protein CasB/Cse2	87	2	14	22	centroid_2335	53.5175749	2.5629E-13	centroid_2335	4.6823E-13
centroid_5348	CT1975-like family protein	87	2	14	22	centroid_5348	53.5175749	2.5629E-13	centroid_5348	4.6823E-13
centroid_5358	SPR-associated protein Cas7/Cse4/CasC, subtype I-E/E(	87	2	14	22	centroid_5358	53.5175749	2.5629E-13	centroid_5358	4.6823E-13
centroid_4109	conserved hypothetical protein	87	8	14	16	centroid_4109	26.8207228	2.23231E-07	centroid_4109	5.9264E-07
centroid_18621	rhs element Vgr family protein	87	9	14	15	centroid_1862	23.0900898	1.54585E-06	centroid_1862	3.4235E-06
centroid_7904	surface antigen family protein	87	9	14	15	centroid_7904	23.0900898	1.54585E-06	centroid_7904	3.4235E-06
centroid_11415	conserved hypothetical protein	87	11	14	13	centroid_1141	16.2987755	5.40989E-05	centroid_1141	7.9458E-05
centroid_8132	conserved hypothetical protein	87	11	14	13	centroid_8132	16.2987755	5.40989E-05	centroid_8132	7.9458E-05
centroid_13505	RHS repeat-associated core domain protein	86	2	15	22	centroid_1350	51.2857491	7.98532E-13	centroid_1350	1.1303E-12
centroid_17647	CRISPR type I-E/ECOLI-associated protein CasA/Cse1	86	2	15	22	centroid_1764	51.2857491	7.98532E-13	centroid_1764	1.1303E-12
centroid_4613	CRISPR type I-E/ECOLI-associated protein CasA/Cse1	86	2	15	22	centroid_4613	51.2857491	7.98532E-13	centroid_4613	1.1303E-12
centroid_6137	CRISPR type I-E/ECOLI-associated protein CasA/Cse1	86	2	15	22	centroid_6137	51.2857491	7.98532E-13	centroid_6137	1.1303E-12
centroid_5556	hypothetical protein	86	7	15	17	centroid_5556	29.0367884	7.10168E-08	centroid_5556	1.7823E-07
centroid_12323	hypothetical protein	86	8	15	16	centroid_1232	25.2076695	5.14771E-07	centroid_1232	1.129E-06
centroid_13670	family 4 glycosyl hydrolase C-terminal domain protein	86	8	15	16	centroid_1367	25.2076695	5.14771E-07	centroid_1367	1.129E-06
centroid_4110	conserved hypothetical protein	86	8	15	16	centroid_4110	25.2076695	5.14771E-07	centroid_4110	1.129E-06
centroid_4439	family 4 glycosyl hydrolase C-terminal domain protein	86	8	15	16	centroid_4439	25.2076695	5.14771E-07	centroid_4439	1.129E-06
centroid_4977	conserved hypothetical protein	86	8	15	16	centroid_4977	25.2076695	5.14771E-07	centroid_4977	1.129E-06
centroid_12675	RHS repeat-associated core domain protein	86	10	15	14	centroid_1267	18.2093148	1.97909E-05	centroid_1267	3.06E-05
centroid_6733	transposase family protein	86	10	15	14	centroid_6733	18.2093148	1.97909E-05	centroid_6733	3.06E-05
centroid_6925	conserved hypothetical protein	86	10	15	14	centroid_6925	18.2093148	1.97909E-05	centroid_6925	3.06E-05
centroid_4612	RISPR-associated protein Cas5/CasD, subtype I-E/ECO	85	2	16	22	centroid_4612	49.1717768	2.34501E-12	centroid_4612	2.6266E-12
centroid_2084	6-phospho-alpha-glucosidase	85	8	16	16	centroid_2084	23.6998132	1.12593E-06	centroid_2084	2.0798E-06
centroid_4438	4 glycosyl hydrolase family protein	85	8	16	16	centroid_4438	23.6998132	1.12593E-06	centroid_4438	2.0798E-06
centroid_8296	hypothetical protein	85	9	16	15	centroid_8296	20.2039788	6.9608E-06	centroid_8296	1.1059E-05
centroid_15685	transposase family protein	85	10	16	14	centroid_1568	16.9369219	3.86426E-05	centroid_1568	5.1902E-05
centroid_11498	conserved hypothetical protein	84	8	17	16	centroid_1149	22.2877289	2.34702E-06	centroid_1149	3.7156E-06
centroid_9934	integrase core domain protein	84	9	17	15	centroid_9934	18.904333	1.3744E-05	centroid_9934	1.8983E-05
centroid_16849	hypothetical protein	83	0	18	24	centroid_1684	55.0731073	1.16129E-13	centroid_1684	1.0987E-14
centroid_2332	SPR-associated protein Cas6/Cse3/CasE, subtype I-E/E(	83	1	18	23	centroid_2332	50.0615376	1.48999E-12	centroid_2332	5.3209E-13
centroid_12681	RISPR-associated protein Cas5/CasD, subtype I-E/ECO	83	2	18	22	centroid_1268	45.2620888	1.72353E-11	centroid_1268	1.281E-11
centroid_9314	SPR-associated protein Cas6/Cse3/CasE, subtype I-E/E(	83	2	18	22	centroid_9314	45.2620888	1.72353E-11	centroid_9314	1.281E-11
centroid_4648	putative rhsC protein in rhs element	83	7	18	17	centroid_4648	24.4660906	7.56292E-07	centroid_4648	1.1467E-06
centroid_13709	POTRA domain, ShlB-type family protein	83	9	18	15	centroid_1370	17.6889335	2.60136E-05	centroid_1370	3.1705E-05
centroid_7156	putative transposase	82	5	19	19	centroid_7156	30.5943179	3.18024E-08	centroid_7156	4.2989E-08
centroid_10218	conserved hypothetical protein	82	6	19	18	centroid_1021	26.7451827	2.3213E-07	centroid_1021	3.2021E-07
centroid_9921	hypothetical protein	82	6	19	18	centroid_9921	26.7451827	2.3213E-07	centroid_9921	3.2021E-07
centroid_7022	putative transposase	82	7	19	17	centroid_7022	23.11857	1.52313E-06	centroid_7022	2.0157E-06
centroid_15206	hypothetical protein	82	8	19	16	centroid_1520	19.718592	8.97242E-06	centroid_1520	1.0923E-05
centroid_2333	RISPR-associated protein Cas5/CasD, subtype I-E/ECO	81	2	20	22	centroid_2333	41.7263149	1.04986E-10	centroid_2333	5.5476E-11
centroid_16544	rhs element Vgr family protein	81	5	20	19	centroid_1654	29.1317632	6.76192E-08	centroid_1654	7.9315E-08
centroid_12706	conserved hypothetical protein	81	6	20	18	centroid_1270	25.3767192	4.71575E-07	centroid_1270	5.6971E-07
centroid_10649	conserved hypothetical protein	81	7	20	17	centroid_1064	21.8473639	2.95222E-06	centroid_1064	3.4568E-06
centroid_5188	transposase DDE domain protein	80	0	21	24	centroid_5188	49.4233912	2.06271E-12	centroid_5188	1.1722E-13
centroid_15252	inner membrane YibH domain protein	80	5	21	19	centroid_1525	27.7442525	1.38459E-07	centroid_1525	1.4285E-07
centroid_12835	tn3 transposase DDE domain protein	79	8	22	16	centroid_1283	16.4038533	5.11811E-05	centroid_1283	4.6163E-05
centroid_580	biquinone/plastoquinone (complex I), various chains fami	78	0	23	24	centroid_580	46.0576857	1.14822E-11	centroid_580	5.0087E-13
centroid_14160	conserved domain protein	78	3	23	21	centroid_1416	32.8380965	1.00163E-08	centroid_1416	5.5511E-09
centroid_4158	phage integrase family protein	78	6	23	18	centroid_4158	21.6873478	3.20901E-06	centroid_4158	2.8036E-06
centroid_4230	conserved hypothetical protein	78	8	23	16	centroid_4230	15.4211317	8.6021E-05	centroid_4230	0.00011021
centroid_4848	tn3 transposase DDE domain protein	78	8	23	16	centroid_4848	15.4211317	8.6021E-05	centroid_4848	0.00011021
centroid_10333	tive pilus biosynthesis transmembrane anchor domain pro	77	3	24	21	centroid_1033	31.482144	2.01283E-08	centroid_1033	1.0046E-08
centroid_10334	tive pilus biosynthesis transmembrane anchor domain pro	77	3	24	21	centroid_1033	31.482144	2.01283E-08	centroid_1033	1.0046E-08
centroid_10335	hypothetical protein	77	3	24	21	centroid_1033	31.482144	2.01283E-08	centroid_1033	1.0046E-08

centroid_14026	type II/IV secretion system family protein	77	3	24	21	centroid_1402	31.482144	2.01283E-08	centroid_1402	1.0046E-08
centroid_4850	type IV leader peptidase family protein	77	3	24	21	centroid_485	31.482144	2.01283E-08	centroid_485	1.0046E-08
centroid_4851	putative pilus biosythesis protein	77	3	24	21	centroid_485	31.482144	2.01283E-08	centroid_485	1.0046E-08
centroid_4852	type II secretion system (T2SS), F family protein	77	3	24	21	centroid_485	31.482144	2.01283E-08	centroid_485	1.0046E-08
centroid_4853	type II/IV secretion system family protein	77	3	24	21	centroid_485	31.482144	2.01283E-08	centroid_485	1.0046E-08
centroid_4854	putative lngG	77	3	24	21	centroid_485	31.482144	2.01283E-08	centroid_485	1.0046E-08
centroid_4856	putative pilus biosynthesis transmembrane anchor protei	77	3	24	21	centroid_485	31.482144	2.01283E-08	centroid_485	1.0046E-08
centroid_4858	toxin co-regulated pilus biosynthesis Q family protein	77	3	24	21	centroid_485	31.482144	2.01283E-08	centroid_485	1.0046E-08
centroid_4859	repilin-type N-terminal cleavage/methylation domain prote	77	3	24	21	centroid_485	31.482144	2.01283E-08	centroid_485	1.0046E-08
centroid_4861	putative lngX2	77	3	24	21	centroid_486	31.482144	2.01283E-08	centroid_486	1.0046E-08
centroid_4862	transglycosylase SLT domain protein	77	3	24	21	centroid_486	31.482144	2.01283E-08	centroid_486	1.0046E-08
centroid_4863	bacterial regulatory helix-turn-helix, AraC family protein	77	3	24	21	centroid_486	31.482144	2.01283E-08	centroid_486	1.0046E-08
centroid_4864	dhesin biosynthesis transcription regulatory family protei	77	3	24	21	centroid_486	31.482144	2.01283E-08	centroid_486	1.0046E-08
centroid_8277	bacterial type II and III secretion system family protein	77	3	24	21	centroid_827	31.482144	2.01283E-08	centroid_827	1.0046E-08
centroid_4432	caudovirales tail fibre assembly family protein	77	4	24	20	centroid_443	27.6147732	1.48044E-07	centroid_443	9.6565E-08
centroid_4849	conserved hypothetical protein	77	4	24	20	centroid_484	27.6147732	1.48044E-07	centroid_484	9.6565E-08
centroid_8329	putative iS91 transposase	77	5	24	19	centroid_832	23.9802803	9.73275E-07	centroid_832	7.3366E-07
centroid_14802	bacterial Ig-like domain family protein	77	6	24	18	centroid_1480	20.5800253	5.71897E-06	centroid_1480	4.581E-06
centroid_16797	bacterial Ig-like domain family protein	77	6	24	18	centroid_1679	20.5800253	5.71897E-06	centroid_1679	4.581E-06
centroid_4917	conserved hypothetical protein	77	6	24	18	centroid_491	20.5800253	5.71897E-06	centroid_491	4.581E-06
centroid_4099	conserved hypothetical protein	77	7	24	17	centroid_409	17.4162461	3.00249E-05	centroid_409	4.4073E-05
centroid_4838	conserved hypothetical protein	77	7	24	17	centroid_483	17.4162461	3.00249E-05	centroid_483	4.4073E-05
centroid_4855	putative pilus biosynthesis protein	76	3	25	21	centroid_485	30.1859843	3.92536E-08	centroid_485	1.7832E-08
centroid_4857	bacterial type II and III secretion system family protein	76	3	25	21	centroid_485	30.1859843	3.92536E-08	centroid_485	1.7832E-08
centroid_17721	major Facilitator Superfamily protein	76	5	25	19	centroid_1772	22.8436074	1.75733E-06	centroid_1772	1.2179E-06
centroid_17722	major Facilitator Superfamily protein	76	5	25	19	centroid_1772	22.8436074	1.75733E-06	centroid_1772	1.2179E-06
centroid_3931	deoR-like helix-turn-helix domain protein	76	5	25	19	centroid_393	22.8436074	1.75733E-06	centroid_393	1.2179E-06
centroid_3932	ribokinase	76	5	25	19	centroid_393	22.8436074	1.75733E-06	centroid_393	1.2179E-06
centroid_3934	putative monosaccharide-transporting ATPase	76	5	25	19	centroid_393	22.8436074	1.75733E-06	centroid_393	1.2179E-06
centroid_7843	phage tail tape measure protein, TP901 family, core regior	76	5	25	19	centroid_784	22.8436074	1.75733E-06	centroid_784	1.2179E-06
centroid_18668	putative membrane protein	76	7	25	17	centroid_1866	16.4491395	4.99729E-05	centroid_1866	5.167E-05
centroid_4397	ftsK/SpoIIIE family protein	75	1	26	23	centroid_439	37.0851248	1.13083E-09	centroid_439	1.397E-10
centroid_3933	H+ symporter permease	75	5	26	19	centroid_393	21.759492	3.09057E-06	centroid_393	1.9867E-06
centroid_10044	utative DNA-binding transcriptional regulator domain prote	75	6	26	18	centroid_1004	18.5245991	1.67726E-05	centroid_1004	1.8759E-05
centroid_11968	bacterial Ig-like domain family protein	75	6	26	18	centroid_1196	18.5245991	1.67726E-05	centroid_1196	1.8759E-05
centroid_12473	bacterial Ig-like domain family protein	75	6	26	18	centroid_1247	18.5245991	1.67726E-05	centroid_1247	1.8759E-05
centroid_14668	hypothetical protein	74	1	27	23	centroid_1466	35.755724	2.23674E-09	centroid_1466	2.5549E-10
centroid_15981	H+ symporter family protein	74	1	27	23	centroid_1598	35.755724	2.23674E-09	centroid_1598	2.5549E-10
centroid_16661	toxin YafO, type II toxin-antitoxin system family protein	74	1	27	23	centroid_1666	35.755724	2.23674E-09	centroid_1666	2.5549E-10
centroid_17455	conserved hypothetical protein	74	1	27	23	centroid_1745	35.755724	2.23674E-09	centroid_1745	2.5549E-10
centroid_2822	antitoxin YafN	74	1	27	23	centroid_282	35.755724	2.23674E-09	centroid_282	2.5549E-10
centroid_4699	conserved hypothetical protein	74	1	27	23	centroid_469	35.755724	2.23674E-09	centroid_469	2.5549E-10
centroid_7700	mRNA interferase HigB	74	1	27	23	centroid_770	35.755724	2.23674E-09	centroid_770	2.5549E-10
centroid_2334	SPR-associated protein Cas7/Cse4/CasC, subtype I-E/E(	74	2	27	22	centroid_233	31.6361793	1.85933E-08	centroid_233	4.5397E-09
centroid_5525	conserved hypothetical protein	74	4	27	20	centroid_552	24.1210352	9.04667E-07	centroid_552	4.6366E-07
centroid_9765	aatD, apolipoN-acyltransferase domain protein	74	5	27	19	centroid_976	20.7245822	5.30308E-06	centroid_976	3.188E-06
centroid_18009	bacterial Ig-like domain family protein	74	6	27	18	centroid_1800	17.5696199	2.76978E-05	centroid_1800	2.3044E-05
centroid_10353	orn/Lys/Arg decarboxylase, major domain protein	73	0	28	24	centroid_1035	38.7764089	4.75237E-10	centroid_1035	1.3245E-11
centroid_12500	ornithine decarboxylase, inducible domain protein	73	0	28	24	centroid_1250	38.7764089	4.75237E-10	centroid_1250	1.3245E-11
centroid_1484	conserved hypothetical protein	73	0	28	24	centroid_148	38.7764089	4.75237E-10	centroid_148	1.3245E-11
centroid_16491	orn/Lys/Arg decarboxylase, N-terminal domain protein	73	0	28	24	centroid_1649	38.7764089	4.75237E-10	centroid_1649	1.3245E-11
centroid_11286	D-serine deaminase transcriptional activator	73	1	28	23	centroid_1128	34.4786252	4.30958E-09	centroid_1128	4.595E-10
centroid_1251	dsdX permease	73	1	28	23	centroid_125	34.4786252	4.30958E-09	centroid_125	4.595E-10
centroid_2823	mRNA interferase YafO	73	1	28	23	centroid_282	34.4786252	4.30958E-09	centroid_282	4.595E-10
centroid_6217	lasmic binding and sugar binding domain of LacI family pr	73	2	28	22	centroid_621	30.4270661	3.46657E-08	centroid_621	7.9059E-09
centroid_733	periplasmic binding domain protein	73	2	28	22	centroid_733	30.4270661	3.46657E-08	centroid_733	7.9059E-09
centroid_734	D-allose transporter subunit domain protein	73	2	28	22	centroid_734	30.4270661	3.46657E-08	centroid_734	7.9059E-09
centroid_735	ABC transporter family protein	73	2	28	22	centroid_735	30.4270661	3.46657E-08	centroid_735	7.9059E-09
centroid_9859	hypothetical protein	73	2	28	22	centroid_985	30.4270661	3.46657E-08	centroid_985	7.9059E-09
centroid_732	HTH-type transcriptional regulator RpiR	73	4	28	20	centroid_732	23.0562991	1.57326E-06	centroid_732	7.568E-07
centroid_13568	putative aatD, apolipoprotein N-acyltransferase	73	5	28	19	centroid_1356	19.7358151	8.89192E-06	centroid_1356	7.4927E-06
centroid_14630	ribose 5-phosphate isomerase B	73	5	28	19	centroid_1463	19.7358151	8.89192E-06	centroid_1463	7.4927E-06
centroid_5879	ABC transporter family protein	73	5	28	19	centroid_587	19.7358151	8.89192E-06	centroid_587	7.4927E-06
centroid_6218	heme ABC exporter, ATP-binding protein CcmA	73	5	28	19	centroid_621	19.7358151	8.89192E-06	centroid_621	7.4927E-06
centroid_731	ribose 5-phosphate isomerase B	73	5	28	19	centroid_731	19.7358151	8.89192E-06	centroid_731	7.4927E-06
centroid_736	ain amino acid transport system / permease component	73	5	28	19	centroid_736	19.7358151	8.89192E-06	centroid_736	7.4927E-06
centroid_737	D-allulose-6-phosphate 3-epimerase	73	5	28	19	centroid_737	19.7358151	8.89192E-06	centroid_737	7.4927E-06
centroid_738	D-allose kinase	73	5	28	19	centroid_738	19.7358151	8.89192E-06	centroid_738	7.4927E-06
centroid_9256	ulp1 protease family, C-terminal catalytic domain protein	73	5	28	19	centroid_925	19.7358151	8.89192E-06	centroid_925	7.4927E-06
centroid_12709	bacterial regulatory helix-turn-helix, AraC family protein	73	6	28	18	centroid_1270	16.6590528	4.47363E-05	centroid_1270	3.1056E-05
centroid_14191	CFA/I fimbrial subunit D domain protein	73	6	28	18	centroid_1419	16.6590528	4.47363E-05	centroid_1419	3.1056E-05
centroid_17756	conserved hypothetical protein	72	2	29	22	centroid_1775	29.2658374	6.30984E-08	centroid_1775	1.3555E-08
centroid_14468	putative aatD, apolipoprotein N-acyltransferase	72	3	29	21	centroid_1446	25.5281388	4.35978E-07	centroid_1446	1.4913E-07
centroid_2426	conserved hypothetical protein	72	4	29	20	centroid_242	22.0364806	2.67517E-06	centroid_242	1.2168E-06
centroid_270	conserved hypothetical protein	72	4	29	20	centroid_270	22.0364806	2.67517E-06	centroid_270	1.2168E-06
centroid_4203	CFA/I fimbrial subunit D	72	6	29	18	centroid_420	15.7901719	7.07692E-05	centroid_420	4.3945E-05
centroid_16443	D-serine deaminase transcriptional activator	71	1	30	23	centroid_1644	32.0695183	1.48753E-08	centroid_1644	1.4185E-09
centroid_15730	putative membrane protein	71	3	30	21	centroid_1573	24.4800473	7.50834E-07	centroid_1573	2.4403E-07
centroid_16886	orn/Lys/Arg decarboxylase, major domain protein	71	3	30	21	centroid_1688	24.4800473	7.50834E-07	centroid_1688	2.4403E-07
centroid_4206	putative membrane protein	71	3	30	21	centroid_420	24.4800473	7.50834E-07	centroid_420	2.4403E-07
centroid_8347	aatD, apolipoN-acyltransferase domain protein	71	3	30	21	centroid_834	24.4800473	7.50834E-07	centroid_834	2.4403E-07
centroid_11294	porin, autotransporter (AT) family	71	4	30	20	centroid_1129	21.0589418	4.4537E-06	centroid_1129	2.7294E-06
centroid_13494	e ATP-binding component of a transport system domain	71	4	30	20	centroid_1349	21.0589418	4.4537E-06	centroid_1349	2.7294E-06
centroid_17797	conserved hypothetical protein	71	4	30	20	centroid_1779	21.0589418	4.4537E-06	centroid_1779	2.7294E-06
centroid_1911	outer membrane autotransporter barrel domain protein	71	4	30	20	centroid_191	21.0589418	4.4537E-06	centroid_191	2.7294E-06
centroid_4111	transposase for ISEc12 domain protein	71	4	30	20	centroid_411	21.0589418	4.4537E-06	centroid_411	2.7294E-06
centroid_8275	phage tail tape measure protein, TP901 family, core regior	71	5	30	19	centroid_827	17.8857273	2.34574E-05	centroid_827	1.3206E-05
centroid_10799	-negative pili assembly chaperone, C-terminal domain pr	70	0	31	24	centroid_1079	35.0433202	3.22451E-09	centroid_1079	7.7305E-11
centroid_16913	putative membrane protein	70	0	31	24	centroid_1691	35.0433202	3.22451E-09	centroid_1691	7.7305E-11
centroid_1250	D-serine deaminase transcriptional activator	70	1	31	23	centroid_125	30.9321508	2.67208E-08	centroid_125	2.4386E-09
centroid_1239	-negative pili assembly chaperone, N-terminal domain pr	69	0	32	24	centroid_123	33.8878812	5.83812E-09	centroid_123	1.3528E-10
centroid_16914	-negative pili assembly chaperone, N-terminal domain pr	69	0	32	24	centroid_1691	33.8878812	5.83812E-09	centroid_1691	1.3528E-10
centroid_4811	putative 4'-phosphopantetheinyl transferase EntD	69	0	32	24	centroid_481	33.8878812	5.83812E-09	centroid_481	1.3528E-10
centroid_1092	mRNA interferase HigB	69	1	32	23	centroid_109	29.8363263	4.70101E-08	centroid_109	4.1358E-09
centroid_13618	rhs element Vgr family protein	68	4	33	20	centroid_1361	18.3566568	1.83178E-05	centroid_1361	1.3752E-05
centroid_4752	putative dNA-binding transcriptional regulator	68	4	33	20	centroid_475	18.3566568	1.83178E-05	centroid_475	1.3752E-05
centroid_10045	putative nucleic acid-binding protein	68	5	33	19	centroid_1004	15.3936825	8.72796E-05	centroid_1004	4.9834E-05
centroid_13984	helix-turn-helix domain protein	68	5	33	19	centroid_1398	15.3936825	8.72796E-05	centroid_1398	4.9834E-05
centroid_15675	putative nucleic acid-binding protein	68	5	33	19	centroid_1567	15.3936825	8.72796E-05	centroid_1567	4.9834E-05
centroid_3263	conserved hypothetical protein	68	5	33	19	centroid_326	15.3936825	8.72796E-05	centroid_326	4.9834E-05
centroid_15359	conserved hypothetical protein	66	4	35	20	centroid_1535	16.7267564	4.31679E-05	centroid_1535	1.9088E-05
centroid_1175	von Willebrand factor family protein	65	0	36	24	centroid_117	29.6514853	5.1713E-08	centroid_117	1.1199E-09
centroid_6930	conserved hypothetical protein	65	0	36	24	centroid_693	29.6514853	5.1713E-08	centroid_693	1.1199E-09
centroid_3558	caudovirales tail fibre assembly family protein	65	1	36	23	centroid_355	25.8263677	3.7355E-07	centroid_355	4.9635E-08
centroid_1176	peptidase M20/M25/M40 family protein	65	4	36	20	centroid_117	15.9579777	6.47643E-05	centroid_117	2.5617E-05
centroid_10249	conserved hypothetical protein	64	0	37	24	centroid_1024	28.6792552	8.54138E-08	centroid_1024	1.8464E-09
centroid_1179	UBA/TS-N domain protein	64	0	37	24	centroid_117	28.6792552	8.54138E-08	centroid_117	1.8464E-09

centroid_1180	conserved hypothetical protein	64	0	37	24	centroid_1180	28.6792552	8.54138E-08	centroid_1180	1.8464E-09
centroid_1181	tetratricopeptide repeat family protein	64	0	37	24	centroid_1181	28.6792552	8.54138E-08	centroid_1181	1.8464E-09
centroid_2946	n-negative pili assembly chaperone, C-terminal domain pr	64	0	37	24	centroid_2946	28.6792552	8.54138E-08	centroid_2946	1.8464E-09
centroid_4219	hypothetical protein	64	3	37	21	centroid_4219	18.1809862	2.00874E-05	centroid_4219	6.3655E-06
centroid_9677	putative transposase	64	3	37	21	centroid_9677	18.1809862	2.00874E-05	centroid_9677	6.3655E-06
centroid_14287	autotransporter beta-domain protein	64	4	37	20	centroid_1428	15.2179169	9.57903E-05	centroid_1428	5.5542E-05
centroid_1235	conserved hypothetical protein	63	0	38	24	centroid_1235	27.7384122	1.38878E-07	centroid_1235	3.0125E-09
centroid_1236	fimbrial family protein	63	0	38	24	centroid_1236	27.7384122	1.38878E-07	centroid_1236	3.0125E-09
centroid_1237	fimbrial family protein	63	0	38	24	centroid_1237	27.7384122	1.38878E-07	centroid_1237	3.0125E-09
centroid_1238	fimbrial family protein	63	0	38	24	centroid_1238	27.7384122	1.38878E-07	centroid_1238	3.0125E-09
centroid_13865	fimbrial family protein	63	0	38	24	centroid_1386	27.7384122	1.38878E-07	centroid_1386	3.0125E-09
centroid_14007	RhsB domain protein	63	1	38	23	centroid_1400	24.0198057	9.53498E-07	centroid_1400	8.4264E-08
centroid_17771	hypothetical protein	63	1	38	23	centroid_1777	24.0198057	9.53498E-07	centroid_1777	8.4264E-08
centroid_373	conserved hypothetical protein	63	1	38	23	centroid_373	24.0198057	9.53498E-07	centroid_373	8.4264E-08
centroid_8305	RHS repeat-associated core domain protein	63	1	38	23	centroid_8305	24.0198057	9.53498E-07	centroid_8305	8.4264E-08
centroid_9920	RHS repeat-associated core domain protein	63	1	38	23	centroid_9920	24.0198057	9.53498E-07	centroid_9920	8.4264E-08
centroid_12832	conserved hypothetical protein	63	3	38	21	centroid_1283	17.407226	3.01677E-05	centroid_1283	8.6631E-06
centroid_10911	tnsA endonuclease N terminal family protein	62	1	39	23	centroid_1091	23.1605615	1.49022E-06	centroid_1091	1.2467E-07
centroid_11449	putative tn7-like transposition protein C	62	1	39	23	centroid_1144	23.1605615	1.49022E-06	centroid_1144	1.2467E-07
centroid_4049	tniQ family protein	62	1	39	23	centroid_4049	23.1605615	1.49022E-06	centroid_4049	1.2467E-07
centroid_14128	e kinase-, DNA gyrase B-, and HSP90-like ATPase family	62	2	39	22	centroid_1412	19.7731352	8.71997E-06	centroid_1412	1.7487E-06
centroid_17103	CRISPR type I-E/ECOLI-associated protein CasA/Cse1	62	2	39	22	centroid_1710	19.7731352	8.71997E-06	centroid_1710	1.7487E-06
centroid_3474	putative copper-binding protein PcoE	62	2	39	22	centroid_3474	19.7731352	8.71997E-06	centroid_3474	1.7487E-06
centroid_17155	helix-turn-helix domain protein	61	0	40	24	centroid_1715	25.945007	3.51283E-07	centroid_1715	9.6325E-09
centroid_3475	heavy metal sensor kinase family protein	61	1	40	23	centroid_3475	22.3288251	2.29732E-06	centroid_3475	3.057E-07
centroid_3486	cation efflux system protein CusA	61	1	40	23	centroid_3486	22.3288251	2.29732E-06	centroid_3486	3.057E-07
centroid_3487	efflux transporter, RND family, MFP subunit	61	1	40	23	centroid_3487	22.3288251	2.29732E-06	centroid_3487	3.057E-07
centroid_3488	cation efflux system protein CusF	61	1	40	23	centroid_3488	22.3288251	2.29732E-06	centroid_3488	3.057E-07
centroid_3489	ansporter, outer membrane factor (OMF) lipo, NodT famil	61	1	40	23	centroid_3489	22.3288251	2.29732E-06	centroid_3489	3.057E-07
centroid_3490	transcriptional regulatory protein CusR	61	1	40	23	centroid_3490	22.3288251	2.29732E-06	centroid_3490	3.057E-07
centroid_3491	heavy metal sensor kinase family protein	61	1	40	23	centroid_3491	22.3288251	2.29732E-06	centroid_3491	3.057E-07
centroid_10828	putative metalloprotease YebA domain protein	61	2	40	22	centroid_1082	18.9952784	1.31042E-05	centroid_1082	2.3875E-06
centroid_10928	peptidase M23 family protein	61	2	40	22	centroid_1092	18.9952784	1.31042E-05	centroid_1092	2.3875E-06
centroid_12338	conserved hypothetical protein	61	2	40	22	centroid_1233	18.9952784	1.31042E-05	centroid_1233	2.3875E-06
centroid_18160	putative pcoS	61	2	40	22	centroid_1816	18.9952784	1.31042E-05	centroid_1816	2.3875E-06
centroid_18161	efflux transporter, RND family, MFP subunit	61	2	40	22	centroid_1816	18.9952784	1.31042E-05	centroid_1816	2.3875E-06
centroid_18179	heavy metal sensor kinase family protein	61	2	40	22	centroid_1817	18.9952784	1.31042E-05	centroid_1817	2.3875E-06
centroid_3476	response regulator	61	2	40	22	centroid_3476	18.9952784	1.31042E-05	centroid_3476	2.3875E-06
centroid_3477	copper resistance D family protein	61	2	40	22	centroid_3477	18.9952784	1.31042E-05	centroid_3477	2.3875E-06
centroid_3478	copper resistance protein C	61	2	40	22	centroid_3478	18.9952784	1.31042E-05	centroid_3478	2.3875E-06
centroid_3479	copper resistance protein B	61	2	40	22	centroid_3479	18.9952784	1.31042E-05	centroid_3479	2.3875E-06
centroid_3480	copper resistance protein A	61	2	40	22	centroid_3480	18.9952784	1.31042E-05	centroid_3480	2.3875E-06
centroid_3481	putative copper resistant protein PcoE	61	2	40	22	centroid_3481	18.9952784	1.31042E-05	centroid_3481	2.3875E-06
centroid_3483	conserved hypothetical protein	61	2	40	22	centroid_3483	18.9952784	1.31042E-05	centroid_3483	2.3875E-06
centroid_3484	copper-translocating P-type ATPase	61	2	40	22	centroid_3484	18.9952784	1.31042E-05	centroid_3484	2.3875E-06
centroid_4847	conserved hypothetical protein	61	3	40	21	centroid_4847	15.9392437	6.54084E-05	centroid_4847	2.2075E-05
centroid_1178	conserved hypothetical protein	60	0	41	24	centroid_1178	25.089731	5.47235E-07	centroid_1178	1.3464E-08
centroid_3323	integrase core domain protein	60	0	41	24	centroid_3323	25.089731	5.47235E-07	centroid_3323	1.3464E-08
centroid_4332	calcineurin-like phosphoesterase family protein	60	0	41	24	centroid_4332	25.089731	5.47235E-07	centroid_4332	1.3464E-08
centroid_6506	hypothetical protein	60	0	41	24	centroid_6506	25.089731	5.47235E-07	centroid_6506	1.3464E-08
centroid_7671	conserved hypothetical protein	60	0	41	24	centroid_7671	25.089731	5.47235E-07	centroid_7671	1.3464E-08
centroid_10929	conserved hypothetical protein	60	1	41	23	centroid_1092	21.5233182	3.49552E-06	centroid_1092	3.6229E-07
centroid_15430	tnsB domain protein	60	1	41	23	centroid_1543	21.5233182	3.49552E-06	centroid_1543	3.6229E-07
centroid_3324	tnsA endonuclease N terminal family protein	60	1	41	23	centroid_3324	21.5233182	3.49552E-06	centroid_3324	3.6229E-07
centroid_4047	putative tnsB domain protein	60	1	41	23	centroid_4047	21.5233182	3.49552E-06	centroid_4047	3.6229E-07
centroid_6507	conserved hypothetical protein	60	1	41	23	centroid_6507	21.5233182	3.49552E-06	centroid_6507	3.6229E-07
centroid_6593	conserved hypothetical protein	60	1	41	23	centroid_6593	21.5233182	3.49552E-06	centroid_6593	3.6229E-07
centroid_4357	putative pantothenate kinase Pantothenic acid kinase	60	3	41	21	centroid_4357	15.242563	9.45483E-05	centroid_4357	2.8199E-05
centroid_4860	CFA/III pilin	59	0	42	24	centroid_4860	24.2604007	8.41514E-07	centroid_4860	2.0069E-08
centroid_864	putative membrane protein	59	0	42	24	centroid_864	24.2604007	8.41514E-07	centroid_864	2.0069E-08
centroid_17486	silver exporting P-type ATPase domain protein	59	1	42	23	centroid_1748	20.742842	5.25276E-06	centroid_1748	4.839E-07
centroid_14725	o-4-hydroxy-6-hydroxymethyldihydropteridine diphosphc	59	2	42	22	centroid_1472	17.5144084	2.85139E-05	centroid_1472	6.3162E-06
centroid_15907	conserved hypothetical protein	59	2	42	22	centroid_1590	17.5144084	2.85139E-05	centroid_1590	6.3162E-06
centroid_16583	CRISPR type I-E/ECOLI-associated protein CasA/Cse1	59	2	42	22	centroid_1658	17.5144084	2.85139E-05	centroid_1658	6.3162E-06
centroid_3482	peptidase M23 family protein	59	2	42	22	centroid_3482	17.5144084	2.85139E-05	centroid_3482	6.3162E-06
centroid_10746	putative ybl54	58	0	43	24	centroid_1074	23.4558559	1.27813E-06	centroid_1074	4.8936E-08
centroid_15898	putative predicted protein	58	0	43	24	centroid_1589	23.4558559	1.27813E-06	centroid_1589	4.8936E-08
centroid_17655	conserved hypothetical protein	58	0	43	24	centroid_1765	23.4558559	1.27813E-06	centroid_1765	4.8936E-08
centroid_8703	putative ybl54	58	0	43	24	centroid_8703	23.4558559	1.27813E-06	centroid_8703	4.8936E-08
centroid_15801	RHS repeat-associated core domain protein	58	1	43	23	centroid_1580	19.986271	7.80002E-06	centroid_1580	1.2857E-06
centroid_4048	AAA domain protein	58	1	43	23	centroid_4048	19.986271	7.80002E-06	centroid_4048	1.2857E-06
centroid_7896	RHS repeat-associated core domain protein	58	1	43	23	centroid_7896	19.986271	7.80002E-06	centroid_7896	1.2857E-06
centroid_2336	CRISPR type I-E/ECOLI-associated protein CasA/Cse1	58	2	43	22	centroid_2336	16.8091554	4.13334E-05	centroid_2336	8.0764E-06
centroid_2780	flagellar biosynthesis protein FlhA	58	2	43	22	centroid_2780	16.8091554	4.13334E-05	centroid_2780	8.0764E-06
centroid_16888	conserved hypothetical protein	57	0	44	24	centroid_1688	22.6750045	1.91847E-06	centroid_1688	5.816E-08
centroid_7913	hypothetical protein	57	0	44	24	centroid_7913	22.6750045	1.91847E-06	centroid_7913	5.816E-08
centroid_16884	outer membrane autotransporter barrel domain protein	57	1	44	23	centroid_1688	19.2525478	1.14517E-05	centroid_1688	1.3912E-06
centroid_2779	conserved hypothetical protein	55	0	46	24	centroid_2779	21.1803267	4.18034E-06	centroid_2779	2.1071E-07
centroid_13662	ATP-dependent endonuclease, OLD family domain protei	55	1	46	23	centroid_1366	17.849726	2.39054E-05	centroid_1366	2.3273E-06
centroid_15488	outer membrane autotransporter barrel domain protein	55	1	46	23	centroid_1548	17.849726	2.39054E-05	centroid_1548	2.3273E-06
centroid_4051	helix-turn-helix family protein	55	1	46	23	centroid_4051	17.849726	2.39054E-05	centroid_4051	2.3273E-06
centroid_7633	conserved hypothetical protein	55	1	46	23	centroid_7633	17.849726	2.39054E-05	centroid_7633	2.3273E-06
centroid_374	RHS repeat-associated core domain protein	54	0	47	24	centroid_374	20.4646158	6.07439E-06	centroid_374	2.2733E-07
centroid_4050	conserved hypothetical protein	54	1	47	23	centroid_4050	17.1788105	3.4021E-05	centroid_4050	4.703E-06
centroid_4052	AAA ATPase domain protein	54	1	47	23	centroid_4052	17.1788105	3.4021E-05	centroid_4052	4.703E-06
centroid_4599	fimbrial family protein	53	0	48	24	centroid_4599	19.7688214	8.73968E-06	centroid_4599	2.8511E-07
centroid_11599	TS system, galactitol-specific IIC component family prote	53	24	48	0	centroid_1159	16.561509	4.70973E-05	centroid_1159	2.4572E-06
centroid_4303	galactitol-1-phosphate 5-dehydrogenase	53	24	48	0	centroid_4303	16.561509	4.70973E-05	centroid_4303	2.4572E-06
centroid_4304	bacterial regulatory, gntR family protein	53	24	48	0	centroid_4304	16.561509	4.70973E-05	centroid_4304	2.4572E-06
centroid_7704	putative lipid A biosynthesis protein	53	24	48	0	centroid_7704	16.561509	4.70973E-05	centroid_7704	2.4572E-06
centroid_8672	utative aDP-heptose:LPS heptosyltransferase-like protei	53	24	48	0	centroid_8672	16.561509	4.70973E-05	centroid_8672	2.4572E-06
centroid_3492	silver-binding protein SilE	53	1	48	23	centroid_3492	16.5271011	4.79597E-05	centroid_3492	5.5966E-06
centroid_3493	conserved hypothetical protein	53	1	48	23	centroid_3493	16.5271011	4.79597E-05	centroid_3493	5.5966E-06
centroid_5014	helix-turn-helix family protein	52	0	49	24	centroid_5014	19.0921268	1.24558E-05	centroid_5014	3.9083E-07
centroid_5015	conserved hypothetical protein	52	0	49	24	centroid_5015	19.0921268	1.24558E-05	centroid_5015	3.9083E-07
centroid_13033	RHS repeat-associated core domain protein	52	1	49	23	centroid_1303	15.8938147	6.69973E-05	centroid_1303	7.2852E-06
centroid_10608	transposase DDE domain protein	51	0	50	24	centroid_1060	18.43376	1.75914E-05	centroid_1060	7.8857E-07
centroid_13558	transposase DDE domain protein	51	0	50	24	centroid_1355	18.43376	1.75914E-05	centroid_1355	7.8857E-07
centroid_16152	putative iS903 transposase	51	0	50	24	centroid_1615	18.43376	1.75914E-05	centroid_1615	7.8857E-07
centroid_3748	conserved hypothetical protein	51	0	50	24	centroid_3748	18.43376	1.75914E-05	centroid_3748	7.8857E-07
centroid_3749	helix-turn-helix domain protein	51	0	50	24	centroid_3749	18.43376	1.75914E-05	centroid_3749	7.8857E-07
centroid_7784	cytidylyltransferase family protein	51	24	50	0	centroid_7784	17.7929902	2.46289E-05	centroid_7784	9.4992E-07
centroid_1579	RHS repeat-associated core domain protein	51	1	50	23	centroid_1579	15.2782123	9.27805E-05	centroid_1579	1.4205E-05
centroid_4440	bacterial regulatory helix-turn-helix, AraC family protein	50	24	51	0	centroid_4440	18.43376	1.75914E-05	centroid_4440	7.8857E-07
centroid_4441	conserved hypothetical protein	50	24	51	0	centroid_4441	18.43376	1.75914E-05	centroid_4441	7.8857E-07
centroid_4442	sulfatase family protein	50	24	51	0	centroid_4442	18.43376	1.75914E-05	centroid_4442	7.8857E-07
centroid_4443	inner membrane protein YdI	50	24	51	0	centroid_4443	18.43376	1.75914E-05	centroid_4443	7.8857E-07

centroid_6460	sulfatase family protein	50	24	51	0	centroid_646	18.43376	1.75914E-05	centroid_646	7.8857E-07
centroid_6461	sulfatase family protein	50	24	51	0	centroid_646	18.43376	1.75914E-05	centroid_646	7.8857E-07
centroid_8995	conserved hypothetical protein	50	24	51	0	centroid_899	18.43376	1.75914E-05	centroid_899	7.8857E-07
centroid_8996	sodium:solute symporter family protein	50	24	51	0	centroid_899	18.43376	1.75914E-05	centroid_899	7.8857E-07
centroid_9344	utative DNA-binding transcriptional regulator domain prote	50	24	51	0	centroid_934	18.43376	1.75914E-05	centroid_934	7.8857E-07
centroid_9345	bacterial regulatory helix-turn-helix, AraC family protein	50	24	51	0	centroid_934	18.43376	1.75914E-05	centroid_934	7.8857E-07
centroid_15320	gram-negative porin family protein	50	0	51	24	centroid_1532	17.7929902	2.46289E-05	centroid_1532	9.4992E-07
centroid_17666	putative phage tail fiber domain protein	50	0	51	24	centroid_1766	17.7929902	2.46289E-05	centroid_1766	9.4992E-07
centroid_3223	outer membrane porin protein OmpD	50	0	51	24	centroid_322	17.7929902	2.46289E-05	centroid_322	9.4992E-07
centroid_4232	transposase DDE domain protein	50	0	51	24	centroid_423	17.7929902	2.46289E-05	centroid_423	9.4992E-07
centroid_4078	chaperone of endosialidase family protein	49	0	52	24	centroid_407	17.1691252	3.41949E-05	centroid_407	2.3872E-06
centroid_4102	outer membrane porin protein OmpD	49	0	52	24	centroid_410	17.1691252	3.41949E-05	centroid_410	2.3872E-06
centroid_8308	autotransporter beta-domain protein	49	0	52	24	centroid_830	17.1691252	3.41949E-05	centroid_830	2.3872E-06
centroid_4541	glycosyltransferase 9 family protein	48	24	53	0	centroid_454	19.7688214	8.73968E-06	centroid_454	2.8511E-07
centroid_7194	ftsK/SpoIIIE family protein	48	24	53	0	centroid_719	19.7688214	8.73968E-06	centroid_719	2.8511E-07
centroid_9248	ftsK/SpoIIIE family protein	48	24	53	0	centroid_924	19.7688214	8.73968E-06	centroid_924	2.8511E-07
centroid_3274	lysR substrate binding domain protein	48	0	53	24	centroid_327	16.561509	4.70973E-05	centroid_327	2.4572E-06
centroid_3275	aldo/keto reductase family protein	48	0	53	24	centroid_327	16.561509	4.70973E-05	centroid_327	2.4572E-06
centroid_3276	enoyl-(Acyl carrier) reductase family protein	48	0	53	24	centroid_327	16.561509	4.70973E-05	centroid_327	2.4572E-06
centroid_3277	yketide cyclase / dehydrase and lipid transport family pro	48	0	53	24	centroid_327	16.561509	4.70973E-05	centroid_327	2.4572E-06
centroid_3278	conserved hypothetical protein	48	0	53	24	centroid_327	16.561509	4.70973E-05	centroid_327	2.4572E-06
centroid_4344	conserved hypothetical protein	48	0	53	24	centroid_434	16.561509	4.70973E-05	centroid_434	2.4572E-06
centroid_4662	cytidylyltransferase family protein	47	24	54	0	centroid_466	20.4646158	6.07439E-06	centroid_466	2.2733E-07
centroid_4663	CDP-alcohol phosphatidyltransferase family protein	47	23	54	1	centroid_466	17.1788105	3.4021E-05	centroid_466	4.703E-06
centroid_10276	major Facilitator Superfamily protein	47	0	54	24	centroid_1027	15.9695194	6.43706E-05	centroid_1027	2.9197E-06
centroid_10277	major Facilitator Superfamily protein	47	0	54	24	centroid_1027	15.9695194	6.43706E-05	centroid_1027	2.9197E-06
centroid_10419	RHS repeat-associated core domain protein	47	0	54	24	centroid_1041	15.9695194	6.43706E-05	centroid_1041	2.9197E-06
centroid_1524	HAD hydrolase, IA, variant 3 family protein	47	0	54	24	centroid_152	15.9695194	6.43706E-05	centroid_152	2.9197E-06
centroid_1525	polyol permease family protein	47	0	54	24	centroid_152	15.9695194	6.43706E-05	centroid_152	2.9197E-06
centroid_1526	xylulokinase	47	0	54	24	centroid_152	15.9695194	6.43706E-05	centroid_152	2.9197E-06
centroid_1527	mannitol dehydrogenase C-terminal domain protein	47	0	54	24	centroid_152	15.9695194	6.43706E-05	centroid_152	2.9197E-06
centroid_1528	putative sugar-binding domain protein	47	0	54	24	centroid_152	15.9695194	6.43706E-05	centroid_152	2.9197E-06
centroid_1529	helix-turn-helix family protein	47	0	54	24	centroid_152	15.9695194	6.43706E-05	centroid_152	2.9197E-06
centroid_1530	ribitol 2-dehydrogenase	47	0	54	24	centroid_153	15.9695194	6.43706E-05	centroid_153	2.9197E-06
centroid_1531	FGGY-pentulose kinase family protein	47	0	54	24	centroid_153	15.9695194	6.43706E-05	centroid_153	2.9197E-06
centroid_4199	putative transposase	47	0	54	24	centroid_419	15.9695194	6.43706E-05	centroid_419	2.9197E-06
centroid_5278	major Facilitator Superfamily protein	47	0	54	24	centroid_527	15.9695194	6.43706E-05	centroid_527	2.9197E-06
centroid_5369	major Facilitator Superfamily protein	47	0	54	24	centroid_536	15.9695194	6.43706E-05	centroid_536	2.9197E-06
centroid_7679	putative membrane protein	47	0	54	24	centroid_767	15.9695194	6.43706E-05	centroid_767	2.9197E-06
centroid_7680	major Facilitator Superfamily protein	47	0	54	24	centroid_768	15.9695194	6.43706E-05	centroid_768	2.9197E-06
centroid_16870	hypothetical protein	46	23	55	1	centroid_1687	17.849726	2.39054E-05	centroid_1687	2.3273E-06
centroid_9351	type IV leader peptidase family protein	41	22	60	2	centroid_935	18.242755	1.94464E-05	centroid_935	5.4848E-06
centroid_2008	conserved hypothetical protein	41	21	60	3	centroid_200	15.242563	9.45483E-05	centroid_200	2.8199E-05
centroid_3499	putative lipoprotein	41	21	60	3	centroid_349	15.242563	9.45483E-05	centroid_349	2.8199E-05
centroid_4382	putative membrane protein	40	21	61	3	centroid_438	15.9392437	6.54084E-05	centroid_438	2.2075E-05
centroid_4383	conserved hypothetical protein	40	21	61	3	centroid_438	15.9392437	6.54084E-05	centroid_438	2.2075E-05
centroid_4384	conserved hypothetical protein	40	21	61	3	centroid_438	15.9392437	6.54084E-05	centroid_438	2.2075E-05
centroid_5551	conserved hypothetical protein	40	21	61	3	centroid_555	15.9392437	6.54084E-05	centroid_555	2.2075E-05
centroid_4559	conserved hypothetical protein	39	23	62	1	centroid_455	23.1605615	1.49022E-06	centroid_455	1.2467E-07
centroid_18583	type VII secretion system (T7SS), usher family protein	37	22	64	2	centroid_1858	21.4098773	3.70856E-06	centroid_1858	6.4695E-07
centroid_4629	conserved hypothetical protein	37	21	64	3	centroid_462	18.1809862	2.00874E-05	centroid_462	6.3655E-06
centroid_9633	conserved hypothetical protein	37	21	64	3	centroid_963	18.1809862	2.00874E-05	centroid_963	6.3655E-06
centroid_11126	conserved hypothetical protein	36	23	65	1	centroid_1112	25.8263677	3.7355E-07	centroid_1112	4.9635E-08
centroid_10979	n-negative pili assembly chaperone, N-terminal domain pr	36	22	65	2	centroid_1097	22.2714984	2.36694E-06	centroid_1097	4.3505E-07
centroid_12391	lamB porin family protein	36	22	65	2	centroid_1239	22.2714984	2.36694E-06	centroid_1239	4.3505E-07
centroid_14262	phosphotransferase system, EIIC family protein	36	22	65	2	centroid_1426	22.2714984	2.36694E-06	centroid_1426	4.3505E-07
centroid_17336	maltoporin periplasmic N-terminal extension family protein	36	22	65	2	centroid_1733	22.2714984	2.36694E-06	centroid_1733	4.3505E-07
centroid_17337	lamB porin family protein	36	22	65	2	centroid_1733	22.2714984	2.36694E-06	centroid_1733	4.3505E-07
centroid_4617	PTS system, Lactose/Cellobiose specific IIA subunit	36	22	65	2	centroid_461	22.2714984	2.36694E-06	centroid_461	4.3505E-07
centroid_4618	maltoporin periplasmic N-terminal extension family protein	36	22	65	2	centroid_461	22.2714984	2.36694E-06	centroid_461	4.3505E-07
centroid_4619	lamB porin family protein	36	22	65	2	centroid_461	22.2714984	2.36694E-06	centroid_461	4.3505E-07
centroid_4708	n-negative pili assembly chaperone, C-terminal domain pr	36	22	65	2	centroid_470	22.2714984	2.36694E-06	centroid_470	4.3505E-07
centroid_5810	cryptic outer membrane porin BglH	36	22	65	2	centroid_581	22.2714984	2.36694E-06	centroid_581	4.3505E-07
centroid_8525	maltoporin periplasmic N-terminal extension family protein	36	22	65	2	centroid_852	22.2714984	2.36694E-06	centroid_852	4.3505E-07
centroid_8526	lamB porin family protein	36	22	65	2	centroid_852	22.2714984	2.36694E-06	centroid_852	4.3505E-07
centroid_10540	glycosyl hydrolase 1 family protein	36	21	65	3	centroid_1054	18.9830476	1.31885E-05	centroid_1054	5.0447E-06
centroid_13126	bacterial regulatory, gntR family protein	36	21	65	3	centroid_1312	18.9830476	1.31885E-05	centroid_1312	5.0447E-06
centroid_13205	UTRA domain protein	36	21	65	3	centroid_1320	18.9830476	1.31885E-05	centroid_1320	5.0447E-06
centroid_18361	PTS system, Lactose/Cellobiose specific IIB subunit	36	21	65	3	centroid_1836	18.9830476	1.31885E-05	centroid_1836	5.0447E-06
centroid_4615	PTS system, Lactose/Cellobiose specific IIB subunit	36	21	65	3	centroid_461	18.9830476	1.31885E-05	centroid_461	5.0447E-06
centroid_4616	TS system, lactose/cellobiose IIC component family prote	36	21	65	3	centroid_461	18.9830476	1.31885E-05	centroid_461	5.0447E-06
centroid_5809	PTS system, Lactose/Cellobiose specific IIB subunit	36	21	65	3	centroid_580	18.9830476	1.31885E-05	centroid_580	5.0447E-06
centroid_7362	glycosyl hydrolase 1 family protein	36	21	65	3	centroid_736	18.9830476	1.31885E-05	centroid_736	5.0447E-06
centroid_18473	conserved hypothetical protein	36	20	65	4	centroid_1847	15.9579777	6.47643E-05	centroid_1847	2.5617E-05
centroid_4614	deoR-like helix-turn-helix domain protein	36	20	65	4	centroid_461	15.9579777	6.47643E-05	centroid_461	2.5617E-05
centroid_16140	conserved hypothetical protein	35	20	66	4	centroid_1614	16.7267564	4.31679E-05	centroid_1614	1.9088E-05
centroid_6099	conserved hypothetical protein	35	20	66	4	centroid_609	16.7267564	4.31679E-05	centroid_609	1.9088E-05
centroid_9309	yqeH domain protein	34	23	67	1	centroid_930	27.7605919	1.37294E-07	centroid_930	1.145E-08
centroid_4714	insA C-terminal domain protein	33	20	68	4	centroid_471	18.3566568	1.83178E-05	centroid_471	1.3752E-05
centroid_4715	conserved hypothetical protein	33	20	68	4	centroid_471	18.3566568	1.83178E-05	centroid_471	1.3752E-05
centroid_4716	putative dsORF-f3	33	20	68	4	centroid_471	18.3566568	1.83178E-05	centroid_471	1.3752E-05
centroid_5901	putative transposase	33	20	68	4	centroid_590	18.3566568	1.83178E-05	centroid_590	1.3752E-05
centroid_5902	putative dsORF-f3	33	20	68	4	centroid_590	18.3566568	1.83178E-05	centroid_590	1.3752E-05
centroid_7724	putative dsORF-f3	33	20	68	4	centroid_772	18.3566568	1.83178E-05	centroid_772	1.3752E-05
centroid_17544	conserved hypothetical protein	33	19	68	5	centroid_1754	15.3936825	8.72796E-05	centroid_1754	4.9834E-05
centroid_4305	plasmid stabilisation system family protein	32	22	69	2	centroid_430	26.0430419	3.3389E-07	centroid_430	6.2689E-08
centroid_4306	ribbon-helix-helix , copG family protein	32	22	69	2	centroid_430	26.0430419	3.3389E-07	centroid_430	6.2689E-08
centroid_10778	BCCT transporter family protein	31	24	70	0	centroid_1077	35.0433202	3.22451E-09	centroid_1077	7.7305E-11
centroid_13577	fimbrial family protein	31	19	70	5	centroid_1357	17.0194754	3.69984E-05	centroid_1357	1.9031E-05
centroid_13894	type VII secretion system (T7SS), usher family protein	31	19	70	5	centroid_1389	17.0194754	3.69984E-05	centroid_1389	1.9031E-05
centroid_13940	type VII secretion system (T7SS), usher family protein	31	19	70	5	centroid_1394	17.0194754	3.69984E-05	centroid_1394	1.9031E-05
centroid_16151	fimbrial family protein	31	19	70	5	centroid_1615	17.0194754	3.69984E-05	centroid_1615	1.9031E-05
centroid_17669	n-negative pili assembly chaperone, N-terminal domain pr	31	19	70	5	centroid_1766	17.0194754	3.69984E-05	centroid_1766	1.9031E-05
centroid_5841	o-4-hydroxy-6-hydroxymethyldihydropteridine diphosphc	31	19	70	5	centroid_584	17.0194754	3.69984E-05	centroid_584	1.9031E-05
centroid_5842	fimbrial family protein	31	19	70	5	centroid_584	17.0194754	3.69984E-05	centroid_584	1.9031E-05
centroid_5843	n-negative pili assembly chaperone, N-terminal domain pr	31	19	70	5	centroid_584	17.0194754	3.69984E-05	centroid_584	1.9031E-05
centroid_8553	conserved hypothetical protein	31	19	70	5	centroid_855	17.0194754	3.69984E-05	centroid_855	1.9031E-05
centroid_13286	conserved hypothetical protein	30	20	71	4	centroid_1328	21.0589418	4.4537E-06	centroid_1328	2.7294E-06
centroid_7092	hypothetical protein	30	20	71	4	centroid_709	21.0589418	4.4537E-06	centroid_709	2.7294E-06
centroid_14250	n-negative pili assembly chaperone, N-terminal domain pr	30	19	71	5	centroid_1425	17.8857273	2.34574E-05	centroid_1425	1.3206E-05
centroid_14251	n-negative pili assembly chaperone, C-terminal domain pr	30	19	71	5	centroid_1425	17.8857273	2.34574E-05	centroid_1425	1.3206E-05
centroid_18344	outer membrane usher protein HtrE	30	19	71	5	centroid_1834	17.8857273	2.34574E-05	centroid_1834	1.3206E-05
centroid_18345	papC N-terminal domain protein	30	19	71	5	centroid_1834	17.8857273	2.34574E-05	centroid_1834	1.3206E-05
centroid_4585	n-negative pili assembly chaperone, N-terminal domain pr	30	19	71	5	centroid_458	17.8857273	2.34574E-05	centroid_458	1.3206E-05
centroid_4586	CS1 type fimbrial major subunit	30	19	71	5	centroid_458	17.8857273	2.34574E-05	centroid_458	1.3206E-05
centroid_4587	putative outer membrane fimbrial usher domain protein	30	19	71	5	centroid_458	17.8857273	2.34574E-05	centroid_458	1.3206E-05
centroid_4627	chaperone protein FocC	30	19	71	5	centroid_462	17.8857273	2.34574E-05	centroid_462	1.3206E-05

centroid_5844	outer membrane usher protein HtrE	30	19	71	5	centroid_5844	17.8857273	2.34574E-05	centroid_5844	1.3206E-05
centroid_5845	fimbrial family protein	30	19	71	5	centroid_5845	17.8857273	2.34574E-05	centroid_5845	1.3206E-05
centroid_12971	conserved hypothetical protein	29	23	72	1	centroid_1297	33.2508176	8.10057E-09	centroid_1297	8.1343E-10
centroid_4408	conserved hypothetical protein	29	21	72	3	centroid_4408	25.5281388	4.35978E-07	centroid_4408	1.4913E-07
centroid_6098	conserved hypothetical protein	29	20	72	4	centroid_6098	22.0364806	2.67517E-06	centroid_6098	1.2168E-06
centroid_4904	methyltransferase domain protein	28	24	73	0	centroid_4904	38.7764089	4.75237E-10	centroid_4904	1.3245E-11
centroid_4387	hicB family protein	28	21	73	3	centroid_4387	26.6199646	2.47671E-07	centroid_4387	8.9816E-08
centroid_17338	type VII secretion system (T7SS), usher family protein	28	20	73	4	centroid_1733	23.0562991	1.57326E-06	centroid_1733	7.568E-07
centroid_4717	conserved hypothetical protein	28	20	73	4	centroid_4717	23.0562991	1.57326E-06	centroid_4717	7.568E-07
centroid_11133	hypothetical protein	28	19	73	5	centroid_1113	19.7358151	8.89192E-06	centroid_1113	7.4927E-06
centroid_15015	conserved hypothetical protein	28	19	73	5	centroid_1501	19.7358151	8.89192E-06	centroid_1501	7.4927E-06
centroid_4626	type-1 fimbrial protein, A chain	28	19	73	5	centroid_4626	19.7358151	8.89192E-06	centroid_4626	7.4927E-06
centroid_10927	conserved hypothetical protein	28	18	73	6	centroid_1092	16.6590528	4.47363E-05	centroid_1092	3.1056E-05
centroid_11168	conserved hypothetical protein	28	18	73	6	centroid_1116	16.6590528	4.47363E-05	centroid_1116	3.1056E-05
centroid_13619	conserved hypothetical protein	28	18	73	6	centroid_1361	16.6590528	4.47363E-05	centroid_1361	3.1056E-05
centroid_18481	conserved hypothetical protein	28	18	73	6	centroid_1848	16.6590528	4.47363E-05	centroid_1848	3.1056E-05
centroid_4607	phenolic acid decarboxylase subunit D	27	23	74	1	centroid_4607	35.755724	2.23674E-09	centroid_4607	2.5549E-10
centroid_4608	ubiD decarboxylase family protein	27	23	74	1	centroid_4608	35.755724	2.23674E-09	centroid_4608	2.5549E-10
centroid_4609	putative aromatic acid decarboxylase	27	23	74	1	centroid_4609	35.755724	2.23674E-09	centroid_4609	2.5549E-10
centroid_4610	marR family protein	27	23	74	1	centroid_4610	35.755724	2.23674E-09	centroid_4610	2.5549E-10
centroid_5672	ubiD decarboxylase family protein	27	23	74	1	centroid_5672	35.755724	2.23674E-09	centroid_5672	2.5549E-10
centroid_5673	octaprenyl-4-hydroxybenzoate carboxy-lyase family prot	27	23	74	1	centroid_5673	35.755724	2.23674E-09	centroid_5673	2.5549E-10
centroid_8769	conserved domain protein	27	23	74	1	centroid_8769	35.755724	2.23674E-09	centroid_8769	2.5549E-10
centroid_4390	conserved hypothetical protein	27	21	74	3	centroid_4390	27.7582202	1.37463E-07	centroid_4390	5.3269E-08
centroid_5759	conserved hypothetical protein	27	21	74	3	centroid_5759	27.7582202	1.37463E-07	centroid_5759	5.3269E-08
centroid_17931	conserved hypothetical protein	26	18	75	6	centroid_1793	18.5245991	1.67726E-05	centroid_1793	1.8759E-05
centroid_18482	conserved hypothetical protein	26	18	75	6	centroid_1848	18.5245991	1.67726E-05	centroid_1848	1.8759E-05
centroid_15014	conserved hypothetical protein	26	17	75	7	centroid_1501	15.5307018	8.11761E-05	centroid_1501	6.7215E-05
centroid_5564	conserved hypothetical protein	26	17	75	7	centroid_5564	15.5307018	8.11761E-05	centroid_5564	6.7215E-05
centroid_14951	conserved hypothetical protein	25	19	76	5	centroid_1495	22.8436074	1.75733E-06	centroid_1495	1.2179E-06
centroid_9569	glycosyl hydrolases 15 family protein	24	18	77	6	centroid_9569	20.5800253	5.71897E-06	centroid_9569	4.581E-06
centroid_5861	glycosyl hydrolases 15 family protein	24	17	77	7	centroid_5861	17.4162461	3.00249E-05	centroid_5861	4.4073E-05
centroid_9789	conserved hypothetical protein	24	17	77	7	centroid_9789	17.4162461	3.00249E-05	centroid_9789	4.4073E-05
centroid_11584	biquinone/plastoquinone (complex I), various chains fami	23	24	78	0	centroid_1158	46.0576857	1.14822E-11	centroid_1158	5.0087E-13
centroid_5580	H-Ubiquinone oxidoreductase (complex I), chain family pr	23	24	78	0	centroid_5580	46.0576857	1.14822E-11	centroid_5580	5.0087E-13
centroid_7285	biquinone/plastoquinone (complex I), various chains fami	23	24	78	0	centroid_7285	46.0576857	1.14822E-11	centroid_7285	5.0087E-13
centroid_13521	conjugative transfer relaxase protein TraI	23	18	78	6	centroid_1352	21.6873478	3.20901E-06	centroid_1352	2.8036E-06
centroid_15858	nucleotide sugar dehydrogenase family protein	23	18	78	6	centroid_1585	21.6873478	3.20901E-06	centroid_1585	2.8036E-06
centroid_12918	conserved hypothetical protein	22	19	79	5	centroid_1291	26.4263615	2.73778E-07	centroid_1291	2.5159E-07
centroid_15012	conserved hypothetical protein	22	19	79	5	centroid_1501	26.4263615	2.73778E-07	centroid_1501	2.5159E-07
centroid_5630	conserved hypothetical protein	22	19	79	5	centroid_5630	26.4263615	2.73778E-07	centroid_5630	2.5159E-07
centroid_6604	conserved hypothetical protein	22	19	79	5	centroid_6604	26.4263615	2.73778E-07	centroid_6604	2.5159E-07
centroid_6605	conserved hypothetical protein	22	19	79	5	centroid_6605	26.4263615	2.73778E-07	centroid_6605	2.5159E-07
centroid_6606	adhesin domain protein	22	19	79	5	centroid_6606	26.4263615	2.73778E-07	centroid_6606	2.5159E-07
centroid_16717	hypothetical protein	22	18	79	6	centroid_1671	22.8529086	1.74884E-06	centroid_1671	1.6832E-06
centroid_10025	conserved hypothetical protein	21	19	80	5	centroid_1002	27.7442525	1.38459E-07	centroid_1002	1.4285E-07
centroid_11324	conserved hypothetical protein	21	19	80	5	centroid_1132	27.7442525	1.38459E-07	centroid_1132	1.4285E-07
centroid_16718	conserved hypothetical protein	21	19	80	5	centroid_1671	27.7442525	1.38459E-07	centroid_1671	1.4285E-07
centroid_5714	conserved hypothetical protein	21	19	80	5	centroid_5714	27.7442525	1.38459E-07	centroid_5714	1.4285E-07
centroid_8551	conserved hypothetical protein	21	19	80	5	centroid_8551	27.7442525	1.38459E-07	centroid_8551	1.4285E-07
centroid_6980	conserved hypothetical protein	20	19	81	5	centroid_6980	29.1317632	6.76192E-08	centroid_6980	7.9315E-08
centroid_11757	rhs core with extension domain protein	20	17	81	7	centroid_1175	21.8473639	2.95222E-06	centroid_1175	3.4568E-06
centroid_10954	intimin C-type lectin domain protein	20	15	81	9	centroid_1095	15.4826774	8.32648E-05	centroid_1095	8.2179E-05
centroid_5633	intimin C-type lectin domain protein	20	15	81	9	centroid_5633	15.4826774	8.32648E-05	centroid_5633	8.2179E-05
centroid_8549	intimin C-type lectin domain protein	20	15	81	9	centroid_8549	15.4826774	8.32648E-05	centroid_8549	8.2179E-05
centroid_16839	bacterial Ig-like domain family protein	19	15	82	9	centroid_1683	16.5505219	4.73709E-05	centroid_1683	5.1641E-05
centroid_12447	bacterial Ig-like domain family protein	18	15	83	9	centroid_1244	17.6889335	2.60136E-05	centroid_1244	3.1705E-05
centroid_17335	bacterial Ig-like domain family protein	18	15	83	9	centroid_1733	17.6889335	2.60136E-05	centroid_1733	3.1705E-05
centroid_5632	bacterial Ig-like domain family protein	18	15	83	9	centroid_5632	17.6889335	2.60136E-05	centroid_5632	3.1705E-05
centroid_13112	bacterial Ig-like domain family protein	17	15	84	9	centroid_1311	18.904333	1.3744E-05	centroid_1311	1.8983E-05
centroid_6608	bacterial Ig-like domain family protein	17	15	84	9	centroid_6608	18.904333	1.3744E-05	centroid_6608	1.8983E-05
centroid_14352	putative predicted protein	16	17	85	7	centroid_1435	27.417318	1.63956E-07	centroid_1435	3.4181E-07
centroid_13448	hypothetical protein	16	16	85	8	centroid_1344	23.6998132	1.12593E-06	centroid_1344	2.0798E-06
centroid_5805	phage minor tail family protein	16	16	85	8	centroid_5805	23.6998132	1.12593E-06	centroid_5805	2.0798E-06
centroid_5803	phage tail assembly protein T	16	15	85	9	centroid_5803	20.2039788	6.9608E-06	centroid_5803	1.1059E-05
centroid_7253	phage minor tail protein G	16	15	85	9	centroid_7253	20.2039788	6.9608E-06	centroid_7253	1.1059E-05
centroid_13083	conserved hypothetical protein	15	14	86	10	centroid_1308	18.2093148	1.97909E-05	centroid_1308	3.06E-05
centroid_15812	hypothetical protein	14	18	87	6	centroid_1581	34.9152587	3.44371E-09	centroid_1581	1.1745E-08
centroid_11244	conserved hypothetical family protein	14	15	87	9	centroid_1124	23.0900898	1.54585E-06	centroid_1124	3.4235E-06
centroid_8844	ive escherichia coli IMT2125 genomic chromosome, IMT	13	22	88	2	centroid_8844	55.8773418	7.71365E-14	centroid_8844	1.8602E-13
centroid_5804	phage tail tape measure protein, lambda family	13	15	88	9	centroid_5804	24.6966593	6.71004E-07	centroid_5804	1.8078E-06
centroid_5679	protein HokA	12	22	89	2	centroid_5679	58.376325	2.1648E-14	centroid_5679	7.0567E-14
centroid_7086	transposase family protein	12	19	89	5	centroid_7086	43.5368392	4.16053E-11	centroid_7086	2.4884E-10
centroid_7274	type VII secretion system (T7SS), usher family protein	12	19	89	5	centroid_7274	43.5368392	4.16053E-11	centroid_7274	2.4884E-10
centroid_15857	aldehyde dehydrogenase family protein	12	17	89	7	centroid_1585	34.5881584	4.07377E-09	centroid_1585	2.025E-08
centroid_6419	homoprotocatechuate degradation operon regulator, HpaR	12	17	89	7	centroid_6419	34.5881584	4.07377E-09	centroid_6419	2.025E-08
centroid_12523	ydroxyphenylacetate 3-hydroxylase C terminal family pro	12	16	89	8	centroid_1252	30.406885	3.50283E-08	centroid_1252	1.4556E-07
centroid_12524	ydroxyphenylacetate 3-hydroxylase N terminal family pro	12	16	89	8	centroid_1252	30.406885	3.50283E-08	centroid_1252	1.4556E-07
centroid_6420	otocatechuate catabolism bifunctional isomerase/decarb	12	16	89	8	centroid_6420	30.406885	3.50283E-08	centroid_6420	1.4556E-07
centroid_6421	boxymethyl-2-hydroxymuconate semialdehyde dehydrog	12	16	89	8	centroid_6421	30.406885	3.50283E-08	centroid_6421	1.4556E-07
centroid_6422	3,4-dihydroxyphenylacetate 2,3-dioxygenase	12	16	89	8	centroid_6422	30.406885	3.50283E-08	centroid_6422	1.4556E-07
centroid_6424	2-oxo-hepta-3-ene-1,7-dioic acid hydratase	12	16	89	8	centroid_6424	30.406885	3.50283E-08	centroid_6424	1.4556E-07
centroid_6425	2,4-dihydroxyhept-2-ene-1,7-dioic acid aldolase	12	16	89	8	centroid_6425	30.406885	3.50283E-08	centroid_6425	1.4556E-07
centroid_6426	4-hydroxyphenylacetate permease	12	16	89	8	centroid_6426	30.406885	3.50283E-08	centroid_6426	1.4556E-07
centroid_6428	lroxyphenylacetate 3-monooxygenase, oxygenase comp	12	16	89	8	centroid_6428	30.406885	3.50283E-08	centroid_6428	1.4556E-07
centroid_6429	droxyphenylacetate 3-monooxygenase, reductase comp	12	16	89	8	centroid_6429	30.406885	3.50283E-08	centroid_6429	1.4556E-07
centroid_6427	-hydroxyphenylacetate catabolism regulatory protein Hpa	12	14	89	10	centroid_6427	22.6591619	1.93436E-06	centroid_6427	5.1191E-06
centroid_10754	phage integrase family protein	12	12	89	12	centroid_1075	15.7890262	7.0812E-05	centroid_1075	0.00011372
centroid_10977	conserved hypothetical protein	12	12	89	12	centroid_1097	15.7890262	7.0812E-05	centroid_1097	0.00011372
centroid_13431	conserved hypothetical protein	12	12	89	12	centroid_1343	15.7890262	7.0812E-05	centroid_1343	0.00011372
centroid_13450	conserved hypothetical protein	12	12	89	12	centroid_1345	15.7890262	7.0812E-05	centroid_1345	0.00011372
centroid_15117	conserved hypothetical protein	12	12	89	12	centroid_1511	15.7890262	7.0812E-05	centroid_1511	0.00011372
centroid_7239	putative excisionase	12	12	89	12	centroid_7239	15.7890262	7.0812E-05	centroid_7239	0.00011372
centroid_7761	putative iS621 protein	11	18	90	6	centroid_7761	41.205452	1.3704E-10	centroid_7761	1.0354E-09
centroid_12632	mu-like prophage major head subunit gpT family protein	11	15	90	9	centroid_1263	28.2987483	1.03963E-07	centroid_1263	4.452E-07
centroid_15492	putative predicted protein	11	15	90	9	centroid_1549	28.2987483	1.03963E-07	centroid_1549	4.452E-07
centroid_10961	trbC domain protein	11	13	90	11	centroid_1096	20.7032707	5.36243E-06	centroid_1096	1.3584E-05
centroid_5798	conserved hypothetical protein	11	13	90	11	centroid_5798	20.7032707	5.36243E-06	centroid_5798	1.3584E-05
centroid_5799	TP-binding sugar transporter from pro-phage family prote	11	13	90	11	centroid_5799	20.7032707	5.36243E-06	centroid_5799	1.3584E-05
centroid_5796	phage portal protein, lambda family	11	12	90	12	centroid_5796	17.2356045	3.3019E-05	centroid_5796	6.3651E-05
centroid_7233	killing protein KilR	11	12	90	12	centroid_7233	17.2356045	3.3019E-05	centroid_7233	6.3651E-05
centroid_13516	putative tail length tape measure domain protein	10	17	91	7	centroid_1351	38.9936116	4.25195E-10	centroid_1351	3.7835E-09
centroid_7317	conserved hypothetical protein	10	17	91	7	centroid_7317	38.9936116	4.25195E-10	centroid_7317	3.7835E-09
centroid_11987	conserved hypothetical protein	10	13	91	11	centroid_1198	22.4451248	2.16234E-06	centroid_1198	6.9087E-06
centroid_7238	double-strand break reduction protein	10	12	91	12	centroid_7238	18.8244761	1.43316E-05	centroid_7238	3.4046E-05
centroid_7240	phage integrase family protein	10	12	91	12	centroid_7240	18.8244761	1.43316E-05	centroid_7240	3.4046E-05
centroid_18615	putative transposase	9	16	92	8	centroid_1861	36.8998814	1.24354E-09	centroid_1861	1.2366E-08

centroid_6792	phage tail tape measure protein, lambda family	9	15	92	9	centroid_679	32.5261772	1.17598E-08	centroid_679	8.9627E-08
centroid_10033	conserved hypothetical protein	9	12	92	12	centroid_1003	20.5756426	5.73208E-06	centroid_1003	1.7292E-05
centroid_13111	conserved hypothetical protein	9	12	92	12	centroid_1311	20.5756426	5.73208E-06	centroid_1311	1.7292E-05
centroid_13445	integrase core domain protein	9	12	92	12	centroid_1344	20.5756426	5.73208E-06	centroid_1344	1.7292E-05
centroid_6100	integrase core domain protein	9	12	92	12	centroid_610	20.5756426	5.73208E-06	centroid_610	1.7292E-05
centroid_9763	integrase core domain protein	9	12	92	12	centroid_976	20.5756426	5.73208E-06	centroid_976	1.7292E-05
centroid_5828	putative cytoplasmic protein	8	19	93	5	centroid_582	53.9952003	2.0098E-13	centroid_582	4.7845E-12
centroid_5911	helix-turn-helix domain protein	8	18	93	6	centroid_591	48.9738928	2.59392E-12	centroid_591	5.5868E-11
centroid_8815	putative permease family protein	8	18	93	6	centroid_881	48.9738928	2.59392E-12	centroid_881	5.5868E-11
centroid_13157	bacterial Ig-like domain family protein	8	13	93	11	centroid_1315	26.4549222	2.6976E-07	centroid_1315	1.509E-06
centroid_13292	conserved hypothetical protein	8	13	93	11	centroid_1329	26.4549222	2.6976E-07	centroid_1329	1.509E-06
centroid_5797	clp protease family protein	8	12	93	12	centroid_579	22.5131191	2.08713E-06	centroid_579	8.2732E-06
centroid_7235	putative protein RacC	8	12	93	12	centroid_723	22.5131191	2.08713E-06	centroid_723	8.2732E-06
centroid_7396	outer membrane porin protein OmpD	8	11	93	13	centroid_739	18.7833333	1.46441E-05	centroid_739	4.1053E-05
centroid_14508	RHS repeat-associated core domain protein	8	10	93	14	centroid_1450	15.2823468	9.25777E-05	centroid_1450	0.00018474
centroid_17334	bacterial Ig-like domain family protein	8	10	93	14	centroid_1733	15.2823468	9.25777E-05	centroid_1733	0.00018474
centroid_18330	RHS repeat-associated core domain protein	8	10	93	14	centroid_1833	15.2823468	9.25777E-05	centroid_1833	0.00018474
centroid_6025	outer membrane lipoprotein blc	8	10	93	14	centroid_602	15.2823468	9.25777E-05	centroid_602	0.00018474
centroid_6111	transposase family protein	8	10	93	14	centroid_611	15.2823468	9.25777E-05	centroid_611	0.00018474
centroid_6607	bacterial Ig-like domain family protein	8	10	93	14	centroid_660	15.2823468	9.25777E-05	centroid_660	0.00018474
centroid_14737	conserved hypothetical protein	7	22	94	2	centroid_1473	73.4630217	1.02539E-17	centroid_1473	2.2251E-16
centroid_7211	ankyrin repeat family protein	7	19	94	5	centroid_721	57.117736	4.10487E-14	centroid_721	1.4887E-12
centroid_5800	prophage minor tail Z family protein	7	13	94	11	centroid_580	28.774906	8.12977E-08	centroid_580	6.3618E-07
centroid_9788	conserved hypothetical protein	7	13	94	11	centroid_978	28.774906	8.12977E-08	centroid_978	6.3618E-07
centroid_7687	conserved hypothetical protein	7	11	94	13	centroid_768	20.7577289	5.21208E-06	centroid_768	1.9423E-05
centroid_16938	bacterial Ig-like domain family protein	7	10	94	14	centroid_1693	17.0662051	3.6099E-05	centroid_1693	9.2899E-05
centroid_7523	plasmid segregation protein ParM	7	10	94	14	centroid_752	17.0662051	3.6099E-05	centroid_752	9.2899E-05
centroid_7524	plasmid stability family protein	7	10	94	14	centroid_752	17.0662051	3.6099E-05	centroid_752	9.2899E-05
centroid_9491	corbate-specific phosphotransferase enzyme IIA compon	7	10	94	14	centroid_949	17.0662051	3.6099E-05	centroid_949	9.2899E-05
centroid_13094	CRISPR-associated endonuclease Cas3-HD	6	20	95	4	centroid_1309	65.8876435	4.77375E-16	centroid_1309	2.7245E-14
centroid_14390	CRISPR-associated endonuclease Cas3-HD	6	20	95	4	centroid_1439	65.8876435	4.77375E-16	centroid_1439	2.7245E-14
centroid_5674	SPR-associated endoribonuclease Cas2, subtype I-E/EC	6	20	95	4	centroid_567	65.8876435	4.77375E-16	centroid_567	2.7245E-14
centroid_13093	CRISPR-associated helicase Cas3	6	19	95	5	centroid_1309	60.4920844	7.3875E-15	centroid_1309	4.2072E-13
centroid_15847	conserved hypothetical protein	6	19	95	5	centroid_1584	60.4920844	7.3875E-15	centroid_1584	4.2072E-13
centroid_5675	SPR-associated protein Cas6/Cse3/CasE, subtype I-E/E	6	19	95	5	centroid_567	60.4920844	7.3875E-15	centroid_567	4.2072E-13
centroid_5676	RISPR-associated protein Cas5/CasD, subtype I-E/ECO	6	19	95	5	centroid_567	60.4920844	7.3875E-15	centroid_567	4.2072E-13
centroid_5678	CRISPR-associated helicase Cas3	6	19	95	5	centroid_567	60.4920844	7.3875E-15	centroid_567	4.2072E-13
centroid_6108	transposase IS116/IS110/IS902 family protein	6	19	95	5	centroid_610	60.4920844	7.3875E-15	centroid_610	4.2072E-13
centroid_7349	SPR-associated protein Cas7/Cse4/CasC, subtype I-E/E	6	19	95	5	centroid_734	60.4920844	7.3875E-15	centroid_734	4.2072E-13
centroid_7675	CRISPR-associated endonuclease Cas1	6	19	95	5	centroid_767	60.4920844	7.3875E-15	centroid_767	4.2072E-13
centroid_8842	SPR-associated protein Cas7/Cse4/CasC, subtype I-E/E	6	19	95	5	centroid_884	60.4920844	7.3875E-15	centroid_884	4.2072E-13
centroid_12532	conserved hypothetical family protein	6	17	95	7	centroid_1253	50.1522993	1.42264E-12	centroid_1253	5.9339E-11
centroid_18577	conserved hypothetical protein	6	17	95	7	centroid_1857	50.1522993	1.42264E-12	centroid_1857	5.9339E-11
centroid_8547	conserved hypothetical protein	6	17	95	7	centroid_854	50.1522993	1.42264E-12	centroid_854	5.9339E-11
centroid_14626	conserved hypothetical protein	6	15	95	9	centroid_1462	40.4270654	2.04094E-10	centroid_1462	4.7981E-09
centroid_5677	CRISPR type I-E/ECOLI-associated protein CasA/Cse1	6	14	95	10	centroid_567	35.8040687	2.18192E-09	centroid_567	3.6225E-08
centroid_7350	CRISPR type I-E/ECOLI-associated protein CasB/Cse2	6	14	95	10	centroid_735	35.8040687	2.18192E-09	centroid_735	3.6225E-08
centroid_8843	CT1975-like family protein	6	14	95	10	centroid_884	35.8040687	2.18192E-09	centroid_884	3.6225E-08
centroid_9176	putative yfdA protein	6	13	95	11	centroid_917	31.3487889	2.15593E-08	centroid_917	2.4621E-07
centroid_12380	integrase core domain protein	6	11	95	13	centroid_1238	22.9785099	1.63823E-06	centroid_1238	8.4793E-06
centroid_7871	integrase core domain protein	6	10	95	14	centroid_787	19.0898572	1.24706E-05	centroid_787	4.324E-05
centroid_10820	zinc-binding dehydrogenase family protein	6	9	95	15	centroid_1082	15.4236127	8.59081E-05	centroid_1082	0.00020111
centroid_12694	bacteriophage lambda tail assembly I family protein	6	9	95	15	centroid_1269	15.4236127	8.59081E-05	centroid_1269	0.00020111
centroid_13104	conserved hypothetical protein	6	9	95	15	centroid_1310	15.4236127	8.59081E-05	centroid_1310	0.00020111
centroid_14715	conserved hypothetical protein	6	9	95	15	centroid_1471	15.4236127	8.59081E-05	centroid_1471	0.00020111
centroid_7234	prokaryotic metallothionein family protein	6	9	95	15	centroid_723	15.4236127	8.59081E-05	centroid_723	0.00020111
centroid_8474	putative sor-operon regulator	6	9	95	15	centroid_847	15.4236127	8.59081E-05	centroid_847	0.00020111
centroid_8475	short chain dehydrogenase family protein	6	9	95	15	centroid_847	15.4236127	8.59081E-05	centroid_847	0.00020111
centroid_8476	m, mannose/fructose/sorbose family, IIA component dom	6	9	95	15	centroid_847	15.4236127	8.59081E-05	centroid_847	0.00020111
centroid_8477	rbose-specific phosphotransferase enzyme IIB compone	6	9	95	15	centroid_847	15.4236127	8.59081E-05	centroid_847	0.00020111
centroid_8478	stem, mannose/fructose/sorbose , IIC component family	6	9	95	15	centroid_847	15.4236127	8.59081E-05	centroid_847	0.00020111
centroid_8479	stem, mannose/fructose/sorbose , IID component family	6	9	95	15	centroid_847	15.4236127	8.59081E-05	centroid_847	0.00020111
centroid_8480	zinc-binding dehydrogenase family protein	6	9	95	15	centroid_848	15.4236127	8.59081E-05	centroid_848	0.00020111
centroid_9043	putative sugar-binding domain protein	6	9	95	15	centroid_904	15.4236127	8.59081E-05	centroid_904	0.00020111
centroid_9044	putative sugar-binding domain protein	6	9	95	15	centroid_904	15.4236127	8.59081E-05	centroid_904	0.00020111
centroid_10235	fibronectin type III family protein	5	11	96	13	centroid_1023	25.4914614	4.44345E-07	centroid_1023	3.3566E-06
centroid_13480	fibronectin type III family protein	5	11	96	13	centroid_1348	25.4914614	4.44345E-07	centroid_1348	3.3566E-06
centroid_11298	phage late control gene D family protein	5	10	96	14	centroid_1129	21.4007729	3.72621E-06	centroid_1129	1.8313E-05
centroid_15188	conserved hypothetical protein	5	9	96	15	centroid_1518	17.514495	2.85126E-05	centroid_1518	9.1433E-05
centroid_5807	bacteriophage lambda tail assembly I family protein	5	9	96	15	centroid_580	17.514495	2.85126E-05	centroid_580	9.1433E-05
centroid_5994	conserved hypothetical protein	5	9	96	15	centroid_599	17.514495	2.85126E-05	centroid_599	9.1433E-05
centroid_6796	conserved hypothetical protein	5	9	96	15	centroid_679	17.514495	2.85126E-05	centroid_679	9.1433E-05
centroid_5814	putative membrane protein	4	17	97	7	centroid_581	57.3506543	3.64642E-14	centroid_581	4.0128E-12
centroid_12688	phage tail tape measure protein, lambda family	4	13	97	11	centroid_1268	37.4362721	9.44492E-10	centroid_1268	2.634E-08
centroid_10919	type IV/VI secretion system , DotU family domain protein	4	10	97	14	centroid_1091	24.0600036	9.33799E-07	centroid_1091	6.8879E-06
centroid_12120	conserved hypothetical protein	4	10	97	14	centroid_1212	24.0600036	9.33799E-07	centroid_1212	6.8879E-06
centroid_12121	conserved hypothetical protein	4	10	97	14	centroid_1212	24.0600036	9.33799E-07	centroid_1212	6.8879E-06
centroid_14008	phage major tail tube protein	4	10	97	14	centroid_1400	24.0600036	9.33799E-07	centroid_1400	6.8879E-06
centroid_14009	phage late control gene D family protein	4	10	97	14	centroid_1400	24.0600036	9.33799E-07	centroid_1400	6.8879E-06
centroid_16551	conserved hypothetical protein	4	10	97	14	centroid_1655	24.0600036	9.33799E-07	centroid_1655	6.8879E-06
centroid_16552	imcF-related N-terminal domain protein	4	10	97	14	centroid_1655	24.0600036	9.33799E-07	centroid_1655	6.8879E-06
centroid_7369	mu-like prophage FluMu gp41 family protein	4	10	97	14	centroid_736	24.0600036	9.33799E-07	centroid_736	6.8879E-06
centroid_7370	phage major tail tube protein	4	10	97	14	centroid_737	24.0600036	9.33799E-07	centroid_737	6.8879E-06
centroid_7371	phage tail sheath family protein	4	10	97	14	centroid_737	24.0600036	9.33799E-07	centroid_737	6.8879E-06
centroid_7411	conserved hypothetical protein	4	10	97	14	centroid_741	24.0600036	9.33799E-07	centroid_741	6.8879E-06
centroid_7412	conserved hypothetical protein	4	10	97	14	centroid_741	24.0600036	9.33799E-07	centroid_741	6.8879E-06
centroid_7414	gene 25-like lysozyme family protein	4	10	97	14	centroid_741	24.0600036	9.33799E-07	centroid_741	6.8879E-06
centroid_7415	conserved hypothetical protein	4	10	97	14	centroid_741	24.0600036	9.33799E-07	centroid_741	6.8879E-06
centroid_7416	conserved hypothetical protein	4	10	97	14	centroid_741	24.0600036	9.33799E-07	centroid_741	6.8879E-06
centroid_7417	conserved hypothetical protein	4	10	97	14	centroid_741	24.0600036	9.33799E-07	centroid_741	6.8879E-06
centroid_7418	PAAR motif family protein	4	10	97	14	centroid_741	24.0600036	9.33799E-07	centroid_741	6.8879E-06
centroid_7419	impA-related N-terminal family protein	4	10	97	14	centroid_741	24.0600036	9.33799E-07	centroid_741	6.8879E-06
centroid_7420	type VI secretion lipofamily protein	4	10	97	14	centroid_742	24.0600036	9.33799E-07	centroid_742	6.8879E-06
centroid_7422	type VI secretion ATPase, ClpV1 family	4	10	97	14	centroid_742	24.0600036	9.33799E-07	centroid_742	6.8879E-06
centroid_9144	type IV/VI secretion system , DotU family domain protein	4	10	97	14	centroid_914	24.0600036	9.33799E-07	centroid_914	6.8879E-06
centroid_5921	traG-like , N-terminal region family protein	4	9	97	15	centroid_592	19.9488294	7.95426E-06	centroid_592	3.7083E-05
centroid_9327	5-carboxymethyl-2-hydroxymuconate Delta-isomerase	4	9	97	15	centroid_932	19.9488294	7.95426E-06	centroid_932	3.7083E-05
centroid_13451	conserved hypothetical protein	4	8	97	16	centroid_1345	16.0426467	6.19317E-05	centroid_1345	0.00018341
centroid_6051	type VII secretion system (T7SS), usher family protein	4	8	97	16	centroid_605	16.0426467	6.19317E-05	centroid_605	0.00018341
centroid_7372	resolvase, N terminal domain protein	4	8	97	16	centroid_737	16.0426467	6.19317E-05	centroid_737	0.00018341
centroid_9242	resolvase, N terminal domain protein	4	8	97	16	centroid_924	16.0426467	6.19317E-05	centroid_924	0.00018341
centroid_14505	conserved hypothetical protein	3	23	98	1	centroid_1450	95.953751	1.17599E-22	centroid_1450	8.0059E-21
centroid_14504	hypothetical protein	3	20	98	4	centroid_1450	78.1452281	9.57366E-19	centroid_1450	2.3514E-16
centroid_18514	putative transcriptional activator Ogr/delta	3	10	98	14	centroid_1851	27.1474051	1.88518E-07	centroid_1851	2.2187E-06
centroid_18607	hypothetical protein	3	10	98	14	centroid_1860	27.1474051	1.88518E-07	centroid_1860	2.2187E-06
centroid_7366	phage late control gene D family protein	3	10	98	14	centroid_736	27.1474051	1.88518E-07	centroid_736	2.2187E-06
centroid_7367	phage P2 GpU family protein	3	10	98	14	centroid_736	27.1474051	1.88518E-07	centroid_736	2.2187E-06

centroid_7377	phage tail protein I	3	10	98	14	centroid_7377	27.1474051	1.88518E-07	centroid_7377	2.2187E-06
centroid_7413	conserved hypothetical protein	3	10	98	14	centroid_7413	27.1474051	1.88518E-07	centroid_7413	2.2187E-06
centroid_7421	hypothetical protein	3	10	98	14	centroid_7421	27.1474051	1.88518E-07	centroid_7421	2.2187E-06
centroid_11094	hypothetical protein	3	9	98	15	centroid_1109	22.8118524	1.7866E-06	centroid_1109	1.2946E-05
centroid_11095	putative phage immunity repressor protein	3	9	98	15	centroid_1109	22.8118524	1.7866E-06	centroid_1109	1.2946E-05
centroid_12122	hemolysin expression-modulating protein Hha	3	9	98	15	centroid_1212	22.8118524	1.7866E-06	centroid_1212	1.2946E-05
centroid_13473	hypothetical protein	3	9	98	15	centroid_1347	22.8118524	1.7866E-06	centroid_1347	1.2946E-05
centroid_6491	ogr/Delta-like zinc finger family protein	3	9	98	15	centroid_6491	22.8118524	1.7866E-06	centroid_6491	1.2946E-05
centroid_6492	putative glyco3, capsid size determination protein Sid	3	9	98	15	centroid_6492	22.8118524	1.7866E-06	centroid_6492	1.2946E-05
centroid_6495	putative derepression protein	3	9	98	15	centroid_6495	22.8118524	1.7866E-06	centroid_6495	1.2946E-05
centroid_6496	putative predicted protein	3	9	98	15	centroid_6496	22.8118524	1.7866E-06	centroid_6496	1.2946E-05
centroid_6497	conserved hypothetical protein	3	9	98	15	centroid_6497	22.8118524	1.7866E-06	centroid_6497	1.2946E-05
centroid_6498	putative P4-specific DNA primase	3	9	98	15	centroid_6498	22.8118524	1.7866E-06	centroid_6498	1.2946E-05
centroid_7429	avrPphF-ORF-2 family protein	3	9	98	15	centroid_7429	22.8118524	1.7866E-06	centroid_7429	1.2946E-05
centroid_15850	putative conjugal transfer pilus assembly protein	3	8	98	16	centroid_1585	18.6532736	1.56778E-05	centroid_1585	6.9768E-05
centroid_18640	transposase family protein	3	8	98	16	centroid_1864	18.6532736	1.56778E-05	centroid_1864	6.9768E-05
centroid_7236	exodeoxyribonuclease 8	3	8	98	16	centroid_7236	18.6532736	1.56778E-05	centroid_7236	6.9768E-05
centroid_5605	putative membrane protein	2	19	99	5	centroid_5605	77.2256886	1.52497E-18	centroid_5605	6.0291E-16
centroid_5833	haemagglutinin family protein	2	19	99	5	centroid_5833	77.2256886	1.52497E-18	centroid_5833	6.0291E-16
centroid_11144	rhs core with extension domain protein	2	15	99	9	centroid_1114	55.4049041	9.80914E-14	centroid_1114	1.6711E-11
centroid_12963	putative dsORF-e4	2	15	99	9	centroid_1296	55.4049041	9.80914E-14	centroid_1296	1.6711E-11
centroid_12964	conserved hypothetical protein	2	15	99	9	centroid_1296	55.4049041	9.80914E-14	centroid_1296	1.6711E-11
centroid_18642	conserved hypothetical protein	2	15	99	9	centroid_1864	55.4049041	9.80914E-14	centroid_1864	1.6711E-11
centroid_6089	putative rHS protein	2	15	99	9	centroid_6089	55.4049041	9.80914E-14	centroid_6089	1.6711E-11
centroid_6090	conserved hypothetical protein	2	15	99	9	centroid_6090	55.4049041	9.80914E-14	centroid_6090	1.6711E-11
centroid_15002	DKNYY family protein	2	12	99	12	centroid_1500	40.2620038	2.22087E-10	centroid_1500	1.1355E-08
centroid_5815	conserved hypothetical protein	2	12	99	12	centroid_5815	40.2620038	2.22087E-10	centroid_5815	1.1355E-08
centroid_14329	conserved hypothetical family protein	2	11	99	13	centroid_1432	35.4527729	2.6131E-09	centroid_1432	8.4124E-08
centroid_11002	putative transposase	2	9	99	15	centroid_1100	26.2198198	3.04679E-07	centroid_1100	3.669E-06
centroid_11785	putative transposase	2	9	99	15	centroid_1178	26.2198198	3.04679E-07	centroid_1178	3.669E-06
centroid_6082	putative transposase	2	9	99	15	centroid_6082	26.2198198	3.04679E-07	centroid_6082	3.669E-06
centroid_10179	CFA/I fimbrial subunit D	2	7	99	17	centroid_1017	17.5750996	2.76181E-05	centroid_1017	0.00011925
centroid_11055	H+ symporter family protein	1	23	100	1	centroid_1105	106.410035	5.99534E-25	centroid_1105	7.533E-23
centroid_11056	lacY proton/sugar symporter family protein	1	23	100	1	centroid_1105	106.410035	5.99534E-25	centroid_1105	7.533E-23
centroid_12882	H+ symporter family protein	1	23	100	1	centroid_1288	106.410035	5.99534E-25	centroid_1288	7.533E-23
centroid_13367	type IV leader peptidase family protein	1	23	100	1	centroid_1336	106.410035	5.99534E-25	centroid_1336	7.533E-23
centroid_5854	H+ symporter family protein	1	23	100	1	centroid_5854	106.410035	5.99534E-25	centroid_5854	7.533E-23
centroid_5855	pfkB carbohydrate kinase family protein	1	23	100	1	centroid_5855	106.410035	5.99534E-25	centroid_5855	7.533E-23
centroid_5856	sucrose-6-phosphate hydrolase family protein	1	23	100	1	centroid_5856	106.410035	5.99534E-25	centroid_5856	7.533E-23
centroid_5857	lasmic binding and sugar binding domain of LacI family pr	1	23	100	1	centroid_5857	106.410035	5.99534E-25	centroid_5857	7.533E-23
centroid_5955	BFD-like [2Fe-2S] binding domain protein	1	23	100	1	centroid_5955	106.410035	5.99534E-25	centroid_5955	7.533E-23
centroid_5956	bacterioferritin	1	23	100	1	centroid_5956	106.410035	5.99534E-25	centroid_5956	7.533E-23
centroid_9104	H+ symporter family protein	1	23	100	1	centroid_9104	106.410035	5.99534E-25	centroid_9104	7.533E-23
centroid_12883	LPXTG cell wall anchor domain protein	1	22	100	2	centroid_1288	100.241727	1.34888E-23	centroid_1288	3.6834E-21
centroid_13024	hypothetical protein	1	22	100	2	centroid_1302	100.241727	1.34888E-23	centroid_1302	3.6834E-21
centroid_14944	putative ybl55 protein	1	20	100	4	centroid_1494	88.2699979	5.7103E-21	centroid_1494	3.0052E-18
centroid_17372	fimbrial family protein	1	20	100	4	centroid_1737	88.2699979	5.7103E-21	centroid_1737	3.0052E-18
centroid_5577	tRNA(fMet)-specific endonuclease VapC	1	20	100	4	centroid_5577	88.2699979	5.7103E-21	centroid_5577	3.0052E-18
centroid_5578	antitoxin VapB	1	20	100	4	centroid_5578	88.2699979	5.7103E-21	centroid_5578	3.0052E-18
centroid_5904	SPFH domain / Band 7 family protein	1	20	100	4	centroid_5904	88.2699979	5.7103E-21	centroid_5904	3.0052E-18
centroid_7287	antitoxin VapB	1	20	100	4	centroid_7287	88.2699979	5.7103E-21	centroid_7287	3.0052E-18
centroid_13023	fimbrial family protein	1	19	100	5	centroid_1302	82.46052	1.07802E-19	centroid_1302	6.0139E-17
centroid_16055	ı-negative pili assembly chaperone, N-terminal domain pr	1	19	100	5	centroid_1605	82.46052	1.07802E-19	centroid_1605	6.0139E-17
centroid_16056	ı-negative pili assembly chaperone, C-terminal domain pr	1	19	100	5	centroid_1605	82.46052	1.07802E-19	centroid_1605	6.0139E-17
centroid_18522	type VII secretion system (T7SS), usher family protein	1	19	100	5	centroid_1852	82.46052	1.07802E-19	centroid_1852	6.0139E-17
centroid_18523	type VII secretion system (T7SS), usher family protein	1	19	100	5	centroid_1852	82.46052	1.07802E-19	centroid_1852	6.0139E-17
centroid_5575	inner membrane protein YmfA	1	19	100	5	centroid_5575	82.46052	1.07802E-19	centroid_5575	6.0139E-17
centroid_5621	cblD like pilus biogenesis initiator family protein	1	19	100	5	centroid_5621	82.46052	1.07802E-19	centroid_5621	6.0139E-17
centroid_5622	fimbrial family protein	1	19	100	5	centroid_5622	82.46052	1.07802E-19	centroid_5622	6.0139E-17
centroid_5684	fimbrial family protein	1	19	100	5	centroid_5684	82.46052	1.07802E-19	centroid_5684	6.0139E-17
centroid_5685	ı-negative pili assembly chaperone, C-terminal domain pr	1	19	100	5	centroid_5685	82.46052	1.07802E-19	centroid_5685	6.0139E-17
centroid_5686	type VII secretion system (T7SS), usher family protein	1	19	100	5	centroid_5686	82.46052	1.07802E-19	centroid_5686	6.0139E-17
centroid_5687	fimbrial family protein	1	19	100	5	centroid_5687	82.46052	1.07802E-19	centroid_5687	6.0139E-17
centroid_5846	conserved hypothetical protein	1	19	100	5	centroid_5846	82.46052	1.07802E-19	centroid_5846	6.0139E-17
centroid_5905	putative nucleotidyltransferase family protein	1	19	100	5	centroid_5905	82.46052	1.07802E-19	centroid_5905	6.0139E-17
centroid_5906	zeta toxin family protein	1	19	100	5	centroid_5906	82.46052	1.07802E-19	centroid_5906	6.0139E-17
centroid_5847	fimbrial family protein	1	17	100	7	centroid_5847	71.1807764	3.25948E-17	centroid_5847	1.4636E-14
centroid_11150	putative yhaC	1	16	100	8	centroid_1115	65.7057964	5.23517E-16	centroid_1115	1.8677E-13
centroid_11165	hypothetical protein	1	16	100	8	centroid_1116	65.7057964	5.23517E-16	centroid_1116	1.8677E-13
centroid_5573	conserved hypothetical protein	1	16	100	8	centroid_5573	65.7057964	5.23517E-16	centroid_5573	1.8677E-13
centroid_5899	L-galactonate transporter	1	16	100	8	centroid_5899	65.7057964	5.23517E-16	centroid_5899	1.8677E-13
centroid_5900	racemase / muconate lactonizing enzyme, N-terminal dor	1	15	100	9	centroid_5900	60.3380689	7.98873E-15	centroid_5900	2.1308E-12
centroid_11109	ı-negative pili assembly chaperone, C-terminal domain pr	1	14	100	10	centroid_1110	55.0760154	1.15957E-13	centroid_1110	2.1997E-11
centroid_5849	conserved hypothetical protein	1	14	100	10	centroid_5849	55.0760154	1.15957E-13	centroid_5849	2.1997E-11
centroid_5850	fimbrial family protein	1	14	100	10	centroid_5850	55.0760154	1.15957E-13	centroid_5850	2.1997E-11
centroid_5851	fimbrial family protein	1	14	100	10	centroid_5851	55.0760154	1.15957E-13	centroid_5851	2.1997E-11
centroid_5852	fimbrial family protein	1	14	100	10	centroid_5852	55.0760154	1.15957E-13	centroid_5852	2.1997E-11
centroid_10046	antitoxin HigA	1	13	100	11	centroid_1004	49.9184956	1.60267E-12	centroid_1004	2.0741E-10
centroid_17830	helix-turn-helix domain protein	1	13	100	11	centroid_1783	49.9184956	1.60267E-12	centroid_1783	2.0741E-10
centroid_17831	conserved hypothetical protein	1	13	100	11	centroid_1783	49.9184956	1.60267E-12	centroid_1783	2.0741E-10
centroid_18198	phage tail tape measure protein, lambda family	1	13	100	11	centroid_1819	49.9184956	1.60267E-12	centroid_1819	2.0741E-10
centroid_7196	acetyltransferase family protein	1	13	100	11	centroid_7196	49.9184956	1.60267E-12	centroid_7196	2.0741E-10
centroid_7197	conserved hypothetical protein	1	13	100	11	centroid_7197	49.9184956	1.60267E-12	centroid_7197	2.0741E-10
centroid_7244	acyl transferase domain protein	1	13	100	11	centroid_7244	49.9184956	1.60267E-12	centroid_7244	2.0741E-10
centroid_7347	antitoxin HigA	1	13	100	11	centroid_7347	49.9184956	1.60267E-12	centroid_7347	2.0741E-10
centroid_11137	putative rhs core protein	1	11	100	13	centroid_1113	39.9155055	2.6519E-10	centroid_1113	1.4465E-08
centroid_11138	conserved hypothetical protein	1	11	100	13	centroid_1113	39.9155055	2.6519E-10	centroid_1113	1.4465E-08
centroid_13461	RHS repeat-associated core domain protein	1	11	100	13	centroid_1346	39.9155055	2.6519E-10	centroid_1346	1.4465E-08
centroid_16123	conserved hypothetical protein	1	11	100	13	centroid_1612	39.9155055	2.6519E-10	centroid_1612	1.4465E-08
centroid_7527	conserved hypothetical protein	1	11	100	13	centroid_7527	39.9155055	2.6519E-10	centroid_7527	1.4465E-08
centroid_11139	putative rhs core protein with extension	1	10	100	14	centroid_1113	35.0714451	3.17827E-09	centroid_1113	1.0817E-07
centroid_13878	gram-negative porin family protein	1	10	100	14	centroid_1387	35.0714451	3.17827E-09	centroid_1387	1.0817E-07
centroid_6106	integrase core domain protein	1	10	100	14	centroid_6106	35.0714451	3.17827E-09	centroid_6106	1.0817E-07
centroid_5887	conserved hypothetical protein	1	9	100	15	centroid_5887	30.3355149	3.63411E-08	centroid_5887	7.556E-07
centroid_5888	plasmid stability family protein	1	9	100	15	centroid_5888	30.3355149	3.63411E-08	centroid_5888	7.556E-07
centroid_11012	conserved hypothetical protein	1	8	100	16	centroid_1101	25.7128117	3.96188E-07	centroid_1101	4.9437E-06
centroid_11013	outer membrane insertion C-terminal signal domain protein	1	8	100	16	centroid_1101	25.7128117	3.96188E-07	centroid_1101	4.9437E-06
centroid_11014	esterase-like activity of phytase family protein	1	8	100	16	centroid_1101	25.7128117	3.96188E-07	centroid_1101	4.9437E-06
centroid_11015	pfkB carbohydrate kinase family protein	1	8	100	16	centroid_1101	25.7128117	3.96188E-07	centroid_1101	4.9437E-06
centroid_11016	SIS domain protein	1	8	100	16	centroid_1101	25.7128117	3.96188E-07	centroid_1101	4.9437E-06
centroid_11017	ADP-ribosylglycohydrolase family protein	1	8	100	16	centroid_1101	25.7128117	3.96188E-07	centroid_1101	4.9437E-06
centroid_11018	bacterial regulatory, gntR family protein	1	8	100	16	centroid_1101	25.7128117	3.96188E-07	centroid_1101	4.9437E-06
centroid_11019	MFS/sugar transport family protein	1	8	100	16	centroid_1101	25.7128117	3.96188E-07	centroid_1101	4.9437E-06
centroid_11020	MFS/sugar transport family protein	1	8	100	16	centroid_1102	25.7128117	3.96188E-07	centroid_1102	4.9437E-06
centroid_13089	esterase-like activity of phytase family protein	1	8	100	16	centroid_1308	25.7128117	3.96188E-07	centroid_1308	4.9437E-06
centroid_13090	(Glycoside-Pentoside-Hexuronide) transporter domain p	1	8	100	16	centroid_1309	25.7128117	3.96188E-07	centroid_1309	4.9437E-06
centroid_13254	ATP-binding region ATPase -containing domain protein	1	8	100	16	centroid_1325	25.7128117	3.96188E-07	centroid_1325	4.9437E-06

centroid_13277	putative 50S ribosomal protein L1	1	8	100	16	centroid_1327	25.7128117	3.96188E-07	centroid_1327 4.9437E-06
centroid_13278	esterase-like activity of phytase family protein	1	8	100	16	centroid_1327	25.7128117	3.96188E-07	centroid_1327 4.9437E-06
centroid_14479	transposase IS66 family protein	1	8	100	16	centroid_1447	25.7128117	3.96188E-07	centroid_1447 4.9437E-06
centroid_15935	ATP-binding region ATPase -containing domain protein	1	8	100	16	centroid_1593	25.7128117	3.96188E-07	centroid_1593 4.9437E-06
centroid_6493	prophage CP4-57 regulatory family protein	1	8	100	16	centroid_649	25.7128117	3.96188E-07	centroid_649 4.9437E-06
centroid_6818	fibronectin type III family protein	1	8	100	16	centroid_681	25.7128117	3.96188E-07	centroid_681 4.9437E-06
centroid_8920	protein TolA	1	8	100	16	centroid_892	25.7128117	3.96188E-07	centroid_892 4.9437E-06
centroid_8921	tolA C-terminal family protein	1	8	100	16	centroid_892	25.7128117	3.96188E-07	centroid_892 4.9437E-06
centroid_13880	gram-negative porin family protein	1	7	100	17	centroid_1388	21.212171	4.11145E-06	centroid_1388 3.0346E-05
centroid_15118	conserved hypothetical protein	1	7	100	17	centroid_1511	21.212171	4.11145E-06	centroid_1511 3.0346E-05
centroid_7368	phage tail tape measure protein, TP901 family, core regior	1	7	100	17	centroid_736	21.212171	4.11145E-06	centroid_736 3.0346E-05
centroid_9473	outer membrane protein N	1	7	100	17	centroid_947	21.212171	4.11145E-06	centroid_947 3.0346E-05
centroid_11096	putative type III secretion protein	1	6	100	18	centroid_1109	16.8487798	4.04793E-05	centroid_1109 0.00017482
centroid_13103	type-1 fimbrial protein, A chain	1	6	100	18	centroid_1310	16.8487798	4.04793E-05	centroid_1310 0.00017482
centroid_13879	conserved hypothetical protein	1	6	100	18	centroid_1387	16.8487798	4.04793E-05	centroid_1387 0.00017482
centroid_18590	hypothetical protein	1	6	100	18	centroid_1859	16.8487798	4.04793E-05	centroid_1859 0.00017482
centroid_5812	conserved hypothetical protein	0	24	101	0	centroid_581	118.637144	1.25752E-27	centroid_581 3.1064E-26
centroid_5883	laminin-binding fimbrial subunit ElfA	0	22	101	2	centroid_588	106.126516	6.91748E-25	centroid_588 1.6318E-22
centroid_14960	outer membrane autotransporter barrel domain protein	0	20	101	4	centroid_1496	94.0939379	3.00899E-22	centroid_1496 1.4849E-19
centroid_8902	conserved hypothetical protein	0	20	101	4	centroid_890	94.0939379	3.00899E-22	centroid_890 1.4849E-19
centroid_13110	outer membrane autotransporter barrel domain protein	0	19	101	5	centroid_1311	88.2486029	5.7724E-21	centroid_1311 3.148E-18
centroid_5572	outer membrane autotransporter barrel domain protein	0	19	101	5	centroid_557	88.2486029	5.7724E-21	centroid_557 3.148E-18
centroid_5620	inner membrane protein YhaI	0	19	101	5	centroid_562	88.2486029	5.7724E-21	centroid_562 3.148E-18
centroid_9422	putative membrane protein	0	19	101	5	centroid_942	88.2486029	5.7724E-21	centroid_942 3.148E-18
centroid_12982	sel1 repeat family protein	0	18	101	6	centroid_1298	82.5130769	1.04973E-19	centroid_1298 5.614E-17
centroid_5581	putative membrane protein	0	18	101	6	centroid_558	82.5130769	1.04973E-19	centroid_558 5.614E-17
centroid_5660	sel1 repeat family protein	0	18	101	6	centroid_566	82.5130769	1.04973E-19	centroid_566 5.614E-17
centroid_5661	acetyltransferase domain protein	0	18	101	6	centroid_566	82.5130769	1.04973E-19	centroid_566 5.614E-17
centroid_5662	conserved hypothetical protein	0	18	101	6	centroid_566	82.5130769	1.04973E-19	centroid_566 5.614E-17
centroid_5819	conserved hypothetical protein	0	18	101	6	centroid_581	82.5130769	1.04973E-19	centroid_581 5.614E-17
centroid_5820	helix-turn-helix family protein	0	18	101	6	centroid_582	82.5130769	1.04973E-19	centroid_582 5.614E-17
centroid_5821	putative transcription elongation factor GreB	0	18	101	6	centroid_582	82.5130769	1.04973E-19	centroid_582 5.614E-17
centroid_5822	alpha/beta hydrolase fold family protein	0	18	101	6	centroid_582	82.5130769	1.04973E-19	centroid_582 5.614E-17
centroid_5891	protein DedA	0	18	101	6	centroid_589	82.5130769	1.04973E-19	centroid_589 5.614E-17
centroid_7345	aldo/keto reductase family protein	0	18	101	6	centroid_734	82.5130769	1.04973E-19	centroid_734 5.614E-17
centroid_17233	lrate uptake ABC transporter 2 (CUT2) family, ATP-bindir	0	17	101	7	centroid_1723	76.884406	1.81263E-18	centroid_1723 8.6616E-16
centroid_17234	ABC transporter family protein	0	17	101	7	centroid_1723	76.884406	1.81263E-18	centroid_1723 8.6616E-16
centroid_5651	ASCH domain protein	0	17	101	7	centroid_565	76.884406	1.81263E-18	centroid_565 8.6616E-16
centroid_5652	putative LACI-type transcriptional regulator	0	17	101	7	centroid_565	76.884406	1.81263E-18	centroid_565 8.6616E-16
centroid_5653	heme ABC exporter, ATP-binding protein CcmA	0	17	101	7	centroid_565	76.884406	1.81263E-18	centroid_565 8.6616E-16
centroid_5654	ain amino acid transport system / permease component	0	17	101	7	centroid_565	76.884406	1.81263E-18	centroid_565 8.6616E-16
centroid_5655	ain amino acid transport system / permease component	0	17	101	7	centroid_565	76.884406	1.81263E-18	centroid_565 8.6616E-16
centroid_5813	putative membrane protein	0	17	101	7	centroid_581	76.884406	1.81263E-18	centroid_581 8.6616E-16
centroid_5873	conserved hypothetical protein	0	17	101	7	centroid_587	76.884406	1.81263E-18	centroid_587 8.6616E-16
centroid_12984	bacterial Ig-like domain family protein	0	16	101	8	centroid_1298	71.3597684	2.9768E-17	centroid_1298 1.1801E-14
centroid_5623	conserved hypothetical protein	0	16	101	8	centroid_562	71.3597684	2.9768E-17	centroid_562 1.1801E-14
centroid_11606	YoaG domain protein	0	15	101	9	centroid_1160	65.9364765	4.65693E-16	centroid_1160 1.4424E-13
centroid_13498	conserved hypothetical protein	0	15	101	9	centroid_1349	65.9364765	4.65693E-16	centroid_1349 1.4424E-13
centroid_15497	outer membrane autotransporter barrel domain protein	0	15	101	9	centroid_1549	65.9364765	4.65693E-16	centroid_1549 1.4424E-13
centroid_15498	nded Signal Peptide of Type V secretion system family pr	0	15	101	9	centroid_1549	65.9364765	4.65693E-16	centroid_1549 1.4424E-13
centroid_5647	outer membrane autotransporter barrel domain protein	0	15	101	9	centroid_564	65.9364765	4.65693E-16	centroid_564 1.4424E-13
centroid_5659	conserved hypothetical protein	0	15	101	9	centroid_565	65.9364765	4.65693E-16	centroid_565 1.4424E-13
centroid_5875	conserved hypothetical protein	0	15	101	9	centroid_587	65.9364765	4.65693E-16	centroid_587 1.4424E-13
centroid_5876	hicB family protein	0	15	101	9	centroid_587	65.9364765	4.65693E-16	centroid_587 1.4424E-13
centroid_8976	conserved hypothetical protein	0	15	101	9	centroid_897	65.9364765	4.65693E-16	centroid_897 1.4424E-13
centroid_18234	lemA family protein	0	14	101	10	centroid_1823	60.6119817	6.95096E-15	centroid_1823 1.6011E-12
centroid_18643	RHS repeat-associated core domain protein	0	14	101	10	centroid_1864	60.6119817	6.95096E-15	centroid_1864 1.6011E-12
centroid_5853	ı-negative pili assembly chaperone, N-terminal domain pr	0	14	101	10	centroid_585	60.6119817	6.95096E-15	centroid_585 1.6011E-12
centroid_5681	lemA family protein	0	13	101	11	centroid_568	55.3838847	9.9146E-14	centroid_568 1.6302E-11
centroid_9079	(Glycoside-Pentoside-Hexuronide) transporter domain p	0	13	101	11	centroid_907	55.3838847	9.9146E-14	centroid_907 1.6302E-11
centroid_9080	helix-turn-helix domain protein	0	13	101	11	centroid_908	55.3838847	9.9146E-14	centroid_908 1.6302E-11
centroid_9576	conserved hypothetical protein	0	13	101	11	centroid_957	55.3838847	9.9146E-14	centroid_957 1.6302E-11
centroid_10088	conserved hypothetical protein	0	12	101	12	centroid_1008	50.2499529	1.35358E-12	centroid_1008 1.5351E-10
centroid_12889	bacterial regulatory , arsR family protein	0	12	101	12	centroid_1288	50.2499529	1.35358E-12	centroid_1288 1.5351E-10
centroid_13466	RHS repeat-associated core domain protein	0	12	101	12	centroid_1346	50.2499529	1.35358E-12	centroid_1346 1.5351E-10
centroid_5604	pfkB carbohydrate kinase family protein	0	12	101	12	centroid_560	50.2499529	1.35358E-12	centroid_560 1.5351E-10
centroid_5816	DKNYY family protein	0	12	101	12	centroid_581	50.2499529	1.35358E-12	centroid_581 1.5351E-10
centroid_7241	alpha/beta hydrolase family protein	0	12	101	12	centroid_724	50.2499529	1.35358E-12	centroid_724 1.5351E-10
centroid_7242	NAD dependent epimerase/dehydratase family protein	0	12	101	12	centroid_724	50.2499529	1.35358E-12	centroid_724 1.5351E-10
centroid_7386	conserved hypothetical protein	0	12	101	12	centroid_738	50.2499529	1.35358E-12	centroid_738 1.5351E-10
centroid_9337	putative carboxymethylenebutenolidase	0	12	101	12	centroid_933	50.2499529	1.35358E-12	centroid_933 1.5351E-10
centroid_9338	X-Pro dipeptidyl-peptidase family protein	0	12	101	12	centroid_933	50.2499529	1.35358E-12	centroid_933 1.5351E-10
centroid_12888	pfkB carbohydrate kinase family protein	0	11	101	13	centroid_1288	45.2081495	1.77166E-11	centroid_1288 1.3461E-09
centroid_11474	penicillin amidase family protein	0	10	101	14	centroid_1147	40.2566836	2.22692E-10	centroid_1147 1.1058E-08
centroid_13225	RHS repeat-associated core domain protein	0	10	101	14	centroid_1322	40.2566836	2.22692E-10	centroid_1322 1.1058E-08
centroid_15860	penicillin amidase family protein	0	10	101	14	centroid_1586	40.2566836	2.22692E-10	centroid_1586 1.1058E-08
centroid_17597	RHS Repeat family protein	0	10	101	14	centroid_1759	40.2566836	2.22692E-10	centroid_1759 1.1058E-08
centroid_18222	ı-negative pili assembly chaperone, C-terminal domain pr	0	10	101	14	centroid_1822	40.2566836	2.22692E-10	centroid_1822 1.1058E-08
centroid_5682	repair family protein	0	10	101	14	centroid_568	40.2566836	2.22692E-10	centroid_568 1.1058E-08
centroid_5683	hypothetical protein	0	10	101	14	centroid_568	40.2566836	2.22692E-10	centroid_568 1.1058E-08
centroid_7531	RHS repeat-associated core domain protein	0	10	101	14	centroid_753	40.2566836	2.22692E-10	centroid_753 1.1058E-08
centroid_9988	penicillin amidase family protein	0	10	101	14	centroid_998	40.2566836	2.22692E-10	centroid_998 1.1058E-08
centroid_12863	putative aTP-binding component of a transport system	0	9	101	15	centroid_1286	35.3940959	2.69302E-09	centroid_1286 8.5512E-08
centroid_14398	conserved hypothetical protein	0	9	101	15	centroid_1439	35.3940959	2.69302E-09	centroid_1439 8.5512E-08
centroid_16094	phage integrase family protein	0	9	101	15	centroid_1609	35.3940959	2.69302E-09	centroid_1609 8.5512E-08
centroid_16130	phage integrase family protein	0	9	101	15	centroid_1613	35.3940959	2.69302E-09	centroid_1613 8.5512E-08
centroid_5658	outer membrane autotransporter barrel domain protein	0	9	101	15	centroid_565	35.3940959	2.69302E-09	centroid_565 8.5512E-08
centroid_5932	putative membrane protein	0	9	101	15	centroid_593	35.3940959	2.69302E-09	centroid_593 8.5512E-08
centroid_7398	RHS repeat-associated core domain protein	0	9	101	15	centroid_739	35.3940959	2.69302E-09	centroid_739 8.5512E-08
centroid_7546	RHS repeat-associated core domain protein	0	9	101	15	centroid_754	35.3940959	2.69302E-09	centroid_754 8.5512E-08
centroid_9014	outer membrane autotransporter barrel domain protein	0	9	101	15	centroid_901	35.3940959	2.69302E-09	centroid_901 8.5512E-08
centroid_9015	conserved hypothetical protein	0	9	101	15	centroid_901	35.3940959	2.69302E-09	centroid_901 8.5512E-08
centroid_9016	conserved hypothetical protein	0	9	101	15	centroid_901	35.3940959	2.69302E-09	centroid_901 8.5512E-08
centroid_9018	phage integrase family protein	0	9	101	15	centroid_901	35.3940959	2.69302E-09	centroid_901 8.5512E-08
centroid_9019	conserved hypothetical protein	0	9	101	15	centroid_901	35.3940959	2.69302E-09	centroid_901 8.5512E-08
centroid_13101	penicillin G acylase	0	8	101	16	centroid_1310	30.6194119	3.13937E-08	centroid_1310 6.2531E-07
centroid_14214	RHS repeat-associated core domain protein	0	8	101	16	centroid_1421	30.6194119	3.13937E-08	centroid_1421 6.2531E-07
centroid_16129	kinase-, DNA gyrase B-, and HSP90-like ATPase family	0	8	101	16	centroid_1612	30.6194119	3.13937E-08	centroid_1612 6.2531E-07
centroid_5869	ROS/MUCR transcriptional regulator family protein	0	8	101	16	centroid_586	30.6194119	3.13937E-08	centroid_586 6.2531E-07
centroid_5931	conserved hypothetical protein	0	8	101	16	centroid_593	30.6194119	3.13937E-08	centroid_593 6.2531E-07
centroid_5933	conserved hypothetical protein	0	8	101	16	centroid_593	30.6194119	3.13937E-08	centroid_593 6.2531E-07
centroid_5974	conserved hypothetical protein	0	8	101	16	centroid_597	30.6194119	3.13937E-08	centroid_597 6.2531E-07
centroid_5975	conserved hypothetical protein	0	8	101	16	centroid_597	30.6194119	3.13937E-08	centroid_597 6.2531E-07
centroid_7389	impA domain family protein	0	8	101	16	centroid_738	30.6194119	3.13937E-08	centroid_738 6.2531E-07
centroid_9017	bacterial regulatory, luxR family protein	0	8	101	16	centroid_901	30.6194119	3.13937E-08	centroid_901 6.2531E-07
centroid_10047	conserved hypothetical protein	0	7	101	17	centroid_1004	25.9324288	3.53579E-07	centroid_1004 4.3404E-06
centroid_10084	RHS repeat-associated core domain protein	0	7	101	17	centroid_1008	25.9324288	3.53579E-07	centroid_1008 4.3404E-06

centroid_13252	fibronectin type III family protein	0	7	101	17	centroid_1325	25.9324288	3.53579E-07	centroid_1325	4.3404E-06
centroid_14463	RHS repeat-associated core domain protein	0	7	101	17	centroid_1446	25.9324288	3.53579E-07	centroid_1446	4.3404E-06
centroid_15179	RHS repeat-associated core domain protein	0	7	101	17	centroid_1517	25.9324288	3.53579E-07	centroid_1517	4.3404E-06
centroid_6837	hypothetical protein	0	7	101	17	centroid_6837	25.9324288	3.53579E-07	centroid_6837	4.3404E-06
centroid_7198	UTRA domain protein	0	7	101	17	centroid_7198	25.9324288	3.53579E-07	centroid_7198	4.3404E-06
centroid_7199	uvate-dependent sugar phosphotransferase system, EIIA	0	7	101	17	centroid_7199	25.9324288	3.53579E-07	centroid_7199	4.3404E-06
centroid_7200	PTS system, Lactose/Cellobiose specific IIB subunit	0	7	101	17	centroid_7200	25.9324288	3.53579E-07	centroid_7200	4.3404E-06
centroid_7201	S system sugar-specific permease component family pro	0	7	101	17	centroid_7201	25.9324288	3.53579E-07	centroid_7201	4.3404E-06
centroid_7202	GY family of carbohydrate kinase, N-terminal domain pro	0	7	101	17	centroid_7202	25.9324288	3.53579E-07	centroid_7202	4.3404E-06
centroid_7203	phosphotransferase system, HPr-related proteins	0	7	101	17	centroid_7203	25.9324288	3.53579E-07	centroid_7203	4.3404E-06
centroid_7204	ketose-bisphosphate aldolase family protein	0	7	101	17	centroid_7204	25.9324288	3.53579E-07	centroid_7204	4.3404E-06
centroid_7381	fil domain protein	0	7	101	17	centroid_7381	25.9324288	3.53579E-07	centroid_7381	4.3404E-06
centroid_7390	conserved hypothetical protein	0	7	101	17	centroid_7390	25.9324288	3.53579E-07	centroid_7390	4.3404E-06
centroid_7391	sigma-54 interaction domain protein	0	7	101	17	centroid_7391	25.9324288	3.53579E-07	centroid_7391	4.3404E-06
centroid_9444	PTS family galactitol porter, component IIC domain protein	0	7	101	17	centroid_9444	25.9324288	3.53579E-07	centroid_9444	4.3404E-06
centroid_9445	S system sugar-specific permease component family pro	0	7	101	17	centroid_9445	25.9324288	3.53579E-07	centroid_9445	4.3404E-06
centroid_9446	conserved hypothetical protein	0	7	101	17	centroid_9446	25.9324288	3.53579E-07	centroid_9446	4.3404E-06
centroid_10065	ash family protein	0	6	101	18	centroid_1006	21.3342943	3.85768E-06	centroid_1006	2.8695E-05
centroid_14364	conserved hypothetical protein	0	6	101	18	centroid_1436	21.3342943	3.85768E-06	centroid_1436	2.8695E-05
centroid_17227	ash family protein	0	6	101	18	centroid_1722	21.3342943	3.85768E-06	centroid_1722	2.8695E-05
centroid_17382	RHS repeat-associated core domain protein	0	6	101	18	centroid_1738	21.3342943	3.85768E-06	centroid_1738	2.8695E-05
centroid_5624	DKNYY family protein	0	6	101	18	centroid_5624	21.3342943	3.85768E-06	centroid_5624	2.8695E-05
centroid_5625	conserved hypothetical protein	0	6	101	18	centroid_5625	21.3342943	3.85768E-06	centroid_5625	2.8695E-05
centroid_5722	conserved hypothetical protein	0	6	101	18	centroid_5722	21.3342943	3.85768E-06	centroid_5722	2.8695E-05
centroid_5892	3-hydroxybutyrate dehydrogenase family protein	0	6	101	18	centroid_5892	21.3342943	3.85768E-06	centroid_5892	2.8695E-05
centroid_5893	citrate transporter family protein	0	6	101	18	centroid_5893	21.3342943	3.85768E-06	centroid_5893	2.8695E-05
centroid_5894	ydroxyacyl-CoA dehydrogenase, NAD binding domain pro	0	6	101	18	centroid_5894	21.3342943	3.85768E-06	centroid_5894	2.8695E-05
centroid_5895	acetyl-CoA C-acetyltransferase family protein	0	6	101	18	centroid_5895	21.3342943	3.85768E-06	centroid_5895	2.8695E-05
centroid_5896	3-oxoacid CoA-transferase, B subunit	0	6	101	18	centroid_5896	21.3342943	3.85768E-06	centroid_5896	2.8695E-05
centroid_5897	3-oxoacid CoA-transferase, A subunit	0	6	101	18	centroid_5897	21.3342943	3.85768E-06	centroid_5897	2.8695E-05
centroid_5898	bacterial regulatory helix-turn-helix , lysR family protein	0	6	101	18	centroid_5898	21.3342943	3.85768E-06	centroid_5898	2.8695E-05
centroid_5934	e kinase-, DNA gyrase B-, and HSP90-like ATPase family	0	6	101	18	centroid_5934	21.3342943	3.85768E-06	centroid_5934	2.8695E-05
centroid_6103	caudovirales tail fibre assembly family protein	0	6	101	18	centroid_6103	21.3342943	3.85768E-06	centroid_6103	2.8695E-05
centroid_8833	tetratricopeptide repeat family protein	0	6	101	18	centroid_8833	21.3342943	3.85768E-06	centroid_8833	2.8695E-05
centroid_8859	conserved hypothetical protein	0	6	101	18	centroid_8859	21.3342943	3.85768E-06	centroid_8859	2.8695E-05
centroid_9418	helix-turn-helix domain protein	0	6	101	18	centroid_9418	21.3342943	3.85768E-06	centroid_9418	2.8695E-05
centroid_9447	conserved hypothetical protein	0	6	101	18	centroid_9447	21.3342943	3.85768E-06	centroid_9447	2.8695E-05
centroid_10142	CRISPR type I-E/ECOLI-associated protein CasB/Cse2	0	5	101	19	centroid_1014	16.8287825	4.09081E-05	centroid_1014	0.00018123
centroid_11585	AAA domain family protein	0	5	101	19	centroid_1158	16.8287825	4.09081E-05	centroid_1158	0.00018123
centroid_11597	hcp domain protein	0	5	101	19	centroid_1159	16.8287825	4.09081E-05	centroid_1159	0.00018123
centroid_11763	fibronectin type III family protein	0	5	101	19	centroid_1176	16.8287825	4.09081E-05	centroid_1176	0.00018123
centroid_12872	prophage CP4-57 integrase	0	5	101	19	centroid_1287	16.8287825	4.09081E-05	centroid_1287	0.00018123
centroid_13004	AAA domain family protein	0	5	101	19	centroid_1300	16.8287825	4.09081E-05	centroid_1300	0.00018123
centroid_13005	C 5-methylcytosine restriction system component family p	0	5	101	19	centroid_1300	16.8287825	4.09081E-05	centroid_1300	0.00018123
centroid_13006	conserved hypothetical protein	0	5	101	19	centroid_1300	16.8287825	4.09081E-05	centroid_1300	0.00018123
centroid_13012	conserved hypothetical protein	0	5	101	19	centroid_1301	16.8287825	4.09081E-05	centroid_1301	0.00018123
centroid_13013	conserved hypothetical protein	0	5	101	19	centroid_1301	16.8287825	4.09081E-05	centroid_1301	0.00018123
centroid_13014	kinase domain protein	0	5	101	19	centroid_1301	16.8287825	4.09081E-05	centroid_1301	0.00018123
centroid_13015	phage integrase family protein	0	5	101	19	centroid_1301	16.8287825	4.09081E-05	centroid_1301	0.00018123
centroid_13470	conserved hypothetical protein	0	5	101	19	centroid_1347	16.8287825	4.09081E-05	centroid_1347	0.00018123
centroid_13509	putative replication protein from bacteriophage origin	0	5	101	19	centroid_1350	16.8287825	4.09081E-05	centroid_1350	0.00018123
centroid_14354	conserved hypothetical protein	0	5	101	19	centroid_1435	16.8287825	4.09081E-05	centroid_1435	0.00018123
centroid_14355	leucine rich repeat family protein	0	5	101	19	centroid_1435	16.8287825	4.09081E-05	centroid_1435	0.00018123
centroid_15005	putative membrane protein	0	5	101	19	centroid_1500	16.8287825	4.09081E-05	centroid_1500	0.00018123
centroid_15006	conserved hypothetical protein	0	5	101	19	centroid_1500	16.8287825	4.09081E-05	centroid_1500	0.00018123
centroid_15016	conserved hypothetical protein	0	5	101	19	centroid_1501	16.8287825	4.09081E-05	centroid_1501	0.00018123
centroid_16137	putative tail length tape measure domain protein	0	5	101	19	centroid_1613	16.8287825	4.09081E-05	centroid_1613	0.00018123
centroid_17365	conserved hypothetical protein	0	5	101	19	centroid_1736	16.8287825	4.09081E-05	centroid_1736	0.00018123
centroid_17373	phage integrase family protein	0	5	101	19	centroid_1737	16.8287825	4.09081E-05	centroid_1737	0.00018123
centroid_17374	conserved hypothetical protein	0	5	101	19	centroid_1737	16.8287825	4.09081E-05	centroid_1737	0.00018123
centroid_17584	conserved hypothetical protein	0	5	101	19	centroid_1758	16.8287825	4.09081E-05	centroid_1758	0.00018123
centroid_17760	type III restriction enzyme, res subunit	0	5	101	19	centroid_1776	16.8287825	4.09081E-05	centroid_1776	0.00018123
centroid_17761	helicase conserved C-terminal domain protein	0	5	101	19	centroid_1776	16.8287825	4.09081E-05	centroid_1776	0.00018123
centroid_17762	ATP-dependent DNA helicase, RecQ family domain protein	0	5	101	19	centroid_1776	16.8287825	4.09081E-05	centroid_1776	0.00018123
centroid_17795	leucine rich repeat family protein	0	5	101	19	centroid_1779	16.8287825	4.09081E-05	centroid_1779	0.00018123
centroid_17835	putative z1097 gene product	0	5	101	19	centroid_1783	16.8287825	4.09081E-05	centroid_1783	0.00018123
centroid_18529	conserved hypothetical protein	0	5	101	19	centroid_1852	16.8287825	4.09081E-05	centroid_1852	0.00018123
centroid_5606	sigma-54 interaction domain protein	0	5	101	19	centroid_5606	16.8287825	4.09081E-05	centroid_5606	0.00018123
centroid_5607	PRD domain protein	0	5	101	19	centroid_5607	16.8287825	4.09081E-05	centroid_5607	0.00018123
centroid_5608	PTS system fructose IIA component family protein	0	5	101	19	centroid_5608	16.8287825	4.09081E-05	centroid_5608	0.00018123
centroid_5609	PTS system sorbose subIIB component family protein	0	5	101	19	centroid_5609	16.8287825	4.09081E-05	centroid_5609	0.00018123
centroid_5610	PTS system sorbose-specific iic component family protein	0	5	101	19	centroid_5610	16.8287825	4.09081E-05	centroid_5610	0.00018123
centroid_5611	ystem mannose/fructose/sorbose IID component family p	0	5	101	19	centroid_5611	16.8287825	4.09081E-05	centroid_5611	0.00018123
centroid_5612	L-seryl-tRNA selenium transferase family protein	0	5	101	19	centroid_5612	16.8287825	4.09081E-05	centroid_5612	0.00018123
centroid_5613	2-dehydro-3-deoxyphosphogluconate aldolase	0	5	101	19	centroid_5613	16.8287825	4.09081E-05	centroid_5613	0.00018123
centroid_5972	type IV/VI secretion system , DotU family domain protein	0	5	101	19	centroid_5972	16.8287825	4.09081E-05	centroid_5972	0.00018123
centroid_5973	conserved hypothetical protein	0	5	101	19	centroid_5973	16.8287825	4.09081E-05	centroid_5973	0.00018123
centroid_6022	conserved hypothetical protein	0	5	101	19	centroid_6022	16.8287825	4.09081E-05	centroid_6022	0.00018123
centroid_6102	side tail fiber family protein	0	5	101	19	centroid_6102	16.8287825	4.09081E-05	centroid_6102	0.00018123
centroid_7351	CRISPR type I-E/ECOLI-associated protein CasA/Cse1	0	5	101	19	centroid_7351	16.8287825	4.09081E-05	centroid_7351	0.00018123
centroid_7551	conserved hypothetical protein	0	5	101	19	centroid_7551	16.8287825	4.09081E-05	centroid_7551	0.00018123
centroid_8767	conserved hypothetical protein	0	5	101	19	centroid_8767	16.8287825	4.09081E-05	centroid_8767	0.00018123
centroid_8785	conserved hypothetical protein	0	5	101	19	centroid_8785	16.8287825	4.09081E-05	centroid_8785	0.00018123
centroid_8801	hypothetical protein	0	5	101	19	centroid_8801	16.8287825	4.09081E-05	centroid_8801	0.00018123
centroid_8802	conserved hypothetical protein	0	5	101	19	centroid_8802	16.8287825	4.09081E-05	centroid_8802	0.00018123
centroid_8816	beta-ketoacyl synthase, N-terminal domain protein	0	5	101	19	centroid_8816	16.8287825	4.09081E-05	centroid_8816	0.00018123
centroid_8817	short chain dehydrogenase family protein	0	5	101	19	centroid_8817	16.8287825	4.09081E-05	centroid_8817	0.00018123
centroid_8818	fabA-like domain protein	0	5	101	19	centroid_8818	16.8287825	4.09081E-05	centroid_8818	0.00018123
centroid_8819	beta-ketoacyl synthase, C-terminal domain protein	0	5	101	19	centroid_8819	16.8287825	4.09081E-05	centroid_8819	0.00018123
centroid_8820	conserved hypothetical protein	0	5	101	19	centroid_8820	16.8287825	4.09081E-05	centroid_8820	0.00018123
centroid_8821	MMPL family protein	0	5	101	19	centroid_8821	16.8287825	4.09081E-05	centroid_8821	0.00018123
centroid_8822	outer membrane lipocarrier LolA family protein	0	5	101	19	centroid_8822	16.8287825	4.09081E-05	centroid_8822	0.00018123
centroid_8823	thioesterase superfamily protein	0	5	101	19	centroid_8823	16.8287825	4.09081E-05	centroid_8823	0.00018123
centroid_8824	glycosyl transferase 2 family protein	0	5	101	19	centroid_8824	16.8287825	4.09081E-05	centroid_8824	0.00018123
centroid_8828	phosphopantetheine attachment site family protein	0	5	101	19	centroid_8828	16.8287825	4.09081E-05	centroid_8828	0.00018123
centroid_8829	phosphopantetheine attachment site family protein	0	5	101	19	centroid_8829	16.8287825	4.09081E-05	centroid_8829	0.00018123
centroid_8830	beta-ketoacyl synthase, N-terminal domain protein	0	5	101	19	centroid_8830	16.8287825	4.09081E-05	centroid_8830	0.00018123
centroid_8831	O-methyltransferase family protein	0	5	101	19	centroid_8831	16.8287825	4.09081E-05	centroid_8831	0.00018123
centroid_8834	ATP-dependent DNA helicase, RecQ family protein	0	5	101	19	centroid_8834	16.8287825	4.09081E-05	centroid_8834	0.00018123
centroid_8835	DNA recombination-mediator A family protein	0	5	101	19	centroid_8835	16.8287825	4.09081E-05	centroid_8835	0.00018123
centroid_8895	conserved hypothetical protein	0	5	101	19	centroid_8895	16.8287825	4.09081E-05	centroid_8895	0.00018123
centroid_8896	tonB-dependent Receptor Plug domain protein	0	5	101	19	centroid_8896	16.8287825	4.09081E-05	centroid_8896	0.00018123
centroid_8897	olinate phosphoribosyl transferase, C-terminal domain pro	0	5	101	19	centroid_8897	16.8287825	4.09081E-05	centroid_8897	0.00018123
centroid_8898	methyltransferase domain protein	0	5	101	19	centroid_8898	16.8287825	4.09081E-05	centroid_8898	0.00018123
centroid_8899	ABC transporter family protein	0	5	101	19	centroid_8899	16.8287825	4.09081E-05	centroid_8899	0.00018123
centroid_8900	fecCD transport family protein	0	5	101	19	centroid_8900	16.8287825	4.09081E-05	centroid_8900	0.00018123
centroid_8901	periplasmic binding family protein	0	5	101	19	centroid_8901	16.8287825	4.09081E-05	centroid_8901	0.00018123

centroid_8947	outer membrane protein C	0	5	101	19	centroid_8947	16.8287825	4.09081E-05	centroid_8947	0.00018123
centroid_8958	type VII secretion system (T7SS), usher family protein	0	5	101	19	centroid_8958	16.8287825	4.09081E-05	centroid_8958	0.00018123
centroid_8959	fimbrial family protein	0	5	101	19	centroid_8959	16.8287825	4.09081E-05	centroid_8959	0.00018123
centroid_9082	fimbrial family protein	0	5	101	19	centroid_9082	16.8287825	4.09081E-05	centroid_9082	0.00018123
centroid_9084	type VII secretion system (T7SS), usher family protein	0	5	101	19	centroid_9084	16.8287825	4.09081E-05	centroid_9084	0.00018123
centroid_9085	fimbrial family protein	0	5	101	19	centroid_9085	16.8287825	4.09081E-05	centroid_9085	0.00018123
centroid_9086	fimbrial family protein	0	5	101	19	centroid_9086	16.8287825	4.09081E-05	centroid_9086	0.00018123
centroid_9106	conserved hypothetical protein	0	5	101	19	centroid_9106	16.8287825	4.09081E-05	centroid_9106	0.00018123
centroid_9221	conserved hypothetical protein	0	5	101	19	centroid_9221	16.8287825	4.09081E-05	centroid_9221	0.00018123
centroid_9252	major Facilitator Superfamily protein	0	5	101	19	centroid_9252	16.8287825	4.09081E-05	centroid_9252	0.00018123
centroid_9262	type VII secretion system (T7SS), usher family protein	0	5	101	19	centroid_9262	16.8287825	4.09081E-05	centroid_9262	0.00018123
centroid_9263	type VII secretion system (T7SS), usher family protein	0	5	101	19	centroid_9263	16.8287825	4.09081E-05	centroid_9263	0.00018123
centroid_9427	phage tail fibre repeat family protein	0	5	101	19	centroid_9427	16.8287825	4.09081E-05	centroid_9427	0.00018123
centroid_9457	n-negative pili assembly chaperone, N-terminal domain pr	0	5	101	19	centroid_9457	16.8287825	4.09081E-05	centroid_9457	0.00018123
centroid_9973	acyltransferase family protein	0	5	101	19	centroid_9973	16.8287825	4.09081E-05	centroid_9973	0.00018123

Table S5. Distribution by Phylogroup B

Gene_ID	Annotation	Phylogroup B_present	Other_present	Phylogroup B_Absent	Other_Absent	Gene_ID	chisq-stats	pvalues	Gene_ID	pvalues
centroid_5620	inner membrane protein YhaI	19	1	0	106	centroid_5620	111.278649	5.14E-26	centroid_5620	1.26E-21
centroid_13023	fimbrial family protein	19	2	0	105	centroid_1302	104.915298	1.27E-24	centroid_1302	1.32E-20
centroid_14960	outer membrane autotransporter barrel domain protein	19	2	0	105	centroid_1496	104.915298	1.27E-24	centroid_1496	1.32E-20
centroid_16055	n-negative pili assembly chaperone, N-terminal domain pr	19	2	0	105	centroid_1605	104.915298	1.27E-24	centroid_1605	1.32E-20
centroid_16056	n-negative pili assembly chaperone, C-terminal domain pr	19	2	0	105	centroid_1605	104.915298	1.27E-24	centroid_1605	1.32E-20
centroid_18522	type VII secretion system (T7SS), usher family protein	19	2	0	105	centroid_1852	104.915298	1.27E-24	centroid_1852	1.32E-20
centroid_18523	type VII secretion system (T7SS), usher family protein	19	2	0	105	centroid_1852	104.915298	1.27E-24	centroid_1852	1.32E-20
centroid_5575	inner membrane protein YmfA	19	2	0	105	centroid_5575	104.915298	1.27E-24	centroid_5575	1.32E-20
centroid_5621	cblD like pilus biogenesis initiator family protein	19	2	0	105	centroid_5621	104.915298	1.27E-24	centroid_5621	1.32E-20
centroid_5622	fimbrial family protein	19	2	0	105	centroid_5622	104.915298	1.27E-24	centroid_5622	1.32E-20
centroid_5684	fimbrial family protein	19	2	0	105	centroid_5684	104.915298	1.27E-24	centroid_5684	1.32E-20
centroid_5685	n-negative pili assembly chaperone, C-terminal domain pr	19	2	0	105	centroid_5685	104.915298	1.27E-24	centroid_5685	1.32E-20
centroid_5686	type VII secretion system (T7SS), usher family protein	19	2	0	105	centroid_5686	104.915298	1.27E-24	centroid_5686	1.32E-20
centroid_5687	fimbrial family protein	19	2	0	105	centroid_5687	104.915298	1.27E-24	centroid_5687	1.32E-20
centroid_5846	conserved hypothetical protein	19	2	0	105	centroid_5846	104.915298	1.27E-24	centroid_5846	1.32E-20
centroid_5905	putative nucleotidyltransferase family protein	19	2	0	105	centroid_5905	104.915298	1.27E-24	centroid_5905	1.32E-20
centroid_5906	zeta toxin family protein	19	2	0	105	centroid_5906	104.915298	1.27E-24	centroid_5906	1.32E-20
centroid_14944	putative ybl55 protein	19	3	0	104	centroid_1494	99.130452	2.36E-23	centroid_1494	9.69E-20
centroid_17372	fimbrial family protein	19	3	0	104	centroid_1737	99.130452	2.36E-23	centroid_1737	9.69E-20
centroid_5577	tRNA(fMet)-specific endonuclease VapC	19	3	0	104	centroid_5577	99.130452	2.36E-23	centroid_5577	9.69E-20
centroid_5578	antitoxin VapB	19	3	0	104	centroid_5578	99.130452	2.36E-23	centroid_5578	9.69E-20
centroid_5833	haemagglutinin family protein	19	3	0	104	centroid_5833	99.130452	2.36E-23	centroid_5833	9.69E-20
centroid_5904	SPFH domain / Band 7 family protein	19	3	0	104	centroid_5904	99.130452	2.36E-23	centroid_5904	9.69E-20
centroid_7287	antitoxin VapB	19	3	0	104	centroid_7287	99.130452	2.36E-23	centroid_7287	9.69E-20
centroid_5883	laminin-binding fimbrial subunit ElfA	19	4	0	103	centroid_5883	93.8486555	3.41E-22	centroid_5883	5.57E-19
centroid_12883	LPXTG cell wall anchor domain protein	19	5	0	102	centroid_1288	89.0070274	3.93E-21	centroid_1288	2.67E-18
centroid_13024	hypothetical protein	19	5	0	102	centroid_1302	89.0070274	3.93E-21	centroid_1302	2.67E-18
centroid_14504	hypothetical protein	19	5	0	102	centroid_1450	89.0070274	3.93E-21	centroid_1450	2.67E-18
centroid_11055	H+ symporter family protein	19	6	0	101	centroid_1105	84.5527482	3.74E-20	centroid_1105	1.11E-17
centroid_11056	lacY proton/sugar symporter family protein	19	6	0	101	centroid_1105	84.5527482	3.74E-20	centroid_1105	1.11E-17
centroid_12882	H+ symporter family protein	19	6	0	101	centroid_1288	84.5527482	3.74E-20	centroid_1288	1.11E-17
centroid_5812	conserved hypothetical protein	19	6	0	101	centroid_5812	84.5527482	3.74E-20	centroid_5812	1.11E-17
centroid_5854	H+ symporter family protein	19	6	0	101	centroid_5854	84.5527482	3.74E-20	centroid_5854	1.11E-17
centroid_5855	pfkB carbohydrate kinase family protein	19	6	0	101	centroid_5855	84.5527482	3.74E-20	centroid_5855	1.11E-17
centroid_5856	sucrose-6-phosphate hydrolase family protein	19	6	0	101	centroid_5856	84.5527482	3.74E-20	centroid_5856	1.11E-17
centroid_5857	lasmic binding and sugar binding domain of LacI family pr	19	6	0	101	centroid_5857	84.5527482	3.74E-20	centroid_5857	1.11E-17
centroid_9104	H+ symporter family protein	19	6	0	101	centroid_9104	84.5527482	3.74E-20	centroid_9104	1.11E-17
centroid_14505	conserved hypothetical protein	19	8	0	99	centroid_1450	76.6340831	2.06E-18	centroid_1450	1.40E-16
centroid_5674	SPR-associated endoribonuclease Cas2, subtype I-E/EC	19	8	0	99	centroid_5674	76.6340831	2.06E-18	centroid_5674	1.40E-16
centroid_5828	putative cytoplasmic protein	19	9	0	98	centroid_5828	73.0989916	1.23E-17	centroid_5828	4.34E-16
centroid_7274	type VII secretion system (T7SS), usher family protein	19	13	0	94	centroid_7274	61.1682266	5.24E-15	centroid_7274	2.18E-14
centroid_5679	protein HokA	19	16	0	91	centroid_5679	54.0099436	1.99E-13	centroid_5679	2.55E-13
centroid_8844	ive escherichia coli IMT2125 genomic chromosome, IMT	19	17	0	90	centroid_8844	51.8890064	5.87E-13	centroid_8844	5.41E-13
centroid_11133	hypothetical protein	19	29	0	78	centroid_1113	33.3320571	7.77E-09	centroid_1113	7.26E-10
centroid_11584	biquinone/plastoquinone (complex I), various chains fami	19	29	0	78	centroid_1158	33.3320571	7.77E-09	centroid_1158	7.26E-10
centroid_5580	H-Ubiquinone oxidoreductase (complex I), chain family pr	19	29	0	78	centroid_5580	33.3320571	7.77E-09	centroid_5580	7.26E-10
centroid_7285	biquinone/plastoquinone (complex I), various chains fami	19	29	0	78	centroid_7285	33.3320571	7.77E-09	centroid_7285	7.26E-10
centroid_4390	conserved hypothetical protein	19	30	0	77	centroid_4390	32.1960381	1.39E-08	centroid_4390	1.19E-09
centroid_4717	conserved hypothetical protein	19	30	0	77	centroid_4717	32.1960381	1.39E-08	centroid_4717	1.19E-09
centroid_14250	n-negative pili assembly chaperone, N-terminal domain pr	19	31	0	76	centroid_1425	31.1054814	2.44E-08	centroid_1425	1.91E-09
centroid_14251	n-negative pili assembly chaperone, C-terminal domain pr	19	31	0	76	centroid_1425	31.1054814	2.44E-08	centroid_1425	1.91E-09
centroid_18344	outer membrane usher protein HtrE	19	31	0	76	centroid_1834	31.1054814	2.44E-08	centroid_1834	1.91E-09
centroid_18345	papC N-terminal domain protein	19	31	0	76	centroid_1834	31.1054814	2.44E-08	centroid_1834	1.91E-09
centroid_4585	n-negative pili assembly chaperone, N-terminal domain pr	19	31	0	76	centroid_4585	31.1054814	2.44E-08	centroid_4585	1.91E-09
centroid_4586	CS1 type fimbrial major subunit	19	31	0	76	centroid_4586	31.1054814	2.44E-08	centroid_4586	1.91E-09
centroid_4587	putative outer membrane fimbrial usher domain protein	19	31	0	76	centroid_4587	31.1054814	2.44E-08	centroid_4587	1.91E-09
centroid_5844	outer membrane usher protein HtrE	19	31	0	76	centroid_5844	31.1054814	2.44E-08	centroid_5844	1.91E-09
centroid_5845	fimbrial family protein	19	31	0	76	centroid_5845	31.1054814	2.44E-08	centroid_5845	1.91E-09
centroid_6098	conserved hypothetical protein	19	31	0	76	centroid_6098	31.1054814	2.44E-08	centroid_6098	1.91E-09
centroid_13286	conserved hypothetical protein	19	32	0	75	centroid_1328	30.0577136	4.19E-08	centroid_1328	3.05E-09
centroid_13577	fimbrial family protein	19	32	0	75	centroid_1357	30.0577136	4.19E-08	centroid_1357	3.05E-09
centroid_13894	type VII secretion system (T7SS), usher family protein	19	32	0	75	centroid_1389	30.0577136	4.19E-08	centroid_1389	3.05E-09
centroid_13940	type VII secretion system (T7SS), usher family protein	19	32	0	75	centroid_1394	30.0577136	4.19E-08	centroid_1394	3.05E-09
centroid_16151	fimbrial family protein	19	32	0	75	centroid_1615	30.0577136	4.19E-08	centroid_1615	3.05E-09
centroid_17669	n-negative pili assembly chaperone, N-terminal domain pr	19	32	0	75	centroid_1766	30.0577136	4.19E-08	centroid_1766	3.05E-09
centroid_4408	conserved hypothetical protein	19	32	0	75	centroid_4408	30.0577136	4.19E-08	centroid_4408	3.05E-09
centroid_5841	o-4-hydroxy-6-hydroxymethyldihydropteridine diphosphc	19	32	0	75	centroid_5841	30.0577136	4.19E-08	centroid_5841	3.05E-09
centroid_5842	fimbrial family protein	19	32	0	75	centroid_5842	30.0577136	4.19E-08	centroid_5842	3.05E-09
centroid_5843	n-negative pili assembly chaperone, N-terminal domain pr	19	32	0	75	centroid_5843	30.0577136	4.19E-08	centroid_5843	3.05E-09
centroid_8769	conserved domain protein	19	32	0	75	centroid_8769	30.0577136	4.19E-08	centroid_8769	3.05E-09
centroid_12971	conserved hypothetical protein	19	34	0	73	centroid_1297	28.0808602	1.16E-07	centroid_1297	7.49E-09
centroid_17544	conserved hypothetical protein	19	34	0	73	centroid_1754	28.0808602	1.16E-07	centroid_1754	7.49E-09
centroid_4904	methyltransferase domain protein	19	34	0	73	centroid_4904	28.0808602	1.16E-07	centroid_4904	7.49E-09
centroid_4714	insA C-terminal domain protein	19	35	0	72	centroid_4714	27.1473807	1.89E-07	centroid_4714	1.16E-08
centroid_4715	conserved hypothetical protein	19	35	0	72	centroid_4715	27.1473807	1.89E-07	centroid_4715	1.16E-08
centroid_4716	putative dsORF-f3	19	35	0	72	centroid_4716	27.1473807	1.89E-07	centroid_4716	1.16E-08
centroid_5901	putative transposase	19	35	0	72	centroid_5901	27.1473807	1.89E-07	centroid_5901	1.16E-08
centroid_5902	putative dsORF-f3	19	35	0	72	centroid_5902	27.1473807	1.89E-07	centroid_5902	1.16E-08
centroid_7724	putative dsORF-f3	19	35	0	72	centroid_7724	27.1473807	1.89E-07	centroid_7724	1.16E-08
centroid_10778	BCCT transporter family protein	19	37	0	70	centroid_1077	25.3805091	4.71E-07	centroid_1077	2.67E-08
centroid_16140	conserved hypothetical protein	19	37	0	70	centroid_1614	25.3805091	4.71E-07	centroid_1614	2.67E-08
centroid_6099	conserved hypothetical protein	19	37	0	70	centroid_6099	25.3805091	4.71E-07	centroid_6099	2.67E-08
centroid_12501	lactonase, 7-bladed beta-propeller family protein	19	38	0	69	centroid_1250	24.5436071	7.26E-07	centroid_1250	4.01E-08
centroid_12502	rhomboid family protein	19	38	0	69	centroid_1250	24.5436071	7.26E-07	centroid_1250	4.01E-08
centroid_18473	conserved hypothetical protein	19	38	0	69	centroid_1847	24.5436071	7.26E-07	centroid_1847	4.01E-08
centroid_4411	rhomboid family protein	19	38	0	69	centroid_4411	24.5436071	7.26E-07	centroid_4411	4.01E-08
centroid_9309	yqeH domain protein	19	39	0	68	centroid_9309	23.7355897	1.11E-06	centroid_9309	5.96E-08
centroid_10979	n-negative pili assembly chaperone, N-terminal domain pr	19	40	0	67	centroid_1097	22.9549892	1.66E-06	centroid_1097	8.79E-08
centroid_12391	lamB porin family protein	19	40	0	67	centroid_1239	22.9549892	1.66E-06	centroid_1239	8.79E-08

centroid_14262	phosphotransferase system, EIIC family protein	19	40	0	67	centroid_1426	22.9549892	1.66E-06	centroid_1426	8.79E-08
centroid_17336	maltoporin periplasmic N-terminal extension family protein	19	40	0	67	centroid_1733	22.9549892	1.66E-06	centroid_1733	8.79E-08
centroid_17337	lamB porin family protein	19	40	0	67	centroid_1733	22.9549892	1.66E-06	centroid_1733	8.79E-08
centroid_4617	PTS system, Lactose/Cellobiose specific IIA subunit	19	40	0	67	centroid_461	22.9549892	1.66E-06	centroid_461	8.79E-08
centroid_4618	maltoporin periplasmic N-terminal extension family protein	19	40	0	67	centroid_461	22.9549892	1.66E-06	centroid_461	8.79E-08
centroid_4619	lamB porin family protein	19	40	0	67	centroid_461	22.9549892	1.66E-06	centroid_461	8.79E-08
centroid_4629	conserved hypothetical protein	19	40	0	67	centroid_462	22.9549892	1.66E-06	centroid_462	8.79E-08
centroid_4708	-negative pili assembly chaperone, C-terminal domain pr	19	40	0	67	centroid_470	22.9549892	1.66E-06	centroid_470	8.79E-08
centroid_5810	cryptic outer membrane porin BglH	19	40	0	67	centroid_581	22.9549892	1.66E-06	centroid_581	8.79E-08
centroid_8525	maltoporin periplasmic N-terminal extension family protein	19	40	0	67	centroid_852	22.9549892	1.66E-06	centroid_852	8.79E-08
centroid_8526	lamB porin family protein	19	40	0	67	centroid_852	22.9549892	1.66E-06	centroid_852	8.79E-08
centroid_9633	conserved hypothetical protein	19	40	0	67	centroid_963	22.9549892	1.66E-06	centroid_963	8.79E-08
centroid_11126	conserved hypothetical protein	19	41	0	66	centroid_1112	22.200436	2.46E-06	centroid_1112	1.29E-07
centroid_18583	type VII secretion system (T7SS), usher family protein	19	41	0	66	centroid_1858	22.200436	2.46E-06	centroid_1858	1.29E-07
centroid_4385	PBP superfamily domain protein	19	41	0	66	centroid_438	22.200436	2.46E-06	centroid_438	1.29E-07
centroid_4382	putative membrane protein	19	43	0	64	centroid_438	20.764435	5.19E-06	centroid_438	2.69E-07
centroid_4383	conserved hypothetical protein	19	43	0	64	centroid_438	20.764435	5.19E-06	centroid_438	2.69E-07
centroid_4384	conserved hypothetical protein	19	43	0	64	centroid_438	20.764435	5.19E-06	centroid_438	2.69E-07
centroid_4559	conserved hypothetical protein	19	44	0	63	centroid_455	20.080669	7.42E-06	centroid_455	7.71E-07
centroid_12474	bacterial Ig-like domain family protein	19	50	0	57	centroid_1247	16.3949299	5.14E-05	centroid_1247	4.30E-06
centroid_12985	conserved hypothetical protein	19	50	0	57	centroid_1298	16.3949299	5.14E-05	centroid_1298	4.30E-06
centroid_15186	hypothetical protein	19	50	0	57	centroid_1518	16.3949299	5.14E-05	centroid_1518	4.30E-06
centroid_16788	conserved hypothetical protein	19	50	0	57	centroid_1678	16.3949299	5.14E-05	centroid_1678	4.30E-06
centroid_18010	conserved hypothetical protein	19	50	0	57	centroid_1801	16.3949299	5.14E-05	centroid_1801	4.30E-06
centroid_10064	HTH-type transcriptional regulator MatA	19	51	0	56	centroid_1006	15.8421913	6.89E-05	centroid_1006	4.95E-06
centroid_16870	hypothetical protein	19	51	0	56	centroid_1687	15.8421913	6.89E-05	centroid_1687	4.95E-06
centroid_4428	primary amine oxidase	19	51	0	56	centroid_442	15.8421913	6.89E-05	centroid_442	4.95E-06
centroid_5377	HTH-type transcriptional regulator MatA	19	51	0	56	centroid_537	15.8421913	6.89E-05	centroid_537	4.95E-06
centroid_9966	copper amine oxidase, N2 domain protein	19	51	0	56	centroid_996	15.8421913	6.89E-05	centroid_996	4.95E-06
centroid_11967	conserved hypothetical protein	19	52	0	55	centroid_1196	15.3050624	9.15E-05	centroid_1196	6.11E-06
centroid_14977	conserved hypothetical protein	19	52	0	55	centroid_1497	15.3050624	9.15E-05	centroid_1497	6.11E-06
centroid_4663	CDP-alcohol phosphatidyltransferase family protein	19	52	0	55	centroid_466	15.3050624	9.15E-05	centroid_466	6.11E-06
centroid_5374	conserved hypothetical protein	19	52	0	55	centroid_537	15.3050624	9.15E-05	centroid_537	6.11E-06
centroid_5375	ribosomal protein L31	19	52	0	55	centroid_537	15.3050624	9.15E-05	centroid_537	6.11E-06
centroid_5376	ribosomal protein L36	19	52	0	55	centroid_537	15.3050624	9.15E-05	centroid_537	6.11E-06
centroid_7339	type VII secretion system (T7SS), usher family protein	19	52	0	55	centroid_733	15.3050624	9.15E-05	centroid_733	6.11E-06
centroid_9007	conserved hypothetical protein	19	52	0	55	centroid_900	15.3050624	9.15E-05	centroid_900	6.11E-06
centroid_5891	protein DedA	18	1	1	106	centroid_589	103.661532	2.40E-24	centroid_589	1.28E-19
centroid_13110	outer membrane autotransporter barrel domain protein	18	2	1	105	centroid_1311	97.3695232	5.75E-23	centroid_1311	1.27E-18
centroid_5572	outer membrane autotransporter barrel domain protein	18	2	1	105	centroid_557	97.3695232	5.75E-23	centroid_557	1.27E-18
centroid_13367	type IV leader peptidase family protein	18	7	1	100	centroid_1336	73.4622102	1.03E-17	centroid_1336	3.06E-15
centroid_5955	BFD-like [2Fe-2S] binding domain protein	18	7	1	100	centroid_595	73.4622102	1.03E-17	centroid_595	3.06E-15
centroid_5956	bacterioferritin	18	7	1	100	centroid_595	73.4622102	1.03E-17	centroid_595	3.06E-15
centroid_13093	CRISPR-associated helicase Cas3	18	8	1	99	centroid_1309	69.7846392	6.61E-17	centroid_1309	9.87E-15
centroid_15847	conserved hypothetical protein	18	8	1	99	centroid_1584	69.7846392	6.61E-17	centroid_1584	9.87E-15
centroid_5675	SPR-associated protein Cas6/Cse3/CasE, subtype I-E/EC	18	8	1	99	centroid_567	69.7846392	6.61E-17	centroid_567	9.87E-15
centroid_5676	RISPR-associated protein Cas5/CasD, subtype I-E/ECO	18	8	1	99	centroid_567	69.7846392	6.61E-17	centroid_567	9.87E-15
centroid_5678	CRISPR-associated helicase Cas3	18	8	1	99	centroid_567	69.7846392	6.61E-17	centroid_567	9.87E-15
centroid_6108	transposase IS116/IS110/IS902 family protein	18	8	1	99	centroid_610	69.7846392	6.61E-17	centroid_610	9.87E-15
centroid_7349	SPR-associated protein Cas7/Cse4/CasC, subtype I-E/E	18	8	1	99	centroid_734	69.7846392	6.61E-17	centroid_734	9.87E-15
centroid_7675	CRISPR-associated endonuclease Cas1	18	8	1	99	centroid_767	69.7846392	6.61E-17	centroid_767	9.87E-15
centroid_8842	SPR-associated protein Cas7/Cse4/CasC, subtype I-E/E	18	8	1	99	centroid_884	69.7846392	6.61E-17	centroid_884	9.87E-15
centroid_13094	CRISPR-associated endonuclease Cas3-HD	18	9	1	98	centroid_1309	66.3796449	3.72E-16	centroid_1309	2.93E-14
centroid_14390	CRISPR-associated endonuclease Cas3-HD	18	9	1	98	centroid_1439	66.3796449	3.72E-16	centroid_1439	2.93E-14
centroid_14737	conserved hypothetical protein	18	12	1	95	centroid_1473	57.5275916	3.33E-14	centroid_1473	5.26E-13
centroid_7086	transposase family protein	18	14	1	93	centroid_708	52.549086	4.20E-13	centroid_708	2.81E-12
centroid_5759	conserved hypothetical protein	18	31	1	76	centroid_575	26.6615391	2.42E-07	centroid_575	5.71E-08
centroid_16189	putative fimbrial subunit YadM domain protein	18	32	1	75	centroid_1618	25.688398	4.01E-07	centroid_1618	8.82E-08
centroid_6222	fimbrial family protein	18	32	1	75	centroid_622	25.688398	4.01E-07	centroid_622	8.82E-08
centroid_4607	phenolic acid decarboxylase subunit D	18	33	1	74	centroid_460	24.7536171	6.51E-07	centroid_460	1.35E-07
centroid_4608	ubiD decarboxylase family protein	18	33	1	74	centroid_460	24.7536171	6.51E-07	centroid_460	1.35E-07
centroid_4609	putative aromatic acid decarboxylase	18	33	1	74	centroid_460	24.7536171	6.51E-07	centroid_460	1.35E-07
centroid_4610	marR family protein	18	33	1	74	centroid_461	24.7536171	6.51E-07	centroid_461	1.35E-07
centroid_5672	ubiD decarboxylase family protein	18	33	1	74	centroid_567	24.7536171	6.51E-07	centroid_567	1.35E-07
centroid_5673	octaprenyl-4-hydroxybenzoate carboxy-lyase family prot	18	33	1	74	centroid_567	24.7536171	6.51E-07	centroid_567	1.35E-07
centroid_10540	glycosyl hydrolase 1 family protein	18	40	1	67	centroid_1054	19.1182125	1.23E-05	centroid_1054	1.98E-06
centroid_13126	bacterial regulatory, gntR family protein	18	40	1	67	centroid_1312	19.1182125	1.23E-05	centroid_1312	1.98E-06
centroid_13205	UTRA domain protein	18	40	1	67	centroid_1320	19.1182125	1.23E-05	centroid_1320	1.98E-06
centroid_18361	PTS system, Lactose/Cellobiose specific IIB subunit	18	40	1	67	centroid_1836	19.1182125	1.23E-05	centroid_1836	1.98E-06
centroid_4615	PTS system, Lactose/Cellobiose specific IIB subunit	18	40	1	67	centroid_461	19.1182125	1.23E-05	centroid_461	1.98E-06
centroid_4616	TS system, lactose/cellobiose IIC component family prote	18	40	1	67	centroid_461	19.1182125	1.23E-05	centroid_461	1.98E-06
centroid_5809	PTS system, Lactose/Cellobiose specific IIB subunit	18	40	1	67	centroid_580	19.1182125	1.23E-05	centroid_580	1.98E-06
centroid_7362	glycosyl hydrolase 1 family protein	18	40	1	67	centroid_736	19.1182125	1.23E-05	centroid_736	1.98E-06
centroid_6140	outer membrane protein C	18	43	1	64	centroid_614	17.1022537	3.54E-05	centroid_614	6.33E-06
centroid_8238	bacterial Ig-like domain family protein	18	43	1	64	centroid_823	17.1022537	3.54E-05	centroid_823	6.33E-06
centroid_9351	type IV leader peptidase family protein	18	46	1	61	centroid_935	15.2775631	9.28E-05	centroid_935	2.23E-05
centroid_5847	fimbrial family protein	17	2	2	105	centroid_584	89.9791957	2.41E-21	centroid_584	6.11E-17
centroid_7211	ankyrin repeat family protein	17	10	2	97	centroid_721	56.8614229	4.68E-14	centroid_721	2.60E-12
centroid_7761	putative iS621 protein	17	13	2	94	centroid_776	49.0026039	2.56E-12	centroid_776	3.49E-11
centroid_15812	hypothetical protein	17	16	2	91	centroid_1581	42.5764562	6.80E-11	centroid_1581	3.20E-10
centroid_4626	type-1 fimbrial protein, A chain	17	31	2	76	centroid_462	22.5444185	2.05E-06	centroid_462	8.38E-07
centroid_15960	conserved hypothetical protein	17	32	2	75	centroid_1596	21.648616	3.27E-06	centroid_1596	1.25E-06
centroid_17338	type VII secretion system (T7SS), usher family protein	17	32	2	75	centroid_1733	21.648616	3.27E-06	centroid_1733	1.25E-06
centroid_4627	chaperone protein FocC	17	33	2	74	centroid_462	20.7891845	5.13E-06	centroid_462	1.85E-06
centroid_6110	type IV secretion system Vgr family domain protein	17	33	2	74	centroid_611	20.7891845	5.13E-06	centroid_611	1.85E-06
centroid_4305	plasmid stabilisation system family protein	17	38	2	69	centroid_430	16.9688811	3.80E-05	centroid_430	1.68E-05
centroid_4306	ribbon-helix-helix , copG family protein	17	38	2	69	centroid_430	16.9688811	3.80E-05	centroid_430	1.68E-05
centroid_4614	deoR-like helix-turn-helix domain protein	17	40	2	67	centroid_461	15.6324805	7.69E-05	centroid_461	2.49E-05
centroid_11165	hypothetical protein	16	2	3	105	centroid_1116	82.7423758	9.35E-20	centroid_1116	1.97E-15
centroid_5573	conserved hypothetical protein	16	2	3	105	centroid_557	82.7423758	9.35E-20	centroid_557	1.97E-15
centroid_5899	L-galactonate transporter	16	2	3	105	centroid_589	82.7423758	9.35E-20	centroid_589	1.97E-15
centroid_5581	putative membrane protein	16	3	3	104	centroid_558	77.2648409	1.50E-18	centroid_558	1.22E-14
centroid_11324	conserved hypothetical protein	16	24	3	83	centroid_1132	25.6422194	4.11E-07	centroid_1132	4.26E-07
centroid_5714	conserved hypothetical protein	16	24	3	83	centroid_571	25.6422194	4.11E-07	centroid_571	4.26E-07
centroid_13521	conjugative transfer relaxase protein TraI	16	26	3	81	centroid_1352	23.4351943	1.29E-06	centroid_1352	1.06E-06
centroid_14951	conserved hypothetical protein	16	28	3	79	centroid_1495	21.4325075	3.67E-06	centroid_1495	2.47E-06
centroid_15014	conserved hypothetical protein	16	28	3	79	centroid_1501	21.4325075	3.67E-06	centroid_1501	2.47E-06
centroid_5564	conserved hypothetical protein	16	28	3	79	centroid_556	21.4325075	3.67E-06	centroid_556	2.47E-06
centroid_17931	conserved hypothetical protein	16	29	3	78	centroid_1793	20.4993606	5.97E-06	centroid_1793	3.70E-06
centroid_18482	conserved hypothetical protein	16	29	3	78	centroid_1848	20.4993606	5.97E-06	centroid_1848	3.70E-06
centroid_10927	conserved hypothetical protein	16	31	3	76	centroid_1092	18.7551243	1.49E-05	centroid_1092	8.03E-06
centroid_11168	conserved hypothetical protein	16	31	3	76	centroid_1116	18.7551243	1.49E-05	centroid_1116	8.03E-06
centroid_13619	conserved hypothetical protein	16	31	3	76	centroid_1361	18.7551243	1.49E-05	centroid_1361	8.03E-06
centroid_18481	conserved hypothetical protein	16	31	3	76	centroid_1848	18.7551243	1.49E-05	centroid_1848	8.03E-06
centroid_11134	hypothetical protein	16	32	3	75	centroid_1113	17.9390229	2.28E-05	centroid_1113	1.16E-05
centroid_15015	conserved hypothetical protein	16	32	3	75	centroid_1501	17.9390229	2.28E-05	centroid_1501	1.16E-05
centroid_6101	putative membrane protein	16	32	3	75	centroid_610	17.9390229	2.28E-05	centroid_610	1.16E-05

centroid_7846	involved in detoxification of methylglyoxal domain protein	16	33	3	74	centroid_7846	17.1572687	3.44E-05	centroid_7846	1.67E-05
centroid_4387	hicB family protein	16	34	3	73	centroid_4387	16.4078409	5.11E-05	centroid_4387	2.36E-05
centroid_7092	hypothetical protein	16	35	3	72	centroid_7092	15.6888794	7.47E-05	centroid_7092	5.12E-05
centroid_11606	YoaG domain protein	15	1	4	106	centroid_1160	81.6808607	1.60E-19	centroid_1160	5.82E-15
centroid_5875	conserved hypothetical protein	15	1	4	106	centroid_5875	81.6808607	1.60E-19	centroid_5875	5.82E-15
centroid_5876	hicB family protein	15	1	4	106	centroid_5876	81.6808607	1.60E-19	centroid_5876	5.82E-15
centroid_12984	bacterial Ig-like domain family protein	15	2	4	105	centroid_1298	75.6577497	3.37E-18	centroid_1298	4.78E-14
centroid_5623	conserved hypothetical protein	15	2	4	105	centroid_5623	75.6577497	3.37E-18	centroid_5623	4.78E-14
centroid_5900	racemase / muconate lactonizing enzyme, N-terminal dor	15	2	4	105	centroid_5900	75.6577497	3.37E-18	centroid_5900	4.78E-14
centroid_11144	rhs core with extension domain protein	15	3	4	104	centroid_1114	70.3055829	5.08E-17	centroid_1114	2.77E-13
centroid_11150	putative yhaC	15	3	4	104	centroid_1115	70.3055829	5.08E-17	centroid_1115	2.77E-13
centroid_12963	putative dsORF-e4	15	3	4	104	centroid_1296	70.3055829	5.08E-17	centroid_1296	2.77E-13
centroid_12964	conserved hypothetical protein	15	3	4	104	centroid_1296	70.3055829	5.08E-17	centroid_1296	2.77E-13
centroid_17233	lrate uptake ABC transporter 2 (CUT2) family, ATP-bindir	15	3	4	104	centroid_1723	70.3055829	5.08E-17	centroid_1723	2.77E-13
centroid_17234	ABC transporter family protein	15	3	4	104	centroid_1723	70.3055829	5.08E-17	centroid_1723	2.77E-13
centroid_18642	conserved hypothetical protein	15	3	4	104	centroid_1864	70.3055829	5.08E-17	centroid_1864	2.77E-13
centroid_5651	ASCH domain protein	15	3	4	104	centroid_5651	70.3055829	5.08E-17	centroid_5651	2.77E-13
centroid_5652	putative LACI-type transcriptional regulator	15	3	4	104	centroid_5652	70.3055829	5.08E-17	centroid_5652	2.77E-13
centroid_5653	heme ABC exporter, ATP-binding protein CcmA	15	3	4	104	centroid_5653	70.3055829	5.08E-17	centroid_5653	2.77E-13
centroid_5654	ain amino acid transport system / permease component f	15	3	4	104	centroid_5654	70.3055829	5.08E-17	centroid_5654	2.77E-13
centroid_5655	ain amino acid transport system / permease component f	15	3	4	104	centroid_5655	70.3055829	5.08E-17	centroid_5655	2.77E-13
centroid_5813	putative membrane protein	15	3	4	104	centroid_5813	70.3055829	5.08E-17	centroid_5813	2.77E-13
centroid_5873	conserved hypothetical protein	15	3	4	104	centroid_5873	70.3055829	5.08E-17	centroid_5873	2.77E-13
centroid_6089	putative rHS protein	15	3	4	104	centroid_6089	70.3055829	5.08E-17	centroid_6089	2.77E-13
centroid_6090	conserved hypothetical protein	15	3	4	104	centroid_6090	70.3055829	5.08E-17	centroid_6090	2.77E-13
centroid_12982	sel1 repeat family protein	15	4	4	103	centroid_1298	65.5184672	5.76E-16	centroid_1298	1.27E-12
centroid_5660	sel1 repeat family protein	15	4	4	103	centroid_5660	65.5184672	5.76E-16	centroid_5660	1.27E-12
centroid_5661	acetyltransferase domain protein	15	4	4	103	centroid_5661	65.5184672	5.76E-16	centroid_5661	1.27E-12
centroid_5662	conserved hypothetical protein	15	4	4	103	centroid_5662	65.5184672	5.76E-16	centroid_5662	1.27E-12
centroid_5819	conserved hypothetical protein	15	4	4	103	centroid_5819	65.5184672	5.76E-16	centroid_5819	1.27E-12
centroid_5820	helix-turn-helix family protein	15	4	4	103	centroid_5820	65.5184672	5.76E-16	centroid_5820	1.27E-12
centroid_5821	putative transcription elongation factor GreB	15	4	4	103	centroid_5821	65.5184672	5.76E-16	centroid_5821	1.27E-12
centroid_5822	alpha/beta hydrolase fold family protein	15	4	4	103	centroid_5822	65.5184672	5.76E-16	centroid_5822	1.27E-12
centroid_7345	aldo/keto reductase family protein	15	4	4	103	centroid_7345	65.5184672	5.76E-16	centroid_7345	1.27E-12
centroid_9422	putative membrane protein	15	4	4	103	centroid_9422	65.5184672	5.76E-16	centroid_9422	1.27E-12
centroid_8902	conserved hypothetical protein	15	5	4	102	centroid_8902	61.2116897	5.13E-15	centroid_8902	4.90E-12
centroid_5605	putative membrane protein	15	7	4	100	centroid_5605	53.7773172	2.25E-13	centroid_5605	5.02E-11
centroid_5814	putative membrane protein	15	7	4	100	centroid_5814	53.7773172	2.25E-13	centroid_5814	5.02E-11
centroid_12532	conserved hypothetical family protein	15	9	4	98	centroid_1253	47.5879134	5.26E-12	centroid_1253	3.58E-10
centroid_18577	conserved hypothetical protein	15	9	4	98	centroid_1857	47.5879134	5.26E-12	centroid_1857	3.58E-10
centroid_8547	conserved hypothetical protein	15	9	4	98	centroid_8547	47.5879134	5.26E-12	centroid_8547	3.58E-10
centroid_5911	helix-turn-helix domain protein	15	12	4	95	centroid_5911	40.033627	2.50E-10	centroid_5911	4.25E-09
centroid_8815	putative permease family protein	15	12	4	95	centroid_8815	40.033627	2.50E-10	centroid_8815	4.25E-09
centroid_15857	aldehyde dehydrogenase family protein	15	15	4	92	centroid_1585	34.0025301	5.50E-09	centroid_1585	3.38E-08
centroid_6419	homoprotocatechuate degradation operon regulator, HpaR	15	15	4	92	centroid_6419	34.0025301	5.50E-09	centroid_6419	3.38E-08
centroid_11757	rhs core with extension domain protein	15	23	4	84	centroid_1175	22.6303595	1.96E-06	centroid_1175	2.43E-06
centroid_5861	glycosyl hydrolases 15 family protein	15	26	4	81	centroid_5861	19.5322804	9.89E-06	centroid_5861	8.75E-06
centroid_9569	glycosyl hydrolases 15 family protein	15	27	4	80	centroid_9569	18.6009592	1.61E-05	centroid_9569	1.30E-05
centroid_4447	putative dNA-damage-inducible protein D	15	107	4	0	centroid_4447	16.9199499	3.90E-05	centroid_4447	0.00038725
centroid_5882	conserved hypothetical protein	15	107	4	0	centroid_5882	16.9199499	3.90E-05	centroid_5882	0.00038725
centroid_7639	putative dNA-damage-inducible protein D	15	107	4	0	centroid_7639	16.9199499	3.90E-05	centroid_7639	0.00038725
centroid_4628	type VII secretion system (T7SS), usher family protein	15	30	4	77	centroid_4628	16.0645352	6.12E-05	centroid_4628	3.98E-05
centroid_18643	RHS repeat-associated core domain protein	14	1	5	106	centroid_1864	74.6354981	5.66E-18	centroid_1864	1.21E-13
centroid_5853	n-negative pili assembly chaperone, N-terminal domain pr	14	1	5	106	centroid_5853	74.6354981	5.66E-18	centroid_5853	1.21E-13
centroid_11109	n-negative pili assembly chaperone, C-terminal domain pr	14	2	5	105	centroid_1110	68.7247725	1.13E-16	centroid_1110	9.30E-13
centroid_5849	conserved hypothetical protein	14	2	5	105	centroid_5849	68.7247725	1.13E-16	centroid_5849	9.30E-13
centroid_5850	fimbrial family protein	14	2	5	105	centroid_5850	68.7247725	1.13E-16	centroid_5850	9.30E-13
centroid_5851	fimbrial family protein	14	2	5	105	centroid_5851	68.7247725	1.13E-16	centroid_5851	9.30E-13
centroid_5852	fimbrial family protein	14	2	5	105	centroid_5852	68.7247725	1.13E-16	centroid_5852	9.30E-13
centroid_14626	conserved hypothetical protein	14	7	5	100	centroid_1462	47.6482046	5.10E-12	centroid_1462	7.23E-10
centroid_7350	CRISPR type I-E/ECOLI-associated protein CasB/Cse2	14	7	5	100	centroid_7350	47.6482046	5.10E-12	centroid_7350	7.23E-10
centroid_6427	-hydroxyphenylacetate catabolism regulatory protein Hpa	14	13	5	94	centroid_6427	32.7240531	1.06E-08	centroid_6427	9.45E-08
centroid_13516	putative tail length tape measure domain protein	14	14	5	93	centroid_1351	30.8658387	2.76E-08	centroid_1351	1.80E-07
centroid_12523	ydroxyphenylacetate 3-hydroxylase C terminal family pro	14	15	5	92	centroid_1252	29.1379581	6.74E-08	centroid_1252	3.32E-07
centroid_12524	ydroxyphenylacetate 3-hydroxylase N terminal family pro	14	15	5	92	centroid_1252	29.1379581	6.74E-08	centroid_1252	3.32E-07
centroid_6420	otocatechuate catabolism bifunctional isomerase/decarb	14	15	5	92	centroid_6420	29.1379581	6.74E-08	centroid_6420	3.32E-07
centroid_6421	oxymethyl-2-hydroxymuconate semialdehyde dehydrog	14	15	5	92	centroid_6421	29.1379581	6.74E-08	centroid_6421	3.32E-07
centroid_6422	3,4-dihydroxyphenylacetate 2,3-dioxygenase	14	15	5	92	centroid_6422	29.1379581	6.74E-08	centroid_6422	3.32E-07
centroid_6424	2-oxo-hepta-3-ene-1,7-dioic acid hydratase	14	15	5	92	centroid_6424	29.1379581	6.74E-08	centroid_6424	3.32E-07
centroid_6425	2,4-dihydroxyhept-2-ene-1,7-dioic acid aldolase	14	15	5	92	centroid_6425	29.1379581	6.74E-08	centroid_6425	3.32E-07
centroid_6426	4-hydroxyphenylacetate permease	14	15	5	92	centroid_6426	29.1379581	6.74E-08	centroid_6426	3.32E-07
centroid_6428	lroxyphenylacetate 3-monooxygenase, oxygenase comp	14	15	5	92	centroid_6428	29.1379581	6.74E-08	centroid_6428	3.32E-07
centroid_6429	droxyphenylacetate 3-monooxygenase, reductase compo	14	15	5	92	centroid_6429	29.1379581	6.74E-08	centroid_6429	3.32E-07
centroid_11086	o-L-gulonate-6-phosphate decarboxylase UlaD domain p	14	107	5	0	centroid_1108	22.822425	1.78E-06	centroid_1108	4.76E-05
centroid_2634	putative 4-phosphopantetheinyl transferase EntD	14	107	5	0	centroid_2634	22.822425	1.78E-06	centroid_2634	4.76E-05
centroid_14352	putative predicted protein	14	20	5	87	centroid_1435	22.053186	2.65E-06	centroid_1435	4.65E-06
centroid_6980	conserved hypothetical protein	14	25	5	82	centroid_6980	16.8341725	4.08E-05	centroid_6980	3.88E-05
centroid_10025	conserved hypothetical protein	14	26	5	81	centroid_1002	15.9534263	6.49E-05	centroid_1002	5.66E-05
centroid_16717	hypothetical protein	14	26	5	81	centroid_1671	15.9534263	6.49E-05	centroid_1671	5.66E-05
centroid_16718	conserved hypothetical protein	14	26	5	81	centroid_1671	15.9534263	6.49E-05	centroid_1671	5.66E-05
centroid_8551	conserved hypothetical protein	14	26	5	81	centroid_8551	15.9534263	6.49E-05	centroid_8551	5.66E-05
centroid_13498	conserved hypothetical protein	13	3	6	104	centroid_1349	56.8868125	4.62E-14	centroid_1349	7.64E-11
centroid_15497	outer membrane autotransporter barrel domain protein	13	3	6	104	centroid_1549	56.8868125	4.62E-14	centroid_1549	7.64E-11
centroid_15498	nded Signal Peptide of Type V secretion system family pr	13	3	6	104	centroid_1549	56.8868125	4.62E-14	centroid_1549	7.64E-11
centroid_5647	outer membrane autotransporter barrel domain protein	13	3	6	104	centroid_5647	56.8868125	4.62E-14	centroid_5647	7.64E-11
centroid_5677	CRISPR type I-E/ECOLI-associated protein CasA/Cse1	13	7	6	100	centroid_5677	41.747754	1.04E-10	centroid_5677	8.57E-09
centroid_8843	CT1975-like family protein	13	7	6	100	centroid_8843	41.747754	1.04E-10	centroid_8843	8.57E-09
centroid_12632	mu-like prophage major head subunit gpT family protein	13	13	6	94	centroid_1263	27.8555159	1.31E-07	centroid_1263	8.27E-07
centroid_18615	putative transposase	13	13	6	94	centroid_1861	27.8555159	1.31E-07	centroid_1861	8.27E-07
centroid_5804	phage tail tape measure protein, lambda family	13	15	6	92	centroid_5804	24.5707083	7.16E-07	centroid_5804	2.66E-06
centroid_7317	conserved hypothetical protein	13	15	6	92	centroid_7317	24.5707083	7.16E-07	centroid_7317	2.66E-06
centroid_6124	yhaC domain protein	13	106	6	1	centroid_6124	23.3326582	1.36E-06	centroid_6124	3.50E-05
centroid_11244	conserved hypothetical family protein	13	17	6	90	centroid_1124	21.7356585	3.13E-06	centroid_1124	7.58E-06
centroid_5803	phage tail assembly protein T	13	18	6	89	centroid_5803	20.4598148	6.09E-06	centroid_5803	1.23E-05
centroid_7253	phage minor tail protein G	13	18	6	89	centroid_7253	20.4598148	6.09E-06	centroid_7253	1.23E-05
centroid_13112	bacterial Ig-like domain family protein	13	19	6	88	centroid_1311	19.266742	1.14E-05	centroid_1311	1.95E-05
centroid_13448	hypothetical protein	13	19	6	88	centroid_1344	19.266742	1.14E-05	centroid_1344	1.95E-05
centroid_5805	phage minor tail family protein	13	19	6	88	centroid_5805	19.266742	1.14E-05	centroid_5805	1.95E-05
centroid_6608	bacterial Ig-like domain family protein	13	19	6	88	centroid_6608	19.266742	1.14E-05	centroid_6608	1.95E-05
centroid_12447	bacterial Ig-like domain family protein	13	20	6	87	centroid_1244	18.1490105	2.04E-05	centroid_1244	3.04E-05
centroid_17335	bacterial Ig-like domain family protein	13	20	6	87	centroid_1733	18.1490105	2.04E-05	centroid_1733	3.04E-05
centroid_5632	bacterial Ig-like domain family protein	13	20	6	87	centroid_5632	18.1490105	2.04E-05	centroid_5632	3.04E-05
centroid_16839	bacterial Ig-like domain family protein	13	21	6	86	centroid_1683	17.1000685	3.55E-05	centroid_1683	4.63E-05
centroid_10954	intimin C-type lectin domain protein	13	22	6	85	centroid_1095	16.1141171	5.96E-05	centroid_1095	6.93E-05
centroid_5633	intimin C-type lectin domain protein	13	22	6	85	centroid_5633	16.1141171	5.96E-05	centroid_5633	6.93E-05
centroid_8549	intimin C-type lectin domain protein	13	22	6	85	centroid_8549	16.1141171	5.96E-05	centroid_8549	6.93E-05
centroid_10088	conserved hypothetical protein	12	0	7	107	centroid_1008	67.5426968	2.06E-16	centroid_1008	2.59E-12

centroid_12889	bacterial regulatory , arsR family protein	12	0	7	107	centroid_1288	67.5426968	2.06E-16	centroid_1288	2.59E-12
centroid_5604	pfkB carbohydrate kinase family protein	12	0	7	107	centroid_5604	67.5426968	2.06E-16	centroid_5604	2.59E-12
centroid_13466	RHS repeat-associated core domain protein	12	1	7	106	centroid_1346	60.9565398	5.83E-15	centroid_1346	3.17E-11
centroid_961	putative pseudouridine transporter	12	107	7	0	centroid_961	35.0135702	3.27E-09	centroid_961	5.97E-07
centroid_9176	putative yfdA protein	12	8	7	99	centroid_9176	33.4081722	7.47E-09	centroid_9176	2.02E-07
centroid_10033	conserved hypothetical protein	12	9	7	98	centroid_1003	30.9886867	2.60E-08	centroid_1003	4.42E-07
centroid_13111	conserved hypothetical protein	12	9	7	98	centroid_1311	30.9886867	2.60E-08	centroid_1311	4.42E-07
centroid_6792	phage tail tape measure protein, lambda family	12	12	7	95	centroid_6792	24.9643493	5.84E-07	centroid_6792	3.36E-06
centroid_10961	trbC domain protein	12	13	7	94	centroid_1096	23.2857214	1.40E-06	centroid_1096	6.06E-06
centroid_15492	putative predicted protein	12	14	7	93	centroid_1549	21.7403515	3.12E-06	centroid_1549	1.06E-05
centroid_12838	integrase core domain protein	12	104	7	3	centroid_1283	21.138612	4.27E-06	centroid_1283	5.42E-05
centroid_4773	conserved hypothetical protein	12	102	7	5	centroid_4773	15.8242097	6.95E-05	centroid_4773	0.00029613
centroid_12888	pfkB carbohydrate kinase family protein	11	0	8	107	centroid_1288	60.8013221	6.31E-15	centroid_1288	3.72E-11
centroid_16123	conserved hypothetical protein	11	2	8	105	centroid_1612	48.846777	2.77E-12	centroid_1612	2.54E-09
centroid_5816	DKNYY family protein	11	2	8	105	centroid_5816	48.846777	2.77E-12	centroid_5816	2.54E-09
centroid_7386	conserved hypothetical protein	11	2	8	105	centroid_7386	48.846777	2.77E-12	centroid_7386	2.54E-09
centroid_7527	conserved hypothetical protein	11	2	8	105	centroid_7527	48.846777	2.77E-12	centroid_7527	2.54E-09
centroid_3606	putative mRNA interferase HicA	11	107	8	0	centroid_3606	41.2864505	1.31E-10	centroid_3606	6.02E-08
centroid_10046	antitoxin HigA	11	4	8	103	centroid_1004	40.1063958	2.41E-10	centroid_1004	3.89E-08
centroid_15002	DKNYY family protein	11	4	8	103	centroid_1500	40.1063958	2.41E-10	centroid_1500	3.89E-08
centroid_17830	helix-turn-helix domain protein	11	4	8	103	centroid_1783	40.1063958	2.41E-10	centroid_1783	3.89E-08
centroid_17831	conserved hypothetical protein	11	4	8	103	centroid_1783	40.1063958	2.41E-10	centroid_1783	3.89E-08
centroid_5815	conserved hypothetical protein	11	4	8	103	centroid_5815	40.1063958	2.41E-10	centroid_5815	3.89E-08
centroid_7196	acetyltransferase family protein	11	4	8	103	centroid_7196	40.1063958	2.41E-10	centroid_7196	3.89E-08
centroid_7197	conserved hypothetical protein	11	4	8	103	centroid_7197	40.1063958	2.41E-10	centroid_7197	3.89E-08
centroid_7347	antitoxin HigA	11	4	8	103	centroid_7347	40.1063958	2.41E-10	centroid_7347	3.89E-08
centroid_10235	fibronectin type III family protein	11	5	8	102	centroid_1023	36.5652769	1.48E-09	centroid_1023	1.16E-07
centroid_13480	fibronectin type III family protein	11	5	8	102	centroid_1348	36.5652769	1.48E-09	centroid_1348	1.16E-07
centroid_7818	conserved hypothetical protein	11	105	8	2	centroid_7818	30.4557347	3.42E-08	centroid_7818	2.28E-06
centroid_5800	prophage minor tail Z family protein	11	9	8	98	centroid_5800	25.9968478	3.42E-07	centroid_5800	3.39E-06
centroid_13292	conserved hypothetical protein	11	10	8	97	centroid_1329	23.997639	9.65E-07	centroid_1329	6.63E-06
centroid_5796	phage portal protein, lambda family	11	12	8	95	centroid_5796	20.5369313	5.85E-06	centroid_5796	2.20E-05
centroid_5798	conserved hypothetical protein	11	13	8	94	centroid_5798	19.0309272	1.29E-05	centroid_5798	3.78E-05
centroid_5799	TP-binding sugar transporter from pro-phage family prote	11	13	8	94	centroid_5799	19.0309272	1.29E-05	centroid_5799	3.78E-05
centroid_11474	penicillin amidase family protein	10	0	9	107	centroid_1147	54.1793909	1.83E-13	centroid_1147	4.79E-10
centroid_13225	RHS repeat-associated core domain protein	10	0	9	107	centroid_1322	54.1793909	1.83E-13	centroid_1322	4.79E-10
centroid_15860	penicillin amidase family protein	10	0	9	107	centroid_1586	54.1793909	1.83E-13	centroid_1586	4.79E-10
centroid_5682	repair family protein	10	0	9	107	centroid_5682	54.1793909	1.83E-13	centroid_5682	4.79E-10
centroid_5683	hypothetical protein	10	0	9	107	centroid_5683	54.1793909	1.83E-13	centroid_5683	4.79E-10
centroid_9988	penicillin amidase family protein	10	0	9	107	centroid_9988	54.1793909	1.83E-13	centroid_9988	4.79E-10
centroid_11139	putative rhs core protein with extension	10	1	9	106	centroid_1113	47.8251708	4.66E-12	centroid_1113	4.90E-09
centroid_17597	RHS Repeat family protein	10	1	9	106	centroid_1759	47.8251708	4.66E-12	centroid_1759	4.90E-09
centroid_6106	integrase core domain protein	10	1	9	106	centroid_6106	47.8251708	4.66E-12	centroid_6106	4.90E-09
centroid_7531	RHS repeat-associated core domain protein	10	1	9	106	centroid_7531	47.8251708	4.66E-12	centroid_7531	4.90E-09
centroid_11137	putative rhs core protein	10	2	9	105	centroid_1113	42.5397196	6.93E-11	centroid_1113	2.73E-08
centroid_11138	conserved hypothetical protein	10	2	9	105	centroid_1113	42.5397196	6.93E-11	centroid_1113	2.73E-08
centroid_13461	RHS repeat-associated core domain protein	10	2	9	105	centroid_1346	42.5397196	6.93E-11	centroid_1346	2.73E-08
centroid_18514	putative transcriptional activator Ogr/delta	10	3	9	104	centroid_1851	38.0766368	6.80E-10	centroid_1851	1.10E-07
centroid_5681	lemA family protein	10	3	9	104	centroid_5681	38.0766368	6.80E-10	centroid_5681	1.10E-07
centroid_18198	phage tail tape measure protein, lambda family	10	4	9	103	centroid_1819	34.2599299	4.82E-09	centroid_1819	3.57E-07
centroid_18234	lemA family protein	10	4	9	103	centroid_1823	34.2599299	4.82E-09	centroid_1823	3.57E-07
centroid_15977	transposase, IS605 OrfB family	10	103	9	4	centroid_1597	28.6461192	8.69E-08	centroid_1597	2.90E-06
centroid_5659	conserved hypothetical protein	10	6	9	101	centroid_5659	28.0817014	1.16E-07	centroid_5659	2.45E-06
centroid_7871	integrase core domain protein	10	6	9	101	centroid_7871	28.0817014	1.16E-07	centroid_7871	2.45E-06
centroid_8976	conserved hypothetical protein	10	6	9	101	centroid_8976	28.0817014	1.16E-07	centroid_8976	2.45E-06
centroid_12688	phage tail tape measure protein, lambda family	10	7	9	100	centroid_1268	25.5493945	4.31E-07	centroid_1268	5.52E-06
centroid_12380	integrase core domain protein	10	8	9	99	centroid_1238	23.3060748	1.38E-06	centroid_1238	1.15E-05
centroid_5797	clp protease family protein	10	10	9	97	centroid_5797	19.5137806	9.99E-06	centroid_5797	4.15E-05
centroid_7396	outer membrane porin protein OmpD	10	10	9	97	centroid_7396	19.5137806	9.99E-06	centroid_7396	4.15E-05
centroid_9788	conserved hypothetical protein	10	10	9	97	centroid_9788	19.5137806	9.99E-06	centroid_9788	4.15E-05
centroid_5520	integrase core domain protein	10	99	9	8	centroid_5520	18.7137552	1.52E-05	centroid_5520	7.07E-05
centroid_15925	reverse transcriptase family protein	10	98	9	9	centroid_1592	16.9430644	3.85E-05	centroid_1592	0.00012992
centroid_4181	reverse transcriptase family protein	10	98	9	9	centroid_4181	16.9430644	3.85E-05	centroid_4181	0.00012992
centroid_7880	reverse transcriptase family protein	10	98	9	9	centroid_7880	16.9430644	3.85E-05	centroid_7880	0.00012992
centroid_7881	reverse transcriptase family protein	10	98	9	9	centroid_7881	16.9430644	3.85E-05	centroid_7881	0.00012992
centroid_7943	group II intron, maturase-specific domain protein	10	98	9	9	centroid_7943	16.9430644	3.85E-05	centroid_7943	0.00012992
centroid_8104	reverse transcriptase family protein	10	98	9	9	centroid_8104	16.9430644	3.85E-05	centroid_8104	0.00012992
centroid_8276	reverse transcriptase family protein	10	98	9	9	centroid_8276	16.9430644	3.85E-05	centroid_8276	0.00012992
centroid_10180	integrase core domain protein	10	97	9	10	centroid_1018	15.3678278	8.85E-05	centroid_1018	0.00022662
centroid_14811	integrase core domain protein	10	97	9	10	centroid_1481	15.3678278	8.85E-05	centroid_1481	0.00022662
centroid_16992	phage antitermination Q family protein	10	97	9	10	centroid_1699	15.3678278	8.85E-05	centroid_1699	0.00022662
centroid_2828	FAD dependent oxidoreductase family protein	9	107	10	0	centroid_2828	54.1793909	1.83E-13	centroid_2828	4.79E-10
centroid_2829	putative 4Fe-4S binding protein	9	107	10	0	centroid_2829	54.1793909	1.83E-13	centroid_2829	4.79E-10
centroid_2830	conserved hypothetical protein	9	107	10	0	centroid_2830	54.1793909	1.83E-13	centroid_2830	4.79E-10
centroid_4505	carbohydrate kinase, FGGY family	9	107	10	0	centroid_4505	54.1793909	1.83E-13	centroid_4505	4.79E-10
centroid_470	conserved hypothetical protein	9	106	10	1	centroid_470	47.8251708	4.66E-12	centroid_470	4.90E-09
centroid_5658	outer membrane autotransporter barrel domain protein	9	0	10	107	centroid_5658	47.6749026	5.03E-12	centroid_5658	5.61E-09
centroid_7546	RHS repeat-associated core domain protein	9	1	10	106	centroid_7546	41.4693276	1.20E-10	centroid_7546	5.18E-08
centroid_17674	conserved hypothetical protein	9	103	10	4	centroid_1767	34.2599299	4.82E-09	centroid_1767	3.57E-07
centroid_2839	conserved hypothetical protein	9	103	10	4	centroid_2839	34.2599299	4.82E-09	centroid_2839	3.57E-07
centroid_2840	repair family protein	9	103	10	4	centroid_2840	34.2599299	4.82E-09	centroid_2840	3.57E-07
centroid_4506	conserved hypothetical protein	9	103	10	4	centroid_4506	34.2599299	4.82E-09	centroid_4506	3.57E-07
centroid_11094	hypothetical protein	9	3	10	104	centroid_1109	32.1960222	1.39E-08	centroid_1109	9.69E-07
centroid_11095	putative phage immunity repressor protein	9	3	10	104	centroid_1109	32.1960222	1.39E-08	centroid_1109	9.69E-07
centroid_13473	hypothetical protein	9	3	10	104	centroid_1347	32.1960222	1.39E-08	centroid_1347	9.69E-07
centroid_6491	ogr/Delta-like zinc finger family protein	9	3	10	104	centroid_6491	32.1960222	1.39E-08	centroid_6491	9.69E-07
centroid_6492	putative glyco3, capsid size determination protein Sid	9	3	10	104	centroid_6492	32.1960222	1.39E-08	centroid_6492	9.69E-07
centroid_6495	putative derepression protein	9	3	10	104	centroid_6495	32.1960222	1.39E-08	centroid_6495	9.69E-07
centroid_6496	putative predicted protein	9	3	10	104	centroid_6496	32.1960222	1.39E-08	centroid_6496	9.69E-07
centroid_6497	conserved hypothetical protein	9	3	10	104	centroid_6497	32.1960222	1.39E-08	centroid_6497	9.69E-07
centroid_6498	putative P4-specific DNA primase	9	3	10	104	centroid_6498	32.1960222	1.39E-08	centroid_6498	9.69E-07
centroid_17686	putative domain protein	9	102	10	5	centroid_1768	30.9605456	2.63E-08	centroid_1768	9.93E-07
centroid_10137	caudovirales tail fibre assembly family protein	9	101	10	6	centroid_1013	28.0817014	1.16E-07	centroid_1013	2.45E-06
centroid_18607	hypothetical protein	9	5	10	102	centroid_1860	25.6140863	4.17E-07	centroid_1860	7.48E-06
centroid_5921	traG-like , N-terminal region family protein	9	5	10	102	centroid_5921	25.6140863	4.17E-07	centroid_5921	7.48E-06
centroid_7413	conserved hypothetical protein	9	5	10	102	centroid_7413	25.6140863	4.17E-07	centroid_7413	7.48E-06
centroid_7421	hypothetical protein	9	5	10	102	centroid_7421	25.6140863	4.17E-07	centroid_7421	7.48E-06
centroid_10919	type IV/VI secretion system , DotU family domain protein	9	6	10	101	centroid_1091	22.9966206	1.62E-06	centroid_1091	1.72E-05
centroid_12120	conserved hypothetical protein	9	6	10	101	centroid_1212	22.9966206	1.62E-06	centroid_1212	1.72E-05
centroid_12121	conserved hypothetical protein	9	6	10	101	centroid_1212	22.9966206	1.62E-06	centroid_1212	1.72E-05
centroid_16551	conserved hypothetical protein	9	6	10	101	centroid_1655	22.9966206	1.62E-06	centroid_1655	1.72E-05
centroid_16552	imcF-related N-terminal domain protein	9	6	10	101	centroid_1655	22.9966206	1.62E-06	centroid_1655	1.72E-05
centroid_7244	acyl transferase domain protein	9	6	10	101	centroid_7244	22.9966206	1.62E-06	centroid_7244	1.72E-05
centroid_7411	conserved hypothetical protein	9	6	10	101	centroid_7411	22.9966206	1.62E-06	centroid_7411	1.72E-05
centroid_7412	conserved hypothetical protein	9	6	10	101	centroid_7412	22.9966206	1.62E-06	centroid_7412	1.72E-05
centroid_7414	gene 25-like lysozyme family protein	9	6	10	101	centroid_7414	22.9966206	1.62E-06	centroid_7414	1.72E-05
centroid_7415	conserved hypothetical protein	9	6	10	101	centroid_7415	22.9966206	1.62E-06	centroid_7415	1.72E-05

centroid_7416	conserved hypothetical protein	9	6	10	101	centroid_7416	22.9966206	1.62E-06	centroid_7416	1.72E-05
centroid_7417	conserved hypothetical protein	9	6	10	101	centroid_7417	22.9966206	1.62E-06	centroid_7417	1.72E-05
centroid_7418	PAAR motif family protein	9	6	10	101	centroid_7418	22.9966206	1.62E-06	centroid_7418	1.72E-05
centroid_7419	impA-related N-terminal family protein	9	6	10	101	centroid_7419	22.9966206	1.62E-06	centroid_7419	1.72E-05
centroid_7420	type VI secretion lipofamily protein	9	6	10	101	centroid_7420	22.9966206	1.62E-06	centroid_7420	1.72E-05
centroid_7422	type VI secretion ATPase, ClpV1 family	9	6	10	101	centroid_7422	22.9966206	1.62E-06	centroid_7422	1.72E-05
centroid_9144	type IV/VI secretion system , DotU family domain protein	9	6	10	101	centroid_9144	22.9966206	1.62E-06	centroid_9144	1.72E-05
centroid_14508	RHS repeat-associated core domain protein	9	9	10	98	centroid_1450	16.9430644	3.85E-05	centroid_1450	0.00012992
centroid_18330	RHS repeat-associated core domain protein	9	9	10	98	centroid_1833	16.9430644	3.85E-05	centroid_1833	0.00012992
centroid_7523	plasmid segregation protein ParM	9	9	10	98	centroid_7523	16.9430644	3.85E-05	centroid_7523	0.00012992
centroid_7524	plasmid stability family protein	9	9	10	98	centroid_7524	16.9430644	3.85E-05	centroid_7524	0.00012992
centroid_6025	outer membrane lipoprotein blc	9	10	10	97	centroid_6025	15.3678278	8.85E-05	centroid_6025	0.00022662
centroid_4319	alcohol dehydrogenase GroES-like domain protein	8	105	11	2	centroid_4319	48.846777	2.77E-12	centroid_4319	2.54E-09
centroid_13101	penicillin G acylase	8	0	11	107	centroid_1310	41.2864505	1.31E-10	centroid_1310	6.02E-08
centroid_16129	e kinase-, DNA gyrase B-, and HSP90-like ATPase family	8	0	11	107	centroid_1612	41.2864505	1.31E-10	centroid_1612	6.02E-08
centroid_5933	conserved hypothetical protein	8	0	11	107	centroid_5933	41.2864505	1.31E-10	centroid_5933	6.02E-08
centroid_15706	hypothetical protein	8	102	11	5	centroid_1570	36.5652769	1.48E-09	centroid_1570	1.16E-07
centroid_3381	alpha amylase, catalytic domain protein	8	102	11	5	centroid_3381	36.5652769	1.48E-09	centroid_3381	1.16E-07
centroid_11012	conserved hypothetical protein	8	1	11	106	centroid_1101	35.2603579	2.88E-09	centroid_1101	4.97E-07
centroid_11013	outer membrane insertion C-terminal signal domain protei	8	1	11	106	centroid_1101	35.2603579	2.88E-09	centroid_1101	4.97E-07
centroid_11014	esterase-like activity of phytase family protein	8	1	11	106	centroid_1101	35.2603579	2.88E-09	centroid_1101	4.97E-07
centroid_11015	pfkB carbohydrate kinase family protein	8	1	11	106	centroid_1101	35.2603579	2.88E-09	centroid_1101	4.97E-07
centroid_11016	SIS domain protein	8	1	11	106	centroid_1101	35.2603579	2.88E-09	centroid_1101	4.97E-07
centroid_11017	ADP-ribosylglycohydrolase family protein	8	1	11	106	centroid_1101	35.2603579	2.88E-09	centroid_1101	4.97E-07
centroid_11018	bacterial regulatory, gntR family protein	8	1	11	106	centroid_1101	35.2603579	2.88E-09	centroid_1101	4.97E-07
centroid_11019	MFS/sugar transport family protein	8	1	11	106	centroid_1101	35.2603579	2.88E-09	centroid_1101	4.97E-07
centroid_11020	MFS/sugar transport family protein	8	1	11	106	centroid_1102	35.2603579	2.88E-09	centroid_1102	4.97E-07
centroid_13089	esterase-like activity of phytase family protein	8	1	11	106	centroid_1308	35.2603579	2.88E-09	centroid_1308	4.97E-07
centroid_13090	(Glycoside-Pentoside-Hexuronide) transporter domain p	8	1	11	106	centroid_1309	35.2603579	2.88E-09	centroid_1309	4.97E-07
centroid_13254	ATP-binding region ATPase -containing domain protein	8	1	11	106	centroid_1325	35.2603579	2.88E-09	centroid_1325	4.97E-07
centroid_13277	putative 50S ribosomal protein L1	8	1	11	106	centroid_1327	35.2603579	2.88E-09	centroid_1327	4.97E-07
centroid_13278	esterase-like activity of phytase family protein	8	1	11	106	centroid_1327	35.2603579	2.88E-09	centroid_1327	4.97E-07
centroid_15935	ATP-binding region ATPase -containing domain protein	8	1	11	106	centroid_1593	35.2603579	2.88E-09	centroid_1593	4.97E-07
centroid_16094	phage integrase family protein	8	1	11	106	centroid_1609	35.2603579	2.88E-09	centroid_1609	4.97E-07
centroid_16130	phage integrase family protein	8	1	11	106	centroid_1613	35.2603579	2.88E-09	centroid_1613	4.97E-07
centroid_5869	ROS/MUCR transcriptional regulator family protein	8	1	11	106	centroid_5869	35.2603579	2.88E-09	centroid_5869	4.97E-07
centroid_5932	putative membrane protein	8	1	11	106	centroid_5932	35.2603579	2.88E-09	centroid_5932	4.97E-07
centroid_5974	conserved hypothetical protein	8	1	11	106	centroid_5974	35.2603579	2.88E-09	centroid_5974	4.97E-07
centroid_5975	conserved hypothetical protein	8	1	11	106	centroid_5975	35.2603579	2.88E-09	centroid_5975	4.97E-07
centroid_6493	prophage CP4-57 regulatory family protein	8	1	11	106	centroid_6493	35.2603579	2.88E-09	centroid_6493	4.97E-07
centroid_6818	fibronectin type III family protein	8	1	11	106	centroid_6818	35.2603579	2.88E-09	centroid_6818	4.97E-07
centroid_7389	impA domain family protein	8	1	11	106	centroid_7389	35.2603579	2.88E-09	centroid_7389	4.97E-07
centroid_5887	conserved hypothetical protein	8	2	11	105	centroid_5887	30.4557347	3.42E-08	centroid_5887	2.28E-06
centroid_5888	plasmid stability family protein	8	2	11	105	centroid_5888	30.4557347	3.42E-08	centroid_5888	2.28E-06
centroid_11002	putative transposase	8	3	11	104	centroid_1100	26.5398381	2.58E-07	centroid_1100	7.65E-06
centroid_11785	putative transposase	8	3	11	104	centroid_1178	26.5398381	2.58E-07	centroid_1178	7.65E-06
centroid_18222	n-negative pili assembly chaperone, C-terminal domain pr	8	3	11	104	centroid_1822	26.5398381	2.58E-07	centroid_1822	7.65E-06
centroid_6082	putative transposase	8	3	11	104	centroid_6082	26.5398381	2.58E-07	centroid_6082	7.65E-06
centroid_12122	hemolysin expression-modulating protein Hha	8	5	11	102	centroid_1212	20.5552241	5.79E-06	centroid_1212	5.00E-05
centroid_12265	RHS repeat-associated core domain protein	8	5	11	102	centroid_1226	20.5552241	5.79E-06	centroid_1226	5.00E-05
centroid_7429	avrPphF-ORF-2 family protein	8	5	11	102	centroid_7429	20.5552241	5.79E-06	centroid_7429	5.00E-05
centroid_9079	(Glycoside-Pentoside-Hexuronide) transporter domain p	8	5	11	102	centroid_9079	20.5552241	5.79E-06	centroid_9079	5.00E-05
centroid_9080	helix-turn-helix domain protein	8	5	11	102	centroid_9080	20.5552241	5.79E-06	centroid_9080	5.00E-05
centroid_9576	conserved hypothetical protein	8	5	11	102	centroid_9576	20.5552241	5.79E-06	centroid_9576	5.00E-05
centroid_5142	integrase core domain protein	8	94	11	13	centroid_5142	19.0309272	1.29E-05	centroid_5142	3.78E-05
centroid_14329	conserved hypothetical family protein	8	6	11	101	centroid_1432	18.2232846	1.96E-05	centroid_1432	0.0001066
centroid_5807	bacteriophage lambda tail assembly I family protein	8	6	11	101	centroid_5807	18.2232846	1.96E-05	centroid_5807	0.0001066
centroid_7366	phage late control gene D family protein	8	6	11	101	centroid_7366	18.2232846	1.96E-05	centroid_7366	0.0001066
centroid_7367	phage P2 GpU family protein	8	6	11	101	centroid_7367	18.2232846	1.96E-05	centroid_7367	0.0001066
centroid_7377	phage tail protein I	8	6	11	101	centroid_7377	18.2232846	1.96E-05	centroid_7377	0.0001066
centroid_9929	transposase family protein	8	93	11	14	centroid_9929	17.6507632	2.65E-05	centroid_9929	6.27E-05
centroid_5521	POTRA domain, ShlB-type family protein	8	92	11	15	centroid_5521	16.3820739	5.18E-05	centroid_5521	0.00010098
centroid_12694	bacteriophage lambda tail assembly I family protein	8	7	11	100	centroid_1269	16.2146209	5.66E-05	centroid_1269	0.00020876
centroid_14008	phage major tail tube protein	8	7	11	100	centroid_1400	16.2146209	5.66E-05	centroid_1400	0.00020876
centroid_14009	phage late control gene D family protein	8	7	11	100	centroid_1400	16.2146209	5.66E-05	centroid_1400	0.00020876
centroid_6796	conserved hypothetical protein	8	7	11	100	centroid_6796	16.2146209	5.66E-05	centroid_6796	0.00020876
centroid_7369	mu-like prophage FluMu gp41 family protein	8	7	11	100	centroid_7369	16.2146209	5.66E-05	centroid_7369	0.00020876
centroid_7370	phage major tail tube protein	8	7	11	100	centroid_7370	16.2146209	5.66E-05	centroid_7370	0.00020876
centroid_7371	phage tail sheath family protein	8	7	11	100	centroid_7371	16.2146209	5.66E-05	centroid_7371	0.00020876
centroid_958	pfkB carbohydrate kinase family protein	7	107	12	0	centroid_958	67.5426968	2.06E-16	centroid_958	2.59E-12
centroid_11278	aldo/keto reductase family protein	7	106	12	1	centroid_1127	60.9565398	5.83E-15	centroid_1127	3.17E-11
centroid_11408	zinc-binding dehydrogenase family protein	7	106	12	1	centroid_1140	60.9565398	5.83E-15	centroid_1140	3.17E-11
centroid_13575	pfkB carbohydrate kinase family protein	7	106	12	1	centroid_1357	60.9565398	5.83E-15	centroid_1357	3.17E-11
centroid_3166	major Facilitator Superfamily protein	7	106	12	1	centroid_3166	60.9565398	5.83E-15	centroid_3166	3.17E-11
centroid_3167	alcohol dehydrogenase GroES-like domain protein	7	106	12	1	centroid_3167	60.9565398	5.83E-15	centroid_3167	3.17E-11
centroid_3844	aldo/keto reductase family protein	7	106	12	1	centroid_3844	60.9565398	5.83E-15	centroid_3844	3.17E-11
centroid_3845	deoR-like helix-turn-helix domain protein	7	106	12	1	centroid_3845	60.9565398	5.83E-15	centroid_3845	3.17E-11
centroid_3846	inner membrane metabolite transport protein YdjE	7	106	12	1	centroid_3846	60.9565398	5.83E-15	centroid_3846	3.17E-11
centroid_4236	major Facilitator Superfamily protein	7	106	12	1	centroid_4236	60.9565398	5.83E-15	centroid_4236	3.17E-11
centroid_5297	pfkB carbohydrate kinase family protein	7	106	12	1	centroid_5297	60.9565398	5.83E-15	centroid_5297	3.17E-11
centroid_5512	sugar (and other) transporter family protein	7	106	12	1	centroid_5512	60.9565398	5.83E-15	centroid_5512	3.17E-11
centroid_7566	zinc-binding dehydrogenase family protein	7	106	12	1	centroid_7566	60.9565398	5.83E-15	centroid_7566	3.17E-11
centroid_7567	alcohol dehydrogenase GroES-like domain protein	7	106	12	1	centroid_7567	60.9565398	5.83E-15	centroid_7567	3.17E-11
centroid_9282	sugar (and other) transporter family protein	7	106	12	1	centroid_9282	60.9565398	5.83E-15	centroid_9282	3.17E-11
centroid_9283	sugar (and other) transporter family protein	7	106	12	1	centroid_9283	60.9565398	5.83E-15	centroid_9283	3.17E-11
centroid_12827	pfkB carbohydrate kinase family protein	7	105	12	2	centroid_1282	55.3167425	1.03E-13	centroid_1282	2.09E-10
centroid_3841	alcohol dehydrogenase GroES-like domain protein	7	105	12	2	centroid_3841	55.3167425	1.03E-13	centroid_3841	2.09E-10
centroid_3842	ketose-bisphosphate aldolase family protein	7	105	12	2	centroid_3842	55.3167425	1.03E-13	centroid_3842	2.09E-10
centroid_4320	zinc-binding dehydrogenase family protein	7	105	12	2	centroid_4320	55.3167425	1.03E-13	centroid_4320	2.09E-10
centroid_4916	conserved hypothetical protein	7	104	12	3	centroid_4916	50.4341713	1.23E-12	centroid_4916	9.87E-10
centroid_5511	conserved hypothetical protein	7	104	12	3	centroid_5511	50.4341713	1.23E-12	centroid_5511	9.87E-10
centroid_1012	sensory box protein	7	102	12	5	centroid_1012	42.4067049	7.41E-11	centroid_1012	1.19E-08
centroid_1013	response regulator	7	102	12	5	centroid_1013	42.4067049	7.41E-11	centroid_1013	1.19E-08
centroid_1014	acetate CoA-transferase subunit alpha	7	102	12	5	centroid_1014	42.4067049	7.41E-11	centroid_1014	1.19E-08
centroid_1015	acetate CoA-transferase subunit beta	7	102	12	5	centroid_1015	42.4067049	7.41E-11	centroid_1015	1.19E-08
centroid_1016	short-chain fatty acids transporter	7	102	12	5	centroid_1016	42.4067049	7.41E-11	centroid_1016	1.19E-08
centroid_1017	acetyl-CoA C-acetyltransferase family protein	7	102	12	5	centroid_1017	42.4067049	7.41E-11	centroid_1017	1.19E-08
centroid_10622	putative transposase DNA-binding domain protein	7	102	12	5	centroid_1062	42.4067049	7.41E-11	centroid_1062	1.19E-08
centroid_7693	ative signal transduction histidine-kinase atoS domain pro	7	102	12	5	centroid_7693	42.4067049	7.41E-11	centroid_7693	1.19E-08
centroid_7694	sensory box protein	7	102	12	5	centroid_7694	42.4067049	7.41E-11	centroid_7694	1.19E-08
centroid_8264	thiolase, N-terminal domain protein	7	102	12	5	centroid_8264	42.4067049	7.41E-11	centroid_8264	1.19E-08
centroid_8265	acetyl-CoA C-acetyltransferase family protein	7	102	12	5	centroid_8265	42.4067049	7.41E-11	centroid_8265	1.19E-08
centroid_17238	alpha amylase, catalytic domain protein	7	101	12	6	centroid_1723	39.0689867	4.09E-10	centroid_1723	3.36E-08
centroid_13252	fibronectin type III family protein	7	0	12	107	centroid_1325	35.0135702	3.27E-09	centroid_1325	5.97E-07
centroid_15179	RHS repeat-associated core domain protein	7	0	12	107	centroid_1517	35.0135702	3.27E-09	centroid_1517	5.97E-07
centroid_6837	hypothetical protein	7	0	12	107	centroid_6837	35.0135702	3.27E-09	centroid_6837	5.97E-07
centroid_14214	RHS repeat-associated core domain protein	7	1	12	106	centroid_1421	29.2087412	6.50E-08	centroid_1421	4.35E-06

centroid_5931	conserved hypothetical protein	7	1	12	106	centroid_5931	29.2087412	6.50E-08	centroid_5931	4.35E-06
centroid_7390	conserved hypothetical protein	7	1	12	106	centroid_7390	29.2087412	6.50E-08	centroid_7390	4.35E-06
centroid_7391	sigma-54 interaction domain protein	7	1	12	106	centroid_7391	29.2087412	6.50E-08	centroid_7391	4.35E-06
centroid_10179	CFA/I fimbrial subunit D	7	2	12	105	centroid_1017	24.7146695	6.65E-07	centroid_1017	1.79E-05
centroid_14398	conserved hypothetical protein	7	2	12	105	centroid_1439	24.7146695	6.65E-07	centroid_1439	1.79E-05
centroid_15118	conserved hypothetical protein	7	2	12	105	centroid_1511	24.7146695	6.65E-07	centroid_1511	1.79E-05
centroid_7398	RHS repeat-associated core domain protein	7	2	12	105	centroid_7398	24.7146695	6.65E-07	centroid_7398	1.79E-05
centroid_9014	outer membrane autotransporter barrel domain protein	7	2	12	105	centroid_9014	24.7146695	6.65E-07	centroid_9014	1.79E-05
centroid_9015	conserved hypothetical protein	7	2	12	105	centroid_9015	24.7146695	6.65E-07	centroid_9015	1.79E-05
centroid_9016	conserved hypothetical protein	7	2	12	105	centroid_9016	24.7146695	6.65E-07	centroid_9016	1.79E-05
centroid_9018	phage integrase family protein	7	2	12	105	centroid_9018	24.7146695	6.65E-07	centroid_9018	1.79E-05
centroid_9019	conserved hypothetical protein	7	2	12	105	centroid_9019	24.7146695	6.65E-07	centroid_9019	1.79E-05
centroid_6945	transposase DDE domain protein	7	93	12	14	centroid_6945	21.7403515	3.12E-06	centroid_6945	1.06E-05
centroid_8205	type VII secretion system (T7SS), usher family protein	7	93	12	14	centroid_8205	21.7403515	3.12E-06	centroid_8205	1.06E-05
centroid_12863	putative aTP-binding component of a transport system	7	3	12	104	centroid_1286	21.138612	4.27E-06	centroid_1286	5.42E-05
centroid_7542	RHS repeat-associated core domain protein	7	3	12	104	centroid_7542	21.138612	4.27E-06	centroid_7542	5.42E-05
centroid_12826	pfkB carbohydrate kinase family protein	7	92	12	15	centroid_1282	20.3135536	6.57E-06	centroid_1282	1.78E-05
centroid_3843	conserved hypothetical protein	7	91	12	16	centroid_3843	18.9927447	1.31E-05	centroid_3843	2.91E-05
centroid_4109	conserved hypothetical protein	7	89	12	18	centroid_4109	16.6271735	4.55E-05	centroid_4109	7.24E-05
centroid_13878	gram-negative porin family protein	7	5	12	102	centroid_1387	15.8242097	6.95E-05	centroid_1387	0.00029613
centroid_15850	putative conjugal transfer pilus assembly protein	7	5	12	102	centroid_1585	15.8242097	6.95E-05	centroid_1585	0.00029613
centroid_7236	exodeoxyribonuclease 8	7	5	12	102	centroid_7236	15.8242097	6.95E-05	centroid_7236	0.00029613
centroid_10619	conserved hypothetical protein	7	88	12	19	centroid_1061	15.5648439	7.97E-05	centroid_1061	0.00011038
centroid_12323	hypothetical protein	7	88	12	19	centroid_1232	15.5648439	7.97E-05	centroid_1232	0.00011038
centroid_4110	conserved hypothetical protein	7	88	12	19	centroid_4110	15.5648439	7.97E-05	centroid_4110	0.00011038
centroid_4977	conserved hypothetical protein	7	88	12	19	centroid_4977	15.5648439	7.97E-05	centroid_4977	0.00011038
centroid_14303	cyclic di-GMP phosphodiesterase YahA	6	104	13	3	centroid_1430	56.8868125	4.62E-14	centroid_1430	7.64E-11
centroid_6531	conserved hypothetical protein	6	95	13	12	centroid_6531	29.7000482	5.04E-08	centroid_6531	4.38E-07
centroid_10065	ash family protein	6	0	13	107	centroid_1006	28.8574397	7.79E-08	centroid_1006	5.51E-06
centroid_17227	ash family protein	6	0	13	107	centroid_1722	28.8574397	7.79E-08	centroid_1722	5.51E-06
centroid_17382	RHS repeat-associated core domain protein	6	0	13	107	centroid_1738	28.8574397	7.79E-08	centroid_1738	5.51E-06
centroid_5624	DKNYY family protein	6	0	13	107	centroid_5624	28.8574397	7.79E-08	centroid_5624	5.51E-06
centroid_5625	conserved hypothetical protein	6	0	13	107	centroid_5625	28.8574397	7.79E-08	centroid_5625	5.51E-06
centroid_5934	e kinase-, DNA gyrase B-, and HSP90-like ATPase family	6	0	13	107	centroid_5934	28.8574397	7.79E-08	centroid_5934	5.51E-06
centroid_10047	conserved hypothetical protein	6	1	13	106	centroid_1004	23.3326582	1.36E-06	centroid_1004	3.50E-05
centroid_10084	RHS repeat-associated core domain protein	6	1	13	106	centroid_1008	23.3326582	1.36E-06	centroid_1008	3.50E-05
centroid_13103	type-1 fimbrial protein, A chain	6	1	13	106	centroid_1310	23.3326582	1.36E-06	centroid_1310	3.50E-05
centroid_14463	RHS repeat-associated core domain protein	6	1	13	106	centroid_1446	23.3326582	1.36E-06	centroid_1446	3.50E-05
centroid_18590	hypothetical protein	6	1	13	106	centroid_1859	23.3326582	1.36E-06	centroid_1859	3.50E-05
centroid_5892	3-hydroxybutyrate dehydrogenase family protein	6	1	13	106	centroid_5892	23.3326582	1.36E-06	centroid_5892	3.50E-05
centroid_5893	citrate transporter family protein	6	1	13	106	centroid_5893	23.3326582	1.36E-06	centroid_5893	3.50E-05
centroid_5894	ydroxyacyl-CoA dehydrogenase, NAD binding domain prc	6	1	13	106	centroid_5894	23.3326582	1.36E-06	centroid_5894	3.50E-05
centroid_5895	acetyl-CoA C-acetyltransferase family protein	6	1	13	106	centroid_5895	23.3326582	1.36E-06	centroid_5895	3.50E-05
centroid_5896	3-oxoacid CoA-transferase, B subunit	6	1	13	106	centroid_5896	23.3326582	1.36E-06	centroid_5896	3.50E-05
centroid_5897	3-oxoacid CoA-transferase, A subunit	6	1	13	106	centroid_5897	23.3326582	1.36E-06	centroid_5897	3.50E-05
centroid_5898	bacterial regulatory helix-turn-helix , lysR family protein	6	1	13	106	centroid_5898	23.3326582	1.36E-06	centroid_5898	3.50E-05
centroid_6103	caudovirales tail fibre assembly family protein	6	1	13	106	centroid_6103	23.3326582	1.36E-06	centroid_6103	3.50E-05
centroid_11183	transposase DDE domain protein	6	91	13	16	centroid_1118	23.1027324	1.54E-06	centroid_1118	4.55E-06
centroid_9017	bacterial regulatory, luxR family protein	6	2	13	105	centroid_9017	19.2156775	1.17E-05	centroid_9017	0.00012687
centroid_17120	initiator Replication family protein	6	87	13	20	centroid_1712	18.1490105	2.04E-05	centroid_1712	3.04E-05
centroid_4867	repFIB replication protein A	6	86	13	21	centroid_4867	17.1000685	3.55E-05	centroid_4867	4.63E-05
centroid_14025	phage integrase family protein	6	85	13	22	centroid_1402	16.1141171	5.96E-05	centroid_1402	6.93E-05
centroid_10184	putative mu prophage; Tail fiber protein	6	3	13	104	centroid_1018	16.0378372	6.21E-05	centroid_1018	0.0003449
centroid_11749	transposase family protein	6	3	13	104	centroid_1174	16.0378372	6.21E-05	centroid_1174	0.0003449
centroid_6866	putative dNA topoisomerase III	6	3	13	104	centroid_6866	16.0378372	6.21E-05	centroid_6866	0.0003449
centroid_8025	integrase core domain protein	6	3	13	104	centroid_8025	16.0378372	6.21E-05	centroid_8025	0.0003449
centroid_8920	protein TolA	6	3	13	104	centroid_8920	16.0378372	6.21E-05	centroid_8920	0.0003449
centroid_8921	tolA C-terminal family protein	6	3	13	104	centroid_8921	16.0378372	6.21E-05	centroid_8921	0.0003449
centroid_2684	conserved hypothetical protein	5	104	14	3	centroid_2684	63.5120575	1.59E-15	centroid_2684	5.04E-12
centroid_2685	inner membrane protein YigG	5	104	14	3	centroid_2685	63.5120575	1.59E-15	centroid_2685	5.04E-12
centroid_2947	papC N-terminal domain protein	5	103	14	4	centroid_2947	58.8810871	1.67E-14	centroid_2947	2.17E-11
centroid_3520	putative predicted protein	5	90	14	17	centroid_3520	26.0230048	3.37E-07	centroid_3520	1.03E-06
centroid_3521	conserved hypothetical protein	5	90	14	17	centroid_3521	26.0230048	3.37E-07	centroid_3521	1.03E-06
centroid_5163	putative domain protein	5	90	14	17	centroid_5163	26.0230048	3.37E-07	centroid_5163	1.03E-06
centroid_5556	hypothetical protein	5	88	14	19	centroid_5556	23.2940413	1.39E-06	centroid_5556	2.88E-06
centroid_11585	AAA domain family protein	5	0	14	107	centroid_1158	22.822425	1.78E-06	centroid_1158	4.76E-05
centroid_11597	hcp domain protein	5	0	14	107	centroid_1159	22.822425	1.78E-06	centroid_1159	4.76E-05
centroid_11763	fibronectin type III family protein	5	0	14	107	centroid_1176	22.822425	1.78E-06	centroid_1176	4.76E-05
centroid_12872	prophage CP4-57 integrase	5	0	14	107	centroid_1287	22.822425	1.78E-06	centroid_1287	4.76E-05
centroid_13004	AAA domain family protein	5	0	14	107	centroid_1300	22.822425	1.78E-06	centroid_1300	4.76E-05
centroid_13005	C 5-methylcytosine restriction system component family p	5	0	14	107	centroid_1300	22.822425	1.78E-06	centroid_1300	4.76E-05
centroid_13006	conserved hypothetical protein	5	0	14	107	centroid_1300	22.822425	1.78E-06	centroid_1300	4.76E-05
centroid_13012	conserved hypothetical protein	5	0	14	107	centroid_1301	22.822425	1.78E-06	centroid_1301	4.76E-05
centroid_13013	conserved hypothetical protein	5	0	14	107	centroid_1301	22.822425	1.78E-06	centroid_1301	4.76E-05
centroid_13014	kinase domain protein	5	0	14	107	centroid_1301	22.822425	1.78E-06	centroid_1301	4.76E-05
centroid_13015	phage integrase family protein	5	0	14	107	centroid_1301	22.822425	1.78E-06	centroid_1301	4.76E-05
centroid_13470	conserved hypothetical protein	5	0	14	107	centroid_1347	22.822425	1.78E-06	centroid_1347	4.76E-05
centroid_15005	putative membrane protein	5	0	14	107	centroid_1500	22.822425	1.78E-06	centroid_1500	4.76E-05
centroid_15006	conserved hypothetical protein	5	0	14	107	centroid_1500	22.822425	1.78E-06	centroid_1500	4.76E-05
centroid_15016	conserved hypothetical protein	5	0	14	107	centroid_1501	22.822425	1.78E-06	centroid_1501	4.76E-05
centroid_17373	phage integrase family protein	5	0	14	107	centroid_1737	22.822425	1.78E-06	centroid_1737	4.76E-05
centroid_17374	conserved hypothetical protein	5	0	14	107	centroid_1737	22.822425	1.78E-06	centroid_1737	4.76E-05
centroid_17584	conserved hypothetical protein	5	0	14	107	centroid_1758	22.822425	1.78E-06	centroid_1758	4.76E-05
centroid_18529	conserved hypothetical protein	5	0	14	107	centroid_1852	22.822425	1.78E-06	centroid_1852	4.76E-05
centroid_5606	sigma-54 interaction domain protein	5	0	14	107	centroid_5606	22.822425	1.78E-06	centroid_5606	4.76E-05
centroid_5607	PRD domain protein	5	0	14	107	centroid_5607	22.822425	1.78E-06	centroid_5607	4.76E-05
centroid_5608	PTS system fructose IIA component family protein	5	0	14	107	centroid_5608	22.822425	1.78E-06	centroid_5608	4.76E-05
centroid_5609	PTS system sorbose subIIB component family protein	5	0	14	107	centroid_5609	22.822425	1.78E-06	centroid_5609	4.76E-05
centroid_5610	PTS system sorbose-specific iic component family protei	5	0	14	107	centroid_5610	22.822425	1.78E-06	centroid_5610	4.76E-05
centroid_5611	ystem mannose/fructose/sorbose IID component family p	5	0	14	107	centroid_5611	22.822425	1.78E-06	centroid_5611	4.76E-05
centroid_5612	L-seryl-tRNA selenium transferase family protein	5	0	14	107	centroid_5612	22.822425	1.78E-06	centroid_5612	4.76E-05
centroid_5613	2-dehydro-3-deoxyphosphogluconate aldolase	5	0	14	107	centroid_5613	22.822425	1.78E-06	centroid_5613	4.76E-05
centroid_5972	type IV/VI secretion system , DotU family domain protein	5	0	14	107	centroid_5972	22.822425	1.78E-06	centroid_5972	4.76E-05
centroid_5973	conserved hypothetical protein	5	0	14	107	centroid_5973	22.822425	1.78E-06	centroid_5973	4.76E-05
centroid_6022	conserved hypothetical protein	5	0	14	107	centroid_6022	22.822425	1.78E-06	centroid_6022	4.76E-05
centroid_11498	conserved hypothetical protein	5	87	14	20	centroid_1149	22.053186	2.65E-06	centroid_1149	4.65E-06
centroid_10649	conserved hypothetical protein	5	83	14	24	centroid_1064	17.763653	2.50E-05	centroid_1064	2.63E-05
centroid_9921	hypothetical protein	5	83	14	24	centroid_9921	17.763653	2.50E-05	centroid_9921	2.63E-05
centroid_10981	putative iSSod4, transposase	5	1	14	106	centroid_1098	17.6643261	2.64E-05	centroid_1098	0.00025813
centroid_11022	transposase family protein	5	1	14	106	centroid_1102	17.6643261	2.64E-05	centroid_1102	0.00025813
centroid_11023	ABC transporter family protein	5	1	14	106	centroid_1102	17.6643261	2.64E-05	centroid_1102	0.00025813
centroid_11024	putative transporter protein AatB	5	1	14	106	centroid_1102	17.6643261	2.64E-05	centroid_1102	0.00025813
centroid_11025	outer membrane efflux family protein	5	1	14	106	centroid_1102	17.6643261	2.64E-05	centroid_1102	0.00025813
centroid_11026	ftsX-like permease family protein	5	1	14	106	centroid_1102	17.6643261	2.64E-05	centroid_1102	0.00025813
centroid_11027	hypothetical protein	5	1	14	106	centroid_1102	17.6643261	2.64E-05	centroid_1102	0.00025813
centroid_11108	putative aatD, apolipoprotein N-acyltransferase	5	1	14	106	centroid_1110	17.6643261	2.64E-05	centroid_1110	0.00025813
centroid_11786	transposase, Mutator family protein	5	1	14	106	centroid_1178	17.6643261	2.64E-05	centroid_1178	0.00025813

centroid_13208	transposase, Mutator family protein	5	1	14	106	centroid_1320	17.6643261	2.64E-05	centroid_1320	0.00025813
centroid_13417	transposase, Mutator family protein	5	1	14	106	centroid_1341	17.6643261	2.64E-05	centroid_1341	0.00025813
centroid_15120	putative type II plasmid partioning protein	5	1	14	106	centroid_1512	17.6643261	2.64E-05	centroid_1512	0.00025813
centroid_6102	side tail fiber family protein	5	1	14	106	centroid_610	17.6643261	2.64E-05	centroid_610	0.00025813
centroid_7351	CRISPR type I-E/ECOLI-associated protein CasA/Cse1	5	1	14	106	centroid_735	17.6643261	2.64E-05	centroid_735	0.00025813
centroid_16544	rhs element Vgr family protein	5	81	14	26	centroid_1654	15.9534263	6.49E-05	centroid_1654	5.66E-05
centroid_8383	protein YoaG	4	106	15	1	centroid_838	81.6808607	1.60E-19	centroid_838	5.82E-15
centroid_11950	FHIPEP family protein	4	105	15	2	centroid_1195	75.6577497	3.37E-18	centroid_1195	4.78E-14
centroid_17737	acterial extracellular solute-binding, 5 Middle family protei	4	105	15	2	centroid_1773	75.6577497	3.37E-18	centroid_1773	4.78E-14
centroid_17738	acterial extracellular solute-binding, 5 Middle family protei	4	105	15	2	centroid_1773	75.6577497	3.37E-18	centroid_1773	4.78E-14
centroid_2746	putative binding protein YgiS	4	105	15	2	centroid_274	75.6577497	3.37E-18	centroid_274	4.78E-14
centroid_3231	conserved hypothetical protein	4	104	15	3	centroid_323	70.3055829	5.08E-17	centroid_323	2.77E-13
centroid_5319	conserved hypothetical protein	4	104	15	3	centroid_531	70.3055829	5.08E-17	centroid_531	2.77E-13
centroid_5432	conserved hypothetical protein	4	102	15	5	centroid_543	61.2116897	5.13E-15	centroid_543	4.90E-12
centroid_2637	conserved hypothetical protein	4	101	15	6	centroid_263	57.3166749	3.71E-14	centroid_263	1.66E-11
centroid_2747	antitoxin MqsA	4	101	15	6	centroid_274	57.3166749	3.71E-14	centroid_274	1.66E-11
centroid_10926	gyrI-like small molecule binding domain protein	4	94	15	13	centroid_1092	37.8781358	7.53E-10	centroid_1092	8.80E-09
centroid_13091	mRNA interferase MqsR	4	92	15	15	centroid_1309	34.0025301	5.50E-09	centroid_1309	3.38E-08
centroid_2748	mRNA interferase MqsR	4	92	15	15	centroid_274	34.0025301	5.50E-09	centroid_274	3.38E-08
centroid_13465	conserved hypothetical protein	4	90	15	17	centroid_1346	30.6170079	3.14E-08	centroid_1346	1.14E-07
centroid_9932	rhs core with extension domain protein	4	90	15	17	centroid_993	30.6170079	3.14E-08	centroid_993	1.14E-07
centroid_11130	conserved hypothetical protein	4	89	15	18	centroid_1113	29.0802925	6.94E-08	centroid_1113	2.00E-07
centroid_7022	putative transposase	4	86	15	21	centroid_702	24.9908264	5.76E-07	centroid_702	9.51E-07
centroid_10218	conserved hypothetical protein	4	84	15	23	centroid_1021	22.6303595	1.96E-06	centroid_1021	2.43E-06
centroid_12706	conserved hypothetical protein	4	83	15	24	centroid_1270	21.5431377	3.46E-06	centroid_1270	3.78E-06
centroid_14802	bacterial Ig-like domain family protein	4	79	15	28	centroid_1480	17.7145191	2.57E-05	centroid_1480	1.91E-05
centroid_16797	bacterial Ig-like domain family protein	4	79	15	28	centroid_1679	17.7145191	2.57E-05	centroid_1679	1.91E-05
centroid_4917	conserved hypothetical protein	4	79	15	28	centroid_491	17.7145191	2.57E-05	centroid_491	1.91E-05
centroid_10028	fimbrial family protein	4	0	15	107	centroid_1002	16.9199499	3.90E-05	centroid_1002	0.00038725
centroid_10029	-negative pili assembly chaperone, N-terminal domain pr	4	0	15	107	centroid_1002	16.9199499	3.90E-05	centroid_1002	0.00038725
centroid_10030	type VII secretion system (T7SS), usher family protein	4	0	15	107	centroid_1003	16.9199499	3.90E-05	centroid_1003	0.00038725
centroid_10031	fimbrial family protein	4	0	15	107	centroid_1003	16.9199499	3.90E-05	centroid_1003	0.00038725
centroid_10032	fimbrial family protein	4	0	15	107	centroid_1003	16.9199499	3.90E-05	centroid_1003	0.00038725
centroid_11610	CRISPR-associated family protein	4	0	15	107	centroid_1161	16.9199499	3.90E-05	centroid_1161	0.00038725
centroid_11777	RHS repeat-associated core domain protein	4	0	15	107	centroid_1177	16.9199499	3.90E-05	centroid_1177	0.00038725
centroid_13007	N-6 DNA Methylase family protein	4	0	15	107	centroid_1300	16.9199499	3.90E-05	centroid_1300	0.00038725
centroid_13009	conserved hypothetical protein	4	0	15	107	centroid_1300	16.9199499	3.90E-05	centroid_1300	0.00038725
centroid_13010	conserved hypothetical protein	4	0	15	107	centroid_1301	16.9199499	3.90E-05	centroid_1301	0.00038725
centroid_13011	conserved hypothetical protein	4	0	15	107	centroid_1301	16.9199499	3.90E-05	centroid_1301	0.00038725
centroid_13018	conserved hypothetical protein	4	0	15	107	centroid_1301	16.9199499	3.90E-05	centroid_1301	0.00038725
centroid_13063	conserved hypothetical protein	4	0	15	107	centroid_1306	16.9199499	3.90E-05	centroid_1306	0.00038725
centroid_13073	putative dNA-damage-inducible protein	4	0	15	107	centroid_1307	16.9199499	3.90E-05	centroid_1307	0.00038725
centroid_13113	glycosyl transferases group 1 family protein	4	0	15	107	centroid_1311	16.9199499	3.90E-05	centroid_1311	0.00038725
centroid_13365	type VI secretion ATPase, ClpV1 family	4	0	15	107	centroid_1336	16.9199499	3.90E-05	centroid_1336	0.00038725
centroid_13479	phage integrase family protein	4	0	15	107	centroid_1347	16.9199499	3.90E-05	centroid_1347	0.00038725
centroid_14637	bacteriophage abortive infection AbiH family protein	4	0	15	107	centroid_1463	16.9199499	3.90E-05	centroid_1463	0.00038725
centroid_14929	conserved hypothetical protein	4	0	15	107	centroid_1492	16.9199499	3.90E-05	centroid_1492	0.00038725
centroid_14949	conserved hypothetical protein	4	0	15	107	centroid_1494	16.9199499	3.90E-05	centroid_1494	0.00038725
centroid_14950	conserved hypothetical protein	4	0	15	107	centroid_1495	16.9199499	3.90E-05	centroid_1495	0.00038725
centroid_15115	conserved hypothetical protein	4	0	15	107	centroid_1511	16.9199499	3.90E-05	centroid_1511	0.00038725
centroid_15194	RHS repeat-associated core domain protein	4	0	15	107	centroid_1519	16.9199499	3.90E-05	centroid_1519	0.00038725
centroid_15195	conserved hypothetical protein	4	0	15	107	centroid_1519	16.9199499	3.90E-05	centroid_1519	0.00038725
centroid_15535	conserved hypothetical protein	4	0	15	107	centroid_1553	16.9199499	3.90E-05	centroid_1553	0.00038725
centroid_15536	rhs element Vgr family protein	4	0	15	107	centroid_1553	16.9199499	3.90E-05	centroid_1553	0.00038725
centroid_15541	glycosyl transferases group 1 family protein	4	0	15	107	centroid_1554	16.9199499	3.90E-05	centroid_1554	0.00038725
centroid_15542	glycosyl transferases group 1 family protein	4	0	15	107	centroid_1554	16.9199499	3.90E-05	centroid_1554	0.00038725
centroid_15792	poxvirus D5 protein-like family protein	4	0	15	107	centroid_1579	16.9199499	3.90E-05	centroid_1579	0.00038725
centroid_15833	fimbrial family protein	4	0	15	107	centroid_1583	16.9199499	3.90E-05	centroid_1583	0.00038725
centroid_15870	conserved hypothetical protein	4	0	15	107	centroid_1587	16.9199499	3.90E-05	centroid_1587	0.00038725
centroid_17237	fibronectin type III family protein	4	0	15	107	centroid_1723	16.9199499	3.90E-05	centroid_1723	0.00038725
centroid_17364	dhesin biosynthesis transcription regulatory family protei	4	0	15	107	centroid_1736	16.9199499	3.90E-05	centroid_1736	0.00038725
centroid_17593	conserved hypothetical protein	4	0	15	107	centroid_1759	16.9199499	3.90E-05	centroid_1759	0.00038725
centroid_17595	RHS repeat-associated core domain protein	4	0	15	107	centroid_1759	16.9199499	3.90E-05	centroid_1759	0.00038725
centroid_18333	conserved hypothetical protein	4	0	15	107	centroid_1833	16.9199499	3.90E-05	centroid_1833	0.00038725
centroid_18397	conserved hypothetical protein	4	0	15	107	centroid_1839	16.9199499	3.90E-05	centroid_1839	0.00038725
centroid_5583	putative addiction module antidote protein	4	0	15	107	centroid_558	16.9199499	3.90E-05	centroid_558	0.00038725
centroid_5584	conserved hypothetical protein	4	0	15	107	centroid_558	16.9199499	3.90E-05	centroid_558	0.00038725
centroid_5585	SIR2-like domain protein	4	0	15	107	centroid_558	16.9199499	3.90E-05	centroid_558	0.00038725
centroid_5586	traC-like family protein	4	0	15	107	centroid_558	16.9199499	3.90E-05	centroid_558	0.00038725
centroid_5587	conserved hypothetical protein	4	0	15	107	centroid_558	16.9199499	3.90E-05	centroid_558	0.00038725
centroid_5588	P-type conjugative transfer protein TrbJ	4	0	15	107	centroid_558	16.9199499	3.90E-05	centroid_558	0.00038725
centroid_5589	hypothetical protein	4	0	15	107	centroid_558	16.9199499	3.90E-05	centroid_558	0.00038725
centroid_5590	P-type conjugative transfer protein TrbL	4	0	15	107	centroid_559	16.9199499	3.90E-05	centroid_559	0.00038725
centroid_5591	hypothetical protein	4	0	15	107	centroid_559	16.9199499	3.90E-05	centroid_559	0.00038725
centroid_5592	superfamily I DNA and RNA helicase	4	0	15	107	centroid_559	16.9199499	3.90E-05	centroid_559	0.00038725
centroid_5593	replication initiator A family protein	4	0	15	107	centroid_559	16.9199499	3.90E-05	centroid_559	0.00038725
centroid_5594	prophage CP4-57 regulatory family protein	4	0	15	107	centroid_559	16.9199499	3.90E-05	centroid_559	0.00038725
centroid_5595	phage integrase family protein	4	0	15	107	centroid_559	16.9199499	3.90E-05	centroid_559	0.00038725
centroid_5734	phage terminase, small subunit, P27 family	4	0	15	107	centroid_573	16.9199499	3.90E-05	centroid_573	0.00038725
centroid_5735	phage Terminase family protein	4	0	15	107	centroid_573	16.9199499	3.90E-05	centroid_573	0.00038725
centroid_5784	hypothetical protein	4	0	15	107	centroid_578	16.9199499	3.90E-05	centroid_578	0.00038725
centroid_5785	conserved hypothetical protein	4	0	15	107	centroid_578	16.9199499	3.90E-05	centroid_578	0.00038725
centroid_5835	CRISPR-associated endonuclease Cas1	4	0	15	107	centroid_583	16.9199499	3.90E-05	centroid_583	0.00038725
centroid_5836	CRISPR-associated helicase Cas3, subtype I-F/YPEST	4	0	15	107	centroid_583	16.9199499	3.90E-05	centroid_583	0.00038725
centroid_5837	CRISPR-associated family protein	4	0	15	107	centroid_583	16.9199499	3.90E-05	centroid_583	0.00038725
centroid_5838	CRISPR-associated protein Csy2	4	0	15	107	centroid_583	16.9199499	3.90E-05	centroid_583	0.00038725
centroid_5839	CRISPR-associated protein Csy3	4	0	15	107	centroid_583	16.9199499	3.90E-05	centroid_583	0.00038725
centroid_5840	CRISPR-associated endonuclease Cas6/Csy4	4	0	15	107	centroid_584	16.9199499	3.90E-05	centroid_584	0.00038725
centroid_5966	conserved hypothetical protein	4	0	15	107	centroid_596	16.9199499	3.90E-05	centroid_596	0.00038725
centroid_5967	conserved hypothetical protein	4	0	15	107	centroid_596	16.9199499	3.90E-05	centroid_596	0.00038725
centroid_5968	transglycosylase SLT domain protein	4	0	15	107	centroid_596	16.9199499	3.90E-05	centroid_596	0.00038725
centroid_5969	type VI secretion ATPase, ClpV1 family	4	0	15	107	centroid_596	16.9199499	3.90E-05	centroid_596	0.00038725
centroid_5970	type VI secretion system effector, Hcp1 family protein	4	0	15	107	centroid_597	16.9199499	3.90E-05	centroid_597	0.00038725
centroid_5971	ompA family protein	4	0	15	107	centroid_597	16.9199499	3.90E-05	centroid_597	0.00038725
centroid_5989	putative predicted protein	4	0	15	107	centroid_598	16.9199499	3.90E-05	centroid_598	0.00038725
centroid_6009	conserved hypothetical protein	4	0	15	107	centroid_600	16.9199499	3.90E-05	centroid_600	0.00038725
centroid_11968	bacterial Ig-like domain family protein	4	77	15	30	centroid_1196	16.0645352	6.12E-05	centroid_1196	3.98E-05
centroid_12473	bacterial Ig-like domain family protein	4	77	15	30	centroid_1247	16.0645352	6.12E-05	centroid_1247	3.98E-05
centroid_7843	hage tail tape measure protein, TP901 family, core regior	4	77	15	30	centroid_784	16.0645352	6.12E-05	centroid_784	3.98E-05
centroid_18009	bacterial Ig-like domain family protein	4	76	15	31	centroid_1800	15.2957631	9.19E-05	centroid_1800	5.64E-05
centroid_9965	biquinone/plastoquinone (complex I), various chains fami	3	104	16	3	centroid_996	77.2648409	1.50E-18	centroid_996	1.22E-14
centroid_13670	family 4 glycosyl hydrolase C-terminal domain protein	3	91	16	16	centroid_1367	37.2734767	1.03E-09	centroid_1367	5.23E-09
centroid_4439	family 4 glycosyl hydrolase C-terminal domain protein	3	91	16	16	centroid_443	37.2734767	1.03E-09	centroid_443	5.23E-09
centroid_2084	6-phospho-alpha-glucosidase	3	90	16	17	centroid_208	35.5077641	2.54E-09	centroid_208	9.84E-09
centroid_4438	4 glycosyl hydrolase family protein	3	90	16	17	centroid_443	35.5077641	2.54E-09	centroid_443	9.84E-09
centroid_14160	conserved domain protein	3	78	16	29	centroid_1416	20.4993606	5.97E-06	centroid_1416	3.70E-06
centroid_4432	caudovirales tail fibre assembly family protein	3	78	16	29	centroid_443	20.4993606	5.97E-06	centroid_443	3.70E-06

centroid_4849	conserved hypothetical protein	3	78	16	29	centroid_4849	20.4993606	5.97E-06	centroid_4849	3.70E-06
centroid_10333	tive pilus biosynthesis transmembrane anchor domain pr	3	77	16	30	centroid_1033	19.6077715	9.51E-06	centroid_1033	5.49E-06
centroid_10334	tive pilus biosynthesis transmembrane anchor domain pr	3	77	16	30	centroid_1033	19.6077715	9.51E-06	centroid_1033	5.49E-06
centroid_10335	hypothetical protein	3	77	16	30	centroid_1033	19.6077715	9.51E-06	centroid_1033	5.49E-06
centroid_12709	bacterial regulatory helix-turn-helix, AraC family protein	3	77	16	30	centroid_1270	19.6077715	9.51E-06	centroid_1270	5.49E-06
centroid_14026	type II/IV secretion system family protein	3	77	16	30	centroid_1402	19.6077715	9.51E-06	centroid_1402	5.49E-06
centroid_14191	CFA/I fimbrial subunit D domain protein	3	77	16	30	centroid_1419	19.6077715	9.51E-06	centroid_1419	5.49E-06
centroid_4850	type IV leader peptidase family protein	3	77	16	30	centroid_4850	19.6077715	9.51E-06	centroid_4850	5.49E-06
centroid_4851	putative pilus biosythesis protein	3	77	16	30	centroid_4851	19.6077715	9.51E-06	centroid_4851	5.49E-06
centroid_4852	type II secretion system (T2SS), F family protein	3	77	16	30	centroid_4852	19.6077715	9.51E-06	centroid_4852	5.49E-06
centroid_4853	type II/IV secretion system family protein	3	77	16	30	centroid_4853	19.6077715	9.51E-06	centroid_4853	5.49E-06
centroid_4854	putative lngG	3	77	16	30	centroid_4854	19.6077715	9.51E-06	centroid_4854	5.49E-06
centroid_4856	putative pilus biosynthesis transmembrane anchor protein	3	77	16	30	centroid_4856	19.6077715	9.51E-06	centroid_4856	5.49E-06
centroid_4858	toxin co-regulated pilus biosynthesis Q family protein	3	77	16	30	centroid_4858	19.6077715	9.51E-06	centroid_4858	5.49E-06
centroid_4859	repilin-type N-terminal cleavage/methylation domain prote	3	77	16	30	centroid_4859	19.6077715	9.51E-06	centroid_4859	5.49E-06
centroid_4861	putative lngX2	3	77	16	30	centroid_4861	19.6077715	9.51E-06	centroid_4861	5.49E-06
centroid_4862	transglycosylase SLT domain protein	3	77	16	30	centroid_4862	19.6077715	9.51E-06	centroid_4862	5.49E-06
centroid_4863	bacterial regulatory helix-turn-helix, AraC family protein	3	77	16	30	centroid_4863	19.6077715	9.51E-06	centroid_4863	5.49E-06
centroid_4864	dhesin biosynthesis transcription regulatory family protei	3	77	16	30	centroid_4864	19.6077715	9.51E-06	centroid_4864	5.49E-06
centroid_8277	bacterial type II and III secretion system family protein	3	77	16	30	centroid_8277	19.6077715	9.51E-06	centroid_8277	5.49E-06
centroid_4203	CFA/I fimbrial subunit D	3	76	16	31	centroid_4203	18.7551243	1.49E-05	centroid_4203	8.03E-06
centroid_4855	putative pilus biosynthesis protein	3	76	16	31	centroid_4855	18.7551243	1.49E-05	centroid_4855	8.03E-06
centroid_4857	bacterial type II and III secretion system family protein	3	76	16	31	centroid_4857	18.7551243	1.49E-05	centroid_4857	8.03E-06
centroid_14832	putative bifunctional chitinase/lysozyme domain protein	2	103	17	4	centroid_1483	79.3310379	5.25E-19	centroid_1483	2.06E-15
centroid_8579	putative bifunctional chitinase/lysozyme domain protein	2	103	17	4	centroid_8579	79.3310379	5.25E-19	centroid_8579	2.06E-15
centroid_2542	helix-turn-helix domain protein	2	100	17	7	centroid_2542	66.6897044	3.18E-16	centroid_2542	1.13E-13
centroid_3555	integrase core domain protein	2	100	17	7	centroid_3555	66.6897044	3.18E-16	centroid_3555	1.13E-13
centroid_5353	integrase core domain protein	2	99	17	8	centroid_5353	63.1510407	1.91E-15	centroid_5353	3.47E-13
centroid_7156	putative transposase	2	86	17	21	centroid_7156	34.1292381	5.16E-09	centroid_7156	7.12E-09
centroid_8329	putative iS91 transposase	2	81	17	26	centroid_8329	27.6570165	1.45E-07	centroid_8329	9.34E-08
centroid_9765	aatD, apolipoN-acyltransferase domain protein	2	77	17	30	centroid_9765	23.4788918	1.26E-06	centroid_9765	5.54E-07
centroid_13568	putative aatD, apolipoprotein N-acyltransferase	2	76	17	31	centroid_1356	22.5444185	2.05E-06	centroid_1356	8.38E-07
centroid_5525	conserved hypothetical protein	2	76	17	31	centroid_5525	22.5444185	2.05E-06	centroid_5525	8.38E-07
centroid_14468	putative aatD, apolipoprotein N-acyltransferase	2	73	17	34	centroid_1446	19.9640057	7.89E-06	centroid_1446	2.71E-06
centroid_6217	lasmic binding and sugar binding domain of LacI family pr	2	73	17	34	centroid_6217	19.9640057	7.89E-06	centroid_6217	2.71E-06
centroid_733	periplasmic binding domain protein	2	73	17	34	centroid_733	19.9640057	7.89E-06	centroid_733	2.71E-06
centroid_734	D-allose transporter subunit domain protein	2	73	17	34	centroid_734	19.9640057	7.89E-06	centroid_734	2.71E-06
centroid_735	ABC transporter family protein	2	73	17	34	centroid_735	19.9640057	7.89E-06	centroid_735	2.71E-06
centroid_9859	hypothetical protein	2	73	17	34	centroid_9859	19.9640057	7.89E-06	centroid_9859	2.71E-06
centroid_15730	putative membrane protein	2	72	17	35	centroid_1573	19.1711252	1.20E-05	centroid_1573	3.93E-06
centroid_4206	putative membrane protein	2	72	17	35	centroid_4206	19.1711252	1.20E-05	centroid_4206	3.93E-06
centroid_8347	aatD, apolipoN-acyltransferase domain protein	2	72	17	35	centroid_8347	19.1711252	1.20E-05	centroid_8347	3.93E-06
centroid_9257	protein DedA	1	106	18	1	centroid_9257	103.661532	2.40E-24	centroid_9257	1.28E-19
centroid_17467	type II secretion system protein L	1	101	18	6	centroid_1746	77.4464244	1.36E-18	centroid_1746	8.66E-16
centroid_17801	putative membrane protein	1	101	18	6	centroid_1780	77.4464244	1.36E-18	centroid_1780	8.66E-16
centroid_1781	inner membrane protein YmfA	1	101	18	6	centroid_1781	77.4464244	1.36E-18	centroid_1781	8.66E-16
centroid_3084	BFD-like [2Fe-2S] binding domain protein	1	101	18	6	centroid_3084	77.4464244	1.36E-18	centroid_3084	8.66E-16
centroid_3085	bacterioferritin	1	101	18	6	centroid_3085	77.4464244	1.36E-18	centroid_3085	8.66E-16
centroid_3086	e 4 prepilin-like proteins leader peptide-processing enzy	1	101	18	6	centroid_3086	77.4464244	1.36E-18	centroid_3086	8.66E-16
centroid_3087	type II secretion system (T2SS), M family protein	1	101	18	6	centroid_3087	77.4464244	1.36E-18	centroid_3087	8.66E-16
centroid_3098	AAA domain protein	1	101	18	6	centroid_3098	77.4464244	1.36E-18	centroid_3098	8.66E-16
centroid_3099	putative peptidoglycan binding domain protein	1	101	18	6	centroid_3099	77.4464244	1.36E-18	centroid_3099	8.66E-16
centroid_3100	lcium-binding protein required for initiation of chromosome	1	101	18	6	centroid_3100	77.4464244	1.36E-18	centroid_3100	8.66E-16
centroid_5500	gspL periplasmic domain protein	1	101	18	6	centroid_5500	77.4464244	1.36E-18	centroid_5500	8.66E-16
centroid_8581	putative general secretion pathway protein A	1	101	18	6	centroid_8581	77.4464244	1.36E-18	centroid_8581	8.66E-16
centroid_9733	conserved hypothetical protein	1	101	18	6	centroid_9733	77.4464244	1.36E-18	centroid_9733	8.66E-16
centroid_9734	hypothetical protein	1	101	18	6	centroid_9734	77.4464244	1.36E-18	centroid_9734	8.66E-16
centroid_10869	type II secretion system protein H	1	100	18	7	centroid_1086	73.4622102	1.03E-17	centroid_1086	3.06E-15
centroid_12074	type II secretion system protein H	1	100	18	7	centroid_1207	73.4622102	1.03E-17	centroid_1207	3.06E-15
centroid_17465	type II secretion system (T2SS), F family protein	1	100	18	7	centroid_1746	73.4622102	1.03E-17	centroid_1746	3.06E-15
centroid_17466	type II secretion system (T2SS), F family protein	1	100	18	7	centroid_1746	73.4622102	1.03E-17	centroid_1746	3.06E-15
centroid_3083	putative bifunctional chitinase/lysozyme	1	100	18	7	centroid_3083	73.4622102	1.03E-17	centroid_3083	3.06E-15
centroid_3089	type II secretion system (T2SS), K family protein	1	100	18	7	centroid_3089	73.4622102	1.03E-17	centroid_3089	3.06E-15
centroid_3090	repilin-type N-terminal cleavage/methylation domain prote	1	100	18	7	centroid_3090	73.4622102	1.03E-17	centroid_3090	3.06E-15
centroid_3091	type II secretion system protein I	1	100	18	7	centroid_3091	73.4622102	1.03E-17	centroid_3091	3.06E-15
centroid_3092	type II secretion system protein H	1	100	18	7	centroid_3092	73.4622102	1.03E-17	centroid_3092	3.06E-15
centroid_3093	type II secretion system protein G	1	100	18	7	centroid_3093	73.4622102	1.03E-17	centroid_3093	3.06E-15
centroid_3094	type II secretion system protein F	1	100	18	7	centroid_3094	73.4622102	1.03E-17	centroid_3094	3.06E-15
centroid_3095	type II secretion system protein E	1	100	18	7	centroid_3095	73.4622102	1.03E-17	centroid_3095	3.06E-15
centroid_4742	repilin-type N-terminal cleavage/methylation domain prote	1	100	18	7	centroid_4742	73.4622102	1.03E-17	centroid_4742	3.06E-15
centroid_7722	type II secretion system protein F	1	100	18	7	centroid_7722	73.4622102	1.03E-17	centroid_7722	3.06E-15
centroid_7723	putative type II secretion system F domain protein	1	100	18	7	centroid_7723	73.4622102	1.03E-17	centroid_7723	3.06E-15
centroid_8069	type II secretion system protein D	1	100	18	7	centroid_8069	73.4622102	1.03E-17	centroid_8069	3.06E-15
centroid_8070	type II secretion system protein L	1	100	18	7	centroid_8070	73.4622102	1.03E-17	centroid_8070	3.06E-15
centroid_8580	carbohydrate binding domain protein	1	100	18	7	centroid_8580	73.4622102	1.03E-17	centroid_8580	3.06E-15
centroid_3096	type II secretion system protein D	1	98	18	9	centroid_3096	66.3796449	3.72E-16	centroid_3096	2.93E-14
centroid_8068	type II secretion system D domain protein	1	98	18	9	centroid_8068	66.3796449	3.72E-16	centroid_8068	2.93E-14
centroid_3097	type II secretion system protein C	1	97	18	10	centroid_3097	63.2180275	1.85E-15	centroid_3097	8.13E-14
centroid_2331	CRISPR-associated endonuclease Cas1	1	91	18	16	centroid_2331	48.1569144	3.93E-12	centroid_2331	1.29E-11
centroid_4611	SPR-associated endoribonuclease Cas2, subtype I-E/EC	1	91	18	16	centroid_4611	48.1569144	3.93E-12	centroid_4611	1.29E-11
centroid_9502	SPR-associated endoribonuclease Cas2, subtype I-E/EC	1	91	18	16	centroid_9502	48.1569144	3.93E-12	centroid_9502	1.29E-11
centroid_10582	SPR-associated protein Cas6/Cse3/CasE, subtype I-E/E(	1	89	18	18	centroid_1058	44.253394	2.89E-11	centroid_1058	5.19E-11
centroid_17493	hypothetical protein	1	88	18	19	centroid_1749	42.4601282	7.21E-11	centroid_1749	1.00E-10
centroid_13505	RHS repeat-associated core domain protein	1	87	18	20	centroid_1350	40.7614101	1.72E-10	centroid_1350	1.88E-10
centroid_3088	type II secretion system protein L	1	87	18	20	centroid_3088	40.7614101	1.72E-10	centroid_3088	1.88E-10
centroid_2332	SPR-associated protein Cas6/Cse3/CasE, subtype I-E/E(	1	83	18	24	centroid_2332	34.7770536	3.70E-09	centroid_2332	1.90E-09
centroid_9256	ulp1 protease family, C-terminal catalytic domain protein	1	77	18	30	centroid_9256	27.6754299	1.43E-07	centroid_9256	3.66E-08
centroid_17455	conserved hypothetical protein	1	74	18	33	centroid_1745	24.7536171	6.51E-07	centroid_1745	1.35E-07
centroid_4699	conserved hypothetical protein	1	74	18	33	centroid_4699	24.7536171	6.51E-07	centroid_4699	1.35E-07
centroid_7700	mRNA interferase HigB	1	74	18	33	centroid_7700	24.7536171	6.51E-07	centroid_7700	1.35E-07
centroid_16886	orn/Lys/Arg decarboxylase, major domain protein	1	73	18	34	centroid_1688	23.8549911	1.04E-06	centroid_1688	2.03E-07
centroid_17756	conserved hypothetical protein	1	73	18	34	centroid_1775	23.8549911	1.04E-06	centroid_1775	2.03E-07
centroid_1092	mRNA interferase HigB	1	69	18	38	centroid_1092	20.5834604	5.71E-06	centroid_1092	9.62E-07
centroid_9677	putative transposase	1	67	18	40	centroid_9677	19.1182125	1.23E-05	centroid_9677	1.98E-06
centroid_3558	caudovirales tail fibre assembly family protein	1	65	18	42	centroid_3558	17.7515874	2.52E-05	centroid_3558	5.06E-06
centroid_14007	RhsB domain protein	1	63	18	44	centroid_1400	16.4741263	4.93E-05	centroid_1400	8.26E-06
centroid_14128	e kinase-, DNA gyrase B-, and HSP90-like ATPase family	1	63	18	44	centroid_1412	16.4741263	4.93E-05	centroid_1412	8.26E-06
centroid_3474	putative copper-binding protein PcoE	1	63	18	44	centroid_3474	16.4741263	4.93E-05	centroid_3474	8.26E-06
centroid_373	conserved hypothetical protein	1	63	18	44	centroid_373	16.4741263	4.93E-05	centroid_373	8.26E-06
centroid_8305	RHS repeat-associated core domain protein	1	63	18	44	centroid_8305	16.4741263	4.93E-05	centroid_8305	8.26E-06
centroid_9920	RHS repeat-associated core domain protein	1	63	18	44	centroid_9920	16.4741263	4.93E-05	centroid_9920	8.26E-06
centroid_10828	putative metalloprotease YebA domain protein	1	62	18	45	centroid_1082	15.8662076	6.80E-05	centroid_1082	2.13E-05
centroid_10911	tnsA endonuclease N terminal family protein	1	62	18	45	centroid_1091	15.8662076	6.80E-05	centroid_1091	2.13E-05
centroid_10928	peptidase M23 family protein	1	62	18	45	centroid_1092	15.8662076	6.80E-05	centroid_1092	2.13E-05
centroid_11449	putative tn7-like transposition protein C	1	62	18	45	centroid_1144	15.8662076	6.80E-05	centroid_1144	2.13E-05
centroid_12338	conserved hypothetical protein	1	62	18	45	centroid_1233	15.8662076	6.80E-05	centroid_1233	2.13E-05
centroid_18160	putative pcoS	1	62	18	45	centroid_1816	15.8662076	6.80E-05	centroid_1816	2.13E-05

centroid_18161	efflux transporter, RND family, MFP subunit	1	62	18	45	centroid_1816	15.8662076	6.80E-05	centroid_1816	2.13E-05
centroid_18179	heavy metal sensor kinase family protein	1	62	18	45	centroid_1817	15.8662076	6.80E-05	centroid_1817	2.13E-05
centroid_3476	response regulator	1	62	18	45	centroid_3476	15.8662076	6.80E-05	centroid_3476	2.13E-05
centroid_3477	copper resistance D family protein	1	62	18	45	centroid_3477	15.8662076	6.80E-05	centroid_3477	2.13E-05
centroid_3478	copper resistance protein C	1	62	18	45	centroid_3478	15.8662076	6.80E-05	centroid_3478	2.13E-05
centroid_3479	copper resistance protein B	1	62	18	45	centroid_3479	15.8662076	6.80E-05	centroid_3479	2.13E-05
centroid_3480	copper resistance protein A	1	62	18	45	centroid_3480	15.8662076	6.80E-05	centroid_3480	2.13E-05
centroid_3481	putative copper resistant protein PcoE	1	62	18	45	centroid_3481	15.8662076	6.80E-05	centroid_3481	2.13E-05
centroid_3483	conserved hypothetical protein	1	62	18	45	centroid_3483	15.8662076	6.80E-05	centroid_3483	2.13E-05
centroid_3484	copper-translocating P-type ATPase	1	62	18	45	centroid_3484	15.8662076	6.80E-05	centroid_3484	2.13E-05
centroid_4049	tniQ family protein	1	62	18	45	centroid_4049	15.8662076	6.80E-05	centroid_4049	2.13E-05
centroid_3475	heavy metal sensor kinase family protein	1	61	18	46	centroid_3475	15.2775631	9.28E-05	centroid_3475	2.23E-05
centroid_3486	cation efflux system protein CusA	1	61	18	46	centroid_3486	15.2775631	9.28E-05	centroid_3486	2.23E-05
centroid_3487	efflux transporter, RND family, MFP subunit	1	61	18	46	centroid_3487	15.2775631	9.28E-05	centroid_3487	2.23E-05
centroid_3488	cation efflux system protein CusF	1	61	18	46	centroid_3488	15.2775631	9.28E-05	centroid_3488	2.23E-05
centroid_3489	nsporter, outer membrane factor (OMF) lipo, NodT family	1	61	18	46	centroid_3489	15.2775631	9.28E-05	centroid_3489	2.23E-05
centroid_3490	transcriptional regulatory protein CusR	1	61	18	46	centroid_3490	15.2775631	9.28E-05	centroid_3490	2.23E-05
centroid_3491	heavy metal sensor kinase family protein	1	61	18	46	centroid_3491	15.2775631	9.28E-05	centroid_3491	2.23E-05
centroid_5187	conserved hypothetical protein	1	61	18	46	centroid_5187	15.2775631	9.28E-05	centroid_5187	2.23E-05
centroid_1113	inner membrane protein YhaI	0	106	19	1	centroid_1113	111.278649	5.14E-26	centroid_1113	1.26E-21
centroid_1340	conserved hypothetical protein	0	106	19	1	centroid_1340	111.278649	5.14E-26	centroid_1340	1.26E-21
centroid_14713	conserved hypothetical protein	0	106	19	1	centroid_1471	111.278649	5.14E-26	centroid_1471	1.26E-21
centroid_14714	conserved hypothetical protein	0	106	19	1	centroid_1471	111.278649	5.14E-26	centroid_1471	1.26E-21
centroid_1035	2-keto-3-deoxy-L-rhamnonate aldolase	0	105	19	2	centroid_1035	104.915298	1.27E-24	centroid_1035	1.32E-20
centroid_1036	major Facilitator Superfamily protein	0	105	19	2	centroid_1036	104.915298	1.27E-24	centroid_1036	1.32E-20
centroid_1037	major Facilitator Superfamily protein	0	105	19	2	centroid_1037	104.915298	1.27E-24	centroid_1037	1.32E-20
centroid_1038	L-rhamnonate dehydratase	0	105	19	2	centroid_1038	104.915298	1.27E-24	centroid_1038	1.32E-20
centroid_1039	conserved hypothetical protein	0	105	19	2	centroid_1039	104.915298	1.27E-24	centroid_1039	1.32E-20
centroid_16153	bacterial transcriptional regulator family protein	0	105	19	2	centroid_1615	104.915298	1.27E-24	centroid_1615	1.32E-20
centroid_16154	iclR helix-turn-helix domain protein	0	105	19	2	centroid_1615	104.915298	1.27E-24	centroid_1615	1.32E-20
centroid_4273	inner membrane transport protein RhmT	0	105	19	2	centroid_4273	104.915298	1.27E-24	centroid_4273	1.32E-20
centroid_7695	bacterial transcriptional regulator family protein	0	105	19	2	centroid_7695	104.915298	1.27E-24	centroid_7695	1.32E-20
centroid_7696	iclR helix-turn-helix domain protein	0	105	19	2	centroid_7696	104.915298	1.27E-24	centroid_7696	1.32E-20
centroid_16783	hypothetical protein	0	104	19	3	centroid_1678	99.130452	2.36E-23	centroid_1678	9.69E-20
centroid_9217	conserved hypothetical protein	0	104	19	3	centroid_9217	99.130452	2.36E-23	centroid_9217	9.69E-20
centroid_16928	yiaA/B two helix domain protein	0	103	19	4	centroid_1692	93.8486555	3.41E-22	centroid_1692	5.57E-19
centroid_4665	conserved hypothetical protein	0	102	19	5	centroid_4665	89.0070274	3.93E-21	centroid_4665	2.67E-18
centroid_4710	gntP permease family protein	0	102	19	5	centroid_4710	89.0070274	3.93E-21	centroid_4710	2.67E-18
centroid_4814	conserved hypothetical protein	0	102	19	5	centroid_4814	89.0070274	3.93E-21	centroid_4814	2.67E-18
centroid_17796	outer membrane autotransporter barrel domain protein	0	101	19	6	centroid_1779	84.5527482	3.74E-20	centroid_1779	1.11E-17
centroid_4666	outer membrane autotransporter barrel domain protein	0	101	19	6	centroid_4666	84.5527482	3.74E-20	centroid_4666	1.11E-17
centroid_8894	outer membrane autotransporter barrel domain protein	0	101	19	6	centroid_8894	84.5527482	3.74E-20	centroid_8894	1.11E-17
centroid_13907	conserved hypothetical protein	0	99	19	8	centroid_1390	76.6340831	2.06E-18	centroid_1390	1.40E-16
centroid_1481	putative lipoprotein	0	99	19	8	centroid_1481	76.6340831	2.06E-18	centroid_1481	1.40E-16
centroid_1482	conserved hypothetical protein	0	99	19	8	centroid_1482	76.6340831	2.06E-18	centroid_1482	1.40E-16
centroid_8471	conserved hypothetical protein	0	99	19	8	centroid_8471	76.6340831	2.06E-18	centroid_8471	1.40E-16
centroid_9316	protein PhnP	0	97	19	10	centroid_9316	69.8077176	6.54E-17	centroid_9316	1.26E-15
centroid_4739	WGR domain protein	0	94	19	13	centroid_4739	61.1682266	5.24E-15	centroid_4739	2.18E-14
centroid_526	conserved hypothetical protein	0	94	19	13	centroid_526	61.1682266	5.24E-15	centroid_526	2.18E-14
centroid_15969	CRISPR-associated endonuclease/helicase Cas3	0	92	19	15	centroid_1596	56.2556611	6.36E-14	centroid_1596	1.17E-13
centroid_15998	SPR-associated endonuclease/helicase Cas3 domain pro	0	91	19	16	centroid_1599	54.0099436	1.99E-13	centroid_1599	2.55E-13
centroid_17627	fimbrial subunit ElfA	0	90	19	17	centroid_1762	51.8890064	5.87E-13	centroid_1762	5.41E-13
centroid_9313	CRISPR-associated endonuclease/helicase Cas3	0	90	19	17	centroid_9313	51.8890064	5.87E-13	centroid_9313	5.41E-13
centroid_12408	RISPR-associated protein Cas5/CasD, subtype I-E/ECO	0	89	19	18	centroid_1240	49.8827327	1.63E-12	centroid_1240	1.11E-12
centroid_13690	CT1975-like family protein	0	89	19	18	centroid_1369	49.8827327	1.63E-12	centroid_1369	1.11E-12
centroid_16204	CRISPR type I-E/ECOLI-associated protein CasA/Cse1	0	89	19	18	centroid_1620	49.8827327	1.63E-12	centroid_1620	1.11E-12
centroid_2335	CRISPR type I-E/ECOLI-associated protein CasB/Cse2	0	89	19	18	centroid_2335	49.8827327	1.63E-12	centroid_2335	1.11E-12
centroid_5348	CT1975-like family protein	0	89	19	18	centroid_5348	49.8827327	1.63E-12	centroid_5348	1.11E-12
centroid_5358	SPR-associated protein Cas7/Cse4/CasC, subtype I-E/E(	0	89	19	18	centroid_5358	49.8827327	1.63E-12	centroid_5358	1.11E-12
centroid_17647	CRISPR type I-E/ECOLI-associated protein CasA/Cse1	0	88	19	19	centroid_1764	47.9820707	4.30E-12	centroid_1764	2.22E-12
centroid_4613	CRISPR type I-E/ECOLI-associated protein CasA/Cse1	0	88	19	19	centroid_4613	47.9820707	4.30E-12	centroid_4613	2.22E-12
centroid_6137	CRISPR type I-E/ECOLI-associated protein CasA/Cse1	0	88	19	19	centroid_6137	47.9820707	4.30E-12	centroid_6137	2.22E-12
centroid_4612	RISPR-associated protein Cas5/CasD, subtype I-E/ECO	0	87	19	20	centroid_4612	46.178897	1.08E-11	centroid_4612	4.33E-12
centroid_12681	RISPR-associated protein Cas5/CasD, subtype I-E/ECO	0	85	19	22	centroid_1268	42.8364841	5.95E-11	centroid_1268	1.54E-11
centroid_9314	SPR-associated protein Cas6/Cse3/CasE, subtype I-E/E(	0	85	19	22	centroid_9314	42.8364841	5.95E-11	centroid_9314	1.54E-11
centroid_16849	hypothetical protein	0	83	19	24	centroid_1684	39.8050679	2.81E-10	centroid_1684	5.03E-11
centroid_2333	RISPR-associated protein Cas5/CasD, subtype I-E/ECO	0	83	19	24	centroid_2333	39.8050679	2.81E-10	centroid_2333	5.03E-11
centroid_5188	transposase DDE domain protein	0	80	19	27	centroid_5188	35.7523383	2.24E-09	centroid_5188	2.61E-10
centroid_580	biquinone/plastoquinone (complex I), various chains fami	0	78	19	29	centroid_580	33.3320571	7.77E-09	centroid_580	7.26E-10
centroid_2334	SPR-associated protein Cas7/Cse4/CasC, subtype I-E/E(	0	76	19	31	centroid_2334	31.1054814	2.44E-08	centroid_2334	1.91E-09
centroid_270	conserved hypothetical protein	0	76	19	31	centroid_270	31.1054814	2.44E-08	centroid_270	1.91E-09
centroid_4397	ftsK/SpoIIIE family protein	0	76	19	31	centroid_4397	31.1054814	2.44E-08	centroid_4397	1.91E-09
centroid_11294	porin, autotransporter (AT) family	0	75	19	32	centroid_1129	30.0577136	4.19E-08	centroid_1129	3.05E-09
centroid_13494	e ATP-binding component of a transport system domain p	0	75	19	32	centroid_1349	30.0577136	4.19E-08	centroid_1349	3.05E-09
centroid_14668	hypothetical protein	0	75	19	32	centroid_1466	30.0577136	4.19E-08	centroid_1466	3.05E-09
centroid_15981	H+ symporter family protein	0	75	19	32	centroid_1598	30.0577136	4.19E-08	centroid_1598	3.05E-09
centroid_16661	toxin YafO, type II toxin-antitoxin system family protein	0	75	19	32	centroid_1666	30.0577136	4.19E-08	centroid_1666	3.05E-09
centroid_17797	conserved hypothetical protein	0	75	19	32	centroid_1779	30.0577136	4.19E-08	centroid_1779	3.05E-09
centroid_1911	outer membrane autotransporter barrel domain protein	0	75	19	32	centroid_1911	30.0577136	4.19E-08	centroid_1911	3.05E-09
centroid_2822	antitoxin YafN	0	75	19	32	centroid_2822	30.0577136	4.19E-08	centroid_2822	3.05E-09
centroid_11286	D-serine deaminase transcriptional activator	0	74	19	33	centroid_1128	29.050267	7.05E-08	centroid_1128	4.80E-09
centroid_1251	dsdX permease	0	74	19	33	centroid_1251	29.050267	7.05E-08	centroid_1251	4.80E-09
centroid_2823	mRNA interferase YafO	0	74	19	33	centroid_2823	29.050267	7.05E-08	centroid_2823	4.80E-09
centroid_10353	orn/Lys/Arg decarboxylase, major domain protein	0	73	19	34	centroid_1035	28.0808602	1.16E-07	centroid_1035	7.49E-09
centroid_12500	ornithine decarboxylase, inducible domain protein	0	73	19	34	centroid_1250	28.0808602	1.16E-07	centroid_1250	7.49E-09
centroid_1484	conserved hypothetical protein	0	73	19	34	centroid_1484	28.0808602	1.16E-07	centroid_1484	7.49E-09
centroid_16491	orn/Lys/Arg decarboxylase, N-terminal domain protein	0	73	19	34	centroid_1649	28.0808602	1.16E-07	centroid_1649	7.49E-09
centroid_16443	D-serine deaminase transcriptional activator	0	72	19	35	centroid_1644	27.1473807	1.89E-07	centroid_1644	1.16E-08
centroid_2671	phosphodiesterase / nucleotide pyrophosphatase family p	0	72	19	35	centroid_2671	27.1473807	1.89E-07	centroid_2671	1.16E-08
centroid_1250	D-serine deaminase transcriptional activator	0	71	19	36	centroid_1250	26.2478699	3.00E-07	centroid_1250	1.76E-08
centroid_10799	ı-negative pili assembly chaperone, C-terminal domain pr	0	70	19	37	centroid_1079	25.3805091	4.71E-07	centroid_1079	2.67E-08
centroid_16913	putative membrane protein	0	70	19	37	centroid_1691	25.3805091	4.71E-07	centroid_1691	2.67E-08
centroid_1239	ı-negative pili assembly chaperone, N-terminal domain pr	0	69	19	38	centroid_1239	24.5436071	7.26E-07	centroid_1239	4.01E-08
centroid_16914	ı-negative pili assembly chaperone, N-terminal domain pr	0	69	19	38	centroid_1691	24.5436071	7.26E-07	centroid_1691	4.01E-08
centroid_4811	putative 4'-phosphopantetheinyl transferase EntD	0	69	19	38	centroid_4811	24.5436071	7.26E-07	centroid_4811	4.01E-08
centroid_1175	von Willebrand factor family protein	0	65	19	42	centroid_1175	21.4706503	3.59E-06	centroid_1175	1.87E-07
centroid_6930	conserved hypothetical protein	0	65	19	42	centroid_6930	21.4706503	3.59E-06	centroid_6930	1.87E-07
centroid_10249	conserved hypothetical protein	0	64	19	43	centroid_1024	20.764435	5.19E-06	centroid_1024	2.69E-07
centroid_1179	UBA/TS-N domain protein	0	64	19	43	centroid_1179	20.764435	5.19E-06	centroid_1179	2.69E-07
centroid_1180	conserved hypothetical protein	0	64	19	43	centroid_1180	20.764435	5.19E-06	centroid_1180	2.69E-07
centroid_1181	tetratricopeptide repeat family protein	0	64	19	43	centroid_1181	20.764435	5.19E-06	centroid_1181	2.69E-07
centroid_17103	CRISPR type I-E/ECOLI-associated protein CasA/Cse1	0	64	19	43	centroid_1710	20.764435	5.19E-06	centroid_1710	2.69E-07
centroid_17771	hypothetical protein	0	64	19	43	centroid_1777	20.764435	5.19E-06	centroid_1777	2.69E-07
centroid_2946	ı-negative pili assembly chaperone, C-terminal domain pr	0	64	19	43	centroid_2946	20.764435	5.19E-06	centroid_2946	2.69E-07
centroid_1235	conserved hypothetical protein	0	63	19	44	centroid_1235	20.080669	7.42E-06	centroid_1235	7.71E-07
centroid_1236	fimbrial family protein	0	63	19	44	centroid_1236	20.080669	7.42E-06	centroid_1236	7.71E-07
centroid_1237	fimbrial family protein	0	63	19	44	centroid_1237	20.080669	7.42E-06	centroid_1237	7.71E-07

centroid_1238	fimbrial family protein	0	63	19	44	centroid_1238	20.080669	7.42E-06	centroid_1238	7.71E-07
centroid_13865	fimbrial family protein	0	63	19	44	centroid_1386	20.080669	7.42E-06	centroid_1386	7.71E-07
centroid_14725	o-4-hydroxy-6-hydroxymethyldihydropteridine diphospho	0	61	19	46	centroid_1472	18.7763462	1.47E-05	centroid_1472	9.62E-07
centroid_16583	CRISPR type I-E/ECOLI-associated protein CasA/Cse1	0	61	19	46	centroid_1658	18.7763462	1.47E-05	centroid_1658	9.62E-07
centroid_17155	helix-turn-helix domain protein	0	61	19	46	centroid_1715	18.7763462	1.47E-05	centroid_1715	9.62E-07
centroid_1178	conserved hypothetical protein	0	60	19	47	centroid_1178	18.1538769	2.04E-05	centroid_1178	1.22E-06
centroid_2336	CRISPR type I-E/ECOLI-associated protein CasA/Cse1	0	60	19	47	centroid_2336	18.1538769	2.04E-05	centroid_2336	1.22E-06
centroid_3323	integrase core domain protein	0	60	19	47	centroid_3323	18.1538769	2.04E-05	centroid_3323	1.22E-06
centroid_4332	calcineurin-like phosphoesterase family protein	0	60	19	47	centroid_4332	18.1538769	2.04E-05	centroid_4332	1.22E-06
centroid_6506	hypothetical protein	0	60	19	47	centroid_6506	18.1538769	2.04E-05	centroid_6506	1.22E-06
centroid_7671	conserved hypothetical protein	0	60	19	47	centroid_7671	18.1538769	2.04E-05	centroid_7671	1.22E-06
centroid_4860	CFA/III pilin	0	59	19	48	centroid_4860	17.5500229	2.80E-05	centroid_4860	1.61E-06
centroid_864	putative membrane protein	0	59	19	48	centroid_864	17.5500229	2.80E-05	centroid_864	1.61E-06
centroid_10746	putative ybl54	0	58	19	49	centroid_1074	16.9639645	3.81E-05	centroid_1074	4.09E-06
centroid_15898	putative predicted protein	0	58	19	49	centroid_1589	16.9639645	3.81E-05	centroid_1589	4.09E-06
centroid_17655	conserved hypothetical protein	0	58	19	49	centroid_1765	16.9639645	3.81E-05	centroid_1765	4.09E-06
centroid_8703	putative ybl54	0	58	19	49	centroid_8703	16.9639645	3.81E-05	centroid_8703	4.09E-06
centroid_13614	putative membrane protein	0	57	19	50	centroid_1361	16.3949299	5.14E-05	centroid_1361	4.30E-06
centroid_16888	conserved hypothetical protein	0	57	19	50	centroid_1688	16.3949299	5.14E-05	centroid_1688	4.30E-06
centroid_7913	hypothetical protein	0	57	19	50	centroid_7913	16.3949299	5.14E-05	centroid_7913	4.30E-06
centroid_2647	sulfite exporter TauE/SafE family protein	0	55	19	52	centroid_2647	15.3050624	9.15E-05	centroid_2647	6.11E-06
centroid_2779	conserved hypothetical protein	0	55	19	52	centroid_2779	15.3050624	9.15E-05	centroid_2779	6.11E-06

Table S5. Distribution by Phylogroup E

Gene_ID	Annotation	Phylogroup E_present	Other_present	Phylogroup E_Absent	Other_Absent	Gene_ID	chisq-stats	pvalues	Gene_ID	pvalues
centroid_14354	conserved hypothetical protein	5	0	0	120	centroid_1435	100.31467	1.30E-23	centroid_1435	4.26E-09
centroid_14355	leucine rich repeat family protein	5	0	0	120	centroid_1435	100.31467	1.30E-23	centroid_1435	4.26E-09
centroid_17760	type III restriction enzyme, res subunit	5	0	0	120	centroid_1776	100.31467	1.30E-23	centroid_1776	4.26E-09
centroid_17761	helicase conserved C-terminal domain protein	5	0	0	120	centroid_1776	100.31467	1.30E-23	centroid_1776	4.26E-09
centroid_17762	TP-dependent DNA helicase, RecQ family domain protei	5	0	0	120	centroid_1776	100.31467	1.30E-23	centroid_1776	4.26E-09
centroid_17795	leucine rich repeat family protein	5	0	0	120	centroid_1779	100.31467	1.30E-23	centroid_1779	4.26E-09
centroid_17835	putative z1097 gene product	5	0	0	120	centroid_1783	100.31467	1.30E-23	centroid_1783	4.26E-09
centroid_8767	conserved hypothetical protein	5	0	0	120	centroid_8767	100.31467	1.30E-23	centroid_8767	4.26E-09
centroid_8785	conserved hypothetical protein	5	0	0	120	centroid_8785	100.31467	1.30E-23	centroid_8785	4.26E-09
centroid_8801	hypothetical protein	5	0	0	120	centroid_8801	100.31467	1.30E-23	centroid_8801	4.26E-09
centroid_8802	conserved hypothetical protein	5	0	0	120	centroid_8802	100.31467	1.30E-23	centroid_8802	4.26E-09
centroid_8834	ATP-dependent DNA helicase, RecQ family protein	5	0	0	120	centroid_8834	100.31467	1.30E-23	centroid_8834	4.26E-09
centroid_8835	DNA recombination-mediator A family protein	5	0	0	120	centroid_8835	100.31467	1.30E-23	centroid_8835	4.26E-09
centroid_8895	conserved hypothetical protein	5	0	0	120	centroid_8895	100.31467	1.30E-23	centroid_8895	4.26E-09
centroid_8896	tonB-dependent Receptor Plug domain protein	5	0	0	120	centroid_8896	100.31467	1.30E-23	centroid_8896	4.26E-09
centroid_8897	olinate phosphoribosyl transferase, C-terminal domain pr	5	0	0	120	centroid_8897	100.31467	1.30E-23	centroid_8897	4.26E-09
centroid_8898	methyltransferase domain protein	5	0	0	120	centroid_8898	100.31467	1.30E-23	centroid_8898	4.26E-09
centroid_8899	ABC transporter family protein	5	0	0	120	centroid_8899	100.31467	1.30E-23	centroid_8899	4.26E-09
centroid_8900	fecCD transport family protein	5	0	0	120	centroid_8900	100.31467	1.30E-23	centroid_8900	4.26E-09
centroid_8901	periplasmic binding family protein	5	0	0	120	centroid_8901	100.31467	1.30E-23	centroid_8901	4.26E-09
centroid_8958	type VII secretion system (T7SS), usher family protein	5	0	0	120	centroid_8958	100.31467	1.30E-23	centroid_8958	4.26E-09
centroid_8959	fimbrial family protein	5	0	0	120	centroid_8959	100.31467	1.30E-23	centroid_8959	4.26E-09
centroid_9082	fimbrial family protein	5	0	0	120	centroid_9082	100.31467	1.30E-23	centroid_9082	4.26E-09
centroid_9084	type VII secretion system (T7SS), usher family protein	5	0	0	120	centroid_9084	100.31467	1.30E-23	centroid_9084	4.26E-09
centroid_9085	fimbrial family protein	5	0	0	120	centroid_9085	100.31467	1.30E-23	centroid_9085	4.26E-09
centroid_9086	fimbrial family protein	5	0	0	120	centroid_9086	100.31467	1.30E-23	centroid_9086	4.26E-09
centroid_9252	major Facilitator Superfamily protein	5	0	0	120	centroid_9252	100.31467	1.30E-23	centroid_9252	4.26E-09
centroid_9262	type VII secretion system (T7SS), usher family protein	5	0	0	120	centroid_9262	100.31467	1.30E-23	centroid_9262	4.26E-09
centroid_9263	type VII secretion system (T7SS), usher family protein	5	0	0	120	centroid_9263	100.31467	1.30E-23	centroid_9263	4.26E-09
centroid_9457	-negative pili assembly chaperone, N-terminal domain pr	5	0	0	120	centroid_9457	100.31467	1.30E-23	centroid_9457	4.26E-09
centroid_12420	conserved hypothetical protein	5	2	0	118	centroid_1242	70.1816611	5.41E-17	centroid_1242	8.95E-08
centroid_8836	PBP superfamily domain protein	5	2	0	118	centroid_8836	70.1816611	5.41E-17	centroid_8836	8.95E-08
centroid_8961	conserved hypothetical protein	5	2	0	118	centroid_8961	70.1816611	5.41E-17	centroid_8961	8.95E-08
centroid_11114	vitamin B12 transporter BtuB	5	7	0	113	centroid_1111	38.7945935	4.71E-10	centroid_1111	3.38E-06
centroid_6297	maltoporin periplasmic N-terminal extension family protein	5	7	0	113	centroid_6297	38.7945935	4.71E-10	centroid_6297	3.38E-06
centroid_6298	tonB-dependent vitamin B12 receptor	5	7	0	113	centroid_6298	38.7945935	4.71E-10	centroid_6298	3.38E-06
centroid_7241	alpha/beta hydrolase family protein	5	7	0	113	centroid_7241	38.7945935	4.71E-10	centroid_7241	3.38E-06
centroid_7242	NAD dependent epimerase/dehydratase family protein	5	7	0	113	centroid_7242	38.7945935	4.71E-10	centroid_7242	3.38E-06
centroid_9337	putative carboxymethylenebutenolidase	5	7	0	113	centroid_9337	38.7945935	4.71E-10	centroid_9337	3.38E-06
centroid_9338	X-Pro dipeptidyl-peptidase family protein	5	7	0	113	centroid_9338	38.7945935	4.71E-10	centroid_9338	3.38E-06
centroid_9079	(Glycoside-Pentoside-Hexuronide) transporter domain p	5	8	0	112	centroid_9079	35.4146992	2.66E-09	centroid_9079	5.49E-06
centroid_9080	helix-turn-helix domain protein	5	8	0	112	centroid_9080	35.4146992	2.66E-09	centroid_9080	5.49E-06
centroid_9576	conserved hypothetical protein	5	8	0	112	centroid_9576	35.4146992	2.66E-09	centroid_9576	5.49E-06
centroid_10820	zinc-binding dehydrogenase family protein	5	10	0	110	centroid_1082	30.0071023	4.30E-08	centroid_1082	1.28E-05
centroid_5659	conserved hypothetical protein	5	10	0	110	centroid_5659	30.0071023	4.30E-08	centroid_5659	1.28E-05
centroid_8474	putative sor-operon regulator	5	10	0	110	centroid_8474	30.0071023	4.30E-08	centroid_8474	1.28E-05
centroid_8475	short chain dehydrogenase family protein	5	10	0	110	centroid_8475	30.0071023	4.30E-08	centroid_8475	1.28E-05
centroid_8476	m, mannose/fructose/sorbose family, IIA component dom	5	10	0	110	centroid_8476	30.0071023	4.30E-08	centroid_8476	1.28E-05
centroid_8477	orbose-specific phosphotransferase enzyme IIB compone	5	10	0	110	centroid_8477	30.0071023	4.30E-08	centroid_8477	1.28E-05
centroid_8478	stem, mannose/fructose/sorbose , IIC component family	5	10	0	110	centroid_8478	30.0071023	4.30E-08	centroid_8478	1.28E-05
centroid_8479	stem, mannose/fructose/sorbose , IID component family	5	10	0	110	centroid_8479	30.0071023	4.30E-08	centroid_8479	1.28E-05
centroid_8480	zinc-binding dehydrogenase family protein	5	10	0	110	centroid_8480	30.0071023	4.30E-08	centroid_8480	1.28E-05
centroid_8976	conserved hypothetical protein	5	10	0	110	centroid_8976	30.0071023	4.30E-08	centroid_8976	1.28E-05
centroid_9043	putative sugar-binding domain protein	5	10	0	110	centroid_9043	30.0071023	4.30E-08	centroid_9043	1.28E-05
centroid_9044	putative sugar-binding domain protein	5	10	0	110	centroid_9044	30.0071023	4.30E-08	centroid_9044	1.28E-05
centroid_16938	bacterial Ig-like domain family protein	5	12	0	108	centroid_1693	25.8721689	3.65E-07	centroid_1693	2.64E-05
centroid_17334	bacterial Ig-like domain family protein	5	13	0	107	centroid_1733	24.1493867	8.91E-07	centroid_1733	3.65E-05
centroid_6607	bacterial Ig-like domain family protein	5	13	0	107	centroid_6607	24.1493867	8.91E-07	centroid_6607	3.65E-05
centroid_8902	conserved hypothetical protein	5	15	0	105	centroid_8902	21.2208581	4.09E-06	centroid_8902	6.61E-05
centroid_13157	bacterial Ig-like domain family protein	5	16	0	104	centroid_1315	19.965874	7.88E-06	centroid_1315	8.68E-05
centroid_8095	shET2 enterotoxin, N-terminal region family protein	5	18	0	102	centroid_8095	17.7834834	2.48E-05	centroid_8095	0.00014347
centroid_9045	shET2 enterotoxin, N-terminal region family protein	5	18	0	102	centroid_9045	17.7834834	2.48E-05	centroid_9045	0.00014347
centroid_13367	type IV leader peptidase family protein	5	19	0	101	centroid_1336	16.8287825	4.09E-05	centroid_1336	0.00018123
centroid_13677	shET2 enterotoxin, N-terminal region family protein	5	19	0	101	centroid_1367	16.8287825	4.09E-05	centroid_1367	0.00018123
centroid_5812	conserved hypothetical protein	5	19	0	101	centroid_5812	16.8287825	4.09E-05	centroid_5812	0.00018123
centroid_5955	BFD-like [2Fe-2S] binding domain protein	5	19	0	101	centroid_5955	16.8287825	4.09E-05	centroid_5955	0.00018123
centroid_5956	bacterioferritin	5	19	0	101	centroid_5956	16.8287825	4.09E-05	centroid_5956	0.00018123
centroid_8094	ankyrin repeat A domain protein	5	19	0	101	centroid_8094	16.8287825	4.09E-05	centroid_8094	0.00018123
centroid_9373	putative ankyrin repeat A domain protein	5	19	0	101	centroid_9373	16.8287825	4.09E-05	centroid_9373	0.00018123
centroid_9374	putative type III effector domain protein	5	19	0	101	centroid_9374	16.8287825	4.09E-05	centroid_9374	0.00018123
centroid_11151	conserved hypothetical protein	5	21	0	99	centroid_1115	15.1398804	9.98E-05	centroid_1115	0.00028047
centroid_355	fimbrial family protein	5	21	0	99	centroid_355	15.1398804	9.98E-05	centroid_355	0.00028047
centroid_9480	conserved hypothetical protein	5	21	0	99	centroid_9480	15.1398804	9.98E-05	centroid_9480	0.00028047
centroid_13491	haemagglutinin family protein	4	0	1	120	centroid_1349	75.0285167	4.64E-18	centroid_1349	5.16E-07
centroid_14343	conserved hypothetical protein	4	0	1	120	centroid_1434	75.0285167	4.64E-18	centroid_1434	5.16E-07
centroid_17758	conserved hypothetical protein	4	0	1	120	centroid_1775	75.0285167	4.64E-18	centroid_1775	5.16E-07
centroid_17794	invasion plasmid antigen domain protein	4	0	1	120	centroid_1779	75.0285167	4.64E-18	centroid_1779	5.16E-07
centroid_17812	repair family protein	4	0	1	120	centroid_1781	75.0285167	4.64E-18	centroid_1781	5.16E-07
centroid_17813	conserved hypothetical protein	4	0	1	120	centroid_1781	75.0285167	4.64E-18	centroid_1781	5.16E-07
centroid_17969	marB family protein	4	0	1	120	centroid_1796	75.0285167	4.64E-18	centroid_1796	5.16E-07
centroid_8790	fimbrial family protein	4	0	1	120	centroid_8790	75.0285167	4.64E-18	centroid_8790	5.16E-07
centroid_8791	-negative pili assembly chaperone, N-terminal domain pr	4	0	1	120	centroid_8791	75.0285167	4.64E-18	centroid_8791	5.16E-07

centroid_8792	outer membrane usher protein HtrE	4	0	1	120	centroid_8792	75.0285167	4.64E-18	centroid_8792	5.16E-07
centroid_8793	fimbrial family protein	4	0	1	120	centroid_8793	75.0285167	4.64E-18	centroid_8793	5.16E-07
centroid_8795	conserved hypothetical protein	4	0	1	120	centroid_8795	75.0285167	4.64E-18	centroid_8795	5.16E-07
centroid_8845	repair family protein	4	0	1	120	centroid_8845	75.0285167	4.64E-18	centroid_8845	5.16E-07
centroid_8846	repair family protein	4	0	1	120	centroid_8846	75.0285167	4.64E-18	centroid_8846	5.16E-07
centroid_8847	repair family protein	4	0	1	120	centroid_8847	75.0285167	4.64E-18	centroid_8847	5.16E-07
centroid_8975	conserved hypothetical protein	4	0	1	120	centroid_8975	75.0285167	4.64E-18	centroid_8975	5.16E-07
centroid_8988	conserved hypothetical protein	4	0	1	120	centroid_8988	75.0285167	4.64E-18	centroid_8988	5.16E-07
centroid_8989	conserved hypothetical protein	4	0	1	120	centroid_8989	75.0285167	4.64E-18	centroid_8989	5.16E-07
centroid_9083	-negative pili assembly chaperone, C-terminal domain pr	4	0	1	120	centroid_9083	75.0285167	4.64E-18	centroid_9083	5.16E-07
centroid_9243	pentapeptide repeats family protein	4	0	1	120	centroid_9243	75.0285167	4.64E-18	centroid_9243	5.16E-07
centroid_9253	bacterial regulatory helix-turn-helix , lysR family protein	4	0	1	120	centroid_9253	75.0285167	4.64E-18	centroid_9253	5.16E-07
centroid_9450	conserved hypothetical protein	4	0	1	120	centroid_9450	75.0285167	4.64E-18	centroid_9450	5.16E-07
centroid_9451	conserved hypothetical protein	4	0	1	120	centroid_9451	75.0285167	4.64E-18	centroid_9451	5.16E-07
centroid_9458	-negative pili assembly chaperone, C-terminal domain pr	4	0	1	120	centroid_9458	75.0285167	4.64E-18	centroid_9458	5.16E-07
centroid_9467	haemagglutinin family protein	4	0	1	120	centroid_9467	75.0285167	4.64E-18	centroid_9467	5.16E-07
centroid_9556	tonB-dependent siderophore receptor family protein	4	0	1	120	centroid_9556	75.0285167	4.64E-18	centroid_9556	5.16E-07
centroid_9557	ABC transporter family protein	4	0	1	120	centroid_9557	75.0285167	4.64E-18	centroid_9557	5.16E-07
centroid_9558	fecCD transport family protein	4	0	1	120	centroid_9558	75.0285167	4.64E-18	centroid_9558	5.16E-07
centroid_9559	fecCD transport family protein	4	0	1	120	centroid_9559	75.0285167	4.64E-18	centroid_9559	5.16E-07
centroid_9560	periplasmic binding family protein	4	0	1	120	centroid_9560	75.0285167	4.64E-18	centroid_9560	5.16E-07
centroid_9561	SIS domain protein	4	0	1	120	centroid_9561	75.0285167	4.64E-18	centroid_9561	5.16E-07
centroid_8947	outer membrane protein C	4	1	1	119	centroid_8947	59.0820313	1.51E-14	centroid_8947	2.56E-06
centroid_8833	tetratricopeptide repeat family protein	4	2	1	118	centroid_8833	48.4524539	3.38E-12	centroid_8833	7.64E-06
centroid_9447	conserved hypothetical protein	4	2	1	118	centroid_9447	48.4524539	3.38E-12	centroid_9447	7.64E-06
centroid_14726	conserved hypothetical protein	4	6	1	114	centroid_1472	27.2022192	1.83E-07	centroid_1472	0.00010405
centroid_9033	conserved hypothetical protein	4	6	1	114	centroid_9033	27.2022192	1.83E-07	centroid_9033	0.00010405
centroid_9711	conserved hypothetical protein	4	6	1	114	centroid_9711	27.2022192	1.83E-07	centroid_9711	0.00010405
centroid_9739	transposase family protein	4	6	1	114	centroid_9739	27.2022192	1.83E-07	centroid_9739	0.00010405
centroid_18640	transposase family protein	4	7	1	113	centroid_1864	24.3065939	8.22E-07	centroid_1864	0.00016237
centroid_8361	putative transposase	4	7	1	113	centroid_8361	24.3065939	8.22E-07	centroid_8361	0.00016237
centroid_9246	conserved hypothetical protein	4	8	1	112	centroid_9246	21.8943968	2.88E-06	centroid_9246	0.00024187
centroid_18149	acetyltransferase domain protein	4	9	1	111	centroid_1814	19.854088	8.36E-06	centroid_1814	0.00034693
centroid_18234	lemA family protein	4	10	1	110	centroid_1823	18.1059966	2.09E-05	centroid_1823	0.00048229
centroid_7244	acyl transferase domain protein	4	10	1	110	centroid_7244	18.1059966	2.09E-05	centroid_7244	0.00048229
centroid_14456	conserved hypothetical protein	4	11	1	109	centroid_1445	16.5916982	4.64E-05	centroid_1445	0.00065302
centroid_15266	conserved hypothetical protein	4	11	1	109	centroid_1526	16.5916982	4.64E-05	centroid_1526	0.00065302
centroid_6478	nucleotide sugar dehydrogenase family protein	4	11	1	109	centroid_6478	16.5916982	4.64E-05	centroid_6478	0.00065302
centroid_6768	enterobacterial Ail/Lom family protein	4	11	1	109	centroid_6768	16.5916982	4.64E-05	centroid_6768	0.00065302
centroid_14323	caudovirales tail fibre assembly family protein	4	12	1	108	centroid_1432	15.267375	9.33E-05	centroid_1432	0.00086448
centroid_9413	bacterial Ig-like domain family protein	4	12	1	108	centroid_9413	15.267375	9.33E-05	centroid_9413	0.00086448
centroid_14327	fimbrial family protein	3	0	2	120	centroid_1432	50.3792407	1.27E-12	centroid_1432	3.15E-05
centroid_14367	conserved hypothetical protein	3	0	2	120	centroid_1436	50.3792407	1.27E-12	centroid_1436	3.15E-05
centroid_14415	conserved hypothetical protein	3	0	2	120	centroid_1441	50.3792407	1.27E-12	centroid_1441	3.15E-05
centroid_14416	hypothetical protein	3	0	2	120	centroid_1441	50.3792407	1.27E-12	centroid_1441	3.15E-05
centroid_14432	ype III secretion outer membrane pore, YscC/HrcC family	3	0	2	120	centroid_1443	50.3792407	1.27E-12	centroid_1443	3.15E-05
centroid_17747	HAMP domain protein	3	0	2	120	centroid_1774	50.3792407	1.27E-12	centroid_1774	3.15E-05
centroid_17757	conserved hypothetical protein	3	0	2	120	centroid_1775	50.3792407	1.27E-12	centroid_1775	3.15E-05
centroid_18012	ive non-LEE-encoded type III secreted effector domain pr	3	0	2	120	centroid_1801	50.3792407	1.27E-12	centroid_1801	3.15E-05
centroid_18123	conserved hypothetical protein	3	0	2	120	centroid_1812	50.3792407	1.27E-12	centroid_1812	3.15E-05
centroid_18144	sopA-like catalytic domain protein	3	0	2	120	centroid_1814	50.3792407	1.27E-12	centroid_1814	3.15E-05
centroid_18145	sopA-like central domain protein	3	0	2	120	centroid_1814	50.3792407	1.27E-12	centroid_1814	3.15E-05
centroid_18251	putative ipaH-like protein	3	0	2	120	centroid_1825	50.3792407	1.27E-12	centroid_1825	3.15E-05
centroid_18252	leucine Rich Repeat family protein	3	0	2	120	centroid_1825	50.3792407	1.27E-12	centroid_1825	3.15E-05
centroid_18253	on-LEE-encoded type III secreted effector domain protei	3	0	2	120	centroid_1825	50.3792407	1.27E-12	centroid_1825	3.15E-05
centroid_8797	(Glycoside-Pentoside-Hexuronide) transporter domain p	3	0	2	120	centroid_8797	50.3792407	1.27E-12	centroid_8797	3.15E-05
centroid_8798	glycosyl hydrolases 43 family protein	3	0	2	120	centroid_8798	50.3792407	1.27E-12	centroid_8798	3.15E-05
centroid_8807	hemin import ATP-binding protein HmuV	3	0	2	120	centroid_8807	50.3792407	1.27E-12	centroid_8807	3.15E-05
centroid_8808	fecCD transport family protein	3	0	2	120	centroid_8808	50.3792407	1.27E-12	centroid_8808	3.15E-05
centroid_8809	NADH(P)-binding family protein	3	0	2	120	centroid_8809	50.3792407	1.27E-12	centroid_8809	3.15E-05
centroid_8810	conserved hypothetical protein	3	0	2	120	centroid_8810	50.3792407	1.27E-12	centroid_8810	3.15E-05
centroid_8811	putative heme utilization radical SAM enzyme HutW	3	0	2	120	centroid_8811	50.3792407	1.27E-12	centroid_8811	3.15E-05
centroid_8812	periplasmic binding family protein	3	0	2	120	centroid_8812	50.3792407	1.27E-12	centroid_8812	3.15E-05
centroid_8813	tonB-dependent heme/hemoglobin receptor family protein	3	0	2	120	centroid_8813	50.3792407	1.27E-12	centroid_8813	3.15E-05
centroid_8814	hemin transport protein HemS	3	0	2	120	centroid_8814	50.3792407	1.27E-12	centroid_8814	3.15E-05
centroid_8857	ype III secretion outer membrane pore, YscC/HrcC family	3	0	2	120	centroid_8857	50.3792407	1.27E-12	centroid_8857	3.15E-05
centroid_8858	invasion protein InvF	3	0	2	120	centroid_8858	50.3792407	1.27E-12	centroid_8858	3.15E-05
centroid_8931	conserved hypothetical protein	3	0	2	120	centroid_8931	50.3792407	1.27E-12	centroid_8931	3.15E-05
centroid_8932	conserved hypothetical protein	3	0	2	120	centroid_8932	50.3792407	1.27E-12	centroid_8932	3.15E-05
centroid_8933	methylaspartate mutase, S subunit	3	0	2	120	centroid_8933	50.3792407	1.27E-12	centroid_8933	3.15E-05
centroid_8934	conserved hypothetical family protein	3	0	2	120	centroid_8934	50.3792407	1.27E-12	centroid_8934	3.15E-05
centroid_8935	methylaspartate mutase, E subunit	3	0	2	120	centroid_8935	50.3792407	1.27E-12	centroid_8935	3.15E-05
centroid_8936	methylaspartate ammonia-lyase	3	0	2	120	centroid_8936	50.3792407	1.27E-12	centroid_8936	3.15E-05
centroid_8937	conserved hypothetical protein	3	0	2	120	centroid_8937	50.3792407	1.27E-12	centroid_8937	3.15E-05
centroid_8957	-negative pili assembly chaperone, N-terminal domain pr	3	0	2	120	centroid_8957	50.3792407	1.27E-12	centroid_8957	3.15E-05
centroid_8967	ABC transporter family protein	3	0	2	120	centroid_8967	50.3792407	1.27E-12	centroid_8967	3.15E-05
centroid_8968	-dependent transport system inner membrane componen	3	0	2	120	centroid_8968	50.3792407	1.27E-12	centroid_8968	3.15E-05
centroid_8969	conserved hypothetical protein	3	0	2	120	centroid_8969	50.3792407	1.27E-12	centroid_8969	3.15E-05
centroid_8970	major Facilitator Superfamily protein	3	0	2	120	centroid_8970	50.3792407	1.27E-12	centroid_8970	3.15E-05
centroid_8971	MASE1 family protein	3	0	2	120	centroid_8971	50.3792407	1.27E-12	centroid_8971	3.15E-05
centroid_8972	response regulator	3	0	2	120	centroid_8972	50.3792407	1.27E-12	centroid_8972	3.15E-05
centroid_8974	conserved hypothetical protein	3	0	2	120	centroid_8974	50.3792407	1.27E-12	centroid_8974	3.15E-05
centroid_8977	conserved hypothetical protein	3	0	2	120	centroid_8977	50.3792407	1.27E-12	centroid_8977	3.15E-05
centroid_8978	conserved hypothetical protein	3	0	2	120	centroid_8978	50.3792407	1.27E-12	centroid_8978	3.15E-05
centroid_8980	pentapeptide repeats family protein	3	0	2	120	centroid_8980	50.3792407	1.27E-12	centroid_8980	3.15E-05
centroid_8984	fimbrial family protein	3	0	2	120	centroid_8984	50.3792407	1.27E-12	centroid_8984	3.15E-05
centroid_8987	amidohydrolase family protein	3	0	2	120	centroid_8987	50.3792407	1.27E-12	centroid_8987	3.15E-05
centroid_8990	conserved hypothetical protein	3	0	2	120	centroid_8990	50.3792407	1.27E-12	centroid_8990	3.15E-05
centroid_9008	ADH:flavin oxidoreductase / NADH oxidase family protei	3	0	2	120	centroid_9008	50.3792407	1.27E-12	centroid_9008	3.15E-05
centroid_9009	alpha/beta hydrolase family protein	3	0	2	120	centroid_9009	50.3792407	1.27E-12	centroid_9009	3.15E-05
centroid_9010	lysR substrate binding domain protein	3	0	2	120	centroid_9010	50.3792407	1.27E-12	centroid_9010	3.15E-05
centroid_9011	lysR substrate binding domain protein	3	0	2	120	centroid_9011	50.3792407	1.27E-12	centroid_9011	3.15E-05
centroid_9012	aldo/keto reductase family protein	3	0	2	120	centroid_9012	50.3792407	1.27E-12	centroid_9012	3.15E-05
centroid_9013	aldo/keto reductase family protein	3	0	2	120	centroid_9013	50.3792407	1.27E-12	centroid_9013	3.15E-05
centroid_9107	type I secretion outer membrane , TolC family protein	3	0	2	120	centroid_9107	50.3792407	1.27E-12	centroid_9107	3.15E-05
centroid_9109	type I secretion system ATPase family protein	3	0	2	120	centroid_9109	50.3792407	1.27E-12	centroid_9109	3.15E-05
centroid_9110	type I secretion membrane fusion , HlyD family protein	3	0	2	120	centroid_9110	50.3792407	1.27E-12	centroid_9110	3.15E-05
centroid_9111	conserved hypothetical protein	3	0	2	120	centroid_9111	50.3792407	1.27E-12	centroid_9111	3.15E-05
centroid_9112	putative lipoprotein	3	0	2	120	centroid_9112	50.3792407	1.27E-12	centroid_9112	3.15E-05
centroid_9113	yadA-like C-terminal region family protein	3	0	2	120	centroid_9113	50.3792407	1.27E-12	centroid_9113	3.15E-05
centroid_9114	conserved hypothetical protein	3	0	2	120	centroid_9114	50.3792407	1.27E-12	centroid_9114	3.15E-05
centroid_9119	outer membrane porin protein OmpD	3	0	2	120	centroid_9119	50.3792407	1.27E-12	centroid_9119	3.15E-05
centroid_9120	leucine Rich Repeat family protein	3	0	2	120	centroid_9120	50.3792407	1.27E-12	centroid_9120	3.15E-05
centroid_9134	transcriptional regulatory , C terminal family protein	3	0	2	120	centroid_9134	50.3792407	1.27E-12	centroid_9134	3.15E-05
centroid_9135	pfkB carbohydrate kinase family protein	3	0	2	120	centroid_9135	50.3792407	1.27E-12	centroid_9135	3.15E-05
centroid_9136	ketose-bisphosphate aldolase family protein	3	0	2	120	centroid_9136	50.3792407	1.27E-12	centroid_9136	3.15E-05
centroid_9137	conserved hypothetical protein	3	0	2	120	centroid_9137	50.3792407	1.27E-12	centroid_9137	3.15E-05

centroid_9138	lasmic binding and sugar binding domain of LacI family pr	3	0	2	120	centroid_9138	50.3792407	1.27E-12	centroid_9138	3.15E-05
centroid_9139	ain amino acid transport system / permease component f	3	0	2	120	centroid_9139	50.3792407	1.27E-12	centroid_9139	3.15E-05
centroid_9140	ABC transporter family protein	3	0	2	120	centroid_9140	50.3792407	1.27E-12	centroid_9140	3.15E-05
centroid_9206	conserved hypothetical protein	3	0	2	120	centroid_9206	50.3792407	1.27E-12	centroid_9206	3.15E-05
centroid_9215	conserved hypothetical protein	3	0	2	120	centroid_9215	50.3792407	1.27E-12	centroid_9215	3.15E-05
centroid_9216	conserved hypothetical protein	3	0	2	120	centroid_9216	50.3792407	1.27E-12	centroid_9216	3.15E-05
centroid_9258	conserved hypothetical protein	3	0	2	120	centroid_9258	50.3792407	1.27E-12	centroid_9258	3.15E-05
centroid_9259	fimbrial family protein	3	0	2	120	centroid_9259	50.3792407	1.27E-12	centroid_9259	3.15E-05
centroid_9260	fimbrial family protein	3	0	2	120	centroid_9260	50.3792407	1.27E-12	centroid_9260	3.15E-05
centroid_9261	ı-negative pili assembly chaperone, N-terminal domain pr	3	0	2	120	centroid_9261	50.3792407	1.27E-12	centroid_9261	3.15E-05
centroid_9274	fimbrial family protein	3	0	2	120	centroid_9274	50.3792407	1.27E-12	centroid_9274	3.15E-05
centroid_9341	conserved hypothetical protein	3	0	2	120	centroid_9341	50.3792407	1.27E-12	centroid_9341	3.15E-05
centroid_9342	conserved hypothetical protein	3	0	2	120	centroid_9342	50.3792407	1.27E-12	centroid_9342	3.15E-05
centroid_9343	helix-turn-helix family protein	3	0	2	120	centroid_9343	50.3792407	1.27E-12	centroid_9343	3.15E-05
centroid_9416	glycosyl transferases group 1 family protein	3	0	2	120	centroid_9416	50.3792407	1.27E-12	centroid_9416	3.15E-05
centroid_9417	polysaccharide biosynthesis family protein	3	0	2	120	centroid_9417	50.3792407	1.27E-12	centroid_9417	3.15E-05
centroid_9419	transposase DDE domain protein	3	0	2	120	centroid_9419	50.3792407	1.27E-12	centroid_9419	3.15E-05
centroid_9420	putative h repeat-associated protein YhhI	3	0	2	120	centroid_9420	50.3792407	1.27E-12	centroid_9420	3.15E-05
centroid_9493	papC N-terminal domain protein	3	0	2	120	centroid_9493	50.3792407	1.27E-12	centroid_9493	3.15E-05
centroid_9494	papC N-terminal domain protein	3	0	2	120	centroid_9494	50.3792407	1.27E-12	centroid_9494	3.15E-05
centroid_9495	ı-negative pili assembly chaperone, N-terminal domain pr	3	0	2	120	centroid_9495	50.3792407	1.27E-12	centroid_9495	3.15E-05
centroid_9496	fimbrial family protein	3	0	2	120	centroid_9496	50.3792407	1.27E-12	centroid_9496	3.15E-05
centroid_9565	CFA/III pilin	3	0	2	120	centroid_9565	50.3792407	1.27E-12	centroid_9565	3.15E-05
centroid_9577	fimbrial family protein	3	0	2	120	centroid_9577	50.3792407	1.27E-12	centroid_9577	3.15E-05
centroid_9578	type VII secretion system (T7SS), usher family protein	3	0	2	120	centroid_9578	50.3792407	1.27E-12	centroid_9578	3.15E-05
centroid_9637	1-phosphate guanylyltransferase/mannose-6-phosphate	3	0	2	120	centroid_9637	50.3792407	1.27E-12	centroid_9637	3.15E-05
centroid_9638	glycosyl transferases group 1 family protein	3	0	2	120	centroid_9638	50.3792407	1.27E-12	centroid_9638	3.15E-05
centroid_9639	glycosyl transferases group 1 family protein	3	0	2	120	centroid_9639	50.3792407	1.27E-12	centroid_9639	3.15E-05
centroid_9640	putative membrane protein	3	0	2	120	centroid_9640	50.3792407	1.27E-12	centroid_9640	3.15E-05
centroid_9641	glycosyl transferases group 1 family protein	3	0	2	120	centroid_9641	50.3792407	1.27E-12	centroid_9641	3.15E-05
centroid_9642	glycosyl transferase 2 family protein	3	0	2	120	centroid_9642	50.3792407	1.27E-12	centroid_9642	3.15E-05
centroid_9464	conserved domain protein	3	1	2	119	centroid_9464	36.8268982	1.29E-09	centroid_9464	0.00012434
centroid_12550	conserved hypothetical protein	3	2	2	118	centroid_1255	28.7000868	8.45E-08	centroid_1255	0.000307
centroid_12631	transposase domain protein	3	2	2	118	centroid_1263	28.7000868	8.45E-08	centroid_1263	0.000307
centroid_12667	transposase IS66 family protein	3	2	2	118	centroid_1266	28.7000868	8.45E-08	centroid_1266	0.000307
centroid_13543	transposase domain protein	3	2	2	118	centroid_1354	28.7000868	8.45E-08	centroid_1354	0.000307
centroid_13549	transposase IS66 family protein	3	2	2	118	centroid_1354	28.7000868	8.45E-08	centroid_1354	0.000307
centroid_14473	putative minor structural subunit AalA	3	2	2	118	centroid_1447	28.7000868	8.45E-08	centroid_1447	0.000307
centroid_14475	helix-turn-helix domain protein	3	2	2	118	centroid_1447	28.7000868	8.45E-08	centroid_1447	0.000307
centroid_14506	transposase family protein	3	2	2	118	centroid_1450	28.7000868	8.45E-08	centroid_1450	0.000307
centroid_14507	transposase domain protein	3	2	2	118	centroid_1450	28.7000868	8.45E-08	centroid_1450	0.000307
centroid_8848	conserved hypothetical protein	3	2	2	118	centroid_8848	28.7000868	8.45E-08	centroid_8848	0.000307
centroid_9427	phage tail fibre repeat family protein	3	2	2	118	centroid_9427	28.7000868	8.45E-08	centroid_9427	0.000307
centroid_9562	ıdhesin biosynthesis transcription regulatory family protei	3	2	2	118	centroid_9562	28.7000868	8.45E-08	centroid_9562	0.000307
centroid_9563	bacterial regulatory helix-turn-helix, AraC family protein	3	2	2	118	centroid_9563	28.7000868	8.45E-08	centroid_9563	0.000307
centroid_9564	transglycosylase SLT domain protein	3	2	2	118	centroid_9564	28.7000868	8.45E-08	centroid_9564	0.000307
centroid_9566	repilin-type N-terminal cleavage/methylation domain prote	3	2	2	118	centroid_9566	28.7000868	8.45E-08	centroid_9566	0.000307
centroid_9598	hypothetical protein	3	2	2	118	centroid_9598	28.7000868	8.45E-08	centroid_9598	0.000307
centroid_9621	K88 fimbrial protein AD	3	2	2	118	centroid_9621	28.7000868	8.45E-08	centroid_9621	0.000307
centroid_9622	K88 fimbrial protein AB	3	2	2	118	centroid_9622	28.7000868	8.45E-08	centroid_9622	0.000307
centroid_9623	hypothetical protein	3	2	2	118	centroid_9623	28.7000868	8.45E-08	centroid_9623	0.000307
centroid_9624	fimbrial, major and minor subunit	3	2	2	118	centroid_9624	28.7000868	8.45E-08	centroid_9624	0.000307
centroid_9625	fimbrial, major and minor subunit	3	2	2	118	centroid_9625	28.7000868	8.45E-08	centroid_9625	0.000307
centroid_9626	putative k88 minor fimbrial subunit FaeF	3	2	2	118	centroid_9626	28.7000868	8.45E-08	centroid_9626	0.000307
centroid_9627	chaperone protein FaeE	3	2	2	118	centroid_9627	28.7000868	8.45E-08	centroid_9627	0.000307
centroid_9628	type VII secretion system (T7SS), usher family protein	3	2	2	118	centroid_9628	28.7000868	8.45E-08	centroid_9628	0.000307
centroid_9629	putative minor structural subunit AalA	3	2	2	118	centroid_9629	28.7000868	8.45E-08	centroid_9629	0.000307
centroid_9630	ıdhesin biosynthesis transcription regulatory family protei	3	2	2	118	centroid_9630	28.7000868	8.45E-08	centroid_9630	0.000307
centroid_9678	transposase family protein	3	2	2	118	centroid_9678	28.7000868	8.45E-08	centroid_9678	0.000307
centroid_9709	transposase family protein	3	2	2	118	centroid_9709	28.7000868	8.45E-08	centroid_9709	0.000307
centroid_9716	tn3 transposase DDE domain protein	3	2	2	118	centroid_9716	28.7000868	8.45E-08	centroid_9716	0.000307
centroid_9738	hypothetical protein	3	2	2	118	centroid_9738	28.7000868	8.45E-08	centroid_9738	0.000307
centroid_9747	putative transposase	3	2	2	118	centroid_9747	28.7000868	8.45E-08	centroid_9747	0.000307
centroid_9776	putative iS91 orf	3	2	2	118	centroid_9776	28.7000868	8.45E-08	centroid_9776	0.000307
centroid_10305	methylmalonyl-CoA mutase C-terminal domain protein	3	120	2	0	centroid_1030	26.6821223	2.40E-07	centroid_1030	0.00129032
centroid_10760	papC C-terminal domain protein	3	120	2	0	centroid_1076	26.6821223	2.40E-07	centroid_1076	0.00129032
centroid_10771	PTS system, glucose-like IIB component domain protein	3	120	2	0	centroid_1077	26.6821223	2.40E-07	centroid_1077	0.00129032
centroid_10772	PTS system beta-glucoside-specific EIIBCA component	3	120	2	0	centroid_1077	26.6821223	2.40E-07	centroid_1077	0.00129032
centroid_1151	fimbrial family protein	3	120	2	0	centroid_1151	26.6821223	2.40E-07	centroid_1151	0.00129032
centroid_11623	HokA domain protein	3	120	2	0	centroid_1162	26.6821223	2.40E-07	centroid_1162	0.00129032
centroid_12412	methylmalonyl-CoA mutase	3	120	2	0	centroid_1241	26.6821223	2.40E-07	centroid_1241	0.00129032
centroid_12547	mannosylglycerate hydrolase	3	120	2	0	centroid_1254	26.6821223	2.40E-07	centroid_1254	0.00129032
centroid_12682	ative phosphoethanolamine transferase YhbX domain pro	3	120	2	0	centroid_1268	26.6821223	2.40E-07	centroid_1268	0.00129032
centroid_1298	alpha amylase, catalytic domain protein	3	120	2	0	centroid_1298	26.6821223	2.40E-07	centroid_1298	0.00129032
centroid_13196	type VII secretion system (T7SS), usher family protein	3	120	2	0	centroid_1319	26.6821223	2.40E-07	centroid_1319	0.00129032
centroid_13615	type VII secretion system (T7SS), usher family protein	3	120	2	0	centroid_1361	26.6821223	2.40E-07	centroid_1361	0.00129032
centroid_13893	lamB porin family protein	3	120	2	0	centroid_1389	26.6821223	2.40E-07	centroid_1389	0.00129032
centroid_14001	6-phospho-beta-glucosidase BglB	3	120	2	0	centroid_1400	26.6821223	2.40E-07	centroid_1400	0.00129032
centroid_14201	transposase, YhgA-like family protein	3	120	2	0	centroid_1420	26.6821223	2.40E-07	centroid_1420	0.00129032
centroid_14261	inner membrane CbrB domain protein	3	120	2	0	centroid_1426	26.6821223	2.40E-07	centroid_1426	0.00129032
centroid_14270	type VII secretion system (T7SS), usher family protein	3	120	2	0	centroid_1427	26.6821223	2.40E-07	centroid_1427	0.00129032
centroid_14351	conserved hypothetical protein	3	120	2	0	centroid_1435	26.6821223	2.40E-07	centroid_1435	0.00129032
centroid_14624	SPFH domain / Band 7 family protein	3	120	2	0	centroid_1462	26.6821223	2.40E-07	centroid_1462	0.00129032
centroid_14723	hypothetical protein	3	120	2	0	centroid_1472	26.6821223	2.40E-07	centroid_1472	0.00129032
centroid_14738	conserved hypothetical protein	3	120	2	0	centroid_1473	26.6821223	2.40E-07	centroid_1473	0.00129032
centroid_14806	efflux transporter, RND family, MFP subunit	3	120	2	0	centroid_1480	26.6821223	2.40E-07	centroid_1480	0.00129032
centroid_16093	fimbrial family protein	3	120	2	0	centroid_1609	26.6821223	2.40E-07	centroid_1609	0.00129032
centroid_16439	leucine rich repeat family protein	3	120	2	0	centroid_1643	26.6821223	2.40E-07	centroid_1643	0.00129032
centroid_16929	conserved hypothetical protein	3	120	2	0	centroid_1692	26.6821223	2.40E-07	centroid_1692	0.00129032
centroid_16993	fimbrial family protein	3	120	2	0	centroid_1699	26.6821223	2.40E-07	centroid_1699	0.00129032
centroid_17220	gamma-glutamyltranspeptidase domain protein	3	120	2	0	centroid_1722	26.6821223	2.40E-07	centroid_1722	0.00129032
centroid_17323	bacterial regulatory, tetR family protein	3	120	2	0	centroid_1732	26.6821223	2.40E-07	centroid_1732	0.00129032
centroid_17324	S-type transcriptional repressor, C-terminal region family	3	120	2	0	centroid_1732	26.6821223	2.40E-07	centroid_1732	0.00129032
centroid_17427	beta galactosidase small chain family protein	3	120	2	0	centroid_1742	26.6821223	2.40E-07	centroid_1742	0.00129032
centroid_17456	S system, fructose subfamily, IIA component domain prot	3	120	2	0	centroid_1745	26.6821223	2.40E-07	centroid_1745	0.00129032
centroid_17457	PTS system, Fru family, IIC component domain protein	3	120	2	0	centroid_1745	26.6821223	2.40E-07	centroid_1745	0.00129032
centroid_1997	methylmalonyl-CoA mutase	3	120	2	0	centroid_1997	26.6821223	2.40E-07	centroid_1997	0.00129032
centroid_2003	arginine exporter protein ArgO	3	120	2	0	centroid_2003	26.6821223	2.40E-07	centroid_2003	0.00129032
centroid_2049	cryptic beta-glucoside bgl operon antiterminator	3	120	2	0	centroid_2049	26.6821223	2.40E-07	centroid_2049	0.00129032
centroid_2050	PTS system beta-glucoside-specific EIIBCA component	3	120	2	0	centroid_2050	26.6821223	2.40E-07	centroid_2050	0.00129032
centroid_2054	inner membrane protein CbrB	3	120	2	0	centroid_2054	26.6821223	2.40E-07	centroid_2054	0.00129032
centroid_2482	conserved hypothetical protein	3	120	2	0	centroid_2482	26.6821223	2.40E-07	centroid_2482	0.00129032
centroid_2483	putative acrEF/envCD operon repressor	3	120	2	0	centroid_2483	26.6821223	2.40E-07	centroid_2483	0.00129032
centroid_253	3-methyl-2-oxobutanoate hydroxymethyltransferase	3	120	2	0	centroid_253	26.6821223	2.40E-07	centroid_253	0.00129032
centroid_2543	protein HokA	3	120	2	0	centroid_2543	26.6821223	2.40E-07	centroid_2543	0.00129032
centroid_2858	serine transporter	3	120	2	0	centroid_2858	26.6821223	2.40E-07	centroid_2858	0.00129032
centroid_2959	osyl-D-glycerate transport/metabolism system repressor	3	120	2	0	centroid_2959	26.6821223	2.40E-07	centroid_2959	0.00129032

centroid_2960	heat-responsive suppressor HrsA	3	120	2	0	centroid_2960	26.6821223	2.40E-07	centroid_2960	0.00129032
centroid_2961	mannosylglycerate hydrolase	3	120	2	0	centroid_2961	26.6821223	2.40E-07	centroid_2961	0.00129032
centroid_3055	N-acetyltransferase family protein	3	120	2	0	centroid_3055	26.6821223	2.40E-07	centroid_3055	0.00129032
centroid_3410	ynbE-like lipofamily protein	3	120	2	0	centroid_3410	26.6821223	2.40E-07	centroid_3410	0.00129032
centroid_3632	regulatory protein SdiA	3	120	2	0	centroid_3632	26.6821223	2.40E-07	centroid_3632	0.00129032
centroid_3744	putative amino-acid metabolite efflux pump	3	120	2	0	centroid_3744	26.6821223	2.40E-07	centroid_3744	0.00129032
centroid_3745	marB family protein	3	120	2	0	centroid_3745	26.6821223	2.40E-07	centroid_3745	0.00129032
centroid_3822	conserved hypothetical protein	3	120	2	0	centroid_3822	26.6821223	2.40E-07	centroid_3822	0.00129032
centroid_4581	fimbrial family protein	3	120	2	0	centroid_4581	26.6821223	2.40E-07	centroid_4581	0.00129032
centroid_4655	HokA domain protein	3	120	2	0	centroid_4655	26.6821223	2.40E-07	centroid_4655	0.00129032
centroid_4810	cyanate hydratase	3	120	2	0	centroid_4810	26.6821223	2.40E-07	centroid_4810	0.00129032
centroid_511	cyanate transporter family protein	3	120	2	0	centroid_511	26.6821223	2.40E-07	centroid_511	0.00129032
centroid_512	cyanate hydratase	3	120	2	0	centroid_512	26.6821223	2.40E-07	centroid_512	0.00129032
centroid_513	carbonic anhydrase 1	3	120	2	0	centroid_513	26.6821223	2.40E-07	centroid_513	0.00129032
centroid_514	HTH-type transcriptional regulator CynR	3	120	2	0	centroid_514	26.6821223	2.40E-07	centroid_514	0.00129032
centroid_515	cytosine deaminase	3	120	2	0	centroid_515	26.6821223	2.40E-07	centroid_515	0.00129032
centroid_516	cytosine permease	3	120	2	0	centroid_516	26.6821223	2.40E-07	centroid_516	0.00129032
centroid_7318	putative acrEF/envCD operon repressor	3	120	2	0	centroid_7318	26.6821223	2.40E-07	centroid_7318	0.00129032
centroid_7642	6-phospho-beta-glucosidase BglB	3	120	2	0	centroid_7642	26.6821223	2.40E-07	centroid_7642	0.00129032
centroid_7716	S system, fructose subfamily, IIA component domain prot	3	120	2	0	centroid_7716	26.6821223	2.40E-07	centroid_7716	0.00129032
centroid_7717	heat-responsive suppressor HrsA domain protein	3	120	2	0	centroid_7717	26.6821223	2.40E-07	centroid_7717	0.00129032
centroid_7755	type VII secretion system (T7SS), usher family protein	3	120	2	0	centroid_7755	26.6821223	2.40E-07	centroid_7755	0.00129032
centroid_8060	PTS system sorbose-specific iic component family protein	3	120	2	0	centroid_8060	26.6821223	2.40E-07	centroid_8060	0.00129032
centroid_8062	ative phosphoethanolamine transferase YhbX domain pro	3	120	2	0	centroid_8062	26.6821223	2.40E-07	centroid_8062	0.00129032
centroid_8089	PRD domain protein	3	120	2	0	centroid_8089	26.6821223	2.40E-07	centroid_8089	0.00129032
centroid_8997	S system, glucose subfamily, IIA component domain prot	3	120	2	0	centroid_8997	26.6821223	2.40E-07	centroid_8997	0.00129032
centroid_8998	PTS system, glucose-like IIB component domain protein	3	120	2	0	centroid_8998	26.6821223	2.40E-07	centroid_8998	0.00129032
centroid_9024	DNA methylase family protein	3	120	2	0	centroid_9024	26.6821223	2.40E-07	centroid_9024	0.00129032
centroid_9028	yltransferase DNA adenine methyltransferase domain pr	3	120	2	0	centroid_9028	26.6821223	2.40E-07	centroid_9028	0.00129032
centroid_12596	proQ/FINO family protein	3	3	2	117	centroid_1259	23.2861374	1.40E-06	centroid_1259	0.00060636
centroid_14469	proQ/FINO family protein	3	3	2	117	centroid_1446	23.2861374	1.40E-06	centroid_1446	0.00060636
centroid_5722	conserved hypothetical protein	3	3	2	117	centroid_5722	23.2861374	1.40E-06	centroid_5722	0.00060636
centroid_8330	type IV leader peptidase family protein	3	3	2	117	centroid_8330	23.2861374	1.40E-06	centroid_8330	0.00060636
centroid_8331	putative cofJ	3	3	2	117	centroid_8331	23.2861374	1.40E-06	centroid_8331	0.00060636
centroid_8332	type II secretion system (T2SS), F family protein	3	3	2	117	centroid_8332	23.2861374	1.40E-06	centroid_8332	0.00060636
centroid_8333	type II/IV secretion system family protein	3	3	2	117	centroid_8333	23.2861374	1.40E-06	centroid_8333	0.00060636
centroid_8334	putative lngG	3	3	2	117	centroid_8334	23.2861374	1.40E-06	centroid_8334	0.00060636
centroid_8335	putative pilus biosynthesis protein	3	3	2	117	centroid_8335	23.2861374	1.40E-06	centroid_8335	0.00060636
centroid_8336	putative pilus biosynthesis transmembrane anchor protein	3	3	2	117	centroid_8336	23.2861374	1.40E-06	centroid_8336	0.00060636
centroid_8337	bacterial type II and III secretion system family protein	3	3	2	117	centroid_8337	23.2861374	1.40E-06	centroid_8337	0.00060636
centroid_8338	toxin co-regulated pilus biosynthesis Q family protein	3	3	2	117	centroid_8338	23.2861374	1.40E-06	centroid_8338	0.00060636
centroid_8346	hypothetical protein	3	3	2	117	centroid_8346	23.2861374	1.40E-06	centroid_8346	0.00060636
centroid_8349	resolvase, N terminal domain protein	3	3	2	117	centroid_8349	23.2861374	1.40E-06	centroid_8349	0.00060636
centroid_8859	conserved hypothetical protein	3	3	2	117	centroid_8859	23.2861374	1.40E-06	centroid_8859	0.00060636
centroid_9418	helix-turn-helix domain protein	3	3	2	117	centroid_9418	23.2861374	1.40E-06	centroid_9418	0.00060636
centroid_9567	proQ/FINO family protein	3	3	2	117	centroid_9567	23.2861374	1.40E-06	centroid_9567	0.00060636
centroid_9680	hypothetical protein	3	3	2	117	centroid_9680	23.2861374	1.40E-06	centroid_9680	0.00060636
centroid_9681	ABC transporter family protein	3	3	2	117	centroid_9681	23.2861374	1.40E-06	centroid_9681	0.00060636
centroid_9682	biotin-lipoyl like family protein	3	3	2	117	centroid_9682	23.2861374	1.40E-06	centroid_9682	0.00060636
centroid_9683	peptidase S24-like family protein	3	3	2	117	centroid_9683	23.2861374	1.40E-06	centroid_9683	0.00060636
centroid_9684	impB/mucB/samB family protein	3	3	2	117	centroid_9684	23.2861374	1.40E-06	centroid_9684	0.00060636
centroid_9686	conserved hypothetical protein	3	3	2	117	centroid_9686	23.2861374	1.40E-06	centroid_9686	0.00060636
centroid_9687	parB/RepB/Spo0J family partition domain protein	3	3	2	117	centroid_9687	23.2861374	1.40E-06	centroid_9687	0.00060636
centroid_9689	itoxin Phd_YefM, type II toxin-antitoxin system family prot	3	3	2	117	centroid_9689	23.2861374	1.40E-06	centroid_9689	0.00060636
centroid_9690	addiction module toxin, RelE/StbE family protein	3	3	2	117	centroid_9690	23.2861374	1.40E-06	centroid_9690	0.00060636
centroid_9732	conserved hypothetical protein	3	3	2	117	centroid_9732	23.2861374	1.40E-06	centroid_9732	0.00060636
centroid_9745	transposase family protein	3	3	2	117	centroid_9745	23.2861374	1.40E-06	centroid_9745	0.00060636
centroid_11096	putative type III secretion protein	3	4	2	116	centroid_1109	19.4224803	1.05E-05	centroid_1109	0.00104786
centroid_7198	UTRA domain protein	3	4	2	116	centroid_7198	19.4224803	1.05E-05	centroid_7198	0.00104786
centroid_7199	uvate-dependent sugar phosphotransferase system, EIIA	3	4	2	116	centroid_7199	19.4224803	1.05E-05	centroid_7199	0.00104786
centroid_7200	PTS system, Lactose/Cellobiose specific IIB subunit	3	4	2	116	centroid_7200	19.4224803	1.05E-05	centroid_7200	0.00104786
centroid_7201	S system sugar-specific permease component family pro	3	4	2	116	centroid_7201	19.4224803	1.05E-05	centroid_7201	0.00104786
centroid_7202	GY family of carbohydrate kinase, N-terminal domain pro	3	4	2	116	centroid_7202	19.4224803	1.05E-05	centroid_7202	0.00104786
centroid_7203	phosphotransferase system, HPr-related proteins	3	4	2	116	centroid_7203	19.4224803	1.05E-05	centroid_7203	0.00104786
centroid_7204	ketose-bisphosphate aldolase family protein	3	4	2	116	centroid_7204	19.4224803	1.05E-05	centroid_7204	0.00104786
centroid_8352	hypothetical protein	3	4	2	116	centroid_8352	19.4224803	1.05E-05	centroid_8352	0.00104786
centroid_8353	hypothetical protein	3	4	2	116	centroid_8353	19.4224803	1.05E-05	centroid_8353	0.00104786
centroid_8438	caudovirales tail fibre assembly family protein	3	4	2	116	centroid_8438	19.4224803	1.05E-05	centroid_8438	0.00104786
centroid_9444	PTS family galactitol porter, component IIC domain protein	3	4	2	116	centroid_9444	19.4224803	1.05E-05	centroid_9444	0.00104786
centroid_9445	S system sugar-specific permease component family pro	3	4	2	116	centroid_9445	19.4224803	1.05E-05	centroid_9445	0.00104786
centroid_9446	conserved hypothetical protein	3	4	2	116	centroid_9446	19.4224803	1.05E-05	centroid_9446	0.00104786
centroid_9685	conserved hypothetical protein	3	4	2	116	centroid_9685	19.4224803	1.05E-05	centroid_9685	0.00104786
centroid_9688	cobQ/CobB/MinD/ParA nucleotide binding domain protein	3	4	2	116	centroid_9688	19.4224803	1.05E-05	centroid_9688	0.00104786
centroid_9721	type I restriction enzyme R N terminus family protein	3	4	2	116	centroid_9721	19.4224803	1.05E-05	centroid_9721	0.00104786
centroid_9731	conserved hypothetical protein	3	4	2	116	centroid_9731	19.4224803	1.05E-05	centroid_9731	0.00104786
centroid_9740	HTH-like domain protein	3	4	2	116	centroid_9740	19.4224803	1.05E-05	centroid_9740	0.00104786
centroid_1085	evolved beta-galactosidase subunit alpha	3	119	2	1	centroid_1085	16.9377561	3.86E-05	centroid_1085	0.00380803
centroid_10980	type VII secretion system (T7SS), usher family protein	3	119	2	1	centroid_1098	16.9377561	3.86E-05	centroid_1098	0.00380803
centroid_1148	n-negative pili assembly chaperone, N-terminal domain pr	3	119	2	1	centroid_1148	16.9377561	3.86E-05	centroid_1148	0.00380803
centroid_1150	conserved hypothetical protein	3	119	2	1	centroid_1150	16.9377561	3.86E-05	centroid_1150	0.00380803
centroid_11582	conserved hypothetical protein	3	119	2	1	centroid_1158	16.9377561	3.86E-05	centroid_1158	0.00380803
centroid_12428	inner membrane protein YqiK	3	119	2	1	centroid_1242	16.9377561	3.86E-05	centroid_1242	0.00380803
centroid_12573	fimbrial domain protein	3	119	2	1	centroid_1257	16.9377561	3.86E-05	centroid_1257	0.00380803
centroid_12605	fimbrial family protein	3	119	2	1	centroid_1260	16.9377561	3.86E-05	centroid_1260	0.00380803
centroid_12973	lysR substrate binding domain protein	3	119	2	1	centroid_1297	16.9377561	3.86E-05	centroid_1297	0.00380803
centroid_13350	cupin family protein	3	119	2	1	centroid_1335	16.9377561	3.86E-05	centroid_1335	0.00380803
centroid_14271	type VII secretion system (T7SS), usher family protein	3	119	2	1	centroid_1427	16.9377561	3.86E-05	centroid_1427	0.00380803
centroid_14282	putative membrane protein	3	119	2	1	centroid_1428	16.9377561	3.86E-05	centroid_1428	0.00380803
centroid_14718	conserved hypothetical protein	3	119	2	1	centroid_1471	16.9377561	3.86E-05	centroid_1471	0.00380803
centroid_15311	sdiA-regulated family protein	3	119	2	1	centroid_1531	16.9377561	3.86E-05	centroid_1531	0.00380803
centroid_15312	sdiA-regulated family protein	3	119	2	1	centroid_1531	16.9377561	3.86E-05	centroid_1531	0.00380803
centroid_17157	glucose inhibited division A family protein	3	119	2	1	centroid_1715	16.9377561	3.86E-05	centroid_1715	0.00380803
centroid_17169	pyridine nucleotide-disulfide oxidoreductase family protein	3	119	2	1	centroid_1716	16.9377561	3.86E-05	centroid_1716	0.00380803
centroid_18594	type VII secretion system (T7SS), usher family protein	3	119	2	1	centroid_1859	16.9377561	3.86E-05	centroid_1859	0.00380803
centroid_2051	cryptic outer membrane porin BglH	3	119	2	1	centroid_2051	16.9377561	3.86E-05	centroid_2051	0.00380803
centroid_2484	efflux transporter, RND family, MFP subunit	3	119	2	1	centroid_2484	16.9377561	3.86E-05	centroid_2484	0.00380803
centroid_3380	alpha amylase, catalytic domain protein	3	119	2	1	centroid_3380	16.9377561	3.86E-05	centroid_3380	0.00380803
centroid_3671	conserved hypothetical protein	3	119	2	1	centroid_3671	16.9377561	3.86E-05	centroid_3671	0.00380803
centroid_3672	conserved hypothetical protein	3	119	2	1	centroid_3672	16.9377561	3.86E-05	centroid_3672	0.00380803
centroid_3808	cupin family protein	3	119	2	1	centroid_3808	16.9377561	3.86E-05	centroid_3808	0.00380803
centroid_4723	conserved hypothetical protein	3	119	2	1	centroid_4723	16.9377561	3.86E-05	centroid_4723	0.00380803
centroid_5930	putative alpha amylase	3	119	2	1	centroid_5930	16.9377561	3.86E-05	centroid_5930	0.00380803
centroid_729	phosphonate metabolism protein PhnP	3	119	2	1	centroid_729	16.9377561	3.86E-05	centroid_729	0.00380803
centroid_7817	major Facilitator Superfamily protein	3	119	2	1	centroid_7817	16.9377561	3.86E-05	centroid_7817	0.00380803
centroid_8520	conserved hypothetical protein	3	119	2	1	centroid_8520	16.9377561	3.86E-05	centroid_8520	0.00380803
centroid_10630	prokaryotic metallothionein family protein	3	5	2	115	centroid_1063	16.5278334	4.79E-05	centroid_1063	0.00165548
centroid_12899	putative caudovirales tail fiber assembly protein	3	5	2	115	centroid_1289	16.5278334	4.79E-05	centroid_1289	0.00165548

centroid_13880	gram-negative porin family protein	3	5	2	115	centroid_1388	16.5278334	4.79E-05	centroid_1388	0.00165548
centroid_9473	outer membrane protein N	3	5	2	115	centroid_947	16.5278334	4.79E-05	centroid_947	0.00165548
centroid_9666	conserved hypothetical protein	3	5	2	115	centroid_966	16.5278334	4.79E-05	centroid_966	0.00165548
centroid_9713	putative domain protein	3	5	2	115	centroid_971	16.5278334	4.79E-05	centroid_971	0.00165548
centroid_9718	conserved hypothetical protein	3	5	2	115	centroid_971	16.5278334	4.79E-05	centroid_971	0.00165548
centroid_9719	conserved hypothetical protein	3	5	2	115	centroid_971	16.5278334	4.79E-05	centroid_971	0.00165548
centroid_9720	conserved hypothetical protein	3	5	2	115	centroid_972	16.5278334	4.79E-05	centroid_972	0.00165548
centroid_9790	putative domain protein	3	5	2	115	centroid_979	16.5278334	4.79E-05	centroid_979	0.00165548
centroid_10279	orn/Lys/Arg decarboxylase, major domain protein	2	120	3	0	centroid_1027	50.3792407	1.27E-12	centroid_1027	3.15E-05
centroid_1034	conserved hypothetical protein	2	120	3	0	centroid_103	50.3792407	1.27E-12	centroid_103	3.15E-05
centroid_11	swarming motility protein YbiA	2	120	3	0	centroid_11	50.3792407	1.27E-12	centroid_11	3.15E-05
centroid_11247	putrescine-ornithine antiporter	2	120	3	0	centroid_1124	50.3792407	1.27E-12	centroid_1124	3.15E-05
centroid_12471	putative acyl transferase domain protein	2	120	3	0	centroid_1247	50.3792407	1.27E-12	centroid_1247	3.15E-05
centroid_12472	putative acyl transferase domain protein	2	120	3	0	centroid_1247	50.3792407	1.27E-12	centroid_1247	3.15E-05
centroid_12548	glycosyl hydrolases family 38 C-terminal domain protein	2	120	3	0	centroid_1254	50.3792407	1.27E-12	centroid_1254	3.15E-05
centroid_12961	leucine Rich repeats family protein	2	120	3	0	centroid_1296	50.3792407	1.27E-12	centroid_1296	3.15E-05
centroid_13999	bacterial transferase hexapeptide family protein	2	120	3	0	centroid_1399	50.3792407	1.27E-12	centroid_1399	3.15E-05
centroid_15515	glcNAc-PI de-N-acetylase family protein	2	120	3	0	centroid_1551	50.3792407	1.27E-12	centroid_1551	3.15E-05
centroid_16360	eamA-like transporter family protein	2	120	3	0	centroid_1636	50.3792407	1.27E-12	centroid_1636	3.15E-05
centroid_16440	leucine Rich repeats family protein	2	120	3	0	centroid_1644	50.3792407	1.27E-12	centroid_1644	3.15E-05
centroid_16484	p-aminobenzoyl-glutamate transport protein	2	120	3	0	centroid_1648	50.3792407	1.27E-12	centroid_1648	3.15E-05
centroid_16885	orn/Lys/Arg decarboxylase, C-terminal domain protein	2	120	3	0	centroid_1688	50.3792407	1.27E-12	centroid_1688	3.15E-05
centroid_17768	ulp1 protease family, C-terminal catalytic domain protein	2	120	3	0	centroid_1776	50.3792407	1.27E-12	centroid_1776	3.15E-05
centroid_18273	ulp1 protease family, C-terminal catalytic domain protein	2	120	3	0	centroid_1827	50.3792407	1.27E-12	centroid_1827	3.15E-05
centroid_1878	conserved hypothetical protein	2	120	3	0	centroid_187	50.3792407	1.27E-12	centroid_187	3.15E-05
centroid_2481	DNA methylase family protein	2	120	3	0	centroid_248	50.3792407	1.27E-12	centroid_248	3.15E-05
centroid_3062	leucine Rich repeats family protein	2	120	3	0	centroid_306	50.3792407	1.27E-12	centroid_306	3.15E-05
centroid_3427	p-aminobenzoyl-glutamate transport protein	2	120	3	0	centroid_342	50.3792407	1.27E-12	centroid_342	3.15E-05
centroid_4270	conserved hypothetical protein	2	120	3	0	centroid_427	50.3792407	1.27E-12	centroid_427	3.15E-05
centroid_493	glcNAc-PI de-N-acetylase family protein	2	120	3	0	centroid_493	50.3792407	1.27E-12	centroid_493	3.15E-05
centroid_494	glycosyl transferase 2 family protein	2	120	3	0	centroid_494	50.3792407	1.27E-12	centroid_494	3.15E-05
centroid_495	putative acyl transferase	2	120	3	0	centroid_495	50.3792407	1.27E-12	centroid_495	3.15E-05
centroid_7341	conserved hypothetical protein	2	120	3	0	centroid_734	50.3792407	1.27E-12	centroid_734	3.15E-05
centroid_7342	conserved hypothetical domain protein	2	120	3	0	centroid_734	50.3792407	1.27E-12	centroid_734	3.15E-05
centroid_8142	putative acyl transferase	2	120	3	0	centroid_814	50.3792407	1.27E-12	centroid_814	3.15E-05
centroid_15215	-negative pili assembly chaperone, N-terminal domain pr	2	119	3	1	centroid_1521	36.8268982	1.29E-09	centroid_1521	0.00012434
centroid_18162	putative ubiquitin carboxyl-terminal hydrolase 45	2	119	3	1	centroid_1816	36.8268982	1.29E-09	centroid_1816	0.00012434
centroid_189	conserved hypothetical protein	2	119	3	1	centroid_189	36.8268982	1.29E-09	centroid_189	0.00012434
centroid_4299	-negative pili assembly chaperone, N-terminal domain pr	2	119	3	1	centroid_429	36.8268982	1.29E-09	centroid_429	0.00012434
centroid_6216	conserved hypothetical protein	2	119	3	1	centroid_621	36.8268982	1.29E-09	centroid_621	0.00012434
centroid_7652	orn/Lys/Arg decarboxylase, major domain protein	2	119	3	1	centroid_765	36.8268982	1.29E-09	centroid_765	0.00012434
centroid_10762	dnaJ domain protein	2	118	3	2	centroid_1076	28.7000868	8.45E-08	centroid_1076	0.000307
centroid_10763	putative heat shock DnaJ domain-containing protein	2	118	3	2	centroid_1076	28.7000868	8.45E-08	centroid_1076	0.000307
centroid_14212	hsp70 family protein	2	118	3	2	centroid_1421	28.7000868	8.45E-08	centroid_1421	0.000307
centroid_1447	sel1 repeat family protein	2	118	3	2	centroid_144	28.7000868	8.45E-08	centroid_144	0.000307
centroid_1448	conserved hypothetical protein	2	118	3	2	centroid_144	28.7000868	8.45E-08	centroid_144	0.000307
centroid_1449	dnaJ domain protein	2	118	3	2	centroid_144	28.7000868	8.45E-08	centroid_144	0.000307
centroid_1450	sel1 repeat family protein	2	118	3	2	centroid_145	28.7000868	8.45E-08	centroid_145	0.000307
centroid_1451	conserved hypothetical protein	2	118	3	2	centroid_145	28.7000868	8.45E-08	centroid_145	0.000307
centroid_1452	dnaJ domain protein	2	118	3	2	centroid_145	28.7000868	8.45E-08	centroid_145	0.000307
centroid_1453	hsp70 family protein	2	118	3	2	centroid_145	28.7000868	8.45E-08	centroid_145	0.000307
centroid_1544	fimbrial family protein	2	118	3	2	centroid_154	28.7000868	8.45E-08	centroid_154	0.000307
centroid_17321	hsp70 family protein	2	118	3	2	centroid_1732	28.7000868	8.45E-08	centroid_1732	0.000307
centroid_17322	putative truncated dnaK protein	2	118	3	2	centroid_1732	28.7000868	8.45E-08	centroid_1732	0.000307
centroid_484	protein SbmA	2	118	3	2	centroid_484	28.7000868	8.45E-08	centroid_484	0.000307
centroid_13488	hypothetical protein	2	0	3	120	centroid_1348	26.6821223	2.40E-07	centroid_1348	0.00129032
centroid_13492	conserved hypothetical protein	2	0	3	120	centroid_1349	26.6821223	2.40E-07	centroid_1349	0.00129032
centroid_13503	putative mobA/MobL protein	2	0	3	120	centroid_1350	26.6821223	2.40E-07	centroid_1350	0.00129032
centroid_14312	inner membrane protein YcfZ	2	0	3	120	centroid_1431	26.6821223	2.40E-07	centroid_1431	0.00129032
centroid_14320	conserved hypothetical protein	2	0	3	120	centroid_1432	26.6821223	2.40E-07	centroid_1432	0.00129032
centroid_14386	RNA ligase family protein	2	0	3	120	centroid_1438	26.6821223	2.40E-07	centroid_1438	0.00129032
centroid_14387	conserved hypothetical protein	2	0	3	120	centroid_1438	26.6821223	2.40E-07	centroid_1438	0.00129032
centroid_14388	AAA domain protein	2	0	3	120	centroid_1438	26.6821223	2.40E-07	centroid_1438	0.00129032
centroid_14406	conserved hypothetical protein	2	0	3	120	centroid_1440	26.6821223	2.40E-07	centroid_1440	0.00129032
centroid_14430	invasion protein InvA	2	0	3	120	centroid_1443	26.6821223	2.40E-07	centroid_1443	0.00129032
centroid_14431	bacterial type II and III secretion system family protein	2	0	3	120	centroid_1443	26.6821223	2.40E-07	centroid_1443	0.00129032
centroid_14464	hypothetical protein	2	0	3	120	centroid_1446	26.6821223	2.40E-07	centroid_1446	0.00129032
centroid_14472	conserved hypothetical protein	2	0	3	120	centroid_1447	26.6821223	2.40E-07	centroid_1447	0.00129032
centroid_14503	conserved hypothetical protein	2	0	3	120	centroid_1450	26.6821223	2.40E-07	centroid_1450	0.00129032
centroid_14521	oglucomutase/phosphomannomutase, C-terminal domain	2	0	3	120	centroid_1452	26.6821223	2.40E-07	centroid_1452	0.00129032
centroid_17822	bacterial regulatory helix-turn-helix, AraC family protein	2	0	3	120	centroid_1782	26.6821223	2.40E-07	centroid_1782	0.00129032
centroid_17823	amidotransferase family protein	2	0	3	120	centroid_1782	26.6821223	2.40E-07	centroid_1782	0.00129032
centroid_17824	conserved hypothetical protein	2	0	3	120	centroid_1782	26.6821223	2.40E-07	centroid_1782	0.00129032
centroid_17921	conserved hypothetical protein	2	0	3	120	centroid_1792	26.6821223	2.40E-07	centroid_1792	0.00129032
centroid_17972	AAA domain protein	2	0	3	120	centroid_1797	26.6821223	2.40E-07	centroid_1797	0.00129032
centroid_17975	on-LEE-encoded type III secreted effector domain protei	2	0	3	120	centroid_1797	26.6821223	2.40E-07	centroid_1797	0.00129032
centroid_17976	hypothetical protein	2	0	3	120	centroid_1797	26.6821223	2.40E-07	centroid_1797	0.00129032
centroid_18220	conserved hypothetical protein	2	0	3	120	centroid_1822	26.6821223	2.40E-07	centroid_1822	0.00129032
centroid_18231	conserved hypothetical protein	2	0	3	120	centroid_1823	26.6821223	2.40E-07	centroid_1823	0.00129032
centroid_18236	autotransporter beta-domain protein	2	0	3	120	centroid_1823	26.6821223	2.40E-07	centroid_1823	0.00129032
centroid_18261	conserved hypothetical protein	2	0	3	120	centroid_1826	26.6821223	2.40E-07	centroid_1826	0.00129032
centroid_8759	conserved hypothetical protein	2	0	3	120	centroid_875	26.6821223	2.40E-07	centroid_875	0.00129032
centroid_8760	conserved hypothetical protein	2	0	3	120	centroid_876	26.6821223	2.40E-07	centroid_876	0.00129032
centroid_8761	sopA-like catalytic domain protein	2	0	3	120	centroid_876	26.6821223	2.40E-07	centroid_876	0.00129032
centroid_8764	S system sugar-specific permease component family pro	2	0	3	120	centroid_876	26.6821223	2.40E-07	centroid_876	0.00129032
centroid_8765	PTS system, Lactose/Cellobiose specific IIB subunit	2	0	3	120	centroid_876	26.6821223	2.40E-07	centroid_876	0.00129032
centroid_8766	sulfatase family protein	2	0	3	120	centroid_876	26.6821223	2.40E-07	centroid_876	0.00129032
centroid_8803	pentapeptide repeats family protein	2	0	3	120	centroid_880	26.6821223	2.40E-07	centroid_880	0.00129032
centroid_8804	ccdB family protein	2	0	3	120	centroid_880	26.6821223	2.40E-07	centroid_880	0.00129032
centroid_8805	post-segregation antitoxin CcdA family protein	2	0	3	120	centroid_880	26.6821223	2.40E-07	centroid_880	0.00129032
centroid_8825	conserved hypothetical protein	2	0	3	120	centroid_882	26.6821223	2.40E-07	centroid_882	0.00129032
centroid_8826	AMP-binding enzyme family protein	2	0	3	120	centroid_882	26.6821223	2.40E-07	centroid_882	0.00129032
centroid_8827	putative membrane protein	2	0	3	120	centroid_882	26.6821223	2.40E-07	centroid_882	0.00129032
centroid_8832	putative lipoprotein	2	0	3	120	centroid_883	26.6821223	2.40E-07	centroid_883	0.00129032
centroid_8840	putative acetyltransferase	2	0	3	120	centroid_884	26.6821223	2.40E-07	centroid_884	0.00129032
centroid_8841	conserved hypothetical protein	2	0	3	120	centroid_884	26.6821223	2.40E-07	centroid_884	0.00129032
centroid_8856	type III secretion regulator YopN/LcrE/InvE/MxiC	2	0	3	120	centroid_885	26.6821223	2.40E-07	centroid_885	0.00129032
centroid_8890	conserved hypothetical protein	2	0	3	120	centroid_889	26.6821223	2.40E-07	centroid_889	0.00129032
centroid_8891	acyl transferase domain protein	2	0	3	120	centroid_889	26.6821223	2.40E-07	centroid_889	0.00129032
centroid_8911	bacterial regulatory, tetR family protein	2	0	3	120	centroid_891	26.6821223	2.40E-07	centroid_891	0.00129032
centroid_8912	efflux transporter, RND family, MFP subunit	2	0	3	120	centroid_891	26.6821223	2.40E-07	centroid_891	0.00129032
centroid_8913	D transporter, hydrophobe/amphiphile efflux-1 family prot	2	0	3	120	centroid_891	26.6821223	2.40E-07	centroid_891	0.00129032
centroid_8914	ansporter, outer membrane factor (OMF) lipo, NodT famil	2	0	3	120	centroid_891	26.6821223	2.40E-07	centroid_891	0.00129032
centroid_8915	drug resistance transporter, Bcr/CflA subfamily protein	2	0	3	120	centroid_891	26.6821223	2.40E-07	centroid_891	0.00129032
centroid_8938	conserved hypothetical protein	2	0	3	120	centroid_893	26.6821223	2.40E-07	centroid_893	0.00129032
centroid_8939	sodium:dicarboxylate symporter family protein	2	0	3	120	centroid_893	26.6821223	2.40E-07	centroid_893	0.00129032
centroid_8940	fumarate hydratase class I, aerobic	2	0	3	120	centroid_894	26.6821223	2.40E-07	centroid_894	0.00129032

centroid_8941	cob(I)yrinic acid a,c-diamide adenosyltransferase	2	0	3	120	centroid_8941	26.6821223	2.40E-07	centroid_8941	0.00129032
centroid_8942	bacterial regulatory helix-turn-helix , lysR family protein	2	0	3	120	centroid_8942	26.6821223	2.40E-07	centroid_8942	0.00129032
centroid_8943	fimbrial family protein	2	0	3	120	centroid_8943	26.6821223	2.40E-07	centroid_8943	0.00129032
centroid_8944	type VII secretion system (T7SS), usher family protein	2	0	3	120	centroid_8944	26.6821223	2.40E-07	centroid_8944	0.00129032
centroid_8945	-negative pili assembly chaperone, C-terminal domain pr	2	0	3	120	centroid_8945	26.6821223	2.40E-07	centroid_8945	0.00129032
centroid_8946	fimbrial family protein	2	0	3	120	centroid_8946	26.6821223	2.40E-07	centroid_8946	0.00129032
centroid_8951	conserved hypothetical protein	2	0	3	120	centroid_8951	26.6821223	2.40E-07	centroid_8951	0.00129032
centroid_8965	amidotransferase family protein	2	0	3	120	centroid_8965	26.6821223	2.40E-07	centroid_8965	0.00129032
centroid_8966	beta-lactamase superfamily domain protein	2	0	3	120	centroid_8966	26.6821223	2.40E-07	centroid_8966	0.00129032
centroid_8973	outer membrane autotransporter barrel domain protein	2	0	3	120	centroid_8973	26.6821223	2.40E-07	centroid_8973	0.00129032
centroid_8986	sugar (and other) transporter family protein	2	0	3	120	centroid_8986	26.6821223	2.40E-07	centroid_8986	0.00129032
centroid_9000	lysR substrate binding domain protein	2	0	3	120	centroid_9000	26.6821223	2.40E-07	centroid_9000	0.00129032
centroid_9001	oxidoreductase	2	0	3	120	centroid_9001	26.6821223	2.40E-07	centroid_9001	0.00129032
centroid_9002	putative ybiJ	2	0	3	120	centroid_9002	26.6821223	2.40E-07	centroid_9002	0.00129032
centroid_9003	bacterial regulatory helix-turn-helix , lysR family protein	2	0	3	120	centroid_9003	26.6821223	2.40E-07	centroid_9003	0.00129032
centroid_9004	conserved hypothetical protein	2	0	3	120	centroid_9004	26.6821223	2.40E-07	centroid_9004	0.00129032
centroid_9005	sugar (and other) transporter family protein	2	0	3	120	centroid_9005	26.6821223	2.40E-07	centroid_9005	0.00129032
centroid_9046	ynbE-like lipofamily protein	2	0	3	120	centroid_9046	26.6821223	2.40E-07	centroid_9046	0.00129032
centroid_9096	putative amino-acid metabolite efflux pump	2	0	3	120	centroid_9096	26.6821223	2.40E-07	centroid_9096	0.00129032
centroid_9118	molybdenum-pterin binding domain protein	2	0	3	120	centroid_9118	26.6821223	2.40E-07	centroid_9118	0.00129032
centroid_9121	leucine Rich repeats family protein	2	0	3	120	centroid_9121	26.6821223	2.40E-07	centroid_9121	0.00129032
centroid_9130	conserved hypothetical protein	2	0	3	120	centroid_9130	26.6821223	2.40E-07	centroid_9130	0.00129032
centroid_9131	conserved hypothetical protein	2	0	3	120	centroid_9131	26.6821223	2.40E-07	centroid_9131	0.00129032
centroid_9132	conserved hypothetical protein	2	0	3	120	centroid_9132	26.6821223	2.40E-07	centroid_9132	0.00129032
centroid_9133	type VI secretion system effector, Hcp1 family protein	2	0	3	120	centroid_9133	26.6821223	2.40E-07	centroid_9133	0.00129032
centroid_9235	conserved hypothetical protein	2	0	3	120	centroid_9235	26.6821223	2.40E-07	centroid_9235	0.00129032
centroid_9251	resolvase, N terminal domain protein	2	0	3	120	centroid_9251	26.6821223	2.40E-07	centroid_9251	0.00129032
centroid_9268	type VI secretion system effector, Hcp1 family protein	2	0	3	120	centroid_9268	26.6821223	2.40E-07	centroid_9268	0.00129032
centroid_9269	colicin-E7 immunity protein	2	0	3	120	centroid_9269	26.6821223	2.40E-07	centroid_9269	0.00129032
centroid_9270	colicin-E7 immunity protein	2	0	3	120	centroid_9270	26.6821223	2.40E-07	centroid_9270	0.00129032
centroid_9271	colicin-E7 immunity protein	2	0	3	120	centroid_9271	26.6821223	2.40E-07	centroid_9271	0.00129032
centroid_9298	evolved beta-galactosidase subunit alpha	2	0	3	120	centroid_9298	26.6821223	2.40E-07	centroid_9298	0.00129032
centroid_9312	conserved hypothetical protein	2	0	3	120	centroid_9312	26.6821223	2.40E-07	centroid_9312	0.00129032
centroid_9322	-negative pili assembly chaperone, C-terminal domain pr	2	0	3	120	centroid_9322	26.6821223	2.40E-07	centroid_9322	0.00129032
centroid_9323	type VII secretion system (T7SS), usher family protein	2	0	3	120	centroid_9323	26.6821223	2.40E-07	centroid_9323	0.00129032
centroid_9325	ype I restriction modification DNA specificity domain prote	2	0	3	120	centroid_9325	26.6821223	2.40E-07	centroid_9325	0.00129032
centroid_9326	helix-turn-helix domain protein	2	0	3	120	centroid_9326	26.6821223	2.40E-07	centroid_9326	0.00129032
centroid_9350	conserved hypothetical protein	2	0	3	120	centroid_9350	26.6821223	2.40E-07	centroid_9350	0.00129032
centroid_9356	poxvirus D5 protein-like family protein	2	0	3	120	centroid_9356	26.6821223	2.40E-07	centroid_9356	0.00129032
centroid_9357	conserved hypothetical protein	2	0	3	120	centroid_9357	26.6821223	2.40E-07	centroid_9357	0.00129032
centroid_9359	ash family protein	2	0	3	120	centroid_9359	26.6821223	2.40E-07	centroid_9359	0.00129032
centroid_9361	putative glyco3, capsid size determination protein Sid	2	0	3	120	centroid_9361	26.6821223	2.40E-07	centroid_9361	0.00129032
centroid_9364	conserved hypothetical protein	2	0	3	120	centroid_9364	26.6821223	2.40E-07	centroid_9364	0.00129032
centroid_9365	conserved hypothetical protein	2	0	3	120	centroid_9365	26.6821223	2.40E-07	centroid_9365	0.00129032
centroid_9366	integrase	2	0	3	120	centroid_9366	26.6821223	2.40E-07	centroid_9366	0.00129032
centroid_9380	PAAR motif family protein	2	0	3	120	centroid_9380	26.6821223	2.40E-07	centroid_9380	0.00129032
centroid_9381	rhs element Vgr family protein	2	0	3	120	centroid_9381	26.6821223	2.40E-07	centroid_9381	0.00129032
centroid_9382	phage-related baseplate assembly family protein	2	0	3	120	centroid_9382	26.6821223	2.40E-07	centroid_9382	0.00129032
centroid_9383	conserved hypothetical protein	2	0	3	120	centroid_9383	26.6821223	2.40E-07	centroid_9383	0.00129032
centroid_9384	conserved hypothetical protein	2	0	3	120	centroid_9384	26.6821223	2.40E-07	centroid_9384	0.00129032
centroid_9385	peptidase C39 family protein	2	0	3	120	centroid_9385	26.6821223	2.40E-07	centroid_9385	0.00129032
centroid_9390	conserved hypothetical protein	2	0	3	120	centroid_9390	26.6821223	2.40E-07	centroid_9390	0.00129032
centroid_9391	conserved hypothetical protein	2	0	3	120	centroid_9391	26.6821223	2.40E-07	centroid_9391	0.00129032
centroid_9395	conserved hypothetical protein	2	0	3	120	centroid_9395	26.6821223	2.40E-07	centroid_9395	0.00129032
centroid_9396	hypothetical protein	2	0	3	120	centroid_9396	26.6821223	2.40E-07	centroid_9396	0.00129032
centroid_9397	conserved hypothetical protein	2	0	3	120	centroid_9397	26.6821223	2.40E-07	centroid_9397	0.00129032
centroid_9398	conserved hypothetical protein	2	0	3	120	centroid_9398	26.6821223	2.40E-07	centroid_9398	0.00129032
centroid_9401	conserved hypothetical protein	2	0	3	120	centroid_9401	26.6821223	2.40E-07	centroid_9401	0.00129032
centroid_9403	DNA N-6-adenine-methyltransferase family protein	2	0	3	120	centroid_9403	26.6821223	2.40E-07	centroid_9403	0.00129032
centroid_9405	flagellin	2	0	3	120	centroid_9405	26.6821223	2.40E-07	centroid_9405	0.00129032
centroid_9407	conserved hypothetical protein	2	0	3	120	centroid_9407	26.6821223	2.40E-07	centroid_9407	0.00129032
centroid_9410	hypothetical protein	2	0	3	120	centroid_9410	26.6821223	2.40E-07	centroid_9410	0.00129032
centroid_9411	hypothetical protein	2	0	3	120	centroid_9411	26.6821223	2.40E-07	centroid_9411	0.00129032
centroid_9412	PAAR motif family protein	2	0	3	120	centroid_9412	26.6821223	2.40E-07	centroid_9412	0.00129032
centroid_9426	conserved hypothetical protein	2	0	3	120	centroid_9426	26.6821223	2.40E-07	centroid_9426	0.00129032
centroid_9428	conserved hypothetical protein	2	0	3	120	centroid_9428	26.6821223	2.40E-07	centroid_9428	0.00129032
centroid_9433	putative exported protein	2	0	3	120	centroid_9433	26.6821223	2.40E-07	centroid_9433	0.00129032
centroid_9434	conserved hypothetical protein	2	0	3	120	centroid_9434	26.6821223	2.40E-07	centroid_9434	0.00129032
centroid_9435	conserved hypothetical protein	2	0	3	120	centroid_9435	26.6821223	2.40E-07	centroid_9435	0.00129032
centroid_9436	conserved hypothetical protein	2	0	3	120	centroid_9436	26.6821223	2.40E-07	centroid_9436	0.00129032
centroid_9437	phage major capsid E family protein	2	0	3	120	centroid_9437	26.6821223	2.40E-07	centroid_9437	0.00129032
centroid_9438	conserved hypothetical protein	2	0	3	120	centroid_9438	26.6821223	2.40E-07	centroid_9438	0.00129032
centroid_9439	hypothetical protein	2	0	3	120	centroid_9439	26.6821223	2.40E-07	centroid_9439	0.00129032
centroid_9440	conserved hypothetical protein	2	0	3	120	centroid_9440	26.6821223	2.40E-07	centroid_9440	0.00129032
centroid_9441	conserved hypothetical protein	2	0	3	120	centroid_9441	26.6821223	2.40E-07	centroid_9441	0.00129032
centroid_9442	conserved hypothetical protein	2	0	3	120	centroid_9442	26.6821223	2.40E-07	centroid_9442	0.00129032
centroid_9443	conserved hypothetical protein	2	0	3	120	centroid_9443	26.6821223	2.40E-07	centroid_9443	0.00129032
centroid_9459	relaxase/Mobilisation nuclease domain protein	2	0	3	120	centroid_9459	26.6821223	2.40E-07	centroid_9459	0.00129032
centroid_9460	conserved hypothetical protein	2	0	3	120	centroid_9460	26.6821223	2.40E-07	centroid_9460	0.00129032
centroid_9461	tetratricopeptide repeat family protein	2	0	3	120	centroid_9461	26.6821223	2.40E-07	centroid_9461	0.00129032
centroid_9462	hypothetical protein	2	0	3	120	centroid_9462	26.6821223	2.40E-07	centroid_9462	0.00129032
centroid_9463	hypothetical protein	2	0	3	120	centroid_9463	26.6821223	2.40E-07	centroid_9463	0.00129032
centroid_9469	conserved hypothetical protein	2	0	3	120	centroid_9469	26.6821223	2.40E-07	centroid_9469	0.00129032
centroid_9470	putative predicted protein	2	0	3	120	centroid_9470	26.6821223	2.40E-07	centroid_9470	0.00129032
centroid_9476	hypothetical protein	2	0	3	120	centroid_9476	26.6821223	2.40E-07	centroid_9476	0.00129032
centroid_9477	conserved hypothetical protein	2	0	3	120	centroid_9477	26.6821223	2.40E-07	centroid_9477	0.00129032
centroid_9478	HNH endonuclease family protein	2	0	3	120	centroid_9478	26.6821223	2.40E-07	centroid_9478	0.00129032
centroid_9479	bacteriophage lysis family protein	2	0	3	120	centroid_9479	26.6821223	2.40E-07	centroid_9479	0.00129032
centroid_9484	hage tail tape measure protein, TP901 family, core region	2	0	3	120	centroid_9484	26.6821223	2.40E-07	centroid_9484	0.00129032
centroid_9485	conserved hypothetical protein	2	0	3	120	centroid_9485	26.6821223	2.40E-07	centroid_9485	0.00129032
centroid_9486	conserved hypothetical protein	2	0	3	120	centroid_9486	26.6821223	2.40E-07	centroid_9486	0.00129032
centroid_9487	conserved hypothetical protein	2	0	3	120	centroid_9487	26.6821223	2.40E-07	centroid_9487	0.00129032
centroid_9488	conserved hypothetical protein	2	0	3	120	centroid_9488	26.6821223	2.40E-07	centroid_9488	0.00129032
centroid_9489	conserved hypothetical protein	2	0	3	120	centroid_9489	26.6821223	2.40E-07	centroid_9489	0.00129032
centroid_9490	conserved hypothetical protein	2	0	3	120	centroid_9490	26.6821223	2.40E-07	centroid_9490	0.00129032
centroid_9497	conserved hypothetical protein	2	0	3	120	centroid_9497	26.6821223	2.40E-07	centroid_9497	0.00129032
centroid_9498	reverse transcriptase family protein	2	0	3	120	centroid_9498	26.6821223	2.40E-07	centroid_9498	0.00129032
centroid_9499	reverse transcriptase family protein	2	0	3	120	centroid_9499	26.6821223	2.40E-07	centroid_9499	0.00129032
centroid_9500	phage integrase family protein	2	0	3	120	centroid_9500	26.6821223	2.40E-07	centroid_9500	0.00129032
centroid_9503	hypothetical protein	2	0	3	120	centroid_9503	26.6821223	2.40E-07	centroid_9503	0.00129032
centroid_9505	resolvase, N terminal domain protein	2	0	3	120	centroid_9505	26.6821223	2.40E-07	centroid_9505	0.00129032
centroid_9506	conserved hypothetical protein	2	0	3	120	centroid_9506	26.6821223	2.40E-07	centroid_9506	0.00129032
centroid_9507	phage-like baseplate assembly domain protein	2	0	3	120	centroid_9507	26.6821223	2.40E-07	centroid_9507	0.00129032
centroid_9508	hypothetical protein	2	0	3	120	centroid_9508	26.6821223	2.40E-07	centroid_9508	0.00129032
centroid_9509	putative zinc-dependent metallopeptidase	2	0	3	120	centroid_9509	26.6821223	2.40E-07	centroid_9509	0.00129032
centroid_9511	conserved hypothetical protein	2	0	3	120	centroid_9511	26.6821223	2.40E-07	centroid_9511	0.00129032
centroid_9512	conserved hypothetical protein	2	0	3	120	centroid_9512	26.6821223	2.40E-07	centroid_9512	0.00129032

centroid_9513	conserved hypothetical protein	2	0	3	120	centroid_951	26.6821223	2.40E-07	centroid_951	0.00129032
centroid_9514	rhs element Vgr family protein	2	0	3	120	centroid_951	26.6821223	2.40E-07	centroid_951	0.00129032
centroid_9515	evfW domain protein	2	0	3	120	centroid_951	26.6821223	2.40E-07	centroid_951	0.00129032
centroid_9516	conserved hypothetical protein	2	0	3	120	centroid_951	26.6821223	2.40E-07	centroid_951	0.00129032
centroid_9517	type VI secretion system effector, Hcp1 family protein	2	0	3	120	centroid_951	26.6821223	2.40E-07	centroid_951	0.00129032
centroid_9518	conserved hypothetical protein	2	0	3	120	centroid_951	26.6821223	2.40E-07	centroid_951	0.00129032
centroid_9519	conserved hypothetical protein	2	0	3	120	centroid_951	26.6821223	2.40E-07	centroid_951	0.00129032
centroid_9520	gene 25-like lysozyme family protein	2	0	3	120	centroid_952	26.6821223	2.40E-07	centroid_952	0.00129032
centroid_9521	conserved hypothetical protein	2	0	3	120	centroid_952	26.6821223	2.40E-07	centroid_952	0.00129032
centroid_9522	conserved hypothetical protein	2	0	3	120	centroid_952	26.6821223	2.40E-07	centroid_952	0.00129032
centroid_9523	FHA domain protein	2	0	3	120	centroid_952	26.6821223	2.40E-07	centroid_952	0.00129032
centroid_9524	type VI secretion lipofamily protein	2	0	3	120	centroid_952	26.6821223	2.40E-07	centroid_952	0.00129032
centroid_9525	conserved hypothetical protein	2	0	3	120	centroid_952	26.6821223	2.40E-07	centroid_952	0.00129032
centroid_9526	conserved hypothetical protein	2	0	3	120	centroid_952	26.6821223	2.40E-07	centroid_952	0.00129032
centroid_9527	impA domain family protein	2	0	3	120	centroid_952	26.6821223	2.40E-07	centroid_952	0.00129032
centroid_9528	type VI secretion system effector, Hcp1 family protein	2	0	3	120	centroid_952	26.6821223	2.40E-07	centroid_952	0.00129032
centroid_9529	putative aminopeptidase	2	0	3	120	centroid_952	26.6821223	2.40E-07	centroid_952	0.00129032
centroid_9535	conserved hypothetical protein	2	0	3	120	centroid_953	26.6821223	2.40E-07	centroid_953	0.00129032
centroid_9536	hypothetical protein	2	0	3	120	centroid_953	26.6821223	2.40E-07	centroid_953	0.00129032
centroid_9537	WYL domain protein	2	0	3	120	centroid_953	26.6821223	2.40E-07	centroid_953	0.00129032
centroid_9540	grating conjugative element , PFL_4695 family domain pro	2	0	3	120	centroid_954	26.6821223	2.40E-07	centroid_954	0.00129032
centroid_9541	conserved hypothetical protein	2	0	3	120	centroid_954	26.6821223	2.40E-07	centroid_954	0.00129032
centroid_9543	phage integrase family protein	2	0	3	120	centroid_954	26.6821223	2.40E-07	centroid_954	0.00129032
centroid_9546	conserved hypothetical protein	2	0	3	120	centroid_954	26.6821223	2.40E-07	centroid_954	0.00129032
centroid_9547	phage integrase family protein	2	0	3	120	centroid_954	26.6821223	2.40E-07	centroid_954	0.00129032
centroid_9548	conserved hypothetical protein	2	0	3	120	centroid_954	26.6821223	2.40E-07	centroid_954	0.00129032
centroid_9549	conserved hypothetical protein	2	0	3	120	centroid_954	26.6821223	2.40E-07	centroid_954	0.00129032
centroid_9574	conserved hypothetical protein	2	0	3	120	centroid_957	26.6821223	2.40E-07	centroid_957	0.00129032
centroid_9579	hypothetical protein	2	0	3	120	centroid_957	26.6821223	2.40E-07	centroid_957	0.00129032
centroid_9580	rhs element Vgr family protein	2	0	3	120	centroid_958	26.6821223	2.40E-07	centroid_958	0.00129032
centroid_9581	conserved hypothetical protein	2	0	3	120	centroid_958	26.6821223	2.40E-07	centroid_958	0.00129032
centroid_9582	conserved hypothetical protein	2	0	3	120	centroid_958	26.6821223	2.40E-07	centroid_958	0.00129032
centroid_9583	hypothetical protein	2	0	3	120	centroid_958	26.6821223	2.40E-07	centroid_958	0.00129032
centroid_9584	conserved hypothetical protein	2	0	3	120	centroid_958	26.6821223	2.40E-07	centroid_958	0.00129032
centroid_9585	winged helix-turn-helix DNA-binding family protein	2	0	3	120	centroid_958	26.6821223	2.40E-07	centroid_958	0.00129032
centroid_9586	addiction module antidote protein, HigA family	2	0	3	120	centroid_958	26.6821223	2.40E-07	centroid_958	0.00129032
centroid_9588	integrase family protein	2	0	3	120	centroid_958	26.6821223	2.40E-07	centroid_958	0.00129032
centroid_9602	conserved hypothetical protein	2	0	3	120	centroid_960	26.6821223	2.40E-07	centroid_960	0.00129032
centroid_9603	conserved hypothetical protein	2	0	3	120	centroid_960	26.6821223	2.40E-07	centroid_960	0.00129032
centroid_9604	conserved hypothetical protein	2	0	3	120	centroid_960	26.6821223	2.40E-07	centroid_960	0.00129032
centroid_9605	conserved hypothetical protein	2	0	3	120	centroid_960	26.6821223	2.40E-07	centroid_960	0.00129032
centroid_9606	conserved hypothetical protein	2	0	3	120	centroid_960	26.6821223	2.40E-07	centroid_960	0.00129032
centroid_9607	sel1 repeat family protein	2	0	3	120	centroid_960	26.6821223	2.40E-07	centroid_960	0.00129032
centroid_9608	conserved hypothetical protein	2	0	3	120	centroid_960	26.6821223	2.40E-07	centroid_960	0.00129032
centroid_9609	conserved hypothetical protein	2	0	3	120	centroid_960	26.6821223	2.40E-07	centroid_960	0.00129032
centroid_9610	conserved hypothetical protein	2	0	3	120	centroid_961	26.6821223	2.40E-07	centroid_961	0.00129032
centroid_9611	hypothetical protein	2	0	3	120	centroid_961	26.6821223	2.40E-07	centroid_961	0.00129032
centroid_9612	conserved hypothetical protein	2	0	3	120	centroid_961	26.6821223	2.40E-07	centroid_961	0.00129032
centroid_9616	divergent AAA domain protein	2	0	3	120	centroid_961	26.6821223	2.40E-07	centroid_961	0.00129032
centroid_9617	conserved hypothetical protein	2	0	3	120	centroid_961	26.6821223	2.40E-07	centroid_961	0.00129032
centroid_9634	hep_Hag family protein	2	0	3	120	centroid_963	26.6821223	2.40E-07	centroid_963	0.00129032
centroid_9644	putative dNA transfer protein p33	2	0	3	120	centroid_964	26.6821223	2.40E-07	centroid_964	0.00129032
centroid_9646	conserved hypothetical protein	2	0	3	120	centroid_964	26.6821223	2.40E-07	centroid_964	0.00129032
centroid_9647	conserved hypothetical protein	2	0	3	120	centroid_964	26.6821223	2.40E-07	centroid_964	0.00129032
centroid_9648	perC transcriptional activator family protein	2	0	3	120	centroid_964	26.6821223	2.40E-07	centroid_964	0.00129032
centroid_9649	conserved hypothetical protein	2	0	3	120	centroid_964	26.6821223	2.40E-07	centroid_964	0.00129032
centroid_9650	plasmid pRiA4b ORF-3-like family protein	2	0	3	120	centroid_965	26.6821223	2.40E-07	centroid_965	0.00129032
centroid_9651	conserved hypothetical protein	2	0	3	120	centroid_965	26.6821223	2.40E-07	centroid_965	0.00129032
centroid_9656	pentapeptide repeats family protein	2	0	3	120	centroid_965	26.6821223	2.40E-07	centroid_965	0.00129032
centroid_9668	phage integrase family protein	2	0	3	120	centroid_966	26.6821223	2.40E-07	centroid_966	0.00129032
centroid_9669	phage integrase family protein	2	0	3	120	centroid_966	26.6821223	2.40E-07	centroid_966	0.00129032
centroid_9670	phage integrase family protein	2	0	3	120	centroid_967	26.6821223	2.40E-07	centroid_967	0.00129032
centroid_9671	conserved hypothetical protein	2	0	3	120	centroid_967	26.6821223	2.40E-07	centroid_967	0.00129032
centroid_9672	hypothetical protein	2	0	3	120	centroid_967	26.6821223	2.40E-07	centroid_967	0.00129032
centroid_9673	conserved hypothetical protein	2	0	3	120	centroid_967	26.6821223	2.40E-07	centroid_967	0.00129032
centroid_9674	conserved hypothetical protein	2	0	3	120	centroid_967	26.6821223	2.40E-07	centroid_967	0.00129032
centroid_9675	conserved hypothetical protein	2	0	3	120	centroid_967	26.6821223	2.40E-07	centroid_967	0.00129032
centroid_9696	lysis S family protein	2	0	3	120	centroid_969	26.6821223	2.40E-07	centroid_969	0.00129032
centroid_9700	conserved hypothetical protein	2	0	3	120	centroid_970	26.6821223	2.40E-07	centroid_970	0.00129032
centroid_9701	conserved hypothetical protein	2	0	3	120	centroid_970	26.6821223	2.40E-07	centroid_970	0.00129032
centroid_9702	modification methylase PvuII	2	0	3	120	centroid_970	26.6821223	2.40E-07	centroid_970	0.00129032
centroid_9703	conserved hypothetical protein	2	0	3	120	centroid_970	26.6821223	2.40E-07	centroid_970	0.00129032
centroid_9704	conjugal transfer TraD family protein	2	0	3	120	centroid_970	26.6821223	2.40E-07	centroid_970	0.00129032
centroid_9726	phage integrase family protein	2	0	3	120	centroid_972	26.6821223	2.40E-07	centroid_972	0.00129032
centroid_9727	putative membrane protein	2	0	3	120	centroid_972	26.6821223	2.40E-07	centroid_972	0.00129032
centroid_9728	conserved hypothetical protein	2	0	3	120	centroid_972	26.6821223	2.40E-07	centroid_972	0.00129032
centroid_9729	hypothetical protein	2	0	3	120	centroid_972	26.6821223	2.40E-07	centroid_972	0.00129032
centroid_9743	hypothetical protein	2	0	3	120	centroid_974	26.6821223	2.40E-07	centroid_974	0.00129032
centroid_9760	conserved hypothetical protein	2	0	3	120	centroid_976	26.6821223	2.40E-07	centroid_976	0.00129032
centroid_9761	P22AR C-terminal domain protein	2	0	3	120	centroid_976	26.6821223	2.40E-07	centroid_976	0.00129032
centroid_9792	prophage P4 integrase domain protein	2	0	3	120	centroid_979	26.6821223	2.40E-07	centroid_979	0.00129032
centroid_9796	recombinase family protein	2	0	3	120	centroid_979	26.6821223	2.40E-07	centroid_979	0.00129032
centroid_14221	papC N-terminal domain protein	2	117	3	3	centroid_1422	23.2861374	1.40E-06	centroid_1422	0.00060636
centroid_1483	orn/Lys/Arg decarboxylase, major domain protein	2	117	3	3	centroid_148	23.2861374	1.40E-06	centroid_148	0.00060636
centroid_1548	conserved hypothetical protein	2	117	3	3	centroid_154	23.2861374	1.40E-06	centroid_154	0.00060636
centroid_10278	type VII secretion system (T7SS), usher family protein	2	116	3	4	centroid_1027	19.4224803	1.05E-05	centroid_1027	0.00104786
centroid_10331	type VII secretion system (T7SS), usher family protein	2	116	3	4	centroid_1033	19.4224803	1.05E-05	centroid_1033	0.00104786
centroid_11884	papC N-terminal domain protein	2	116	3	4	centroid_1188	19.4224803	1.05E-05	centroid_1188	0.00104786
centroid_15214	putative membrane protein	2	116	3	4	centroid_1521	19.4224803	1.05E-05	centroid_1521	0.00104786
centroid_1546	conserved hypothetical protein	2	116	3	4	centroid_154	19.4224803	1.05E-05	centroid_154	0.00104786
centroid_1547	fimbrial family protein	2	116	3	4	centroid_154	19.4224803	1.05E-05	centroid_154	0.00104786
centroid_6913	HEAT repeats family protein	2	116	3	4	centroid_691	19.4224803	1.05E-05	centroid_691	0.00104786
centroid_8444	-negative pili assembly chaperone, N-terminal domain pr	2	116	3	4	centroid_844	19.4224803	1.05E-05	centroid_844	0.00104786
centroid_12933	single-stranded DNA-binding family protein	2	1	3	119	centroid_1293	16.9377561	3.86E-05	centroid_1293	0.00380803
centroid_15949	conserved hypothetical protein	2	1	3	119	centroid_1594	16.9377561	3.86E-05	centroid_1594	0.00380803
centroid_8320	peptidase C39 family protein	2	1	3	119	centroid_832	16.9377561	3.86E-05	centroid_832	0.00380803
centroid_8321	conserved hypothetical protein	2	1	3	119	centroid_832	16.9377561	3.86E-05	centroid_832	0.00380803
centroid_8322	putative zinc-dependent metallopeptidase domain protein	2	1	3	119	centroid_832	16.9377561	3.86E-05	centroid_832	0.00380803
centroid_8323	putative zinc-dependent metallopeptidase domain protein	2	1	3	119	centroid_832	16.9377561	3.86E-05	centroid_832	0.00380803
centroid_8324	conserved hypothetical protein	2	1	3	119	centroid_832	16.9377561	3.86E-05	centroid_832	0.00380803
centroid_8358	hypothetical protein	2	1	3	119	centroid_835	16.9377561	3.86E-05	centroid_835	0.00380803
centroid_8359	hypothetical protein	2	1	3	119	centroid_835	16.9377561	3.86E-05	centroid_835	0.00380803
centroid_9031	nosyl-l-methionine hydroxide adenosyltransferase family	2	1	3	119	centroid_903	16.9377561	3.86E-05	centroid_903	0.00380803
centroid_9161	putative membrane protein	2	1	3	119	centroid_916	16.9377561	3.86E-05	centroid_916	0.00380803
centroid_9181	putative yfdA	2	1	3	119	centroid_918	16.9377561	3.86E-05	centroid_918	0.00380803
centroid_9239	phage tail-collar fibre family protein	2	1	3	119	centroid_923	16.9377561	3.86E-05	centroid_923	0.00380803
centroid_9340	conserved hypothetical protein	2	1	3	119	centroid_934	16.9377561	3.86E-05	centroid_934	0.00380803

centroid_9358	conserved hypothetical protein	2	1	3	119	centroid_9358	16.9377561	3.86E-05	centroid_9358	0.00380803
centroid_9360	prophage CP4-57 regulatory family protein	2	1	3	119	centroid_9360	16.9377561	3.86E-05	centroid_9360	0.00380803
centroid_9362	ogr/Delta-like zinc finger family protein	2	1	3	119	centroid_9362	16.9377561	3.86E-05	centroid_9362	0.00380803
centroid_9363	polarity suppression protein	2	1	3	119	centroid_9363	16.9377561	3.86E-05	centroid_9363	0.00380803
centroid_9399	conserved hypothetical protein	2	1	3	119	centroid_9399	16.9377561	3.86E-05	centroid_9399	0.00380803
centroid_9400	conserved hypothetical protein	2	1	3	119	centroid_9400	16.9377561	3.86E-05	centroid_9400	0.00380803
centroid_9402	conserved hypothetical protein	2	1	3	119	centroid_9402	16.9377561	3.86E-05	centroid_9402	0.00380803
centroid_9550	putative prophage protein	2	1	3	119	centroid_9550	16.9377561	3.86E-05	centroid_9550	0.00380803
centroid_9554	conserved hypothetical protein	2	1	3	119	centroid_9554	16.9377561	3.86E-05	centroid_9554	0.00380803
centroid_9590	phage integrase family protein	2	1	3	119	centroid_9590	16.9377561	3.86E-05	centroid_9590	0.00380803
centroid_9591	conserved hypothetical protein	2	1	3	119	centroid_9591	16.9377561	3.86E-05	centroid_9591	0.00380803
centroid_9653	conserved hypothetical protein	2	1	3	119	centroid_9653	16.9377561	3.86E-05	centroid_9653	0.00380803
centroid_9654	major Facilitator Superfamily protein	2	1	3	119	centroid_9654	16.9377561	3.86E-05	centroid_9654	0.00380803
centroid_9655	conserved hypothetical protein	2	1	3	119	centroid_9655	16.9377561	3.86E-05	centroid_9655	0.00380803
centroid_9748	bacterial Ig-like domain family protein	2	1	3	119	centroid_9748	16.9377561	3.86E-05	centroid_9748	0.00380803
centroid_9793	conserved hypothetical protein	2	1	3	119	centroid_9793	16.9377561	3.86E-05	centroid_9793	0.00380803
centroid_17241	conserved hypothetical protein	2	115	3	5	centroid_1724	16.5278334	4.79E-05	centroid_1724	0.00165548
centroid_4046	conserved hypothetical protein	2	115	3	5	centroid_4046	16.5278334	4.79E-05	centroid_4046	0.00165548
centroid_4202	YibA domain protein	2	115	3	5	centroid_4202	16.5278334	4.79E-05	centroid_4202	0.00165548
centroid_1029	putative yfaH	1	120	4	0	centroid_1029	75.0285167	4.64E-18	centroid_1029	5.16E-07
centroid_12417	outer membrane protein C	1	120	4	0	centroid_1241	75.0285167	4.64E-18	centroid_1241	5.16E-07
centroid_14253	conserved hypothetical protein	1	120	4	0	centroid_1425	75.0285167	4.64E-18	centroid_1425	5.16E-07
centroid_14412	ribbon-helix-helix , copG family protein	1	120	4	0	centroid_1441	75.0285167	4.64E-18	centroid_1441	5.16E-07
centroid_18326	2-keto-3-deoxy-galactonokinase family protein	1	120	4	0	centroid_1832	75.0285167	4.64E-18	centroid_1832	5.16E-07
centroid_18327	2-keto-3-deoxy-galactonokinase family protein	1	120	4	0	centroid_1832	75.0285167	4.64E-18	centroid_1832	5.16E-07
centroid_2074	FCD domain protein	1	120	4	0	centroid_2074	75.0285167	4.64E-18	centroid_2074	5.16E-07
centroid_2075	2-keto-3-deoxy-galactonokinase family protein	1	120	4	0	centroid_2075	75.0285167	4.64E-18	centroid_2075	5.16E-07
centroid_2076	2-dehydro-3-deoxy-6-phosphogalactonate aldolase	1	120	4	0	centroid_2076	75.0285167	4.64E-18	centroid_2076	5.16E-07
centroid_2077	D-galactonate dehydratase	1	120	4	0	centroid_2077	75.0285167	4.64E-18	centroid_2077	5.16E-07
centroid_2078	D-galactonate transporter	1	120	4	0	centroid_2078	75.0285167	4.64E-18	centroid_2078	5.16E-07
centroid_311	sugar efflux transporter A	1	120	4	0	centroid_311	75.0285167	4.64E-18	centroid_311	5.16E-07
centroid_8456	2-keto-3-deoxy-galactonokinase family protein	1	120	4	0	centroid_8456	75.0285167	4.64E-18	centroid_8456	5.16E-07
centroid_10308	conserved hypothetical protein	1	119	4	1	centroid_1030	59.0820313	1.51E-14	centroid_1030	2.56E-06
centroid_13075	conserved hypothetical protein	1	119	4	1	centroid_1307	59.0820313	1.51E-14	centroid_1307	2.56E-06
centroid_15451	conserved hypothetical protein	1	119	4	1	centroid_1545	59.0820313	1.51E-14	centroid_1545	2.56E-06
centroid_1583	conserved hypothetical protein	1	119	4	1	centroid_1583	59.0820313	1.51E-14	centroid_1583	2.56E-06
centroid_1666	conserved hypothetical protein	1	119	4	1	centroid_1666	59.0820313	1.51E-14	centroid_1666	2.56E-06
centroid_2052	e-6-phosphate isomerases/6-phosphogluconolactonase fa	1	119	4	1	centroid_2052	59.0820313	1.51E-14	centroid_2052	2.56E-06
centroid_7208	conserved hypothetical protein	1	119	4	1	centroid_7208	59.0820313	1.51E-14	centroid_7208	2.56E-06
centroid_9962	gamma-glutamyltranspeptidase domain protein	1	119	4	1	centroid_9962	59.0820313	1.51E-14	centroid_9962	2.56E-06
centroid_18532	type VI secretion system effector, Hcp1 family protein	1	116	4	4	centroid_1853	35.16877	3.02E-09	centroid_1853	3.52E-05
centroid_848	type VI secretion system effector, Hcp1 family protein	1	116	4	4	centroid_848	35.16877	3.02E-09	centroid_848	3.52E-05
centroid_11169	conserved hypothetical protein	1	115	4	5	centroid_1116	30.7423871	2.95E-08	centroid_1116	6.29E-05
centroid_7792	thanolamine utilization - propanediol utilization family prote	1	113	4	7	centroid_7792	24.3065939	8.22E-07	centroid_7792	0.00016237
centroid_8736	thanolamine utilization - propanediol utilization family prote	1	113	4	7	centroid_8736	24.3065939	8.22E-07	centroid_8736	0.00016237
centroid_13232	integrase core domain protein	1	112	4	8	centroid_1323	21.8943968	2.88E-06	centroid_1323	0.00024187
centroid_17674	conserved hypothetical protein	1	110	4	10	centroid_1767	18.1059966	2.09E-05	centroid_1767	0.00048229
centroid_2839	conserved hypothetical protein	1	110	4	10	centroid_2839	18.1059966	2.09E-05	centroid_2839	0.00048229
centroid_2840	repair family protein	1	110	4	10	centroid_2840	18.1059966	2.09E-05	centroid_2840	0.00048229
centroid_4254	hypothetical protein	1	110	4	10	centroid_4254	18.1059966	2.09E-05	centroid_4254	0.00048229
centroid_4506	conserved hypothetical protein	1	110	4	10	centroid_4506	18.1059966	2.09E-05	centroid_4506	0.00048229
centroid_5513	integrase core domain protein	1	110	4	10	centroid_5513	18.1059966	2.09E-05	centroid_5513	0.00048229
centroid_12444	uvate-dependent sugar phosphotransferase system, EIIA	1	109	4	11	centroid_1244	16.5916982	4.64E-05	centroid_1244	0.00065302
centroid_14010	alpha-glucosidase YihQ domain protein	1	109	4	11	centroid_1401	16.5916982	4.64E-05	centroid_1401	0.00065302
centroid_14132	integrase core domain protein	1	109	4	11	centroid_1413	16.5916982	4.64E-05	centroid_1413	0.00065302
centroid_17686	putative domain protein	1	109	4	11	centroid_1768	16.5916982	4.64E-05	centroid_1768	0.00065302
centroid_2249	corbate-specific phosphotransferase enzyme IIA compon	1	109	4	11	centroid_2249	16.5916982	4.64E-05	centroid_2249	0.00065302
centroid_1012	sensory box protein	1	108	4	12	centroid_1012	15.267375	9.33E-05	centroid_1012	0.00086448
centroid_1013	response regulator	1	108	4	12	centroid_1013	15.267375	9.33E-05	centroid_1013	0.00086448
centroid_1014	acetate CoA-transferase subunit alpha	1	108	4	12	centroid_1014	15.267375	9.33E-05	centroid_1014	0.00086448
centroid_1015	acetate CoA-transferase subunit beta	1	108	4	12	centroid_1015	15.267375	9.33E-05	centroid_1015	0.00086448
centroid_1016	short-chain fatty acids transporter	1	108	4	12	centroid_1016	15.267375	9.33E-05	centroid_1016	0.00086448
centroid_1017	acetyl-CoA C-acetyltransferase family protein	1	108	4	12	centroid_1017	15.267375	9.33E-05	centroid_1017	0.00086448
centroid_10599	putative predicted inner membrane protein	1	108	4	12	centroid_1059	15.267375	9.33E-05	centroid_1059	0.00086448
centroid_11719	integrase core domain protein	1	108	4	12	centroid_1171	15.267375	9.33E-05	centroid_1171	0.00086448
centroid_13637	integrase core domain protein	1	108	4	12	centroid_1363	15.267375	9.33E-05	centroid_1363	0.00086448
centroid_17355	conserved hypothetical protein	1	108	4	12	centroid_1735	15.267375	9.33E-05	centroid_1735	0.00086448
centroid_3651	conserved hypothetical protein	1	108	4	12	centroid_3651	15.267375	9.33E-05	centroid_3651	0.00086448
centroid_4246	integrase core domain protein	1	108	4	12	centroid_4246	15.267375	9.33E-05	centroid_4246	0.00086448
centroid_5438	conserved hypothetical protein	1	108	4	12	centroid_5438	15.267375	9.33E-05	centroid_5438	0.00086448
centroid_5520	integrase core domain protein	1	108	4	12	centroid_5520	15.267375	9.33E-05	centroid_5520	0.00086448
centroid_5995	integrase core domain protein	1	108	4	12	centroid_5995	15.267375	9.33E-05	centroid_5995	0.00086448
centroid_7693	ative signal transduction histidine-kinase atoS domain pro	1	108	4	12	centroid_7693	15.267375	9.33E-05	centroid_7693	0.00086448
centroid_7694	sensory box protein	1	108	4	12	centroid_7694	15.267375	9.33E-05	centroid_7694	0.00086448
centroid_8264	thiolase, N-terminal domain protein	1	108	4	12	centroid_8264	15.267375	9.33E-05	centroid_8264	0.00086448
centroid_8265	acetyl-CoA C-acetyltransferase family protein	1	108	4	12	centroid_8265	15.267375	9.33E-05	centroid_8265	0.00086448
centroid_1060	fimbrial family protein	0	120	5	0	centroid_1060	100.31467	1.30E-23	centroid_1060	4.26E-09
centroid_11948	GY family of carbohydrate kinase, N-terminal domain pro	0	120	5	0	centroid_1194	100.31467	1.30E-23	centroid_1194	4.26E-09
centroid_1240	type VII secretion system (T7SS), usher family protein	0	120	5	0	centroid_1240	100.31467	1.30E-23	centroid_1240	4.26E-09
centroid_1241	fimbrial family protein	0	120	5	0	centroid_1241	100.31467	1.30E-23	centroid_1241	4.26E-09
centroid_13867	type VII secretion system (T7SS), usher family protein	0	120	5	0	centroid_1386	100.31467	1.30E-23	centroid_1386	4.26E-09
centroid_14011	class II Aldolase and Adducin N-terminal domain protein	0	120	5	0	centroid_1401	100.31467	1.30E-23	centroid_1401	4.26E-09
centroid_1582	conserved hypothetical protein	0	120	5	0	centroid_1582	100.31467	1.30E-23	centroid_1582	4.26E-09
centroid_16905	major MR/P fimbria domain protein	0	120	5	0	centroid_1690	100.31467	1.30E-23	centroid_1690	4.26E-09
centroid_17640	conserved hypothetical protein	0	120	5	0	centroid_1764	100.31467	1.30E-23	centroid_1764	4.26E-09
centroid_1885	conserved hypothetical protein	0	120	5	0	centroid_1885	100.31467	1.30E-23	centroid_1885	4.26E-09
centroid_2767	conserved hypothetical protein	0	120	5	0	centroid_2767	100.31467	1.30E-23	centroid_2767	4.26E-09
centroid_3536	conserved hypothetical protein	0	120	5	0	centroid_3536	100.31467	1.30E-23	centroid_3536	4.26E-09
centroid_3537	2,3-diketo-L-gulonate reductase	0	120	5	0	centroid_3537	100.31467	1.30E-23	centroid_3537	4.26E-09
centroid_3538	HTH-type transcriptional regulator YiaJ	0	120	5	0	centroid_3538	100.31467	1.30E-23	centroid_3538	4.26E-09
centroid_3815	TRAP transporter solute receptor, DctP family protein	0	120	5	0	centroid_3815	100.31467	1.30E-23	centroid_3815	4.26E-09
centroid_3816	3-keto-L-gulonate-6-phosphate decarboxylase SgbH	0	120	5	0	centroid_3816	100.31467	1.30E-23	centroid_3816	4.26E-09
centroid_3817	xylose isomerase-like TIM barrel family protein	0	120	5	0	centroid_3817	100.31467	1.30E-23	centroid_3817	4.26E-09
centroid_6935	hypothetical protein	0	120	5	0	centroid_6935	100.31467	1.30E-23	centroid_6935	4.26E-09
centroid_7184	papC N-terminal domain protein	0	120	5	0	centroid_7184	100.31467	1.30E-23	centroid_7184	4.26E-09
centroid_7185	type VII secretion system (T7SS), usher family protein	0	120	5	0	centroid_7185	100.31467	1.30E-23	centroid_7185	4.26E-09
centroid_77	conserved hypothetical protein	0	120	5	0	centroid_77	100.31467	1.30E-23	centroid_77	4.26E-09
centroid_9912	type VII secretion system (T7SS), usher family protein	0	120	5	0	centroid_9912	100.31467	1.30E-23	centroid_9912	4.26E-09
centroid_9913	type VII secretion system (T7SS), usher family protein	0	120	5	0	centroid_9913	100.31467	1.30E-23	centroid_9913	4.26E-09
centroid_11173	carbohydrate kinase, FGGY family	0	119	5	1	centroid_1117	82.7370011	9.37E-20	centroid_1117	2.56E-08
centroid_17637	L-xylulose/3-keto-L-gulonate kinase	0	119	5	1	centroid_1763	82.7370011	9.37E-20	centroid_1763	2.56E-08
centroid_18274	gram-negative porin family protein	0	119	5	1	centroid_1827	82.7370011	9.37E-20	centroid_1827	2.56E-08
centroid_3818	L-ribulose-5-phosphate 4-epimerase	0	119	5	1	centroid_3818	82.7370011	9.37E-20	centroid_3818	2.56E-08
centroid_8454	fimbrial family protein	0	119	5	1	centroid_8454	82.7370011	9.37E-20	centroid_8454	2.56E-08
centroid_14943	inner membrane YcfZ domain protein	0	118	5	2	centroid_1494	70.1816611	5.41E-17	centroid_1494	8.95E-08
centroid_15256	conserved hypothetical protein	0	118	5	2	centroid_1525	70.1816611	5.41E-17	centroid_1525	8.95E-08
centroid_17545	conserved hypothetical protein	0	118	5	2	centroid_1754	70.1816611	5.41E-17	centroid_1754	8.95E-08

centroid_1782	inner membrane protein YcfZ	0	118	5	2	centroid_1782	70.1816611	5.41E-17	centroid_1782	8.95E-08
centroid_4537	inner membrane protein YcfZ	0	118	5	2	centroid_4537	70.1816611	5.41E-17	centroid_4537	8.95E-08
centroid_5240	conserved hypothetical protein	0	118	5	2	centroid_5240	70.1816611	5.41E-17	centroid_5240	8.95E-08
centroid_5924	conserved hypothetical protein	0	117	5	3	centroid_5924	60.76528	6.43E-15	centroid_5924	2.39E-07
centroid_13071	conserved hypothetical protein	0	116	5	4	centroid_1307	53.4415409	2.66E-13	centroid_1307	5.37E-07
centroid_16855	ia polymorphic membrane (Chlamydia_PMP) repeat famil	0	116	5	4	centroid_1685	53.4415409	2.66E-13	centroid_1685	5.37E-07
centroid_16930	phage late control gene D family protein	0	116	5	4	centroid_1693	53.4415409	2.66E-13	centroid_1693	5.37E-07
centroid_7097	conserved hypothetical protein	0	116	5	4	centroid_7097	53.4415409	2.66E-13	centroid_7097	5.37E-07
centroid_2768	putative type-1 fimbrial protein, A chain	0	115	5	5	centroid_2768	47.582654	5.27E-12	centroid_2768	1.07E-06
centroid_13866	type VII secretion system (T7SS), usher family protein	0	114	5	6	centroid_1386	42.7891165	6.10E-11	centroid_1386	1.97E-06
centroid_2145	tonB-dependent vitamin B12 receptor	0	114	5	6	centroid_2145	42.7891165	6.10E-11	centroid_2145	1.97E-06
centroid_6916	conserved hypothetical protein	0	114	5	6	centroid_6916	42.7891165	6.10E-11	centroid_6916	1.97E-06
centroid_14039	class II Aldolase and Adducin N-terminal domain protein	0	113	5	7	centroid_1403	38.7945935	4.71E-10	centroid_1403	3.38E-06
centroid_4431	conserved hypothetical protein	0	113	5	7	centroid_4431	38.7945935	4.71E-10	centroid_4431	3.38E-06
centroid_1247	conserved hypothetical protein	0	109	5	11	centroid_1247	27.8103796	1.34E-07	centroid_1247	1.86E-05
centroid_13565	putative colanic acid biosynthesis domain protein	0	109	5	11	centroid_1356	27.8103796	1.34E-07	centroid_1356	1.86E-05
centroid_14852	-independent periplasmic transporters, DctQ component	0	105	5	15	centroid_1485	21.2208581	4.09E-06	centroid_1485	6.61E-05
centroid_17471	conserved hypothetical protein	0	105	5	15	centroid_1747	21.2208581	4.09E-06	centroid_1747	6.61E-05
centroid_2637	conserved hypothetical protein	0	105	5	15	centroid_2637	21.2208581	4.09E-06	centroid_2637	6.61E-05
centroid_2747	antitoxin MqsA	0	105	5	15	centroid_2747	21.2208581	4.09E-06	centroid_2747	6.61E-05
centroid_4650	2,3-diketo-L-gulonate-binding periplasmic protein YiaO	0	105	5	15	centroid_4650	21.2208581	4.09E-06	centroid_4650	6.61E-05
centroid_4651	TRAP transporter, DctM subunit	0	105	5	15	centroid_4651	21.2208581	4.09E-06	centroid_4651	6.61E-05
centroid_4652	keto-L-gulonate TRAP transporter small permease protei	0	105	5	15	centroid_4652	21.2208581	4.09E-06	centroid_4652	6.61E-05
centroid_4653	conserved hypothetical protein	0	104	5	16	centroid_4653	19.965874	7.88E-06	centroid_4653	8.68E-05
centroid_17467	type II secretion system protein L	0	102	5	18	centroid_1746	17.7834834	2.48E-05	centroid_1746	0.00014347
centroid_3084	BFD-like [2Fe-2S] binding domain protein	0	102	5	18	centroid_3084	17.7834834	2.48E-05	centroid_3084	0.00014347
centroid_3085	bacterioferritin	0	102	5	18	centroid_3085	17.7834834	2.48E-05	centroid_3085	0.00014347
centroid_3086	e 4 prepilin-like proteins leader peptide-processing enzy	0	102	5	18	centroid_3086	17.7834834	2.48E-05	centroid_3086	0.00014347
centroid_3087	type II secretion system (T2SS), M family protein	0	102	5	18	centroid_3087	17.7834834	2.48E-05	centroid_3087	0.00014347
centroid_3098	AAA domain protein	0	102	5	18	centroid_3098	17.7834834	2.48E-05	centroid_3098	0.00014347
centroid_3099	putative peptidoglycan binding domain protein	0	102	5	18	centroid_3099	17.7834834	2.48E-05	centroid_3099	0.00014347
centroid_3100	lcium-binding protein required for initiation of chromosome	0	102	5	18	centroid_3100	17.7834834	2.48E-05	centroid_3100	0.00014347
centroid_3700	UDP-glucose 6-dehydrogenase	0	102	5	18	centroid_3700	17.7834834	2.48E-05	centroid_3700	0.00014347
centroid_5500	gspL periplasmic domain protein	0	102	5	18	centroid_5500	17.7834834	2.48E-05	centroid_5500	0.00014347
centroid_8581	putative general secretion pathway protein A	0	102	5	18	centroid_8581	17.7834834	2.48E-05	centroid_8581	0.00014347
centroid_10869	type II secretion system protein H	0	101	5	19	centroid_1086	16.8287825	4.09E-05	centroid_1086	0.00018123
centroid_12074	type II secretion system protein H	0	101	5	19	centroid_1207	16.8287825	4.09E-05	centroid_1207	0.00018123
centroid_17465	type II secretion system (T2SS), F family protein	0	101	5	19	centroid_1746	16.8287825	4.09E-05	centroid_1746	0.00018123
centroid_17466	type II secretion system (T2SS), F family protein	0	101	5	19	centroid_1746	16.8287825	4.09E-05	centroid_1746	0.00018123
centroid_3083	putative bifunctional chitinase/lysozyme	0	101	5	19	centroid_3083	16.8287825	4.09E-05	centroid_3083	0.00018123
centroid_3089	type II secretion system (T2SS), K family protein	0	101	5	19	centroid_3089	16.8287825	4.09E-05	centroid_3089	0.00018123
centroid_3090	repilin-type N-terminal cleavage/methylation domain prote	0	101	5	19	centroid_3090	16.8287825	4.09E-05	centroid_3090	0.00018123
centroid_3091	type II secretion system protein I	0	101	5	19	centroid_3091	16.8287825	4.09E-05	centroid_3091	0.00018123
centroid_3092	type II secretion system protein H	0	101	5	19	centroid_3092	16.8287825	4.09E-05	centroid_3092	0.00018123
centroid_3093	type II secretion system protein G	0	101	5	19	centroid_3093	16.8287825	4.09E-05	centroid_3093	0.00018123
centroid_3094	type II secretion system protein F	0	101	5	19	centroid_3094	16.8287825	4.09E-05	centroid_3094	0.00018123
centroid_3095	type II secretion system protein E	0	101	5	19	centroid_3095	16.8287825	4.09E-05	centroid_3095	0.00018123
centroid_4742	repilin-type N-terminal cleavage/methylation domain prote	0	101	5	19	centroid_4742	16.8287825	4.09E-05	centroid_4742	0.00018123
centroid_5294	shET2 enterotoxin, N-terminal region family protein	0	101	5	19	centroid_5294	16.8287825	4.09E-05	centroid_5294	0.00018123
centroid_6220	shET2 enterotoxin, N-terminal region family protein	0	101	5	19	centroid_6220	16.8287825	4.09E-05	centroid_6220	0.00018123
centroid_7722	type II secretion system protein F	0	101	5	19	centroid_7722	16.8287825	4.09E-05	centroid_7722	0.00018123
centroid_7723	putative type II secretion system F domain protein	0	101	5	19	centroid_7723	16.8287825	4.09E-05	centroid_7723	0.00018123
centroid_8069	type II secretion system protein D	0	101	5	19	centroid_8069	16.8287825	4.09E-05	centroid_8069	0.00018123
centroid_8070	type II secretion system protein L	0	101	5	19	centroid_8070	16.8287825	4.09E-05	centroid_8070	0.00018123
centroid_8580	carbohydrate binding domain protein	0	101	5	19	centroid_8580	16.8287825	4.09E-05	centroid_8580	0.00018123
centroid_4096	heat-stable enterotoxin A3/A4	0	100	5	20	centroid_4096	15.9505208	6.50E-05	centroid_4096	0.00022654
centroid_7923	heat-stable enterotoxin A3/A4	0	100	5	20	centroid_7923	15.9505208	6.50E-05	centroid_7923	0.00022654
centroid_13907	conserved hypothetical protein	0	99	5	21	centroid_1390	15.1398804	9.98E-05	centroid_1390	0.00028047
centroid_1481	putative lipoprotein	0	99	5	21	centroid_1481	15.1398804	9.98E-05	centroid_1481	0.00028047
centroid_1482	conserved hypothetical protein	0	99	5	21	centroid_1482	15.1398804	9.98E-05	centroid_1482	0.00028047
centroid_3096	type II secretion system protein D	0	99	5	21	centroid_3096	15.1398804	9.98E-05	centroid_3096	0.00028047
centroid_7749	hypothetical protein	0	99	5	21	centroid_7749	15.1398804	9.98E-05	centroid_7749	0.00028047
centroid_8068	type II secretion system D domain protein	0	99	5	21	centroid_8068	15.1398804	9.98E-05	centroid_8068	0.00028047
centroid_8471	conserved hypothetical protein	0	99	5	21	centroid_8471	15.1398804	9.98E-05	centroid_8471	0.00028047
