# Supplementary material for: Comparative genomic analysis and molecular examination of the diversity of enterotoxigenic Escherichia coli isolates from Chile
Source: PLoS Negl Trop Dis. 2019 Nov 20;13(11):e0007828. doi: 10.1371/journal.pntd.0007828 (PMC6901236; doi:10.1371/journal.pntd.0007828)
Supplement: S2 Table — (PDF) [file pntd.0007828.s003.pdf]

Table S2: Reference genomes and corresponding pathotypes

Isolate	Pathotype	Phylogroup	GenBank Accession
BL21	lab adapted	A	NC_012947.1
BW2952	lab adapted	A	NC_012759.1
SE11	fecal isolate	B1	NC_011415.1
IAI1	fecal isolate	B1	NC_011741.1
SMS_3_5	other	F	CP000970.1
HS	commensal	A	NC_009800.1
ATCC 8739	lab adapted	A	NC_010468.1
536	ExPEC	B2	NC_008253.1
S88	ExPEC	B2	NC_011742.1
UTI89	ExPEC	B2	NC_007946.1
CFT073	ExPEC	B2	AE014075.1
IA139	ExPEC	F	NC_011750.1
UMN026	ExPEC	D	NC_011751.1
042	EAEC	D	FN554766.1
53638	EIEC	A	AAKB00000000.2
55989	EAEC	B1	NC_011748.1
TY-2482	EAEC/STEC	B1	AFOG00000000.1
S. flexneri 2a 2457T	Shigella	B1	NC_004741.1
S. boydii 3083-94	Shigella	B1	NC_010658.1
S. sonnei 046	Shigella	B1	NC_007384.1
S. dysenteriae 197	Shigella	E	NC_007606.1
EDL933	EHEC	E	NC_002655.2
Sakai	EHEC	E	NC_002695.1
CB9615	EPEC	E	NC_013941.1
11368	EHEC	B1	NC_013361.1
11128	EHEC	B1	NC_013364.1
32/73	EPEC	B1	LAFA00000000
702324	EPEC	B2	JHRR00000000
B171	EPEC	B1	AAJX00000000.2
E110019	AEEC	B1	AAJW00000000.2
E2348/69	EPEC	B2	NC_011601.1
ETEC References			
TW11681	ETEC	A	AELD00000000
H10407	ETEC	A	FN649414.1
TW10598	ETEC	A	AELA00000000
UMNK88	ETEC	A	NC_017641.1
2846750	ETEC	A	AQGG00000000
MP021566.1	ETEC	A	AQEU00000000
BCE019_MS-13	ETEC	A	AQCZ00000000
B7A	ETEC	B1	AAJT00000000.2
TW14425	ETEC	B1	AELE00000000
E24377A	ETEC	B1	NC_009801.1
TW10828	ETEC	B1	AELC00000000
Jurua 18/11	ETEC	B1	AQFB00000000

ThroopD	ETEC	B1	AQEJ00000000
2726800	ETEC	B1	AQFE00000000
2854350	ETEC	B1	APZL00000000
BCE002_MS-12	ETEC	B1	AQDA00000000
Envira 8/11	ETEC	B1	AQFC00000000
2866350	ETEC	B1	APXJ00000000
BCE034_MS-14	ETEC	B1	AQCY00000000
2851500	ETEC	B1	AQDN00000000
2866450	ETEC	B1	AQDI00000000
2845650	ETEC	E	AQDR00000000
ETEC Lineage References			
E8_ETEC_L1	ETEC_lineage 1	A	ERS038927
E632_ETEC_L1	ETEC_lineage 1	A	ERS038942
E66_ETEC_L2	ETEC_lineage 2	A	ERS044460
E822_ETEC_L2	ETEC_lineage 2	A	ERS044484
E36_ETEC_L3	ETEC_lineage 3	B1	ERS044458
E810_ETEC_L3	ETEC_lineage 3	B1	ERS044481
E1115_ETEC_L4	ETEC_lineage 4	A	ERS206737
E1365_ETEC_L4	ETEC_lineage 4	A	ERS077686
E157_ETEC_L5	ETEC_lineage 5	B1	ERS077703
E21_ETEC_L5	ETEC_lineage 5	B1	ERS044456
E636_ETEC_L6	ETEC_lineage 6	A	ERS038943
E897_ETEC_L6	ETEC_lineage 6	A	ERS038948
E1484_ETEC_L7	ETEC_lineage 7	E	ERS206752
E370_ETEC_L7	ETEC_lineage 7	E	ERS038935
E2108_ETEC_L8	ETEC_lineage 8	B1	ERS077758
E224_ETEC_L8	ETEC_lineage 8	B1	ERS044476
E856_ETEC_L9	ETEC_lineage 9	A	ERS044486
E943_ETEC_L9	ETEC_lineage 9	A	ERS044499
E1285_ETEC_L10	ETEC_lineage 10	A	ERS077680
E945_ETEC_L10	ETEC_lineage 10	A	ERS077657
E167_ETEC_L11	ETEC_lineage 11	A	ERS077743
E1057_ETEC_L11	ETEC_lineage 11	A	ERS077667
E330_ETEC_L12	ETEC_lineage 12	A	ERS077581
E1556_ETEC_L12	ETEC_lineage 12	A	ERS077700
E628_ETEC_L13	ETEC_lineage 13	A	ERS077614
E1525_ETEC_L13	ETEC_lineage 13	A	ERS077692
E1091_ETEC_L15	ETEC_lineage 15	A	ERS038960
E2377_ETEC_L15	ETEC_lineage 15	A	ERS077765
E85_ETEC_L16	ETEC_lineage 16	A	ERS044463
E816_ETEC_L16	ETEC_lineage 16	A	ERS044483
E333_ETEC_L17	ETEC_lineage 17	B1	ERS038933
E2404_ETEC_L17	ETEC_lineage 17	B1	ERS077770
E659_ETEC_L18	ETEC_lineage 18	B1	ERS077617
E5085_ETEC_L18	ETEC_lineage 18	B1	ERS055670
E907_ETEC_L19	ETEC_lineage 19	B1	ERS044496

E920_ETEC_L19	ETEC_lineage 19	B1	ERS044497
E873_ETEC_L20	ETEC_lineage 20	B1	ERS077633
E2395_ETEC_L20	ETEC_lineage 20	B1	ERS077769
E1564_ETEC_L21	ETEC_lineage 21	A	ERS077702
E2367_ETEC_L21	ETEC_lineage 21	A	ERS077762
