# Supplementary material for: Comparative genomic analysis and molecular examination of the diversity of enterotoxigenic Escherichia coli isolates from Chile
Source: PLoS Negl Trop Dis. 2019 Nov 20;13(11):e0007828. doi: 10.1371/journal.pntd.0007828 (PMC6901236; doi:10.1371/journal.pntd.0007828)
Supplement: S3 Table — (PDF) [file pntd.0007828.s004.pdf]

Table S3. O and H antigen prevalence in Chile ETEC isolates

O-antigen			H-antigen			O and H antigen		
O-antigen	number	percent	H-antigen	number	Percent	O and H serotype	number	Percent
O6	39	31.2	H16	39	31.2	O6:H16	25	20
O128ab/ac	20	16	H45	28	22.4	O128ab/ac:H45	19	15.2
ONT	16	12.8	H-	14	11.2	O6:H-	14	11.2
O25	11	8.8	H9	7	5.6	O25:H16	10	8
O80	4	3.2	H21	7	5.6	ONT:H45	8	6.4
O148	3	2.4	H10	5	4	ONT:H21	5	4
O169	3	2.4	H12	5	4	O80:H9	4	3.2
O23	3	2.4	H28	3	2.4	O148:H28	3	2.4
O27	3	2.4	H4	2	1.6	O23:H16	3	2.4
O49	3	2.4	H20	2	1.6	O49:H12	3	2.4
O78	3	2.4	H25	2	1.6	O8:H9	3	2.4
O8	3	2.4	H32	2	1.6	ND	2	1.6
ND	2	1.6	ND	2	1.6	O159:H4	2	1.6
O15	2	1.6	H2	1	0.8	O169:H25	2	1.6
O159	2	1.6	H5	1	0.8	O27:H20	2	1.6
O21	2	1.6	H11	1	0.8	O78:H10	2	1.6
O114	1	0.8	H40	1	0.8	O16:H48	1	0.8
O115	1	0.8	H41	1	0.8	O114:H10	1	0.8
O16	1	0.8	H48	1	0.8	O115:H40	1	0.8
O4	1	0.8	H51	1	0.8	O128ab:H21	1	0.8
O64	1	0.8				O15:H11	1	0.8
O97	1	0.8				O15:H12	1	0.8
						O169:H41	1	0.8
						O21:H12	1	0.8
						O21:H21	1	0.8
						O25:H51	1	0.8
						O27:H10	1	0.8
						O4:H16	1	0.8
						O64:H5	1	0.8
						O78:H32	1	0.8
						O97:H45	1	0.8
						ONT:H10	1	0.8
						ONT:H2	1	0.8
						ONT:H32	1	0.8
