# Supplementary material for: Comparative genomic analysis and molecular examination of the diversity of enterotoxigenic Escherichia coli isolates from Chile
Source: PLoS Negl Trop Dis. 2019 Nov 20;13(11):e0007828. doi: 10.1371/journal.pntd.0007828 (PMC6901236; doi:10.1371/journal.pntd.0007828)
Supplement: S4 Table — (PDF) [file pntd.0007828.s005.pdf]

Table S4. Distribution by Geography - Santiago

Gene_ID	Annotation	antiago_prese	Other_presen	antiago_Abse	Other_Absent	Gene_ID	chisq-stats	pvalues	Gene_ID	pvalues
centroid_144	gram-negative pili assembly chaperone, N-terminal domain protein	88	23	0	14	centroid_144	33.788978	6.14E-09	centroid_144	4.99E-09
centroid_15982	glutamate decarboxylase	88	23	0	14	centroid_15982	33.788978	6.14E-09	centroid_15982	4.99E-09
centroid_17423	gram-negative pili assembly chaperone, N-terminal domain protein	88	23	0	14	centroid_17423	33.788978	6.14E-09	centroid_17423	4.99E-09
centroid_145	type VII secretion system (T7SS), usher family protein	88	24	0	13	centroid_145	30.840207	2.80E-08	centroid_145	2.33E-08
centroid_7651	type VII secretion system (T7SS), usher family protein	88	24	0	13	centroid_7651	30.840207	2.80E-08	centroid_7651	2.33E-08
centroid_9887	hypothetical protein	88	26	0	11	centroid_9887	25.101807	5.44E-07	centroid_9887	4.61E-07
centroid_12405	ABC transporter family protein	88	27	0	10	centroid_12405	22.310192	2.32E-06	centroid_12405	1.96E-06
centroid_1287	helix-turn-helix family protein	88	27	0	10	centroid_1287	22.310192	2.32E-06	centroid_1287	1.96E-06
centroid_1288	outer membrane protein G	88	27	0	10	centroid_1288	22.310192	2.32E-06	centroid_1288	1.96E-06
centroid_1289	ABC transporter family protein	88	27	0	10	centroid_1289	22.310192	2.32E-06	centroid_1289	1.96E-06
centroid_1290	beta-phosphoglucomutase	88	27	0	10	centroid_1290	22.310192	2.32E-06	centroid_1290	1.96E-06
centroid_13072	beta-phosphoglucomutase	88	27	0	10	centroid_13072	22.310192	2.32E-06	centroid_13072	1.96E-06
centroid_17428	haloacid dehalogenase-like hydrolase family protein	88	27	0	10	centroid_17428	22.310192	2.32E-06	centroid_17428	1.96E-06
centroid_1916	conserved hypothetical protein	88	27	0	10	centroid_1916	22.310192	2.32E-06	centroid_1916	1.96E-06
centroid_687	transcriptional activator CadC	88	27	0	10	centroid_687	22.310192	2.32E-06	centroid_687	1.96E-06
centroid_692	conserved hypothetical protein	88	27	0	10	centroid_692	22.310192	2.32E-06	centroid_692	1.96E-06
centroid_8199	ABC transporter family protein	88	27	0	10	centroid_8199	22.310192	2.32E-06	centroid_8199	1.96E-06
centroid_11295	fimbrial family protein	88	28	0	9	centroid_11295	19.569319	9.70E-06	centroid_11295	8.14E-06
centroid_12429	phosphate transporter family protein	88	28	0	9	centroid_12429	19.569319	9.70E-06	centroid_12429	8.14E-06
centroid_12430	phosphate transporter family protein	88	28	0	9	centroid_12430	19.569319	9.70E-06	centroid_12430	8.14E-06
centroid_1277	EAL domain protein	88	28	0	9	centroid_1277	19.569319	9.70E-06	centroid_1277	8.14E-06
centroid_146	fimbrial family protein	88	28	0	9	centroid_146	19.569319	9.70E-06	centroid_146	8.14E-06
centroid_17105	type VII secretion system (T7SS), usher family protein	88	28	0	9	centroid_17105	19.569319	9.70E-06	centroid_17105	8.14E-06
centroid_17641	conserved hypothetical protein	88	28	0	9	centroid_17641	19.569319	9.70E-06	centroid_17641	8.14E-06
centroid_2664	conserved hypothetical protein	88	28	0	9	centroid_2664	19.569319	9.70E-06	centroid_2664	8.14E-06
centroid_2717	phosphate transporter family protein	88	28	0	9	centroid_2717	19.569319	9.70E-06	centroid_2717	8.14E-06
centroid_2989	PTS system, cellobiose-specific IIC component	88	28	0	9	centroid_2989	19.569319	9.70E-06	centroid_2989	8.14E-06
centroid_2990	-diacetylchitobiose-specific phosphotransferase enzyme IIB compo	88	28	0	9	centroid_2990	19.569319	9.70E-06	centroid_2990	8.14E-06
centroid_3080	oxygen sensor protein DosP	88	28	0	9	centroid_3080	19.569319	9.70E-06	centroid_3080	8.14E-06
centroid_3180	inner membrane transport protein YeaN	88	28	0	9	centroid_3180	19.569319	9.70E-06	centroid_3180	8.14E-06
centroid_4400	glycine zipper 2TM domain protein	88	28	0	9	centroid_4400	19.569319	9.70E-06	centroid_4400	8.14E-06
centroid_4457	conserved hypothetical protein	88	28	0	9	centroid_4457	19.569319	9.70E-06	centroid_4457	8.14E-06
centroid_4668	haemolysin E family protein	88	28	0	9	centroid_4668	19.569319	9.70E-06	centroid_4668	8.14E-06
centroid_492	taurine ABC transporter, periplasmic binding protein	88	28	0	9	centroid_492	19.569319	9.70E-06	centroid_492	8.14E-06
centroid_5570	haemolysin E family protein	88	28	0	9	centroid_5570	19.569319	9.70E-06	centroid_5570	8.14E-06
centroid_602	glycine zipper 2TM domain protein	88	28	0	9	centroid_602	19.569319	9.70E-06	centroid_602	8.14E-06
centroid_688	transporter, basic amino acid/polyamine antiporter family protein	88	28	0	9	centroid_688	19.569319	9.70E-06	centroid_688	8.14E-06
centroid_689	lysine decarboxylase, inducible	88	28	0	9	centroid_689	19.569319	9.70E-06	centroid_689	8.14E-06
centroid_690	H+ symporter) family protein	88	28	0	9	centroid_690	19.569319	9.70E-06	centroid_690	8.14E-06
centroid_691	lysine--tRNA ligase	88	28	0	9	centroid_691	19.569319	9.70E-06	centroid_691	8.14E-06
centroid_693	conserved hypothetical protein	88	28	0	9	centroid_693	19.569319	9.70E-06	centroid_693	8.14E-06
centroid_7650	type VII secretion system (T7SS), usher family protein	88	28	0	9	centroid_7650	19.569319	9.70E-06	centroid_7650	8.14E-06
centroid_8096	conserved hypothetical protein	88	28	0	9	centroid_8096	19.569319	9.70E-06	centroid_8096	8.14E-06
centroid_8220	major Facilitator Superfamily protein	88	28	0	9	centroid_8220	19.569319	9.70E-06	centroid_8220	8.14E-06
centroid_8446	taurine ABC transporter, periplasmic binding protein	88	28	0	9	centroid_8446	19.569319	9.70E-06	centroid_8446	8.14E-06
centroid_8926	putative pTS system, cellobiose-specific, IIC component	88	28	0	9	centroid_8926	19.569319	9.70E-06	centroid_8926	8.14E-06
centroid_893	fimbrial assembly family protein	88	28	0	9	centroid_893	19.569319	9.70E-06	centroid_893	8.14E-06
centroid_9097	major Facilitator Superfamily protein	88	28	0	9	centroid_9097	19.569319	9.70E-06	centroid_9097	8.14E-06
centroid_9909	fimbrial family protein	88	28	0	9	centroid_9909	19.569319	9.70E-06	centroid_9909	8.14E-06
centroid_9910	conserved hypothetical protein	88	28	0	9	centroid_9910	19.569319	9.70E-06	centroid_9910	8.14E-06
centroid_3011	CTP pyrophosphohydrolase	88	29	0	8	centroid_3011	16.878859	3.98E-05	centroid_3011	3.28E-05
centroid_7219	conserved hypothetical protein	87	24	1	13	centroid_7219	26.952031	2.09E-07	centroid_7219	2.61E-07
centroid_2311	bacterial regulatory, gntR family protein	87	26	1	11	centroid_2311	21.355295	3.82E-06	centroid_2311	4.38E-06
centroid_2312	D binding domain of 6-phosphogluconate dehydrogenase family pro	87	26	1	11	centroid_2312	21.355295	3.82E-06	centroid_2312	4.38E-06
centroid_2313	conserved hypothetical protein	87	26	1	11	centroid_2313	21.355295	3.82E-06	centroid_2313	4.38E-06
centroid_17045	conserved hypothetical protein	87	27	1	10	centroid_17045	18.649788	1.57E-05	centroid_17045	1.70E-05
centroid_4351	conserved hypothetical protein	87	27	1	10	centroid_4351	18.649788	1.57E-05	centroid_4351	1.70E-05
centroid_13909	EAL domain protein	87	28	1	9	centroid_13909	16.009115	6.30E-05	centroid_13909	6.37E-05
centroid_13964	sensors of blue-light using FAD family protein	87	28	1	9	centroid_13964	16.009115	6.30E-05	centroid_13964	6.37E-05
centroid_14633	conserved hypothetical protein	87	28	1	9	centroid_14633	16.009115	6.30E-05	centroid_14633	6.37E-05
centroid_147	fimbrial family protein	87	28	1	9	centroid_147	16.009115	6.30E-05	centroid_147	6.37E-05
centroid_16436	major Facilitator Superfamily protein	87	28	1	9	centroid_16436	16.009115	6.30E-05	centroid_16436	6.37E-05
centroid_17685	conserved hypothetical protein	87	28	1	9	centroid_17685	16.009115	6.30E-05	centroid_17685	6.37E-05
centroid_1915	HTH-type transcriptional repressor YcgE	87	28	1	9	centroid_1915	16.009115	6.30E-05	centroid_1915	6.37E-05
centroid_2521	inner membrane transport protein YdiM	87	28	1	9	centroid_2521	16.009115	6.30E-05	centroid_2521	6.37E-05
centroid_2715	molybdopterin guanine dinucleotide synthesis B family protein	87	28	1	9	centroid_2715	16.009115	6.30E-05	centroid_2715	6.37E-05
centroid_2716	conserved hypothetical protein	87	28	1	9	centroid_2716	16.009115	6.30E-05	centroid_2716	6.37E-05
centroid_3182	putative diguanylate cyclase YeaP	87	28	1	9	centroid_3182	16.009115	6.30E-05	centroid_3182	6.37E-05
centroid_13336	shikimate transporter domain protein	86	25	2	12	centroid_13336	20.887096	4.87E-06	centroid_13336	6.05E-06
centroid_5486	putative aminopeptidase	86	25	2	12	centroid_5486	20.887096	4.87E-06	centroid_5486	6.05E-06
centroid_9791	papC C-terminal domain protein	86	27	2	10	centroid_9791	15.650488	7.62E-05	centroid_9791	8.01E-05
centroid_10606	helix-turn-helix domain protein	85	18	3	19	centroid_10606	38.04339	6.92E-10	centroid_10606	1.18E-09
centroid_10599	putative predicted inner membrane protein	85	24	3	13	centroid_10599	20.733385	5.28E-06	centroid_10599	6.66E-06
centroid_17355	conserved hypothetical protein	85	24	3	13	centroid_17355	20.733385	5.28E-06	centroid_17355	6.66E-06
centroid_3651	conserved hypothetical protein	85	24	3	13	centroid_3651	20.733385	5.28E-06	centroid_3651	6.66E-06
centroid_5438	conserved hypothetical protein	85	24	3	13	centroid_5438	20.733385	5.28E-06	centroid_5438	6.66E-06
centroid_6147	conserved hypothetical protein	85	26	3	11	centroid_6147	15.594175	7.85E-05	centroid_6147	8.26E-05
centroid_10357	type VI secretion system effector, Hcp1 family protein	84	24	4	13	centroid_10357	18.221393	1.97E-05	centroid_10357	2.26E-05
centroid_3649	conserved hypothetical protein	84	24	4	13	centroid_3649	18.221393	1.97E-05	centroid_3649	2.26E-05
centroid_3650	putative inner membrane protein yafU	84	24	4	13	centroid_3650	18.221393	1.97E-05	centroid_3650	2.26E-05
centroid_9099	dependent phosphotransferase enzyme II for cellobiose domain pr	83	19	5	18	centroid_9099	29.230452	6.43E-08	centroid_9099	9.71E-08
centroid_12699	putative transposase	83	21	5	16	centroid_12699	23.673544	1.14E-06	centroid_12699	1.52E-06
centroid_3648	type VI secretion system effector, Hcp1 family protein	83	23	5	14	centroid_3648	18.475559	1.72E-05	centroid_3648	1.94E-05
centroid_16558	type VI secretion system effector, Hcp1 family protein	83	24	5	13	centroid_16558	16.020273	6.27E-05	centroid_16558	6.46E-05
centroid_4905	putative inner membrane protein	83	24	5	13	centroid_4905	16.020273	6.27E-05	centroid_4905	6.46E-05
centroid_12314	transposase, IS605 OrfB family	82	16	6	21	centroid_12314	35.467579	2.59E-09	centroid_12314	4.01E-09
centroid_9182	transglycosylase SLT domain protein	82	22	6	15	centroid_9182	18.848344	1.42E-05	centroid_9182	1.57E-05
centroid_12686	transposase, IS605 OrfB family	81	16	7	21	centroid_12686	32.937412	9.52E-09	centroid_12686	1.36E-08
centroid_4208	transposase, IS605 OrfB family	81	19	7	18	centroid_4208	24.476447	7.52E-07	centroid_4208	9.41E-07
centroid_8226	transposase, IS605 OrfB family	81	19	7	18	centroid_8226	24.476447	7.52E-07	centroid_8226	9.41E-07
centroid_10607	transposase, IS605 OrfB family	80	15	8	22	centroid_10607	33.521195	7.05E-09	centroid_10607	9.35E-09
centroid_3998	prophage CP4-57 regulatory family protein	80	16	8	21	centroid_3998	30.59408	3.18E-08	centroid_3998	4.15E-08
centroid_10758	conserved hypothetical protein	80	21	8	16	centroid_10758	17.444474	2.96E-05	centroid_10758	2.92E-05
centroid_12941	conserved hypothetical protein	80	21	8	16	centroid_12941	17.444474	2.96E-05	centroid_12941	2.92E-05
centroid_2389	conserved hypothetical protein	80	21	8	16	centroid_2389	17.444474	2.96E-05	centroid_2389	2.92E-05
centroid_5185	conserved hypothetical protein	80	21	8	16	centroid_5185	17.444474	2.96E-05	centroid_5185	2.92E-05
centroid_11498	conserved hypothetical protein	79	13	9	24	centroid_11498	37.257307	1.04E-09	centroid_11498	1.24E-09
centroid_2391	conserved hypothetical protein	79	21	9	16	centroid_2391	15.742571	7.26E-05	centroid_2391	0.0001117
centroid_8205	type VII secretion system (T7SS), usher family protein	79	21	9	16	centroid_8205	15.742571	7.26E-05	centroid_8205	0.0001117
centroid_13621	transglycosylase SLT domain protein	78	20	10	17	centroid_13621	16.410083	5.10E-05	centroid_13621	6.36E-05
centroid_13091	mRNA interferase MqsR	77	19	11	18	centroid_13091	17.12839	3.49E-05	centroid_13091	3.76E-05
centroid_2748	mRNA interferase MqsR	77	19	11	18	centroid_2748	17.12839	3.49E-05	centroid_2748	3.76E-05
centroid_8297	transglycosylase SLT domain protein	76	19	12	18	centroid_8297	15.639228	7.66E-05	centroid_8297	6.65E-05
centroid_8076	glutamate decarboxylase	75	12	13	25	centroid_8076	31.864393	1.65E-08	centroid_8076	1.41E-08

centroid_17627	fimbrial subunit ElfA	75	15	13	22	centroid_17627	23.632272	1.17E-06	centroid_17627	1.37E-06
centroid_11069	conserved hypothetical protein	75	17	13	20	centroid_11069	18.713201	1.52E-05	centroid_11069	1.36E-05
centroid_10219	phage tail sheath family protein	74	11	14	26	centroid_10219	32.920629	9.60E-09	centroid_10219	7.26E-09
centroid_18677	phage tail sheath family protein	74	12	14	25	centroid_18677	30.020952	4.27E-08	centroid_18677	3.42E-08
centroid_13199	phage late control gene D family protein	74	13	14	24	centroid_13199	27.236843	1.80E-07	centroid_13199	2.17E-07
centroid_3638	ogr/Delta-like zinc finger family protein	74	13	14	24	centroid_3638	27.236843	1.80E-07	centroid_3638	2.17E-07
centroid_10205	phage late control gene D family protein	74	14	14	23	centroid_10205	24.568282	7.17E-07	centroid_10205	7.14E-07
centroid_13559	putative phage tail tape measure domain protein	74	14	14	23	centroid_13559	24.568282	7.17E-07	centroid_13559	7.14E-07
centroid_3637	hok/gef family protein	74	15	14	22	centroid_3637	22.015682	2.70E-06	centroid_3637	2.43E-06
centroid_3640	phage tail sheath family protein	73	11	15	26	centroid_3640	31.106807	2.44E-08	centroid_3640	3.00E-08
centroid_16096	phage late control gene D family protein	73	13	15	24	centroid_16096	25.565506	4.28E-07	centroid_16096	3.70E-07
centroid_7906	phage late control gene D family protein	73	13	15	24	centroid_7906	25.565506	4.28E-07	centroid_7906	3.70E-07
centroid_10197	phage P2 GpU family protein	73	15	15	22	centroid_10197	20.497528	5.97E-06	centroid_10197	7.84E-06
centroid_11465	hypothetical protein	73	15	15	22	centroid_11465	20.497528	5.97E-06	centroid_11465	7.84E-06
centroid_16146	conserved hypothetical protein	73	15	15	22	centroid_16146	20.497528	5.97E-06	centroid_16146	7.84E-06
centroid_7095	phage P2 GpU family protein	73	15	15	22	centroid_7095	20.497528	5.97E-06	centroid_7095	7.84E-06
centroid_14165	conserved hypothetical protein	73	17	15	20	centroid_14165	15.908436	6.65E-05	centroid_14165	5.75E-05
centroid_5538	conserved hypothetical protein	73	17	15	20	centroid_5538	15.908436	6.65E-05	centroid_5538	5.75E-05
centroid_4244	conserved hypothetical protein	72	12	16	25	centroid_4244	26.625667	2.47E-07	centroid_4244	1.88E-07
centroid_10421	conserved predicted domain protein	72	13	16	24	centroid_10421	23.986331	9.70E-07	centroid_10421	7.31E-07
centroid_8326	putative conserved predicted protein	72	13	16	24	centroid_8326	23.986331	9.70E-07	centroid_8326	7.31E-07
centroid_3641	phage major tail tube protein	71	9	17	28	centroid_3641	33.503919	7.11E-09	centroid_3641	4.91E-09
centroid_13238	phage tail tube FII family protein	71	10	17	27	centroid_13238	30.565391	3.23E-08	centroid_13238	2.12E-08
centroid_3639	phage late control gene D family protein	71	11	17	26	centroid_3639	27.751161	1.38E-07	centroid_3639	9.22E-08
centroid_5227	conserved hypothetical protein	71	12	17	25	centroid_5227	25.06045	5.56E-07	centroid_5227	6.15E-07
centroid_3835	putative conserved predicted protein	71	13	17	24	centroid_3835	22.492874	2.11E-06	centroid_3835	1.87E-06
centroid_3643	phage P2 GpU family protein	71	15	17	22	centroid_3643	17.727699	2.55E-05	centroid_3643	1.89E-05
centroid_17429	conserved hypothetical protein	70	14	18	23	centroid_17429	18.708429	1.52E-05	centroid_17429	1.64E-05
centroid_3256	conserved hypothetical protein	70	15	18	22	centroid_3256	16.463455	4.96E-05	centroid_3256	4.31E-05
centroid_3257	conserved hypothetical protein	70	15	18	22	centroid_3257	16.463455	4.96E-05	centroid_3257	4.31E-05
centroid_3258	putative cPS-53 (KpLE1) prophage, predicted protein	70	15	18	22	centroid_3258	16.463455	4.96E-05	centroid_3258	4.31E-05
centroid_4195	plasmid stability family protein	70	15	18	22	centroid_4195	16.463455	4.96E-05	centroid_4195	4.31E-05
centroid_7397	putative yfdP	70	15	18	22	centroid_7397	16.463455	4.96E-05	centroid_7397	4.31E-05
centroid_7753	conserved hypothetical protein	70	15	18	22	centroid_7753	16.463455	4.96E-05	centroid_7753	4.31E-05
centroid_10247	hypothetical protein	69	15	19	22	centroid_10247	15.27233	9.31E-05	centroid_10247	6.56E-05
centroid_13239	phage tail tube FII family protein	69	15	19	22	centroid_13239	15.27233	9.31E-05	centroid_13239	6.56E-05
centroid_16143	hypothetical protein	69	15	19	22	centroid_16143	15.27233	9.31E-05	centroid_16143	6.56E-05
centroid_7843	phage tail tape measure protein, TP901 family, core region	68	13	20	24	centroid_7843	18.471362	1.72E-05	centroid_7843	1.19E-05
centroid_3834	terminase-like family protein	68	14	20	23	centroid_3834	16.245394	5.56E-05	centroid_3834	5.83E-05
centroid_7081	putative phage tail protein	68	14	20	23	centroid_7081	16.245394	5.56E-05	centroid_7081	5.83E-05
centroid_16147	baseplate J-like family protein	66	12	22	25	centroid_16147	18.343451	1.84E-05	centroid_16147	1.37E-05
centroid_13561	phage integrase family protein	66	13	22	24	centroid_13561	16.125983	5.93E-05	centroid_13561	3.95E-05
centroid_1940	conserved hypothetical protein	66	13	22	24	centroid_1940	16.125983	5.93E-05	centroid_1940	3.95E-05
centroid_11068	conserved hypothetical protein	65	11	23	26	centroid_11068	19.476254	1.02E-05	centroid_11068	6.48E-06
centroid_10953	baseplate J-like family protein	65	12	23	25	centroid_10953	17.191479	3.38E-05	centroid_10953	2.04E-05
centroid_10126	conserved hypothetical protein	64	10	24	27	centroid_10126	20.670842	5.45E-06	centroid_10126	2.98E-06
centroid_5033	conserved hypothetical protein	64	10	24	27	centroid_5033	20.670842	5.45E-06	centroid_5033	2.98E-06
centroid_10127	cold shock-like protein CspG	64	11	24	26	centroid_10127	18.313946	1.87E-05	centroid_10127	1.65E-05
centroid_10226	baseplate J-like family protein	64	11	24	26	centroid_10226	18.313946	1.87E-05	centroid_10226	1.65E-05
centroid_12178	putative ynfN	64	11	24	26	centroid_12178	18.313946	1.87E-05	centroid_12178	1.65E-05
centroid_5031	conserved hypothetical protein	64	11	24	26	centroid_5031	18.313946	1.87E-05	centroid_5031	1.65E-05
centroid_8212	conserved hypothetical protein	64	11	24	26	centroid_8212	18.313946	1.87E-05	centroid_8212	1.65E-05
centroid_5030	gnsA/GnsB family protein	62	11	26	26	centroid_5030	16.145441	5.87E-05	centroid_5030	4.90E-05
centroid_10248	bacteriophage replication gene A family protein	57	5	31	32	centroid_10248	25.366166	4.74E-07	centroid_10248	1.34E-07
centroid_5110	PIN domain protein	56	8	32	29	centroid_5110	16.759828	4.24E-05	centroid_5110	2.64E-05
centroid_4328	electron transport complex, RnfABCDGE type, C subunit	51	37	37	0	centroid_4328	20.12612	7.25E-06	centroid_4328	1.81E-07
centroid_10643	putative dNA primase competative inhibitor domain protein	47	4	41	33	centroid_10643	17.845456	2.40E-05	centroid_10643	6.84E-06
centroid_12376	hypothetical protein	47	5	41	32	centroid_12376	15.462783	8.41E-05	centroid_12376	2.51E-05
centroid_10359	conserved hypothetical protein	44	34	44	3	centroid_10359	17.738688	2.53E-05	centroid_10359	4.96E-06
centroid_14162	initiator Replication family protein	43	0	45	37	centroid_14162	25.437486	4.57E-07	centroid_14162	4.56E-09
centroid_4143	initiator Replication family protein	42	0	46	37	centroid_4143	24.498796	7.44E-07	centroid_4143	1.06E-08
centroid_4684	plasmid stability family protein	42	2	46	35	centroid_4684	18.641018	1.58E-05	centroid_4684	1.58E-06
centroid_5169	putative membrane protein	42	2	46	35	centroid_5169	18.641018	1.58E-05	centroid_5169	1.58E-06
centroid_10625	putative mobA domain protein	42	3	46	34	centroid_10625	16.068159	6.11E-05	centroid_10625	1.23E-05
centroid_3982	hypothetical protein	41	34	47	3	centroid_3982	20.425433	6.20E-06	centroid_3982	8.87E-07
centroid_3983	conserved hypothetical protein	41	34	47	3	centroid_3983	20.425433	6.20E-06	centroid_3983	8.87E-07
centroid_4242	conserved hypothetical protein	41	34	47	3	centroid_4242	20.425433	6.20E-06	centroid_4242	8.87E-07
centroid_11088	putative integrase core domain protein	41	33	47	4	centroid_11088	17.845456	2.40E-05	centroid_11088	6.84E-06
centroid_16011	conserved hypothetical protein	41	33	47	4	centroid_16011	17.845456	2.40E-05	centroid_16011	6.84E-06
centroid_10641	hypothetical protein	39	0	49	37	centroid_10641	21.814004	3.00E-06	centroid_10641	7.34E-08
centroid_3978	R.Ecl18kI	39	0	49	37	centroid_3978	21.814004	3.00E-06	centroid_3978	7.34E-08
centroid_3980	conserved hypothetical protein	39	0	49	37	centroid_3980	21.814004	3.00E-06	centroid_3980	7.34E-08
centroid_4038	helix-turn-helix domain protein	39	33	49	4	centroid_4038	19.676244	9.17E-06	centroid_4038	2.69E-06
centroid_4687	bacteriophage replication gene A family protein	38	1	50	36	centroid_4687	18.04247	2.16E-05	centroid_4687	1.50E-06
centroid_14139	conserved hypothetical protein	38	2	50	35	centroid_14139	15.390775	8.74E-05	centroid_14139	1.55E-05
centroid_8561	putative transposase	37	33	51	4	centroid_8561	21.620986	3.32E-06	centroid_8561	1.02E-06
centroid_3979	DNA (cytosine-5-)-methyltransferase family protein	37	0	51	37	centroid_3979	20.12612	7.25E-06	centroid_3979	1.81E-07
centroid_13566	conserved hypothetical protein	37	1	51	36	centroid_13566	17.241452	3.29E-05	centroid_13566	3.23E-06
centroid_5446	conserved hypothetical protein	36	34	52	3	centroid_5446	25.447588	4.55E-07	centroid_5446	4.59E-08
centroid_6892	conserved hypothetical protein	36	34	52	3	centroid_6892	25.447588	4.55E-07	centroid_6892	4.59E-08
centroid_4940	thiol peroxidase	36	0	52	37	centroid_4940	19.310722	1.11E-05	centroid_4940	2.28E-07
centroid_3981	rop family protein	35	0	53	37	centroid_3981	18.513517	1.69E-05	centroid_3981	4.55E-07
centroid_6825	hypothetical protein	32	0	56	37	centroid_6825	16.225244	5.62E-05	centroid_6825	2.34E-06
centroid_10415	hypothetical protein	31	28	57	9	centroid_10415	15.515687	8.18E-05	centroid_10415	6.30E-05
centroid_16109	putative parB-like partitioning protein	31	28	57	9	centroid_16109	15.515687	8.18E-05	centroid_16109	6.30E-05
centroid_13545	hypothetical protein	23	26	65	11	centroid_13545	19.476254	1.02E-05	centroid_13545	6.48E-06
centroid_12243	conserved hypothetical protein	18	25	70	12	centroid_12243	23.575659	1.20E-06	centroid_12243	9.11E-07
centroid_8121	conserved hypothetical protein	16	26	72	11	centroid_8121	29.385732	5.93E-08	centroid_8121	4.70E-08
centroid_7857	putative yceA protein	15	23	73	14	centroid_7857	22.972182	1.64E-06	centroid_7857	1.35E-06
centroid_16743	integrase core domain protein	15	20	73	17	centroid_16743	15.908436	6.65E-05	centroid_16743	5.75E-05
centroid_10644	phage portal protein, lambda family	14	22	74	15	centroid_10644	22.015682	2.70E-06	centroid_10644	2.43E-06
centroid_15283	conserved hypothetical protein	14	20	74	17	centroid_15283	17.262401	3.26E-05	centroid_15283	3.92E-05
centroid_9698	conserved hypothetical protein	13	22	75	15	centroid_9698	23.632272	1.17E-06	centroid_9698	1.37E-06
centroid_11141	haemolysin expression modulating family protein	13	20	75	17	centroid_11141	18.713201	1.52E-05	centroid_11141	1.36E-05
centroid_17734	caudovirales tail fibre assembly family protein	13	19	75	18	centroid_17734	16.428421	5.05E-05	centroid_17734	7.52E-05
centroid_12696	resolvase, N terminal domain protein	12	22	76	15	centroid_12696	25.355585	4.77E-07	centroid_12696	4.53E-07
centroid_17368	conserved hypothetical protein	12	20	76	17	centroid_17368	20.269422	6.73E-06	centroid_17368	7.62E-06
centroid_5345	phage Tail Collar domain protein	12	18	76	19	centroid_5345	15.639228	7.66E-05	centroid_5345	6.65E-05
centroid_13202	HNH endonuclease family protein	11	18	77	19	centroid_13202	17.12839	3.49E-05	centroid_13202	3.76E-05
centroid_16015	resolvase, N terminal domain protein	10	23	78	14	centroid_16015	32.02854	1.52E-08	centroid_16015	1.71E-08
centroid_5993	resolvase, N terminal domain protein	10	23	78	14	centroid_5993	32.02854	1.52E-08	centroid_5993	1.71E-08
centroid_15181	resolvase, N terminal domain protein	10	22	78	15	centroid_15181	29.160808	6.66E-08	centroid_15181	7.44E-08
centroid_15371	putative regulatory domain protein	10	19	78	18	centroid_15371	21.186023	4.17E-06	centroid_15371	4.18E-06
centroid_6754	prophage CP4-57 regulatory family protein	10	19	78	18	centroid_6754	21.186023	4.17E-06	centroid_6754	4.18E-06
centroid_11766	putative antigen 43 domain protein	10	18	78	19	centroid_11766	18.742337	1.50E-05	centroid_11766	2.56E-05
centroid_12617	phage-related baseplate assembly family protein	10	18	78	19	centroid_12617	18.742337	1.50E-05	centroid_12617	2.56E-05

centroid_15154	rhs element Vgr family protein	10	18	78	19	centroid_15154	18.742337	1.50E-05	centroid_15154	2.56E-05
centroid_9125	HNH endonuclease family protein	10	18	78	19	centroid_9125	18.742337	1.50E-05	centroid_9125	2.56E-05
centroid_9597	hok/gef family protein	8	22	80	15	centroid_9597	33.521195	7.05E-09	centroid_9597	9.35E-09
centroid_15103	hok/gef family protein	8	21	80	16	centroid_15103	30.59408	3.18E-08	centroid_15103	4.15E-08
centroid_5795	phage terminase large subunit family protein	8	20	80	17	centroid_5795	27.764001	1.37E-07	centroid_5795	1.73E-07
centroid_15117	conserved hypothetical protein	8	16	80	21	centroid_15117	17.444474	2.96E-05	centroid_15117	2.92E-05
centroid_14826	conserved hypothetical protein	7	18	81	19	centroid_14826	24.476447	7.52E-07	centroid_14826	9.41E-07
centroid_9752	conserved hypothetical protein	7	18	81	19	centroid_9752	24.476447	7.52E-07	centroid_9752	9.41E-07
centroid_12267	conserved hypothetical protein	7	16	81	21	centroid_12267	19.317774	1.11E-05	centroid_12267	1.20E-05
centroid_11622	putative dLP12 prophage; DNA base-flipping protein	7	15	81	22	centroid_11622	16.891252	3.96E-05	centroid_11622	3.97E-05
centroid_6120	replication regulatory RepB family protein	7	15	81	22	centroid_6120	16.891252	3.96E-05	centroid_6120	3.97E-05
centroid_10579	RHS repeat-associated core domain protein	6	17	82	20	centroid_10579	24.018421	9.54E-07	centroid_10579	1.24E-06
centroid_14606	integron integrase family protein	6	16	82	21	centroid_14606	21.385129	3.76E-06	centroid_14606	4.52E-06
centroid_15131	antirestriction family protein	6	15	82	22	centroid_15131	18.848344	1.42E-05	centroid_15131	1.57E-05
centroid_15999	istB-like ATP binding family protein	5	17	83	20	centroid_15999	26.408443	2.76E-07	centroid_15999	3.94E-07
centroid_8682	integrase core domain protein	5	16	83	21	centroid_8682	23.673544	1.14E-06	centroid_8682	1.52E-06
centroid_13428	putative transposase subunit 1	5	15	83	22	centroid_13428	21.028143	4.53E-06	centroid_13428	5.57E-06
centroid_15643	RHS repeat-associated core domain protein	5	15	83	22	centroid_15643	21.028143	4.53E-06	centroid_15643	5.57E-06
centroid_8522	bacterial dnaA family protein	5	15	83	22	centroid_8522	21.028143	4.53E-06	centroid_8522	5.57E-06
centroid_15941	putative gifsy-1 prophage VtiI	5	14	83	23	centroid_15941	18.475559	1.72E-05	centroid_15941	1.94E-05
centroid_6210	integrase	5	13	83	24	centroid_6210	16.020273	6.27E-05	centroid_6210	6.46E-05
centroid_18684	putative transposase subunit	4	15	84	22	centroid_18684	23.465011	1.27E-06	centroid_18684	1.73E-06
centroid_8521	putative transposase	4	15	84	22	centroid_8521	23.465011	1.27E-06	centroid_8521	1.73E-06
centroid_10383	regulatory protein rop	4	13	84	24	centroid_10383	18.221393	1.97E-05	centroid_10383	2.26E-05
centroid_12799	transposase family protein	4	13	84	24	centroid_12799	18.221393	1.97E-05	centroid_12799	2.26E-05
centroid_12811	putative tail length tape measure domain protein	4	13	84	24	centroid_12811	18.221393	1.97E-05	centroid_12811	2.26E-05
centroid_15841	endodeoxyribonuclease RusA family protein	4	13	84	24	centroid_15841	18.221393	1.97E-05	centroid_15841	2.26E-05
centroid_9697	antitermination family protein	4	13	84	24	centroid_9697	18.221393	1.97E-05	centroid_9697	2.26E-05
centroid_10378	hypothetical protein	4	12	84	25	centroid_10378	15.736435	7.28E-05	centroid_10378	7.60E-05
centroid_10379	conserved hypothetical protein	4	12	84	25	centroid_10379	15.736435	7.28E-05	centroid_10379	7.60E-05
centroid_10380	conserved hypothetical protein	4	12	84	25	centroid_10380	15.736435	7.28E-05	centroid_10380	7.60E-05
centroid_10602	hypothetical protein	4	12	84	25	centroid_10602	15.736435	7.28E-05	centroid_10602	7.60E-05
centroid_15173	transposase family protein	4	12	84	25	centroid_15173	15.736435	7.28E-05	centroid_15173	7.60E-05
centroid_8523	cytoskeleton-binding toxin CbtA	4	12	84	25	centroid_8523	15.736435	7.28E-05	centroid_8523	7.60E-05
centroid_10382	exc1 domain protein	3	13	85	24	centroid_10382	20.733385	5.28E-06	centroid_10382	6.66E-06
centroid_11463	conserved hypothetical protein	3	13	85	24	centroid_11463	20.733385	5.28E-06	centroid_11463	6.66E-06
centroid_15687	helix-turn-helix domain protein	3	13	85	24	centroid_15687	20.733385	5.28E-06	centroid_15687	6.66E-06
centroid_6060	putative transposase	3	13	85	24	centroid_6060	20.733385	5.28E-06	centroid_6060	6.66E-06
centroid_15820	ead/Ea22-like family protein	3	12	85	25	centroid_15820	18.120539	2.07E-05	centroid_15820	2.40E-05
centroid_10381	homeo-like domain protein	3	11	85	26	centroid_10381	15.594175	7.85E-05	centroid_10381	8.26E-05
centroid_10877	tail needle protein gp26	3	11	85	26	centroid_10877	15.594175	7.85E-05	centroid_10877	8.26E-05
centroid_16664	yafA domain protein	3	11	85	26	centroid_16664	15.594175	7.85E-05	centroid_16664	8.26E-05
centroid_13441	conserved hypothetical protein	2	34	86	3	centroid_13441	97.700647	4.87E-23	centroid_13441	9.72E-25
centroid_13442	conserved hypothetical protein	2	34	86	3	centroid_13442	97.700647	4.87E-23	centroid_13442	9.72E-25
centroid_13443	hindVP restriction endonuclease family protein	2	34	86	3	centroid_13443	97.700647	4.87E-23	centroid_13443	9.72E-25
centroid_13444	DNA (cytosine-5-)-methyltransferase family protein	2	34	86	3	centroid_13444	97.700647	4.87E-23	centroid_13444	9.72E-25
centroid_17138	hypothetical protein	2	34	86	3	centroid_17138	97.700647	4.87E-23	centroid_17138	9.72E-25
centroid_12233	putative entry exclusion protein 1	2	15	86	22	centroid_12233	29.287983	6.24E-08	centroid_12233	9.26E-08
centroid_15716	putative entry exclusion protein 1	2	15	86	22	centroid_15716	29.287983	6.24E-08	centroid_15716	9.26E-08
centroid_11770	conserved hypothetical protein	2	13	86	24	centroid_11770	23.617384	1.18E-06	centroid_11770	1.56E-06
centroid_12234	putative membrane protein	2	13	86	24	centroid_12234	23.617384	1.18E-06	centroid_12234	1.56E-06
centroid_12235	conserved hypothetical protein	2	13	86	24	centroid_12235	23.617384	1.18E-06	centroid_12235	1.56E-06
centroid_15176	conserved hypothetical protein	2	13	86	24	centroid_15176	23.617384	1.18E-06	centroid_15176	1.56E-06
centroid_15717	conserved hypothetical protein	2	13	86	24	centroid_15717	23.617384	1.18E-06	centroid_15717	1.56E-06
centroid_17514	hsdM N-terminal domain protein	2	13	86	24	centroid_17514	23.617384	1.18E-06	centroid_17514	1.56E-06
centroid_6005	CFA/I fimbrial subunit D	2	13	86	24	centroid_6005	23.617384	1.18E-06	centroid_6005	1.56E-06
centroid_12232	putative entry exclusion protein 2	2	12	86	25	centroid_12232	20.887096	4.87E-06	centroid_12232	6.05E-06
centroid_18634	type VII secretion system (T7SS), usher family protein	2	12	86	25	centroid_18634	20.887096	4.87E-06	centroid_18634	6.05E-06
centroid_5998	putative transposase domain protein	2	12	86	25	centroid_5998	20.887096	4.87E-06	centroid_5998	6.05E-06
centroid_6061	SEF14-like adhesin family protein	2	12	86	25	centroid_6061	20.887096	4.87E-06	centroid_6061	6.05E-06
centroid_6067	hypothetical protein	2	12	86	25	centroid_6067	20.887096	4.87E-06	centroid_6067	6.05E-06
centroid_6357	type I restriction-modification system, M subunit	2	12	86	25	centroid_6357	20.887096	4.87E-06	centroid_6357	6.05E-06
centroid_10337	T5orf172 domain protein	2	11	86	26	centroid_10337	18.230087	1.96E-05	centroid_10337	2.25E-05
centroid_13223	conserved hypothetical protein	2	11	86	26	centroid_13223	18.230087	1.96E-05	centroid_13223	2.25E-05
centroid_14810	transposase IS116/IS110/IS902 family protein	2	11	86	26	centroid_14810	18.230087	1.96E-05	centroid_14810	2.25E-05
centroid_15411	protein CexE	2	11	86	26	centroid_15411	18.230087	1.96E-05	centroid_15411	2.25E-05
centroid_15927	putative membrane protein	2	11	86	26	centroid_15927	18.230087	1.96E-05	centroid_15927	2.25E-05
centroid_16102	conserved hypothetical protein	2	11	86	26	centroid_16102	18.230087	1.96E-05	centroid_16102	2.25E-05
centroid_17015	gram-negative pili assembly chaperone, N-terminal domain protein	2	11	86	26	centroid_17015	18.230087	1.96E-05	centroid_17015	2.25E-05
centroid_5793	conserved hypothetical protein	2	11	86	26	centroid_5793	18.230087	1.96E-05	centroid_5793	2.25E-05
centroid_5999	transposase family protein	2	11	86	26	centroid_5999	18.230087	1.96E-05	centroid_5999	2.25E-05
centroid_6000	ABC transporter family protein	2	11	86	26	centroid_6000	18.230087	1.96E-05	centroid_6000	2.25E-05
centroid_6001	putative transporter protein AatB	2	11	86	26	centroid_6001	18.230087	1.96E-05	centroid_6001	2.25E-05
centroid_6002	outer membrane efflux family protein	2	11	86	26	centroid_6002	18.230087	1.96E-05	centroid_6002	2.25E-05
centroid_6003	permease family protein	2	11	86	26	centroid_6003	18.230087	1.96E-05	centroid_6003	2.25E-05
centroid_6059	serine protease SepA autotransporter	2	11	86	26	centroid_6059	18.230087	1.96E-05	centroid_6059	2.25E-05
centroid_6062	type VII secretion system (T7SS), usher family protein	2	11	86	26	centroid_6062	18.230087	1.96E-05	centroid_6062	2.25E-05
centroid_6066	putative membrane protein	2	11	86	26	centroid_6066	18.230087	1.96E-05	centroid_6066	2.25E-05
centroid_6068	hypothetical protein	2	11	86	26	centroid_6068	18.230087	1.96E-05	centroid_6068	2.25E-05
centroid_6894	integrase core domain protein	2	11	86	26	centroid_6894	18.230087	1.96E-05	centroid_6894	2.25E-05
centroid_15906	N-6 DNA Methylase family protein	2	10	86	27	centroid_15906	15.650488	7.62E-05	centroid_15906	8.01E-05
centroid_5615	resolvase, N terminal domain protein	2	10	86	27	centroid_5615	15.650488	7.62E-05	centroid_5615	8.01E-05
centroid_6014	conserved hypothetical protein	2	10	86	27	centroid_6014	15.650488	7.62E-05	centroid_6014	8.01E-05
centroid_6015	conserved hypothetical protein	2	10	86	27	centroid_6015	15.650488	7.62E-05	centroid_6015	8.01E-05
centroid_6016	conserved hypothetical protein	2	10	86	27	centroid_6016	15.650488	7.62E-05	centroid_6016	8.01E-05
centroid_6017	conserved hypothetical protein	2	10	86	27	centroid_6017	15.650488	7.62E-05	centroid_6017	8.01E-05
centroid_6018	putative translation elongation factor P domain protein	2	10	86	27	centroid_6018	15.650488	7.62E-05	centroid_6018	8.01E-05
centroid_6019	conserved hypothetical protein	2	10	86	27	centroid_6019	15.650488	7.62E-05	centroid_6019	8.01E-05
centroid_9613	csp231I C protein	2	10	86	27	centroid_9613	15.650488	7.62E-05	centroid_9613	8.01E-05
centroid_9614	putative dNA-directed RNA polymerase beta subunit	2	10	86	27	centroid_9614	15.650488	7.62E-05	centroid_9614	8.01E-05
centroid_13240	C-5 cytosine-specific DNA methylase family protein	1	25	87	12	centroid_13240	65.805538	4.98E-16	centroid_13240	3.28E-16
centroid_13241	putative r.SnaBI endonuclease	1	25	87	12	centroid_13241	65.805538	4.98E-16	centroid_13241	3.28E-16
centroid_13242	helix-turn-helix family protein	1	25	87	12	centroid_13242	65.805538	4.98E-16	centroid_13242	3.28E-16
centroid_13418	integrase core domain protein	1	16	87	21	centroid_13418	35.801432	2.18E-09	centroid_13418	2.88E-09
centroid_11972	putative gp55	1	14	87	23	centroid_11972	29.841324	4.69E-08	centroid_11972	6.03E-08
centroid_11974	conserved hypothetical protein	1	14	87	23	centroid_11974	29.841324	4.69E-08	centroid_11974	6.03E-08
centroid_11975	conserved hypothetical protein	1	14	87	23	centroid_11975	29.841324	4.69E-08	centroid_11975	6.03E-08
centroid_11976	conserved hypothetical protein	1	14	87	23	centroid_11976	29.841324	4.69E-08	centroid_11976	6.03E-08
centroid_11984	conserved hypothetical protein	1	14	87	23	centroid_11984	29.841324	4.69E-08	centroid_11984	6.03E-08
centroid_11985	hypothetical protein	1	14	87	23	centroid_11985	29.841324	4.69E-08	centroid_11985	6.03E-08
centroid_14867	conserved hypothetical protein	1	14	87	23	centroid_14867	29.841324	4.69E-08	centroid_14867	6.03E-08
centroid_11980	putative gp16	1	13	87	24	centroid_11980	26.952031	2.09E-07	centroid_11980	2.61E-07
centroid_11981	marR family protein	1	13	87	24	centroid_11981	26.952031	2.09E-07	centroid_11981	2.61E-07
centroid_12299	conserved hypothetical protein	1	13	87	24	centroid_12299	26.952031	2.09E-07	centroid_12299	2.61E-07
centroid_13167	conserved hypothetical protein	1	12	87	25	centroid_13167	24.12316	9.04E-07	centroid_13167	1.09E-06
centroid_17716	conserved hypothetical protein	1	12	87	25	centroid_17716	24.12316	9.04E-07	centroid_17716	1.09E-06

centroid_18633	putative transposase	1	12	87	25	centroid_18633	24.12316	9.04E-07	centroid_18633	1.09E-06
centroid_9090	conserved hypothetical protein	1	12	87	25	centroid_9090	24.12316	9.04E-07	centroid_9090	1.09E-06
centroid_11693	bacteriophage lysis family protein	1	11	87	26	centroid_11693	21.355295	3.82E-06	centroid_11693	4.38E-06
centroid_11732	HNH endonuclease family protein	1	11	87	26	centroid_11732	21.355295	3.82E-06	centroid_11732	4.38E-06
centroid_11739	conserved hypothetical protein	1	11	87	26	centroid_11739	21.355295	3.82E-06	centroid_11739	4.38E-06
centroid_11740	conserved hypothetical protein	1	11	87	26	centroid_11740	21.355295	3.82E-06	centroid_11740	4.38E-06
centroid_11741	immunoglobulin domain protein	1	11	87	26	centroid_11741	21.355295	3.82E-06	centroid_11741	4.38E-06
centroid_11742	phage tail assembly chaperone family protein	1	11	87	26	centroid_11742	21.355295	3.82E-06	centroid_11742	4.38E-06
centroid_11743	conserved hypothetical protein	1	11	87	26	centroid_11743	21.355295	3.82E-06	centroid_11743	4.38E-06
centroid_11744	phage tail tape measure protein, lambda family	1	11	87	26	centroid_11744	21.355295	3.82E-06	centroid_11744	4.38E-06
centroid_11745	phage minor tail family protein	1	11	87	26	centroid_11745	21.355295	3.82E-06	centroid_11745	4.38E-06
centroid_11872	cupin fold metallo, WbuC family protein	1	11	87	26	centroid_11872	21.355295	3.82E-06	centroid_11872	4.38E-06
centroid_11873	glycosyl transferases group 1 family protein	1	11	87	26	centroid_11873	21.355295	3.82E-06	centroid_11873	4.38E-06
centroid_11874	UDP-N-acetylglucosamine 2-epimerase	1	11	87	26	centroid_11874	21.355295	3.82E-06	centroid_11874	4.38E-06
centroid_11875	NAD dependent epimerase/dehydratase family protein	1	11	87	26	centroid_11875	21.355295	3.82E-06	centroid_11875	4.38E-06
centroid_11877	glycosyl transferases group 1 family protein	1	11	87	26	centroid_11877	21.355295	3.82E-06	centroid_11877	4.38E-06
centroid_12067	methyltransferase domain protein	1	11	87	26	centroid_12067	21.355295	3.82E-06	centroid_12067	4.38E-06
centroid_13168	conserved hypothetical protein	1	11	87	26	centroid_13168	21.355295	3.82E-06	centroid_13168	4.38E-06
centroid_15976	conserved hypothetical protein	1	11	87	26	centroid_15976	21.355295	3.82E-06	centroid_15976	4.38E-06
centroid_16037	conserved hypothetical protein	1	11	87	26	centroid_16037	21.355295	3.82E-06	centroid_16037	4.38E-06
centroid_6658	HNH endonuclease family protein	1	11	87	26	centroid_6658	21.355295	3.82E-06	centroid_6658	4.38E-06
centroid_6659	sensory box protein	1	11	87	26	centroid_6659	21.355295	3.82E-06	centroid_6659	4.38E-06
centroid_6699	bacterial regulatory, tetR family protein	1	11	87	26	centroid_6699	21.355295	3.82E-06	centroid_6699	4.38E-06
centroid_12069	ncrBC 5-methylcytosine restriction system component family protei	1	10	87	27	centroid_12069	18.649788	1.57E-05	centroid_12069	1.70E-05
centroid_12907	putative transposase	1	10	87	27	centroid_12907	18.649788	1.57E-05	centroid_12907	1.70E-05
centroid_15239	integrase core domain protein	1	10	87	27	centroid_15239	18.649788	1.57E-05	centroid_15239	1.70E-05
centroid_16486	AAA domain family protein	1	10	87	27	centroid_16486	18.649788	1.57E-05	centroid_16486	1.70E-05
centroid_17513	methyltransferase domain protein	1	10	87	27	centroid_17513	18.649788	1.57E-05	centroid_17513	1.70E-05
centroid_17573	nucleotidyltransferase domain protein	1	10	87	27	centroid_17573	18.649788	1.57E-05	centroid_17573	1.70E-05
centroid_6660	coA-transferase III family protein	1	10	87	27	centroid_6660	18.649788	1.57E-05	centroid_6660	1.70E-05
centroid_6661	HMGL-like family protein	1	10	87	27	centroid_6661	18.649788	1.57E-05	centroid_6661	1.70E-05
centroid_6662	sugar (and other) transporter family protein	1	10	87	27	centroid_6662	18.649788	1.57E-05	centroid_6662	1.70E-05
centroid_8929	integrase core domain protein	1	10	87	27	centroid_8929	18.649788	1.57E-05	centroid_8929	1.70E-05
centroid_9093	ead/Ea22-like family protein	1	10	87	27	centroid_9093	18.649788	1.57E-05	centroid_9093	1.70E-05
centroid_10121	conserved hypothetical protein	1	9	87	28	centroid_10121	16.009115	6.30E-05	centroid_10121	6.37E-05
centroid_11855	ninB family protein	1	9	87	28	centroid_11855	16.009115	6.30E-05	centroid_11855	6.37E-05
centroid_12068	ATPase associated with various cellular activities family protein	1	9	87	28	centroid_12068	16.009115	6.30E-05	centroid_12068	6.37E-05
centroid_12109	conserved hypothetical protein	1	9	87	28	centroid_12109	16.009115	6.30E-05	centroid_12109	6.37E-05
centroid_13971	conserved hypothetical protein	1	9	87	28	centroid_13971	16.009115	6.30E-05	centroid_13971	6.37E-05
centroid_13972	ERF superfamily protein	1	9	87	28	centroid_13972	16.009115	6.30E-05	centroid_13972	6.37E-05
centroid_13978	regulatory protein cro	1	9	87	28	centroid_13978	16.009115	6.30E-05	centroid_13978	6.37E-05
centroid_13980	dnaB-like helicase C terminal domain protein	1	9	87	28	centroid_13980	16.009115	6.30E-05	centroid_13980	6.37E-05
centroid_15260	phage tail fibre repeat family protein	1	9	87	28	centroid_15260	16.009115	6.30E-05	centroid_15260	6.37E-05
centroid_15548	hypothetical protein	1	9	87	28	centroid_15548	16.009115	6.30E-05	centroid_15548	6.37E-05
centroid_17625	conserved hypothetical protein	1	9	87	28	centroid_17625	16.009115	6.30E-05	centroid_17625	6.37E-05
centroid_14499	hypothetical protein	0	20	88	17	centroid_14499	52.677589	3.93E-13	centroid_14499	2.22E-13
centroid_14496	hypothetical protein	0	19	88	18	centroid_14496	49.379662	2.11E-12	centroid_14496	1.31E-12
centroid_15928	hypothetical protein	0	19	88	18	centroid_15928	49.379662	2.11E-12	centroid_15928	1.31E-12
centroid_17181	hypothetical protein	0	19	88	18	centroid_17181	49.379662	2.11E-12	centroid_17181	1.31E-12
centroid_17394	hypothetical protein	0	19	88	18	centroid_17394	49.379662	2.11E-12	centroid_17394	1.31E-12
centroid_14498	hypothetical protein	0	18	88	19	centroid_14498	46.143788	1.10E-11	centroid_14498	7.37E-12
centroid_14915	mbeD/MobD like family protein	0	18	88	19	centroid_14915	46.143788	1.10E-11	centroid_14915	7.37E-12
centroid_14916	mbeB-like, N-term conserved region family protein	0	18	88	19	centroid_14916	46.143788	1.10E-11	centroid_14916	7.37E-12
centroid_14495	replication family protein	0	17	88	20	centroid_14495	42.968316	5.56E-11	centroid_14495	3.98E-11
centroid_14640	conserved hypothetical protein	0	17	88	20	centroid_14640	42.968316	5.56E-11	centroid_14640	3.98E-11
centroid_14641	DNA relaxase MbeA	0	17	88	20	centroid_14641	42.968316	5.56E-11	centroid_14641	3.98E-11
centroid_14497	mobA/MobL family protein	0	16	88	21	centroid_14497	39.851671	2.74E-10	centroid_14497	2.07E-10
centroid_14827	integrase core domain protein	0	16	88	21	centroid_14827	39.851671	2.74E-10	centroid_14827	2.07E-10
centroid_14908	mobA/MobL family protein	0	16	88	21	centroid_14908	39.851671	2.74E-10	centroid_14908	2.07E-10
centroid_15762	bacterial regulatory , Fis family protein	0	15	88	22	centroid_15762	36.792361	1.31E-09	centroid_15762	1.03E-09
centroid_17401	pentapeptide repeats family protein	0	15	88	22	centroid_17401	36.792361	1.31E-09	centroid_17401	1.03E-09
centroid_14863	phage tail sheath family protein	0	14	88	23	centroid_14863	33.788978	6.14E-09	centroid_14863	4.99E-09
centroid_15760	conserved hypothetical protein	0	14	88	23	centroid_15760	33.788978	6.14E-09	centroid_15760	4.99E-09
centroid_15761	qnrB1	0	14	88	23	centroid_15761	33.788978	6.14E-09	centroid_15761	4.99E-09
centroid_16135	hypothetical protein	0	14	88	23	centroid_16135	33.788978	6.14E-09	centroid_16135	4.99E-09
centroid_14864	conserved hypothetical protein	0	13	88	24	centroid_14864	30.840207	2.80E-08	centroid_14864	2.33E-08
centroid_14865	putative lipoprotein	0	13	88	24	centroid_14865	30.840207	2.80E-08	centroid_14865	2.33E-08
centroid_14868	DNA adenine methylase family protein	0	13	88	24	centroid_14868	30.840207	2.80E-08	centroid_14868	2.33E-08
centroid_14642	mobilization protein MbeC	0	12	88	25	centroid_14642	27.944843	1.25E-07	centroid_14642	1.05E-07
centroid_14917	firmicute plasmid replication family protein	0	12	88	25	centroid_14917	27.944843	1.25E-07	centroid_14917	1.05E-07
centroid_14918	putative oRF3	0	12	88	25	centroid_14918	27.944843	1.25E-07	centroid_14918	1.05E-07
centroid_18675	putative variable tail fiber protein	0	12	88	25	centroid_18675	27.944843	1.25E-07	centroid_18675	1.05E-07
centroid_14919	putative taxA	0	11	88	26	centroid_14919	25.101807	5.44E-07	centroid_14919	4.61E-07
centroid_15183	type-2 restriction enzyme Cfr10I	0	11	88	26	centroid_15183	25.101807	5.44E-07	centroid_15183	4.61E-07
centroid_15226	integrase	0	11	88	26	centroid_15226	25.101807	5.44E-07	centroid_15226	4.61E-07
centroid_15228	DEAD/DEAH box helicase family protein	0	11	88	26	centroid_15228	25.101807	5.44E-07	centroid_15228	4.61E-07
centroid_15229	conserved hypothetical protein	0	11	88	26	centroid_15229	25.101807	5.44E-07	centroid_15229	4.61E-07
centroid_15272	dTDP-4-dehydrorhamnose 3,5-epimerase	0	11	88	26	centroid_15272	25.101807	5.44E-07	centroid_15272	4.61E-07
centroid_15273	glycosyl transferase 2 family protein	0	11	88	26	centroid_15273	25.101807	5.44E-07	centroid_15273	4.61E-07
centroid_15274	glycosyl transferases group 1 family protein	0	11	88	26	centroid_15274	25.101807	5.44E-07	centroid_15274	4.61E-07
centroid_15275	putative membrane protein	0	11	88	26	centroid_15275	25.101807	5.44E-07	centroid_15275	4.61E-07
centroid_15276	bacterial transferase hexapeptide family protein	0	11	88	26	centroid_15276	25.101807	5.44E-07	centroid_15276	4.61E-07
centroid_15277	UDP-N-acetylglucosamine 4,6-dehydratase/5-epimerase	0	11	88	26	centroid_15277	25.101807	5.44E-07	centroid_15277	4.61E-07
centroid_15363	phage gp6-like head-tail connector family protein	0	11	88	26	centroid_15363	25.101807	5.44E-07	centroid_15363	4.61E-07
centroid_15364	putative head-tail adaptor	0	11	88	26	centroid_15364	25.101807	5.44E-07	centroid_15364	4.61E-07
centroid_15372	phage portal protein, HK97 family	0	11	88	26	centroid_15372	25.101807	5.44E-07	centroid_15372	4.61E-07
centroid_15373	phage prohead protease, HK97 family	0	11	88	26	centroid_15373	25.101807	5.44E-07	centroid_15373	4.61E-07
centroid_15374	phage major capsid protein, HK97 family	0	11	88	26	centroid_15374	25.101807	5.44E-07	centroid_15374	4.61E-07
centroid_15547	bacteriophage lysis family protein	0	11	88	26	centroid_15547	25.101807	5.44E-07	centroid_15547	4.61E-07
centroid_15738	putative mobilization protein 1	0	11	88	26	centroid_15738	25.101807	5.44E-07	centroid_15738	4.61E-07
centroid_15874	DEAD/DEAH box helicase family protein	0	11	88	26	centroid_15874	25.101807	5.44E-07	centroid_15874	4.61E-07
centroid_16784	putative dead/deah box helicase domain protein	0	11	88	26	centroid_16784	25.101807	5.44E-07	centroid_16784	4.61E-07
centroid_16817	prophage CP4-57 regulatory family protein	0	11	88	26	centroid_16817	25.101807	5.44E-07	centroid_16817	4.61E-07
centroid_18276	polysaccharide biosynthesis family protein	0	11	88	26	centroid_18276	25.101807	5.44E-07	centroid_18276	4.61E-07
centroid_14339	conserved hypothetical protein	0	10	88	27	centroid_14339	22.310192	2.32E-06	centroid_14339	1.96E-06
centroid_14531	mobA/MobL family protein	0	10	88	27	centroid_14531	22.310192	2.32E-06	centroid_14531	1.96E-06
centroid_14954	conserved hypothetical protein	0	10	88	27	centroid_14954	22.310192	2.32E-06	centroid_14954	1.96E-06
centroid_15121	conserved hypothetical protein	0	10	88	27	centroid_15121	22.310192	2.32E-06	centroid_15121	1.96E-06
centroid_15122	conserved hypothetical protein	0	10	88	27	centroid_15122	22.310192	2.32E-06	centroid_15122	1.96E-06
centroid_15162	colicin pore forming domain protein	0	10	88	27	centroid_15162	22.310192	2.32E-06	centroid_15162	1.96E-06
centroid_15164	colicin E1 (microcin) immunity family protein	0	10	88	27	centroid_15164	22.310192	2.32E-06	centroid_15164	1.96E-06
centroid_15227	putative dead/deah box helicase domain protein	0	10	88	27	centroid_15227	22.310192	2.32E-06	centroid_15227	1.96E-06
centroid_15281	phage tail tape measure protein, lambda family	0	10	88	27	centroid_15281	22.310192	2.32E-06	centroid_15281	1.96E-06
centroid_15286	conserved hypothetical protein	0	10	88	27	centroid_15286	22.310192	2.32E-06	centroid_15286	1.96E-06
centroid_15313	type I restriction enzyme, S subunit domain protein	0	10	88	27	centroid_15313	22.310192	2.32E-06	centroid_15313	1.96E-06
centroid_15314	putative membrane protein	0	10	88	27	centroid_15314	22.310192	2.32E-06	centroid_15314	1.96E-06

centroid_15322	polysaccharide biosynthesis/export family protein	0	10	88	27	centroid_15322	22.310192	2.32E-06	centroid_15322	1.96E-06
centroid_15323	ABC-2 type transporter family protein	0	10	88	27	centroid_15323	22.310192	2.32E-06	centroid_15323	1.96E-06
centroid_15324	ABC transporter family protein	0	10	88	27	centroid_15324	22.310192	2.32E-06	centroid_15324	1.96E-06
centroid_15325	lysaccharide export inner-membrane , BexC/CtrB/KpsE family prot	0	10	88	27	centroid_15325	22.310192	2.32E-06	centroid_15325	1.96E-06
centroid_15326	methyltransferase domain protein	0	10	88	27	centroid_15326	22.310192	2.32E-06	centroid_15326	1.96E-06
centroid_15327	glycosyltransferase WbsX family protein	0	10	88	27	centroid_15327	22.310192	2.32E-06	centroid_15327	1.96E-06
centroid_15328	glycosyl transferase 2 family protein	0	10	88	27	centroid_15328	22.310192	2.32E-06	centroid_15328	1.96E-06
centroid_15329	nucleotide sugar dehydrogenase family protein	0	10	88	27	centroid_15329	22.310192	2.32E-06	centroid_15329	1.96E-06
centroid_15330	hypothetical protein	0	10	88	27	centroid_15330	22.310192	2.32E-06	centroid_15330	1.96E-06
centroid_15331	glycosyl transferase 2 family protein	0	10	88	27	centroid_15331	22.310192	2.32E-06	centroid_15331	1.96E-06
centroid_15332	glycosyl transferase 2 family protein	0	10	88	27	centroid_15332	22.310192	2.32E-06	centroid_15332	1.96E-06
centroid_15333	glycosyl transferases group 1 family protein	0	10	88	27	centroid_15333	22.310192	2.32E-06	centroid_15333	1.96E-06
centroid_15334	dTDP-glucose 4,6-dehydratase	0	10	88	27	centroid_15334	22.310192	2.32E-06	centroid_15334	1.96E-06
centroid_15335	dTDP-4-dehydrorhamnose reductase	0	10	88	27	centroid_15335	22.310192	2.32E-06	centroid_15335	1.96E-06
centroid_15336	glucose-1-phosphate thymidylyltransferase	0	10	88	27	centroid_15336	22.310192	2.32E-06	centroid_15336	1.96E-06
centroid_15337	dTDP-4-dehydrorhamnose 3,5-epimerase	0	10	88	27	centroid_15337	22.310192	2.32E-06	centroid_15337	1.96E-06
centroid_15338	capsule polysaccharide biosynthesis family protein	0	10	88	27	centroid_15338	22.310192	2.32E-06	centroid_15338	1.96E-06
centroid_15339	capsule polysaccharide biosynthesis family protein	0	10	88	27	centroid_15339	22.310192	2.32E-06	centroid_15339	1.96E-06
centroid_15340	putative pH01	0	10	88	27	centroid_15340	22.310192	2.32E-06	centroid_15340	1.96E-06
centroid_15347	DNA transfer protein gp7	0	10	88	27	centroid_15347	22.310192	2.32E-06	centroid_15347	1.96E-06
centroid_15353	type I restriction modification DNA specificity domain protein	0	10	88	27	centroid_15353	22.310192	2.32E-06	centroid_15353	1.96E-06
centroid_15354	yecA family protein	0	10	88	27	centroid_15354	22.310192	2.32E-06	centroid_15354	1.96E-06
centroid_15355	'Cold-shock' DNA-binding domain protein	0	10	88	27	centroid_15355	22.310192	2.32E-06	centroid_15355	1.96E-06
centroid_15381	putative transposase	0	10	88	27	centroid_15381	22.310192	2.32E-06	centroid_15381	1.96E-06
centroid_15392	putative outer membrane lipoprotein SlyB	0	10	88	27	centroid_15392	22.310192	2.32E-06	centroid_15392	1.96E-06
centroid_15789	adenine-specific methyltransferase EcoRI family protein	0	10	88	27	centroid_15789	22.310192	2.32E-06	centroid_15789	1.96E-06
centroid_15790	type-2 restriction enzyme EcoRI	0	10	88	27	centroid_15790	22.310192	2.32E-06	centroid_15790	1.96E-06
centroid_15882	tail spike protein	0	10	88	27	centroid_15882	22.310192	2.32E-06	centroid_15882	1.96E-06
centroid_17479	putative yacA	0	10	88	27	centroid_17479	22.310192	2.32E-06	centroid_17479	1.96E-06
centroid_17652	conserved hypothetical protein	0	10	88	27	centroid_17652	22.310192	2.32E-06	centroid_17652	1.96E-06
centroid_18682	conserved hypothetical protein	0	10	88	27	centroid_18682	22.310192	2.32E-06	centroid_18682	1.96E-06
centroid_14835	recT family protein	0	9	88	28	centroid_14835	19.569319	9.70E-06	centroid_14835	8.14E-06
centroid_14839	putative transmembrane anchored domain protein	0	9	88	28	centroid_14839	19.569319	9.70E-06	centroid_14839	8.14E-06
centroid_14840	conserved hypothetical protein	0	9	88	28	centroid_14840	19.569319	9.70E-06	centroid_14840	8.14E-06
centroid_14841	conserved hypothetical protein	0	9	88	28	centroid_14841	19.569319	9.70E-06	centroid_14841	8.14E-06
centroid_14842	putative predicted protein	0	9	88	28	centroid_14842	19.569319	9.70E-06	centroid_14842	8.14E-06
centroid_14844	conserved hypothetical protein	0	9	88	28	centroid_14844	19.569319	9.70E-06	centroid_14844	8.14E-06
centroid_14845	conserved hypothetical protein	0	9	88	28	centroid_14845	19.569319	9.70E-06	centroid_14845	8.14E-06
centroid_14847	putative membrane protein	0	9	88	28	centroid_14847	19.569319	9.70E-06	centroid_14847	8.14E-06
centroid_14848	conserved hypothetical protein	0	9	88	28	centroid_14848	19.569319	9.70E-06	centroid_14848	8.14E-06
centroid_15123	conserved hypothetical protein	0	9	88	28	centroid_15123	19.569319	9.70E-06	centroid_15123	8.14E-06
centroid_15218	prophage tail fibre N-terminal family protein	0	9	88	28	centroid_15218	19.569319	9.70E-06	centroid_15218	8.14E-06
centroid_15223	conserved hypothetical protein	0	9	88	28	centroid_15223	19.569319	9.70E-06	centroid_15223	8.14E-06
centroid_15224	putative transcriptional regulator	0	9	88	28	centroid_15224	19.569319	9.70E-06	centroid_15224	8.14E-06
centroid_15231	lysR family regulatory helix-turn-helix protein	0	9	88	28	centroid_15231	19.569319	9.70E-06	centroid_15231	8.14E-06
centroid_15262	hypothetical protein	0	9	88	28	centroid_15262	19.569319	9.70E-06	centroid_15262	8.14E-06
centroid_15285	conserved hypothetical protein	0	9	88	28	centroid_15285	19.569319	9.70E-06	centroid_15285	8.14E-06
centroid_15357	hypothetical protein	0	9	88	28	centroid_15357	19.569319	9.70E-06	centroid_15357	8.14E-06
centroid_15365	phage minor tail protein L	0	9	88	28	centroid_15365	19.569319	9.70E-06	centroid_15365	8.14E-06
centroid_15376	conserved hypothetical protein	0	9	88	28	centroid_15376	19.569319	9.70E-06	centroid_15376	8.14E-06
centroid_15377	P63C domain protein	0	9	88	28	centroid_15377	19.569319	9.70E-06	centroid_15377	8.14E-06
centroid_15379	DNA transfer gp20 domain protein	0	9	88	28	centroid_15379	19.569319	9.70E-06	centroid_15379	8.14E-06
centroid_15398	putative sel1	0	9	88	28	centroid_15398	19.569319	9.70E-06	centroid_15398	8.14E-06
centroid_15893	peptidase S24-like family protein	0	9	88	28	centroid_15893	19.569319	9.70E-06	centroid_15893	8.14E-06
centroid_15923	dihydrofolate reductase type 1	0	9	88	28	centroid_15923	19.569319	9.70E-06	centroid_15923	8.14E-06
centroid_17083	conserved hypothetical protein	0	9	88	28	centroid_17083	19.569319	9.70E-06	centroid_17083	8.14E-06
centroid_17084	conserved hypothetical protein	0	9	88	28	centroid_17084	19.569319	9.70E-06	centroid_17084	8.14E-06
centroid_17085	phage T7 tail fibre family protein	0	9	88	28	centroid_17085	19.569319	9.70E-06	centroid_17085	8.14E-06
centroid_17111	hypothetical protein	0	9	88	28	centroid_17111	19.569319	9.70E-06	centroid_17111	8.14E-06
centroid_18676	colicin pore forming domain protein	0	9	88	28	centroid_18676	19.569319	9.70E-06	centroid_18676	8.14E-06
centroid_14645	putative transposase	0	8	88	29	centroid_14645	16.878859	3.98E-05	centroid_14645	3.28E-05
centroid_14812	transposase IS66 family protein	0	8	88	29	centroid_14812	16.878859	3.98E-05	centroid_14812	3.28E-05
centroid_14858	conserved hypothetical protein	0	8	88	29	centroid_14858	16.878859	3.98E-05	centroid_14858	3.28E-05
centroid_14926	conserved hypothetical protein	0	8	88	29	centroid_14926	16.878859	3.98E-05	centroid_14926	3.28E-05
centroid_14986	conserved hypothetical protein	0	8	88	29	centroid_14986	16.878859	3.98E-05	centroid_14986	3.28E-05
centroid_15184	DNA (cytosine-5-)-methyltransferase family protein	0	8	88	29	centroid_15184	16.878859	3.98E-05	centroid_15184	3.28E-05
centroid_15225	phage integrase family protein	0	8	88	29	centroid_15225	16.878859	3.98E-05	centroid_15225	3.28E-05
centroid_15375	conserved hypothetical protein	0	8	88	29	centroid_15375	16.878859	3.98E-05	centroid_15375	3.28E-05
centroid_15397	putative entry exclusion protein 2	0	8	88	29	centroid_15397	16.878859	3.98E-05	centroid_15397	3.28E-05
centroid_15883	putative dNA transfer protein gp20	0	8	88	29	centroid_15883	16.878859	3.98E-05	centroid_15883	3.28E-05
centroid_15901	conserved hypothetical protein	0	8	88	29	centroid_15901	16.878859	3.98E-05	centroid_15901	3.28E-05
centroid_15929	phage tail fibre repeat family protein	0	8	88	29	centroid_15929	16.878859	3.98E-05	centroid_15929	3.28E-05
centroid_15961	conserved hypothetical protein	0	8	88	29	centroid_15961	16.878859	3.98E-05	centroid_15961	3.28E-05
centroid_15968	conserved hypothetical protein	0	8	88	29	centroid_15968	16.878859	3.98E-05	centroid_15968	3.28E-05

Table S4. Distribut+A939ion by Geography - Antogasta

Gene_ID	Annotation	ogasta_pres	Other_presen	togasta_Abs	Other_Absent	Gene_ID	chisq-stats	pvalues	Gene_ID	pvalues
centroid_4328	electron transport complex, RnfABCDGE type, C subunit	31	57	0	37	centroid_4328	15.495128	8.27E-05	centroid_4328	5.36E-06
centroid_13441	conserved hypothetical protein	28	8	3	86	centroid_13441	72.154818	1.99E-17	centroid_13441	1.65E-17
centroid_13442	conserved hypothetical protein	28	8	3	86	centroid_13442	72.154818	1.99E-17	centroid_13442	1.65E-17
centroid_13443	hindVP restriction endonuclease family protein	28	8	3	86	centroid_13443	72.154818	1.99E-17	centroid_13443	1.65E-17
centroid_13444	DNA (cytosine-5-)-methyltransferase family protein	28	8	3	86	centroid_13444	72.154818	1.99E-17	centroid_13444	1.65E-17
centroid_17138	hypothetical protein	28	8	3	86	centroid_17138	72.154818	1.99E-17	centroid_17138	1.65E-17
centroid_5446	conserved hypothetical protein	28	42	3	52	centroid_5446	17.900109	2.33E-05	centroid_5446	5.93E-06
centroid_6892	conserved hypothetical protein	28	42	3	52	centroid_6892	17.900109	2.33E-05	centroid_6892	5.93E-06
centroid_8561	putative transposase	28	42	3	52	centroid_8561	17.900109	2.33E-05	centroid_8561	5.93E-06
centroid_10415	hypothetical protein	25	34	6	60	centroid_10415	16.761112	4.24E-05	centroid_10415	2.08E-05
centroid_16109	putative parB-like partitioning protein	25	34	6	60	centroid_16109	16.761112	4.24E-05	centroid_16109	2.08E-05
centroid_10311	serine/threonine-phosphatase 2 domain protein	24	94	7	0	centroid_10311	18.416367	1.78E-05	centroid_10311	3.30E-05
centroid_1273	voltage gated chloride channel family protein	24	94	7	0	centroid_1273	18.416367	1.78E-05	centroid_1273	3.30E-05
centroid_5247	putative parB-like partitioning protein	24	31	7	63	centroid_5247	16.925192	3.89E-05	centroid_5247	2.01E-05
centroid_6902	conserved hypothetical protein	24	32	7	62	centroid_6902	16.026213	6.25E-05	centroid_6902	4.78E-05
centroid_3011	CTP pyrophosphohydrolase	23	94	8	0	centroid_3011	21.787789	3.05E-06	centroid_3011	6.71E-06
centroid_15442	conserved hypothetical protein	23	93	8	1	centroid_15442	17.816875	2.43E-05	centroid_15442	4.98E-05
centroid_17170	putative two-component-system connector protein AriR	23	93	8	1	centroid_17170	17.816875	2.43E-05	centroid_17170	4.98E-05
centroid_4815	biofilm development YmgB/AriR family protein	23	93	8	1	centroid_4815	17.816875	2.43E-05	centroid_4815	4.98E-05
centroid_11295	fimbrial family protein	22	94	9	0	centroid_11295	25.223071	5.11E-07	centroid_11295	1.32E-06
centroid_1277	EAL domain protein	22	94	9	0	centroid_1277	25.223071	5.11E-07	centroid_1277	1.32E-06
centroid_146	fimbrial family protein	22	94	9	0	centroid_146	25.223071	5.11E-07	centroid_146	1.32E-06
centroid_17105	type VII secretion system (T7SS), usher family protein	22	94	9	0	centroid_17105	25.223071	5.11E-07	centroid_17105	1.32E-06
centroid_17641	conserved hypothetical protein	22	94	9	0	centroid_17641	25.223071	5.11E-07	centroid_17641	1.32E-06
centroid_2664	conserved hypothetical protein	22	94	9	0	centroid_2664	25.223071	5.11E-07	centroid_2664	1.32E-06
centroid_2989	PTS system, cellobiose-specific IIC component	22	94	9	0	centroid_2989	25.223071	5.11E-07	centroid_2989	1.32E-06
centroid_2990	-diacetylchitobiose-specific phosphotransferase enzyme IIB compo	22	94	9	0	centroid_2990	25.223071	5.11E-07	centroid_2990	1.32E-06
centroid_3080	oxygen sensor protein DosP	22	94	9	0	centroid_3080	25.223071	5.11E-07	centroid_3080	1.32E-06

centroid_3180	inner membrane transport protein YeaN	22	94	9	0	centroid_3180	25.223071	5.11E-07	centroid_3180	1.32E-06
centroid_4400	glycine zipper 2TM domain protein	22	94	9	0	centroid_4400	25.223071	5.11E-07	centroid_4400	1.32E-06
centroid_4457	conserved hypothetical protein	22	94	9	0	centroid_4457	25.223071	5.11E-07	centroid_4457	1.32E-06
centroid_4668	haemolysin E family protein	22	94	9	0	centroid_4668	25.223071	5.11E-07	centroid_4668	1.32E-06
centroid_492	taurine ABC transporter, periplasmic binding protein	22	94	9	0	centroid_492	25.223071	5.11E-07	centroid_492	1.32E-06
centroid_5570	haemolysin E family protein	22	94	9	0	centroid_5570	25.223071	5.11E-07	centroid_5570	1.32E-06
centroid_602	glycine zipper 2TM domain protein	22	94	9	0	centroid_602	25.223071	5.11E-07	centroid_602	1.32E-06
centroid_688	transporter, basic amino acid/polyamine antiporter family protein	22	94	9	0	centroid_688	25.223071	5.11E-07	centroid_688	1.32E-06
centroid_689	lysine decarboxylase, inducible	22	94	9	0	centroid_689	25.223071	5.11E-07	centroid_689	1.32E-06
centroid_690	H+ symporter) family protein	22	94	9	0	centroid_690	25.223071	5.11E-07	centroid_690	1.32E-06
centroid_691	lysine--tRNA ligase	22	94	9	0	centroid_691	25.223071	5.11E-07	centroid_691	1.32E-06
centroid_693	conserved hypothetical protein	22	94	9	0	centroid_693	25.223071	5.11E-07	centroid_693	1.32E-06
centroid_7650	type VII secretion system (T7SS), usher family protein	22	94	9	0	centroid_7650	25.223071	5.11E-07	centroid_7650	1.32E-06
centroid_8096	conserved hypothetical protein	22	94	9	0	centroid_8096	25.223071	5.11E-07	centroid_8096	1.32E-06
centroid_8220	major Facilitator Superfamily protein	22	94	9	0	centroid_8220	25.223071	5.11E-07	centroid_8220	1.32E-06
centroid_8446	taurine ABC transporter, periplasmic binding protein	22	94	9	0	centroid_8446	25.223071	5.11E-07	centroid_8446	1.32E-06
centroid_8926	putative pTS system, cellobiose-specific, IIC component	22	94	9	0	centroid_8926	25.223071	5.11E-07	centroid_8926	1.32E-06
centroid_893	fimbrial assembly family protein	22	94	9	0	centroid_893	25.223071	5.11E-07	centroid_893	1.32E-06
centroid_9097	major Facilitator Superfamily protein	22	94	9	0	centroid_9097	25.223071	5.11E-07	centroid_9097	1.32E-06
centroid_9909	fimbrial family protein	22	94	9	0	centroid_9909	25.223071	5.11E-07	centroid_9909	1.32E-06
centroid_9910	conserved hypothetical protein	22	94	9	0	centroid_9910	25.223071	5.11E-07	centroid_9910	1.32E-06
centroid_8121	conserved hypothetical protein	22	20	9	74	centroid_8121	23.621428	1.17E-06	centroid_8121	1.18E-06
centroid_13909	EAL domain protein	22	93	9	1	centroid_13909	21.122029	4.31E-06	centroid_13909	1.09E-05
centroid_13964	sensors of blue-light using FAD family protein	22	93	9	1	centroid_13964	21.122029	4.31E-06	centroid_13964	1.09E-05
centroid_147	fimbrial family protein	22	93	9	1	centroid_147	21.122029	4.31E-06	centroid_147	1.09E-05
centroid_16436	major Facilitator Superfamily protein	22	93	9	1	centroid_16436	21.122029	4.31E-06	centroid_16436	1.09E-05
centroid_1915	HTH-type transcriptional repressor YcgE	22	93	9	1	centroid_1915	21.122029	4.31E-06	centroid_1915	1.09E-05
centroid_2521	inner membrane transport protein YdiM	22	93	9	1	centroid_2521	21.122029	4.31E-06	centroid_2521	1.09E-05
centroid_3182	putative diguanylate cyclase YeaP	22	93	9	1	centroid_3182	21.122029	4.31E-06	centroid_3182	1.09E-05
centroid_687	transcriptional activator CadC	22	93	9	1	centroid_687	21.122029	4.31E-06	centroid_687	1.09E-05
centroid_10816	fimH, mannose binding family protein	22	92	9	2	centroid_10816	17.807199	2.44E-05	centroid_10816	4.98E-05
centroid_17045	conserved hypothetical protein	22	92	9	2	centroid_17045	17.807199	2.44E-05	centroid_17045	4.98E-05
centroid_8893	blue light- and temperature-regulated antirepressor YcgF	22	92	9	2	centroid_8893	17.807199	2.44E-05	centroid_8893	4.98E-05
centroid_9571	fimbrial family protein	22	92	9	2	centroid_9571	17.807199	2.44E-05	centroid_9571	4.98E-05
centroid_13545	hypothetical protein	22	27	9	67	centroid_13545	15.727809	7.31E-05	centroid_13545	4.45E-05
centroid_12405	ABC transporter family protein	21	94	10	0	centroid_12405	28.722145	8.35E-08	centroid_12405	2.50E-07
centroid_1287	helix-turn-helix family protein	21	94	10	0	centroid_1287	28.722145	8.35E-08	centroid_1287	2.50E-07
centroid_1288	outer membrane protein G	21	94	10	0	centroid_1288	28.722145	8.35E-08	centroid_1288	2.50E-07
centroid_1289	ABC transporter family protein	21	94	10	0	centroid_1289	28.722145	8.35E-08	centroid_1289	2.50E-07
centroid_1290	beta-phosphoglucomutase	21	94	10	0	centroid_1290	28.722145	8.35E-08	centroid_1290	2.50E-07
centroid_13072	beta-phosphoglucomutase	21	94	10	0	centroid_13072	28.722145	8.35E-08	centroid_13072	2.50E-07
centroid_17428	haloacid dehalogenase-like hydrolase family protein	21	94	10	0	centroid_17428	28.722145	8.35E-08	centroid_17428	2.50E-07
centroid_1916	conserved hypothetical protein	21	94	10	0	centroid_1916	28.722145	8.35E-08	centroid_1916	2.50E-07
centroid_692	conserved hypothetical protein	21	94	10	0	centroid_692	28.722145	8.35E-08	centroid_692	2.50E-07
centroid_8199	ABC transporter family protein	21	94	10	0	centroid_8199	28.722145	8.35E-08	centroid_8199	2.50E-07
centroid_2311	bacterial regulatory, gntR family protein	21	92	10	2	centroid_2311	21.038206	4.50E-06	centroid_2311	1.15E-05
centroid_2312	D binding domain of 6-phosphogluconate dehydrogenase family prc	21	92	10	2	centroid_2312	21.038206	4.50E-06	centroid_2312	1.15E-05
centroid_2313	conserved hypothetical protein	21	92	10	2	centroid_2313	21.038206	4.50E-06	centroid_2313	1.15E-05
centroid_9791	papC C-terminal domain protein	21	92	10	2	centroid_9791	21.038206	4.50E-06	centroid_9791	1.15E-05
centroid_12243	conserved hypothetical protein	21	22	10	72	centroid_12243	18.390571	1.80E-05	centroid_12243	1.70E-05
centroid_10727	xylose isomerase-like TIM barrel family protein	21	91	10	3	centroid_10727	18.13197	2.06E-05	centroid_10727	4.13E-05
centroid_1291	glycosyl hydrolase family 65 central catalytic domain protein	21	91	10	3	centroid_1291	18.13197	2.06E-05	centroid_1291	4.13E-05
centroid_1292	conserved hypothetical protein	21	91	10	3	centroid_1292	18.13197	2.06E-05	centroid_1292	4.13E-05
centroid_1293	xylose isomerase-like TIM barrel family protein	21	91	10	3	centroid_1293	18.13197	2.06E-05	centroid_1293	4.13E-05
centroid_14310	conserved hypothetical protein	21	91	10	3	centroid_14310	18.13197	2.06E-05	centroid_14310	4.13E-05
centroid_14311	oxidoreductase , NAD-binding Rossmann fold family protein	21	91	10	3	centroid_14311	18.13197	2.06E-05	centroid_14311	4.13E-05
centroid_9336	conserved hypothetical protein	21	91	10	3	centroid_9336	18.13197	2.06E-05	centroid_9336	4.13E-05
centroid_13240	C-5 cytosine-specific DNA methylase family protein	20	6	11	88	centroid_13240	44.359442	2.73E-11	centroid_13240	1.43E-10
centroid_13241	putative r.SnaBI endonuclease	20	6	11	88	centroid_13241	44.359442	2.73E-11	centroid_13241	1.43E-10
centroid_13242	helix-turn-helix family protein	20	6	11	88	centroid_13242	44.359442	2.73E-11	centroid_13242	1.43E-10
centroid_9597	hok/gef family protein	20	10	11	84	centroid_9597	34.205051	4.96E-09	centroid_9597	1.19E-08
centroid_15181	resolvase, N terminal domain protein	20	12	11	82	centroid_15181	30.117834	4.07E-08	centroid_15181	7.06E-08
centroid_16015	resolvase, N terminal domain protein	20	13	11	81	centroid_16015	28.269921	1.06E-07	centroid_16015	1.59E-07
centroid_5993	resolvase, N terminal domain protein	20	13	11	81	centroid_5993	28.269921	1.06E-07	centroid_5993	1.59E-07
centroid_12696	resolvase, N terminal domain protein	20	14	11	80	centroid_12696	26.537407	2.58E-07	centroid_12696	3.43E-07
centroid_13336	shikimate transporter domain protein	20	91	11	3	centroid_13336	21.303599	3.92E-06	centroid_13336	9.78E-06
centroid_5486	putative aminopeptidase	20	91	11	3	centroid_5486	21.303599	3.92E-06	centroid_5486	9.78E-06
centroid_6147	conserved hypothetical protein	20	91	11	3	centroid_6147	21.303599	3.92E-06	centroid_6147	9.78E-06
centroid_17050	putative parB-like nuclease	20	20	11	74	centroid_17050	18.092251	2.10E-05	centroid_17050	1.69E-05
centroid_1247	conserved hypothetical protein	20	89	11	5	centroid_1247	16.397829	5.13E-05	centroid_1247	8.19E-05
centroid_145	type VII secretion system (T7SS), usher family protein	19	93	12	1	centroid_145	31.529718	1.96E-08	centroid_145	8.80E-08
centroid_7651	type VII secretion system (T7SS), usher family protein	19	93	12	1	centroid_7651	31.529718	1.96E-08	centroid_7651	8.80E-08
centroid_15103	hok/gef family protein	19	10	12	84	centroid_15103	30.785262	2.88E-08	centroid_15103	6.39E-08
centroid_15982	glutamate decarboxylase	19	92	12	2	centroid_15982	27.797402	1.35E-07	centroid_15982	5.20E-07
centroid_7219	conserved hypothetical protein	19	92	12	2	centroid_7219	27.797402	1.35E-07	centroid_7219	5.20E-07
centroid_10599	putative predicted inner membrane protein	19	90	12	4	centroid_10599	21.802919	3.02E-06	centroid_10599	7.37E-06
centroid_17355	conserved hypothetical protein	19	90	12	4	centroid_17355	21.802919	3.02E-06	centroid_17355	7.37E-06
centroid_3651	conserved hypothetical protein	19	90	12	4	centroid_3651	21.802919	3.02E-06	centroid_3651	7.37E-06
centroid_5438	conserved hypothetical protein	19	90	12	4	centroid_5438	21.802919	3.02E-06	centroid_5438	7.37E-06
centroid_9698	conserved hypothetical protein	19	16	12	78	centroid_9698	20.518845	5.90E-06	centroid_9698	5.99E-06
centroid_10357	type VI secretion system effector, Hcp1 family protein	19	89	12	5	centroid_10357	19.369022	1.08E-05	centroid_10357	2.11E-05
centroid_3649	conserved hypothetical protein	19	89	12	5	centroid_3649	19.369022	1.08E-05	centroid_3649	2.11E-05
centroid_3650	putative inner membrane protein yafU	19	89	12	5	centroid_3650	19.369022	1.08E-05	centroid_3650	2.11E-05
centroid_10644	phage portal protein, lambda family	19	17	12	77	centroid_10644	19.166966	1.20E-05	centroid_10644	1.52E-05
centroid_16558	type VI secretion system effector, Hcp1 family protein	19	88	12	6	centroid_16558	17.22804	3.32E-05	centroid_16558	5.30E-05
centroid_3721	bacterial regulatory, gntR family protein	19	88	12	6	centroid_3721	17.22804	3.32E-05	centroid_3721	5.30E-05
centroid_4905	putative inner membrane protein	19	88	12	6	centroid_4905	17.22804	3.32E-05	centroid_4905	5.30E-05
centroid_17492	conjugative transfer relaxase protein TraI	19	19	12	75	centroid_17492	16.70039	4.38E-05	centroid_17492	3.66E-05
centroid_7857	putative yceA protein	19	19	12	75	centroid_7857	16.70039	4.38E-05	centroid_7857	3.66E-05
centroid_16045	putative parB-like nuclease	19	20	12	74	centroid_16045	15.574069	7.93E-05	centroid_16045	8.41E-05
centroid_144	gram-negative pili assembly chaperone, N-terminal domain protein	18	93	13	1	centroid_144	35.153824	3.05E-09	centroid_144	1.60E-08
centroid_17423	gram-negative pili assembly chaperone, N-terminal domain protein	18	93	13	1	centroid_17423	35.153824	3.05E-09	centroid_17423	1.60E-08
centroid_3648	type VI secretion system effector, Hcp1 family protein	18	88	13	6	centroid_3648	20.185193	7.03E-06	centroid_3648	1.36E-05
centroid_11141	haemolysin expression modulating family protein	18	15	13	79	centroid_11141	19.160096	1.20E-05	centroid_11141	1.26E-05
centroid_5795	phage terminase large subunit family protein	17	11	14	83	centroid_5795	22.535283	2.06E-06	centroid_5795	3.17E-06
centroid_12699	putative transposase	17	87	14	7	centroid_12699	21.101166	4.36E-06	centroid_12699	8.32E-06
centroid_15371	putative regulatory domain protein	17	12	14	82	centroid_15371	20.858545	4.94E-06	centroid_15371	6.62E-06
centroid_6754	prophage CP4-57 regulatory family protein	17	12	14	82	centroid_6754	20.858545	4.94E-06	centroid_6754	6.62E-06
centroid_17368	conserved hypothetical protein	17	15	14	79	centroid_17368	16.51813	4.82E-05	centroid_17368	6.76E-05
centroid_6095	transposase family protein	17	16	14	78	centroid_6095	15.267491	9.33E-05	centroid_6095	9.65E-05
centroid_17394	hypothetical protein	16	3	15	91	centroid_17394	38.731374	4.86E-10	centroid_17394	3.07E-09
centroid_14499	hypothetical protein	16	4	15	90	centroid_14499	35.457035	2.61E-09	centroid_14499	1.33E-08
centroid_14826	conserved hypothetical protein	16	9	15	85	centroid_14826	23.188165	1.47E-06	centroid_14826	2.70E-06
centroid_9752	conserved hypothetical protein	16	9	15	85	centroid_9752	23.188165	1.47E-06	centroid_9752	2.70E-06
centroid_13672	poly-beta-1,6 N-acetyl-D-glucosamine export porin PgaA	16	85	15	9	centroid_13672	20.203979	6.96E-06	centroid_13672	1.11E-05
centroid_2895	poly-beta-1,6 N-acetyl-D-glucosamine export porin PgaA	16	84	15	10	centroid_2895	18.469565	1.73E-05	centroid_2895	2.36E-05

centroid_4208	transposase, IS605 OrfB family	16	84	15	10	centroid_4208	18.469565	1.73E-05	centroid_4208	2.36E-05
centroid_8226	transposase, IS605 OrfB family	16	84	15	10	centroid_8226	18.469565	1.73E-05	centroid_8226	2.36E-05
centroid_12617	phage-related baseplate assembly family protein	16	12	15	82	centroid_12617	18.065595	2.13E-05	centroid_12617	2.55E-05
centroid_15154	rhs element Vgr family protein	16	12	15	82	centroid_15154	18.065595	2.13E-05	centroid_15154	2.55E-05
centroid_9125	HNH endonuclease family protein	16	12	15	82	centroid_9125	18.065595	2.13E-05	centroid_9125	2.55E-05
centroid_13202	HNH endonuclease family protein	16	13	15	81	centroid_13202	16.617444	4.57E-05	centroid_13202	4.88E-05
centroid_14498	hypothetical protein	15	3	16	91	centroid_14498	35.051412	3.21E-09	centroid_14498	1.74E-08
centroid_14496	hypothetical protein	15	4	16	90	centroid_14496	31.883717	1.64E-08	centroid_14496	7.10E-08
centroid_15928	hypothetical protein	15	4	16	90	centroid_15928	31.883717	1.64E-08	centroid_15928	7.10E-08
centroid_17181	hypothetical protein	15	4	16	90	centroid_17181	31.883717	1.64E-08	centroid_17181	7.10E-08
centroid_9099	dependent phosphotransferase enzyme II for cellobiose domain pr	15	87	16	7	centroid_9099	27.416375	1.64E-07	centroid_9099	4.36E-07
centroid_14606	integron integrase family protein	15	7	16	87	centroid_14606	24.193667	8.71E-07	centroid_14606	1.97E-06
centroid_10579	RHS repeat-associated core domain protein	15	8	16	86	centroid_10579	22.104613	2.58E-06	centroid_10579	4.85E-06
centroid_15117	conserved hypothetical protein	15	9	16	85	centroid_15117	20.203979	6.96E-06	centroid_15117	1.11E-05
centroid_6792	phage tail tape measure protein, lambda family	15	9	16	85	centroid_6792	20.203979	6.96E-06	centroid_6792	1.11E-05
centroid_12632	mu-like prophage major head subunit gpT family protein	15	11	16	83	centroid_12632	16.882606	3.98E-05	centroid_12632	4.75E-05
centroid_5997	tn3 transposase DDE domain protein	15	11	16	83	centroid_5997	16.882606	3.98E-05	centroid_5997	4.75E-05
centroid_13091	mRNA interferase MqsR	15	81	16	13	centroid_13091	16.617444	4.57E-05	centroid_13091	4.88E-05
centroid_2748	mRNA interferase MqsR	15	81	16	13	centroid_2748	16.617444	4.57E-05	centroid_2748	4.88E-05
centroid_13516	putative tail length tape measure domain protein	15	12	16	82	centroid_13516	15.427131	8.57E-05	centroid_13516	9.10E-05
centroid_6535	conserved hypothetical protein	15	12	16	82	centroid_6535	15.427131	8.57E-05	centroid_6535	9.10E-05
centroid_10606	helix-turn-helix domain protein	14	89	17	5	centroid_10606	36.077246	1.90E-09	centroid_10606	8.99E-09
centroid_14495	replication family protein	14	3	17	91	centroid_14495	31.465749	2.03E-08	centroid_14495	9.23E-08
centroid_14640	conserved hypothetical protein	14	3	17	91	centroid_14640	31.465749	2.03E-08	centroid_14640	9.23E-08
centroid_14915	mbeD/MobD like family protein	14	4	17	90	centroid_14915	28.41428	9.79E-08	centroid_14915	3.55E-07
centroid_14916	mbeB-like, N-term conserved region family protein	14	4	17	90	centroid_14916	28.41428	9.79E-08	centroid_14916	3.55E-07
centroid_15941	putative gifsy-1 prophage VtiI	14	5	17	89	centroid_15941	25.701657	3.98E-07	centroid_15941	1.15E-06
centroid_15643	RHS repeat-associated core domain protein	14	6	17	88	centroid_15643	23.277532	1.40E-06	centroid_15643	3.26E-06
centroid_15131	antirestriction family protein	14	7	17	87	centroid_15131	21.101166	4.36E-06	centroid_15131	8.32E-06
centroid_11622	putative dLP12 prophage; DNA base-flipping protein	14	8	17	86	centroid_11622	19.139241	1.22E-05	centroid_11622	1.94E-05
centroid_6120	replication regulatory RepB family protein	14	8	17	86	centroid_6120	19.139241	1.22E-05	centroid_6120	1.94E-05
centroid_5798	conserved hypothetical protein	14	10	17	84	centroid_5798	15.753304	7.22E-05	centroid_5798	8.55E-05
centroid_5799	ATP-binding sugar transporter from pro-phage family protein	14	10	17	84	centroid_5799	15.753304	7.22E-05	centroid_5799	8.55E-05
centroid_11069	conserved hypothetical protein	14	78	17	16	centroid_11069	15.267491	9.33E-05	centroid_11069	9.65E-05
centroid_17514	hsdM N-terminal domain protein	13	2	18	92	centroid_17514	31.314507	2.19E-08	centroid_17514	1.02E-07
centroid_14827	integrase core domain protein	13	3	18	91	centroid_14827	27.97665	1.23E-07	centroid_14827	4.63E-07
centroid_3998	prophage CP4-57 regulatory family protein	13	83	18	11	centroid_3998	25.581151	4.24E-07	centroid_3998	7.27E-07
centroid_12799	transposase family protein	13	4	18	90	centroid_12799	25.052323	5.58E-07	centroid_12799	1.67E-06
centroid_12811	putative tail length tape measure domain protein	13	4	18	90	centroid_12811	25.052323	5.58E-07	centroid_12811	1.67E-06
centroid_13418	integrase core domain protein	13	4	18	90	centroid_13418	25.052323	5.58E-07	centroid_13418	1.67E-06
centroid_14641	DNA relaxase MbeA	13	4	18	90	centroid_14641	25.052323	5.58E-07	centroid_14641	1.67E-06
centroid_15841	endodeoxyribonuclease RusA family protein	13	4	18	90	centroid_15841	25.052323	5.58E-07	centroid_15841	1.67E-06
centroid_9697	antitermination family protein	13	4	18	90	centroid_9697	25.052323	5.58E-07	centroid_9697	1.67E-06
centroid_6210	integrase	13	5	18	89	centroid_6210	22.473156	2.13E-06	centroid_6210	5.08E-06
centroid_5797	clp protease family protein	13	7	18	87	centroid_5797	18.145289	2.05E-05	centroid_5797	3.28E-05
centroid_5800	prophage minor tail Z family protein	13	7	18	87	centroid_5800	18.145289	2.05E-05	centroid_5800	3.28E-05
centroid_12674	phage integrase family protein	13	77	18	17	centroid_12674	16.552634	4.73E-05	centroid_12674	4.76E-05
centroid_5965	CFA/III pilin	13	8	18	86	centroid_5965	16.318535	5.35E-05	centroid_5965	7.25E-05
centroid_6747	lysis S family protein	13	8	18	86	centroid_6747	16.318535	5.35E-05	centroid_6747	7.25E-05
centroid_8682	integrase core domain protein	13	8	18	86	centroid_8682	16.318535	5.35E-05	centroid_8682	7.25E-05
centroid_12314	transposase, IS605 OrfB family	12	86	19	8	centroid_12314	35.294664	2.83E-09	centroid_12314	9.06E-09
centroid_12686	transposase, IS605 OrfB family	12	85	19	9	centroid_12686	32.95534	9.43E-09	centroid_12686	2.49E-08
centroid_13167	conserved hypothetical protein	12	1	19	93	centroid_13167	31.529718	1.96E-08	centroid_13167	8.80E-08
centroid_17716	conserved hypothetical protein	12	1	19	93	centroid_17716	31.529718	1.96E-08	centroid_17716	8.80E-08
centroid_9090	conserved hypothetical protein	12	1	19	93	centroid_9090	31.529718	1.96E-08	centroid_9090	8.80E-08
centroid_10607	transposase, IS605 OrfB family	12	83	19	11	centroid_10607	28.767749	8.16E-08	centroid_10607	1.54E-07
centroid_12299	conserved hypothetical protein	12	2	19	92	centroid_12299	27.797402	1.35E-07	centroid_12299	5.20E-07
centroid_14863	phage tail sheath family protein	12	2	19	92	centroid_14863	27.797402	1.35E-07	centroid_14863	5.20E-07
centroid_6357	type I restriction-modification system, M subunit	12	2	19	92	centroid_6357	27.797402	1.35E-07	centroid_6357	5.20E-07
centroid_11770	conserved hypothetical protein	12	3	19	91	centroid_11770	24.587577	7.10E-07	centroid_11770	2.19E-06
centroid_11972	putative gp55	12	3	19	91	centroid_11972	24.587577	7.10E-07	centroid_11972	2.19E-06
centroid_11974	conserved hypothetical protein	12	3	19	91	centroid_11974	24.587577	7.10E-07	centroid_11974	2.19E-06
centroid_11975	conserved hypothetical protein	12	3	19	91	centroid_11975	24.587577	7.10E-07	centroid_11975	2.19E-06
centroid_11976	conserved hypothetical protein	12	3	19	91	centroid_11976	24.587577	7.10E-07	centroid_11976	2.19E-06
centroid_11984	conserved hypothetical protein	12	3	19	91	centroid_11984	24.587577	7.10E-07	centroid_11984	2.19E-06
centroid_11985	hypothetical protein	12	3	19	91	centroid_11985	24.587577	7.10E-07	centroid_11985	2.19E-06
centroid_14867	conserved hypothetical protein	12	3	19	91	centroid_14867	24.587577	7.10E-07	centroid_14867	2.19E-06
centroid_10382	exc1 domain protein	12	4	19	90	centroid_10382	21.802919	3.02E-06	centroid_10382	7.37E-06
centroid_14497	mobA/MobL family protein	12	4	19	90	centroid_14497	21.802919	3.02E-06	centroid_14497	7.37E-06
centroid_14908	mobA/MobL family protein	12	4	19	90	centroid_14908	21.802919	3.02E-06	centroid_14908	7.37E-06
centroid_15687	helix-turn-helix domain protein	12	4	19	90	centroid_15687	21.802919	3.02E-06	centroid_15687	7.37E-06
centroid_8523	cytoskeleton-binding toxin CbtA	12	4	19	90	centroid_8523	21.802919	3.02E-06	centroid_8523	7.37E-06
centroid_17627	fimbrial subunit ElfA	12	78	19	16	centroid_17627	20.518845	5.90E-06	centroid_17627	5.99E-06
centroid_10383	regulatory protein rop	12	5	19	89	centroid_10383	19.369022	1.08E-05	centroid_10383	2.11E-05
centroid_3637	hok/gef family protein	12	77	19	17	centroid_3637	19.166966	1.20E-05	centroid_3637	1.52E-05
centroid_10197	phage P2 GpU family protein	12	76	19	18	centroid_10197	17.896192	2.33E-05	centroid_10197	2.26E-05
centroid_16146	conserved hypothetical protein	12	76	19	18	centroid_16146	17.896192	2.33E-05	centroid_16146	2.26E-05
centroid_7095	phage P2 GpU family protein	12	76	19	18	centroid_7095	17.896192	2.33E-05	centroid_7095	2.26E-05
centroid_7450	conserved hypothetical protein	12	6	19	88	centroid_7450	17.22804	3.32E-05	centroid_7450	5.30E-05
centroid_3643	phage P2 GpU family protein	12	74	19	20	centroid_3643	15.574069	7.93E-05	centroid_3643	8.41E-05
centroid_13385	phage tail tape measure protein, lambda family	12	7	19	87	centroid_13385	15.334326	9.01E-05	centroid_13385	0.0001206
centroid_13425	putative phage domain protein	12	7	19	87	centroid_13425	15.334326	9.01E-05	centroid_13425	0.0001206
centroid_15710	conserved hypothetical protein	12	7	19	87	centroid_15710	15.334326	9.01E-05	centroid_15710	0.0001206
centroid_7452	phage stabilisation family protein	12	7	19	87	centroid_7452	15.334326	9.01E-05	centroid_7452	0.0001206
centroid_9750	putative yheA protein	12	7	19	87	centroid_9750	15.334326	9.01E-05	centroid_9750	0.0001206
centroid_9751	putative phage protein	12	7	19	87	centroid_9751	15.334326	9.01E-05	centroid_9751	0.0001206
centroid_15183	type-2 restriction enzyme Cfr10I	11	0	20	94	centroid_15183	32.285576	1.33E-08	centroid_15183	4.57E-08
centroid_15226	integrase	11	0	20	94	centroid_15226	32.285576	1.33E-08	centroid_15226	4.57E-08
centroid_15228	DEAD/DEAH box helicase family protein	11	0	20	94	centroid_15228	32.285576	1.33E-08	centroid_15228	4.57E-08
centroid_15229	conserved hypothetical protein	11	0	20	94	centroid_15229	32.285576	1.33E-08	centroid_15229	4.57E-08
centroid_15363	phage gp6-like head-tail connector family protein	11	0	20	94	centroid_15363	32.285576	1.33E-08	centroid_15363	4.57E-08
centroid_15364	putative head-tail adaptor	11	0	20	94	centroid_15364	32.285576	1.33E-08	centroid_15364	4.57E-08
centroid_15372	phage portal protein, HK97 family	11	0	20	94	centroid_15372	32.285576	1.33E-08	centroid_15372	4.57E-08
centroid_15373	phage prohead protease, HK97 family	11	0	20	94	centroid_15373	32.285576	1.33E-08	centroid_15373	4.57E-08
centroid_15374	phage major capsid protein, HK97 family	11	0	20	94	centroid_15374	32.285576	1.33E-08	centroid_15374	4.57E-08
centroid_15547	bacteriophage lysis family protein	11	0	20	94	centroid_15547	32.285576	1.33E-08	centroid_15547	4.57E-08
centroid_15874	DEAD/DEAH box helicase family protein	11	0	20	94	centroid_15874	32.285576	1.33E-08	centroid_15874	4.57E-08
centroid_16784	putative dead/deah box helicase domain protein	11	0	20	94	centroid_16784	32.285576	1.33E-08	centroid_16784	4.57E-08
centroid_11498	conserved hypothetical protein	11	81	20	13	centroid_11498	28.269921	1.06E-07	centroid_11498	1.59E-07
centroid_11732	HNH endonuclease family protein	11	1	20	93	centroid_11732	27.981976	1.22E-07	centroid_11732	4.60E-07
centroid_11739	conserved hypothetical protein	11	1	20	93	centroid_11739	27.981976	1.22E-07	centroid_11739	4.60E-07
centroid_11740	conserved hypothetical protein	11	1	20	93	centroid_11740	27.981976	1.22E-07	centroid_11740	4.60E-07
centroid_11741	immunoglobulin domain protein	11	1	20	93	centroid_11741	27.981976	1.22E-07	centroid_11741	4.60E-07
centroid_11742	phage tail assembly chaperone family protein	11	1	20	93	centroid_11742	27.981976	1.22E-07	centroid_11742	4.60E-07
centroid_11743	conserved hypothetical protein	11	1	20	93	centroid_11743	27.981976	1.22E-07	centroid_11743	4.60E-07
centroid_11744	phage tail tape measure protein, lambda family	11	1	20	93	centroid_11744	27.981976	1.22E-07	centroid_11744	4.60E-07

centroid_11745	phage minor tail family protein	11	1	20	93	centroid_11745	27.981976	1.22E-07	centroid_11745	4.60E-07
centroid_12067	methyltransferase domain protein	11	1	20	93	centroid_12067	27.981976	1.22E-07	centroid_12067	4.60E-07
centroid_13168	conserved hypothetical protein	11	1	20	93	centroid_13168	27.981976	1.22E-07	centroid_13168	4.60E-07
centroid_14642	mobilization protein MbeC	11	1	20	93	centroid_14642	27.981976	1.22E-07	centroid_14642	4.60E-07
centroid_15976	conserved hypothetical protein	11	1	20	93	centroid_15976	27.981976	1.22E-07	centroid_15976	4.60E-07
centroid_16037	conserved hypothetical protein	11	1	20	93	centroid_16037	27.981976	1.22E-07	centroid_16037	4.60E-07
centroid_6658	HNH endonuclease family protein	11	1	20	93	centroid_6658	27.981976	1.22E-07	centroid_6658	4.60E-07
centroid_6659	sensory box protein	11	1	20	93	centroid_6659	27.981976	1.22E-07	centroid_6659	4.60E-07
centroid_6699	bacterial regulatory, tetR family protein	11	1	20	93	centroid_6699	27.981976	1.22E-07	centroid_6699	4.60E-07
centroid_14864	conserved hypothetical protein	11	2	20	92	centroid_14864	24.370503	7.95E-07	centroid_14864	2.51E-06
centroid_14865	putative lipoprotein	11	2	20	92	centroid_14865	24.370503	7.95E-07	centroid_14865	2.51E-06
centroid_14868	DNA adenine methylase family protein	11	2	20	92	centroid_14868	24.370503	7.95E-07	centroid_14868	2.51E-06
centroid_5793	conserved hypothetical protein	11	2	20	92	centroid_5793	24.370503	7.95E-07	centroid_5793	2.51E-06
centroid_10205	phage late control gene D family protein	11	77	20	17	centroid_10205	21.940781	2.81E-06	centroid_10205	2.72E-06
centroid_11465	hypothetical protein	11	77	20	17	centroid_11465	21.940781	2.81E-06	centroid_11465	2.72E-06
centroid_13559	putative phage tail tape measure domain protein	11	77	20	17	centroid_13559	21.940781	2.81E-06	centroid_13559	2.72E-06
centroid_10877	tail needle protein gp26	11	3	20	91	centroid_10877	21.303599	3.92E-06	centroid_10877	9.78E-06
centroid_11980	putative gp16	11	3	20	91	centroid_11980	21.303599	3.92E-06	centroid_11980	9.78E-06
centroid_11981	marR family protein	11	3	20	91	centroid_11981	21.303599	3.92E-06	centroid_11981	9.78E-06
centroid_5998	putative transposase domain protein	11	3	20	91	centroid_5998	21.303599	3.92E-06	centroid_5998	9.78E-06
centroid_3638	ogr/Delta-like zinc finger family protein	11	76	20	18	centroid_3638	20.583251	5.71E-06	centroid_3638	6.78E-06
centroid_14456	conserved hypothetical protein	11	4	20	90	centroid_14456	18.673079	1.55E-05	centroid_14456	3.07E-05
centroid_15341	conserved hypothetical protein	11	4	20	90	centroid_15341	18.673079	1.55E-05	centroid_15341	3.07E-05
centroid_15762	bacterial regulatory , Fis family protein	11	4	20	90	centroid_15762	18.673079	1.55E-05	centroid_15762	3.07E-05
centroid_15820	ead/Ea22-like family protein	11	4	20	90	centroid_15820	18.673079	1.55E-05	centroid_15820	3.07E-05
centroid_17401	pentapeptide repeats family protein	11	4	20	90	centroid_17401	18.673079	1.55E-05	centroid_17401	3.07E-05
centroid_6005	CFA/I fimbrial subunit D	11	4	20	90	centroid_6005	18.673079	1.55E-05	centroid_6005	3.07E-05
centroid_17429	conserved hypothetical protein	11	73	20	21	centroid_17429	16.948326	3.84E-05	centroid_17429	3.87E-05
centroid_10378	hypothetical protein	11	5	20	89	centroid_10378	16.397829	5.13E-05	centroid_10378	8.19E-05
centroid_10379	conserved hypothetical protein	11	5	20	89	centroid_10379	16.397829	5.13E-05	centroid_10379	8.19E-05
centroid_10380	conserved hypothetical protein	11	5	20	89	centroid_10380	16.397829	5.13E-05	centroid_10380	8.19E-05
centroid_10602	hypothetical protein	11	5	20	89	centroid_10602	16.397829	5.13E-05	centroid_10602	8.19E-05
centroid_10886	conserved hypothetical protein	11	5	20	89	centroid_10886	16.397829	5.13E-05	centroid_10886	8.19E-05
centroid_11463	conserved hypothetical protein	11	5	20	89	centroid_11463	16.397829	5.13E-05	centroid_11463	8.19E-05
centroid_15173	transposase family protein	11	5	20	89	centroid_15173	16.397829	5.13E-05	centroid_15173	8.19E-05
centroid_6060	putative transposase	11	5	20	89	centroid_6060	16.397829	5.13E-05	centroid_6060	8.19E-05
centroid_14954	conserved hypothetical protein	10	0	21	94	centroid_14954	28.722145	8.35E-08	centroid_14954	2.50E-07
centroid_15227	putative dead/deah box helicase domain protein	10	0	21	94	centroid_15227	28.722145	8.35E-08	centroid_15227	2.50E-07
centroid_15281	phage tail tape measure protein, lambda family	10	0	21	94	centroid_15281	28.722145	8.35E-08	centroid_15281	2.50E-07
centroid_15286	conserved hypothetical protein	10	0	21	94	centroid_15286	28.722145	8.35E-08	centroid_15286	2.50E-07
centroid_15313	type I restriction enzyme, S subunit domain protein	10	0	21	94	centroid_15313	28.722145	8.35E-08	centroid_15313	2.50E-07
centroid_15314	putative membrane protein	10	0	21	94	centroid_15314	28.722145	8.35E-08	centroid_15314	2.50E-07
centroid_15322	polysaccharide biosynthesis/export family protein	10	0	21	94	centroid_15322	28.722145	8.35E-08	centroid_15322	2.50E-07
centroid_15323	ABC-2 type transporter family protein	10	0	21	94	centroid_15323	28.722145	8.35E-08	centroid_15323	2.50E-07
centroid_15324	ABC transporter family protein	10	0	21	94	centroid_15324	28.722145	8.35E-08	centroid_15324	2.50E-07
centroid_15325	lysaccharide export inner-membrane , BexC/CtrB/KpsE family prot	10	0	21	94	centroid_15325	28.722145	8.35E-08	centroid_15325	2.50E-07
centroid_15326	methyltransferase domain protein	10	0	21	94	centroid_15326	28.722145	8.35E-08	centroid_15326	2.50E-07
centroid_15327	glycosyltransferase WbsX family protein	10	0	21	94	centroid_15327	28.722145	8.35E-08	centroid_15327	2.50E-07
centroid_15328	glycosyl transferase 2 family protein	10	0	21	94	centroid_15328	28.722145	8.35E-08	centroid_15328	2.50E-07
centroid_15329	nucleotide sugar dehydrogenase family protein	10	0	21	94	centroid_15329	28.722145	8.35E-08	centroid_15329	2.50E-07
centroid_15330	hypothetical protein	10	0	21	94	centroid_15330	28.722145	8.35E-08	centroid_15330	2.50E-07
centroid_15331	glycosyl transferase 2 family protein	10	0	21	94	centroid_15331	28.722145	8.35E-08	centroid_15331	2.50E-07
centroid_15332	glycosyl transferase 2 family protein	10	0	21	94	centroid_15332	28.722145	8.35E-08	centroid_15332	2.50E-07
centroid_15333	glycosyl transferases group 1 family protein	10	0	21	94	centroid_15333	28.722145	8.35E-08	centroid_15333	2.50E-07
centroid_15334	dTDP-glucose 4,6-dehydratase	10	0	21	94	centroid_15334	28.722145	8.35E-08	centroid_15334	2.50E-07
centroid_15335	dTDP-4-dehydrorhamnose reductase	10	0	21	94	centroid_15335	28.722145	8.35E-08	centroid_15335	2.50E-07
centroid_15336	glucose-1-phosphate thymidylyltransferase	10	0	21	94	centroid_15336	28.722145	8.35E-08	centroid_15336	2.50E-07
centroid_15337	dTDP-4-dehydrorhamnose 3,5-epimerase	10	0	21	94	centroid_15337	28.722145	8.35E-08	centroid_15337	2.50E-07
centroid_15338	capsule polysaccharide biosynthesis family protein	10	0	21	94	centroid_15338	28.722145	8.35E-08	centroid_15338	2.50E-07
centroid_15339	capsule polysaccharide biosynthesis family protein	10	0	21	94	centroid_15339	28.722145	8.35E-08	centroid_15339	2.50E-07
centroid_15340	putative pH01	10	0	21	94	centroid_15340	28.722145	8.35E-08	centroid_15340	2.50E-07
centroid_15347	DNA transfer protein gp7	10	0	21	94	centroid_15347	28.722145	8.35E-08	centroid_15347	2.50E-07
centroid_15353	type I restriction modification DNA specificity domain protein	10	0	21	94	centroid_15353	28.722145	8.35E-08	centroid_15353	2.50E-07
centroid_15354	yecA family protein	10	0	21	94	centroid_15354	28.722145	8.35E-08	centroid_15354	2.50E-07
centroid_15355	'Cold-shock' DNA-binding domain protein	10	0	21	94	centroid_15355	28.722145	8.35E-08	centroid_15355	2.50E-07
centroid_15392	putative outer membrane lipoprotein SlyB	10	0	21	94	centroid_15392	28.722145	8.35E-08	centroid_15392	2.50E-07
centroid_15789	adenine-specific methyltransferase EcoRI family protein	10	0	21	94	centroid_15789	28.722145	8.35E-08	centroid_15789	2.50E-07
centroid_15790	type-2 restriction enzyme EcoRI	10	0	21	94	centroid_15790	28.722145	8.35E-08	centroid_15790	2.50E-07
centroid_15882	tail spike protein	10	0	21	94	centroid_15882	28.722145	8.35E-08	centroid_15882	2.50E-07
centroid_17652	conserved hypothetical protein	10	0	21	94	centroid_17652	28.722145	8.35E-08	centroid_17652	2.50E-07
centroid_13199	phage late control gene D family protein	10	77	21	17	centroid_13199	24.871589	6.13E-07	centroid_13199	6.06E-07
centroid_8076	glutamate decarboxylase	10	77	21	17	centroid_8076	24.871589	6.13E-07	centroid_8076	6.06E-07
centroid_12069	ncrBC 5-methylcytosine restriction system component family protei	10	1	21	93	centroid_12069	24.511893	7.39E-07	centroid_12069	2.29E-06
centroid_12907	putative transposase	10	1	21	93	centroid_12907	24.511893	7.39E-07	centroid_12907	2.29E-06
centroid_15239	integrase core domain protein	10	1	21	93	centroid_15239	24.511893	7.39E-07	centroid_15239	2.29E-06
centroid_15272	dTDP-4-dehydrorhamnose 3,5-epimerase	10	1	21	93	centroid_15272	24.511893	7.39E-07	centroid_15272	2.29E-06
centroid_15273	glycosyl transferase 2 family protein	10	1	21	93	centroid_15273	24.511893	7.39E-07	centroid_15273	2.29E-06
centroid_15274	glycosyl transferases group 1 family protein	10	1	21	93	centroid_15274	24.511893	7.39E-07	centroid_15274	2.29E-06
centroid_15275	putative membrane protein	10	1	21	93	centroid_15275	24.511893	7.39E-07	centroid_15275	2.29E-06
centroid_15276	bacterial transferase hexapeptide family protein	10	1	21	93	centroid_15276	24.511893	7.39E-07	centroid_15276	2.29E-06
centroid_15277	UDP-N-acetylglucosamine 4,6-dehydratase/5-epimerase	10	1	21	93	centroid_15277	24.511893	7.39E-07	centroid_15277	2.29E-06
centroid_15738	putative mobilization protein 1	10	1	21	93	centroid_15738	24.511893	7.39E-07	centroid_15738	2.29E-06
centroid_16486	AAA domain family protein	10	1	21	93	centroid_16486	24.511893	7.39E-07	centroid_16486	2.29E-06
centroid_17513	methyltransferase domain protein	10	1	21	93	centroid_17513	24.511893	7.39E-07	centroid_17513	2.29E-06
centroid_18276	polysaccharide biosynthesis family protein	10	1	21	93	centroid_18276	24.511893	7.39E-07	centroid_18276	2.29E-06
centroid_6660	coA-transferase III family protein	10	1	21	93	centroid_6660	24.511893	7.39E-07	centroid_6660	2.29E-06
centroid_6661	HMGL-like family protein	10	1	21	93	centroid_6661	24.511893	7.39E-07	centroid_6661	2.29E-06
centroid_6662	sugar (and other) transporter family protein	10	1	21	93	centroid_6662	24.511893	7.39E-07	centroid_6662	2.29E-06
centroid_8929	integrase core domain protein	10	1	21	93	centroid_8929	24.511893	7.39E-07	centroid_8929	2.29E-06
centroid_9093	ead/Ea22-like family protein	10	1	21	93	centroid_9093	24.511893	7.39E-07	centroid_9093	2.29E-06
centroid_16096	phage late control gene D family protein	10	76	21	18	centroid_16096	23.430088	1.30E-06	centroid_16096	1.17E-06
centroid_18677	phage tail sheath family protein	10	76	21	18	centroid_18677	23.430088	1.30E-06	centroid_18677	1.17E-06
centroid_7906	phage late control gene D family protein	10	76	21	18	centroid_7906	23.430088	1.30E-06	centroid_7906	1.17E-06
centroid_10219	phage tail sheath family protein	10	75	21	19	centroid_10219	22.066472	2.63E-06	centroid_10219	2.90E-06
centroid_10421	conserved predicted domain protein	10	75	21	19	centroid_10421	22.066472	2.63E-06	centroid_10421	2.90E-06
centroid_8326	putative conserved predicted protein	10	75	21	19	centroid_8326	22.066472	2.63E-06	centroid_8326	2.90E-06
centroid_11693	bacteriophage lysis family protein	10	2	21	92	centroid_11693	21.038206	4.50E-06	centroid_11693	1.15E-05
centroid_11872	cupin fold metallo, WbuC family protein	10	2	21	92	centroid_11872	21.038206	4.50E-06	centroid_11872	1.15E-05
centroid_11873	glycosyl transferases group 1 family protein	10	2	21	92	centroid_11873	21.038206	4.50E-06	centroid_11873	1.15E-05
centroid_11874	UDP-N-acetylglucosamine 2-epimerase	10	2	21	92	centroid_11874	21.038206	4.50E-06	centroid_11874	1.15E-05
centroid_11875	NAD dependent epimerase/dehydratase family protein	10	2	21	92	centroid_11875	21.038206	4.50E-06	centroid_11875	1.15E-05
centroid_11877	glycosyl transferases group 1 family protein	10	2	21	92	centroid_11877	21.038206	4.50E-06	centroid_11877	1.15E-05
centroid_15906	N-6 DNA Methylase family protein	10	2	21	92	centroid_15906	21.038206	4.50E-06	centroid_15906	1.15E-05
centroid_18675	putative variable tail fiber protein	10	2	21	92	centroid_18675	21.038206	4.50E-06	centroid_18675	1.15E-05
centroid_5615	resolvase, N terminal domain protein	10	2	21	92	centroid_5615	21.038206	4.50E-06	centroid_5615	1.15E-05
centroid_6014	conserved hypothetical protein	10	2	21	92	centroid_6014	21.038206	4.50E-06	centroid_6014	1.15E-05

centroid_6015	conserved hypothetical protein	10	2	21	92	centroid_6015	21.038206	4.50E-06	centroid_6015	1.15E-05
centroid_6016	conserved hypothetical protein	10	2	21	92	centroid_6016	21.038206	4.50E-06	centroid_6016	1.15E-05
centroid_6017	conserved hypothetical protein	10	2	21	92	centroid_6017	21.038206	4.50E-06	centroid_6017	1.15E-05
centroid_6018	putative translation elongation factor P domain protein	10	2	21	92	centroid_6018	21.038206	4.50E-06	centroid_6018	1.15E-05
centroid_6019	conserved hypothetical protein	10	2	21	92	centroid_6019	21.038206	4.50E-06	centroid_6019	1.15E-05
centroid_3640	phage tail sheath family protein	10	74	21	20	centroid_3640	20.775245	5.16E-06	centroid_3640	4.45E-06
centroid_3835	putative conserved predicted protein	10	74	21	20	centroid_3835	20.775245	5.16E-06	centroid_3835	4.45E-06
centroid_13223	conserved hypothetical protein	10	3	21	91	centroid_13223	18.13197	2.06E-05	centroid_13223	4.13E-05
centroid_14810	transposase IS116/IS110/IS902 family protein	10	3	21	91	centroid_14810	18.13197	2.06E-05	centroid_14810	4.13E-05
centroid_15411	protein CexE	10	3	21	91	centroid_15411	18.13197	2.06E-05	centroid_15411	4.13E-05
centroid_15980	phage terminase, large subunit, PBSX family	10	3	21	91	centroid_15980	18.13197	2.06E-05	centroid_15980	4.13E-05
centroid_17015	gram-negative pili assembly chaperone, N-terminal domain protein	10	3	21	91	centroid_17015	18.13197	2.06E-05	centroid_17015	4.13E-05
centroid_18633	putative transposase	10	3	21	91	centroid_18633	18.13197	2.06E-05	centroid_18633	4.13E-05
centroid_5999	transposase family protein	10	3	21	91	centroid_5999	18.13197	2.06E-05	centroid_5999	4.13E-05
centroid_6000	ABC transporter family protein	10	3	21	91	centroid_6000	18.13197	2.06E-05	centroid_6000	4.13E-05
centroid_6001	putative transporter protein AatB	10	3	21	91	centroid_6001	18.13197	2.06E-05	centroid_6001	4.13E-05
centroid_6002	outer membrane efflux family protein	10	3	21	91	centroid_6002	18.13197	2.06E-05	centroid_6002	4.13E-05
centroid_6003	permease family protein	10	3	21	91	centroid_6003	18.13197	2.06E-05	centroid_6003	4.13E-05
centroid_6059	serine protease SepA autotransporter	10	3	21	91	centroid_6059	18.13197	2.06E-05	centroid_6059	4.13E-05
centroid_6062	type VII secretion system (T7SS), usher family protein	10	3	21	91	centroid_6062	18.13197	2.06E-05	centroid_6062	4.13E-05
centroid_6066	putative membrane protein	10	3	21	91	centroid_6066	18.13197	2.06E-05	centroid_6066	4.13E-05
centroid_6068	hypothetical protein	10	3	21	91	centroid_6068	18.13197	2.06E-05	centroid_6068	4.13E-05
centroid_6894	integrase core domain protein	10	3	21	91	centroid_6894	18.13197	2.06E-05	centroid_6894	4.13E-05
centroid_9784	ead/Ea22-like family protein	10	3	21	91	centroid_9784	18.13197	2.06E-05	centroid_9784	4.13E-05
centroid_7843	phage tail tape measure protein, TP901 family, core region	10	71	21	23	centroid_7843	17.288561	3.21E-05	centroid_7843	2.40E-05
centroid_10381	homeo-like domain protein	10	4	21	90	centroid_10381	15.672416	7.53E-05	centroid_10381	0.0001202
centroid_12479	conserved hypothetical protein	10	4	21	90	centroid_12479	15.672416	7.53E-05	centroid_12479	0.0001202
centroid_15760	conserved hypothetical protein	10	4	21	90	centroid_15760	15.672416	7.53E-05	centroid_15760	0.0001202
centroid_15761	qnrB1	10	4	21	90	centroid_15761	15.672416	7.53E-05	centroid_15761	0.0001202
centroid_16135	hypothetical protein	10	4	21	90	centroid_16135	15.672416	7.53E-05	centroid_16135	0.0001202
centroid_18634	type VII secretion system (T7SS), usher family protein	10	4	21	90	centroid_18634	15.672416	7.53E-05	centroid_18634	0.0001202
centroid_6061	SEF14-like adhesin family protein	10	4	21	90	centroid_6061	15.672416	7.53E-05	centroid_6061	0.0001202
centroid_6067	hypothetical protein	10	4	21	90	centroid_6067	15.672416	7.53E-05	centroid_6067	0.0001202
centroid_13561	phage integrase family protein	10	69	21	25	centroid_13561	15.24665	9.43E-05	centroid_13561	7.40E-05
centroid_15218	prophage tail fibre N-terminal family protein	9	0	22	94	centroid_15218	25.223071	5.11E-07	centroid_15218	1.32E-06
centroid_15223	conserved hypothetical protein	9	0	22	94	centroid_15223	25.223071	5.11E-07	centroid_15223	1.32E-06
centroid_15224	putative transcriptional regulator	9	0	22	94	centroid_15224	25.223071	5.11E-07	centroid_15224	1.32E-06
centroid_15231	lysR family regulatory helix-turn-helix protein	9	0	22	94	centroid_15231	25.223071	5.11E-07	centroid_15231	1.32E-06
centroid_15262	hypothetical protein	9	0	22	94	centroid_15262	25.223071	5.11E-07	centroid_15262	1.32E-06
centroid_15285	conserved hypothetical protein	9	0	22	94	centroid_15285	25.223071	5.11E-07	centroid_15285	1.32E-06
centroid_15357	hypothetical protein	9	0	22	94	centroid_15357	25.223071	5.11E-07	centroid_15357	1.32E-06
centroid_15365	phage minor tail protein L	9	0	22	94	centroid_15365	25.223071	5.11E-07	centroid_15365	1.32E-06
centroid_15376	conserved hypothetical protein	9	0	22	94	centroid_15376	25.223071	5.11E-07	centroid_15376	1.32E-06
centroid_15377	P63C domain protein	9	0	22	94	centroid_15377	25.223071	5.11E-07	centroid_15377	1.32E-06
centroid_15379	DNA transfer gp20 domain protein	9	0	22	94	centroid_15379	25.223071	5.11E-07	centroid_15379	1.32E-06
centroid_3639	phage late control gene D family protein	9	73	22	21	centroid_3639	22.320102	2.31E-06	centroid_3639	1.82E-06
centroid_11855	ninB family protein	9	1	22	93	centroid_11855	21.122029	4.31E-06	centroid_11855	1.09E-05
centroid_12068	ATPase associated with various cellular activities family protein	9	1	22	93	centroid_12068	21.122029	4.31E-06	centroid_12068	1.09E-05
centroid_12109	conserved hypothetical protein	9	1	22	93	centroid_12109	21.122029	4.31E-06	centroid_12109	1.09E-05
centroid_13971	conserved hypothetical protein	9	1	22	93	centroid_13971	21.122029	4.31E-06	centroid_13971	1.09E-05
centroid_13972	ERF superfamily protein	9	1	22	93	centroid_13972	21.122029	4.31E-06	centroid_13972	1.09E-05
centroid_13980	dnaB-like helicase C terminal domain protein	9	1	22	93	centroid_13980	21.122029	4.31E-06	centroid_13980	1.09E-05
centroid_15260	phage tail fibre repeat family protein	9	1	22	93	centroid_15260	21.122029	4.31E-06	centroid_15260	1.09E-05
centroid_15381	putative transposase	9	1	22	93	centroid_15381	21.122029	4.31E-06	centroid_15381	1.09E-05
centroid_15548	hypothetical protein	9	1	22	93	centroid_15548	21.122029	4.31E-06	centroid_15548	1.09E-05
centroid_17479	putative yacA	9	1	22	93	centroid_17479	21.122029	4.31E-06	centroid_17479	1.09E-05
centroid_17625	conserved hypothetical protein	9	1	22	93	centroid_17625	21.122029	4.31E-06	centroid_17625	1.09E-05
centroid_13238	phage tail tube FII family protein	9	72	22	22	centroid_13238	21.082915	4.40E-06	centroid_13238	3.06E-06
centroid_10882	phage terminase, large subunit, PBSX family	9	2	22	92	centroid_10882	17.807199	2.44E-05	centroid_10882	4.98E-05
centroid_14636	conserved hypothetical protein	9	2	22	92	centroid_14636	17.807199	2.44E-05	centroid_14636	4.98E-05
centroid_14649	conserved hypothetical protein	9	2	22	92	centroid_14649	17.807199	2.44E-05	centroid_14649	4.98E-05
centroid_16817	prophage CP4-57 regulatory family protein	9	2	22	92	centroid_16817	17.807199	2.44E-05	centroid_16817	4.98E-05
centroid_17573	nucleotidyltransferase domain protein	9	2	22	92	centroid_17573	17.807199	2.44E-05	centroid_17573	4.98E-05
centroid_5614	hypothetical protein	9	2	22	92	centroid_5614	17.807199	2.44E-05	centroid_5614	4.98E-05
centroid_6012	conserved hypothetical protein	9	2	22	92	centroid_6012	17.807199	2.44E-05	centroid_6012	4.98E-05
centroid_6013	conserved hypothetical protein	9	2	22	92	centroid_6013	17.807199	2.44E-05	centroid_6013	4.98E-05
centroid_11068	conserved hypothetical protein	9	67	22	27	centroid_11068	15.727809	7.31E-05	centroid_11068	4.45E-05
centroid_3641	phage major tail tube protein	8	72	23	22	centroid_3641	23.942203	9.93E-07	centroid_3641	6.96E-07
centroid_14812	transposase IS66 family protein	8	0	23	94	centroid_14812	21.787789	3.05E-06	centroid_14812	6.71E-06
centroid_15184	DNA (cytosine-5-)-methyltransferase family protein	8	0	23	94	centroid_15184	21.787789	3.05E-06	centroid_15184	6.71E-06
centroid_15225	phage integrase family protein	8	0	23	94	centroid_15225	21.787789	3.05E-06	centroid_15225	6.71E-06
centroid_15375	conserved hypothetical protein	8	0	23	94	centroid_15375	21.787789	3.05E-06	centroid_15375	6.71E-06
centroid_15883	putative dNA transfer protein gp20	8	0	23	94	centroid_15883	21.787789	3.05E-06	centroid_15883	6.71E-06
centroid_15929	phage tail fibre repeat family protein	8	0	23	94	centroid_15929	21.787789	3.05E-06	centroid_15929	6.71E-06
centroid_15961	conserved hypothetical protein	8	0	23	94	centroid_15961	21.787789	3.05E-06	centroid_15961	6.71E-06
centroid_15968	conserved hypothetical protein	8	0	23	94	centroid_15968	21.787789	3.05E-06	centroid_15968	6.71E-06
centroid_11979	helix-turn-helix family protein	8	1	23	93	centroid_11979	17.816875	2.43E-05	centroid_11979	4.98E-05
centroid_11982	conserved hypothetical protein	8	1	23	93	centroid_11982	17.816875	2.43E-05	centroid_11982	4.98E-05
centroid_13974	conserved hypothetical protein	8	1	23	93	centroid_13974	17.816875	2.43E-05	centroid_13974	4.98E-05
centroid_15123	conserved hypothetical protein	8	1	23	93	centroid_15123	17.816875	2.43E-05	centroid_15123	4.98E-05
centroid_15893	peptidase S24-like family protein	8	1	23	93	centroid_15893	17.816875	2.43E-05	centroid_15893	4.98E-05
centroid_15923	dihydrofolate reductase type 1	8	1	23	93	centroid_15923	17.816875	2.43E-05	centroid_15923	4.98E-05
centroid_6230	helix-turn-helix domain protein	8	1	23	93	centroid_6230	17.816875	2.43E-05	centroid_6230	4.98E-05
centroid_6231	hypothetical protein	8	1	23	93	centroid_6231	17.816875	2.43E-05	centroid_6231	4.98E-05
centroid_10126	conserved hypothetical protein	8	66	23	28	centroid_10126	17.238021	3.30E-05	centroid_10126	1.83E-05
centroid_5033	conserved hypothetical protein	8	66	23	28	centroid_5033	17.238021	3.30E-05	centroid_5033	1.83E-05
centroid_4244	conserved hypothetical protein	7	77	24	17	centroid_4244	34.591386	4.07E-09	centroid_4244	3.47E-09
centroid_5227	conserved hypothetical protein	7	76	24	18	centroid_5227	32.915024	9.63E-09	centroid_5227	7.45E-09
centroid_14501	regulatory protein rop	7	0	24	94	centroid_14501	18.416367	1.78E-05	centroid_14501	3.30E-05
centroid_15880	conserved hypothetical protein	7	0	24	94	centroid_15880	18.416367	1.78E-05	centroid_15880	3.30E-05
centroid_15889	putative membrane protein	7	0	24	94	centroid_15889	18.416367	1.78E-05	centroid_15889	3.30E-05
centroid_15890	conserved hypothetical protein	7	0	24	94	centroid_15890	18.416367	1.78E-05	centroid_15890	3.30E-05
centroid_15891	bacteriophage replication O family protein	7	0	24	94	centroid_15891	18.416367	1.78E-05	centroid_15891	3.30E-05
centroid_15892	conserved hypothetical protein	7	0	24	94	centroid_15892	18.416367	1.78E-05	centroid_15892	3.30E-05
centroid_15895	conserved hypothetical protein	7	0	24	94	centroid_15895	18.416367	1.78E-05	centroid_15895	3.30E-05
centroid_15896	ead/Ea22-like family protein	7	0	24	94	centroid_15896	18.416367	1.78E-05	centroid_15896	3.30E-05
centroid_15909	conserved hypothetical protein	7	0	24	94	centroid_15909	18.416367	1.78E-05	centroid_15909	3.30E-05
centroid_15910	conserved hypothetical protein	7	0	24	94	centroid_15910	18.416367	1.78E-05	centroid_15910	3.30E-05
centroid_15911	putative korC protein	7	0	24	94	centroid_15911	18.416367	1.78E-05	centroid_15911	3.30E-05
centroid_16923	repressor protein C2	7	0	24	94	centroid_16923	18.416367	1.78E-05	centroid_16923	3.30E-05
centroid_17409	hypothetical protein	7	0	24	94	centroid_17409	18.416367	1.78E-05	centroid_17409	3.30E-05
centroid_5110	PIN domain protein	5	59	26	35	centroid_5110	18.469524	1.73E-05	centroid_5110	7.23E-06
centroid_10248	bacteriophage replication gene A family protein	5	57	26	37	centroid_10248	16.736722	4.29E-05	centroid_10248	2.03E-05
centroid_5169	putative membrane protein	1	43	30	51	centroid_5169	16.659679	4.47E-05	centroid_5169	6.00E-06
centroid_14162	initiator Replication family protein	0	43	31	51	centroid_14162	19.637559	9.36E-06	centroid_14162	3.55E-07
centroid_4143	initiator Replication family protein	0	42	31	52	centroid_4143	18.905412	1.37E-05	centroid_4143	3.74E-07

centroid_10641	hypothetical protein	0	39	31	55	centroid_10641	16.811464	4.13E-05	centroid_10641	2.27E-06
centroid_3978	R.Ecl18kI	0	39	31	55	centroid_3978	16.811464	4.13E-05	centroid_3978	2.27E-06
centroid_3980	conserved hypothetical protein	0	39	31	55	centroid_3980	16.811464	4.13E-05	centroid_3980	2.27E-06
centroid_3979	DNA (cytosine-5-)-methyltransferase family protein	0	37	31	57	centroid_3979	15.495128	8.27E-05	centroid_3979	5.36E-06

Table S4. Distribution by Geography - Calama

Gene_ID	Annotation	alama_prese	Other_prese	alama_Abse	Other_Absent	Gene_ID	chisq-stats	pvalues	Gene_ID	pvalues
centroid_17039	hypothetical protein	4	119	2	0	centroid_17039	21.919515	2.84E-06	centroid_17039	0.0019355
centroid_14919	putative taxA	4	7	2	112	centroid_14919	19.26779	1.14E-05	centroid_14919	0.0004645
centroid_14917	firmicute plasmid replication family protein	4	8	2	111	centroid_14917	17.247529	3.28E-05	centroid_14917	0.0006871
centroid_14918	putative oRF3	4	8	2	111	centroid_14918	17.247529	3.28E-05	centroid_14918	0.0006871
centroid_15409	plasmid mobilization domain protein	3	1	3	118	centroid_15409	30.106348	4.09E-08	centroid_15409	0.0002471
centroid_14646	putative membrane protein	3	4	3	115	centroid_14646	15.508375	8.21E-05	centroid_14646	0.0020437
centroid_14860	conserved hypothetical protein	3	4	3	115	centroid_14860	15.508375	8.21E-05	centroid_14860	0.0020437
centroid_14920	conserved hypothetical protein	3	4	3	115	centroid_14920	15.508375	8.21E-05	centroid_14920	0.0020437
centroid_15649	putative mobC	3	4	3	115	centroid_15649	15.508375	8.21E-05	centroid_15649	0.0020437
centroid_15650	mobA/MobL family protein	3	4	3	115	centroid_15650	15.508375	8.21E-05	centroid_15650	0.0020437
centroid_15651	mobilization A domain protein	3	4	3	115	centroid_15651	15.508375	8.21E-05	centroid_15651	0.0020437
centroid_15652	hypothetical protein	3	4	3	115	centroid_15652	15.508375	8.21E-05	centroid_15652	0.0020437
centroid_15653	putative repF	3	4	3	115	centroid_15653	15.508375	8.21E-05	centroid_15653	0.0020437
centroid_15654	regulatory protein RepA	3	4	3	115	centroid_15654	15.508375	8.21E-05	centroid_15654	0.0020437
centroid_15655	replication C family protein	3	4	3	115	centroid_15655	15.508375	8.21E-05	centroid_15655	0.0020437
centroid_16122	mobilization protein A	3	4	3	115	centroid_16122	15.508375	8.21E-05	centroid_16122	0.0020437
centroid_18288	phage tail protein I	2	0	4	119	centroid_18288	21.919515	2.84E-06	centroid_18288	0.0019355
centroid_18289	phage baseplate assembly V family protein	2	0	4	119	centroid_18289	21.919515	2.84E-06	centroid_18289	0.0019355
centroid_18290	phage late control gene D family protein	2	0	4	119	centroid_18290	21.919515	2.84E-06	centroid_18290	0.0019355
centroid_18291	phage P2 GpU family protein	2	0	4	119	centroid_18291	21.919515	2.84E-06	centroid_18291	0.0019355
centroid_18292	phage tail tape measure protein, TP901 family, core region	2	0	4	119	centroid_18292	21.919515	2.84E-06	centroid_18292	0.0019355
centroid_18294	conserved hypothetical protein	2	0	4	119	centroid_18294	21.919515	2.84E-06	centroid_18294	0.0019355
centroid_18295	phage major tail tube protein	2	0	4	119	centroid_18295	21.919515	2.84E-06	centroid_18295	0.0019355
centroid_18296	gp37 family protein	2	0	4	119	centroid_18296	21.919515	2.84E-06	centroid_18296	0.0019355
centroid_18297	conserved hypothetical protein	2	0	4	119	centroid_18297	21.919515	2.84E-06	centroid_18297	0.0019355
centroid_18298	putative dihydrolipoamide acyltransferase	2	0	4	119	centroid_18298	21.919515	2.84E-06	centroid_18298	0.0019355
centroid_18299	conserved hypothetical protein	2	0	4	119	centroid_18299	21.919515	2.84E-06	centroid_18299	0.0019355
centroid_18300	putative protease	2	0	4	119	centroid_18300	21.919515	2.84E-06	centroid_18300	0.0019355
centroid_18301	conserved hypothetical protein	2	0	4	119	centroid_18301	21.919515	2.84E-06	centroid_18301	0.0019355
centroid_18302	terminase-like family protein	2	0	4	119	centroid_18302	21.919515	2.84E-06	centroid_18302	0.0019355
centroid_18303	conserved hypothetical protein	2	0	4	119	centroid_18303	21.919515	2.84E-06	centroid_18303	0.0019355
centroid_18304	putative phage membrane protein	2	0	4	119	centroid_18304	21.919515	2.84E-06	centroid_18304	0.0019355
centroid_18307	conserved hypothetical protein	2	0	4	119	centroid_18307	21.919515	2.84E-06	centroid_18307	0.0019355
centroid_18308	DNA adenine methylase family protein	2	0	4	119	centroid_18308	21.919515	2.84E-06	centroid_18308	0.0019355
centroid_18309	conserved hypothetical protein	2	0	4	119	centroid_18309	21.919515	2.84E-06	centroid_18309	0.0019355
centroid_18310	helix-turn-helix family protein	2	0	4	119	centroid_18310	21.919515	2.84E-06	centroid_18310	0.0019355
centroid_18311	iclR helix-turn-helix domain protein	2	0	4	119	centroid_18311	21.919515	2.84E-06	centroid_18311	0.0019355
centroid_18313	integrase core domain protein	2	0	4	119	centroid_18313	21.919515	2.84E-06	centroid_18313	0.0019355
centroid_18314	AAA domain protein	2	0	4	119	centroid_18314	21.919515	2.84E-06	centroid_18314	0.0019355
centroid_18315	putative prophage exported protein	2	0	4	119	centroid_18315	21.919515	2.84E-06	centroid_18315	0.0019355
centroid_18316	conserved hypothetical protein	2	0	4	119	centroid_18316	21.919515	2.84E-06	centroid_18316	0.0019355
centroid_18317	conserved hypothetical protein	2	0	4	119	centroid_18317	21.919515	2.84E-06	centroid_18317	0.0019355
centroid_18318	conserved hypothetical protein	2	0	4	119	centroid_18318	21.919515	2.84E-06	centroid_18318	0.0019355
centroid_18319	conserved hypothetical protein	2	0	4	119	centroid_18319	21.919515	2.84E-06	centroid_18319	0.0019355
centroid_18320	putative membrane protein	2	0	4	119	centroid_18320	21.919515	2.84E-06	centroid_18320	0.0019355
centroid_18364	conserved hypothetical protein	2	0	4	119	centroid_18364	21.919515	2.84E-06	centroid_18364	0.0019355
centroid_18366	putative membrane protein	2	0	4	119	centroid_18366	21.919515	2.84E-06	centroid_18366	0.0019355
centroid_18367	hypothetical protein	2	0	4	119	centroid_18367	21.919515	2.84E-06	centroid_18367	0.0019355
centroid_18368	conjugal transfer/type IV secretion DotA/TraY family protein	2	0	4	119	centroid_18368	21.919515	2.84E-06	centroid_18368	0.0019355
centroid_18369	hypothetical protein	2	0	4	119	centroid_18369	21.919515	2.84E-06	centroid_18369	0.0019355
centroid_18370	putative membrane protein	2	0	4	119	centroid_18370	21.919515	2.84E-06	centroid_18370	0.0019355
centroid_18371	hypothetical protein	2	0	4	119	centroid_18371	21.919515	2.84E-06	centroid_18371	0.0019355
centroid_18372	hypothetical protein	2	0	4	119	centroid_18372	21.919515	2.84E-06	centroid_18372	0.0019355
centroid_18373	hypothetical protein	2	0	4	119	centroid_18373	21.919515	2.84E-06	centroid_18373	0.0019355
centroid_18374	AAA domain protein	2	0	4	119	centroid_18374	21.919515	2.84E-06	centroid_18374	0.0019355
centroid_18375	conserved hypothetical protein	2	0	4	119	centroid_18375	21.919515	2.84E-06	centroid_18375	0.0019355
centroid_18376	type III restriction-modification system EcoPI enzyme mod	2	0	4	119	centroid_18376	21.919515	2.84E-06	centroid_18376	0.0019355
centroid_18377	type III restriction enzyme, res subunit	2	0	4	119	centroid_18377	21.919515	2.84E-06	centroid_18377	0.0019355
centroid_18378	hypothetical protein	2	0	4	119	centroid_18378	21.919515	2.84E-06	centroid_18378	0.0019355
centroid_18379	hypothetical protein	2	0	4	119	centroid_18379	21.919515	2.84E-06	centroid_18379	0.0019355
centroid_18380	putative membrane protein	2	0	4	119	centroid_18380	21.919515	2.84E-06	centroid_18380	0.0019355
centroid_18381	hypothetical protein	2	0	4	119	centroid_18381	21.919515	2.84E-06	centroid_18381	0.0019355
centroid_18384	NACHT domain protein	2	0	4	119	centroid_18384	21.919515	2.84E-06	centroid_18384	0.0019355
centroid_18385	hypothetical protein	2	0	4	119	centroid_18385	21.919515	2.84E-06	centroid_18385	0.0019355
centroid_18386	conserved hypothetical protein	2	0	4	119	centroid_18386	21.919515	2.84E-06	centroid_18386	0.0019355
centroid_18387	transposase IS116/IS110/IS902 family protein	2	0	4	119	centroid_18387	21.919515	2.84E-06	centroid_18387	0.0019355
centroid_18388	plasmid partitioning protein ParF	2	0	4	119	centroid_18388	21.919515	2.84E-06	centroid_18388	0.0019355
centroid_18389	plasmid partition protein ParG	2	0	4	119	centroid_18389	21.919515	2.84E-06	centroid_18389	0.0019355
centroid_18390	ribbon-helix-helix , copG family protein	2	0	4	119	centroid_18390	21.919515	2.84E-06	centroid_18390	0.0019355
centroid_18391	conserved hypothetical protein	2	0	4	119	centroid_18391	21.919515	2.84E-06	centroid_18391	0.0019355
centroid_18392	conserved hypothetical protein	2	0	4	119	centroid_18392	21.919515	2.84E-06	centroid_18392	0.0019355
centroid_18393	conserved hypothetical protein	2	0	4	119	centroid_18393	21.919515	2.84E-06	centroid_18393	0.0019355
centroid_18394	initiator Replication family protein	2	0	4	119	centroid_18394	21.919515	2.84E-06	centroid_18394	0.0019355
centroid_18395	PI protein	2	0	4	119	centroid_18395	21.919515	2.84E-06	centroid_18395	0.0019355
centroid_18396	conserved hypothetical protein	2	0	4	119	centroid_18396	21.919515	2.84E-06	centroid_18396	0.0019355
centroid_18431	putative membrane protein	2	0	4	119	centroid_18431	21.919515	2.84E-06	centroid_18431	0.0019355
centroid_18433	conserved hypothetical protein	2	0	4	119	centroid_18433	21.919515	2.84E-06	centroid_18433	0.0019355
centroid_18434	putative membrane protein	2	0	4	119	centroid_18434	21.919515	2.84E-06	centroid_18434	0.0019355
centroid_18435	sigma-54 interaction domain protein	2	0	4	119	centroid_18435	21.919515	2.84E-06	centroid_18435	0.0019355
centroid_18436	conserved hypothetical protein	2	0	4	119	centroid_18436	21.919515	2.84E-06	centroid_18436	0.0019355
centroid_18439	trbL/VirB6 plasmid conjugal transfer family protein	2	0	4	119	centroid_18439	21.919515	2.84E-06	centroid_18439	0.0019355
centroid_18453	putative eex protein	2	0	4	119	centroid_18453	21.919515	2.84E-06	centroid_18453	0.0019355
centroid_18454	bacterial regulatory, luxR family protein	2	0	4	119	centroid_18454	21.919515	2.84E-06	centroid_18454	0.0019355
centroid_18455	arc-like DNA binding domain protein	2	0	4	119	centroid_18455	21.919515	2.84E-06	centroid_18455	0.0019355
centroid_18456	conserved hypothetical protein	2	0	4	119	centroid_18456	21.919515	2.84E-06	centroid_18456	0.0019355
centroid_18458	hypothetical protein	2	0	4	119	centroid_18458	21.919515	2.84E-06	centroid_18458	0.0019355
centroid_18459	conserved hypothetical protein	2	0	4	119	centroid_18459	21.919515	2.84E-06	centroid_18459	0.0019355
centroid_18471	putative plasmid mobilization protein	2	0	4	119	centroid_18471	21.919515	2.84E-06	centroid_18471	0.0019355
centroid_18589	baseplate J-like family protein	2	0	4	119	centroid_18589	21.919515	2.84E-06	centroid_18589	0.0019355
centroid_18593	conserved hypothetical protein	2	0	4	119	centroid_18593	21.919515	2.84E-06	centroid_18593	0.0019355
centroid_18678	type III restriction enzyme, res subunit	2	0	4	119	centroid_18678	21.919515	2.84E-06	centroid_18678	0.0019355
centroid_18679	putative type III restriction-modification system enzyme, res subuni	2	0	4	119	centroid_18679	21.919515	2.84E-06	centroid_18679	0.0019355
centroid_18681	putative taxA	2	0	4	119	centroid_18681	21.919515	2.84E-06	centroid_18681	0.0019355
centroid_11791	conserved hypothetical protein	2	111	4	8	centroid_11791	17.247529	3.28E-05	centroid_11791	0.0006871
