# Supplementary material for: Comparative genomic analysis and molecular examination of the diversity of enterotoxigenic Escherichia coli isolates from Chile
Source: PLoS Negl Trop Dis. 2019 Nov 20;13(11):e0007828. doi: 10.1371/journal.pntd.0007828 (PMC6901236; doi:10.1371/journal.pntd.0007828)
Supplement: S5 Table — (PDF) [file pntd.0007828.s006.pdf]

Table S5. Distribution by Phylogroup A

Gene_ID	Annotation	Phylogroup A_present	Other_present	Phylogroup A_Absent	Other_Absent	Gene_ID	chisq-stats	pvalues	Gene_ID	pvalues
centroid_17467	type II secretion system protein L	101	1	0	23	centroid_1746	112.320341	3.04001E-26	centroid_1746	3.1685E-24
centroid_3084	BFD-like [2Fe-2S] binding domain protein	101	1	0	23	centroid_3084	112.320341	3.04001E-26	centroid_3084	3.1685E-24
centroid_3085	bacterioferritin	101	1	0	23	centroid_3085	112.320341	3.04001E-26	centroid_3085	3.1685E-24
centroid_3086	e 4 prepilin-like proteins leader peptide-processing enzy	101	1	0	23	centroid_3086	112.320341	3.04001E-26	centroid_3086	3.1685E-24
centroid_3087	type II secretion system (T2SS), M family protein	101	1	0	23	centroid_3087	112.320341	3.04001E-26	centroid_3087	3.1685E-24
centroid_3098	AAA domain protein	101	1	0	23	centroid_3098	112.320341	3.04001E-26	centroid_3098	3.1685E-24
centroid_3099	putative peptidoglycan binding domain protein	101	1	0	23	centroid_3099	112.320341	3.04001E-26	centroid_3099	3.1685E-24
centroid_3100	lcium-binding protein required for initiation of chromosome	101	1	0	23	centroid_3100	112.320341	3.04001E-26	centroid_3100	3.1685E-24
centroid_4710	gntP permease family protein	101	1	0	23	centroid_4710	112.320341	3.04001E-26	centroid_4710	3.1685E-24
centroid_5500	gspL periplasmic domain protein	101	1	0	23	centroid_5500	112.320341	3.04001E-26	centroid_5500	3.1685E-24
centroid_8581	putative general secretion pathway protein A	101	1	0	23	centroid_8581	112.320341	3.04001E-26	centroid_8581	3.1685E-24
centroid_16783	hypothetical protein	101	3	0	21	centroid_1678	100.052169	1.48435E-23	centroid_1678	5.5668E-21
centroid_9217	conserved hypothetical protein	101	3	0	21	centroid_9217	100.052169	1.48435E-23	centroid_9217	5.5668E-21
centroid_1035	2-keto-3-deoxy-L-rhamnonate aldolase	101	4	0	20	centroid_1035	94.0939379	3.00899E-22	centroid_1035	1.4849E-19
centroid_1036	major Facilitator Superfamily protein	101	4	0	20	centroid_1036	94.0939379	3.00899E-22	centroid_1036	1.4849E-19
centroid_1037	major Facilitator Superfamily protein	101	4	0	20	centroid_1037	94.0939379	3.00899E-22	centroid_1037	1.4849E-19
centroid_1038	L-rhamnonate dehydratase	101	4	0	20	centroid_1038	94.0939379	3.00899E-22	centroid_1038	1.4849E-19
centroid_1039	conserved hypothetical protein	101	4	0	20	centroid_1039	94.0939379	3.00899E-22	centroid_1039	1.4849E-19
centroid_14832	putative bifunctional chitinase/lysozyme domain protein	101	4	0	20	centroid_1483	94.0939379	3.00899E-22	centroid_1483	1.4849E-19
centroid_16153	bacterial transcriptional regulator family protein	101	4	0	20	centroid_1615	94.0939379	3.00899E-22	centroid_1615	1.4849E-19
centroid_16154	iclR helix-turn-helix domain protein	101	4	0	20	centroid_1615	94.0939379	3.00899E-22	centroid_1615	1.4849E-19
centroid_2637	conserved hypothetical protein	101	4	0	20	centroid_2637	94.0939379	3.00899E-22	centroid_2637	1.4849E-19
centroid_2747	antitoxin MqsA	101	4	0	20	centroid_2747	94.0939379	3.00899E-22	centroid_2747	1.4849E-19
centroid_4273	inner membrane transport protein RhmT	101	4	0	20	centroid_4273	94.0939379	3.00899E-22	centroid_4273	1.4849E-19
centroid_7695	bacterial transcriptional regulator family protein	101	4	0	20	centroid_7695	94.0939379	3.00899E-22	centroid_7695	1.4849E-19
centroid_7696	iclR helix-turn-helix domain protein	101	4	0	20	centroid_7696	94.0939379	3.00899E-22	centroid_7696	1.4849E-19
centroid_8579	putative bifunctional chitinase/lysozyme domain protein	101	4	0	20	centroid_8579	94.0939379	3.00899E-22	centroid_8579	1.4849E-19
centroid_1113	inner membrane protein YhaI	101	5	0	19	centroid_1113	88.2486029	5.7724E-21	centroid_1113	3.148E-18
centroid_1340	conserved hypothetical protein	101	5	0	19	centroid_1340	88.2486029	5.7724E-21	centroid_1340	3.148E-18
centroid_14713	conserved hypothetical protein	101	5	0	19	centroid_1471	88.2486029	5.7724E-21	centroid_1471	3.148E-18
centroid_14714	conserved hypothetical protein	101	5	0	19	centroid_1471	88.2486029	5.7724E-21	centroid_1471	3.148E-18
centroid_2947	papC N-terminal domain protein	101	6	0	18	centroid_2947	82.5130769	1.04973E-19	centroid_2947	5.614E-17
centroid_9257	protein DedA	101	6	0	18	centroid_9257	82.5130769	1.04973E-19	centroid_9257	5.614E-17
centroid_9965	biquinone/plastoquinone (complex I), various chains fami	101	6	0	18	centroid_9965	82.5130769	1.04973E-19	centroid_9965	5.614E-17
centroid_3231	conserved hypothetical protein	101	7	0	17	centroid_3231	76.884406	1.81263E-18	centroid_3231	8.6616E-16
centroid_5319	conserved hypothetical protein	101	7	0	17	centroid_5319	76.884406	1.81263E-18	centroid_5319	8.6616E-16
centroid_1012	sensory box protein	101	8	0	16	centroid_1012	71.3597684	2.9768E-17	centroid_1012	1.1801E-14
centroid_1013	response regulator	101	8	0	16	centroid_1013	71.3597684	2.9768E-17	centroid_1013	1.1801E-14
centroid_1014	acetate CoA-transferase subunit alpha	101	8	0	16	centroid_1014	71.3597684	2.9768E-17	centroid_1014	1.1801E-14
centroid_1015	acetate CoA-transferase subunit beta	101	8	0	16	centroid_1015	71.3597684	2.9768E-17	centroid_1015	1.1801E-14
centroid_1016	short-chain fatty acids transporter	101	8	0	16	centroid_1016	71.3597684	2.9768E-17	centroid_1016	1.1801E-14
centroid_1017	acetyl-CoA C-acetyltransferase family protein	101	8	0	16	centroid_1017	71.3597684	2.9768E-17	centroid_1017	1.1801E-14
centroid_17737	acterial extracellular solute-binding, 5 Middle family protei	101	8	0	16	centroid_1773	71.3597684	2.9768E-17	centroid_1773	1.1801E-14
centroid_17738	acterial extracellular solute-binding, 5 Middle family protei	101	8	0	16	centroid_1773	71.3597684	2.9768E-17	centroid_1773	1.1801E-14
centroid_2684	conserved hypothetical protein	101	8	0	16	centroid_2684	71.3597684	2.9768E-17	centroid_2684	1.1801E-14
centroid_2685	inner membrane protein YigG	101	8	0	16	centroid_2685	71.3597684	2.9768E-17	centroid_2685	1.1801E-14
centroid_2746	putative binding protein YgiS	101	8	0	16	centroid_2746	71.3597684	2.9768E-17	centroid_2746	1.1801E-14
centroid_7693	ative signal transduction histidine-kinase atoS domain pro	101	8	0	16	centroid_7693	71.3597684	2.9768E-17	centroid_7693	1.1801E-14
centroid_7694	sensory box protein	101	8	0	16	centroid_7694	71.3597684	2.9768E-17	centroid_7694	1.1801E-14
centroid_8264	thiolase, N-terminal domain protein	101	8	0	16	centroid_8264	71.3597684	2.9768E-17	centroid_8264	1.1801E-14
centroid_8265	acetyl-CoA C-acetyltransferase family protein	101	8	0	16	centroid_8265	71.3597684	2.9768E-17	centroid_8265	1.1801E-14
centroid_14303	cyclic di-GMP phosphodiesterase YahA	101	9	0	15	centroid_1430	65.9364765	4.65693E-16	centroid_1430	1.4424E-13
centroid_8383	protein YoaG	101	9	0	15	centroid_8383	65.9364765	4.65693E-16	centroid_8383	1.4424E-13
centroid_17674	conserved hypothetical protein	101	10	0	14	centroid_1767	60.6119817	6.95096E-15	centroid_1767	1.6011E-12
centroid_2839	conserved hypothetical protein	101	10	0	14	centroid_2839	60.6119817	6.95096E-15	centroid_2839	1.6011E-12
centroid_2840	repair family protein	101	10	0	14	centroid_2840	60.6119817	6.95096E-15	centroid_2840	1.6011E-12
centroid_4506	conserved hypothetical protein	101	10	0	14	centroid_4506	60.6119817	6.95096E-15	centroid_4506	1.6011E-12
centroid_4916	conserved hypothetical protein	101	10	0	14	centroid_4916	60.6119817	6.95096E-15	centroid_4916	1.6011E-12
centroid_4431	conserved hypothetical protein	101	12	0	12	centroid_4431	50.2499529	1.35358E-12	centroid_4431	1.5351E-10
centroid_958	pfkB carbohydrate kinase family protein	101	12	0	12	centroid_958	50.2499529	1.35358E-12	centroid_958	1.5351E-10
centroid_2828	FAD dependent oxidoreductase family protein	101	14	0	10	centroid_2828	40.2566836	2.22692E-10	centroid_2828	1.1058E-08
centroid_2829	putative 4Fe-4S binding protein	101	14	0	10	centroid_2829	40.2566836	2.22692E-10	centroid_2829	1.1058E-08
centroid_2830	conserved hypothetical protein	101	14	0	10	centroid_2830	40.2566836	2.22692E-10	centroid_2830	1.1058E-08
centroid_4505	carbohydrate kinase, FGGY family	101	14	0	10	centroid_4505	40.2566836	2.22692E-10	centroid_4505	1.1058E-08
centroid_470	conserved hypothetical protein	101	14	0	10	centroid_470	40.2566836	2.22692E-10	centroid_470	1.1058E-08
centroid_13071	conserved hypothetical protein	101	15	0	9	centroid_1307	35.3940959	2.69302E-09	centroid_1307	8.5512E-08
centroid_18532	type VI secretion system effector, Hcp1 family protein	101	16	0	8	centroid_1853	30.6194119	3.13937E-08	centroid_1853	6.2531E-07
centroid_3606	putative mRNA interferase HicA	101	16	0	8	centroid_3606	30.6194119	3.13937E-08	centroid_3606	6.2531E-07
centroid_848	type VI secretion system effector, Hcp1 family protein	101	16	0	8	centroid_848	30.6194119	3.13937E-08	centroid_848	6.2531E-07
centroid_6124	yhaC domain protein	101	17	0	7	centroid_6124	25.9324288	3.53579E-07	centroid_6124	4.3404E-06
centroid_961	putative pseudouridine transporter	101	17	0	7	centroid_961	25.9324288	3.53579E-07	centroid_961	4.3404E-06
centroid_18274	gram-negative porin family protein	101	18	0	6	centroid_1827	21.3342943	3.85768E-06	centroid_1827	2.8695E-05
centroid_846	conserved hypothetical protein	101	18	0	6	centroid_846	21.3342943	3.85768E-06	centroid_846	2.8695E-05
centroid_847	conserved hypothetical protein	101	18	0	6	centroid_847	21.3342943	3.85768E-06	centroid_847	2.8695E-05
centroid_9306	type VI secretion system effector, Hcp1 family protein	101	18	0	6	centroid_9306	21.3342943	3.85768E-06	centroid_9306	2.8695E-05
centroid_1060	fimbrial family protein	101	19	0	5	centroid_1060	16.8287825	4.09081E-05	centroid_1060	0.00018123
centroid_10769	amino acid permease family protein	101	19	0	5	centroid_1076	16.8287825	4.09081E-05	centroid_1076	0.00018123
centroid_10770	amino acid permease family protein	101	19	0	5	centroid_1077	16.8287825	4.09081E-05	centroid_1077	0.00018123
centroid_11086	o-L-gulonate-6-phosphate decarboxylase UlaD domain p	101	19	0	5	centroid_1108	16.8287825	4.09081E-05	centroid_1108	0.00018123
centroid_11948	GY family of carbohydrate kinase, N-terminal domain pro	101	19	0	5	centroid_1194	16.8287825	4.09081E-05	centroid_1194	0.00018123
centroid_1240	type VII secretion system (T7SS), usher family protein	101	19	0	5	centroid_1240	16.8287825	4.09081E-05	centroid_1240	0.00018123
centroid_1241	fimbrial family protein	101	19	0	5	centroid_1241	16.8287825	4.09081E-05	centroid_1241	0.00018123
centroid_13580	type VII secretion system (T7SS), usher family protein	101	19	0	5	centroid_1358	16.8287825	4.09081E-05	centroid_1358	0.00018123
centroid_13867	type VII secretion system (T7SS), usher family protein	101	19	0	5	centroid_1386	16.8287825	4.09081E-05	centroid_1386	0.00018123
centroid_14011	class II Aldolase and Adducin N-terminal domain protein	101	19	0	5	centroid_1401	16.8287825	4.09081E-05	centroid_1401	0.00018123
centroid_14613	conserved hypothetical protein	101	19	0	5	centroid_1461	16.8287825	4.09081E-05	centroid_1461	0.00018123
centroid_15451	conserved hypothetical protein	101	19	0	5	centroid_1545	16.8287825	4.09081E-05	centroid_1545	0.00018123
centroid_1582	conserved hypothetical protein	101	19	0	5	centroid_1582	16.8287825	4.09081E-05	centroid_1582	0.00018123
centroid_1666	conserved hypothetical protein	101	19	0	5	centroid_1666	16.8287825	4.09081E-05	centroid_1666	0.00018123
centroid_16905	major MR/P fimbria domain protein	101	19	0	5	centroid_1690	16.8287825	4.09081E-05	centroid_1690	0.00018123
centroid_17640	conserved hypothetical protein	101	19	0	5	centroid_1764	16.8287825	4.09081E-05	centroid_1764	0.00018123
centroid_1885	conserved hypothetical protein	101	19	0	5	centroid_1885	16.8287825	4.09081E-05	centroid_1885	0.00018123
centroid_2634	putative 4-phosphopantetheinyl transferase EntD	101	19	0	5	centroid_2634	16.8287825	4.09081E-05	centroid_2634	0.00018123
centroid_2767	conserved hypothetical protein	101	19	0	5	centroid_2767	16.8287825	4.09081E-05	centroid_2767	0.00018123
centroid_3536	conserved hypothetical protein	101	19	0	5	centroid_3536	16.8287825	4.09081E-05	centroid_3536	0.00018123
centroid_3537	2,3-diketo-L-gulonate reductase	101	19	0	5	centroid_3537	16.8287825	4.09081E-05	centroid_3537	0.00018123
centroid_3538	HTH-type transcriptional regulator YiaJ	101	19	0	5	centroid_3538	16.8287825	4.09081E-05	centroid_3538	0.00018123
centroid_3815	TRAP transporter solute receptor, DctP family protein	101	19	0	5	centroid_3815	16.8287825	4.09081E-05	centroid_3815	0.00018123
centroid_3816	3-keto-L-gulonate-6-phosphate decarboxylase SgbH	101	19	0	5	centroid_3816	16.8287825	4.09081E-05	centroid_3816	0.00018123
centroid_3817	xylose isomerase-like TIM barrel family protein	101	19	0	5	centroid_3817	16.8287825	4.09081E-05	centroid_3817	0.00018123
centroid_4604	inner membrane protein YgiZ	101	19	0	5	centroid_4604	16.8287825	4.09081E-05	centroid_4604	0.00018123
centroid_5298	xylose isomerase-like TIM barrel family protein	101	19	0	5	centroid_5298	16.8287825	4.09081E-05	centroid_5298	0.00018123
centroid_5299	xylose isomerase-like TIM barrel family protein	101	19	0	5	centroid_5299	16.8287825	4.09081E-05	centroid_5299	0.00018123
centroid_6197	maltose acetyltransferase family protein	101	19	0	5	centroid_6197	16.8287825	4.09081E-05	centroid_6197	0.00018123
centroid_6935	hypothetical protein	101	19	0	5	centroid_6935	16.8287825	4.09081E-05	centroid_6935	0.00018123

centroid_7184	papC N-terminal domain protein	101	19	0	5	centroid_7184	16.8287825	4.09081E-05	centroid_7184	0.00018123
centroid_7185	type VII secretion system (T7SS), usher family protein	101	19	0	5	centroid_7185	16.8287825	4.09081E-05	centroid_7185	0.00018123
centroid_7218	galactoside O-acetyltransferase	101	19	0	5	centroid_7218	16.8287825	4.09081E-05	centroid_7218	0.00018123
centroid_77	conserved hypothetical protein	101	19	0	5	centroid_77	16.8287825	4.09081E-05	centroid_77	0.00018123
centroid_845	conserved hypothetical protein	101	19	0	5	centroid_845	16.8287825	4.09081E-05	centroid_845	0.00018123
centroid_911	HTH-type transcriptional regulator FrlR	101	19	0	5	centroid_911	16.8287825	4.09081E-05	centroid_911	0.00018123
centroid_912	pfkB carbohydrate kinase family protein	101	19	0	5	centroid_912	16.8287825	4.09081E-05	centroid_912	0.00018123
centroid_913	xylose isomerase-like TIM barrel family protein	101	19	0	5	centroid_913	16.8287825	4.09081E-05	centroid_913	0.00018123
centroid_914	SIS domain protein	101	19	0	5	centroid_914	16.8287825	4.09081E-05	centroid_914	0.00018123
centroid_915	amino acid permease family protein	101	19	0	5	centroid_915	16.8287825	4.09081E-05	centroid_915	0.00018123
centroid_9912	type VII secretion system (T7SS), usher family protein	101	19	0	5	centroid_9912	16.8287825	4.09081E-05	centroid_9912	0.00018123
centroid_9913	type VII secretion system (T7SS), usher family protein	101	19	0	5	centroid_9913	16.8287825	4.09081E-05	centroid_9913	0.00018123
centroid_9962	gamma-glutamyltranspeptidase domain protein	101	19	0	5	centroid_9962	16.8287825	4.09081E-05	centroid_9962	0.00018123
centroid_10869	type II secretion system protein H	100	1	1	23	centroid_1086	106.410035	5.99534E-25	centroid_1086	7.533E-23
centroid_12074	type II secretion system protein H	100	1	1	23	centroid_1207	106.410035	5.99534E-25	centroid_1207	7.533E-23
centroid_17465	type II secretion system (T2SS), F family protein	100	1	1	23	centroid_1746	106.410035	5.99534E-25	centroid_1746	7.533E-23
centroid_17466	type II secretion system (T2SS), F family protein	100	1	1	23	centroid_1746	106.410035	5.99534E-25	centroid_1746	7.533E-23
centroid_3083	putative bifunctional chitinase/lysozyme	100	1	1	23	centroid_3083	106.410035	5.99534E-25	centroid_3083	7.533E-23
centroid_3089	type II secretion system (T2SS), K family protein	100	1	1	23	centroid_3089	106.410035	5.99534E-25	centroid_3089	7.533E-23
centroid_3090	repilin-type N-terminal cleavage/methylation domain prote	100	1	1	23	centroid_3090	106.410035	5.99534E-25	centroid_3090	7.533E-23
centroid_3091	type II secretion system protein I	100	1	1	23	centroid_3091	106.410035	5.99534E-25	centroid_3091	7.533E-23
centroid_3092	type II secretion system protein H	100	1	1	23	centroid_3092	106.410035	5.99534E-25	centroid_3092	7.533E-23
centroid_3093	type II secretion system protein G	100	1	1	23	centroid_3093	106.410035	5.99534E-25	centroid_3093	7.533E-23
centroid_3094	type II secretion system protein F	100	1	1	23	centroid_3094	106.410035	5.99534E-25	centroid_3094	7.533E-23
centroid_3095	type II secretion system protein E	100	1	1	23	centroid_3095	106.410035	5.99534E-25	centroid_3095	7.533E-23
centroid_4742	repilin-type N-terminal cleavage/methylation domain prote	100	1	1	23	centroid_4742	106.410035	5.99534E-25	centroid_4742	7.533E-23
centroid_7722	type II secretion system protein F	100	1	1	23	centroid_7722	106.410035	5.99534E-25	centroid_7722	7.533E-23
centroid_7723	putative type II secretion system F domain protein	100	1	1	23	centroid_7723	106.410035	5.99534E-25	centroid_7723	7.533E-23
centroid_8069	type II secretion system protein D	100	1	1	23	centroid_8069	106.410035	5.99534E-25	centroid_8069	7.533E-23
centroid_8070	type II secretion system protein L	100	1	1	23	centroid_8070	106.410035	5.99534E-25	centroid_8070	7.533E-23
centroid_8580	carbohydrate binding domain protein	100	1	1	23	centroid_8580	106.410035	5.99534E-25	centroid_8580	7.533E-23
centroid_9733	conserved hypothetical protein	100	2	1	22	centroid_9733	100.241727	1.34888E-23	centroid_9733	3.6834E-21
centroid_9734	hypothetical protein	100	2	1	22	centroid_9734	100.241727	1.34888E-23	centroid_9734	3.6834E-21
centroid_5432	conserved hypothetical protein	100	6	1	18	centroid_5432	76.7649415	1.92566E-18	centroid_5432	1.01E-15
centroid_11950	FHIPEP family protein	100	9	1	15	centroid_1195	60.3380689	7.98873E-15	centroid_1195	2.1308E-12
centroid_17686	putative domain protein	100	10	1	14	centroid_1768	55.0760154	1.15957E-13	centroid_1768	2.1997E-11
centroid_11278	aldo/keto reductase family protein	100	12	1	12	centroid_1127	44.8649328	2.11105E-11	centroid_1127	1.8E-09
centroid_11408	zinc-binding dehydrogenase family protein	100	12	1	12	centroid_1140	44.8649328	2.11105E-11	centroid_1140	1.8E-09
centroid_13575	pfkB carbohydrate kinase family protein	100	12	1	12	centroid_1357	44.8649328	2.11105E-11	centroid_1357	1.8E-09
centroid_3166	major Facilitator Superfamily protein	100	12	1	12	centroid_3166	44.8649328	2.11105E-11	centroid_3166	1.8E-09
centroid_3167	alcohol dehydrogenase GroES-like domain protein	100	12	1	12	centroid_3167	44.8649328	2.11105E-11	centroid_3167	1.8E-09
centroid_3844	aldo/keto reductase family protein	100	12	1	12	centroid_3844	44.8649328	2.11105E-11	centroid_3844	1.8E-09
centroid_3845	deoR-like helix-turn-helix domain protein	100	12	1	12	centroid_3845	44.8649328	2.11105E-11	centroid_3845	1.8E-09
centroid_3846	inner membrane metabolite transport protein YdjE	100	12	1	12	centroid_3846	44.8649328	2.11105E-11	centroid_3846	1.8E-09
centroid_4236	major Facilitator Superfamily protein	100	12	1	12	centroid_4236	44.8649328	2.11105E-11	centroid_4236	1.8E-09
centroid_5297	pfkB carbohydrate kinase family protein	100	12	1	12	centroid_5297	44.8649328	2.11105E-11	centroid_5297	1.8E-09
centroid_5512	sugar (and other) transporter family protein	100	12	1	12	centroid_5512	44.8649328	2.11105E-11	centroid_5512	1.8E-09
centroid_7566	zinc-binding dehydrogenase family protein	100	12	1	12	centroid_7566	44.8649328	2.11105E-11	centroid_7566	1.8E-09
centroid_7567	alcohol dehydrogenase GroES-like domain protein	100	12	1	12	centroid_7567	44.8649328	2.11105E-11	centroid_7567	1.8E-09
centroid_9282	sugar (and other) transporter family protein	100	12	1	12	centroid_9282	44.8649328	2.11105E-11	centroid_9282	1.8E-09
centroid_9283	sugar (and other) transporter family protein	100	12	1	12	centroid_9283	44.8649328	2.11105E-11	centroid_9283	1.8E-09
centroid_13866	type VII secretion system (T7SS), usher family protein	100	14	1	10	centroid_1386	35.0714451	3.17827E-09	centroid_1386	1.0817E-07
centroid_7792	thanolamine utilization - propanediol utilization family prote	100	14	1	10	centroid_7792	35.0714451	3.17827E-09	centroid_7792	1.0817E-07
centroid_8736	thanolamine utilization - propanediol utilization family prote	100	14	1	10	centroid_8736	35.0714451	3.17827E-09	centroid_8736	1.0817E-07
centroid_17241	conserved hypothetical protein	100	17	1	7	centroid_1724	21.212171	4.11145E-06	centroid_1724	3.0346E-05
centroid_10278	type VII secretion system (T7SS), usher family protein	100	18	1	6	centroid_1027	16.8487798	4.04793E-05	centroid_1027	0.00017482
centroid_10331	type VII secretion system (T7SS), usher family protein	100	18	1	6	centroid_1033	16.8487798	4.04793E-05	centroid_1033	0.00017482
centroid_11121	hypothetical protein	100	18	1	6	centroid_1112	16.8487798	4.04793E-05	centroid_1112	0.00017482
centroid_11884	papC N-terminal domain protein	100	18	1	6	centroid_1188	16.8487798	4.04793E-05	centroid_1188	0.00017482
centroid_13001	tonB-dependent siderophore receptor family protein	100	18	1	6	centroid_1300	16.8487798	4.04793E-05	centroid_1300	0.00017482
centroid_13002	tonB-dependent Receptor Plug domain protein	100	18	1	6	centroid_1300	16.8487798	4.04793E-05	centroid_1300	0.00017482
centroid_1513	putative ybl100	100	18	1	6	centroid_1513	16.8487798	4.04793E-05	centroid_1513	0.00017482
centroid_240	ferrichrome-iron receptor	100	18	1	6	centroid_240	16.8487798	4.04793E-05	centroid_240	0.00017482
centroid_9275	tonB-dependent Receptor Plug domain protein	100	18	1	6	centroid_9275	16.8487798	4.04793E-05	centroid_9275	0.00017482
centroid_9957	ferrichrome-iron receptor	100	18	1	6	centroid_9957	16.8487798	4.04793E-05	centroid_9957	0.00017482
centroid_13907	conserved hypothetical protein	99	0	2	24	centroid_1390	107.227915	3.96808E-25	centroid_1390	1.0096E-23
centroid_1481	putative lipoprotein	99	0	2	24	centroid_1481	107.227915	3.96808E-25	centroid_1481	1.0096E-23
centroid_1482	conserved hypothetical protein	99	0	2	24	centroid_1482	107.227915	3.96808E-25	centroid_1482	1.0096E-23
centroid_8471	conserved hypothetical protein	99	0	2	24	centroid_8471	107.227915	3.96808E-25	centroid_8471	1.0096E-23
centroid_17801	putative membrane protein	99	3	2	21	centroid_1780	88.850023	4.25909E-21	centroid_1780	1.3531E-18
centroid_1781	inner membrane protein YmfA	99	3	2	21	centroid_1781	88.850023	4.25909E-21	centroid_1781	1.3531E-18
centroid_15706	hypothetical protein	99	11	2	13	centroid_1570	45.1922145	1.78614E-11	centroid_1570	1.4126E-09
centroid_3381	alpha amylase, catalytic domain protein	99	11	2	13	centroid_3381	45.1922145	1.78614E-11	centroid_3381	1.4126E-09
centroid_12827	pfkB carbohydrate kinase family protein	99	12	2	12	centroid_1282	40.2620038	2.22087E-10	centroid_1282	1.1355E-08
centroid_3841	alcohol dehydrogenase GroES-like domain protein	99	12	2	12	centroid_3841	40.2620038	2.22087E-10	centroid_3841	1.1355E-08
centroid_3842	ketose-bisphosphate aldolase family protein	99	12	2	12	centroid_3842	40.2620038	2.22087E-10	centroid_3842	1.1355E-08
centroid_4320	zinc-binding dehydrogenase family protein	99	12	2	12	centroid_4320	40.2620038	2.22087E-10	centroid_4320	1.1355E-08
centroid_4319	alcohol dehydrogenase GroES-like domain protein	99	13	2	11	centroid_4319	35.4527729	2.6131E-09	centroid_4319	8.4124E-08
centroid_4773	conserved hypothetical protein	99	14	2	10	centroid_4773	30.769472	2.90573E-08	centroid_4773	5.7671E-07
centroid_6916	conserved hypothetical protein	99	15	2	9	centroid_6916	26.2198198	3.04679E-07	centroid_6916	3.669E-06
centroid_2768	putative type-1 fimbrial protein, A chain	99	16	2	8	centroid_2768	21.8156411	3.00143E-06	centroid_2768	2.1696E-05
centroid_7818	conserved hypothetical protein	99	16	2	8	centroid_7818	21.8156411	3.00143E-06	centroid_7818	2.1696E-05
centroid_7097	conserved hypothetical protein	99	17	2	7	centroid_7097	17.5750996	2.76181E-05	centroid_7097	0.00011925
centroid_3096	type II secretion system protein D	98	1	3	23	centroid_3096	95.953751	1.17599E-22	centroid_3096	8.0059E-21
centroid_8068	type II secretion system D domain protein	98	1	3	23	centroid_8068	95.953751	1.17599E-22	centroid_8068	8.0059E-21
centroid_2542	helix-turn-helix domain protein	98	4	3	20	centroid_2542	78.1452281	9.57366E-19	centroid_2542	2.3514E-16
centroid_3555	integrase core domain protein	98	4	3	20	centroid_3555	78.1452281	9.57366E-19	centroid_3555	2.3514E-16
centroid_4665	conserved hypothetical protein	98	4	3	20	centroid_4665	78.1452281	9.57366E-19	centroid_4665	2.3514E-16
centroid_4814	conserved hypothetical protein	98	4	3	20	centroid_4814	78.1452281	9.57366E-19	centroid_4814	2.3514E-16
centroid_16928	yiaA/B two helix domain protein	98	5	3	19	centroid_1692	72.468721	1.697E-17	centroid_1692	4.2197E-15
centroid_17238	alpha amylase, catalytic domain protein	98	10	3	14	centroid_1723	45.9817294	1.19361E-11	centroid_1723	8.3467E-10
centroid_10622	putative transposase DNA-binding domain protein	98	11	3	13	centroid_1062	41.066727	1.4712E-10	centroid_1062	6.8359E-09
centroid_5520	integrase core domain protein	98	11	3	13	centroid_5520	41.066727	1.4712E-10	centroid_5520	6.8359E-09
centroid_5511	conserved hypothetical protein	98	12	3	12	centroid_5511	36.2850738	1.70464E-09	centroid_5511	5.1127E-08
centroid_3097	type II secretion system protein C	97	1	4	23	centroid_3097	91.3067192	1.23042E-21	centroid_3097	5.3518E-20
centroid_17796	outer membrane autotransporter barrel domain protein	97	4	4	20	centroid_1779	73.7175449	9.01342E-18	centroid_1779	1.3582E-15
centroid_4666	outer membrane autotransporter barrel domain protein	97	4	4	20	centroid_4666	73.7175449	9.01342E-18	centroid_4666	1.3582E-15
centroid_5353	integrase core domain protein	97	4	4	20	centroid_5353	73.7175449	9.01342E-18	centroid_5353	1.3582E-15
centroid_8894	outer membrane autotransporter barrel domain protein	97	4	4	20	centroid_8894	73.7175449	9.01342E-18	centroid_8894	1.3582E-15
centroid_14132	integrase core domain protein	97	13	4	11	centroid_1413	28.3545933	1.01007E-07	centroid_1413	1.1754E-06
centroid_5513	integrase core domain protein	97	14	4	10	centroid_5513	24.0600036	9.33799E-07	centroid_5513	6.8879E-06
centroid_15977	transposase, IS605 OrfB family	97	15	4	9	centroid_1597	19.9488294	7.95426E-06	centroid_1597	3.7083E-05
centroid_10180	integrase core domain protein	96	11	5	13	centroid_1018	34.2186009	4.92559E-09	centroid_1018	8.59E-08
centroid_14811	integrase core domain protein	96	11	5	13	centroid_1481	34.2186009	4.92559E-09	centroid_1481	8.59E-08
centroid_11719	integrase core domain protein	96	13	5	11	centroid_1171	25.4914614	4.44345E-07	centroid_1171	3.3566E-06
centroid_13637	integrase core domain protein	96	13	5	11	centroid_1363	25.4914614	4.44345E-07	centroid_1363	3.3566E-06
centroid_4246	integrase core domain protein	96	13	5	11	centroid_4246	25.4914614	4.44345E-07	centroid_4246	3.3566E-06

centroid_10137	caudovirales tail fibre assembly family protein	96	14	5	10	centroid_1013	21.4007729	3.72621E-06	centroid_1013	1.8313E-05
centroid_7172	istB-like ATP binding family protein	96	15	5	9	centroid_7172	17.514495	2.85126E-05	centroid_7172	9.1433E-05
centroid_14088	integrase core domain protein	95	11	6	13	centroid_1408	31.3487889	2.15593E-08	centroid_1408	2.4621E-07
centroid_12444	uvate-dependent sugar phosphotransferase system, EIIA	95	15	6	9	centroid_1244	15.4236127	8.59081E-05	centroid_1244	0.00020111
centroid_2249	corbate-specific phosphotransferase enzyme IIA compon	95	15	6	9	centroid_2249	15.4236127	8.59081E-05	centroid_2249	0.00020111
centroid_4739	WGR domain protein	94	0	7	24	centroid_4739	85.1457799	2.77159E-20	centroid_4739	8.1685E-20
centroid_526	conserved hypothetical protein	94	0	7	24	centroid_526	85.1457799	2.77159E-20	centroid_526	8.1685E-20
centroid_15925	reverse transcriptase family protein	94	13	7	11	centroid_1592	20.7577289	5.21208E-06	centroid_1592	1.9423E-05
centroid_4181	reverse transcriptase family protein	94	13	7	11	centroid_4181	20.7577289	5.21208E-06	centroid_4181	1.9423E-05
centroid_7880	reverse transcriptase family protein	94	13	7	11	centroid_7880	20.7577289	5.21208E-06	centroid_7880	1.9423E-05
centroid_7881	reverse transcriptase family protein	94	13	7	11	centroid_7881	20.7577289	5.21208E-06	centroid_7881	1.9423E-05
centroid_7943	group II intron, maturase-specific domain protein	94	13	7	11	centroid_7943	20.7577289	5.21208E-06	centroid_7943	1.9423E-05
centroid_8104	reverse transcriptase family protein	94	13	7	11	centroid_8104	20.7577289	5.21208E-06	centroid_8104	1.9423E-05
centroid_8276	reverse transcriptase family protein	94	13	7	11	centroid_8276	20.7577289	5.21208E-06	centroid_8276	1.9423E-05
centroid_18647	istB-like ATP binding family protein	93	12	8	12	centroid_1864	22.5131191	2.08713E-06	centroid_1864	8.2732E-06
centroid_5141	integrase core domain protein	93	13	8	11	centroid_5141	18.7833333	1.46441E-05	centroid_5141	4.1053E-05
centroid_2894	oly-beta-1,6-N-acetyl-D-glucosamine N-deacetylase Pga	93	14	8	10	centroid_2894	15.2823468	9.25777E-05	centroid_2894	0.00018474
centroid_13091	mRNA interferase MqsR	92	4	9	20	centroid_1309	56.1765578	6.62467E-14	centroid_1309	1.0527E-12
centroid_2748	mRNA interferase MqsR	92	4	9	20	centroid_2748	56.1765578	6.62467E-14	centroid_2748	1.0527E-12
centroid_9316	protein PhnP	92	5	9	19	centroid_9316	51.0975528	8.78878E-13	centroid_9316	1.4167E-11
centroid_10926	gyrI-like small molecule binding domain protein	92	6	9	18	centroid_1092	46.1898917	1.07329E-11	centroid_1092	1.5804E-10
centroid_17190	bacterial dnaA family protein	92	12	9	12	centroid_1719	20.5756426	5.73208E-06	centroid_1719	1.7292E-05
centroid_9797	phage DNA packaging Nu1 family protein	92	13	9	11	centroid_9797	17.018708	3.70133E-05	centroid_9797	8.1118E-05
centroid_5142	integrase core domain protein	91	11	10	13	centroid_5142	22.4451248	2.16234E-06	centroid_5142	6.9087E-06
centroid_15969	CRISPR-associated endonuclease/helicase Cas3	90	2	11	22	centroid_1596	61.0271667	5.62926E-15	centroid_1596	2.5432E-14
centroid_7749	hypothetical protein	90	9	11	15	centroid_7749	28.2987483	1.03963E-07	centroid_7749	4.452E-07
centroid_6945	transposase DDE domain protein	90	10	11	14	centroid_6945	24.3947246	7.84833E-07	centroid_6945	2.6024E-06
centroid_8205	type VII secretion system (T7SS), usher family protein	90	10	11	14	centroid_8205	24.3947246	7.84833E-07	centroid_8205	2.6024E-06
centroid_9929	transposase family protein	90	10	11	14	centroid_9929	24.3947246	7.84833E-07	centroid_9929	2.6024E-06
centroid_15610	transposase family protein	90	11	11	13	centroid_1561	20.7032707	5.36243E-06	centroid_1561	1.3584E-05
centroid_6531	conserved hypothetical protein	90	11	11	13	centroid_6531	20.7032707	5.36243E-06	centroid_6531	1.3584E-05
centroid_10582	SPR-associated protein Cas6/Cse3/CasE, subtype I-E/E(	89	1	12	23	centroid_1058	63.6943438	1.45301E-15	centroid_1058	2.3459E-15
centroid_15998	SPR-associated endonuclease/helicase Cas3 domain pro	89	2	12	22	centroid_1599	58.376325	2.1648E-14	centroid_1599	7.0567E-14
centroid_2331	CRISPR-associated endonuclease Cas1	89	3	12	21	centroid_2331	53.2436099	2.94644E-13	centroid_2331	1.4096E-12
centroid_4611	SPR-associated endoribonuclease Cas2, subtype I-E/EC	89	3	12	21	centroid_4611	53.2436099	2.94644E-13	centroid_4611	1.4096E-12
centroid_9502	SPR-associated endoribonuclease Cas2, subtype I-E/EC	89	3	12	21	centroid_9502	53.2436099	2.94644E-13	centroid_9502	1.4096E-12
centroid_13465	conserved hypothetical protein	89	5	12	19	centroid_1346	43.5368392	4.16053E-11	centroid_1346	2.4884E-10
centroid_9932	rhs core with extension domain protein	89	5	12	19	centroid_9932	43.5368392	4.16053E-11	centroid_9932	2.4884E-10
centroid_5521	POTRA domain, ShlB-type family protein	89	10	12	14	centroid_5521	22.6591619	1.93436E-06	centroid_5521	5.1191E-06
centroid_17493	hypothetical protein	88	1	13	23	centroid_1749	61.106261	5.40759E-15	centroid_1749	6.4275E-15
centroid_17627	fimbrial subunit ElfA	88	2	13	22	centroid_1762	55.8773418	7.71365E-14	centroid_1762	1.8602E-13
centroid_9313	CRISPR-associated endonuclease/helicase Cas3	88	2	13	22	centroid_9313	55.8773418	7.71365E-14	centroid_9313	1.8602E-13
centroid_11130	conserved hypothetical protein	88	5	13	19	centroid_1113	41.335224	1.28237E-10	centroid_1113	5.8182E-10
centroid_3520	putative predicted protein	88	6	13	18	centroid_3520	36.8740998	1.26009E-09	centroid_3520	5.4655E-09
centroid_3521	conserved hypothetical protein	88	6	13	18	centroid_3521	36.8740998	1.26009E-09	centroid_3521	5.4655E-09
centroid_5163	putative domain protein	88	6	13	18	centroid_5163	36.8740998	1.26009E-09	centroid_5163	5.4655E-09
centroid_10619	conserved hypothetical protein	88	7	13	17	centroid_1061	32.6107755	1.12589E-08	centroid_1061	4.3545E-08
centroid_11183	transposase DDE domain protein	88	9	13	15	centroid_1118	24.6966593	6.71004E-07	centroid_1118	1.8078E-06
centroid_7910	hypothetical protein	88	9	13	15	centroid_7910	24.6966593	6.71004E-07	centroid_7910	1.8078E-06
centroid_3088	type II secretion system protein L	87	1	14	23	centroid_3088	58.6582135	1.87583E-14	centroid_3088	1.6805E-14
centroid_12408	RISPR-associated protein Cas5/CasD, subtype I-E/ECO	87	2	14	22	centroid_1240	53.5175749	2.5629E-13	centroid_1240	4.6823E-13
centroid_13690	CT1975-like family protein	87	2	14	22	centroid_1369	53.5175749	2.5629E-13	centroid_1369	4.6823E-13
centroid_16204	CRISPR type I-E/ECOLI-associated protein CasA/Cse1	87	2	14	22	centroid_1620	53.5175749	2.5629E-13	centroid_1620	4.6823E-13
centroid_2335	CRISPR type I-E/ECOLI-associated protein CasB/Cse2	87	2	14	22	centroid_2335	53.5175749	2.5629E-13	centroid_2335	4.6823E-13
centroid_5348	CT1975-like family protein	87	2	14	22	centroid_5348	53.5175749	2.5629E-13	centroid_5348	4.6823E-13
centroid_5358	SPR-associated protein Cas7/Cse4/CasC, subtype I-E/E(	87	2	14	22	centroid_5358	53.5175749	2.5629E-13	centroid_5358	4.6823E-13
centroid_4109	conserved hypothetical protein	87	8	14	16	centroid_4109	26.8207228	2.23231E-07	centroid_4109	5.9264E-07
centroid_18621	rhs element Vgr family protein	87	9	14	15	centroid_1862	23.0900898	1.54585E-06	centroid_1862	3.4235E-06
centroid_7904	surface antigen family protein	87	9	14	15	centroid_7904	23.0900898	1.54585E-06	centroid_7904	3.4235E-06
centroid_11415	conserved hypothetical protein	87	11	14	13	centroid_1141	16.2987755	5.40989E-05	centroid_1141	7.9458E-05
centroid_8132	conserved hypothetical protein	87	11	14	13	centroid_8132	16.2987755	5.40989E-05	centroid_8132	7.9458E-05
centroid_13505	RHS repeat-associated core domain protein	86	2	15	22	centroid_1350	51.2857491	7.98532E-13	centroid_1350	1.1303E-12
centroid_17647	CRISPR type I-E/ECOLI-associated protein CasA/Cse1	86	2	15	22	centroid_1764	51.2857491	7.98532E-13	centroid_1764	1.1303E-12
centroid_4613	CRISPR type I-E/ECOLI-associated protein CasA/Cse1	86	2	15	22	centroid_4613	51.2857491	7.98532E-13	centroid_4613	1.1303E-12
centroid_6137	CRISPR type I-E/ECOLI-associated protein CasA/Cse1	86	2	15	22	centroid_6137	51.2857491	7.98532E-13	centroid_6137	1.1303E-12
centroid_5556	hypothetical protein	86	7	15	17	centroid_5556	29.0367884	7.10168E-08	centroid_5556	1.7823E-07
centroid_12323	hypothetical protein	86	8	15	16	centroid_1232	25.2076695	5.14771E-07	centroid_1232	1.129E-06
centroid_13670	family 4 glycosyl hydrolase C-terminal domain protein	86	8	15	16	centroid_1367	25.2076695	5.14771E-07	centroid_1367	1.129E-06
centroid_4110	conserved hypothetical protein	86	8	15	16	centroid_4110	25.2076695	5.14771E-07	centroid_4110	1.129E-06
centroid_4439	family 4 glycosyl hydrolase C-terminal domain protein	86	8	15	16	centroid_4439	25.2076695	5.14771E-07	centroid_4439	1.129E-06
centroid_4977	conserved hypothetical protein	86	8	15	16	centroid_4977	25.2076695	5.14771E-07	centroid_4977	1.129E-06
centroid_12675	RHS repeat-associated core domain protein	86	10	15	14	centroid_1267	18.2093148	1.97909E-05	centroid_1267	3.06E-05
centroid_6733	transposase family protein	86	10	15	14	centroid_6733	18.2093148	1.97909E-05	centroid_6733	3.06E-05
centroid_6925	conserved hypothetical protein	86	10	15	14	centroid_6925	18.2093148	1.97909E-05	centroid_6925	3.06E-05
centroid_4612	RISPR-associated protein Cas5/CasD, subtype I-E/ECO	85	2	16	22	centroid_4612	49.1717768	2.34501E-12	centroid_4612	2.6266E-12
centroid_2084	6-phospho-alpha-glucosidase	85	8	16	16	centroid_2084	23.6998132	1.12593E-06	centroid_2084	2.0798E-06
centroid_4438	4 glycosyl hydrolase family protein	85	8	16	16	centroid_4438	23.6998132	1.12593E-06	centroid_4438	2.0798E-06
centroid_8296	hypothetical protein	85	9	16	15	centroid_8296	20.2039788	6.9608E-06	centroid_8296	1.1059E-05
centroid_15685	transposase family protein	85	10	16	14	centroid_1568	16.9369219	3.86426E-05	centroid_1568	5.1902E-05
centroid_11498	conserved hypothetical protein	84	8	17	16	centroid_1149	22.2877289	2.34702E-06	centroid_1149	3.7156E-06
centroid_9934	integrase core domain protein	84	9	17	15	centroid_9934	18.904333	1.3744E-05	centroid_9934	1.8983E-05
centroid_16849	hypothetical protein	83	0	18	24	centroid_1684	55.0731073	1.16129E-13	centroid_1684	1.0987E-14
centroid_2332	SPR-associated protein Cas6/Cse3/CasE, subtype I-E/E(	83	1	18	23	centroid_2332	50.0615376	1.48999E-12	centroid_2332	5.3209E-13
centroid_12681	RISPR-associated protein Cas5/CasD, subtype I-E/ECO	83	2	18	22	centroid_1268	45.2620888	1.72353E-11	centroid_1268	1.281E-11
centroid_9314	SPR-associated protein Cas6/Cse3/CasE, subtype I-E/E(	83	2	18	22	centroid_9314	45.2620888	1.72353E-11	centroid_9314	1.281E-11
centroid_4648	putative rhsC protein in rhs element	83	7	18	17	centroid_4648	24.4660906	7.56292E-07	centroid_4648	1.1467E-06
centroid_13709	POTRA domain, ShlB-type family protein	83	9	18	15	centroid_1370	17.6889335	2.60136E-05	centroid_1370	3.1705E-05
centroid_7156	putative transposase	82	5	19	19	centroid_7156	30.5943179	3.18024E-08	centroid_7156	4.2989E-08
centroid_10218	conserved hypothetical protein	82	6	19	18	centroid_1021	26.7451827	2.3213E-07	centroid_1021	3.2021E-07
centroid_9921	hypothetical protein	82	6	19	18	centroid_9921	26.7451827	2.3213E-07	centroid_9921	3.2021E-07
centroid_7022	putative transposase	82	7	19	17	centroid_7022	23.11857	1.52313E-06	centroid_7022	2.0157E-06
centroid_15206	hypothetical protein	82	8	19	16	centroid_1520	19.718592	8.97242E-06	centroid_1520	1.0923E-05
centroid_2333	RISPR-associated protein Cas5/CasD, subtype I-E/ECO	81	2	20	22	centroid_2333	41.7263149	1.04986E-10	centroid_2333	5.5476E-11
centroid_16544	rhs element Vgr family protein	81	5	20	19	centroid_1654	29.1317632	6.76192E-08	centroid_1654	7.9315E-08
centroid_12706	conserved hypothetical protein	81	6	20	18	centroid_1270	25.3767192	4.71575E-07	centroid_1270	5.6971E-07
centroid_10649	conserved hypothetical protein	81	7	20	17	centroid_1064	21.8473639	2.95222E-06	centroid_1064	3.4568E-06
centroid_5188	transposase DDE domain protein	80	0	21	24	centroid_5188	49.4233912	2.06271E-12	centroid_5188	1.1722E-13
centroid_15252	inner membrane YibH domain protein	80	5	21	19	centroid_1525	27.7442525	1.38459E-07	centroid_1525	1.4285E-07
centroid_12835	tn3 transposase DDE domain protein	79	8	22	16	centroid_1283	16.4038533	5.11811E-05	centroid_1283	4.6163E-05
centroid_580	biquinone/plastoquinone (complex I), various chains fami	78	0	23	24	centroid_580	46.0576857	1.14822E-11	centroid_580	5.0087E-13
centroid_14160	conserved domain protein	78	3	23	21	centroid_1416	32.8380965	1.00163E-08	centroid_1416	5.5511E-09
centroid_4158	phage integrase family protein	78	6	23	18	centroid_4158	21.6873478	3.20901E-06	centroid_4158	2.8036E-06
centroid_4230	conserved hypothetical protein	78	8	23	16	centroid_4230	15.4211317	8.6021E-05	centroid_4230	0.00011021
centroid_4848	tn3 transposase DDE domain protein	78	8	23	16	centroid_4848	15.4211317	8.6021E-05	centroid_4848	0.00011021
centroid_10333	tive pilus biosynthesis transmembrane anchor domain pro	77	3	24	21	centroid_1033	31.482144	2.01283E-08	centroid_1033	1.0046E-08
centroid_10334	tive pilus biosynthesis transmembrane anchor domain pro	77	3	24	21	centroid_1033	31.482144	2.01283E-08	centroid_1033	1.0046E-08
centroid_10335	hypothetical protein	77	3	24	21	centroid_1033	31.482144	2.01283E-08	centroid_1033	1.0046E-08

centroid_14026	type II/IV secretion system family protein	77	3	24	21	centroid_1402	31.482144	2.01283E-08	centroid_1402	1.0046E-08
centroid_4850	type IV leader peptidase family protein	77	3	24	21	centroid_485	31.482144	2.01283E-08	centroid_485	1.0046E-08
centroid_4851	putative pilus biosythesis protein	77	3	24	21	centroid_485	31.482144	2.01283E-08	centroid_485	1.0046E-08
centroid_4852	type II secretion system (T2SS), F family protein	77	3	24	21	centroid_485	31.482144	2.01283E-08	centroid_485	1.0046E-08
centroid_4853	type II/IV secretion system family protein	77	3	24	21	centroid_485	31.482144	2.01283E-08	centroid_485	1.0046E-08
centroid_4854	putative IngG	77	3	24	21	centroid_485	31.482144	2.01283E-08	centroid_485	1.0046E-08
centroid_4856	utative pilus biosynthesis transmembrane anchor protei	77	3	24	21	centroid_485	31.482144	2.01283E-08	centroid_485	1.0046E-08
centroid_4858	toxin co-regulated pilus biosynthesis Q family protein	77	3	24	21	centroid_485	31.482144	2.01283E-08	centroid_485	1.0046E-08
centroid_4859	repilin-type N-terminal cleavage/methylation domain prote	77	3	24	21	centroid_485	31.482144	2.01283E-08	centroid_485	1.0046E-08
centroid_4861	putative IngX2	77	3	24	21	centroid_486	31.482144	2.01283E-08	centroid_486	1.0046E-08
centroid_4862	transglycosylase SLT domain protein	77	3	24	21	centroid_486	31.482144	2.01283E-08	centroid_486	1.0046E-08
centroid_4863	bacterial regulatory helix-turn-helix, AraC family protein	77	3	24	21	centroid_486	31.482144	2.01283E-08	centroid_486	1.0046E-08
centroid_4864	dhesin biosynthesis transcription regulatory family protei	77	3	24	21	centroid_486	31.482144	2.01283E-08	centroid_486	1.0046E-08
centroid_8277	bacterial type II and III secretion system family protein	77	3	24	21	centroid_827	31.482144	2.01283E-08	centroid_827	1.0046E-08
centroid_4432	caudovirales tail fibre assembly family protein	77	4	24	20	centroid_443	27.6147732	1.48044E-07	centroid_443	9.6565E-08
centroid_4849	conserved hypothetical protein	77	4	24	20	centroid_484	27.6147732	1.48044E-07	centroid_484	9.6565E-08
centroid_8329	putative iS91 transposase	77	5	24	19	centroid_832	23.9802803	9.73275E-07	centroid_832	7.3366E-07
centroid_14802	bacterial Ig-like domain family protein	77	6	24	18	centroid_1480	20.5800253	5.71897E-06	centroid_1480	4.581E-06
centroid_16797	bacterial Ig-like domain family protein	77	6	24	18	centroid_1679	20.5800253	5.71897E-06	centroid_1679	4.581E-06
centroid_4917	conserved hypothetical protein	77	6	24	18	centroid_491	20.5800253	5.71897E-06	centroid_491	4.581E-06
centroid_4099	conserved hypothetical protein	77	7	24	17	centroid_409	17.4162461	3.00249E-05	centroid_409	4.4073E-05
centroid_4838	conserved hypothetical protein	77	7	24	17	centroid_483	17.4162461	3.00249E-05	centroid_483	4.4073E-05
centroid_4855	putative pilus biosynthesis protein	76	3	25	21	centroid_485	30.1859843	3.92536E-08	centroid_485	1.7832E-08
centroid_4857	bacterial type II and III secretion system family protein	76	3	25	21	centroid_485	30.1859843	3.92536E-08	centroid_485	1.7832E-08
centroid_17721	major Facilitator Superfamily protein	76	5	25	19	centroid_1772	22.8436074	1.75733E-06	centroid_1772	1.2179E-06
centroid_17722	major Facilitator Superfamily protein	76	5	25	19	centroid_1772	22.8436074	1.75733E-06	centroid_1772	1.2179E-06
centroid_3931	deoR-like helix-turn-helix domain protein	76	5	25	19	centroid_393	22.8436074	1.75733E-06	centroid_393	1.2179E-06
centroid_3932	ribokinase	76	5	25	19	centroid_393	22.8436074	1.75733E-06	centroid_393	1.2179E-06
centroid_3934	putative monosaccharide-transporting ATPase	76	5	25	19	centroid_393	22.8436074	1.75733E-06	centroid_393	1.2179E-06
centroid_7843	hage tail tape measure protein, TP901 family, core regior	76	5	25	19	centroid_784	22.8436074	1.75733E-06	centroid_784	1.2179E-06
centroid_18668	putative membrane protein	76	7	25	17	centroid_1866	16.4491395	4.99729E-05	centroid_1866	5.167E-05
centroid_4397	ftsK/SpoIIIE family protein	75	1	26	23	centroid_439	37.0851248	1.13083E-09	centroid_439	1.397E-10
centroid_3933	H+ symporter permease	75	5	26	19	centroid_393	21.759492	3.09057E-06	centroid_393	1.9867E-06
centroid_10044	utative DNA-binding transcriptional regulator domain prote	75	6	26	18	centroid_1004	18.5245991	1.67726E-05	centroid_1004	1.8759E-05
centroid_11968	bacterial Ig-like domain family protein	75	6	26	18	centroid_1196	18.5245991	1.67726E-05	centroid_1196	1.8759E-05
centroid_12473	bacterial Ig-like domain family protein	75	6	26	18	centroid_1247	18.5245991	1.67726E-05	centroid_1247	1.8759E-05
centroid_14668	hypothetical protein	74	1	27	23	centroid_1466	35.755724	2.23674E-09	centroid_1466	2.5549E-10
centroid_15981	H+ symporter family protein	74	1	27	23	centroid_1598	35.755724	2.23674E-09	centroid_1598	2.5549E-10
centroid_16661	toxin YafO, type II toxin-antitoxin system family protein	74	1	27	23	centroid_1666	35.755724	2.23674E-09	centroid_1666	2.5549E-10
centroid_17455	conserved hypothetical protein	74	1	27	23	centroid_1745	35.755724	2.23674E-09	centroid_1745	2.5549E-10
centroid_2822	antitoxin YafN	74	1	27	23	centroid_282	35.755724	2.23674E-09	centroid_282	2.5549E-10
centroid_4699	conserved hypothetical protein	74	1	27	23	centroid_469	35.755724	2.23674E-09	centroid_469	2.5549E-10
centroid_7700	mRNA interferase HigB	74	1	27	23	centroid_770	35.755724	2.23674E-09	centroid_770	2.5549E-10
centroid_2334	SPR-associated protein Cas7/Cse4/CasC, subtype I-E/E(	74	2	27	22	centroid_233	31.6361793	1.85933E-08	centroid_233	4.5397E-09
centroid_5525	conserved hypothetical protein	74	4	27	20	centroid_552	24.1210352	9.04667E-07	centroid_552	4.6366E-07
centroid_9765	aatD, apolipoN-acyltransferase domain protein	74	5	27	19	centroid_976	20.7245822	5.30308E-06	centroid_976	3.188E-06
centroid_18009	bacterial Ig-like domain family protein	74	6	27	18	centroid_1800	17.5696199	2.76978E-05	centroid_1800	2.3044E-05
centroid_10353	orn/Lys/Arg decarboxylase, major domain protein	73	0	28	24	centroid_1035	38.7764089	4.75237E-10	centroid_1035	1.3245E-11
centroid_12500	ornithine decarboxylase, inducible domain protein	73	0	28	24	centroid_1250	38.7764089	4.75237E-10	centroid_1250	1.3245E-11
centroid_1484	conserved hypothetical protein	73	0	28	24	centroid_148	38.7764089	4.75237E-10	centroid_148	1.3245E-11
centroid_16491	orn/Lys/Arg decarboxylase, N-terminal domain protein	73	0	28	24	centroid_1649	38.7764089	4.75237E-10	centroid_1649	1.3245E-11
centroid_11286	D-serine deaminase transcriptional activator	73	1	28	23	centroid_1128	34.4786252	4.30958E-09	centroid_1128	4.595E-10
centroid_1251	dsdX permease	73	1	28	23	centroid_125	34.4786252	4.30958E-09	centroid_125	4.595E-10
centroid_2823	mRNA interferase YafO	73	1	28	23	centroid_282	34.4786252	4.30958E-09	centroid_282	4.595E-10
centroid_6217	lasmic binding and sugar binding domain of LacI family pr	73	2	28	22	centroid_621	30.4270661	3.46657E-08	centroid_621	7.9059E-09
centroid_733	periplasmic binding domain protein	73	2	28	22	centroid_733	30.4270661	3.46657E-08	centroid_733	7.9059E-09
centroid_734	D-allose transporter subunit domain protein	73	2	28	22	centroid_734	30.4270661	3.46657E-08	centroid_734	7.9059E-09
centroid_735	ABC transporter family protein	73	2	28	22	centroid_735	30.4270661	3.46657E-08	centroid_735	7.9059E-09
centroid_9859	hypothetical protein	73	2	28	22	centroid_985	30.4270661	3.46657E-08	centroid_985	7.9059E-09
centroid_732	HTH-type transcriptional regulator RpiR	73	4	28	20	centroid_732	23.0562991	1.57326E-06	centroid_732	7.568E-07
centroid_13568	putative aatD, apolipoprotein N-acyltransferase	73	5	28	19	centroid_1356	19.7358151	8.89192E-06	centroid_1356	7.4927E-06
centroid_14630	ribose 5-phosphate isomerase B	73	5	28	19	centroid_1463	19.7358151	8.89192E-06	centroid_1463	7.4927E-06
centroid_5879	ABC transporter family protein	73	5	28	19	centroid_587	19.7358151	8.89192E-06	centroid_587	7.4927E-06
centroid_6218	heme ABC exporter, ATP-binding protein CcmA	73	5	28	19	centroid_621	19.7358151	8.89192E-06	centroid_621	7.4927E-06
centroid_731	ribose 5-phosphate isomerase B	73	5	28	19	centroid_731	19.7358151	8.89192E-06	centroid_731	7.4927E-06
centroid_736	ain amino acid transport system / permease component	73	5	28	19	centroid_736	19.7358151	8.89192E-06	centroid_736	7.4927E-06
centroid_737	D-allulose-6-phosphate 3-epimerase	73	5	28	19	centroid_737	19.7358151	8.89192E-06	centroid_737	7.4927E-06
centroid_738	D-allose kinase	73	5	28	19	centroid_738	19.7358151	8.89192E-06	centroid_738	7.4927E-06
centroid_9256	ulp1 protease family, C-terminal catalytic domain protein	73	5	28	19	centroid_925	19.7358151	8.89192E-06	centroid_925	7.4927E-06
centroid_12709	bacterial regulatory helix-turn-helix, AraC family protein	73	6	28	18	centroid_1270	16.6590528	4.47363E-05	centroid_1270	3.1056E-05
centroid_14191	CFA/I fimbrial subunit D domain protein	73	6	28	18	centroid_1419	16.6590528	4.47363E-05	centroid_1419	3.1056E-05
centroid_17756	conserved hypothetical protein	72	2	29	22	centroid_1775	29.2658374	6.30984E-08	centroid_1775	1.3555E-08
centroid_14468	putative aatD, apolipoprotein N-acyltransferase	72	3	29	21	centroid_1446	25.5281388	4.35978E-07	centroid_1446	1.4913E-07
centroid_2426	conserved hypothetical protein	72	4	29	20	centroid_242	22.0364806	2.67517E-06	centroid_242	1.2168E-06
centroid_270	conserved hypothetical protein	72	4	29	20	centroid_270	22.0364806	2.67517E-06	centroid_270	1.2168E-06
centroid_4203	CFA/I fimbrial subunit D	72	6	29	18	centroid_420	15.7901719	7.07692E-05	centroid_420	4.3945E-05
centroid_16443	D-serine deaminase transcriptional activator	71	1	30	23	centroid_1644	32.0695183	1.48753E-08	centroid_1644	1.4185E-09
centroid_15730	putative membrane protein	71	3	30	21	centroid_1573	24.4800473	7.50834E-07	centroid_1573	2.4403E-07
centroid_16886	orn/Lys/Arg decarboxylase, major domain protein	71	3	30	21	centroid_1688	24.4800473	7.50834E-07	centroid_1688	2.4403E-07
centroid_4206	putative membrane protein	71	3	30	21	centroid_420	24.4800473	7.50834E-07	centroid_420	2.4403E-07
centroid_8347	aatD, apolipoN-acyltransferase domain protein	71	3	30	21	centroid_834	24.4800473	7.50834E-07	centroid_834	2.4403E-07
centroid_11294	porin, autotransporter (AT) family	71	4	30	20	centroid_1129	21.0589418	4.4537E-06	centroid_1129	2.7294E-06
centroid_13494	e ATP-binding component of a transport system domain	71	4	30	20	centroid_1349	21.0589418	4.4537E-06	centroid_1349	2.7294E-06
centroid_17797	conserved hypothetical protein	71	4	30	20	centroid_1779	21.0589418	4.4537E-06	centroid_1779	2.7294E-06
centroid_1911	outer membrane autotransporter barrel domain protein	71	4	30	20	centroid_191	21.0589418	4.4537E-06	centroid_191	2.7294E-06
centroid_4111	transposase for ISEc12 domain protein	71	4	30	20	centroid_411	21.0589418	4.4537E-06	centroid_411	2.7294E-06
centroid_8275	hage tail tape measure protein, TP901 family, core regior	71	5	30	19	centroid_827	17.8857273	2.34574E-05	centroid_827	1.3206E-05
centroid_10799	-negative pili assembly chaperone, C-terminal domain pr	70	0	31	24	centroid_1079	35.0433202	3.22451E-09	centroid_1079	7.7305E-11
centroid_16913	putative membrane protein	70	0	31	24	centroid_1691	35.0433202	3.22451E-09	centroid_1691	7.7305E-11
centroid_1250	D-serine deaminase transcriptional activator	70	1	31	23	centroid_125	30.9321508	2.67208E-08	centroid_125	2.4386E-09
centroid_1239	-negative pili assembly chaperone, N-terminal domain pr	69	0	32	24	centroid_123	33.8878812	5.83812E-09	centroid_123	1.3528E-10
centroid_16914	-negative pili assembly chaperone, N-terminal domain pr	69	0	32	24	centroid_1691	33.8878812	5.83812E-09	centroid_1691	1.3528E-10
centroid_4811	putative 4'-phosphopantetheinyl transferase EntD	69	0	32	24	centroid_481	33.8878812	5.83812E-09	centroid_481	1.3528E-10
centroid_1092	mRNA interferase HigB	69	1	32	23	centroid_109	29.8363263	4.70101E-08	centroid_109	4.1358E-09
centroid_13618	rhs element Vgr family protein	68	4	33	20	centroid_1361	18.3566568	1.83178E-05	centroid_1361	1.3752E-05
centroid_4752	putative dNA-binding transcriptional regulator	68	4	33	20	centroid_475	18.3566568	1.83178E-05	centroid_475	1.3752E-05
centroid_10045	putative nucleic acid-binding protein	68	5	33	19	centroid_1004	15.3936825	8.72796E-05	centroid_1004	4.9834E-05
centroid_13984	helix-turn-helix domain protein	68	5	33	19	centroid_1398	15.3936825	8.72796E-05	centroid_1398	4.9834E-05
centroid_15675	putative nucleic acid-binding protein	68	5	33	19	centroid_1567	15.3936825	8.72796E-05	centroid_1567	4.9834E-05
centroid_3263	conserved hypothetical protein	68	5	33	19	centroid_326	15.3936825	8.72796E-05	centroid_326	4.9834E-05
centroid_15359	conserved hypothetical protein	66	4	35	20	centroid_1535	16.7267564	4.31679E-05	centroid_1535	1.9088E-05
centroid_1175	von Willebrand factor family protein	65	0	36	24	centroid_117	29.6514853	5.1713E-08	centroid_117	1.1199E-09
centroid_6930	conserved hypothetical protein	65	0	36	24	centroid_693	29.6514853	5.1713E-08	centroid_693	1.1199E-09
centroid_3558	caudovirales tail fibre assembly family protein	65	1	36	23	centroid_355	25.8263677	3.7355E-07	centroid_355	4.9635E-08
centroid_1176	peptidase M20/M25/M40 family protein	65	4	36	20	centroid_117	15.9579777	6.47643E-05	centroid_117	2.5617E-05
centroid_10249	conserved hypothetical protein	64	0	37	24	centroid_1024	28.6792552	8.54138E-08	centroid_1024	1.8464E-09
centroid_1179	UBA/TS-N domain protein	64	0	37	24	centroid_117	28.6792552	8.54138E-08	centroid_117	1.8464E-09

centroid_1180	conserved hypothetical protein	64	0	37	24	centroid_118	28.6792552	8.54138E-08	centroid_118	1.8464E-09
centroid_1181	tetratricopeptide repeat family protein	64	0	37	24	centroid_118	28.6792552	8.54138E-08	centroid_118	1.8464E-09
centroid_2946	-negative pili assembly chaperone, C-terminal domain pr	64	0	37	24	centroid_294	28.6792552	8.54138E-08	centroid_294	1.8464E-09
centroid_4219	hypothetical protein	64	3	37	21	centroid_421	18.1809862	2.00874E-05	centroid_421	6.3655E-06
centroid_9677	putative transposase	64	3	37	21	centroid_967	18.1809862	2.00874E-05	centroid_967	6.3655E-06
centroid_14287	autotransporter beta-domain protein	64	4	37	20	centroid_1428	15.2179169	9.57903E-05	centroid_1428	5.5542E-05
centroid_1235	conserved hypothetical protein	63	0	38	24	centroid_123	27.7384122	1.38878E-07	centroid_123	3.0125E-09
centroid_1236	fimbrial family protein	63	0	38	24	centroid_123	27.7384122	1.38878E-07	centroid_123	3.0125E-09
centroid_1237	fimbrial family protein	63	0	38	24	centroid_123	27.7384122	1.38878E-07	centroid_123	3.0125E-09
centroid_1238	fimbrial family protein	63	0	38	24	centroid_123	27.7384122	1.38878E-07	centroid_123	3.0125E-09
centroid_13865	fimbrial family protein	63	0	38	24	centroid_1386	27.7384122	1.38878E-07	centroid_1386	3.0125E-09
centroid_14007	RhsB domain protein	63	1	38	23	centroid_1400	24.0198057	9.53498E-07	centroid_1400	8.4264E-08
centroid_17771	hypothetical protein	63	1	38	23	centroid_1777	24.0198057	9.53498E-07	centroid_1777	8.4264E-08
centroid_373	conserved hypothetical protein	63	1	38	23	centroid_373	24.0198057	9.53498E-07	centroid_373	8.4264E-08
centroid_8305	RHS repeat-associated core domain protein	63	1	38	23	centroid_830	24.0198057	9.53498E-07	centroid_830	8.4264E-08
centroid_9920	RHS repeat-associated core domain protein	63	1	38	23	centroid_992	24.0198057	9.53498E-07	centroid_992	8.4264E-08
centroid_12832	conserved hypothetical protein	63	3	38	21	centroid_1283	17.407226	3.01677E-05	centroid_1283	8.6631E-06
centroid_10911	tnsA endonuclease N terminal family protein	62	1	39	23	centroid_1091	23.1605615	1.49022E-06	centroid_1091	1.2467E-07
centroid_11449	putative tn7-like transposition protein C	62	1	39	23	centroid_1144	23.1605615	1.49022E-06	centroid_1144	1.2467E-07
centroid_4049	tniQ family protein	62	1	39	23	centroid_404	23.1605615	1.49022E-06	centroid_404	1.2467E-07
centroid_14128	e kinase-, DNA gyrase B-, and HSP90-like ATPase family	62	2	39	22	centroid_1412	19.7731352	8.71997E-06	centroid_1412	1.7487E-06
centroid_17103	CRISPR type I-E/ECOLI-associated protein CasA/Cse1	62	2	39	22	centroid_1710	19.7731352	8.71997E-06	centroid_1710	1.7487E-06
centroid_3474	putative copper-binding protein PcoE	62	2	39	22	centroid_347	19.7731352	8.71997E-06	centroid_347	1.7487E-06
centroid_17155	helix-turn-helix domain protein	61	0	40	24	centroid_1715	25.945007	3.51283E-07	centroid_1715	9.6325E-09
centroid_3475	heavy metal sensor kinase family protein	61	1	40	23	centroid_347	22.3288251	2.29732E-06	centroid_347	3.057E-07
centroid_3486	cation efflux system protein CusA	61	1	40	23	centroid_348	22.3288251	2.29732E-06	centroid_348	3.057E-07
centroid_3487	efflux transporter, RND family, MFP subunit	61	1	40	23	centroid_348	22.3288251	2.29732E-06	centroid_348	3.057E-07
centroid_3488	cation efflux system protein CusF	61	1	40	23	centroid_348	22.3288251	2.29732E-06	centroid_348	3.057E-07
centroid_3489	nsporter, outer membrane factor (OMF) lipo, NodT famil	61	1	40	23	centroid_348	22.3288251	2.29732E-06	centroid_348	3.057E-07
centroid_3490	transcriptional regulatory protein CusR	61	1	40	23	centroid_349	22.3288251	2.29732E-06	centroid_349	3.057E-07
centroid_3491	heavy metal sensor kinase family protein	61	1	40	23	centroid_349	22.3288251	2.29732E-06	centroid_349	3.057E-07
centroid_10828	putative metalloprotease YebA domain protein	61	2	40	22	centroid_1082	18.9952784	1.31042E-05	centroid_1082	2.3875E-06
centroid_10928	peptidase M23 family protein	61	2	40	22	centroid_1092	18.9952784	1.31042E-05	centroid_1092	2.3875E-06
centroid_12338	conserved hypothetical protein	61	2	40	22	centroid_1233	18.9952784	1.31042E-05	centroid_1233	2.3875E-06
centroid_18160	putative pcoS	61	2	40	22	centroid_1816	18.9952784	1.31042E-05	centroid_1816	2.3875E-06
centroid_18161	efflux transporter, RND family, MFP subunit	61	2	40	22	centroid_1816	18.9952784	1.31042E-05	centroid_1816	2.3875E-06
centroid_18179	heavy metal sensor kinase family protein	61	2	40	22	centroid_1817	18.9952784	1.31042E-05	centroid_1817	2.3875E-06
centroid_3476	response regulator	61	2	40	22	centroid_347	18.9952784	1.31042E-05	centroid_347	2.3875E-06
centroid_3477	copper resistance D family protein	61	2	40	22	centroid_347	18.9952784	1.31042E-05	centroid_347	2.3875E-06
centroid_3478	copper resistance protein C	61	2	40	22	centroid_347	18.9952784	1.31042E-05	centroid_347	2.3875E-06
centroid_3479	copper resistance protein B	61	2	40	22	centroid_347	18.9952784	1.31042E-05	centroid_347	2.3875E-06
centroid_3480	copper resistance protein A	61	2	40	22	centroid_348	18.9952784	1.31042E-05	centroid_348	2.3875E-06
centroid_3481	putative copper resistant protein PcoE	61	2	40	22	centroid_348	18.9952784	1.31042E-05	centroid_348	2.3875E-06
centroid_3483	conserved hypothetical protein	61	2	40	22	centroid_348	18.9952784	1.31042E-05	centroid_348	2.3875E-06
centroid_3484	copper-translocating P-type ATPase	61	2	40	22	centroid_348	18.9952784	1.31042E-05	centroid_348	2.3875E-06
centroid_4847	conserved hypothetical protein	61	3	40	21	centroid_484	15.9392437	6.54084E-05	centroid_484	2.2075E-05
centroid_1178	conserved hypothetical protein	60	0	41	24	centroid_117	25.089731	5.47235E-07	centroid_117	1.3464E-08
centroid_3323	integrase core domain protein	60	0	41	24	centroid_332	25.089731	5.47235E-07	centroid_332	1.3464E-08
centroid_4332	calcineurin-like phosphoesterase family protein	60	0	41	24	centroid_433	25.089731	5.47235E-07	centroid_433	1.3464E-08
centroid_6506	hypothetical protein	60	0	41	24	centroid_650	25.089731	5.47235E-07	centroid_650	1.3464E-08
centroid_7671	conserved hypothetical protein	60	0	41	24	centroid_767	25.089731	5.47235E-07	centroid_767	1.3464E-08
centroid_10929	conserved hypothetical protein	60	1	41	23	centroid_1092	21.5233182	3.49552E-06	centroid_1092	3.6229E-07
centroid_15430	tnsB domain protein	60	1	41	23	centroid_1543	21.5233182	3.49552E-06	centroid_1543	3.6229E-07
centroid_3324	tnsA endonuclease N terminal family protein	60	1	41	23	centroid_332	21.5233182	3.49552E-06	centroid_332	3.6229E-07
centroid_4047	putative tnsB domain protein	60	1	41	23	centroid_404	21.5233182	3.49552E-06	centroid_404	3.6229E-07
centroid_6507	conserved hypothetical protein	60	1	41	23	centroid_650	21.5233182	3.49552E-06	centroid_650	3.6229E-07
centroid_6593	conserved hypothetical protein	60	1	41	23	centroid_659	21.5233182	3.49552E-06	centroid_659	3.6229E-07
centroid_4357	putative pantothenate kinase Pantothenic acid kinase	60	3	41	21	centroid_435	15.242563	9.45483E-05	centroid_435	2.8199E-05
centroid_4860	CFA/III pilin	59	0	42	24	centroid_486	24.2604007	8.41514E-07	centroid_486	2.0069E-08
centroid_864	putative membrane protein	59	0	42	24	centroid_864	24.2604007	8.41514E-07	centroid_864	2.0069E-08
centroid_17486	silver exporting P-type ATPase domain protein	59	1	42	23	centroid_1748	20.742842	5.25276E-06	centroid_1748	4.839E-07
centroid_14725	o-4-hydroxy-6-hydroxymethyldihydropteridine diphosphc	59	2	42	22	centroid_1472	17.5144084	2.85139E-05	centroid_1472	6.3162E-06
centroid_15907	conserved hypothetical protein	59	2	42	22	centroid_1590	17.5144084	2.85139E-05	centroid_1590	6.3162E-06
centroid_16583	CRISPR type I-E/ECOLI-associated protein CasA/Cse1	59	2	42	22	centroid_1658	17.5144084	2.85139E-05	centroid_1658	6.3162E-06
centroid_3482	peptidase M23 family protein	59	2	42	22	centroid_348	17.5144084	2.85139E-05	centroid_348	6.3162E-06
centroid_10746	putative ybl54	58	0	43	24	centroid_1074	23.4558559	1.27813E-06	centroid_1074	4.8936E-08
centroid_15898	putative predicted protein	58	0	43	24	centroid_1589	23.4558559	1.27813E-06	centroid_1589	4.8936E-08
centroid_17655	conserved hypothetical protein	58	0	43	24	centroid_1765	23.4558559	1.27813E-06	centroid_1765	4.8936E-08
centroid_8703	putative ybl54	58	0	43	24	centroid_870	23.4558559	1.27813E-06	centroid_870	4.8936E-08
centroid_15801	RHS repeat-associated core domain protein	58	1	43	23	centroid_1580	19.986271	7.80002E-06	centroid_1580	1.2857E-06
centroid_4048	AAA domain protein	58	1	43	23	centroid_404	19.986271	7.80002E-06	centroid_404	1.2857E-06
centroid_7896	RHS repeat-associated core domain protein	58	1	43	23	centroid_789	19.986271	7.80002E-06	centroid_789	1.2857E-06
centroid_2336	CRISPR type I-E/ECOLI-associated protein CasA/Cse1	58	2	43	22	centroid_233	16.8091554	4.13334E-05	centroid_233	8.0764E-06
centroid_2780	flagellar biosynthesis protein FlhA	58	2	43	22	centroid_278	16.8091554	4.13334E-05	centroid_278	8.0764E-06
centroid_16888	conserved hypothetical protein	57	0	44	24	centroid_1688	22.6750045	1.91847E-06	centroid_1688	5.816E-08
centroid_7913	hypothetical protein	57	0	44	24	centroid_791	22.6750045	1.91847E-06	centroid_791	5.816E-08
centroid_16884	outer membrane autotransporter barrel domain protein	57	1	44	23	centroid_1688	19.2525478	1.14517E-05	centroid_1688	1.3912E-06
centroid_2779	conserved hypothetical protein	55	0	46	24	centroid_277	21.1803267	4.18034E-06	centroid_277	2.1071E-07
centroid_13662	ATP-dependent endonuclease, OLD family domain protei	55	1	46	23	centroid_1366	17.849726	2.39054E-05	centroid_1366	2.3273E-06
centroid_15488	outer membrane autotransporter barrel domain protein	55	1	46	23	centroid_1548	17.849726	2.39054E-05	centroid_1548	2.3273E-06
centroid_4051	helix-turn-helix family protein	55	1	46	23	centroid_405	17.849726	2.39054E-05	centroid_405	2.3273E-06
centroid_7633	conserved hypothetical protein	55	1	46	23	centroid_763	17.849726	2.39054E-05	centroid_763	2.3273E-06
centroid_374	RHS repeat-associated core domain protein	54	0	47	24	centroid_374	20.4646158	6.07439E-06	centroid_374	2.2733E-07
centroid_4050	conserved hypothetical protein	54	1	47	23	centroid_405	17.1788105	3.4021E-05	centroid_405	4.703E-06
centroid_4052	AAA ATPase domain protein	54	1	47	23	centroid_405	17.1788105	3.4021E-05	centroid_405	4.703E-06
centroid_4599	fimbrial family protein	53	0	48	24	centroid_459	19.7688214	8.73968E-06	centroid_459	2.8511E-07
centroid_11599	TS system, galactitol-specific IIC component family prote	53	24	48	0	centroid_1159	16.561509	4.70973E-05	centroid_1159	2.4572E-06
centroid_4303	galactitol-1-phosphate 5-dehydrogenase	53	24	48	0	centroid_430	16.561509	4.70973E-05	centroid_430	2.4572E-06
centroid_4304	bacterial regulatory, gntR family protein	53	24	48	0	centroid_430	16.561509	4.70973E-05	centroid_430	2.4572E-06
centroid_7704	putative lipid A biosynthesis protein	53	24	48	0	centroid_770	16.561509	4.70973E-05	centroid_770	2.4572E-06
centroid_8672	utative aDP-heptose:LPS heptosyltransferase-like protei	53	24	48	0	centroid_867	16.561509	4.70973E-05	centroid_867	2.4572E-06
centroid_3492	silver-binding protein SilE	53	1	48	23	centroid_349	16.5271011	4.79597E-05	centroid_349	5.5966E-06
centroid_3493	conserved hypothetical protein	53	1	48	23	centroid_349	16.5271011	4.79597E-05	centroid_349	5.5966E-06
centroid_5014	helix-turn-helix family protein	52	0	49	24	centroid_501	19.0921268	1.24558E-05	centroid_501	3.9083E-07
centroid_5015	conserved hypothetical protein	52	0	49	24	centroid_501	19.0921268	1.24558E-05	centroid_501	3.9083E-07
centroid_13033	RHS repeat-associated core domain protein	52	1	49	23	centroid_1303	15.8938147	6.69973E-05	centroid_1303	7.2852E-06
centroid_10608	transposase DDE domain protein	51	0	50	24	centroid_1060	18.43376	1.75914E-05	centroid_1060	7.8857E-07
centroid_13558	transposase DDE domain protein	51	0	50	24	centroid_1355	18.43376	1.75914E-05	centroid_1355	7.8857E-07
centroid_16152	putative iS903 transposase	51	0	50	24	centroid_1615	18.43376	1.75914E-05	centroid_1615	7.8857E-07
centroid_3748	conserved hypothetical protein	51	0	50	24	centroid_374	18.43376	1.75914E-05	centroid_374	7.8857E-07
centroid_3749	helix-turn-helix domain protein	51	0	50	24	centroid_374	18.43376	1.75914E-05	centroid_374	7.8857E-07
centroid_7784	cytidylyltransferase family protein	51	24	50	0	centroid_778	17.7929902	2.46289E-05	centroid_778	9.4992E-07
centroid_1579	RHS repeat-associated core domain protein	51	1	50	23	centroid_157	15.2782123	9.27805E-05	centroid_157	1.4205E-05
centroid_4440	bacterial regulatory helix-turn-helix, AraC family protein	50	24	51	0	centroid_444	18.43376	1.75914E-05	centroid_444	7.8857E-07
centroid_4441	conserved hypothetical protein	50	24	51	0	centroid_444	18.43376	1.75914E-05	centroid_444	7.8857E-07
centroid_4442	sulfatase family protein	50	24	51	0	centroid_444	18.43376	1.75914E-05	centroid_444	7.8857E-07
centroid_4443	inner membrane protein YdI	50	24	51	0	centroid_444	18.43376	1.75914E-05	centroid_444	7.8857E-07

centroid_6460	sulfatase family protein	50	24	51	0	centroid_646	18.43376	1.75914E-05	centroid_646	7.8857E-07
centroid_6461	sulfatase family protein	50	24	51	0	centroid_646	18.43376	1.75914E-05	centroid_646	7.8857E-07
centroid_8995	conserved hypothetical protein	50	24	51	0	centroid_899	18.43376	1.75914E-05	centroid_899	7.8857E-07
centroid_8996	sodium:solute symporter family protein	50	24	51	0	centroid_899	18.43376	1.75914E-05	centroid_899	7.8857E-07
centroid_9344	utative DNA-binding transcriptional regulator domain prote	50	24	51	0	centroid_934	18.43376	1.75914E-05	centroid_934	7.8857E-07
centroid_9345	bacterial regulatory helix-turn-helix, AraC family protein	50	24	51	0	centroid_934	18.43376	1.75914E-05	centroid_934	7.8857E-07
centroid_15320	gram-negative porin family protein	50	0	51	24	centroid_1532	17.7929902	2.46289E-05	centroid_1532	9.4992E-07
centroid_17666	putative phage tail fiber domain protein	50	0	51	24	centroid_1766	17.7929902	2.46289E-05	centroid_1766	9.4992E-07
centroid_3223	outer membrane porin protein OmpD	50	0	51	24	centroid_322	17.7929902	2.46289E-05	centroid_322	9.4992E-07
centroid_4232	transposase DDE domain protein	50	0	51	24	centroid_423	17.7929902	2.46289E-05	centroid_423	9.4992E-07
centroid_4078	chaperone of endosialidase family protein	49	0	52	24	centroid_407	17.1691252	3.41949E-05	centroid_407	2.3872E-06
centroid_4102	outer membrane porin protein OmpD	49	0	52	24	centroid_410	17.1691252	3.41949E-05	centroid_410	2.3872E-06
centroid_8308	autotransporter beta-domain protein	49	0	52	24	centroid_830	17.1691252	3.41949E-05	centroid_830	2.3872E-06
centroid_4541	glycosyltransferase 9 family protein	48	24	53	0	centroid_454	19.7688214	8.73968E-06	centroid_454	2.8511E-07
centroid_7194	ftsK/SpoIIIE family protein	48	24	53	0	centroid_719	19.7688214	8.73968E-06	centroid_719	2.8511E-07
centroid_9248	ftsK/SpoIIIE family protein	48	24	53	0	centroid_924	19.7688214	8.73968E-06	centroid_924	2.8511E-07
centroid_3274	lysR substrate binding domain protein	48	0	53	24	centroid_327	16.561509	4.70973E-05	centroid_327	2.4572E-06
centroid_3275	aldo/keto reductase family protein	48	0	53	24	centroid_327	16.561509	4.70973E-05	centroid_327	2.4572E-06
centroid_3276	enoyl-(Acyl carrier) reductase family protein	48	0	53	24	centroid_327	16.561509	4.70973E-05	centroid_327	2.4572E-06
centroid_3277	yketide cyclase / dehydrase and lipid transport family pro	48	0	53	24	centroid_327	16.561509	4.70973E-05	centroid_327	2.4572E-06
centroid_3278	conserved hypothetical protein	48	0	53	24	centroid_327	16.561509	4.70973E-05	centroid_327	2.4572E-06
centroid_4344	conserved hypothetical protein	48	0	53	24	centroid_434	16.561509	4.70973E-05	centroid_434	2.4572E-06
centroid_4662	cytidylyltransferase family protein	47	24	54	0	centroid_466	20.4646158	6.07439E-06	centroid_466	2.2733E-07
centroid_4663	CDP-alcohol phosphatidyltransferase family protein	47	23	54	1	centroid_466	17.1788105	3.4021E-05	centroid_466	4.703E-06
centroid_10276	major Facilitator Superfamily protein	47	0	54	24	centroid_1027	15.9695194	6.43706E-05	centroid_1027	2.9197E-06
centroid_10277	major Facilitator Superfamily protein	47	0	54	24	centroid_1027	15.9695194	6.43706E-05	centroid_1027	2.9197E-06
centroid_10419	RHS repeat-associated core domain protein	47	0	54	24	centroid_1041	15.9695194	6.43706E-05	centroid_1041	2.9197E-06
centroid_1524	HAD hydrolase, IA, variant 3 family protein	47	0	54	24	centroid_152	15.9695194	6.43706E-05	centroid_152	2.9197E-06
centroid_1525	polyol permease family protein	47	0	54	24	centroid_152	15.9695194	6.43706E-05	centroid_152	2.9197E-06
centroid_1526	xylulokinase	47	0	54	24	centroid_152	15.9695194	6.43706E-05	centroid_152	2.9197E-06
centroid_1527	mannitol dehydrogenase C-terminal domain protein	47	0	54	24	centroid_152	15.9695194	6.43706E-05	centroid_152	2.9197E-06
centroid_1528	putative sugar-binding domain protein	47	0	54	24	centroid_152	15.9695194	6.43706E-05	centroid_152	2.9197E-06
centroid_1529	helix-turn-helix family protein	47	0	54	24	centroid_152	15.9695194	6.43706E-05	centroid_152	2.9197E-06
centroid_1530	ribitol 2-dehydrogenase	47	0	54	24	centroid_153	15.9695194	6.43706E-05	centroid_153	2.9197E-06
centroid_1531	FGGY-pentulose kinase family protein	47	0	54	24	centroid_153	15.9695194	6.43706E-05	centroid_153	2.9197E-06
centroid_4199	putative transposase	47	0	54	24	centroid_419	15.9695194	6.43706E-05	centroid_419	2.9197E-06
centroid_5278	major Facilitator Superfamily protein	47	0	54	24	centroid_527	15.9695194	6.43706E-05	centroid_527	2.9197E-06
centroid_5369	major Facilitator Superfamily protein	47	0	54	24	centroid_536	15.9695194	6.43706E-05	centroid_536	2.9197E-06
centroid_7679	putative membrane protein	47	0	54	24	centroid_767	15.9695194	6.43706E-05	centroid_767	2.9197E-06
centroid_7680	major Facilitator Superfamily protein	47	0	54	24	centroid_768	15.9695194	6.43706E-05	centroid_768	2.9197E-06
centroid_16870	hypothetical protein	46	23	55	1	centroid_1687	17.849726	2.39054E-05	centroid_1687	2.3273E-06
centroid_9351	type IV leader peptidase family protein	41	22	60	2	centroid_935	18.242755	1.94464E-05	centroid_935	5.4848E-06
centroid_2008	conserved hypothetical protein	41	21	60	3	centroid_200	15.242563	9.45483E-05	centroid_200	2.8199E-05
centroid_3499	putative lipoprotein	41	21	60	3	centroid_349	15.242563	9.45483E-05	centroid_349	2.8199E-05
centroid_4382	putative membrane protein	40	21	61	3	centroid_438	15.9392437	6.54084E-05	centroid_438	2.2075E-05
centroid_4383	conserved hypothetical protein	40	21	61	3	centroid_438	15.9392437	6.54084E-05	centroid_438	2.2075E-05
centroid_4384	conserved hypothetical protein	40	21	61	3	centroid_438	15.9392437	6.54084E-05	centroid_438	2.2075E-05
centroid_5551	conserved hypothetical protein	40	21	61	3	centroid_555	15.9392437	6.54084E-05	centroid_555	2.2075E-05
centroid_4559	conserved hypothetical protein	39	23	62	1	centroid_455	23.1605615	1.49022E-06	centroid_455	1.2467E-07
centroid_18583	type VII secretion system (T7SS), usher family protein	37	22	64	2	centroid_1858	21.4098773	3.70856E-06	centroid_1858	6.4695E-07
centroid_4629	conserved hypothetical protein	37	21	64	3	centroid_462	18.1809862	2.00874E-05	centroid_462	6.3655E-06
centroid_9633	conserved hypothetical protein	37	21	64	3	centroid_963	18.1809862	2.00874E-05	centroid_963	6.3655E-06
centroid_11126	conserved hypothetical protein	36	23	65	1	centroid_1112	25.8263677	3.7355E-07	centroid_1112	4.9635E-08
centroid_10979	-negative pili assembly chaperone, N-terminal domain pr	36	22	65	2	centroid_1097	22.2714984	2.36694E-06	centroid_1097	4.3505E-07
centroid_12391	lamB porin family protein	36	22	65	2	centroid_1239	22.2714984	2.36694E-06	centroid_1239	4.3505E-07
centroid_14262	phosphotransferase system, EIIC family protein	36	22	65	2	centroid_1426	22.2714984	2.36694E-06	centroid_1426	4.3505E-07
centroid_17336	naltoporin periplasmic N-terminal extension family protei	36	22	65	2	centroid_1733	22.2714984	2.36694E-06	centroid_1733	4.3505E-07
centroid_17337	lamB porin family protein	36	22	65	2	centroid_1733	22.2714984	2.36694E-06	centroid_1733	4.3505E-07
centroid_4617	PTS system, Lactose/Cellobiose specific IIA subunit	36	22	65	2	centroid_461	22.2714984	2.36694E-06	centroid_461	4.3505E-07
centroid_4618	naltoporin periplasmic N-terminal extension family protei	36	22	65	2	centroid_461	22.2714984	2.36694E-06	centroid_461	4.3505E-07
centroid_4619	lamB porin family protein	36	22	65	2	centroid_461	22.2714984	2.36694E-06	centroid_461	4.3505E-07
centroid_4708	-negative pili assembly chaperone, C-terminal domain pr	36	22	65	2	centroid_470	22.2714984	2.36694E-06	centroid_470	4.3505E-07
centroid_5810	cryptic outer membrane porin BglH	36	22	65	2	centroid_581	22.2714984	2.36694E-06	centroid_581	4.3505E-07
centroid_8525	naltoporin periplasmic N-terminal extension family protei	36	22	65	2	centroid_852	22.2714984	2.36694E-06	centroid_852	4.3505E-07
centroid_8526	lamB porin family protein	36	22	65	2	centroid_852	22.2714984	2.36694E-06	centroid_852	4.3505E-07
centroid_10540	glycosyl hydrolase 1 family protein	36	21	65	3	centroid_1054	18.9830476	1.31885E-05	centroid_1054	5.0447E-06
centroid_13126	bacterial regulatory, gntR family protein	36	21	65	3	centroid_1312	18.9830476	1.31885E-05	centroid_1312	5.0447E-06
centroid_13205	UTRA domain protein	36	21	65	3	centroid_1320	18.9830476	1.31885E-05	centroid_1320	5.0447E-06
centroid_18361	PTS system, Lactose/Cellobiose specific IIB subunit	36	21	65	3	centroid_1836	18.9830476	1.31885E-05	centroid_1836	5.0447E-06
centroid_4615	PTS system, Lactose/Cellobiose specific IIB subunit	36	21	65	3	centroid_461	18.9830476	1.31885E-05	centroid_461	5.0447E-06
centroid_4616	TS system, lactose/cellobiose IIC component family prote	36	21	65	3	centroid_461	18.9830476	1.31885E-05	centroid_461	5.0447E-06
centroid_5809	PTS system, Lactose/Cellobiose specific IIB subunit	36	21	65	3	centroid_580	18.9830476	1.31885E-05	centroid_580	5.0447E-06
centroid_7362	glycosyl hydrolase 1 family protein	36	21	65	3	centroid_736	18.9830476	1.31885E-05	centroid_736	5.0447E-06
centroid_18473	conserved hypothetical protein	36	20	65	4	centroid_1847	15.9579777	6.47643E-05	centroid_1847	2.5617E-05
centroid_4614	deoR-like helix-turn-helix domain protein	36	20	65	4	centroid_461	15.9579777	6.47643E-05	centroid_461	2.5617E-05
centroid_16140	conserved hypothetical protein	35	20	66	4	centroid_1614	16.7267564	4.31679E-05	centroid_1614	1.9088E-05
centroid_6099	conserved hypothetical protein	35	20	66	4	centroid_609	16.7267564	4.31679E-05	centroid_609	1.9088E-05
centroid_9309	yqeH domain protein	34	23	67	1	centroid_930	27.7605919	1.37294E-07	centroid_930	1.145E-08
centroid_4714	insA C-terminal domain protein	33	20	68	4	centroid_471	18.3566568	1.83178E-05	centroid_471	1.3752E-05
centroid_4715	conserved hypothetical protein	33	20	68	4	centroid_471	18.3566568	1.83178E-05	centroid_471	1.3752E-05
centroid_4716	putative dsORF-f3	33	20	68	4	centroid_471	18.3566568	1.83178E-05	centroid_471	1.3752E-05
centroid_5901	putative transposase	33	20	68	4	centroid_590	18.3566568	1.83178E-05	centroid_590	1.3752E-05
centroid_5902	putative dsORF-f3	33	20	68	4	centroid_590	18.3566568	1.83178E-05	centroid_590	1.3752E-05
centroid_7724	putative dsORF-f3	33	20	68	4	centroid_772	18.3566568	1.83178E-05	centroid_772	1.3752E-05
centroid_17544	conserved hypothetical protein	33	19	68	5	centroid_1754	15.3936825	8.72796E-05	centroid_1754	4.9834E-05
centroid_4305	plasmid stabilisation system family protein	32	22	69	2	centroid_430	26.0430419	3.3389E-07	centroid_430	6.2689E-08
centroid_4306	ribbon-helix-helix , copG family protein	32	22	69	2	centroid_430	26.0430419	3.3389E-07	centroid_430	6.2689E-08
centroid_10778	BCCT transporter family protein	31	24	70	0	centroid_1077	35.0433202	3.22451E-09	centroid_1077	7.7305E-11
centroid_13577	fimbrial family protein	31	19	70	5	centroid_1357	17.0194754	3.69984E-05	centroid_1357	1.9031E-05
centroid_13894	type VII secretion system (T7SS), usher family protein	31	19	70	5	centroid_1389	17.0194754	3.69984E-05	centroid_1389	1.9031E-05
centroid_13940	type VII secretion system (T7SS), usher family protein	31	19	70	5	centroid_1394	17.0194754	3.69984E-05	centroid_1394	1.9031E-05
centroid_16151	fimbrial family protein	31	19	70	5	centroid_1615	17.0194754	3.69984E-05	centroid_1615	1.9031E-05
centroid_17669	-negative pili assembly chaperone, N-terminal domain pr	31	19	70	5	centroid_1766	17.0194754	3.69984E-05	centroid_1766	1.9031E-05
centroid_5841	o-4-hydroxy-6-hydroxymethyldihydropteridine diphosphc	31	19	70	5	centroid_584	17.0194754	3.69984E-05	centroid_584	1.9031E-05
centroid_5842	fimbrial family protein	31	19	70	5	centroid_584	17.0194754	3.69984E-05	centroid_584	1.9031E-05
centroid_5843	-negative pili assembly chaperone, N-terminal domain pr	31	19	70	5	centroid_584	17.0194754	3.69984E-05	centroid_584	1.9031E-05
centroid_8553	conserved hypothetical protein	31	19	70	5	centroid_855	17.0194754	3.69984E-05	centroid_855	1.9031E-05
centroid_13286	conserved hypothetical protein	30	20	71	4	centroid_1328	21.0589418	4.4537E-06	centroid_1328	2.7294E-06
centroid_7092	hypothetical protein	30	20	71	4	centroid_709	21.0589418	4.4537E-06	centroid_709	2.7294E-06
centroid_14250	-negative pili assembly chaperone, N-terminal domain pr	30	19	71	5	centroid_1425	17.8857273	2.34574E-05	centroid_1425	1.3206E-05
centroid_14251	-negative pili assembly chaperone, C-terminal domain pr	30	19	71	5	centroid_1425	17.8857273	2.34574E-05	centroid_1425	1.3206E-05
centroid_18344	outer membrane usher protein HtrE	30	19	71	5	centroid_1834	17.8857273	2.34574E-05	centroid_1834	1.3206E-05
centroid_18345	papC N-terminal domain protein	30	19	71	5	centroid_1834	17.8857273	2.34574E-05	centroid_1834	1.3206E-05
centroid_4585	-negative pili assembly chaperone, N-terminal domain pr	30	19	71	5	centroid_458	17.8857273	2.34574E-05	centroid_458	1.3206E-05
centroid_4586	CS1 type fimbrial major subunit	30	19	71	5	centroid_458	17.8857273	2.34574E-05	centroid_458	1.3206E-05
centroid_4587	putative outer membrane fimbrial usher domain protein	30	19	71	5	centroid_458	17.8857273	2.34574E-05	centroid_458	1.3206E-05
centroid_4627	chaperone protein FocC	30	19	71	5	centroid_462	17.8857273	2.34574E-05	centroid_462	1.3206E-05

centroid_5844	outer membrane usher protein HtrE	30	19	71	5	centroid_5844	17.8857273	2.34574E-05	centroid_5844	1.3206E-05
centroid_5845	fimbrial family protein	30	19	71	5	centroid_5845	17.8857273	2.34574E-05	centroid_5845	1.3206E-05
centroid_12971	conserved hypothetical protein	29	23	72	1	centroid_1297	33.2508176	8.10057E-09	centroid_1297	8.1343E-10
centroid_4408	conserved hypothetical protein	29	21	72	3	centroid_4408	25.5281388	4.35978E-07	centroid_4408	1.4913E-07
centroid_6098	conserved hypothetical protein	29	20	72	4	centroid_6098	22.0364806	2.67517E-06	centroid_6098	1.2168E-06
centroid_4904	methyltransferase domain protein	28	24	73	0	centroid_4904	38.7764089	4.75237E-10	centroid_4904	1.3245E-11
centroid_4387	hicB family protein	28	21	73	3	centroid_4387	26.6199646	2.47671E-07	centroid_4387	8.9816E-08
centroid_17338	type VII secretion system (T7SS), usher family protein	28	20	73	4	centroid_1733	23.0562991	1.57326E-06	centroid_1733	7.568E-07
centroid_4717	conserved hypothetical protein	28	20	73	4	centroid_4717	23.0562991	1.57326E-06	centroid_4717	7.568E-07
centroid_11133	hypothetical protein	28	19	73	5	centroid_1113	19.7358151	8.89192E-06	centroid_1113	7.4927E-06
centroid_15015	conserved hypothetical protein	28	19	73	5	centroid_1501	19.7358151	8.89192E-06	centroid_1501	7.4927E-06
centroid_4626	type-1 fimbrial protein, A chain	28	19	73	5	centroid_4626	19.7358151	8.89192E-06	centroid_4626	7.4927E-06
centroid_10927	conserved hypothetical protein	28	18	73	6	centroid_1092	16.6590528	4.47363E-05	centroid_1092	3.1056E-05
centroid_11168	conserved hypothetical protein	28	18	73	6	centroid_1116	16.6590528	4.47363E-05	centroid_1116	3.1056E-05
centroid_13619	conserved hypothetical protein	28	18	73	6	centroid_1361	16.6590528	4.47363E-05	centroid_1361	3.1056E-05
centroid_18481	conserved hypothetical protein	28	18	73	6	centroid_1848	16.6590528	4.47363E-05	centroid_1848	3.1056E-05
centroid_4607	phenolic acid decarboxylase subunit D	27	23	74	1	centroid_4607	35.755724	2.23674E-09	centroid_4607	2.5549E-10
centroid_4608	ubiD decarboxylase family protein	27	23	74	1	centroid_4608	35.755724	2.23674E-09	centroid_4608	2.5549E-10
centroid_4609	putative aromatic acid decarboxylase	27	23	74	1	centroid_4609	35.755724	2.23674E-09	centroid_4609	2.5549E-10
centroid_4610	marR family protein	27	23	74	1	centroid_4610	35.755724	2.23674E-09	centroid_4610	2.5549E-10
centroid_5672	ubiD decarboxylase family protein	27	23	74	1	centroid_5672	35.755724	2.23674E-09	centroid_5672	2.5549E-10
centroid_5673	octaprenyl-4-hydroxybenzoate carboxy-lyase family prot	27	23	74	1	centroid_5673	35.755724	2.23674E-09	centroid_5673	2.5549E-10
centroid_8769	conserved domain protein	27	23	74	1	centroid_8769	35.755724	2.23674E-09	centroid_8769	2.5549E-10
centroid_4390	conserved hypothetical protein	27	21	74	3	centroid_4390	27.7582202	1.37463E-07	centroid_4390	5.3269E-08
centroid_5759	conserved hypothetical protein	27	21	74	3	centroid_5759	27.7582202	1.37463E-07	centroid_5759	5.3269E-08
centroid_17931	conserved hypothetical protein	26	18	75	6	centroid_1793	18.5245991	1.67726E-05	centroid_1793	1.8759E-05
centroid_18482	conserved hypothetical protein	26	18	75	6	centroid_1848	18.5245991	1.67726E-05	centroid_1848	1.8759E-05
centroid_15014	conserved hypothetical protein	26	17	75	7	centroid_1501	15.5307018	8.11761E-05	centroid_1501	6.7215E-05
centroid_5564	conserved hypothetical protein	26	17	75	7	centroid_5564	15.5307018	8.11761E-05	centroid_5564	6.7215E-05
centroid_14951	conserved hypothetical protein	25	19	76	5	centroid_1495	22.8436074	1.75733E-06	centroid_1495	1.2179E-06
centroid_9569	glycosyl hydrolases 15 family protein	24	18	77	6	centroid_9569	20.5800253	5.71897E-06	centroid_9569	4.581E-06
centroid_5861	glycosyl hydrolases 15 family protein	24	17	77	7	centroid_5861	17.4162461	3.00249E-05	centroid_5861	4.4073E-05
centroid_9789	conserved hypothetical protein	24	17	77	7	centroid_9789	17.4162461	3.00249E-05	centroid_9789	4.4073E-05
centroid_11584	biquinone/plastoquinone (complex I), various chains fami	23	24	78	0	centroid_1158	46.0576857	1.14822E-11	centroid_1158	5.0087E-13
centroid_5580	H-Ubiquinone oxidoreductase (complex I), chain family pr	23	24	78	0	centroid_5580	46.0576857	1.14822E-11	centroid_5580	5.0087E-13
centroid_7285	biquinone/plastoquinone (complex I), various chains fami	23	24	78	0	centroid_7285	46.0576857	1.14822E-11	centroid_7285	5.0087E-13
centroid_13521	conjugative transfer relaxase protein TraI	23	18	78	6	centroid_1352	21.6873478	3.20901E-06	centroid_1352	2.8036E-06
centroid_15858	nucleotide sugar dehydrogenase family protein	23	18	78	6	centroid_1585	21.6873478	3.20901E-06	centroid_1585	2.8036E-06
centroid_12918	conserved hypothetical protein	22	19	79	5	centroid_1291	26.4263615	2.73778E-07	centroid_1291	2.5159E-07
centroid_15012	conserved hypothetical protein	22	19	79	5	centroid_1501	26.4263615	2.73778E-07	centroid_1501	2.5159E-07
centroid_5630	conserved hypothetical protein	22	19	79	5	centroid_5630	26.4263615	2.73778E-07	centroid_5630	2.5159E-07
centroid_6604	conserved hypothetical protein	22	19	79	5	centroid_6604	26.4263615	2.73778E-07	centroid_6604	2.5159E-07
centroid_6605	conserved hypothetical protein	22	19	79	5	centroid_6605	26.4263615	2.73778E-07	centroid_6605	2.5159E-07
centroid_6606	adhesin domain protein	22	19	79	5	centroid_6606	26.4263615	2.73778E-07	centroid_6606	2.5159E-07
centroid_16717	hypothetical protein	22	18	79	6	centroid_1671	22.8529086	1.74884E-06	centroid_1671	1.6832E-06
centroid_10025	conserved hypothetical protein	21	19	80	5	centroid_1002	27.7442525	1.38459E-07	centroid_1002	1.4285E-07
centroid_11324	conserved hypothetical protein	21	19	80	5	centroid_1132	27.7442525	1.38459E-07	centroid_1132	1.4285E-07
centroid_16718	conserved hypothetical protein	21	19	80	5	centroid_1671	27.7442525	1.38459E-07	centroid_1671	1.4285E-07
centroid_5714	conserved hypothetical protein	21	19	80	5	centroid_5714	27.7442525	1.38459E-07	centroid_5714	1.4285E-07
centroid_8551	conserved hypothetical protein	21	19	80	5	centroid_8551	27.7442525	1.38459E-07	centroid_8551	1.4285E-07
centroid_6980	conserved hypothetical protein	20	19	81	5	centroid_6980	29.1317632	6.76192E-08	centroid_6980	7.9315E-08
centroid_11757	rhs core with extension domain protein	20	17	81	7	centroid_1175	21.8473639	2.95222E-06	centroid_1175	3.4568E-06
centroid_10954	intimin C-type lectin domain protein	20	15	81	9	centroid_1095	15.4826774	8.32648E-05	centroid_1095	8.2179E-05
centroid_5633	intimin C-type lectin domain protein	20	15	81	9	centroid_5633	15.4826774	8.32648E-05	centroid_5633	8.2179E-05
centroid_8549	intimin C-type lectin domain protein	20	15	81	9	centroid_8549	15.4826774	8.32648E-05	centroid_8549	8.2179E-05
centroid_16839	bacterial Ig-like domain family protein	19	15	82	9	centroid_1683	16.5505219	4.73709E-05	centroid_1683	5.1641E-05
centroid_12447	bacterial Ig-like domain family protein	18	15	83	9	centroid_1244	17.6889335	2.60136E-05	centroid_1244	3.1705E-05
centroid_17335	bacterial Ig-like domain family protein	18	15	83	9	centroid_1733	17.6889335	2.60136E-05	centroid_1733	3.1705E-05
centroid_5632	bacterial Ig-like domain family protein	18	15	83	9	centroid_5632	17.6889335	2.60136E-05	centroid_5632	3.1705E-05
centroid_13112	bacterial Ig-like domain family protein	17	15	84	9	centroid_1311	18.904333	1.3744E-05	centroid_1311	1.8983E-05
centroid_6608	bacterial Ig-like domain family protein	17	15	84	9	centroid_6608	18.904333	1.3744E-05	centroid_6608	1.8983E-05
centroid_14352	putative predicted protein	16	17	85	7	centroid_1435	27.417318	1.63956E-07	centroid_1435	3.4181E-07
centroid_13448	hypothetical protein	16	16	85	8	centroid_1344	23.6998132	1.12593E-06	centroid_1344	2.0798E-06
centroid_5805	phage minor tail family protein	16	16	85	8	centroid_5805	23.6998132	1.12593E-06	centroid_5805	2.0798E-06
centroid_5803	phage tail assembly protein T	16	15	85	9	centroid_5803	20.2039788	6.9608E-06	centroid_5803	1.1059E-05
centroid_7253	phage minor tail protein G	16	15	85	9	centroid_7253	20.2039788	6.9608E-06	centroid_7253	1.1059E-05
centroid_13083	conserved hypothetical protein	15	14	86	10	centroid_1308	18.2093148	1.97909E-05	centroid_1308	3.06E-05
centroid_15812	hypothetical protein	14	18	87	6	centroid_1581	34.9152587	3.44371E-09	centroid_1581	1.1745E-08
centroid_11244	conserved hypothetical family protein	14	15	87	9	centroid_1124	23.0900898	1.54585E-06	centroid_1124	3.4235E-06
centroid_8844	ive escherichia coli IMT2125 genomic chromosome, IMT	13	22	88	2	centroid_8844	55.8773418	7.71365E-14	centroid_8844	1.8602E-13
centroid_5804	phage tail tape measure protein, lambda family	13	15	88	9	centroid_5804	24.6966593	6.71004E-07	centroid_5804	1.8078E-06
centroid_5679	protein HokA	12	22	89	2	centroid_5679	58.376325	2.1648E-14	centroid_5679	7.0567E-14
centroid_7086	transposase family protein	12	19	89	5	centroid_7086	43.5368392	4.16053E-11	centroid_7086	2.4884E-10
centroid_7274	type VII secretion system (T7SS), usher family protein	12	19	89	5	centroid_7274	43.5368392	4.16053E-11	centroid_7274	2.4884E-10
centroid_15857	aldehyde dehydrogenase family protein	12	17	89	7	centroid_1585	34.5881584	4.07377E-09	centroid_1585	2.025E-08
centroid_6419	homoprotocatechuate degradation operon regulator, HpaR	12	17	89	7	centroid_6419	34.5881584	4.07377E-09	centroid_6419	2.025E-08
centroid_12523	ydroxyphenylacetate 3-hydroxylase C terminal family pro	12	16	89	8	centroid_1252	30.406885	3.50283E-08	centroid_1252	1.4556E-07
centroid_12524	ydroxyphenylacetate 3-hydroxylase N terminal family pro	12	16	89	8	centroid_1252	30.406885	3.50283E-08	centroid_1252	1.4556E-07
centroid_6420	rotocatechuate catabolism bifunctional isomerase/decarb	12	16	89	8	centroid_6420	30.406885	3.50283E-08	centroid_6420	1.4556E-07
centroid_6421	boxymethyl-2-hydroxymuconate semialdehyde dehydrog	12	16	89	8	centroid_6421	30.406885	3.50283E-08	centroid_6421	1.4556E-07
centroid_6422	3,4-dihydroxyphenylacetate 2,3-dioxygenase	12	16	89	8	centroid_6422	30.406885	3.50283E-08	centroid_6422	1.4556E-07
centroid_6424	2-oxo-hepta-3-ene-1,7-dioic acid hydratase	12	16	89	8	centroid_6424	30.406885	3.50283E-08	centroid_6424	1.4556E-07
centroid_6425	2,4-dihydroxyhept-2-ene-1,7-dioic acid aldolase	12	16	89	8	centroid_6425	30.406885	3.50283E-08	centroid_6425	1.4556E-07
centroid_6426	4-hydroxyphenylacetate permease	12	16	89	8	centroid_6426	30.406885	3.50283E-08	centroid_6426	1.4556E-07
centroid_6428	lroxyphenylacetate 3-monooxygenase, oxygenase comp	12	16	89	8	centroid_6428	30.406885	3.50283E-08	centroid_6428	1.4556E-07
centroid_6429	droxyphenylacetate 3-monooxygenase, reductase comp	12	16	89	8	centroid_6429	30.406885	3.50283E-08	centroid_6429	1.4556E-07
centroid_6427	-hydroxyphenylacetate catabolism regulatory protein Hpa	12	14	89	10	centroid_6427	22.6591619	1.93436E-06	centroid_6427	5.1191E-06
centroid_10754	phage integrase family protein	12	12	89	12	centroid_1075	15.7890262	7.0812E-05	centroid_1075	0.00011372
centroid_10977	conserved hypothetical protein	12	12	89	12	centroid_1097	15.7890262	7.0812E-05	centroid_1097	0.00011372
centroid_13431	conserved hypothetical protein	12	12	89	12	centroid_1343	15.7890262	7.0812E-05	centroid_1343	0.00011372
centroid_13450	conserved hypothetical protein	12	12	89	12	centroid_1345	15.7890262	7.0812E-05	centroid_1345	0.00011372
centroid_15117	conserved hypothetical protein	12	12	89	12	centroid_1511	15.7890262	7.0812E-05	centroid_1511	0.00011372
centroid_7239	putative excisionase	12	12	89	12	centroid_7239	15.7890262	7.0812E-05	centroid_7239	0.00011372
centroid_7761	putative iS621 protein	11	18	90	6	centroid_7761	41.205452	1.3704E-10	centroid_7761	1.0354E-09
centroid_12632	mu-like prophage major head subunit gpT family protein	11	15	90	9	centroid_1263	28.2987483	1.03963E-07	centroid_1263	4.452E-07
centroid_15492	putative predicted protein	11	15	90	9	centroid_1549	28.2987483	1.03963E-07	centroid_1549	4.452E-07
centroid_10961	trbC domain protein	11	13	90	11	centroid_1096	20.7032707	5.36243E-06	centroid_1096	1.3584E-05
centroid_5798	conserved hypothetical protein	11	13	90	11	centroid_5798	20.7032707	5.36243E-06	centroid_5798	1.3584E-05
centroid_5799	TP-binding sugar transporter from pro-phage family prote	11	13	90	11	centroid_5799	20.7032707	5.36243E-06	centroid_5799	1.3584E-05
centroid_5796	phage portal protein, lambda family	11	12	90	12	centroid_5796	17.2356045	3.3019E-05	centroid_5796	6.3651E-05
centroid_7233	killing protein KilR	11	12	90	12	centroid_7233	17.2356045	3.3019E-05	centroid_7233	6.3651E-05
centroid_13516	putative tail length tape measure domain protein	10	17	91	7	centroid_1351	38.9936116	4.25195E-10	centroid_1351	3.7835E-09
centroid_7317	conserved hypothetical protein	10	17	91	7	centroid_7317	38.9936116	4.25195E-10	centroid_7317	3.7835E-09
centroid_11987	conserved hypothetical protein	10	13	91	11	centroid_1198	22.4451248	2.16234E-06	centroid_1198	6.9087E-06
centroid_7238	double-strand break reduction protein	10	12	91	12	centroid_7238	18.8244761	1.43316E-05	centroid_7238	3.4046E-05
centroid_7240	phage integrase family protein	10	12	91	12	centroid_7240	18.8244761	1.43316E-05	centroid_7240	3.4046E-05
centroid_18615	putative transposase	9	16	92	8	centroid_1861	36.8998814	1.24354E-09	centroid_1861	1.2366E-08

centroid_6792	phage tail tape measure protein, lambda family	9	15	92	9	centroid_6792	32.5261772	1.17598E-08	centroid_6792	8.9627E-08
centroid_10033	conserved hypothetical protein	9	12	92	12	centroid_1003	20.5756426	5.73208E-06	centroid_1003	1.7292E-05
centroid_13111	conserved hypothetical protein	9	12	92	12	centroid_1311	20.5756426	5.73208E-06	centroid_1311	1.7292E-05
centroid_13445	integrase core domain protein	9	12	92	12	centroid_1344	20.5756426	5.73208E-06	centroid_1344	1.7292E-05
centroid_6100	integrase core domain protein	9	12	92	12	centroid_6100	20.5756426	5.73208E-06	centroid_6100	1.7292E-05
centroid_9763	integrase core domain protein	9	12	92	12	centroid_9763	20.5756426	5.73208E-06	centroid_9763	1.7292E-05
centroid_5828	putative cytoplasmic protein	8	19	93	5	centroid_5828	53.9952003	2.0098E-13	centroid_5828	4.7845E-12
centroid_5911	helix-turn-helix domain protein	8	18	93	6	centroid_5911	48.9738928	2.59392E-12	centroid_5911	5.5868E-11
centroid_8815	putative permease family protein	8	18	93	6	centroid_8815	48.9738928	2.59392E-12	centroid_8815	5.5868E-11
centroid_13157	bacterial Ig-like domain family protein	8	13	93	11	centroid_1315	26.4549222	2.6976E-07	centroid_1315	1.509E-06
centroid_13292	conserved hypothetical protein	8	13	93	11	centroid_1329	26.4549222	2.6976E-07	centroid_1329	1.509E-06
centroid_5797	clp protease family protein	8	12	93	12	centroid_5797	22.5131191	2.08713E-06	centroid_5797	8.2732E-06
centroid_7235	putative protein RacC	8	12	93	12	centroid_7235	22.5131191	2.08713E-06	centroid_7235	8.2732E-06
centroid_7396	outer membrane porin protein OmpD	8	11	93	13	centroid_7396	18.7833333	1.46441E-05	centroid_7396	4.1053E-05
centroid_14508	RHS repeat-associated core domain protein	8	10	93	14	centroid_1450	15.2823468	9.25777E-05	centroid_1450	0.00018474
centroid_17334	bacterial Ig-like domain family protein	8	10	93	14	centroid_1733	15.2823468	9.25777E-05	centroid_1733	0.00018474
centroid_18330	RHS repeat-associated core domain protein	8	10	93	14	centroid_1833	15.2823468	9.25777E-05	centroid_1833	0.00018474
centroid_6025	outer membrane lipoprotein blc	8	10	93	14	centroid_6025	15.2823468	9.25777E-05	centroid_6025	0.00018474
centroid_6111	transposase family protein	8	10	93	14	centroid_6111	15.2823468	9.25777E-05	centroid_6111	0.00018474
centroid_6607	bacterial Ig-like domain family protein	8	10	93	14	centroid_6607	15.2823468	9.25777E-05	centroid_6607	0.00018474
centroid_14737	conserved hypothetical protein	7	22	94	2	centroid_1473	73.4630217	1.02539E-17	centroid_1473	2.2251E-16
centroid_7211	ankyrin repeat family protein	7	19	94	5	centroid_7211	57.117736	4.10487E-14	centroid_7211	1.4887E-12
centroid_5800	prophage minor tail Z family protein	7	13	94	11	centroid_5800	28.774906	8.12977E-08	centroid_5800	6.3618E-07
centroid_9788	conserved hypothetical protein	7	13	94	11	centroid_9788	28.774906	8.12977E-08	centroid_9788	6.3618E-07
centroid_7687	conserved hypothetical protein	7	11	94	13	centroid_7687	20.7577289	5.21208E-06	centroid_7687	1.9423E-05
centroid_16938	bacterial Ig-like domain family protein	7	10	94	14	centroid_1693	17.0662051	3.6099E-05	centroid_1693	9.2899E-05
centroid_7523	plasmid segregation protein ParM	7	10	94	14	centroid_7523	17.0662051	3.6099E-05	centroid_7523	9.2899E-05
centroid_7524	plasmid stability family protein	7	10	94	14	centroid_7524	17.0662051	3.6099E-05	centroid_7524	9.2899E-05
centroid_9491	:orbate-specific phosphotransferase enzyme IIA compon	7	10	94	14	centroid_9491	17.0662051	3.6099E-05	centroid_9491	9.2899E-05
centroid_13094	CRISPR-associated endonuclease Cas3-HD	6	20	95	4	centroid_1309	65.8876435	4.77375E-16	centroid_1309	2.7245E-14
centroid_14390	CRISPR-associated endonuclease Cas3-HD	6	20	95	4	centroid_1439	65.8876435	4.77375E-16	centroid_1439	2.7245E-14
centroid_5674	SPR-associated endoribonuclease Cas2, subtype I-E/EC	6	20	95	4	centroid_5674	65.8876435	4.77375E-16	centroid_5674	2.7245E-14
centroid_13093	CRISPR-associated helicase Cas3	6	19	95	5	centroid_1309	60.4920844	7.3875E-15	centroid_1309	4.2072E-13
centroid_15847	conserved hypothetical protein	6	19	95	5	centroid_1584	60.4920844	7.3875E-15	centroid_1584	4.2072E-13
centroid_5675	SPR-associated protein Cas6/Cse3/CasE, subtype I-E/E(	6	19	95	5	centroid_5675	60.4920844	7.3875E-15	centroid_5675	4.2072E-13
centroid_5676	RISPR-associated protein Cas5/CasD, subtype I-E/ECO	6	19	95	5	centroid_5676	60.4920844	7.3875E-15	centroid_5676	4.2072E-13
centroid_5678	CRISPR-associated helicase Cas3	6	19	95	5	centroid_5678	60.4920844	7.3875E-15	centroid_5678	4.2072E-13
centroid_6108	transposase IS116/IS110/IS902 family protein	6	19	95	5	centroid_6108	60.4920844	7.3875E-15	centroid_6108	4.2072E-13
centroid_7349	SPR-associated protein Cas7/Cse4/CasC, subtype I-E/E(	6	19	95	5	centroid_7349	60.4920844	7.3875E-15	centroid_7349	4.2072E-13
centroid_7675	CRISPR-associated endonuclease Cas1	6	19	95	5	centroid_7675	60.4920844	7.3875E-15	centroid_7675	4.2072E-13
centroid_8842	SPR-associated protein Cas7/Cse4/CasC, subtype I-E/E(	6	19	95	5	centroid_8842	60.4920844	7.3875E-15	centroid_8842	4.2072E-13
centroid_12532	conserved hypothetical family protein	6	17	95	7	centroid_1253	50.1522993	1.42264E-12	centroid_1253	5.9339E-11
centroid_18577	conserved hypothetical protein	6	17	95	7	centroid_1857	50.1522993	1.42264E-12	centroid_1857	5.9339E-11
centroid_8547	conserved hypothetical protein	6	17	95	7	centroid_8547	50.1522993	1.42264E-12	centroid_8547	5.9339E-11
centroid_14626	conserved hypothetical protein	6	15	95	9	centroid_1462	40.4270654	2.04094E-10	centroid_1462	4.7981E-09
centroid_5677	CRISPR type I-E/ECOLI-associated protein CasA/Cse1	6	14	95	10	centroid_5677	35.8040687	2.18192E-09	centroid_5677	3.6225E-08
centroid_7350	CRISPR type I-E/ECOLI-associated protein CasB/Cse2	6	14	95	10	centroid_7350	35.8040687	2.18192E-09	centroid_7350	3.6225E-08
centroid_8843	CT1975-like family protein	6	14	95	10	centroid_8843	35.8040687	2.18192E-09	centroid_8843	3.6225E-08
centroid_9176	putative yfdA protein	6	13	95	11	centroid_9176	31.3487889	2.15593E-08	centroid_9176	2.4621E-07
centroid_12380	integrase core domain protein	6	11	95	13	centroid_1238	22.9785099	1.63823E-06	centroid_1238	8.4793E-06
centroid_7871	integrase core domain protein	6	10	95	14	centroid_7871	19.0898572	1.24706E-05	centroid_7871	4.324E-05
centroid_10820	zinc-binding dehydrogenase family protein	6	9	95	15	centroid_1082	15.4236127	8.59081E-05	centroid_1082	0.00020111
centroid_12694	bacteriophage lambda tail assembly I family protein	6	9	95	15	centroid_1269	15.4236127	8.59081E-05	centroid_1269	0.00020111
centroid_13104	conserved hypothetical protein	6	9	95	15	centroid_1310	15.4236127	8.59081E-05	centroid_1310	0.00020111
centroid_14715	conserved hypothetical protein	6	9	95	15	centroid_1471	15.4236127	8.59081E-05	centroid_1471	0.00020111
centroid_7234	prokaryotic metallothionein family protein	6	9	95	15	centroid_7234	15.4236127	8.59081E-05	centroid_7234	0.00020111
centroid_8474	putative sor-operon regulator	6	9	95	15	centroid_8474	15.4236127	8.59081E-05	centroid_8474	0.00020111
centroid_8475	short chain dehydrogenase family protein	6	9	95	15	centroid_8475	15.4236127	8.59081E-05	centroid_8475	0.00020111
centroid_8476	m, mannose/fructose/sorbose family, IIA component dom	6	9	95	15	centroid_8476	15.4236127	8.59081E-05	centroid_8476	0.00020111
centroid_8477	rbose-specific phosphotransferase enzyme IIB compone	6	9	95	15	centroid_8477	15.4236127	8.59081E-05	centroid_8477	0.00020111
centroid_8478	stem, mannose/fructose/sorbose , IIC component family	6	9	95	15	centroid_8478	15.4236127	8.59081E-05	centroid_8478	0.00020111
centroid_8479	stem, mannose/fructose/sorbose , IID component family	6	9	95	15	centroid_8479	15.4236127	8.59081E-05	centroid_8479	0.00020111
centroid_8480	zinc-binding dehydrogenase family protein	6	9	95	15	centroid_8480	15.4236127	8.59081E-05	centroid_8480	0.00020111
centroid_9043	putative sugar-binding domain protein	6	9	95	15	centroid_9043	15.4236127	8.59081E-05	centroid_9043	0.00020111
centroid_9044	putative sugar-binding domain protein	6	9	95	15	centroid_9044	15.4236127	8.59081E-05	centroid_9044	0.00020111
centroid_10235	fibronectin type III family protein	5	11	96	13	centroid_1023	25.4914614	4.44345E-07	centroid_1023	3.3566E-06
centroid_13480	fibronectin type III family protein	5	11	96	13	centroid_1348	25.4914614	4.44345E-07	centroid_1348	3.3566E-06
centroid_11298	phage late control gene D family protein	5	10	96	14	centroid_1129	21.4007729	3.72621E-06	centroid_1129	1.8313E-05
centroid_15188	conserved hypothetical protein	5	9	96	15	centroid_1518	17.514495	2.85126E-05	centroid_1518	9.1433E-05
centroid_5807	bacteriophage lambda tail assembly I family protein	5	9	96	15	centroid_5807	17.514495	2.85126E-05	centroid_5807	9.1433E-05
centroid_5994	conserved hypothetical protein	5	9	96	15	centroid_5994	17.514495	2.85126E-05	centroid_5994	9.1433E-05
centroid_6796	conserved hypothetical protein	5	9	96	15	centroid_6796	17.514495	2.85126E-05	centroid_6796	9.1433E-05
centroid_5814	putative membrane protein	4	17	97	7	centroid_5814	57.3506543	3.64642E-14	centroid_5814	4.0128E-12
centroid_12688	phage tail tape measure protein, lambda family	4	13	97	11	centroid_1268	37.4362721	9.44492E-10	centroid_1268	2.634E-08
centroid_10919	type IV/VI secretion system , DotU family domain protein	4	10	97	14	centroid_1091	24.0600036	9.33799E-07	centroid_1091	6.8879E-06
centroid_12120	conserved hypothetical protein	4	10	97	14	centroid_1212	24.0600036	9.33799E-07	centroid_1212	6.8879E-06
centroid_12121	conserved hypothetical protein	4	10	97	14	centroid_1212	24.0600036	9.33799E-07	centroid_1212	6.8879E-06
centroid_14008	phage major tail tube protein	4	10	97	14	centroid_1400	24.0600036	9.33799E-07	centroid_1400	6.8879E-06
centroid_14009	phage late control gene D family protein	4	10	97	14	centroid_1400	24.0600036	9.33799E-07	centroid_1400	6.8879E-06
centroid_16551	conserved hypothetical protein	4	10	97	14	centroid_1655	24.0600036	9.33799E-07	centroid_1655	6.8879E-06
centroid_16552	imcF-related N-terminal domain protein	4	10	97	14	centroid_1655	24.0600036	9.33799E-07	centroid_1655	6.8879E-06
centroid_7369	mu-like prophage FluMu gp41 family protein	4	10	97	14	centroid_7369	24.0600036	9.33799E-07	centroid_7369	6.8879E-06
centroid_7370	phage major tail tube protein	4	10	97	14	centroid_7370	24.0600036	9.33799E-07	centroid_7370	6.8879E-06
centroid_7371	phage tail sheath family protein	4	10	97	14	centroid_7371	24.0600036	9.33799E-07	centroid_7371	6.8879E-06
centroid_7411	conserved hypothetical protein	4	10	97	14	centroid_7411	24.0600036	9.33799E-07	centroid_7411	6.8879E-06
centroid_7412	conserved hypothetical protein	4	10	97	14	centroid_7412	24.0600036	9.33799E-07	centroid_7412	6.8879E-06
centroid_7414	gene 25-like lysozyme family protein	4	10	97	14	centroid_7414	24.0600036	9.33799E-07	centroid_7414	6.8879E-06
centroid_7415	conserved hypothetical protein	4	10	97	14	centroid_7415	24.0600036	9.33799E-07	centroid_7415	6.8879E-06
centroid_7416	conserved hypothetical protein	4	10	97	14	centroid_7416	24.0600036	9.33799E-07	centroid_7416	6.8879E-06
centroid_7417	conserved hypothetical protein	4	10	97	14	centroid_7417	24.0600036	9.33799E-07	centroid_7417	6.8879E-06
centroid_7418	PAAR motif family protein	4	10	97	14	centroid_7418	24.0600036	9.33799E-07	centroid_7418	6.8879E-06
centroid_7419	impA-related N-terminal family protein	4	10	97	14	centroid_7419	24.0600036	9.33799E-07	centroid_7419	6.8879E-06
centroid_7420	type VI secretion lipofamily protein	4	10	97	14	centroid_7420	24.0600036	9.33799E-07	centroid_7420	6.8879E-06
centroid_7422	type VI secretion ATPase, ClpV1 family	4	10	97	14	centroid_7422	24.0600036	9.33799E-07	centroid_7422	6.8879E-06
centroid_9144	type IV/VI secretion system , DotU family domain protein	4	10	97	14	centroid_9144	24.0600036	9.33799E-07	centroid_9144	6.8879E-06
centroid_5921	traG-like , N-terminal region family protein	4	9	97	15	centroid_5921	19.9488294	7.95426E-06	centroid_5921	3.7083E-05
centroid_9327	5-carboxymethyl-2-hydroxymuconate Delta-isomerase	4	9	97	15	centroid_9327	19.9488294	7.95426E-06	centroid_9327	3.7083E-05
centroid_13451	conserved hypothetical protein	4	8	97	16	centroid_1345	16.0426467	6.19317E-05	centroid_1345	0.00018341
centroid_6051	type VII secretion system (T7SS), usher family protein	4	8	97	16	centroid_6051	16.0426467	6.19317E-05	centroid_6051	0.00018341
centroid_7372	resolvase, N terminal domain protein	4	8	97	16	centroid_7372	16.0426467	6.19317E-05	centroid_7372	0.00018341
centroid_9242	resolvase, N terminal domain protein	4	8	97	16	centroid_9242	16.0426467	6.19317E-05	centroid_9242	0.00018341
centroid_14505	conserved hypothetical protein	3	23	98	1	centroid_1450	95.953751	1.17599E-22	centroid_1450	8.0059E-21
centroid_14504	hypothetical protein	3	20	98	4	centroid_1450	78.1452281	9.57366E-19	centroid_1450	2.3514E-16
centroid_18514	putative transcriptional activator Ogr/delta	3	10	98	14	centroid_1851	27.1474051	1.88518E-07	centroid_1851	2.2187E-06
centroid_18607	hypothetical protein	3	10	98	14	centroid_1860	27.1474051	1.88518E-07	centroid_1860	2.2187E-06
centroid_7366	phage late control gene D family protein	3	10	98	14	centroid_7366	27.1474051	1.88518E-07	centroid_7366	2.2187E-06
centroid_7367	phage P2 GpU family protein	3	10	98	14	centroid_7367	27.1474051	1.88518E-07	centroid_7367	2.2187E-06

centroid_7377	phage tail protein I	3	10	98	14	centroid_7377	27.1474051	1.88518E-07	centroid_7377 2.2187E-06
centroid_7413	conserved hypothetical protein	3	10	98	14	centroid_7413	27.1474051	1.88518E-07	centroid_7413 2.2187E-06
centroid_7421	hypothetical protein	3	10	98	14	centroid_7421	27.1474051	1.88518E-07	centroid_7421 2.2187E-06
centroid_11094	hypothetical protein	3	9	98	15	centroid_1109	22.8118524	1.7866E-06	centroid_1109 1.2946E-05
centroid_11095	putative phage immunity repressor protein	3	9	98	15	centroid_1109	22.8118524	1.7866E-06	centroid_1109 1.2946E-05
centroid_12122	hemolysin expression-modulating protein Hha	3	9	98	15	centroid_1212	22.8118524	1.7866E-06	centroid_1212 1.2946E-05
centroid_13473	hypothetical protein	3	9	98	15	centroid_1347	22.8118524	1.7866E-06	centroid_1347 1.2946E-05
centroid_6491	ogr/Delta-like zinc finger family protein	3	9	98	15	centroid_6491	22.8118524	1.7866E-06	centroid_6491 1.2946E-05
centroid_6492	putative glyco3, capsid size determination protein Sid	3	9	98	15	centroid_6492	22.8118524	1.7866E-06	centroid_6492 1.2946E-05
centroid_6495	putative derepression protein	3	9	98	15	centroid_6495	22.8118524	1.7866E-06	centroid_6495 1.2946E-05
centroid_6496	putative predicted protein	3	9	98	15	centroid_6496	22.8118524	1.7866E-06	centroid_6496 1.2946E-05
centroid_6497	conserved hypothetical protein	3	9	98	15	centroid_6497	22.8118524	1.7866E-06	centroid_6497 1.2946E-05
centroid_6498	putative P4-specific DNA primase	3	9	98	15	centroid_6498	22.8118524	1.7866E-06	centroid_6498 1.2946E-05
centroid_7429	avrPphF-ORF-2 family protein	3	9	98	15	centroid_7429	22.8118524	1.7866E-06	centroid_7429 1.2946E-05
centroid_15850	putative conjugal transfer pilus assembly protein	3	8	98	16	centroid_1585	18.6532736	1.56778E-05	centroid_1585 6.9768E-05
centroid_18640	transposase family protein	3	8	98	16	centroid_1864	18.6532736	1.56778E-05	centroid_1864 6.9768E-05
centroid_7236	exodeoxyribonuclease 8	3	8	98	16	centroid_7236	18.6532736	1.56778E-05	centroid_7236 6.9768E-05
centroid_5605	putative membrane protein	2	19	99	5	centroid_5605	77.2256886	1.52497E-18	centroid_5605 6.0291E-16
centroid_5833	haemagglutinin family protein	2	19	99	5	centroid_5833	77.2256886	1.52497E-18	centroid_5833 6.0291E-16
centroid_11144	rhs core with extension domain protein	2	15	99	9	centroid_1114	55.4049041	9.80914E-14	centroid_1114 1.6711E-11
centroid_12963	putative dsORF-e4	2	15	99	9	centroid_1296	55.4049041	9.80914E-14	centroid_1296 1.6711E-11
centroid_12964	conserved hypothetical protein	2	15	99	9	centroid_1296	55.4049041	9.80914E-14	centroid_1296 1.6711E-11
centroid_18642	conserved hypothetical protein	2	15	99	9	centroid_1864	55.4049041	9.80914E-14	centroid_1864 1.6711E-11
centroid_6089	putative rHS protein	2	15	99	9	centroid_6089	55.4049041	9.80914E-14	centroid_6089 1.6711E-11
centroid_6090	conserved hypothetical protein	2	15	99	9	centroid_6090	55.4049041	9.80914E-14	centroid_6090 1.6711E-11
centroid_15002	DKNYY family protein	2	12	99	12	centroid_1500	40.2620038	2.22087E-10	centroid_1500 1.1355E-08
centroid_5815	conserved hypothetical protein	2	12	99	12	centroid_5815	40.2620038	2.22087E-10	centroid_5815 1.1355E-08
centroid_14329	conserved hypothetical family protein	2	11	99	13	centroid_1432	35.4527729	2.6131E-09	centroid_1432 8.4124E-08
centroid_11002	putative transposase	2	9	99	15	centroid_1100	26.2198198	3.04679E-07	centroid_1100 3.669E-06
centroid_11785	putative transposase	2	9	99	15	centroid_1178	26.2198198	3.04679E-07	centroid_1178 3.669E-06
centroid_6082	putative transposase	2	9	99	15	centroid_6082	26.2198198	3.04679E-07	centroid_6082 3.669E-06
centroid_10179	CFA/I fimbrial subunit D	2	7	99	17	centroid_1017	17.5750996	2.76181E-05	centroid_1017 0.00011925
centroid_11055	H+ symporter family protein	1	23	100	1	centroid_1105	106.410035	5.99534E-25	centroid_1105 7.533E-23
centroid_11056	lacY proton/sugar symporter family protein	1	23	100	1	centroid_1105	106.410035	5.99534E-25	centroid_1105 7.533E-23
centroid_12882	H+ symporter family protein	1	23	100	1	centroid_1288	106.410035	5.99534E-25	centroid_1288 7.533E-23
centroid_13367	type IV leader peptidase family protein	1	23	100	1	centroid_1336	106.410035	5.99534E-25	centroid_1336 7.533E-23
centroid_5854	H+ symporter family protein	1	23	100	1	centroid_5854	106.410035	5.99534E-25	centroid_5854 7.533E-23
centroid_5855	pfkB carbohydrate kinase family protein	1	23	100	1	centroid_5855	106.410035	5.99534E-25	centroid_5855 7.533E-23
centroid_5856	sucrose-6-phosphate hydrolase family protein	1	23	100	1	centroid_5856	106.410035	5.99534E-25	centroid_5856 7.533E-23
centroid_5857	lasmic binding and sugar binding domain of LacI family pr	1	23	100	1	centroid_5857	106.410035	5.99534E-25	centroid_5857 7.533E-23
centroid_5955	BFD-like [2Fe-2S] binding domain protein	1	23	100	1	centroid_5955	106.410035	5.99534E-25	centroid_5955 7.533E-23
centroid_5956	bacterioferritin	1	23	100	1	centroid_5956	106.410035	5.99534E-25	centroid_5956 7.533E-23
centroid_9104	H+ symporter family protein	1	23	100	1	centroid_9104	106.410035	5.99534E-25	centroid_9104 7.533E-23
centroid_12883	LPXTG cell wall anchor domain protein	1	22	100	2	centroid_1288	100.241727	1.34888E-23	centroid_1288 3.6834E-21
centroid_13024	hypothetical protein	1	22	100	2	centroid_1302	100.241727	1.34888E-23	centroid_1302 3.6834E-21
centroid_14944	putative ybl55 protein	1	20	100	4	centroid_1494	88.2699979	5.7103E-21	centroid_1494 3.0052E-18
centroid_17372	fimbrial family protein	1	20	100	4	centroid_1737	88.2699979	5.7103E-21	centroid_1737 3.0052E-18
centroid_5577	tRNA(fMet)-specific endonuclease VapC	1	20	100	4	centroid_5577	88.2699979	5.7103E-21	centroid_5577 3.0052E-18
centroid_5578	antitoxin VapB	1	20	100	4	centroid_5578	88.2699979	5.7103E-21	centroid_5578 3.0052E-18
centroid_5904	SPFH domain / Band 7 family protein	1	20	100	4	centroid_5904	88.2699979	5.7103E-21	centroid_5904 3.0052E-18
centroid_7287	antitoxin VapB	1	20	100	4	centroid_7287	88.2699979	5.7103E-21	centroid_7287 3.0052E-18
centroid_13023	fimbrial family protein	1	19	100	5	centroid_1302	82.46052	1.07802E-19	centroid_1302 6.0139E-17
centroid_16055	1-negative pili assembly chaperone, N-terminal domain pr	1	19	100	5	centroid_1605	82.46052	1.07802E-19	centroid_1605 6.0139E-17
centroid_16056	1-negative pili assembly chaperone, C-terminal domain pr	1	19	100	5	centroid_1605	82.46052	1.07802E-19	centroid_1605 6.0139E-17
centroid_18522	type VII secretion system (T7SS), usher family protein	1	19	100	5	centroid_1852	82.46052	1.07802E-19	centroid_1852 6.0139E-17
centroid_18523	type VII secretion system (T7SS), usher family protein	1	19	100	5	centroid_1852	82.46052	1.07802E-19	centroid_1852 6.0139E-17
centroid_5575	inner membrane protein YmfA	1	19	100	5	centroid_5575	82.46052	1.07802E-19	centroid_5575 6.0139E-17
centroid_5621	cblD like pilus biogenesis initiator family protein	1	19	100	5	centroid_5621	82.46052	1.07802E-19	centroid_5621 6.0139E-17
centroid_5622	fimbrial family protein	1	19	100	5	centroid_5622	82.46052	1.07802E-19	centroid_5622 6.0139E-17
centroid_5684	fimbrial family protein	1	19	100	5	centroid_5684	82.46052	1.07802E-19	centroid_5684 6.0139E-17
centroid_5685	1-negative pili assembly chaperone, C-terminal domain pr	1	19	100	5	centroid_5685	82.46052	1.07802E-19	centroid_5685 6.0139E-17
centroid_5686	type VII secretion system (T7SS), usher family protein	1	19	100	5	centroid_5686	82.46052	1.07802E-19	centroid_5686 6.0139E-17
centroid_5687	fimbrial family protein	1	19	100	5	centroid_5687	82.46052	1.07802E-19	centroid_5687 6.0139E-17
centroid_5846	conserved hypothetical protein	1	19	100	5	centroid_5846	82.46052	1.07802E-19	centroid_5846 6.0139E-17
centroid_5905	putative nucleotidyltransferase family protein	1	19	100	5	centroid_5905	82.46052	1.07802E-19	centroid_5905 6.0139E-17
centroid_5906	zeta toxin family protein	1	19	100	5	centroid_5906	82.46052	1.07802E-19	centroid_5906 6.0139E-17
centroid_5847	fimbrial family protein	1	17	100	7	centroid_5847	71.1807764	3.25948E-17	centroid_5847 1.4636E-14
centroid_11150	putative yhaC	1	16	100	8	centroid_1115	65.7057964	5.23517E-16	centroid_1115 1.8677E-13
centroid_11165	hypothetical protein	1	16	100	8	centroid_1116	65.7057964	5.23517E-16	centroid_1116 1.8677E-13
centroid_5573	conserved hypothetical protein	1	16	100	8	centroid_5573	65.7057964	5.23517E-16	centroid_5573 1.8677E-13
centroid_5899	L-galactonate transporter	1	16	100	8	centroid_5899	65.7057964	5.23517E-16	centroid_5899 1.8677E-13
centroid_5900	racemase / muconate lactonizing enzyme, N-terminal dor	1	15	100	9	centroid_5900	60.3380689	7.98873E-15	centroid_5900 2.1308E-12
centroid_11109	1-negative pili assembly chaperone, C-terminal domain pr	1	14	100	10	centroid_1110	55.0760154	1.15957E-13	centroid_1110 2.1997E-11
centroid_5849	conserved hypothetical protein	1	14	100	10	centroid_5849	55.0760154	1.15957E-13	centroid_5849 2.1997E-11
centroid_5850	fimbrial family protein	1	14	100	10	centroid_5850	55.0760154	1.15957E-13	centroid_5850 2.1997E-11
centroid_5851	fimbrial family protein	1	14	100	10	centroid_5851	55.0760154	1.15957E-13	centroid_5851 2.1997E-11
centroid_5852	fimbrial family protein	1	14	100	10	centroid_5852	55.0760154	1.15957E-13	centroid_5852 2.1997E-11
centroid_10046	antitoxin HigA	1	13	100	11	centroid_1004	49.9184956	1.60267E-12	centroid_1004 2.0741E-10
centroid_17830	helix-turn-helix domain protein	1	13	100	11	centroid_1783	49.9184956	1.60267E-12	centroid_1783 2.0741E-10
centroid_17831	conserved hypothetical protein	1	13	100	11	centroid_1783	49.9184956	1.60267E-12	centroid_1783 2.0741E-10
centroid_18198	phage tail tape measure protein, lambda family	1	13	100	11	centroid_1819	49.9184956	1.60267E-12	centroid_1819 2.0741E-10
centroid_7196	acetyltransferase family protein	1	13	100	11	centroid_7196	49.9184956	1.60267E-12	centroid_7196 2.0741E-10
centroid_7197	conserved hypothetical protein	1	13	100	11	centroid_7197	49.9184956	1.60267E-12	centroid_7197 2.0741E-10
centroid_7244	acyl transferase domain protein	1	13	100	11	centroid_7244	49.9184956	1.60267E-12	centroid_7244 2.0741E-10
centroid_7347	antitoxin HigA	1	13	100	11	centroid_7347	49.9184956	1.60267E-12	centroid_7347 2.0741E-10
centroid_11137	putative rhs core protein	1	11	100	13	centroid_1113	39.9155055	2.6519E-10	centroid_1113 1.4465E-08
centroid_11138	conserved hypothetical protein	1	11	100	13	centroid_1113	39.9155055	2.6519E-10	centroid_1113 1.4465E-08
centroid_13461	RHS repeat-associated core domain protein	1	11	100	13	centroid_1346	39.9155055	2.6519E-10	centroid_1346 1.4465E-08
centroid_16123	conserved hypothetical protein	1	11	100	13	centroid_1612	39.9155055	2.6519E-10	centroid_1612 1.4465E-08
centroid_7527	conserved hypothetical protein	1	11	100	13	centroid_7527	39.9155055	2.6519E-10	centroid_7527 1.4465E-08
centroid_11139	putative rhs core protein with extension	1	10	100	14	centroid_1113	35.0714451	3.17827E-09	centroid_1113 1.0817E-07
centroid_13878	gram-negative porin family protein	1	10	100	14	centroid_1387	35.0714451	3.17827E-09	centroid_1387 1.0817E-07
centroid_6106	integrase core domain protein	1	10	100	14	centroid_6106	35.0714451	3.17827E-09	centroid_6106 1.0817E-07
centroid_5887	conserved hypothetical protein	1	9	100	15	centroid_5887	30.3355149	3.63411E-08	centroid_5887 7.556E-07
centroid_5888	plasmid stability family protein	1	9	100	15	centroid_5888	30.3355149	3.63411E-08	centroid_5888 7.556E-07
centroid_11012	conserved hypothetical protein	1	8	100	16	centroid_1101	25.7128117	3.96188E-07	centroid_1101 4.9437E-06
centroid_11013	outer membrane insertion C-terminal signal domain protein	1	8	100	16	centroid_1101	25.7128117	3.96188E-07	centroid_1101 4.9437E-06
centroid_11014	esterase-like activity of phytase family protein	1	8	100	16	centroid_1101	25.7128117	3.96188E-07	centroid_1101 4.9437E-06
centroid_11015	pfkB carbohydrate kinase family protein	1	8	100	16	centroid_1101	25.7128117	3.96188E-07	centroid_1101 4.9437E-06
centroid_11016	SIS domain protein	1	8	100	16	centroid_1101	25.7128117	3.96188E-07	centroid_1101 4.9437E-06
centroid_11017	ADP-ribosylglycohydrolase family protein	1	8	100	16	centroid_1101	25.7128117	3.96188E-07	centroid_1101 4.9437E-06
centroid_11018	bacterial regulatory, gntR family protein	1	8	100	16	centroid_1101	25.7128117	3.96188E-07	centroid_1101 4.9437E-06
centroid_11019	MFS/sugar transport family protein	1	8	100	16	centroid_1101	25.7128117	3.96188E-07	centroid_1101 4.9437E-06
centroid_11020	MFS/sugar transport family protein	1	8	100	16	centroid_1102	25.7128117	3.96188E-07	centroid_1102 4.9437E-06
centroid_13089	esterase-like activity of phytase family protein	1	8	100	16	centroid_1308	25.7128117	3.96188E-07	centroid_1308 4.9437E-06
centroid_13090	(Glycoside-Pentoside-Hexuronide) transporter domain p	1	8	100	16	centroid_1309	25.7128117	3.96188E-07	centroid_1309 4.9437E-06
centroid_13254	ATP-binding region ATPase -containing domain protein	1	8	100	16	centroid_1325	25.7128117	3.96188E-07	centroid_1325 4.9437E-06

centroid_13277	putative 50S ribosomal protein L1	1	8	100	16	centroid_1327	25.7128117	3.96188E-07	centroid_1327	4.9437E-06
centroid_13278	esterase-like activity of phytase family protein	1	8	100	16	centroid_1327	25.7128117	3.96188E-07	centroid_1327	4.9437E-06
centroid_14479	transposase IS66 family protein	1	8	100	16	centroid_1447	25.7128117	3.96188E-07	centroid_1447	4.9437E-06
centroid_15935	ATP-binding region ATPase -containing domain protein	1	8	100	16	centroid_1593	25.7128117	3.96188E-07	centroid_1593	4.9437E-06
centroid_6493	prophage CP4-57 regulatory family protein	1	8	100	16	centroid_649	25.7128117	3.96188E-07	centroid_649	4.9437E-06
centroid_6818	fibronectin type III family protein	1	8	100	16	centroid_681	25.7128117	3.96188E-07	centroid_681	4.9437E-06
centroid_8920	protein TolA	1	8	100	16	centroid_892	25.7128117	3.96188E-07	centroid_892	4.9437E-06
centroid_8921	tolA C-terminal family protein	1	8	100	16	centroid_892	25.7128117	3.96188E-07	centroid_892	4.9437E-06
centroid_13880	gram-negative porin family protein	1	7	100	17	centroid_1388	21.212171	4.11145E-06	centroid_1388	3.0346E-05
centroid_15118	conserved hypothetical protein	1	7	100	17	centroid_1511	21.212171	4.11145E-06	centroid_1511	3.0346E-05
centroid_7368	phage tail tape measure protein, TP901 family, core regior	1	7	100	17	centroid_736	21.212171	4.11145E-06	centroid_736	3.0346E-05
centroid_9473	outer membrane protein N	1	7	100	17	centroid_947	21.212171	4.11145E-06	centroid_947	3.0346E-05
centroid_11096	putative type III secretion protein	1	6	100	18	centroid_1109	16.8487798	4.04793E-05	centroid_1109	0.00017482
centroid_13103	type-1 fimbrial protein, A chain	1	6	100	18	centroid_1310	16.8487798	4.04793E-05	centroid_1310	0.00017482
centroid_13879	conserved hypothetical protein	1	6	100	18	centroid_1387	16.8487798	4.04793E-05	centroid_1387	0.00017482
centroid_18590	hypothetical protein	1	6	100	18	centroid_1859	16.8487798	4.04793E-05	centroid_1859	0.00017482
centroid_5812	conserved hypothetical protein	0	24	101	0	centroid_581	118.637144	1.25752E-27	centroid_581	3.1064E-26
centroid_5883	laminin-binding fimbrial subunit ElfA	0	22	101	2	centroid_588	106.126516	6.91748E-25	centroid_588	1.6318E-22
centroid_14960	outer membrane autotransporter barrel domain protein	0	20	101	4	centroid_1496	94.0939379	3.00899E-22	centroid_1496	1.4849E-19
centroid_8902	conserved hypothetical protein	0	20	101	4	centroid_890	94.0939379	3.00899E-22	centroid_890	1.4849E-19
centroid_13110	outer membrane autotransporter barrel domain protein	0	19	101	5	centroid_1311	88.2486029	5.7724E-21	centroid_1311	3.148E-18
centroid_5572	outer membrane autotransporter barrel domain protein	0	19	101	5	centroid_557	88.2486029	5.7724E-21	centroid_557	3.148E-18
centroid_5620	inner membrane protein YhaI	0	19	101	5	centroid_562	88.2486029	5.7724E-21	centroid_562	3.148E-18
centroid_9422	putative membrane protein	0	19	101	5	centroid_942	88.2486029	5.7724E-21	centroid_942	3.148E-18
centroid_12982	sel1 repeat family protein	0	18	101	6	centroid_1298	82.5130769	1.04973E-19	centroid_1298	5.614E-17
centroid_5581	putative membrane protein	0	18	101	6	centroid_558	82.5130769	1.04973E-19	centroid_558	5.614E-17
centroid_5660	sel1 repeat family protein	0	18	101	6	centroid_566	82.5130769	1.04973E-19	centroid_566	5.614E-17
centroid_5661	acetyltransferase domain protein	0	18	101	6	centroid_566	82.5130769	1.04973E-19	centroid_566	5.614E-17
centroid_5662	conserved hypothetical protein	0	18	101	6	centroid_566	82.5130769	1.04973E-19	centroid_566	5.614E-17
centroid_5819	conserved hypothetical protein	0	18	101	6	centroid_581	82.5130769	1.04973E-19	centroid_581	5.614E-17
centroid_5820	helix-turn-helix family protein	0	18	101	6	centroid_582	82.5130769	1.04973E-19	centroid_582	5.614E-17
centroid_5821	putative transcription elongation factor GreB	0	18	101	6	centroid_582	82.5130769	1.04973E-19	centroid_582	5.614E-17
centroid_5822	alpha/beta hydrolase fold family protein	0	18	101	6	centroid_582	82.5130769	1.04973E-19	centroid_582	5.614E-17
centroid_5891	protein DedA	0	18	101	6	centroid_589	82.5130769	1.04973E-19	centroid_589	5.614E-17
centroid_7345	aldo/keto reductase family protein	0	18	101	6	centroid_734	82.5130769	1.04973E-19	centroid_734	5.614E-17
centroid_17233	rate uptake ABC transporter 2 (CUT2) family, ATP-bindir	0	17	101	7	centroid_1723	76.884406	1.81263E-18	centroid_1723	8.6616E-16
centroid_17234	ABC transporter family protein	0	17	101	7	centroid_1723	76.884406	1.81263E-18	centroid_1723	8.6616E-16
centroid_5651	ASCH domain protein	0	17	101	7	centroid_565	76.884406	1.81263E-18	centroid_565	8.6616E-16
centroid_5652	putative LACI-type transcriptional regulator	0	17	101	7	centroid_565	76.884406	1.81263E-18	centroid_565	8.6616E-16
centroid_5653	heme ABC exporter, ATP-binding protein CcmA	0	17	101	7	centroid_565	76.884406	1.81263E-18	centroid_565	8.6616E-16
centroid_5654	ain amino acid transport system / permease component	0	17	101	7	centroid_565	76.884406	1.81263E-18	centroid_565	8.6616E-16
centroid_5655	ain amino acid transport system / permease component	0	17	101	7	centroid_565	76.884406	1.81263E-18	centroid_565	8.6616E-16
centroid_5813	putative membrane protein	0	17	101	7	centroid_581	76.884406	1.81263E-18	centroid_581	8.6616E-16
centroid_5873	conserved hypothetical protein	0	17	101	7	centroid_587	76.884406	1.81263E-18	centroid_587	8.6616E-16
centroid_12984	bacterial Ig-like domain family protein	0	16	101	8	centroid_1298	71.3597684	2.9768E-17	centroid_1298	1.1801E-14
centroid_5623	conserved hypothetical protein	0	16	101	8	centroid_562	71.3597684	2.9768E-17	centroid_562	1.1801E-14
centroid_11606	YoaG domain protein	0	15	101	9	centroid_1160	65.9364765	4.65693E-16	centroid_1160	1.4424E-13
centroid_13498	conserved hypothetical protein	0	15	101	9	centroid_1349	65.9364765	4.65693E-16	centroid_1349	1.4424E-13
centroid_15497	outer membrane autotransporter barrel domain protein	0	15	101	9	centroid_1549	65.9364765	4.65693E-16	centroid_1549	1.4424E-13
centroid_15498	nded Signal Peptide of Type V secretion system family pr	0	15	101	9	centroid_1549	65.9364765	4.65693E-16	centroid_1549	1.4424E-13
centroid_5647	outer membrane autotransporter barrel domain protein	0	15	101	9	centroid_564	65.9364765	4.65693E-16	centroid_564	1.4424E-13
centroid_5659	conserved hypothetical protein	0	15	101	9	centroid_565	65.9364765	4.65693E-16	centroid_565	1.4424E-13
centroid_5875	conserved hypothetical protein	0	15	101	9	centroid_587	65.9364765	4.65693E-16	centroid_587	1.4424E-13
centroid_5876	hicB family protein	0	15	101	9	centroid_587	65.9364765	4.65693E-16	centroid_587	1.4424E-13
centroid_8976	conserved hypothetical protein	0	15	101	9	centroid_897	65.9364765	4.65693E-16	centroid_897	1.4424E-13
centroid_18234	lemA family protein	0	14	101	10	centroid_1823	60.6119817	6.95096E-15	centroid_1823	1.6011E-12
centroid_18643	RHS repeat-associated core domain protein	0	14	101	10	centroid_1864	60.6119817	6.95096E-15	centroid_1864	1.6011E-12
centroid_5853	-negative pili assembly chaperone, N-terminal domain pr	0	14	101	10	centroid_585	60.6119817	6.95096E-15	centroid_585	1.6011E-12
centroid_5681	lemA family protein	0	13	101	11	centroid_568	55.3838847	9.9146E-14	centroid_568	1.6302E-11
centroid_9079	(Glycoside-Pentoside-Hexuronide) transporter domain p	0	13	101	11	centroid_907	55.3838847	9.9146E-14	centroid_907	1.6302E-11
centroid_9080	helix-turn-helix domain protein	0	13	101	11	centroid_908	55.3838847	9.9146E-14	centroid_908	1.6302E-11
centroid_9576	conserved hypothetical protein	0	13	101	11	centroid_957	55.3838847	9.9146E-14	centroid_957	1.6302E-11
centroid_10088	conserved hypothetical protein	0	12	101	12	centroid_1008	50.2499529	1.35358E-12	centroid_1008	1.5351E-10
centroid_12889	bacterial regulatory , arsR family protein	0	12	101	12	centroid_1288	50.2499529	1.35358E-12	centroid_1288	1.5351E-10
centroid_13466	RHS repeat-associated core domain protein	0	12	101	12	centroid_1346	50.2499529	1.35358E-12	centroid_1346	1.5351E-10
centroid_5604	pfkB carbohydrate kinase family protein	0	12	101	12	centroid_560	50.2499529	1.35358E-12	centroid_560	1.5351E-10
centroid_5816	DKNYY family protein	0	12	101	12	centroid_581	50.2499529	1.35358E-12	centroid_581	1.5351E-10
centroid_7241	alpha/beta hydrolase family protein	0	12	101	12	centroid_724	50.2499529	1.35358E-12	centroid_724	1.5351E-10
centroid_7242	NAD dependent epimerase/dehydratase family protein	0	12	101	12	centroid_724	50.2499529	1.35358E-12	centroid_724	1.5351E-10
centroid_7386	conserved hypothetical protein	0	12	101	12	centroid_738	50.2499529	1.35358E-12	centroid_738	1.5351E-10
centroid_9337	putative carboxymethylenebutenolidase	0	12	101	12	centroid_933	50.2499529	1.35358E-12	centroid_933	1.5351E-10
centroid_9338	X-Pro dipeptidyl-peptidase family protein	0	12	101	12	centroid_933	50.2499529	1.35358E-12	centroid_933	1.5351E-10
centroid_12888	pfkB carbohydrate kinase family protein	0	11	101	13	centroid_1288	45.2081495	1.77166E-11	centroid_1288	1.3461E-09
centroid_11474	penicillin amidase family protein	0	10	101	14	centroid_1147	40.2566836	2.22692E-10	centroid_1147	1.1058E-08
centroid_13225	RHS repeat-associated core domain protein	0	10	101	14	centroid_1322	40.2566836	2.22692E-10	centroid_1322	1.1058E-08
centroid_15860	penicillin amidase family protein	0	10	101	14	centroid_1586	40.2566836	2.22692E-10	centroid_1586	1.1058E-08
centroid_17597	RHS Repeat family protein	0	10	101	14	centroid_1759	40.2566836	2.22692E-10	centroid_1759	1.1058E-08
centroid_18222	-negative pili assembly chaperone, C-terminal domain pr	0	10	101	14	centroid_1822	40.2566836	2.22692E-10	centroid_1822	1.1058E-08
centroid_5682	repair family protein	0	10	101	14	centroid_568	40.2566836	2.22692E-10	centroid_568	1.1058E-08
centroid_5683	hypothetical protein	0	10	101	14	centroid_568	40.2566836	2.22692E-10	centroid_568	1.1058E-08
centroid_7531	RHS repeat-associated core domain protein	0	10	101	14	centroid_753	40.2566836	2.22692E-10	centroid_753	1.1058E-08
centroid_9988	penicillin amidase family protein	0	10	101	14	centroid_998	40.2566836	2.22692E-10	centroid_998	1.1058E-08
centroid_12863	putative aTP-binding component of a transport system	0	9	101	15	centroid_1286	35.3940959	2.69302E-09	centroid_1286	8.5512E-08
centroid_14398	conserved hypothetical protein	0	9	101	15	centroid_1439	35.3940959	2.69302E-09	centroid_1439	8.5512E-08
centroid_16094	phage integrase family protein	0	9	101	15	centroid_1609	35.3940959	2.69302E-09	centroid_1609	8.5512E-08
centroid_16130	phage integrase family protein	0	9	101	15	centroid_1613	35.3940959	2.69302E-09	centroid_1613	8.5512E-08
centroid_5658	outer membrane autotransporter barrel domain protein	0	9	101	15	centroid_565	35.3940959	2.69302E-09	centroid_565	8.5512E-08
centroid_5932	putative membrane protein	0	9	101	15	centroid_593	35.3940959	2.69302E-09	centroid_593	8.5512E-08
centroid_7398	RHS repeat-associated core domain protein	0	9	101	15	centroid_739	35.3940959	2.69302E-09	centroid_739	8.5512E-08
centroid_7546	RHS repeat-associated core domain protein	0	9	101	15	centroid_754	35.3940959	2.69302E-09	centroid_754	8.5512E-08
centroid_9014	outer membrane autotransporter barrel domain protein	0	9	101	15	centroid_901	35.3940959	2.69302E-09	centroid_901	8.5512E-08
centroid_9015	conserved hypothetical protein	0	9	101	15	centroid_901	35.3940959	2.69302E-09	centroid_901	8.5512E-08
centroid_9016	conserved hypothetical protein	0	9	101	15	centroid_901	35.3940959	2.69302E-09	centroid_901	8.5512E-08
centroid_9018	phage integrase family protein	0	9	101	15	centroid_901	35.3940959	2.69302E-09	centroid_901	8.5512E-08
centroid_9019	conserved hypothetical protein	0	9	101	15	centroid_901	35.3940959	2.69302E-09	centroid_901	8.5512E-08
centroid_13101	penicillin G acylase	0	8	101	16	centroid_1310	30.6194119	3.13937E-08	centroid_1310	6.2531E-07
centroid_14214	RHS repeat-associated core domain protein	0	8	101	16	centroid_1421	30.6194119	3.13937E-08	centroid_1421	6.2531E-07
centroid_16129	kinase-, DNA gyrase B-, and HSP90-like ATPase family	0	8	101	16	centroid_1612	30.6194119	3.13937E-08	centroid_1612	6.2531E-07
centroid_5869	ROS/MUCR transcriptional regulator family protein	0	8	101	16	centroid_586	30.6194119	3.13937E-08	centroid_586	6.2531E-07
centroid_5931	conserved hypothetical protein	0	8	101	16	centroid_593	30.6194119	3.13937E-08	centroid_593	6.2531E-07
centroid_5933	conserved hypothetical protein	0	8	101	16	centroid_593	30.6194119	3.13937E-08	centroid_593	6.2531E-07
centroid_5974	conserved hypothetical protein	0	8	101	16	centroid_597	30.6194119	3.13937E-08	centroid_597	6.2531E-07
centroid_5975	conserved hypothetical protein	0	8	101	16	centroid_597	30.6194119	3.13937E-08	centroid_597	6.2531E-07
centroid_7389	impA domain family protein	0	8	101	16	centroid_738	30.6194119	3.13937E-08	centroid_738	6.2531E-07
centroid_9017	bacterial regulatory, luxR family protein	0	8	101	16	centroid_901	30.6194119	3.13937E-08	centroid_901	6.2531E-07
centroid_10047	conserved hypothetical protein	0	7	101	17	centroid_1004	25.9324288	3.53579E-07	centroid_1004	4.3404E-06
centroid_10084	RHS repeat-associated core domain protein	0	7	101	17	centroid_1008	25.9324288	3.53579E-07	centroid_1008	4.3404E-06

centroid_13252	fibronectin type III family protein	0	7	101	17	centroid_1325	25.9324288	3.53579E-07	centroid_1325	4.3404E-06
centroid_14463	RHS repeat-associated core domain protein	0	7	101	17	centroid_1446	25.9324288	3.53579E-07	centroid_1446	4.3404E-06
centroid_15179	RHS repeat-associated core domain protein	0	7	101	17	centroid_1517	25.9324288	3.53579E-07	centroid_1517	4.3404E-06
centroid_6837	hypothetical protein	0	7	101	17	centroid_6837	25.9324288	3.53579E-07	centroid_6837	4.3404E-06
centroid_7198	UTRA domain protein	0	7	101	17	centroid_7198	25.9324288	3.53579E-07	centroid_7198	4.3404E-06
centroid_7199	uvate-dependent sugar phosphotransferase system, EIIA	0	7	101	17	centroid_7199	25.9324288	3.53579E-07	centroid_7199	4.3404E-06
centroid_7200	PTS system, Lactose/Cellobiose specific IIB subunit	0	7	101	17	centroid_7200	25.9324288	3.53579E-07	centroid_7200	4.3404E-06
centroid_7201	S system sugar-specific permease component family pro	0	7	101	17	centroid_7201	25.9324288	3.53579E-07	centroid_7201	4.3404E-06
centroid_7202	GY family of carbohydrate kinase, N-terminal domain pro	0	7	101	17	centroid_7202	25.9324288	3.53579E-07	centroid_7202	4.3404E-06
centroid_7203	phosphotransferase system, HPr-related proteins	0	7	101	17	centroid_7203	25.9324288	3.53579E-07	centroid_7203	4.3404E-06
centroid_7204	ketose-bisphosphate aldolase family protein	0	7	101	17	centroid_7204	25.9324288	3.53579E-07	centroid_7204	4.3404E-06
centroid_7381	fil domain protein	0	7	101	17	centroid_7381	25.9324288	3.53579E-07	centroid_7381	4.3404E-06
centroid_7390	conserved hypothetical protein	0	7	101	17	centroid_7390	25.9324288	3.53579E-07	centroid_7390	4.3404E-06
centroid_7391	sigma-54 interaction domain protein	0	7	101	17	centroid_7391	25.9324288	3.53579E-07	centroid_7391	4.3404E-06
centroid_9444	PTS family galactitol porter, component IIC domain protein	0	7	101	17	centroid_9444	25.9324288	3.53579E-07	centroid_9444	4.3404E-06
centroid_9445	S system sugar-specific permease component family pro	0	7	101	17	centroid_9445	25.9324288	3.53579E-07	centroid_9445	4.3404E-06
centroid_9446	conserved hypothetical protein	0	7	101	17	centroid_9446	25.9324288	3.53579E-07	centroid_9446	4.3404E-06
centroid_10065	ash family protein	0	6	101	18	centroid_1006	21.3342943	3.85768E-06	centroid_1006	2.8695E-05
centroid_14364	conserved hypothetical protein	0	6	101	18	centroid_1436	21.3342943	3.85768E-06	centroid_1436	2.8695E-05
centroid_17227	ash family protein	0	6	101	18	centroid_1722	21.3342943	3.85768E-06	centroid_1722	2.8695E-05
centroid_17382	RHS repeat-associated core domain protein	0	6	101	18	centroid_1738	21.3342943	3.85768E-06	centroid_1738	2.8695E-05
centroid_5624	DKNYY family protein	0	6	101	18	centroid_5624	21.3342943	3.85768E-06	centroid_5624	2.8695E-05
centroid_5625	conserved hypothetical protein	0	6	101	18	centroid_5625	21.3342943	3.85768E-06	centroid_5625	2.8695E-05
centroid_5722	conserved hypothetical protein	0	6	101	18	centroid_5722	21.3342943	3.85768E-06	centroid_5722	2.8695E-05
centroid_5892	3-hydroxybutyrate dehydrogenase family protein	0	6	101	18	centroid_5892	21.3342943	3.85768E-06	centroid_5892	2.8695E-05
centroid_5893	citrate transporter family protein	0	6	101	18	centroid_5893	21.3342943	3.85768E-06	centroid_5893	2.8695E-05
centroid_5894	ydroxyacyl-CoA dehydrogenase, NAD binding domain pro	0	6	101	18	centroid_5894	21.3342943	3.85768E-06	centroid_5894	2.8695E-05
centroid_5895	acetyl-CoA C-acetyltransferase family protein	0	6	101	18	centroid_5895	21.3342943	3.85768E-06	centroid_5895	2.8695E-05
centroid_5896	3-oxoacid CoA-transferase, B subunit	0	6	101	18	centroid_5896	21.3342943	3.85768E-06	centroid_5896	2.8695E-05
centroid_5897	3-oxoacid CoA-transferase, A subunit	0	6	101	18	centroid_5897	21.3342943	3.85768E-06	centroid_5897	2.8695E-05
centroid_5898	bacterial regulatory helix-turn-helix , lysR family protein	0	6	101	18	centroid_5898	21.3342943	3.85768E-06	centroid_5898	2.8695E-05
centroid_5934	e kinase-, DNA gyrase B-, and HSP90-like ATPase family	0	6	101	18	centroid_5934	21.3342943	3.85768E-06	centroid_5934	2.8695E-05
centroid_6103	caudovirales tail fibre assembly family protein	0	6	101	18	centroid_6103	21.3342943	3.85768E-06	centroid_6103	2.8695E-05
centroid_8833	tetratricopeptide repeat family protein	0	6	101	18	centroid_8833	21.3342943	3.85768E-06	centroid_8833	2.8695E-05
centroid_8859	conserved hypothetical protein	0	6	101	18	centroid_8859	21.3342943	3.85768E-06	centroid_8859	2.8695E-05
centroid_9418	helix-turn-helix domain protein	0	6	101	18	centroid_9418	21.3342943	3.85768E-06	centroid_9418	2.8695E-05
centroid_9447	conserved hypothetical protein	0	6	101	18	centroid_9447	21.3342943	3.85768E-06	centroid_9447	2.8695E-05
centroid_10142	CRISPR type I-E/ECOLI-associated protein CasB/Cse2	0	5	101	19	centroid_1014	16.8287825	4.09081E-05	centroid_1014	0.00018123
centroid_11585	AAA domain family protein	0	5	101	19	centroid_1158	16.8287825	4.09081E-05	centroid_1158	0.00018123
centroid_11597	hcp domain protein	0	5	101	19	centroid_1159	16.8287825	4.09081E-05	centroid_1159	0.00018123
centroid_11763	fibronectin type III family protein	0	5	101	19	centroid_1176	16.8287825	4.09081E-05	centroid_1176	0.00018123
centroid_12872	prophage CP4-57 integrase	0	5	101	19	centroid_1287	16.8287825	4.09081E-05	centroid_1287	0.00018123
centroid_13004	AAA domain family protein	0	5	101	19	centroid_1300	16.8287825	4.09081E-05	centroid_1300	0.00018123
centroid_13005	C 5-methylcytosine restriction system component family p	0	5	101	19	centroid_1300	16.8287825	4.09081E-05	centroid_1300	0.00018123
centroid_13006	conserved hypothetical protein	0	5	101	19	centroid_1300	16.8287825	4.09081E-05	centroid_1300	0.00018123
centroid_13012	conserved hypothetical protein	0	5	101	19	centroid_1301	16.8287825	4.09081E-05	centroid_1301	0.00018123
centroid_13013	conserved hypothetical protein	0	5	101	19	centroid_1301	16.8287825	4.09081E-05	centroid_1301	0.00018123
centroid_13014	kinase domain protein	0	5	101	19	centroid_1301	16.8287825	4.09081E-05	centroid_1301	0.00018123
centroid_13015	phage integrase family protein	0	5	101	19	centroid_1301	16.8287825	4.09081E-05	centroid_1301	0.00018123
centroid_13470	conserved hypothetical protein	0	5	101	19	centroid_1347	16.8287825	4.09081E-05	centroid_1347	0.00018123
centroid_13509	putative replication protein from bacteriophage origin	0	5	101	19	centroid_1350	16.8287825	4.09081E-05	centroid_1350	0.00018123
centroid_14354	conserved hypothetical protein	0	5	101	19	centroid_1435	16.8287825	4.09081E-05	centroid_1435	0.00018123
centroid_14355	leucine rich repeat family protein	0	5	101	19	centroid_1435	16.8287825	4.09081E-05	centroid_1435	0.00018123
centroid_15005	putative membrane protein	0	5	101	19	centroid_1500	16.8287825	4.09081E-05	centroid_1500	0.00018123
centroid_15006	conserved hypothetical protein	0	5	101	19	centroid_1500	16.8287825	4.09081E-05	centroid_1500	0.00018123
centroid_15016	conserved hypothetical protein	0	5	101	19	centroid_1501	16.8287825	4.09081E-05	centroid_1501	0.00018123
centroid_16137	putative tail length tape measure domain protein	0	5	101	19	centroid_1613	16.8287825	4.09081E-05	centroid_1613	0.00018123
centroid_17365	conserved hypothetical protein	0	5	101	19	centroid_1736	16.8287825	4.09081E-05	centroid_1736	0.00018123
centroid_17373	phage integrase family protein	0	5	101	19	centroid_1737	16.8287825	4.09081E-05	centroid_1737	0.00018123
centroid_17374	conserved hypothetical protein	0	5	101	19	centroid_1737	16.8287825	4.09081E-05	centroid_1737	0.00018123
centroid_17584	conserved hypothetical protein	0	5	101	19	centroid_1758	16.8287825	4.09081E-05	centroid_1758	0.00018123
centroid_17760	type III restriction enzyme, res subunit	0	5	101	19	centroid_1776	16.8287825	4.09081E-05	centroid_1776	0.00018123
centroid_17761	helicase conserved C-terminal domain protein	0	5	101	19	centroid_1776	16.8287825	4.09081E-05	centroid_1776	0.00018123
centroid_17762	ATP-dependent DNA helicase, RecQ family domain protein	0	5	101	19	centroid_1776	16.8287825	4.09081E-05	centroid_1776	0.00018123
centroid_17795	leucine rich repeat family protein	0	5	101	19	centroid_1779	16.8287825	4.09081E-05	centroid_1779	0.00018123
centroid_17835	putative z1097 gene product	0	5	101	19	centroid_1783	16.8287825	4.09081E-05	centroid_1783	0.00018123
centroid_18529	conserved hypothetical protein	0	5	101	19	centroid_1852	16.8287825	4.09081E-05	centroid_1852	0.00018123
centroid_5606	sigma-54 interaction domain protein	0	5	101	19	centroid_5606	16.8287825	4.09081E-05	centroid_5606	0.00018123
centroid_5607	PRD domain protein	0	5	101	19	centroid_5607	16.8287825	4.09081E-05	centroid_5607	0.00018123
centroid_5608	PTS system fructose IIA component family protein	0	5	101	19	centroid_5608	16.8287825	4.09081E-05	centroid_5608	0.00018123
centroid_5609	PTS system sorbose subIIB component family protein	0	5	101	19	centroid_5609	16.8287825	4.09081E-05	centroid_5609	0.00018123
centroid_5610	PTS system sorbose-specific iic component family protein	0	5	101	19	centroid_5610	16.8287825	4.09081E-05	centroid_5610	0.00018123
centroid_5611	ystem mannose/fructose/sorbose IID component family p	0	5	101	19	centroid_5611	16.8287825	4.09081E-05	centroid_5611	0.00018123
centroid_5612	L-seryl-tRNA selenium transferase family protein	0	5	101	19	centroid_5612	16.8287825	4.09081E-05	centroid_5612	0.00018123
centroid_5613	2-dehydro-3-deoxyphosphogluconate aldolase	0	5	101	19	centroid_5613	16.8287825	4.09081E-05	centroid_5613	0.00018123
centroid_5972	type IV/VI secretion system , DotU family domain protein	0	5	101	19	centroid_5972	16.8287825	4.09081E-05	centroid_5972	0.00018123
centroid_5973	conserved hypothetical protein	0	5	101	19	centroid_5973	16.8287825	4.09081E-05	centroid_5973	0.00018123
centroid_6022	conserved hypothetical protein	0	5	101	19	centroid_6022	16.8287825	4.09081E-05	centroid_6022	0.00018123
centroid_6102	side tail fiber family protein	0	5	101	19	centroid_6102	16.8287825	4.09081E-05	centroid_6102	0.00018123
centroid_7351	CRISPR type I-E/ECOLI-associated protein CasA/Cse1	0	5	101	19	centroid_7351	16.8287825	4.09081E-05	centroid_7351	0.00018123
centroid_7551	conserved hypothetical protein	0	5	101	19	centroid_7551	16.8287825	4.09081E-05	centroid_7551	0.00018123
centroid_8767	conserved hypothetical protein	0	5	101	19	centroid_8767	16.8287825	4.09081E-05	centroid_8767	0.00018123
centroid_8785	conserved hypothetical protein	0	5	101	19	centroid_8785	16.8287825	4.09081E-05	centroid_8785	0.00018123
centroid_8801	hypothetical protein	0	5	101	19	centroid_8801	16.8287825	4.09081E-05	centroid_8801	0.00018123
centroid_8802	conserved hypothetical protein	0	5	101	19	centroid_8802	16.8287825	4.09081E-05	centroid_8802	0.00018123
centroid_8816	beta-ketoacyl synthase, N-terminal domain protein	0	5	101	19	centroid_8816	16.8287825	4.09081E-05	centroid_8816	0.00018123
centroid_8817	short chain dehydrogenase family protein	0	5	101	19	centroid_8817	16.8287825	4.09081E-05	centroid_8817	0.00018123
centroid_8818	fabA-like domain protein	0	5	101	19	centroid_8818	16.8287825	4.09081E-05	centroid_8818	0.00018123
centroid_8819	beta-ketoacyl synthase, C-terminal domain protein	0	5	101	19	centroid_8819	16.8287825	4.09081E-05	centroid_8819	0.00018123
centroid_8820	conserved hypothetical protein	0	5	101	19	centroid_8820	16.8287825	4.09081E-05	centroid_8820	0.00018123
centroid_8821	MMPL family protein	0	5	101	19	centroid_8821	16.8287825	4.09081E-05	centroid_8821	0.00018123
centroid_8822	outer membrane lipocarrier LolA family protein	0	5	101	19	centroid_8822	16.8287825	4.09081E-05	centroid_8822	0.00018123
centroid_8823	thioesterase superfamily protein	0	5	101	19	centroid_8823	16.8287825	4.09081E-05	centroid_8823	0.00018123
centroid_8824	glycosyl transferase 2 family protein	0	5	101	19	centroid_8824	16.8287825	4.09081E-05	centroid_8824	0.00018123
centroid_8828	phosphopantetheine attachment site family protein	0	5	101	19	centroid_8828	16.8287825	4.09081E-05	centroid_8828	0.00018123
centroid_8829	phosphopantetheine attachment site family protein	0	5	101	19	centroid_8829	16.8287825	4.09081E-05	centroid_8829	0.00018123
centroid_8830	beta-ketoacyl synthase, N-terminal domain protein	0	5	101	19	centroid_8830	16.8287825	4.09081E-05	centroid_8830	0.00018123
centroid_8831	O-methyltransferase family protein	0	5	101	19	centroid_8831	16.8287825	4.09081E-05	centroid_8831	0.00018123
centroid_8834	ATP-dependent DNA helicase, RecQ family protein	0	5	101	19	centroid_8834	16.8287825	4.09081E-05	centroid_8834	0.00018123
centroid_8835	DNA recombination-mediator A family protein	0	5	101	19	centroid_8835	16.8287825	4.09081E-05	centroid_8835	0.00018123
centroid_8895	conserved hypothetical protein	0	5	101	19	centroid_8895	16.8287825	4.09081E-05	centroid_8895	0.00018123
centroid_8896	tonB-dependent Receptor Plug domain protein	0	5	101	19	centroid_8896	16.8287825	4.09081E-05	centroid_8896	0.00018123
centroid_8897	olinate phosphoribosyl transferase, C-terminal domain pro	0	5	101	19	centroid_8897	16.8287825	4.09081E-05	centroid_8897	0.00018123
centroid_8898	methyltransferase domain protein	0	5	101	19	centroid_8898	16.8287825	4.09081E-05	centroid_8898	0.00018123
centroid_8899	ABC transporter family protein	0	5	101	19	centroid_8899	16.8287825	4.09081E-05	centroid_8899	0.00018123
centroid_8900	fecCD transport family protein	0	5	101	19	centroid_8900	16.8287825	4.09081E-05	centroid_8900	0.00018123
centroid_8901	periplasmic binding family protein	0	5	101	19	centroid_8901	16.8287825	4.09081E-05	centroid_8901	0.00018123

centroid_8947	outer membrane protein C	0	5	101	19	centroid_8947	16.8287825	4.09081E-05	centroid_8947	0.00018123
centroid_8958	type VII secretion system (T7SS), usher family protein	0	5	101	19	centroid_8958	16.8287825	4.09081E-05	centroid_8958	0.00018123
centroid_8959	fimbrial family protein	0	5	101	19	centroid_8959	16.8287825	4.09081E-05	centroid_8959	0.00018123
centroid_9082	fimbrial family protein	0	5	101	19	centroid_9082	16.8287825	4.09081E-05	centroid_9082	0.00018123
centroid_9084	type VII secretion system (T7SS), usher family protein	0	5	101	19	centroid_9084	16.8287825	4.09081E-05	centroid_9084	0.00018123
centroid_9085	fimbrial family protein	0	5	101	19	centroid_9085	16.8287825	4.09081E-05	centroid_9085	0.00018123
centroid_9086	fimbrial family protein	0	5	101	19	centroid_9086	16.8287825	4.09081E-05	centroid_9086	0.00018123
centroid_9106	conserved hypothetical protein	0	5	101	19	centroid_9106	16.8287825	4.09081E-05	centroid_9106	0.00018123
centroid_9221	conserved hypothetical protein	0	5	101	19	centroid_9221	16.8287825	4.09081E-05	centroid_9221	0.00018123
centroid_9252	major Facilitator Superfamily protein	0	5	101	19	centroid_9252	16.8287825	4.09081E-05	centroid_9252	0.00018123
centroid_9262	type VII secretion system (T7SS), usher family protein	0	5	101	19	centroid_9262	16.8287825	4.09081E-05	centroid_9262	0.00018123
centroid_9263	type VII secretion system (T7SS), usher family protein	0	5	101	19	centroid_9263	16.8287825	4.09081E-05	centroid_9263	0.00018123
centroid_9427	phage tail fibre repeat family protein	0	5	101	19	centroid_9427	16.8287825	4.09081E-05	centroid_9427	0.00018123
centroid_9457	n-negative pili assembly chaperone, N-terminal domain pr	0	5	101	19	centroid_9457	16.8287825	4.09081E-05	centroid_9457	0.00018123
centroid_9973	acyltransferase family protein	0	5	101	19	centroid_9973	16.8287825	4.09081E-05	centroid_9973	0.00018123

Table S5. Distribution by Phylogroup B

Gene_ID	Annotation	Phylogroup B_present	Other_present	Phylogroup B_Absent	Other_Absent	Gene_ID	chisq-stats	pvalues	Gene_ID	pvalues
centroid_5620	inner membrane protein YhaI	19	1	0	106	centroid_5620	111.278649	5.14E-26	centroid_5620	1.26E-21
centroid_13023	fimbrial family protein	19	2	0	105	centroid_1302	104.915298	1.27E-24	centroid_1302	1.32E-20
centroid_14960	outer membrane autotransporter barrel domain protein	19	2	0	105	centroid_1496	104.915298	1.27E-24	centroid_1496	1.32E-20
centroid_16055	n-negative pili assembly chaperone, N-terminal domain pr	19	2	0	105	centroid_1605	104.915298	1.27E-24	centroid_1605	1.32E-20
centroid_16056	n-negative pili assembly chaperone, C-terminal domain pr	19	2	0	105	centroid_1605	104.915298	1.27E-24	centroid_1605	1.32E-20
centroid_18522	type VII secretion system (T7SS), usher family protein	19	2	0	105	centroid_1852	104.915298	1.27E-24	centroid_1852	1.32E-20
centroid_18523	type VII secretion system (T7SS), usher family protein	19	2	0	105	centroid_1852	104.915298	1.27E-24	centroid_1852	1.32E-20
centroid_5575	inner membrane protein YmfA	19	2	0	105	centroid_5575	104.915298	1.27E-24	centroid_5575	1.32E-20
centroid_5621	cblD like pilus biogenesis initiator family protein	19	2	0	105	centroid_5621	104.915298	1.27E-24	centroid_5621	1.32E-20
centroid_5622	fimbrial family protein	19	2	0	105	centroid_5622	104.915298	1.27E-24	centroid_5622	1.32E-20
centroid_5684	fimbrial family protein	19	2	0	105	centroid_5684	104.915298	1.27E-24	centroid_5684	1.32E-20
centroid_5685	n-negative pili assembly chaperone, C-terminal domain pr	19	2	0	105	centroid_5685	104.915298	1.27E-24	centroid_5685	1.32E-20
centroid_5686	type VII secretion system (T7SS), usher family protein	19	2	0	105	centroid_5686	104.915298	1.27E-24	centroid_5686	1.32E-20
centroid_5687	fimbrial family protein	19	2	0	105	centroid_5687	104.915298	1.27E-24	centroid_5687	1.32E-20
centroid_5846	conserved hypothetical protein	19	2	0	105	centroid_5846	104.915298	1.27E-24	centroid_5846	1.32E-20
centroid_5905	putative nucleotidyltransferase family protein	19	2	0	105	centroid_5905	104.915298	1.27E-24	centroid_5905	1.32E-20
centroid_5906	zeta toxin family protein	19	2	0	105	centroid_5906	104.915298	1.27E-24	centroid_5906	1.32E-20
centroid_14944	putative ybl55 protein	19	3	0	104	centroid_1494	99.130452	2.36E-23	centroid_1494	9.69E-20
centroid_17372	fimbrial family protein	19	3	0	104	centroid_1737	99.130452	2.36E-23	centroid_1737	9.69E-20
centroid_5577	tRNA(fMet)-specific endonuclease VapC	19	3	0	104	centroid_5577	99.130452	2.36E-23	centroid_5577	9.69E-20
centroid_5578	antitoxin VapB	19	3	0	104	centroid_5578	99.130452	2.36E-23	centroid_5578	9.69E-20
centroid_5833	haemagglutinin family protein	19	3	0	104	centroid_5833	99.130452	2.36E-23	centroid_5833	9.69E-20
centroid_5904	SPFH domain / Band 7 family protein	19	3	0	104	centroid_5904	99.130452	2.36E-23	centroid_5904	9.69E-20
centroid_7287	antitoxin VapB	19	3	0	104	centroid_7287	99.130452	2.36E-23	centroid_7287	9.69E-20
centroid_5883	laminin-binding fimbrial subunit ElfA	19	4	0	103	centroid_5883	93.8486555	3.41E-22	centroid_5883	5.57E-19
centroid_12883	LPXTG cell wall anchor domain protein	19	5	0	102	centroid_1288	89.0070274	3.93E-21	centroid_1288	2.67E-18
centroid_13024	hypothetical protein	19	5	0	102	centroid_1302	89.0070274	3.93E-21	centroid_1302	2.67E-18
centroid_14504	hypothetical protein	19	5	0	102	centroid_1450	89.0070274	3.93E-21	centroid_1450	2.67E-18
centroid_11055	H+ symporter family protein	19	6	0	101	centroid_1105	84.5527482	3.74E-20	centroid_1105	1.11E-17
centroid_11056	lacY proton/sugar symporter family protein	19	6	0	101	centroid_1105	84.5527482	3.74E-20	centroid_1105	1.11E-17
centroid_12882	H+ symporter family protein	19	6	0	101	centroid_1288	84.5527482	3.74E-20	centroid_1288	1.11E-17
centroid_5812	conserved hypothetical protein	19	6	0	101	centroid_5812	84.5527482	3.74E-20	centroid_5812	1.11E-17
centroid_5854	H+ symporter family protein	19	6	0	101	centroid_5854	84.5527482	3.74E-20	centroid_5854	1.11E-17
centroid_5855	pfkB carbohydrate kinase family protein	19	6	0	101	centroid_5855	84.5527482	3.74E-20	centroid_5855	1.11E-17
centroid_5856	sucrose-6-phosphate hydrolase family protein	19	6	0	101	centroid_5856	84.5527482	3.74E-20	centroid_5856	1.11E-17
centroid_5857	lasmic binding and sugar binding domain of LacI family pr	19	6	0	101	centroid_5857	84.5527482	3.74E-20	centroid_5857	1.11E-17
centroid_9104	H+ symporter family protein	19	6	0	101	centroid_9104	84.5527482	3.74E-20	centroid_9104	1.11E-17
centroid_14505	conserved hypothetical protein	19	8	0	99	centroid_1450	76.6340831	2.06E-18	centroid_1450	1.40E-16
centroid_5674	SPR-associated endoribonuclease Cas2, subtype I-E/EC	19	8	0	99	centroid_5674	76.6340831	2.06E-18	centroid_5674	1.40E-16
centroid_5828	putative cytoplasmic protein	19	9	0	98	centroid_5828	73.0989916	1.23E-17	centroid_5828	4.34E-16
centroid_7274	type VII secretion system (T7SS), usher family protein	19	13	0	94	centroid_7274	61.1682266	5.24E-15	centroid_7274	2.18E-14
centroid_5679	protein HokA	19	16	0	91	centroid_5679	54.0099436	1.99E-13	centroid_5679	2.55E-13
centroid_8844	ive escherichia coli IMT2125 genomic chromosome, IMT	19	17	0	90	centroid_8844	51.8890064	5.87E-13	centroid_8844	5.41E-13
centroid_11133	hypothetical protein	19	29	0	78	centroid_1113	33.3320571	7.77E-09	centroid_1113	7.26E-10
centroid_11584	biquinone/plastoquinone (complex I), various chains fami	19	29	0	78	centroid_1158	33.3320571	7.77E-09	centroid_1158	7.26E-10
centroid_5580	H-Ubiquinone oxidoreductase (complex I), chain family pr	19	29	0	78	centroid_5580	33.3320571	7.77E-09	centroid_5580	7.26E-10
centroid_7285	biquinone/plastoquinone (complex I), various chains fami	19	29	0	78	centroid_7285	33.3320571	7.77E-09	centroid_7285	7.26E-10
centroid_4390	conserved hypothetical protein	19	30	0	77	centroid_4390	32.1960381	1.39E-08	centroid_4390	1.19E-09
centroid_4717	conserved hypothetical protein	19	30	0	77	centroid_4717	32.1960381	1.39E-08	centroid_4717	1.19E-09
centroid_14250	n-negative pili assembly chaperone, N-terminal domain pr	19	31	0	76	centroid_1425	31.1054814	2.44E-08	centroid_1425	1.91E-09
centroid_14251	n-negative pili assembly chaperone, C-terminal domain pr	19	31	0	76	centroid_1425	31.1054814	2.44E-08	centroid_1425	1.91E-09
centroid_18344	outer membrane usher protein HtrE	19	31	0	76	centroid_1834	31.1054814	2.44E-08	centroid_1834	1.91E-09
centroid_18345	papC N-terminal domain protein	19	31	0	76	centroid_1834	31.1054814	2.44E-08	centroid_1834	1.91E-09
centroid_4585	n-negative pili assembly chaperone, N-terminal domain pr	19	31	0	76	centroid_4585	31.1054814	2.44E-08	centroid_4585	1.91E-09
centroid_4586	CS1 type fimbrial major subunit	19	31	0	76	centroid_4586	31.1054814	2.44E-08	centroid_4586	1.91E-09
centroid_4587	putative outer membrane fimbrial usher domain protein	19	31	0	76	centroid_4587	31.1054814	2.44E-08	centroid_4587	1.91E-09
centroid_5844	outer membrane usher protein HtrE	19	31	0	76	centroid_5844	31.1054814	2.44E-08	centroid_5844	1.91E-09
centroid_5845	fimbrial family protein	19	31	0	76	centroid_5845	31.1054814	2.44E-08	centroid_5845	1.91E-09
centroid_6098	conserved hypothetical protein	19	31	0	76	centroid_6098	31.1054814	2.44E-08	centroid_6098	1.91E-09
centroid_13286	conserved hypothetical protein	19	32	0	75	centroid_1328	30.0577136	4.19E-08	centroid_1328	3.05E-09
centroid_13577	fimbrial family protein	19	32	0	75	centroid_1357	30.0577136	4.19E-08	centroid_1357	3.05E-09
centroid_13894	type VII secretion system (T7SS), usher family protein	19	32	0	75	centroid_1389	30.0577136	4.19E-08	centroid_1389	3.05E-09
centroid_13940	type VII secretion system (T7SS), usher family protein	19	32	0	75	centroid_1394	30.0577136	4.19E-08	centroid_1394	3.05E-09
centroid_16151	fimbrial family protein	19	32	0	75	centroid_1615	30.0577136	4.19E-08	centroid_1615	3.05E-09
centroid_17669	n-negative pili assembly chaperone, N-terminal domain pr	19	32	0	75	centroid_1766	30.0577136	4.19E-08	centroid_1766	3.05E-09
centroid_4408	conserved hypothetical protein	19	32	0	75	centroid_4408	30.0577136	4.19E-08	centroid_4408	3.05E-09
centroid_5841	o-4-hydroxy-6-hydroxymethyldihydropteridine diphosphc	19	32	0	75	centroid_5841	30.0577136	4.19E-08	centroid_5841	3.05E-09
centroid_5842	fimbrial family protein	19	32	0	75	centroid_5842	30.0577136	4.19E-08	centroid_5842	3.05E-09
centroid_5843	n-negative pili assembly chaperone, N-terminal domain pr	19	32	0	75	centroid_5843	30.0577136	4.19E-08	centroid_5843	3.05E-09
centroid_8769	conserved domain protein	19	32	0	75	centroid_8769	30.0577136	4.19E-08	centroid_8769	3.05E-09
centroid_12971	conserved hypothetical protein	19	34	0	73	centroid_1297	28.0808602	1.16E-07	centroid_1297	7.49E-09
centroid_17544	conserved hypothetical protein	19	34	0	73	centroid_1754	28.0808602	1.16E-07	centroid_1754	7.49E-09
centroid_4904	methyltransferase domain protein	19	34	0	73	centroid_4904	28.0808602	1.16E-07	centroid_4904	7.49E-09
centroid_4714	insA C-terminal domain protein	19	35	0	72	centroid_4714	27.1473807	1.89E-07	centroid_4714	1.16E-08
centroid_4715	conserved hypothetical protein	19	35	0	72	centroid_4715	27.1473807	1.89E-07	centroid_4715	1.16E-08
centroid_4716	putative dsORF-f3	19	35	0	72	centroid_4716	27.1473807	1.89E-07	centroid_4716	1.16E-08
centroid_5901	putative transposase	19	35	0	72	centroid_5901	27.1473807	1.89E-07	centroid_5901	1.16E-08
centroid_5902	putative dsORF-f3	19	35	0	72	centroid_5902	27.1473807	1.89E-07	centroid_5902	1.16E-08
centroid_7724	putative dsORF-f3	19	35	0	72	centroid_7724	27.1473807	1.89E-07	centroid_7724	1.16E-08
centroid_10778	BCCT transporter family protein	19	37	0	70	centroid_1077	25.3805091	4.71E-07	centroid_1077	2.67E-08
centroid_16140	conserved hypothetical protein	19	37	0	70	centroid_1614	25.3805091	4.71E-07	centroid_1614	2.67E-08
centroid_6099	conserved hypothetical protein	19	37	0	70	centroid_6099	25.3805091	4.71E-07	centroid_6099	2.67E-08
centroid_12501	lactonase, 7-bladed beta-propeller family protein	19	38	0	69	centroid_1250	24.5436071	7.26E-07	centroid_1250	4.01E-08
centroid_12502	rhomboid family protein	19	38	0	69	centroid_1250	24.5436071	7.26E-07	centroid_1250	4.01E-08
centroid_18473	conserved hypothetical protein	19	38	0	69	centroid_1847	24.5436071	7.26E-07	centroid_1847	4.01E-08
centroid_4411	rhomboid family protein	19	38	0	69	centroid_4411	24.5436071	7.26E-07	centroid_4411	4.01E-08
centroid_9309	yqeH domain protein	19	39	0	68	centroid_9309	23.7355897	1.11E-06	centroid_9309	5.96E-08
centroid_10979	n-negative pili assembly chaperone, N-terminal domain pr	19	40	0	67	centroid_1097	22.9549892	1.66E-06	centroid_1097	8.79E-08
centroid_12391	lamB porin family protein	19	40	0	67	centroid_1239	22.9549892	1.66E-06	centroid_1239	8.79E-08

centroid_14262	phosphotransferase system, EIIC family protein	19	40	0	67	centroid_1426	22.9549892	1.66E-06	centroid_1426	8.79E-08
centroid_17336	maltoporin periplasmic N-terminal extension family protein	19	40	0	67	centroid_1733	22.9549892	1.66E-06	centroid_1733	8.79E-08
centroid_17337	lamB porin family protein	19	40	0	67	centroid_1733	22.9549892	1.66E-06	centroid_1733	8.79E-08
centroid_4617	PTS system, Lactose/Cellobiose specific IIA subunit	19	40	0	67	centroid_4617	22.9549892	1.66E-06	centroid_4617	8.79E-08
centroid_4618	maltoporin periplasmic N-terminal extension family protein	19	40	0	67	centroid_4618	22.9549892	1.66E-06	centroid_4618	8.79E-08
centroid_4619	lamB porin family protein	19	40	0	67	centroid_4619	22.9549892	1.66E-06	centroid_4619	8.79E-08
centroid_4629	conserved hypothetical protein	19	40	0	67	centroid_4629	22.9549892	1.66E-06	centroid_4629	8.79E-08
centroid_4708	n-negative pili assembly chaperone, C-terminal domain pr	19	40	0	67	centroid_4708	22.9549892	1.66E-06	centroid_4708	8.79E-08
centroid_5810	cryptic outer membrane porin BglH	19	40	0	67	centroid_5810	22.9549892	1.66E-06	centroid_5810	8.79E-08
centroid_8525	maltoporin periplasmic N-terminal extension family protein	19	40	0	67	centroid_8525	22.9549892	1.66E-06	centroid_8525	8.79E-08
centroid_8526	lamB porin family protein	19	40	0	67	centroid_8526	22.9549892	1.66E-06	centroid_8526	8.79E-08
centroid_9633	conserved hypothetical protein	19	40	0	67	centroid_9633	22.9549892	1.66E-06	centroid_9633	8.79E-08
centroid_11126	conserved hypothetical protein	19	41	0	66	centroid_1112	22.200436	2.46E-06	centroid_1112	1.29E-07
centroid_18583	type VII secretion system (T7SS), usher family protein	19	41	0	66	centroid_1858	22.200436	2.46E-06	centroid_1858	1.29E-07
centroid_4385	PBP superfamily domain protein	19	41	0	66	centroid_4385	22.200436	2.46E-06	centroid_4385	1.29E-07
centroid_4382	putative membrane protein	19	43	0	64	centroid_4382	20.764435	5.19E-06	centroid_4382	2.69E-07
centroid_4383	conserved hypothetical protein	19	43	0	64	centroid_4383	20.764435	5.19E-06	centroid_4383	2.69E-07
centroid_4384	conserved hypothetical protein	19	43	0	64	centroid_4384	20.764435	5.19E-06	centroid_4384	2.69E-07
centroid_4559	conserved hypothetical protein	19	44	0	63	centroid_4559	20.080669	7.42E-06	centroid_4559	7.71E-07
centroid_12474	bacterial Ig-like domain family protein	19	50	0	57	centroid_1247	16.3949299	5.14E-05	centroid_1247	4.30E-06
centroid_12985	conserved hypothetical protein	19	50	0	57	centroid_1298	16.3949299	5.14E-05	centroid_1298	4.30E-06
centroid_15186	hypothetical protein	19	50	0	57	centroid_1518	16.3949299	5.14E-05	centroid_1518	4.30E-06
centroid_16788	conserved hypothetical protein	19	50	0	57	centroid_1678	16.3949299	5.14E-05	centroid_1678	4.30E-06
centroid_18010	conserved hypothetical protein	19	50	0	57	centroid_1801	16.3949299	5.14E-05	centroid_1801	4.30E-06
centroid_10064	HTH-type transcriptional regulator MatA	19	51	0	56	centroid_1006	15.8421913	6.89E-05	centroid_1006	4.95E-06
centroid_16870	hypothetical protein	19	51	0	56	centroid_1687	15.8421913	6.89E-05	centroid_1687	4.95E-06
centroid_4428	primary amine oxidase	19	51	0	56	centroid_4428	15.8421913	6.89E-05	centroid_4428	4.95E-06
centroid_5377	HTH-type transcriptional regulator MatA	19	51	0	56	centroid_5377	15.8421913	6.89E-05	centroid_5377	4.95E-06
centroid_9966	copper amine oxidase, N2 domain protein	19	51	0	56	centroid_9966	15.8421913	6.89E-05	centroid_9966	4.95E-06
centroid_11967	conserved hypothetical protein	19	52	0	55	centroid_1196	15.3050624	9.15E-05	centroid_1196	6.11E-06
centroid_14977	conserved hypothetical protein	19	52	0	55	centroid_1497	15.3050624	9.15E-05	centroid_1497	6.11E-06
centroid_4663	CDP-alcohol phosphatidyltransferase family protein	19	52	0	55	centroid_4663	15.3050624	9.15E-05	centroid_4663	6.11E-06
centroid_5374	conserved hypothetical protein	19	52	0	55	centroid_5374	15.3050624	9.15E-05	centroid_5374	6.11E-06
centroid_5375	ribosomal protein L31	19	52	0	55	centroid_5375	15.3050624	9.15E-05	centroid_5375	6.11E-06
centroid_5376	ribosomal protein L36	19	52	0	55	centroid_5376	15.3050624	9.15E-05	centroid_5376	6.11E-06
centroid_7339	type VII secretion system (T7SS), usher family protein	19	52	0	55	centroid_7339	15.3050624	9.15E-05	centroid_7339	6.11E-06
centroid_9007	conserved hypothetical protein	19	52	0	55	centroid_9007	15.3050624	9.15E-05	centroid_9007	6.11E-06
centroid_5891	protein DedA	18	1	1	106	centroid_5891	103.661532	2.40E-24	centroid_5891	1.28E-19
centroid_13110	outer membrane autotransporter barrel domain protein	18	2	1	105	centroid_1311	97.3695232	5.75E-23	centroid_1311	1.27E-18
centroid_5572	outer membrane autotransporter barrel domain protein	18	2	1	105	centroid_5572	97.3695232	5.75E-23	centroid_5572	1.27E-18
centroid_13367	type IV leader peptidase family protein	18	7	1	100	centroid_1336	73.4622102	1.03E-17	centroid_1336	3.06E-15
centroid_5955	BFD-like [2Fe-2S] binding domain protein	18	7	1	100	centroid_5955	73.4622102	1.03E-17	centroid_5955	3.06E-15
centroid_5956	bacterioferritin	18	7	1	100	centroid_5956	73.4622102	1.03E-17	centroid_5956	3.06E-15
centroid_13093	CRISPR-associated helicase Cas3	18	8	1	99	centroid_1309	69.7846392	6.61E-17	centroid_1309	9.87E-15
centroid_15847	conserved hypothetical protein	18	8	1	99	centroid_1584	69.7846392	6.61E-17	centroid_1584	9.87E-15
centroid_5675	SPR-associated protein Cas6/Cse3/CasE, subtype I-E/EC	18	8	1	99	centroid_5675	69.7846392	6.61E-17	centroid_5675	9.87E-15
centroid_5676	RISPR-associated protein Cas5/CasD, subtype I-E/ECO	18	8	1	99	centroid_5676	69.7846392	6.61E-17	centroid_5676	9.87E-15
centroid_5678	CRISPR-associated helicase Cas3	18	8	1	99	centroid_5678	69.7846392	6.61E-17	centroid_5678	9.87E-15
centroid_6108	transposase IS116/IS110/IS902 family protein	18	8	1	99	centroid_6108	69.7846392	6.61E-17	centroid_6108	9.87E-15
centroid_7349	SPR-associated protein Cas7/Cse4/CasC, subtype I-E/E	18	8	1	99	centroid_7349	69.7846392	6.61E-17	centroid_7349	9.87E-15
centroid_7675	CRISPR-associated endonuclease Cas1	18	8	1	99	centroid_7675	69.7846392	6.61E-17	centroid_7675	9.87E-15
centroid_8842	SPR-associated protein Cas7/Cse4/CasC, subtype I-E/E	18	8	1	99	centroid_8842	69.7846392	6.61E-17	centroid_8842	9.87E-15
centroid_13094	CRISPR-associated endonuclease Cas3-HD	18	9	1	98	centroid_1309	66.3796449	3.72E-16	centroid_1309	2.93E-14
centroid_14390	CRISPR-associated endonuclease Cas3-HD	18	9	1	98	centroid_1439	66.3796449	3.72E-16	centroid_1439	2.93E-14
centroid_14737	conserved hypothetical protein	18	12	1	95	centroid_1473	57.5275916	3.33E-14	centroid_1473	5.26E-13
centroid_7086	transposase family protein	18	14	1	93	centroid_7086	52.549086	4.20E-13	centroid_7086	2.81E-12
centroid_5759	conserved hypothetical protein	18	31	1	76	centroid_5759	26.6615391	2.42E-07	centroid_5759	5.71E-08
centroid_16189	putative fimbrial subunit YadM domain protein	18	32	1	75	centroid_1618	25.688398	4.01E-07	centroid_1618	8.82E-08
centroid_6222	fimbrial family protein	18	32	1	75	centroid_6222	25.688398	4.01E-07	centroid_6222	8.82E-08
centroid_4607	phenolic acid decarboxylase subunit D	18	33	1	74	centroid_4607	24.7536171	6.51E-07	centroid_4607	1.35E-07
centroid_4608	ubiD decarboxylase family protein	18	33	1	74	centroid_4608	24.7536171	6.51E-07	centroid_4608	1.35E-07
centroid_4609	putative aromatic acid decarboxylase	18	33	1	74	centroid_4609	24.7536171	6.51E-07	centroid_4609	1.35E-07
centroid_4610	marR family protein	18	33	1	74	centroid_4610	24.7536171	6.51E-07	centroid_4610	1.35E-07
centroid_5672	ubiD decarboxylase family protein	18	33	1	74	centroid_5672	24.7536171	6.51E-07	centroid_5672	1.35E-07
centroid_5673	octaprenyl-4-hydroxybenzoate carboxy-lyase family prot	18	33	1	74	centroid_5673	24.7536171	6.51E-07	centroid_5673	1.35E-07
centroid_10540	glycosyl hydrolase 1 family protein	18	40	1	67	centroid_1054	19.1182125	1.23E-05	centroid_1054	1.98E-06
centroid_13126	bacterial regulatory, gntR family protein	18	40	1	67	centroid_1312	19.1182125	1.23E-05	centroid_1312	1.98E-06
centroid_13205	UTRA domain protein	18	40	1	67	centroid_1320	19.1182125	1.23E-05	centroid_1320	1.98E-06
centroid_18361	PTS system, Lactose/Cellobiose specific IIB subunit	18	40	1	67	centroid_1836	19.1182125	1.23E-05	centroid_1836	1.98E-06
centroid_4615	PTS system, Lactose/Cellobiose specific IIB subunit	18	40	1	67	centroid_4615	19.1182125	1.23E-05	centroid_4615	1.98E-06
centroid_4616	TS system, lactose/cellobiose IIC component family prote	18	40	1	67	centroid_4616	19.1182125	1.23E-05	centroid_4616	1.98E-06
centroid_5809	PTS system, Lactose/Cellobiose specific IIB subunit	18	40	1	67	centroid_5809	19.1182125	1.23E-05	centroid_5809	1.98E-06
centroid_7362	glycosyl hydrolase 1 family protein	18	40	1	67	centroid_7362	19.1182125	1.23E-05	centroid_7362	1.98E-06
centroid_6140	outer membrane protein C	18	43	1	64	centroid_6140	17.1022537	3.54E-05	centroid_6140	6.33E-06
centroid_8238	bacterial Ig-like domain family protein	18	43	1	64	centroid_8238	17.1022537	3.54E-05	centroid_8238	6.33E-06
centroid_9351	type IV leader peptidase family protein	18	46	1	61	centroid_9351	15.2775631	9.28E-05	centroid_9351	2.23E-05
centroid_5847	fimbrial family protein	17	2	2	105	centroid_5847	89.9791957	2.41E-21	centroid_5847	6.11E-17
centroid_7211	ankyrin repeat family protein	17	10	2	97	centroid_7211	56.8614229	4.68E-14	centroid_7211	2.60E-12
centroid_7761	putative iS621 protein	17	13	2	94	centroid_7761	49.0026039	2.56E-12	centroid_7761	3.49E-11
centroid_15812	hypothetical protein	17	16	2	91	centroid_1581	42.5764562	6.80E-11	centroid_1581	3.20E-10
centroid_4626	type-1 fimbrial protein, A chain	17	31	2	76	centroid_4626	22.5444185	2.05E-06	centroid_4626	8.38E-07
centroid_15960	conserved hypothetical protein	17	32	2	75	centroid_1596	21.648616	3.27E-06	centroid_1596	1.25E-06
centroid_17338	type VII secretion system (T7SS), usher family protein	17	32	2	75	centroid_1733	21.648616	3.27E-06	centroid_1733	1.25E-06
centroid_4627	chaperone protein FocC	17	33	2	74	centroid_4627	20.7891845	5.13E-06	centroid_4627	1.85E-06
centroid_6110	type IV secretion system Vgr family domain protein	17	33	2	74	centroid_6110	20.7891845	5.13E-06	centroid_6110	1.85E-06
centroid_4305	plasmid stabilisation system family protein	17	38	2	69	centroid_4305	16.9688811	3.80E-05	centroid_4305	1.68E-05
centroid_4306	ribbon-helix-helix , copG family protein	17	38	2	69	centroid_4306	16.9688811	3.80E-05	centroid_4306	1.68E-05
centroid_4614	deoR-like helix-turn-helix domain protein	17	40	2	67	centroid_4614	15.6324805	7.69E-05	centroid_4614	2.49E-05
centroid_11165	hypothetical protein	16	2	3	105	centroid_1116	82.7423758	9.35E-20	centroid_1116	1.97E-15
centroid_5573	conserved hypothetical protein	16	2	3	105	centroid_5573	82.7423758	9.35E-20	centroid_5573	1.97E-15
centroid_5899	L-galactonate transporter	16	2	3	105	centroid_5899	82.7423758	9.35E-20	centroid_5899	1.97E-15
centroid_5581	putative membrane protein	16	3	3	104	centroid_5581	77.2648409	1.50E-18	centroid_5581	1.22E-14
centroid_11324	conserved hypothetical protein	16	24	3	83	centroid_1132	25.6422194	4.11E-07	centroid_1132	4.26E-07
centroid_5714	conserved hypothetical protein	16	24	3	83	centroid_5714	25.6422194	4.11E-07	centroid_5714	4.26E-07
centroid_13521	conjugative transfer relaxase protein TraI	16	26	3	81	centroid_1352	23.4351943	1.29E-06	centroid_1352	1.06E-06
centroid_14951	conserved hypothetical protein	16	28	3	79	centroid_1495	21.4325075	3.67E-06	centroid_1495	2.47E-06
centroid_15014	conserved hypothetical protein	16	28	3	79	centroid_1501	21.4325075	3.67E-06	centroid_1501	2.47E-06
centroid_5564	conserved hypothetical protein	16	28	3	79	centroid_5564	21.4325075	3.67E-06	centroid_5564	2.47E-06
centroid_17931	conserved hypothetical protein	16	29	3	78	centroid_1793	20.4993606	5.97E-06	centroid_1793	3.70E-06
centroid_18482	conserved hypothetical protein	16	29	3	78	centroid_1848	20.4993606	5.97E-06	centroid_1848	3.70E-06
centroid_10927	conserved hypothetical protein	16	31	3	76	centroid_1092	18.7551243	1.49E-05	centroid_1092	8.03E-06
centroid_11168	conserved hypothetical protein	16	31	3	76	centroid_1116	18.7551243	1.49E-05	centroid_1116	8.03E-06
centroid_13619	conserved hypothetical protein	16	31	3	76	centroid_1361	18.7551243	1.49E-05	centroid_1361	8.03E-06
centroid_18481	conserved hypothetical protein	16	31	3	76	centroid_1848	18.7551243	1.49E-05	centroid_1848	8.03E-06
centroid_11134	hypothetical protein	16	32	3	75	centroid_1113	17.9390229	2.28E-05	centroid_1113	1.16E-05
centroid_15015	conserved hypothetical protein	16	32	3	75	centroid_1501	17.9390229	2.28E-05	centroid_1501	1.16E-05
centroid_6101	putative membrane protein	16	32	3	75	centroid_6101	17.9390229	2.28E-05	centroid_6101	1.16E-05

centroid_7846	involved in detoxification of methylglyoxal domain protein	16	33	3	74	centroid_7846	17.1572687	3.44E-05	centroid_7846	1.67E-05
centroid_4387	hicB family protein	16	34	3	73	centroid_4387	16.4078409	5.11E-05	centroid_4387	2.36E-05
centroid_7092	hypothetical protein	16	35	3	72	centroid_7092	15.6888794	7.47E-05	centroid_7092	5.12E-05
centroid_11606	YoaG domain protein	15	1	4	106	centroid_1160	81.6808607	1.60E-19	centroid_1160	5.82E-15
centroid_5875	conserved hypothetical protein	15	1	4	106	centroid_5875	81.6808607	1.60E-19	centroid_5875	5.82E-15
centroid_5876	hicB family protein	15	1	4	106	centroid_5876	81.6808607	1.60E-19	centroid_5876	5.82E-15
centroid_12984	bacterial Ig-like domain family protein	15	2	4	105	centroid_1298	75.6577497	3.37E-18	centroid_1298	4.78E-14
centroid_5623	conserved hypothetical protein	15	2	4	105	centroid_5623	75.6577497	3.37E-18	centroid_5623	4.78E-14
centroid_5900	racemase / muconate lactonizing enzyme, N-terminal dor	15	2	4	105	centroid_5900	75.6577497	3.37E-18	centroid_5900	4.78E-14
centroid_11144	rhs core with extension domain protein	15	3	4	104	centroid_1114	70.3055829	5.08E-17	centroid_1114	2.77E-13
centroid_11150	putative yhaC	15	3	4	104	centroid_1115	70.3055829	5.08E-17	centroid_1115	2.77E-13
centroid_12963	putative dsORF-e4	15	3	4	104	centroid_1296	70.3055829	5.08E-17	centroid_1296	2.77E-13
centroid_12964	conserved hypothetical protein	15	3	4	104	centroid_1296	70.3055829	5.08E-17	centroid_1296	2.77E-13
centroid_17233	lrate uptake ABC transporter 2 (CUT2) family, ATP-bindir	15	3	4	104	centroid_1723	70.3055829	5.08E-17	centroid_1723	2.77E-13
centroid_17234	ABC transporter family protein	15	3	4	104	centroid_1723	70.3055829	5.08E-17	centroid_1723	2.77E-13
centroid_18642	conserved hypothetical protein	15	3	4	104	centroid_1864	70.3055829	5.08E-17	centroid_1864	2.77E-13
centroid_5651	ASCH domain protein	15	3	4	104	centroid_5651	70.3055829	5.08E-17	centroid_5651	2.77E-13
centroid_5652	putative LACI-type transcriptional regulator	15	3	4	104	centroid_5652	70.3055829	5.08E-17	centroid_5652	2.77E-13
centroid_5653	heme ABC exporter, ATP-binding protein CcmA	15	3	4	104	centroid_5653	70.3055829	5.08E-17	centroid_5653	2.77E-13
centroid_5654	ain amino acid transport system / permease component f	15	3	4	104	centroid_5654	70.3055829	5.08E-17	centroid_5654	2.77E-13
centroid_5655	ain amino acid transport system / permease component f	15	3	4	104	centroid_5655	70.3055829	5.08E-17	centroid_5655	2.77E-13
centroid_5813	putative membrane protein	15	3	4	104	centroid_5813	70.3055829	5.08E-17	centroid_5813	2.77E-13
centroid_5873	conserved hypothetical protein	15	3	4	104	centroid_5873	70.3055829	5.08E-17	centroid_5873	2.77E-13
centroid_6089	putative rHS protein	15	3	4	104	centroid_6089	70.3055829	5.08E-17	centroid_6089	2.77E-13
centroid_6090	conserved hypothetical protein	15	3	4	104	centroid_6090	70.3055829	5.08E-17	centroid_6090	2.77E-13
centroid_12982	sel1 repeat family protein	15	4	4	103	centroid_1298	65.5184672	5.76E-16	centroid_1298	1.27E-12
centroid_5660	sel1 repeat family protein	15	4	4	103	centroid_5660	65.5184672	5.76E-16	centroid_5660	1.27E-12
centroid_5661	acetyltransferase domain protein	15	4	4	103	centroid_5661	65.5184672	5.76E-16	centroid_5661	1.27E-12
centroid_5662	conserved hypothetical protein	15	4	4	103	centroid_5662	65.5184672	5.76E-16	centroid_5662	1.27E-12
centroid_5819	conserved hypothetical protein	15	4	4	103	centroid_5819	65.5184672	5.76E-16	centroid_5819	1.27E-12
centroid_5820	helix-turn-helix family protein	15	4	4	103	centroid_5820	65.5184672	5.76E-16	centroid_5820	1.27E-12
centroid_5821	putative transcription elongation factor GreB	15	4	4	103	centroid_5821	65.5184672	5.76E-16	centroid_5821	1.27E-12
centroid_5822	alpha/beta hydrolase fold family protein	15	4	4	103	centroid_5822	65.5184672	5.76E-16	centroid_5822	1.27E-12
centroid_7345	aldo/keto reductase family protein	15	4	4	103	centroid_7345	65.5184672	5.76E-16	centroid_7345	1.27E-12
centroid_9422	putative membrane protein	15	4	4	103	centroid_9422	65.5184672	5.76E-16	centroid_9422	1.27E-12
centroid_8902	conserved hypothetical protein	15	5	4	102	centroid_8902	61.2116897	5.13E-15	centroid_8902	4.90E-12
centroid_5605	putative membrane protein	15	7	4	100	centroid_5605	53.7773172	2.25E-13	centroid_5605	5.02E-11
centroid_5814	putative membrane protein	15	7	4	100	centroid_5814	53.7773172	2.25E-13	centroid_5814	5.02E-11
centroid_12532	conserved hypothetical family protein	15	9	4	98	centroid_1253	47.5879134	5.26E-12	centroid_1253	3.58E-10
centroid_18577	conserved hypothetical protein	15	9	4	98	centroid_1857	47.5879134	5.26E-12	centroid_1857	3.58E-10
centroid_8547	conserved hypothetical protein	15	9	4	98	centroid_8547	47.5879134	5.26E-12	centroid_8547	3.58E-10
centroid_5911	helix-turn-helix domain protein	15	12	4	95	centroid_5911	40.033627	2.50E-10	centroid_5911	4.25E-09
centroid_8815	putative permease family protein	15	12	4	95	centroid_8815	40.033627	2.50E-10	centroid_8815	4.25E-09
centroid_15857	aldehyde dehydrogenase family protein	15	15	4	92	centroid_1585	34.0025301	5.50E-09	centroid_1585	3.38E-08
centroid_6419	homoprotocatechuate degradation operon regulator, HpaR	15	15	4	92	centroid_6419	34.0025301	5.50E-09	centroid_6419	3.38E-08
centroid_11757	rhs core with extension domain protein	15	23	4	84	centroid_1175	22.6303595	1.96E-06	centroid_1175	2.43E-06
centroid_5861	glycosyl hydrolases 15 family protein	15	26	4	81	centroid_5861	19.5322804	9.89E-06	centroid_5861	8.75E-06
centroid_9569	glycosyl hydrolases 15 family protein	15	27	4	80	centroid_9569	18.6009592	1.61E-05	centroid_9569	1.30E-05
centroid_4447	putative dNA-damage-inducible protein D	15	107	4	0	centroid_4447	16.9199499	3.90E-05	centroid_4447	0.00038725
centroid_5882	conserved hypothetical protein	15	107	4	0	centroid_5882	16.9199499	3.90E-05	centroid_5882	0.00038725
centroid_7639	putative dNA-damage-inducible protein D	15	107	4	0	centroid_7639	16.9199499	3.90E-05	centroid_7639	0.00038725
centroid_4628	type VII secretion system (T7SS), usher family protein	15	30	4	77	centroid_4628	16.0645352	6.12E-05	centroid_4628	3.98E-05
centroid_18643	RHS repeat-associated core domain protein	14	1	5	106	centroid_1864	74.6354981	5.66E-18	centroid_1864	1.21E-13
centroid_5853	n-negative pili assembly chaperone, N-terminal domain pr	14	1	5	106	centroid_5853	74.6354981	5.66E-18	centroid_5853	1.21E-13
centroid_11109	n-negative pili assembly chaperone, C-terminal domain pr	14	2	5	105	centroid_1110	68.7247725	1.13E-16	centroid_1110	9.30E-13
centroid_5849	conserved hypothetical protein	14	2	5	105	centroid_5849	68.7247725	1.13E-16	centroid_5849	9.30E-13
centroid_5850	fimbrial family protein	14	2	5	105	centroid_5850	68.7247725	1.13E-16	centroid_5850	9.30E-13
centroid_5851	fimbrial family protein	14	2	5	105	centroid_5851	68.7247725	1.13E-16	centroid_5851	9.30E-13
centroid_5852	fimbrial family protein	14	2	5	105	centroid_5852	68.7247725	1.13E-16	centroid_5852	9.30E-13
centroid_14626	conserved hypothetical protein	14	7	5	100	centroid_1462	47.6482046	5.10E-12	centroid_1462	7.23E-10
centroid_7350	CRISPR type I-E/ECOLI-associated protein CasB/Cse2	14	7	5	100	centroid_7350	47.6482046	5.10E-12	centroid_7350	7.23E-10
centroid_6427	-hydroxyphenylacetate catabolism regulatory protein Hpa	14	13	5	94	centroid_6427	32.7240531	1.06E-08	centroid_6427	9.45E-08
centroid_13516	putative tail length tape measure domain protein	14	14	5	93	centroid_1351	30.8658387	2.76E-08	centroid_1351	1.80E-07
centroid_12523	ydroxyphenylacetate 3-hydroxylase C terminal family pro	14	15	5	92	centroid_1252	29.1379581	6.74E-08	centroid_1252	3.32E-07
centroid_12524	ydroxyphenylacetate 3-hydroxylase N terminal family pro	14	15	5	92	centroid_1252	29.1379581	6.74E-08	centroid_1252	3.32E-07
centroid_6420	otocatechuate catabolism bifunctional isomerase/decarbo	14	15	5	92	centroid_6420	29.1379581	6.74E-08	centroid_6420	3.32E-07
centroid_6421	boxymethyl-2-hydroxymuconate semialdehyde dehydrog	14	15	5	92	centroid_6421	29.1379581	6.74E-08	centroid_6421	3.32E-07
centroid_6422	3,4-dihydroxyphenylacetate 2,3-dioxygenase	14	15	5	92	centroid_6422	29.1379581	6.74E-08	centroid_6422	3.32E-07
centroid_6424	2-oxo-hepta-3-ene-1,7-dioic acid hydratase	14	15	5	92	centroid_6424	29.1379581	6.74E-08	centroid_6424	3.32E-07
centroid_6425	2,4-dihydroxyhept-2-ene-1,7-dioic acid aldolase	14	15	5	92	centroid_6425	29.1379581	6.74E-08	centroid_6425	3.32E-07
centroid_6426	4-hydroxyphenylacetate permease	14	15	5	92	centroid_6426	29.1379581	6.74E-08	centroid_6426	3.32E-07
centroid_6428	lroxyphenylacetate 3-monooxygenase, oxygenase comp	14	15	5	92	centroid_6428	29.1379581	6.74E-08	centroid_6428	3.32E-07
centroid_6429	droxyphenylacetate 3-monooxygenase, reductase compo	14	15	5	92	centroid_6429	29.1379581	6.74E-08	centroid_6429	3.32E-07
centroid_11086	o-L-gulonate-6-phosphate decarboxylase UlaD domain p	14	107	5	0	centroid_1108	22.822425	1.78E-06	centroid_1108	4.76E-05
centroid_2634	putative 4-phosphopantetheinyl transferase EntD	14	107	5	0	centroid_2634	22.822425	1.78E-06	centroid_2634	4.76E-05
centroid_14352	putative predicted protein	14	20	5	87	centroid_1435	22.053186	2.65E-06	centroid_1435	4.65E-06
centroid_6980	conserved hypothetical protein	14	25	5	82	centroid_6980	16.8341725	4.08E-05	centroid_6980	3.88E-05
centroid_10025	conserved hypothetical protein	14	26	5	81	centroid_1002	15.9534263	6.49E-05	centroid_1002	5.66E-05
centroid_16717	hypothetical protein	14	26	5	81	centroid_1671	15.9534263	6.49E-05	centroid_1671	5.66E-05
centroid_16718	conserved hypothetical protein	14	26	5	81	centroid_1671	15.9534263	6.49E-05	centroid_1671	5.66E-05
centroid_8551	conserved hypothetical protein	14	26	5	81	centroid_8551	15.9534263	6.49E-05	centroid_8551	5.66E-05
centroid_13498	conserved hypothetical protein	13	3	6	104	centroid_1349	56.8868125	4.62E-14	centroid_1349	7.64E-11
centroid_15497	outer membrane autotransporter barrel domain protein	13	3	6	104	centroid_1549	56.8868125	4.62E-14	centroid_1549	7.64E-11
centroid_15498	nded Signal Peptide of Type V secretion system family pr	13	3	6	104	centroid_1549	56.8868125	4.62E-14	centroid_1549	7.64E-11
centroid_5647	outer membrane autotransporter barrel domain protein	13	3	6	104	centroid_5647	56.8868125	4.62E-14	centroid_5647	7.64E-11
centroid_5677	CRISPR type I-E/ECOLI-associated protein CasA/Cse1	13	7	6	100	centroid_5677	41.747754	1.04E-10	centroid_5677	8.57E-09
centroid_8843	CT1975-like family protein	13	7	6	100	centroid_8843	41.747754	1.04E-10	centroid_8843	8.57E-09
centroid_12632	mu-like prophage major head subunit gpT family protein	13	13	6	94	centroid_1263	27.8555159	1.31E-07	centroid_1263	8.27E-07
centroid_18615	putative transposase	13	13	6	94	centroid_1861	27.8555159	1.31E-07	centroid_1861	8.27E-07
centroid_5804	phage tail tape measure protein, lambda family	13	15	6	92	centroid_5804	24.5707083	7.16E-07	centroid_5804	2.66E-06
centroid_7317	conserved hypothetical protein	13	15	6	92	centroid_7317	24.5707083	7.16E-07	centroid_7317	2.66E-06
centroid_6124	yhaC domain protein	13	106	6	1	centroid_6124	23.3326582	1.36E-06	centroid_6124	3.50E-05
centroid_11244	conserved hypothetical family protein	13	17	6	90	centroid_1124	21.7356585	3.13E-06	centroid_1124	7.58E-06
centroid_5803	phage tail assembly protein T	13	18	6	89	centroid_5803	20.4598148	6.09E-06	centroid_5803	1.23E-05
centroid_7253	phage minor tail protein G	13	18	6	89	centroid_7253	20.4598148	6.09E-06	centroid_7253	1.23E-05
centroid_13112	bacterial Ig-like domain family protein	13	19	6	88	centroid_1311	19.266742	1.14E-05	centroid_1311	1.95E-05
centroid_13448	hypothetical protein	13	19	6	88	centroid_1344	19.266742	1.14E-05	centroid_1344	1.95E-05
centroid_5805	phage minor tail family protein	13	19	6	88	centroid_5805	19.266742	1.14E-05	centroid_5805	1.95E-05
centroid_6608	bacterial Ig-like domain family protein	13	19	6	88	centroid_6608	19.266742	1.14E-05	centroid_6608	1.95E-05
centroid_12447	bacterial Ig-like domain family protein	13	20	6	87	centroid_1244	18.1490105	2.04E-05	centroid_1244	3.04E-05
centroid_17335	bacterial Ig-like domain family protein	13	20	6	87	centroid_1733	18.1490105	2.04E-05	centroid_1733	3.04E-05
centroid_5632	bacterial Ig-like domain family protein	13	20	6	87	centroid_5632	18.1490105	2.04E-05	centroid_5632	3.04E-05
centroid_16839	bacterial Ig-like domain family protein	13	21	6	86	centroid_1683	17.1000685	3.55E-05	centroid_1683	4.63E-05
centroid_10954	intimin C-type lectin domain protein	13	22	6	85	centroid_1095	16.1141171	5.96E-05	centroid_1095	6.93E-05
centroid_5633	intimin C-type lectin domain protein	13	22	6	85	centroid_5633	16.1141171	5.96E-05	centroid_5633	6.93E-05
centroid_8549	intimin C-type lectin domain protein	13	22	6	85	centroid_8549	16.1141171	5.96E-05	centroid_8549	6.93E-05
centroid_10088	conserved hypothetical protein	12	0	7	107	centroid_1008	67.5426968	2.06E-16	centroid_1008	2.59E-12

centroid_12889	bacterial regulatory , arsR family protein	12	0	7	107	centroid_1288	67.5426968	2.06E-16	centroid_1288	2.59E-12
centroid_5604	pfkB carbohydrate kinase family protein	12	0	7	107	centroid_5604	67.5426968	2.06E-16	centroid_5604	2.59E-12
centroid_13466	RHS repeat-associated core domain protein	12	1	7	106	centroid_1346	60.9565398	5.83E-15	centroid_1346	3.17E-11
centroid_961	putative pseudouridine transporter	12	107	7	0	centroid_961	35.0135702	3.27E-09	centroid_961	5.97E-07
centroid_9176	putative yfdA protein	12	8	7	99	centroid_9176	33.4081722	7.47E-09	centroid_9176	2.02E-07
centroid_10033	conserved hypothetical protein	12	9	7	98	centroid_1003	30.9886867	2.60E-08	centroid_1003	4.42E-07
centroid_13111	conserved hypothetical protein	12	9	7	98	centroid_1311	30.9886867	2.60E-08	centroid_1311	4.42E-07
centroid_6792	phage tail tape measure protein, lambda family	12	12	7	95	centroid_6792	24.9643493	5.84E-07	centroid_6792	3.36E-06
centroid_10961	trbC domain protein	12	13	7	94	centroid_1096	23.2857214	1.40E-06	centroid_1096	6.06E-06
centroid_15492	putative predicted protein	12	14	7	93	centroid_1549	21.7403515	3.12E-06	centroid_1549	1.06E-05
centroid_12838	integrase core domain protein	12	104	7	3	centroid_1283	21.138612	4.27E-06	centroid_1283	5.42E-05
centroid_4773	conserved hypothetical protein	12	102	7	5	centroid_4773	15.8242097	6.95E-05	centroid_4773	0.00029613
centroid_12888	pfkB carbohydrate kinase family protein	11	0	8	107	centroid_1288	60.8013221	6.31E-15	centroid_1288	3.72E-11
centroid_16123	conserved hypothetical protein	11	2	8	105	centroid_1612	48.846777	2.77E-12	centroid_1612	2.54E-09
centroid_5816	DKNYY family protein	11	2	8	105	centroid_5816	48.846777	2.77E-12	centroid_5816	2.54E-09
centroid_7386	conserved hypothetical protein	11	2	8	105	centroid_7386	48.846777	2.77E-12	centroid_7386	2.54E-09
centroid_7527	conserved hypothetical protein	11	2	8	105	centroid_7527	48.846777	2.77E-12	centroid_7527	2.54E-09
centroid_3606	putative mRNA interferase HicA	11	107	8	0	centroid_3606	41.2864505	1.31E-10	centroid_3606	6.02E-08
centroid_10046	antitoxin HigA	11	4	8	103	centroid_1004	40.1063958	2.41E-10	centroid_1004	3.89E-08
centroid_15002	DKNYY family protein	11	4	8	103	centroid_1500	40.1063958	2.41E-10	centroid_1500	3.89E-08
centroid_17830	helix-turn-helix domain protein	11	4	8	103	centroid_1783	40.1063958	2.41E-10	centroid_1783	3.89E-08
centroid_17831	conserved hypothetical protein	11	4	8	103	centroid_1783	40.1063958	2.41E-10	centroid_1783	3.89E-08
centroid_5815	conserved hypothetical protein	11	4	8	103	centroid_5815	40.1063958	2.41E-10	centroid_5815	3.89E-08
centroid_7196	acetyltransferase family protein	11	4	8	103	centroid_7196	40.1063958	2.41E-10	centroid_7196	3.89E-08
centroid_7197	conserved hypothetical protein	11	4	8	103	centroid_7197	40.1063958	2.41E-10	centroid_7197	3.89E-08
centroid_7347	antitoxin HigA	11	4	8	103	centroid_7347	40.1063958	2.41E-10	centroid_7347	3.89E-08
centroid_10235	fibronectin type III family protein	11	5	8	102	centroid_1023	36.5652769	1.48E-09	centroid_1023	1.16E-07
centroid_13480	fibronectin type III family protein	11	5	8	102	centroid_1348	36.5652769	1.48E-09	centroid_1348	1.16E-07
centroid_7818	conserved hypothetical protein	11	105	8	2	centroid_7818	30.4557347	3.42E-08	centroid_7818	2.28E-06
centroid_5800	prophage minor tail Z family protein	11	9	8	98	centroid_5800	25.9968478	3.42E-07	centroid_5800	3.39E-06
centroid_13292	conserved hypothetical protein	11	10	8	97	centroid_1329	23.997639	9.65E-07	centroid_1329	6.63E-06
centroid_5796	phage portal protein, lambda family	11	12	8	95	centroid_5796	20.5369313	5.85E-06	centroid_5796	2.20E-05
centroid_5798	conserved hypothetical protein	11	13	8	94	centroid_5798	19.0309272	1.29E-05	centroid_5798	3.78E-05
centroid_5799	TP-binding sugar transporter from pro-phage family prote	11	13	8	94	centroid_5799	19.0309272	1.29E-05	centroid_5799	3.78E-05
centroid_11474	penicillin amidase family protein	10	0	9	107	centroid_1147	54.1793909	1.83E-13	centroid_1147	4.79E-10
centroid_13225	RHS repeat-associated core domain protein	10	0	9	107	centroid_1322	54.1793909	1.83E-13	centroid_1322	4.79E-10
centroid_15860	penicillin amidase family protein	10	0	9	107	centroid_1586	54.1793909	1.83E-13	centroid_1586	4.79E-10
centroid_5682	repair family protein	10	0	9	107	centroid_5682	54.1793909	1.83E-13	centroid_5682	4.79E-10
centroid_5683	hypothetical protein	10	0	9	107	centroid_5683	54.1793909	1.83E-13	centroid_5683	4.79E-10
centroid_9988	penicillin amidase family protein	10	0	9	107	centroid_9988	54.1793909	1.83E-13	centroid_9988	4.79E-10
centroid_11139	putative rhs core protein with extension	10	1	9	106	centroid_1113	47.8251708	4.66E-12	centroid_1113	4.90E-09
centroid_17597	RHS Repeat family protein	10	1	9	106	centroid_1759	47.8251708	4.66E-12	centroid_1759	4.90E-09
centroid_6106	integrase core domain protein	10	1	9	106	centroid_6106	47.8251708	4.66E-12	centroid_6106	4.90E-09
centroid_7531	RHS repeat-associated core domain protein	10	1	9	106	centroid_7531	47.8251708	4.66E-12	centroid_7531	4.90E-09
centroid_11137	putative rhs core protein	10	2	9	105	centroid_1113	42.5397196	6.93E-11	centroid_1113	2.73E-08
centroid_11138	conserved hypothetical protein	10	2	9	105	centroid_1113	42.5397196	6.93E-11	centroid_1113	2.73E-08
centroid_13461	RHS repeat-associated core domain protein	10	2	9	105	centroid_1346	42.5397196	6.93E-11	centroid_1346	2.73E-08
centroid_18514	putative transcriptional activator Ogr/delta	10	3	9	104	centroid_1851	38.0766368	6.80E-10	centroid_1851	1.10E-07
centroid_5681	lemA family protein	10	3	9	104	centroid_5681	38.0766368	6.80E-10	centroid_5681	1.10E-07
centroid_18198	phage tail tape measure protein, lambda family	10	4	9	103	centroid_1819	34.2599299	4.82E-09	centroid_1819	3.57E-07
centroid_18234	lemA family protein	10	4	9	103	centroid_1823	34.2599299	4.82E-09	centroid_1823	3.57E-07
centroid_15977	transposase, IS605 OrfB family	10	103	9	4	centroid_1597	28.6461192	8.69E-08	centroid_1597	2.90E-06
centroid_5659	conserved hypothetical protein	10	6	9	101	centroid_5659	28.0817014	1.16E-07	centroid_5659	2.45E-06
centroid_7871	integrase core domain protein	10	6	9	101	centroid_7871	28.0817014	1.16E-07	centroid_7871	2.45E-06
centroid_8976	conserved hypothetical protein	10	6	9	101	centroid_8976	28.0817014	1.16E-07	centroid_8976	2.45E-06
centroid_12688	phage tail tape measure protein, lambda family	10	7	9	100	centroid_1268	25.5493945	4.31E-07	centroid_1268	5.52E-06
centroid_12380	integrase core domain protein	10	8	9	99	centroid_1238	23.3060748	1.38E-06	centroid_1238	1.15E-05
centroid_5797	clp protease family protein	10	10	9	97	centroid_5797	19.5137806	9.99E-06	centroid_5797	4.15E-05
centroid_7396	outer membrane porin protein OmpD	10	10	9	97	centroid_7396	19.5137806	9.99E-06	centroid_7396	4.15E-05
centroid_9788	conserved hypothetical protein	10	10	9	97	centroid_9788	19.5137806	9.99E-06	centroid_9788	4.15E-05
centroid_5520	integrase core domain protein	10	99	9	8	centroid_5520	18.7137552	1.52E-05	centroid_5520	7.07E-05
centroid_15925	reverse transcriptase family protein	10	98	9	9	centroid_1592	16.9430644	3.85E-05	centroid_1592	0.00012992
centroid_4181	reverse transcriptase family protein	10	98	9	9	centroid_4181	16.9430644	3.85E-05	centroid_4181	0.00012992
centroid_7880	reverse transcriptase family protein	10	98	9	9	centroid_7880	16.9430644	3.85E-05	centroid_7880	0.00012992
centroid_7881	reverse transcriptase family protein	10	98	9	9	centroid_7881	16.9430644	3.85E-05	centroid_7881	0.00012992
centroid_7943	group II intron, maturase-specific domain protein	10	98	9	9	centroid_7943	16.9430644	3.85E-05	centroid_7943	0.00012992
centroid_8104	reverse transcriptase family protein	10	98	9	9	centroid_8104	16.9430644	3.85E-05	centroid_8104	0.00012992
centroid_8276	reverse transcriptase family protein	10	98	9	9	centroid_8276	16.9430644	3.85E-05	centroid_8276	0.00012992
centroid_10180	integrase core domain protein	10	97	9	10	centroid_1018	15.3678278	8.85E-05	centroid_1018	0.00022662
centroid_14811	integrase core domain protein	10	97	9	10	centroid_1481	15.3678278	8.85E-05	centroid_1481	0.00022662
centroid_16992	phage antitermination Q family protein	10	97	9	10	centroid_1699	15.3678278	8.85E-05	centroid_1699	0.00022662
centroid_2828	FAD dependent oxidoreductase family protein	9	107	10	0	centroid_2828	54.1793909	1.83E-13	centroid_2828	4.79E-10
centroid_2829	putative 4Fe-4S binding protein	9	107	10	0	centroid_2829	54.1793909	1.83E-13	centroid_2829	4.79E-10
centroid_2830	conserved hypothetical protein	9	107	10	0	centroid_2830	54.1793909	1.83E-13	centroid_2830	4.79E-10
centroid_4505	carbohydrate kinase, FGGY family	9	107	10	0	centroid_4505	54.1793909	1.83E-13	centroid_4505	4.79E-10
centroid_470	conserved hypothetical protein	9	106	10	1	centroid_470	47.8251708	4.66E-12	centroid_470	4.90E-09
centroid_5658	outer membrane autotransporter barrel domain protein	9	0	10	107	centroid_5658	47.6749026	5.03E-12	centroid_5658	5.61E-09
centroid_7546	RHS repeat-associated core domain protein	9	1	10	106	centroid_7546	41.4693276	1.20E-10	centroid_7546	5.18E-08
centroid_17674	conserved hypothetical protein	9	103	10	4	centroid_1767	34.2599299	4.82E-09	centroid_1767	3.57E-07
centroid_2839	conserved hypothetical protein	9	103	10	4	centroid_2839	34.2599299	4.82E-09	centroid_2839	3.57E-07
centroid_2840	repair family protein	9	103	10	4	centroid_2840	34.2599299	4.82E-09	centroid_2840	3.57E-07
centroid_4506	conserved hypothetical protein	9	103	10	4	centroid_4506	34.2599299	4.82E-09	centroid_4506	3.57E-07
centroid_11094	hypothetical protein	9	3	10	104	centroid_1109	32.1960222	1.39E-08	centroid_1109	9.69E-07
centroid_11095	putative phage immunity repressor protein	9	3	10	104	centroid_1109	32.1960222	1.39E-08	centroid_1109	9.69E-07
centroid_13473	hypothetical protein	9	3	10	104	centroid_1347	32.1960222	1.39E-08	centroid_1347	9.69E-07
centroid_6491	ogr/Delta-like zinc finger family protein	9	3	10	104	centroid_6491	32.1960222	1.39E-08	centroid_6491	9.69E-07
centroid_6492	putative glyco3, capsid size determination protein Sid	9	3	10	104	centroid_6492	32.1960222	1.39E-08	centroid_6492	9.69E-07
centroid_6495	putative derepression protein	9	3	10	104	centroid_6495	32.1960222	1.39E-08	centroid_6495	9.69E-07
centroid_6496	putative predicted protein	9	3	10	104	centroid_6496	32.1960222	1.39E-08	centroid_6496	9.69E-07
centroid_6497	conserved hypothetical protein	9	3	10	104	centroid_6497	32.1960222	1.39E-08	centroid_6497	9.69E-07
centroid_6498	putative P4-specific DNA primase	9	3	10	104	centroid_6498	32.1960222	1.39E-08	centroid_6498	9.69E-07
centroid_17686	putative domain protein	9	102	10	5	centroid_1768	30.9605456	2.63E-08	centroid_1768	9.93E-07
centroid_10137	caudovirales tail fibre assembly family protein	9	101	10	6	centroid_1013	28.0817014	1.16E-07	centroid_1013	2.45E-06
centroid_18607	hypothetical protein	9	5	10	102	centroid_1860	25.6140863	4.17E-07	centroid_1860	7.48E-06
centroid_5921	traG-like , N-terminal region family protein	9	5	10	102	centroid_5921	25.6140863	4.17E-07	centroid_5921	7.48E-06
centroid_7413	conserved hypothetical protein	9	5	10	102	centroid_7413	25.6140863	4.17E-07	centroid_7413	7.48E-06
centroid_7421	hypothetical protein	9	5	10	102	centroid_7421	25.6140863	4.17E-07	centroid_7421	7.48E-06
centroid_10919	type IV/VI secretion system , DotU family domain protein	9	6	10	101	centroid_1091	22.9966206	1.62E-06	centroid_1091	1.72E-05
centroid_12120	conserved hypothetical protein	9	6	10	101	centroid_1212	22.9966206	1.62E-06	centroid_1212	1.72E-05
centroid_12121	conserved hypothetical protein	9	6	10	101	centroid_1212	22.9966206	1.62E-06	centroid_1212	1.72E-05
centroid_16551	conserved hypothetical protein	9	6	10	101	centroid_1655	22.9966206	1.62E-06	centroid_1655	1.72E-05
centroid_16552	imcF-related N-terminal domain protein	9	6	10	101	centroid_1655	22.9966206	1.62E-06	centroid_1655	1.72E-05
centroid_7244	acyl transferase domain protein	9	6	10	101	centroid_7244	22.9966206	1.62E-06	centroid_7244	1.72E-05
centroid_7411	conserved hypothetical protein	9	6	10	101	centroid_7411	22.9966206	1.62E-06	centroid_7411	1.72E-05
centroid_7412	conserved hypothetical protein	9	6	10	101	centroid_7412	22.9966206	1.62E-06	centroid_7412	1.72E-05
centroid_7414	gene 25-like lysozyme family protein	9	6	10	101	centroid_7414	22.9966206	1.62E-06	centroid_7414	1.72E-05
centroid_7415	conserved hypothetical protein	9	6	10	101	centroid_7415	22.9966206	1.62E-06	centroid_7415	1.72E-05

centroid_7416	conserved hypothetical protein	9	6	10	101	centroid_7416	22.9966206	1.62E-06	centroid_7416	1.72E-05
centroid_7417	conserved hypothetical protein	9	6	10	101	centroid_7417	22.9966206	1.62E-06	centroid_7417	1.72E-05
centroid_7418	PAAR motif family protein	9	6	10	101	centroid_7418	22.9966206	1.62E-06	centroid_7418	1.72E-05
centroid_7419	impA-related N-terminal family protein	9	6	10	101	centroid_7419	22.9966206	1.62E-06	centroid_7419	1.72E-05
centroid_7420	type VI secretion lipofamily protein	9	6	10	101	centroid_7420	22.9966206	1.62E-06	centroid_7420	1.72E-05
centroid_7422	type VI secretion ATPase, ClpV1 family	9	6	10	101	centroid_7422	22.9966206	1.62E-06	centroid_7422	1.72E-05
centroid_9144	type IV/VI secretion system , DotU family domain protein	9	6	10	101	centroid_9144	22.9966206	1.62E-06	centroid_9144	1.72E-05
centroid_14508	RHS repeat-associated core domain protein	9	9	10	98	centroid_1450	16.9430644	3.85E-05	centroid_1450	0.00012992
centroid_18330	RHS repeat-associated core domain protein	9	9	10	98	centroid_1833	16.9430644	3.85E-05	centroid_1833	0.00012992
centroid_7523	plasmid segregation protein ParM	9	9	10	98	centroid_7523	16.9430644	3.85E-05	centroid_7523	0.00012992
centroid_7524	plasmid stability family protein	9	9	10	98	centroid_7524	16.9430644	3.85E-05	centroid_7524	0.00012992
centroid_6025	outer membrane lipoprotein blc	9	10	10	97	centroid_6025	15.3678278	8.85E-05	centroid_6025	0.00022662
centroid_4319	alcohol dehydrogenase GroES-like domain protein	8	105	11	2	centroid_4319	48.846777	2.77E-12	centroid_4319	2.54E-09
centroid_13101	penicillin G acylase	8	0	11	107	centroid_1310	41.2864505	1.31E-10	centroid_1310	6.02E-08
centroid_16129	e kinase-, DNA gyrase B-, and HSP90-like ATPase family	8	0	11	107	centroid_1612	41.2864505	1.31E-10	centroid_1612	6.02E-08
centroid_5933	conserved hypothetical protein	8	0	11	107	centroid_5933	41.2864505	1.31E-10	centroid_5933	6.02E-08
centroid_15706	hypothetical protein	8	102	11	5	centroid_1570	36.5652769	1.48E-09	centroid_1570	1.16E-07
centroid_3381	alpha amylase, catalytic domain protein	8	102	11	5	centroid_3381	36.5652769	1.48E-09	centroid_3381	1.16E-07
centroid_11012	conserved hypothetical protein	8	1	11	106	centroid_1101	35.2603579	2.88E-09	centroid_1101	4.97E-07
centroid_11013	outer membrane insertion C-terminal signal domain protei	8	1	11	106	centroid_1101	35.2603579	2.88E-09	centroid_1101	4.97E-07
centroid_11014	esterase-like activity of phytase family protein	8	1	11	106	centroid_1101	35.2603579	2.88E-09	centroid_1101	4.97E-07
centroid_11015	pfkB carbohydrate kinase family protein	8	1	11	106	centroid_1101	35.2603579	2.88E-09	centroid_1101	4.97E-07
centroid_11016	SIS domain protein	8	1	11	106	centroid_1101	35.2603579	2.88E-09	centroid_1101	4.97E-07
centroid_11017	ADP-ribosylglycohydrolase family protein	8	1	11	106	centroid_1101	35.2603579	2.88E-09	centroid_1101	4.97E-07
centroid_11018	bacterial regulatory, gntR family protein	8	1	11	106	centroid_1101	35.2603579	2.88E-09	centroid_1101	4.97E-07
centroid_11019	MFS/sugar transport family protein	8	1	11	106	centroid_1101	35.2603579	2.88E-09	centroid_1101	4.97E-07
centroid_11020	MFS/sugar transport family protein	8	1	11	106	centroid_1102	35.2603579	2.88E-09	centroid_1102	4.97E-07
centroid_13089	esterase-like activity of phytase family protein	8	1	11	106	centroid_1308	35.2603579	2.88E-09	centroid_1308	4.97E-07
centroid_13090	(Glycoside-Pentoside-Hexuronide) transporter domain p	8	1	11	106	centroid_1309	35.2603579	2.88E-09	centroid_1309	4.97E-07
centroid_13254	ATP-binding region ATPase -containing domain protein	8	1	11	106	centroid_1325	35.2603579	2.88E-09	centroid_1325	4.97E-07
centroid_13277	putative 50S ribosomal protein L1	8	1	11	106	centroid_1327	35.2603579	2.88E-09	centroid_1327	4.97E-07
centroid_13278	esterase-like activity of phytase family protein	8	1	11	106	centroid_1327	35.2603579	2.88E-09	centroid_1327	4.97E-07
centroid_15935	ATP-binding region ATPase -containing domain protein	8	1	11	106	centroid_1593	35.2603579	2.88E-09	centroid_1593	4.97E-07
centroid_16094	phage integrase family protein	8	1	11	106	centroid_1609	35.2603579	2.88E-09	centroid_1609	4.97E-07
centroid_16130	phage integrase family protein	8	1	11	106	centroid_1613	35.2603579	2.88E-09	centroid_1613	4.97E-07
centroid_5869	ROS/MUCR transcriptional regulator family protein	8	1	11	106	centroid_5869	35.2603579	2.88E-09	centroid_5869	4.97E-07
centroid_5932	putative membrane protein	8	1	11	106	centroid_5932	35.2603579	2.88E-09	centroid_5932	4.97E-07
centroid_5974	conserved hypothetical protein	8	1	11	106	centroid_5974	35.2603579	2.88E-09	centroid_5974	4.97E-07
centroid_5975	conserved hypothetical protein	8	1	11	106	centroid_5975	35.2603579	2.88E-09	centroid_5975	4.97E-07
centroid_6493	prophage CP4-57 regulatory family protein	8	1	11	106	centroid_6493	35.2603579	2.88E-09	centroid_6493	4.97E-07
centroid_6818	fibronectin type III family protein	8	1	11	106	centroid_6818	35.2603579	2.88E-09	centroid_6818	4.97E-07
centroid_7389	impA domain family protein	8	1	11	106	centroid_7389	35.2603579	2.88E-09	centroid_7389	4.97E-07
centroid_5887	conserved hypothetical protein	8	2	11	105	centroid_5887	30.4557347	3.42E-08	centroid_5887	2.28E-06
centroid_5888	plasmid stability family protein	8	2	11	105	centroid_5888	30.4557347	3.42E-08	centroid_5888	2.28E-06
centroid_11002	putative transposase	8	3	11	104	centroid_1100	26.5398381	2.58E-07	centroid_1100	7.65E-06
centroid_11785	putative transposase	8	3	11	104	centroid_1178	26.5398381	2.58E-07	centroid_1178	7.65E-06
centroid_18222	n-negative pili assembly chaperone, C-terminal domain pr	8	3	11	104	centroid_1822	26.5398381	2.58E-07	centroid_1822	7.65E-06
centroid_6082	putative transposase	8	3	11	104	centroid_6082	26.5398381	2.58E-07	centroid_6082	7.65E-06
centroid_12122	hemolysin expression-modulating protein Hha	8	5	11	102	centroid_1212	20.5552241	5.79E-06	centroid_1212	5.00E-05
centroid_12265	RHS repeat-associated core domain protein	8	5	11	102	centroid_1226	20.5552241	5.79E-06	centroid_1226	5.00E-05
centroid_7429	avrPphF-ORF-2 family protein	8	5	11	102	centroid_7429	20.5552241	5.79E-06	centroid_7429	5.00E-05
centroid_9079	(Glycoside-Pentoside-Hexuronide) transporter domain p	8	5	11	102	centroid_9079	20.5552241	5.79E-06	centroid_9079	5.00E-05
centroid_9080	helix-turn-helix domain protein	8	5	11	102	centroid_9080	20.5552241	5.79E-06	centroid_9080	5.00E-05
centroid_9576	conserved hypothetical protein	8	5	11	102	centroid_9576	20.5552241	5.79E-06	centroid_9576	5.00E-05
centroid_5142	integrase core domain protein	8	94	11	13	centroid_5142	19.0309272	1.29E-05	centroid_5142	3.78E-05
centroid_14329	conserved hypothetical family protein	8	6	11	101	centroid_1432	18.2232846	1.96E-05	centroid_1432	0.0001066
centroid_5807	bacteriophage lambda tail assembly I family protein	8	6	11	101	centroid_5807	18.2232846	1.96E-05	centroid_5807	0.0001066
centroid_7366	phage late control gene D family protein	8	6	11	101	centroid_7366	18.2232846	1.96E-05	centroid_7366	0.0001066
centroid_7367	phage P2 GpU family protein	8	6	11	101	centroid_7367	18.2232846	1.96E-05	centroid_7367	0.0001066
centroid_7377	phage tail protein I	8	6	11	101	centroid_7377	18.2232846	1.96E-05	centroid_7377	0.0001066
centroid_9929	transposase family protein	8	93	11	14	centroid_9929	17.6507632	2.65E-05	centroid_9929	6.27E-05
centroid_5521	POTRA domain, ShlB-type family protein	8	92	11	15	centroid_5521	16.3820739	5.18E-05	centroid_5521	0.00010098
centroid_12694	bacteriophage lambda tail assembly I family protein	8	7	11	100	centroid_1269	16.2146209	5.66E-05	centroid_1269	0.00020876
centroid_14008	phage major tail tube protein	8	7	11	100	centroid_1400	16.2146209	5.66E-05	centroid_1400	0.00020876
centroid_14009	phage late control gene D family protein	8	7	11	100	centroid_1400	16.2146209	5.66E-05	centroid_1400	0.00020876
centroid_6796	conserved hypothetical protein	8	7	11	100	centroid_6796	16.2146209	5.66E-05	centroid_6796	0.00020876
centroid_7369	mu-like prophage FluMu gp41 family protein	8	7	11	100	centroid_7369	16.2146209	5.66E-05	centroid_7369	0.00020876
centroid_7370	phage major tail tube protein	8	7	11	100	centroid_7370	16.2146209	5.66E-05	centroid_7370	0.00020876
centroid_7371	phage tail sheath family protein	8	7	11	100	centroid_7371	16.2146209	5.66E-05	centroid_7371	0.00020876
centroid_958	pfkB carbohydrate kinase family protein	7	107	12	0	centroid_958	67.5426968	2.06E-16	centroid_958	2.59E-12
centroid_11278	aldo/keto reductase family protein	7	106	12	1	centroid_1127	60.9565398	5.83E-15	centroid_1127	3.17E-11
centroid_11408	zinc-binding dehydrogenase family protein	7	106	12	1	centroid_1140	60.9565398	5.83E-15	centroid_1140	3.17E-11
centroid_13575	pfkB carbohydrate kinase family protein	7	106	12	1	centroid_1357	60.9565398	5.83E-15	centroid_1357	3.17E-11
centroid_3166	major Facilitator Superfamily protein	7	106	12	1	centroid_3166	60.9565398	5.83E-15	centroid_3166	3.17E-11
centroid_3167	alcohol dehydrogenase GroES-like domain protein	7	106	12	1	centroid_3167	60.9565398	5.83E-15	centroid_3167	3.17E-11
centroid_3844	aldo/keto reductase family protein	7	106	12	1	centroid_3844	60.9565398	5.83E-15	centroid_3844	3.17E-11
centroid_3845	deoR-like helix-turn-helix domain protein	7	106	12	1	centroid_3845	60.9565398	5.83E-15	centroid_3845	3.17E-11
centroid_3846	inner membrane metabolite transport protein YdjE	7	106	12	1	centroid_3846	60.9565398	5.83E-15	centroid_3846	3.17E-11
centroid_4236	major Facilitator Superfamily protein	7	106	12	1	centroid_4236	60.9565398	5.83E-15	centroid_4236	3.17E-11
centroid_5297	pfkB carbohydrate kinase family protein	7	106	12	1	centroid_5297	60.9565398	5.83E-15	centroid_5297	3.17E-11
centroid_5512	sugar (and other) transporter family protein	7	106	12	1	centroid_5512	60.9565398	5.83E-15	centroid_5512	3.17E-11
centroid_7566	zinc-binding dehydrogenase family protein	7	106	12	1	centroid_7566	60.9565398	5.83E-15	centroid_7566	3.17E-11
centroid_7567	alcohol dehydrogenase GroES-like domain protein	7	106	12	1	centroid_7567	60.9565398	5.83E-15	centroid_7567	3.17E-11
centroid_9282	sugar (and other) transporter family protein	7	106	12	1	centroid_9282	60.9565398	5.83E-15	centroid_9282	3.17E-11
centroid_9283	sugar (and other) transporter family protein	7	106	12	1	centroid_9283	60.9565398	5.83E-15	centroid_9283	3.17E-11
centroid_12827	pfkB carbohydrate kinase family protein	7	105	12	2	centroid_1282	55.3167425	1.03E-13	centroid_1282	2.09E-10
centroid_3841	alcohol dehydrogenase GroES-like domain protein	7	105	12	2	centroid_3841	55.3167425	1.03E-13	centroid_3841	2.09E-10
centroid_3842	ketose-bisphosphate aldolase family protein	7	105	12	2	centroid_3842	55.3167425	1.03E-13	centroid_3842	2.09E-10
centroid_4320	zinc-binding dehydrogenase family protein	7	105	12	2	centroid_4320	55.3167425	1.03E-13	centroid_4320	2.09E-10
centroid_4916	conserved hypothetical protein	7	104	12	3	centroid_4916	50.4341713	1.23E-12	centroid_4916	9.87E-10
centroid_5511	conserved hypothetical protein	7	104	12	3	centroid_5511	50.4341713	1.23E-12	centroid_5511	9.87E-10
centroid_1012	sensory box protein	7	102	12	5	centroid_1012	42.4067049	7.41E-11	centroid_1012	1.19E-08
centroid_1013	response regulator	7	102	12	5	centroid_1013	42.4067049	7.41E-11	centroid_1013	1.19E-08
centroid_1014	acetate CoA-transferase subunit alpha	7	102	12	5	centroid_1014	42.4067049	7.41E-11	centroid_1014	1.19E-08
centroid_1015	acetate CoA-transferase subunit beta	7	102	12	5	centroid_1015	42.4067049	7.41E-11	centroid_1015	1.19E-08
centroid_1016	short-chain fatty acids transporter	7	102	12	5	centroid_1016	42.4067049	7.41E-11	centroid_1016	1.19E-08
centroid_1017	acetyl-CoA C-acetyltransferase family protein	7	102	12	5	centroid_1017	42.4067049	7.41E-11	centroid_1017	1.19E-08
centroid_10622	putative transposase DNA-binding domain protein	7	102	12	5	centroid_1062	42.4067049	7.41E-11	centroid_1062	1.19E-08
centroid_7693	ative signal transduction histidine-kinase atoS domain pro	7	102	12	5	centroid_7693	42.4067049	7.41E-11	centroid_7693	1.19E-08
centroid_7694	sensory box protein	7	102	12	5	centroid_7694	42.4067049	7.41E-11	centroid_7694	1.19E-08
centroid_8264	thiolase, N-terminal domain protein	7	102	12	5	centroid_8264	42.4067049	7.41E-11	centroid_8264	1.19E-08
centroid_8265	acetyl-CoA C-acetyltransferase family protein	7	102	12	5	centroid_8265	42.4067049	7.41E-11	centroid_8265	1.19E-08
centroid_17238	alpha amylase, catalytic domain protein	7	101	12	6	centroid_1723	39.0689867	4.09E-10	centroid_1723	3.36E-08
centroid_13252	fibronectin type III family protein	7	0	12	107	centroid_1325	35.0135702	3.27E-09	centroid_1325	5.97E-07
centroid_15179	RHS repeat-associated core domain protein	7	0	12	107	centroid_1517	35.0135702	3.27E-09	centroid_1517	5.97E-07
centroid_6837	hypothetical protein	7	0	12	107	centroid_6837	35.0135702	3.27E-09	centroid_6837	5.97E-07
centroid_14214	RHS repeat-associated core domain protein	7	1	12	106	centroid_1421	29.2087412	6.50E-08	centroid_1421	4.35E-06

centroid_5931	conserved hypothetical protein	7	1	12	106	centroid_5931	29.2087412	6.50E-08	centroid_5931	4.35E-06
centroid_7390	conserved hypothetical protein	7	1	12	106	centroid_7390	29.2087412	6.50E-08	centroid_7390	4.35E-06
centroid_7391	sigma-54 interaction domain protein	7	1	12	106	centroid_7391	29.2087412	6.50E-08	centroid_7391	4.35E-06
centroid_10179	CFA/I fimbrial subunit D	7	2	12	105	centroid_1017	24.7146695	6.65E-07	centroid_1017	1.79E-05
centroid_14398	conserved hypothetical protein	7	2	12	105	centroid_1439	24.7146695	6.65E-07	centroid_1439	1.79E-05
centroid_15118	conserved hypothetical protein	7	2	12	105	centroid_1511	24.7146695	6.65E-07	centroid_1511	1.79E-05
centroid_7398	RHS repeat-associated core domain protein	7	2	12	105	centroid_7398	24.7146695	6.65E-07	centroid_7398	1.79E-05
centroid_9014	outer membrane autotransporter barrel domain protein	7	2	12	105	centroid_9014	24.7146695	6.65E-07	centroid_9014	1.79E-05
centroid_9015	conserved hypothetical protein	7	2	12	105	centroid_9015	24.7146695	6.65E-07	centroid_9015	1.79E-05
centroid_9016	conserved hypothetical protein	7	2	12	105	centroid_9016	24.7146695	6.65E-07	centroid_9016	1.79E-05
centroid_9018	phage integrase family protein	7	2	12	105	centroid_9018	24.7146695	6.65E-07	centroid_9018	1.79E-05
centroid_9019	conserved hypothetical protein	7	2	12	105	centroid_9019	24.7146695	6.65E-07	centroid_9019	1.79E-05
centroid_6945	transposase DDE domain protein	7	93	12	14	centroid_6945	21.7403515	3.12E-06	centroid_6945	1.06E-05
centroid_8205	type VII secretion system (T7SS), usher family protein	7	93	12	14	centroid_8205	21.7403515	3.12E-06	centroid_8205	1.06E-05
centroid_12863	putative aTP-binding component of a transport system	7	3	12	104	centroid_1286	21.138612	4.27E-06	centroid_1286	5.42E-05
centroid_7542	RHS repeat-associated core domain protein	7	3	12	104	centroid_7542	21.138612	4.27E-06	centroid_7542	5.42E-05
centroid_12826	pfkB carbohydrate kinase family protein	7	92	12	15	centroid_1282	20.3135536	6.57E-06	centroid_1282	1.78E-05
centroid_3843	conserved hypothetical protein	7	91	12	16	centroid_3843	18.9927447	1.31E-05	centroid_3843	2.91E-05
centroid_4109	conserved hypothetical protein	7	89	12	18	centroid_4109	16.6271735	4.55E-05	centroid_4109	7.24E-05
centroid_13878	gram-negative porin family protein	7	5	12	102	centroid_1387	15.8242097	6.95E-05	centroid_1387	0.00029613
centroid_15850	putative conjugal transfer pilus assembly protein	7	5	12	102	centroid_1585	15.8242097	6.95E-05	centroid_1585	0.00029613
centroid_7236	exodeoxyribonuclease 8	7	5	12	102	centroid_7236	15.8242097	6.95E-05	centroid_7236	0.00029613
centroid_10619	conserved hypothetical protein	7	88	12	19	centroid_1061	15.5648439	7.97E-05	centroid_1061	0.00011038
centroid_12323	hypothetical protein	7	88	12	19	centroid_1232	15.5648439	7.97E-05	centroid_1232	0.00011038
centroid_4110	conserved hypothetical protein	7	88	12	19	centroid_4110	15.5648439	7.97E-05	centroid_4110	0.00011038
centroid_4977	conserved hypothetical protein	7	88	12	19	centroid_4977	15.5648439	7.97E-05	centroid_4977	0.00011038
centroid_14303	cyclic di-GMP phosphodiesterase YahA	6	104	13	3	centroid_1430	56.8868125	4.62E-14	centroid_1430	7.64E-11
centroid_6531	conserved hypothetical protein	6	95	13	12	centroid_6531	29.7000482	5.04E-08	centroid_6531	4.38E-07
centroid_10065	ash family protein	6	0	13	107	centroid_1006	28.8574397	7.79E-08	centroid_1006	5.51E-06
centroid_17227	ash family protein	6	0	13	107	centroid_1722	28.8574397	7.79E-08	centroid_1722	5.51E-06
centroid_17382	RHS repeat-associated core domain protein	6	0	13	107	centroid_1738	28.8574397	7.79E-08	centroid_1738	5.51E-06
centroid_5624	DKNYY family protein	6	0	13	107	centroid_5624	28.8574397	7.79E-08	centroid_5624	5.51E-06
centroid_5625	conserved hypothetical protein	6	0	13	107	centroid_5625	28.8574397	7.79E-08	centroid_5625	5.51E-06
centroid_5934	kinase-, DNA gyrase B-, and HSP90-like ATPase family	6	0	13	107	centroid_5934	28.8574397	7.79E-08	centroid_5934	5.51E-06
centroid_10047	conserved hypothetical protein	6	1	13	106	centroid_1004	23.3326582	1.36E-06	centroid_1004	3.50E-05
centroid_10084	RHS repeat-associated core domain protein	6	1	13	106	centroid_1008	23.3326582	1.36E-06	centroid_1008	3.50E-05
centroid_13103	type-1 fimbrial protein, A chain	6	1	13	106	centroid_1310	23.3326582	1.36E-06	centroid_1310	3.50E-05
centroid_14463	RHS repeat-associated core domain protein	6	1	13	106	centroid_1446	23.3326582	1.36E-06	centroid_1446	3.50E-05
centroid_18590	hypothetical protein	6	1	13	106	centroid_1859	23.3326582	1.36E-06	centroid_1859	3.50E-05
centroid_5892	3-hydroxybutyrate dehydrogenase family protein	6	1	13	106	centroid_5892	23.3326582	1.36E-06	centroid_5892	3.50E-05
centroid_5893	citrate transporter family protein	6	1	13	106	centroid_5893	23.3326582	1.36E-06	centroid_5893	3.50E-05
centroid_5894	droxyacyl-CoA dehydrogenase, NAD binding domain pro	6	1	13	106	centroid_5894	23.3326582	1.36E-06	centroid_5894	3.50E-05
centroid_5895	acetyl-CoA C-acetyltransferase family protein	6	1	13	106	centroid_5895	23.3326582	1.36E-06	centroid_5895	3.50E-05
centroid_5896	3-oxoacid CoA-transferase, B subunit	6	1	13	106	centroid_5896	23.3326582	1.36E-06	centroid_5896	3.50E-05
centroid_5897	3-oxoacid CoA-transferase, A subunit	6	1	13	106	centroid_5897	23.3326582	1.36E-06	centroid_5897	3.50E-05
centroid_5898	bacterial regulatory helix-turn-helix , lysR family protein	6	1	13	106	centroid_5898	23.3326582	1.36E-06	centroid_5898	3.50E-05
centroid_6103	caudovirales tail fibre assembly family protein	6	1	13	106	centroid_6103	23.3326582	1.36E-06	centroid_6103	3.50E-05
centroid_11183	transposase DDE domain protein	6	91	13	16	centroid_1118	23.1027324	1.54E-06	centroid_1118	4.55E-06
centroid_9017	bacterial regulatory, luxR family protein	6	2	13	105	centroid_9017	19.2156775	1.17E-05	centroid_9017	0.00012687
centroid_17120	initiator Replication family protein	6	87	13	20	centroid_1712	18.1490105	2.04E-05	centroid_1712	3.04E-05
centroid_4867	repFIB replication protein A	6	86	13	21	centroid_4867	17.1000685	3.55E-05	centroid_4867	4.63E-05
centroid_14025	phage integrase family protein	6	85	13	22	centroid_1402	16.1141171	5.96E-05	centroid_1402	6.93E-05
centroid_10184	putative mu prophage; Tail fiber protein	6	3	13	104	centroid_1018	16.0378372	6.21E-05	centroid_1018	0.0003449
centroid_11749	transposase family protein	6	3	13	104	centroid_1174	16.0378372	6.21E-05	centroid_1174	0.0003449
centroid_6866	putative dNA topoisomerase III	6	3	13	104	centroid_6866	16.0378372	6.21E-05	centroid_6866	0.0003449
centroid_8025	integrase core domain protein	6	3	13	104	centroid_8025	16.0378372	6.21E-05	centroid_8025	0.0003449
centroid_8920	protein TolA	6	3	13	104	centroid_8920	16.0378372	6.21E-05	centroid_8920	0.0003449
centroid_8921	tolA C-terminal family protein	6	3	13	104	centroid_8921	16.0378372	6.21E-05	centroid_8921	0.0003449
centroid_2684	conserved hypothetical protein	5	104	14	3	centroid_2684	63.5120575	1.59E-15	centroid_2684	5.04E-12
centroid_2685	inner membrane protein YigG	5	104	14	3	centroid_2685	63.5120575	1.59E-15	centroid_2685	5.04E-12
centroid_2947	papC N-terminal domain protein	5	103	14	4	centroid_2947	58.8810871	1.67E-14	centroid_2947	2.17E-11
centroid_3520	putative predicted protein	5	90	14	17	centroid_3520	26.0230048	3.37E-07	centroid_3520	1.03E-06
centroid_3521	conserved hypothetical protein	5	90	14	17	centroid_3521	26.0230048	3.37E-07	centroid_3521	1.03E-06
centroid_5163	putative domain protein	5	90	14	17	centroid_5163	26.0230048	3.37E-07	centroid_5163	1.03E-06
centroid_5556	hypothetical protein	5	88	14	19	centroid_5556	23.2940413	1.39E-06	centroid_5556	2.88E-06
centroid_11585	AAA domain family protein	5	0	14	107	centroid_1158	22.822425	1.78E-06	centroid_1158	4.76E-05
centroid_11597	hcp domain protein	5	0	14	107	centroid_1159	22.822425	1.78E-06	centroid_1159	4.76E-05
centroid_11763	fibronectin type III family protein	5	0	14	107	centroid_1176	22.822425	1.78E-06	centroid_1176	4.76E-05
centroid_12872	prophage CP4-57 integrase	5	0	14	107	centroid_1287	22.822425	1.78E-06	centroid_1287	4.76E-05
centroid_13004	AAA domain family protein	5	0	14	107	centroid_1300	22.822425	1.78E-06	centroid_1300	4.76E-05
centroid_13005	5-methylcytosine restriction system component family	5	0	14	107	centroid_1300	22.822425	1.78E-06	centroid_1300	4.76E-05
centroid_13006	conserved hypothetical protein	5	0	14	107	centroid_1300	22.822425	1.78E-06	centroid_1300	4.76E-05
centroid_13012	conserved hypothetical protein	5	0	14	107	centroid_1301	22.822425	1.78E-06	centroid_1301	4.76E-05
centroid_13013	conserved hypothetical protein	5	0	14	107	centroid_1301	22.822425	1.78E-06	centroid_1301	4.76E-05
centroid_13014	kinase domain protein	5	0	14	107	centroid_1301	22.822425	1.78E-06	centroid_1301	4.76E-05
centroid_13015	phage integrase family protein	5	0	14	107	centroid_1301	22.822425	1.78E-06	centroid_1301	4.76E-05
centroid_13470	conserved hypothetical protein	5	0	14	107	centroid_1347	22.822425	1.78E-06	centroid_1347	4.76E-05
centroid_15005	putative membrane protein	5	0	14	107	centroid_1500	22.822425	1.78E-06	centroid_1500	4.76E-05
centroid_15006	conserved hypothetical protein	5	0	14	107	centroid_1500	22.822425	1.78E-06	centroid_1500	4.76E-05
centroid_15016	conserved hypothetical protein	5	0	14	107	centroid_1501	22.822425	1.78E-06	centroid_1501	4.76E-05
centroid_17373	phage integrase family protein	5	0	14	107	centroid_1737	22.822425	1.78E-06	centroid_1737	4.76E-05
centroid_17374	conserved hypothetical protein	5	0	14	107	centroid_1737	22.822425	1.78E-06	centroid_1737	4.76E-05
centroid_17584	conserved hypothetical protein	5	0	14	107	centroid_1758	22.822425	1.78E-06	centroid_1758	4.76E-05
centroid_18529	conserved hypothetical protein	5	0	14	107	centroid_1852	22.822425	1.78E-06	centroid_1852	4.76E-05
centroid_5606	sigma-54 interaction domain protein	5	0	14	107	centroid_5606	22.822425	1.78E-06	centroid_5606	4.76E-05
centroid_5607	PRD domain protein	5	0	14	107	centroid_5607	22.822425	1.78E-06	centroid_5607	4.76E-05
centroid_5608	PTS system fructose IIA component family protein	5	0	14	107	centroid_5608	22.822425	1.78E-06	centroid_5608	4.76E-05
centroid_5609	PTS system sorbose subIIB component family protein	5	0	14	107	centroid_5609	22.822425	1.78E-06	centroid_5609	4.76E-05
centroid_5610	PTS system sorbose-specific iic component family protei	5	0	14	107	centroid_5610	22.822425	1.78E-06	centroid_5610	4.76E-05
centroid_5611	ystem mannose/fructose/sorbose IID component family	5	0	14	107	centroid_5611	22.822425	1.78E-06	centroid_5611	4.76E-05
centroid_5612	L-seryl-tRNA selenium transferase family protein	5	0	14	107	centroid_5612	22.822425	1.78E-06	centroid_5612	4.76E-05
centroid_5613	2-dehydro-3-deoxyphosphogluconate aldolase	5	0	14	107	centroid_5613	22.822425	1.78E-06	centroid_5613	4.76E-05
centroid_5972	type IV/VI secretion system , DotU family domain protein	5	0	14	107	centroid_5972	22.822425	1.78E-06	centroid_5972	4.76E-05
centroid_5973	conserved hypothetical protein	5	0	14	107	centroid_5973	22.822425	1.78E-06	centroid_5973	4.76E-05
centroid_6022	conserved hypothetical protein	5	0	14	107	centroid_6022	22.822425	1.78E-06	centroid_6022	4.76E-05
centroid_11498	conserved hypothetical protein	5	87	14	20	centroid_1149	22.053186	2.65E-06	centroid_1149	4.65E-06
centroid_10649	conserved hypothetical protein	5	83	14	24	centroid_1064	17.763653	2.50E-05	centroid_1064	2.63E-05
centroid_9921	hypothetical protein	5	83	14	24	centroid_9921	17.763653	2.50E-05	centroid_9921	2.63E-05
centroid_10981	putative iSSod4, transposase	5	1	14	106	centroid_1098	17.6643261	2.64E-05	centroid_1098	0.00025813
centroid_11022	transposase family protein	5	1	14	106	centroid_1102	17.6643261	2.64E-05	centroid_1102	0.00025813
centroid_11023	ABC transporter family protein	5	1	14	106	centroid_1102	17.6643261	2.64E-05	centroid_1102	0.00025813
centroid_11024	putative transporter protein AatB	5	1	14	106	centroid_1102	17.6643261	2.64E-05	centroid_1102	0.00025813
centroid_11025	outer membrane efflux family protein	5	1	14	106	centroid_1102	17.6643261	2.64E-05	centroid_1102	0.00025813
centroid_11026	ftsX-like permease family protein	5	1	14	106	centroid_1102	17.6643261	2.64E-05	centroid_1102	0.00025813
centroid_11027	hypothetical protein	5	1	14	106	centroid_1102	17.6643261	2.64E-05	centroid_1102	0.00025813
centroid_11108	putative aatD, apolipoprotein N-acyltransferase	5	1	14	106	centroid_1110	17.6643261	2.64E-05	centroid_1110	0.00025813
centroid_11786	transposase, Mutator family protein	5	1	14	106	centroid_1178	17.6643261	2.64E-05	centroid_1178	0.00025813

centroid_13208	transposase, Mutator family protein	5	1	14	106	centroid_1320	17.6643261	2.64E-05	centroid_1320	0.00025813
centroid_13417	transposase, Mutator family protein	5	1	14	106	centroid_1341	17.6643261	2.64E-05	centroid_1341	0.00025813
centroid_15120	putative type II plasmid partioning protein	5	1	14	106	centroid_1512	17.6643261	2.64E-05	centroid_1512	0.00025813
centroid_6102	side tail fiber family protein	5	1	14	106	centroid_610	17.6643261	2.64E-05	centroid_610	0.00025813
centroid_7351	CRISPR type I-E/ECOLI-associated protein CasA/Cse1	5	1	14	106	centroid_735	17.6643261	2.64E-05	centroid_735	0.00025813
centroid_16544	rhs element Vgr family protein	5	81	14	26	centroid_1654	15.9534263	6.49E-05	centroid_1654	5.66E-05
centroid_8383	protein YoaG	4	106	15	1	centroid_838	81.6808607	1.60E-19	centroid_838	5.82E-15
centroid_11950	FHIPEP family protein	4	105	15	2	centroid_1195	75.6577497	3.37E-18	centroid_1195	4.78E-14
centroid_17737	acterial extracellular solute-binding, 5 Middle family protei	4	105	15	2	centroid_1773	75.6577497	3.37E-18	centroid_1773	4.78E-14
centroid_17738	acterial extracellular solute-binding, 5 Middle family protei	4	105	15	2	centroid_1773	75.6577497	3.37E-18	centroid_1773	4.78E-14
centroid_2746	putative binding protein YgiS	4	105	15	2	centroid_274	75.6577497	3.37E-18	centroid_274	4.78E-14
centroid_3231	conserved hypothetical protein	4	104	15	3	centroid_323	70.3055829	5.08E-17	centroid_323	2.77E-13
centroid_5319	conserved hypothetical protein	4	104	15	3	centroid_531	70.3055829	5.08E-17	centroid_531	2.77E-13
centroid_5432	conserved hypothetical protein	4	102	15	5	centroid_543	61.2116897	5.13E-15	centroid_543	4.90E-12
centroid_2637	conserved hypothetical protein	4	101	15	6	centroid_263	57.3166749	3.71E-14	centroid_263	1.66E-11
centroid_2747	antitoxin MqsA	4	101	15	6	centroid_274	57.3166749	3.71E-14	centroid_274	1.66E-11
centroid_10926	gyrI-like small molecule binding domain protein	4	94	15	13	centroid_1092	37.8781358	7.53E-10	centroid_1092	8.80E-09
centroid_13091	mRNA interferase MqsR	4	92	15	15	centroid_1309	34.0025301	5.50E-09	centroid_1309	3.38E-08
centroid_2748	mRNA interferase MqsR	4	92	15	15	centroid_274	34.0025301	5.50E-09	centroid_274	3.38E-08
centroid_13465	conserved hypothetical protein	4	90	15	17	centroid_1346	30.6170079	3.14E-08	centroid_1346	1.14E-07
centroid_9932	rhs core with extension domain protein	4	90	15	17	centroid_993	30.6170079	3.14E-08	centroid_993	1.14E-07
centroid_11130	conserved hypothetical protein	4	89	15	18	centroid_1113	29.0802925	6.94E-08	centroid_1113	2.00E-07
centroid_7022	putative transposase	4	86	15	21	centroid_702	24.9908264	5.76E-07	centroid_702	9.51E-07
centroid_10218	conserved hypothetical protein	4	84	15	23	centroid_1021	22.6303595	1.96E-06	centroid_1021	2.43E-06
centroid_12706	conserved hypothetical protein	4	83	15	24	centroid_1270	21.5431377	3.46E-06	centroid_1270	3.78E-06
centroid_14802	bacterial Ig-like domain family protein	4	79	15	28	centroid_1480	17.7145191	2.57E-05	centroid_1480	1.91E-05
centroid_16797	bacterial Ig-like domain family protein	4	79	15	28	centroid_1679	17.7145191	2.57E-05	centroid_1679	1.91E-05
centroid_4917	conserved hypothetical protein	4	79	15	28	centroid_491	17.7145191	2.57E-05	centroid_491	1.91E-05
centroid_10028	fimbrial family protein	4	0	15	107	centroid_1002	16.9199499	3.90E-05	centroid_1002	0.00038725
centroid_10029	-negative pili assembly chaperone, N-terminal domain pr	4	0	15	107	centroid_1002	16.9199499	3.90E-05	centroid_1002	0.00038725
centroid_10030	type VII secretion system (T7SS), usher family protein	4	0	15	107	centroid_1003	16.9199499	3.90E-05	centroid_1003	0.00038725
centroid_10031	fimbrial family protein	4	0	15	107	centroid_1003	16.9199499	3.90E-05	centroid_1003	0.00038725
centroid_10032	fimbrial family protein	4	0	15	107	centroid_1003	16.9199499	3.90E-05	centroid_1003	0.00038725
centroid_11610	CRISPR-associated family protein	4	0	15	107	centroid_1161	16.9199499	3.90E-05	centroid_1161	0.00038725
centroid_11777	RHS repeat-associated core domain protein	4	0	15	107	centroid_1177	16.9199499	3.90E-05	centroid_1177	0.00038725
centroid_13007	N-6 DNA Methylase family protein	4	0	15	107	centroid_1300	16.9199499	3.90E-05	centroid_1300	0.00038725
centroid_13009	conserved hypothetical protein	4	0	15	107	centroid_1300	16.9199499	3.90E-05	centroid_1300	0.00038725
centroid_13010	conserved hypothetical protein	4	0	15	107	centroid_1301	16.9199499	3.90E-05	centroid_1301	0.00038725
centroid_13011	conserved hypothetical protein	4	0	15	107	centroid_1301	16.9199499	3.90E-05	centroid_1301	0.00038725
centroid_13018	conserved hypothetical protein	4	0	15	107	centroid_1301	16.9199499	3.90E-05	centroid_1301	0.00038725
centroid_13063	conserved hypothetical protein	4	0	15	107	centroid_1306	16.9199499	3.90E-05	centroid_1306	0.00038725
centroid_13073	putative dNA-damage-inducible protein	4	0	15	107	centroid_1307	16.9199499	3.90E-05	centroid_1307	0.00038725
centroid_13113	glycosyl transferases group 1 family protein	4	0	15	107	centroid_1311	16.9199499	3.90E-05	centroid_1311	0.00038725
centroid_13365	type VI secretion ATPase, ClpV1 family	4	0	15	107	centroid_1336	16.9199499	3.90E-05	centroid_1336	0.00038725
centroid_13479	phage integrase family protein	4	0	15	107	centroid_1347	16.9199499	3.90E-05	centroid_1347	0.00038725
centroid_14637	bacteriophage abortive infection AbiH family protein	4	0	15	107	centroid_1463	16.9199499	3.90E-05	centroid_1463	0.00038725
centroid_14929	conserved hypothetical protein	4	0	15	107	centroid_1492	16.9199499	3.90E-05	centroid_1492	0.00038725
centroid_14949	conserved hypothetical protein	4	0	15	107	centroid_1494	16.9199499	3.90E-05	centroid_1494	0.00038725
centroid_14950	conserved hypothetical protein	4	0	15	107	centroid_1495	16.9199499	3.90E-05	centroid_1495	0.00038725
centroid_15115	conserved hypothetical protein	4	0	15	107	centroid_1511	16.9199499	3.90E-05	centroid_1511	0.00038725
centroid_15194	RHS repeat-associated core domain protein	4	0	15	107	centroid_1519	16.9199499	3.90E-05	centroid_1519	0.00038725
centroid_15195	conserved hypothetical protein	4	0	15	107	centroid_1519	16.9199499	3.90E-05	centroid_1519	0.00038725
centroid_15535	conserved hypothetical protein	4	0	15	107	centroid_1553	16.9199499	3.90E-05	centroid_1553	0.00038725
centroid_15536	rhs element Vgr family protein	4	0	15	107	centroid_1553	16.9199499	3.90E-05	centroid_1553	0.00038725
centroid_15541	glycosyl transferases group 1 family protein	4	0	15	107	centroid_1554	16.9199499	3.90E-05	centroid_1554	0.00038725
centroid_15542	glycosyl transferases group 1 family protein	4	0	15	107	centroid_1554	16.9199499	3.90E-05	centroid_1554	0.00038725
centroid_15792	poxvirus D5 protein-like family protein	4	0	15	107	centroid_1579	16.9199499	3.90E-05	centroid_1579	0.00038725
centroid_15833	fimbrial family protein	4	0	15	107	centroid_1583	16.9199499	3.90E-05	centroid_1583	0.00038725
centroid_15870	conserved hypothetical protein	4	0	15	107	centroid_1587	16.9199499	3.90E-05	centroid_1587	0.00038725
centroid_17237	fibronectin type III family protein	4	0	15	107	centroid_1723	16.9199499	3.90E-05	centroid_1723	0.00038725
centroid_17364	dhesin biosynthesis transcription regulatory family protei	4	0	15	107	centroid_1736	16.9199499	3.90E-05	centroid_1736	0.00038725
centroid_17593	conserved hypothetical protein	4	0	15	107	centroid_1759	16.9199499	3.90E-05	centroid_1759	0.00038725
centroid_17595	RHS repeat-associated core domain protein	4	0	15	107	centroid_1759	16.9199499	3.90E-05	centroid_1759	0.00038725
centroid_18333	conserved hypothetical protein	4	0	15	107	centroid_1833	16.9199499	3.90E-05	centroid_1833	0.00038725
centroid_18397	conserved hypothetical protein	4	0	15	107	centroid_1839	16.9199499	3.90E-05	centroid_1839	0.00038725
centroid_5583	putative addiction module antidote protein	4	0	15	107	centroid_558	16.9199499	3.90E-05	centroid_558	0.00038725
centroid_5584	conserved hypothetical protein	4	0	15	107	centroid_558	16.9199499	3.90E-05	centroid_558	0.00038725
centroid_5585	SIR2-like domain protein	4	0	15	107	centroid_558	16.9199499	3.90E-05	centroid_558	0.00038725
centroid_5586	traC-like family protein	4	0	15	107	centroid_558	16.9199499	3.90E-05	centroid_558	0.00038725
centroid_5587	conserved hypothetical protein	4	0	15	107	centroid_558	16.9199499	3.90E-05	centroid_558	0.00038725
centroid_5588	P-type conjugative transfer protein TrbJ	4	0	15	107	centroid_558	16.9199499	3.90E-05	centroid_558	0.00038725
centroid_5589	hypothetical protein	4	0	15	107	centroid_558	16.9199499	3.90E-05	centroid_558	0.00038725
centroid_5590	P-type conjugative transfer protein TrbL	4	0	15	107	centroid_559	16.9199499	3.90E-05	centroid_559	0.00038725
centroid_5591	hypothetical protein	4	0	15	107	centroid_559	16.9199499	3.90E-05	centroid_559	0.00038725
centroid_5592	superfamily I DNA and RNA helicase	4	0	15	107	centroid_559	16.9199499	3.90E-05	centroid_559	0.00038725
centroid_5593	replication initiator A family protein	4	0	15	107	centroid_559	16.9199499	3.90E-05	centroid_559	0.00038725
centroid_5594	prophage CP4-57 regulatory family protein	4	0	15	107	centroid_559	16.9199499	3.90E-05	centroid_559	0.00038725
centroid_5595	phage integrase family protein	4	0	15	107	centroid_559	16.9199499	3.90E-05	centroid_559	0.00038725
centroid_5734	phage terminase, small subunit, P27 family	4	0	15	107	centroid_573	16.9199499	3.90E-05	centroid_573	0.00038725
centroid_5735	phage Terminase family protein	4	0	15	107	centroid_573	16.9199499	3.90E-05	centroid_573	0.00038725
centroid_5784	hypothetical protein	4	0	15	107	centroid_578	16.9199499	3.90E-05	centroid_578	0.00038725
centroid_5785	conserved hypothetical protein	4	0	15	107	centroid_578	16.9199499	3.90E-05	centroid_578	0.00038725
centroid_5835	CRISPR-associated endonuclease Cas1	4	0	15	107	centroid_583	16.9199499	3.90E-05	centroid_583	0.00038725
centroid_5836	CRISPR-associated helicase Cas3, subtype I-F/YPEST	4	0	15	107	centroid_583	16.9199499	3.90E-05	centroid_583	0.00038725
centroid_5837	CRISPR-associated family protein	4	0	15	107	centroid_583	16.9199499	3.90E-05	centroid_583	0.00038725
centroid_5838	CRISPR-associated protein Csy2	4	0	15	107	centroid_583	16.9199499	3.90E-05	centroid_583	0.00038725
centroid_5839	CRISPR-associated protein Csy3	4	0	15	107	centroid_583	16.9199499	3.90E-05	centroid_583	0.00038725
centroid_5840	CRISPR-associated endonuclease Cas6/Csy4	4	0	15	107	centroid_584	16.9199499	3.90E-05	centroid_584	0.00038725
centroid_5966	conserved hypothetical protein	4	0	15	107	centroid_596	16.9199499	3.90E-05	centroid_596	0.00038725
centroid_5967	conserved hypothetical protein	4	0	15	107	centroid_596	16.9199499	3.90E-05	centroid_596	0.00038725
centroid_5968	transglycosylase SLT domain protein	4	0	15	107	centroid_596	16.9199499	3.90E-05	centroid_596	0.00038725
centroid_5969	type VI secretion ATPase, ClpV1 family	4	0	15	107	centroid_596	16.9199499	3.90E-05	centroid_596	0.00038725
centroid_5970	type VI secretion system effector, Hcp1 family protein	4	0	15	107	centroid_597	16.9199499	3.90E-05	centroid_597	0.00038725
centroid_5971	ompA family protein	4	0	15	107	centroid_597	16.9199499	3.90E-05	centroid_597	0.00038725
centroid_5989	putative predicted protein	4	0	15	107	centroid_598	16.9199499	3.90E-05	centroid_598	0.00038725
centroid_6009	conserved hypothetical protein	4	0	15	107	centroid_600	16.9199499	3.90E-05	centroid_600	0.00038725
centroid_11968	bacterial Ig-like domain family protein	4	77	15	30	centroid_1196	16.0645352	6.12E-05	centroid_1196	3.98E-05
centroid_12473	bacterial Ig-like domain family protein	4	77	15	30	centroid_1247	16.0645352	6.12E-05	centroid_1247	3.98E-05
centroid_7843	hage tail tape measure protein, TP901 family, core regior	4	77	15	30	centroid_784	16.0645352	6.12E-05	centroid_784	3.98E-05
centroid_18009	bacterial Ig-like domain family protein	4	76	15	31	centroid_1800	15.2957631	9.19E-05	centroid_1800	5.64E-05
centroid_9965	biquinone/plastoquinone (complex I), various chains fami	3	104	16	3	centroid_996	77.2648409	1.50E-18	centroid_996	1.22E-14
centroid_13670	family 4 glycosyl hydrolase C-terminal domain protein	3	91	16	16	centroid_1367	37.2734767	1.03E-09	centroid_1367	5.23E-09
centroid_4439	family 4 glycosyl hydrolase C-terminal domain protein	3	91	16	16	centroid_443	37.2734767	1.03E-09	centroid_443	5.23E-09
centroid_2084	6-phospho-alpha-glucosidase	3	90	16	17	centroid_208	35.5077641	2.54E-09	centroid_208	9.84E-09
centroid_4438	4 glycosyl hydrolase family protein	3	90	16	17	centroid_443	35.5077641	2.54E-09	centroid_443	9.84E-09
centroid_14160	conserved domain protein	3	78	16	29	centroid_1416	20.4993606	5.97E-06	centroid_1416	3.70E-06
centroid_4432	caudovirales tail fibre assembly family protein	3	78	16	29	centroid_443	20.4993606	5.97E-06	centroid_443	3.70E-06

centroid_4849	conserved hypothetical protein	3	78	16	29	centroid_484	20.4993606	5.97E-06	centroid_484	3.70E-06
centroid_10333	tive pilus biosynthesis transmembrane anchor domain pr	3	77	16	30	centroid_1033	19.6077715	9.51E-06	centroid_1033	5.49E-06
centroid_10334	tive pilus biosynthesis transmembrane anchor domain pr	3	77	16	30	centroid_1033	19.6077715	9.51E-06	centroid_1033	5.49E-06
centroid_10335	hypothetical protein	3	77	16	30	centroid_1033	19.6077715	9.51E-06	centroid_1033	5.49E-06
centroid_12709	bacterial regulatory helix-turn-helix, AraC family protein	3	77	16	30	centroid_1270	19.6077715	9.51E-06	centroid_1270	5.49E-06
centroid_14026	type II/IV secretion system family protein	3	77	16	30	centroid_1402	19.6077715	9.51E-06	centroid_1402	5.49E-06
centroid_14191	CFA/I fimbrial subunit D domain protein	3	77	16	30	centroid_1419	19.6077715	9.51E-06	centroid_1419	5.49E-06
centroid_4850	type IV leader peptidase family protein	3	77	16	30	centroid_485	19.6077715	9.51E-06	centroid_485	5.49E-06
centroid_4851	putative pilus biosythesis protein	3	77	16	30	centroid_485	19.6077715	9.51E-06	centroid_485	5.49E-06
centroid_4852	type II secretion system (T2SS), F family protein	3	77	16	30	centroid_485	19.6077715	9.51E-06	centroid_485	5.49E-06
centroid_4853	type II/IV secretion system family protein	3	77	16	30	centroid_485	19.6077715	9.51E-06	centroid_485	5.49E-06
centroid_4854	putative lngG	3	77	16	30	centroid_485	19.6077715	9.51E-06	centroid_485	5.49E-06
centroid_4856	putative pilus biosynthesis transmembrane anchor protei	3	77	16	30	centroid_485	19.6077715	9.51E-06	centroid_485	5.49E-06
centroid_4858	toxin co-regulated pilus biosynthesis Q family protein	3	77	16	30	centroid_485	19.6077715	9.51E-06	centroid_485	5.49E-06
centroid_4859	repilin-type N-terminal cleavage/methylation domain prote	3	77	16	30	centroid_485	19.6077715	9.51E-06	centroid_485	5.49E-06
centroid_4861	putative lngX2	3	77	16	30	centroid_486	19.6077715	9.51E-06	centroid_486	5.49E-06
centroid_4862	transglycosylase SLT domain protein	3	77	16	30	centroid_486	19.6077715	9.51E-06	centroid_486	5.49E-06
centroid_4863	bacterial regulatory helix-turn-helix, AraC family protein	3	77	16	30	centroid_486	19.6077715	9.51E-06	centroid_486	5.49E-06
centroid_4864	dhesin biosynthesis transcription regulatory family protei	3	77	16	30	centroid_486	19.6077715	9.51E-06	centroid_486	5.49E-06
centroid_8277	bacterial type II and III secretion system family protein	3	77	16	30	centroid_827	19.6077715	9.51E-06	centroid_827	5.49E-06
centroid_4203	CFA/I fimbrial subunit D	3	76	16	31	centroid_420	18.7551243	1.49E-05	centroid_420	8.03E-06
centroid_4855	putative pilus biosynthesis protein	3	76	16	31	centroid_485	18.7551243	1.49E-05	centroid_485	8.03E-06
centroid_4857	bacterial type II and III secretion system family protein	3	76	16	31	centroid_485	18.7551243	1.49E-05	centroid_485	8.03E-06
centroid_14832	putative bifunctional chitinase/lysozyme domain protein	2	103	17	4	centroid_1483	79.3310379	5.25E-19	centroid_1483	2.06E-15
centroid_8579	putative bifunctional chitinase/lysozyme domain protein	2	103	17	4	centroid_857	79.3310379	5.25E-19	centroid_857	2.06E-15
centroid_2542	helix-turn-helix domain protein	2	100	17	7	centroid_254	66.6897044	3.18E-16	centroid_254	1.13E-13
centroid_3555	integrase core domain protein	2	100	17	7	centroid_355	66.6897044	3.18E-16	centroid_355	1.13E-13
centroid_5353	integrase core domain protein	2	99	17	8	centroid_535	63.1510407	1.91E-15	centroid_535	3.47E-13
centroid_7156	putative transposase	2	86	17	21	centroid_715	34.1292381	5.16E-09	centroid_715	7.12E-09
centroid_8329	putative iS91 transposase	2	81	17	26	centroid_832	27.6570165	1.45E-07	centroid_832	9.34E-08
centroid_9765	aatD, apolipoN-acyltransferase domain protein	2	77	17	30	centroid_976	23.4788918	1.26E-06	centroid_976	5.54E-07
centroid_13568	putative aatD, apolipoprotein N-acyltransferase	2	76	17	31	centroid_1356	22.5444185	2.05E-06	centroid_1356	8.38E-07
centroid_5525	conserved hypothetical protein	2	76	17	31	centroid_552	22.5444185	2.05E-06	centroid_552	8.38E-07
centroid_14468	putative aatD, apolipoprotein N-acyltransferase	2	73	17	34	centroid_1446	19.9640057	7.89E-06	centroid_1446	2.71E-06
centroid_6217	lasmic binding and sugar binding domain of LacI family pr	2	73	17	34	centroid_621	19.9640057	7.89E-06	centroid_621	2.71E-06
centroid_733	periplasmic binding domain protein	2	73	17	34	centroid_733	19.9640057	7.89E-06	centroid_733	2.71E-06
centroid_734	D-allose transporter subunit domain protein	2	73	17	34	centroid_734	19.9640057	7.89E-06	centroid_734	2.71E-06
centroid_735	ABC transporter family protein	2	73	17	34	centroid_735	19.9640057	7.89E-06	centroid_735	2.71E-06
centroid_9859	hypothetical protein	2	73	17	34	centroid_985	19.9640057	7.89E-06	centroid_985	2.71E-06
centroid_15730	putative membrane protein	2	72	17	35	centroid_1573	19.1711252	1.20E-05	centroid_1573	3.93E-06
centroid_4206	putative membrane protein	2	72	17	35	centroid_420	19.1711252	1.20E-05	centroid_420	3.93E-06
centroid_8347	aatD, apolipoN-acyltransferase domain protein	2	72	17	35	centroid_834	19.1711252	1.20E-05	centroid_834	3.93E-06
centroid_9257	protein DedA	1	106	18	1	centroid_925	103.661532	2.40E-24	centroid_925	1.28E-19
centroid_17467	type II secretion system protein L	1	101	18	6	centroid_1746	77.4464244	1.36E-18	centroid_1746	8.66E-16
centroid_17801	putative membrane protein	1	101	18	6	centroid_1780	77.4464244	1.36E-18	centroid_1780	8.66E-16
centroid_1781	inner membrane protein YmfA	1	101	18	6	centroid_178	77.4464244	1.36E-18	centroid_178	8.66E-16
centroid_3084	BFD-like [2Fe-2S] binding domain protein	1	101	18	6	centroid_308	77.4464244	1.36E-18	centroid_308	8.66E-16
centroid_3085	bacterioferritin	1	101	18	6	centroid_308	77.4464244	1.36E-18	centroid_308	8.66E-16
centroid_3086	e 4 prepilin-like proteins leader peptide-processing enzy	1	101	18	6	centroid_308	77.4464244	1.36E-18	centroid_308	8.66E-16
centroid_3087	type II secretion system (T2SS), M family protein	1	101	18	6	centroid_308	77.4464244	1.36E-18	centroid_308	8.66E-16
centroid_3098	AAA domain protein	1	101	18	6	centroid_309	77.4464244	1.36E-18	centroid_309	8.66E-16
centroid_3099	putative peptidoglycan binding domain protein	1	101	18	6	centroid_309	77.4464244	1.36E-18	centroid_309	8.66E-16
centroid_3100	lcium-binding protein required for initiation of chromosome	1	101	18	6	centroid_310	77.4464244	1.36E-18	centroid_310	8.66E-16
centroid_5500	gspL periplasmic domain protein	1	101	18	6	centroid_550	77.4464244	1.36E-18	centroid_550	8.66E-16
centroid_8581	putative general secretion pathway protein A	1	101	18	6	centroid_858	77.4464244	1.36E-18	centroid_858	8.66E-16
centroid_9733	conserved hypothetical protein	1	101	18	6	centroid_973	77.4464244	1.36E-18	centroid_973	8.66E-16
centroid_9734	hypothetical protein	1	101	18	6	centroid_973	77.4464244	1.36E-18	centroid_973	8.66E-16
centroid_10869	type II secretion system protein H	1	100	18	7	centroid_1086	73.4622102	1.03E-17	centroid_1086	3.06E-15
centroid_12074	type II secretion system protein H	1	100	18	7	centroid_1207	73.4622102	1.03E-17	centroid_1207	3.06E-15
centroid_17465	type II secretion system (T2SS), F family protein	1	100	18	7	centroid_1746	73.4622102	1.03E-17	centroid_1746	3.06E-15
centroid_17466	type II secretion system (T2SS), F family protein	1	100	18	7	centroid_1746	73.4622102	1.03E-17	centroid_1746	3.06E-15
centroid_3083	putative bifunctional chitinase/lysozyme	1	100	18	7	centroid_308	73.4622102	1.03E-17	centroid_308	3.06E-15
centroid_3089	type II secretion system (T2SS), K family protein	1	100	18	7	centroid_308	73.4622102	1.03E-17	centroid_308	3.06E-15
centroid_3090	repilin-type N-terminal cleavage/methylation domain prote	1	100	18	7	centroid_309	73.4622102	1.03E-17	centroid_309	3.06E-15
centroid_3091	type II secretion system protein I	1	100	18	7	centroid_309	73.4622102	1.03E-17	centroid_309	3.06E-15
centroid_3092	type II secretion system protein H	1	100	18	7	centroid_309	73.4622102	1.03E-17	centroid_309	3.06E-15
centroid_3093	type II secretion system protein G	1	100	18	7	centroid_309	73.4622102	1.03E-17	centroid_309	3.06E-15
centroid_3094	type II secretion system protein F	1	100	18	7	centroid_309	73.4622102	1.03E-17	centroid_309	3.06E-15
centroid_3095	type II secretion system protein E	1	100	18	7	centroid_309	73.4622102	1.03E-17	centroid_309	3.06E-15
centroid_4742	repilin-type N-terminal cleavage/methylation domain prote	1	100	18	7	centroid_474	73.4622102	1.03E-17	centroid_474	3.06E-15
centroid_7722	type II secretion system protein F	1	100	18	7	centroid_772	73.4622102	1.03E-17	centroid_772	3.06E-15
centroid_7723	putative type II secretion system F domain protein	1	100	18	7	centroid_772	73.4622102	1.03E-17	centroid_772	3.06E-15
centroid_8069	type II secretion system protein D	1	100	18	7	centroid_806	73.4622102	1.03E-17	centroid_806	3.06E-15
centroid_8070	type II secretion system protein L	1	100	18	7	centroid_807	73.4622102	1.03E-17	centroid_807	3.06E-15
centroid_8580	carbohydrate binding domain protein	1	100	18	7	centroid_858	73.4622102	1.03E-17	centroid_858	3.06E-15
centroid_3096	type II secretion system protein D	1	98	18	9	centroid_309	66.3796449	3.72E-16	centroid_309	2.93E-14
centroid_8068	type II secretion system D domain protein	1	98	18	9	centroid_806	66.3796449	3.72E-16	centroid_806	2.93E-14
centroid_3097	type II secretion system protein C	1	97	18	10	centroid_309	63.2180275	1.85E-15	centroid_309	8.13E-14
centroid_2331	CRISPR-associated endonuclease Cas1	1	91	18	16	centroid_233	48.1569144	3.93E-12	centroid_233	1.29E-11
centroid_4611	SPR-associated endoribonuclease Cas2, subtype I-E/EC	1	91	18	16	centroid_461	48.1569144	3.93E-12	centroid_461	1.29E-11
centroid_9502	SPR-associated endoribonuclease Cas2, subtype I-E/EC	1	91	18	16	centroid_950	48.1569144	3.93E-12	centroid_950	1.29E-11
centroid_10582	SPR-associated protein Cas6/Cse3/CasE, subtype I-E/E	1	89	18	18	centroid_1058	44.253394	2.89E-11	centroid_1058	5.19E-11
centroid_17493	hypothetical protein	1	88	18	19	centroid_1749	42.4601282	7.21E-11	centroid_1749	1.00E-10
centroid_13505	RHS repeat-associated core domain protein	1	87	18	20	centroid_1350	40.7614101	1.72E-10	centroid_1350	1.88E-10
centroid_3088	type II secretion system protein L	1	87	18	20	centroid_308	40.7614101	1.72E-10	centroid_308	1.88E-10
centroid_2332	SPR-associated protein Cas6/Cse3/CasE, subtype I-E/E	1	83	18	24	centroid_233	34.7770536	3.70E-09	centroid_233	1.90E-09
centroid_9256	ulp1 protease family, C-terminal catalytic domain protein	1	77	18	30	centroid_925	27.6754299	1.43E-07	centroid_925	3.66E-08
centroid_17455	conserved hypothetical protein	1	74	18	33	centroid_1745	24.7536171	6.51E-07	centroid_1745	1.35E-07
centroid_4699	conserved hypothetical protein	1	74	18	33	centroid_469	24.7536171	6.51E-07	centroid_469	1.35E-07
centroid_7700	mRNA interferase HigB	1	74	18	33	centroid_770	24.7536171	6.51E-07	centroid_770	1.35E-07
centroid_16886	orn/Lys/Arg decarboxylase, major domain protein	1	73	18	34	centroid_1688	23.8549911	1.04E-06	centroid_1688	2.03E-07
centroid_17756	conserved hypothetical protein	1	73	18	34	centroid_1775	23.8549911	1.04E-06	centroid_1775	2.03E-07
centroid_1092	mRNA interferase HigB	1	69	18	38	centroid_109	20.5834604	5.71E-06	centroid_109	9.62E-07
centroid_9677	putative transposase	1	67	18	40	centroid_967	19.1182125	1.23E-05	centroid_967	1.98E-06
centroid_3558	caudovirales tail fibre assembly family protein	1	65	18	42	centroid_355	17.7515874	2.52E-05	centroid_355	5.06E-06
centroid_14007	RhsB domain protein	1	63	18	44	centroid_1400	16.4741263	4.93E-05	centroid_1400	8.26E-06
centroid_14128	e kinase-, DNA gyrase B-, and HSP90-like ATPase family	1	63	18	44	centroid_1412	16.4741263	4.93E-05	centroid_1412	8.26E-06
centroid_3474	putative copper-binding protein PcoE	1	63	18	44	centroid_347	16.4741263	4.93E-05	centroid_347	8.26E-06
centroid_373	conserved hypothetical protein	1	63	18	44	centroid_373	16.4741263	4.93E-05	centroid_373	8.26E-06
centroid_8305	RHS repeat-associated core domain protein	1	63	18	44	centroid_830	16.4741263	4.93E-05	centroid_830	8.26E-06
centroid_9920	RHS repeat-associated core domain protein	1	63	18	44	centroid_992	16.4741263	4.93E-05	centroid_992	8.26E-06
centroid_10828	putative metalloprotease YebA domain protein	1	62	18	45	centroid_1082	15.8662076	6.80E-05	centroid_1082	2.13E-05
centroid_10911	tnsA endonuclease N terminal family protein	1	62	18	45	centroid_1091	15.8662076	6.80E-05	centroid_1091	2.13E-05
centroid_10928	peptidase M23 family protein	1	62	18	45	centroid_1092	15.8662076	6.80E-05	centroid_1092	2.13E-05
centroid_11449	putative tn7-like transposition protein C	1	62	18	45	centroid_1144	15.8662076	6.80E-05	centroid_1144	2.13E-05
centroid_12338	conserved hypothetical protein	1	62	18	45	centroid_1233	15.8662076	6.80E-05	centroid_1233	2.13E-05
centroid_18160	putative pcoS	1	62	18	45	centroid_1816	15.8662076	6.80E-05	centroid_1816	2.13E-05

centroid_18161	efflux transporter, RND family, MFP subunit	1	62	18	45	centroid_1816	15.8662076	6.80E-05	centroid_1816	2.13E-05
centroid_18179	heavy metal sensor kinase family protein	1	62	18	45	centroid_1817	15.8662076	6.80E-05	centroid_1817	2.13E-05
centroid_3476	response regulator	1	62	18	45	centroid_3476	15.8662076	6.80E-05	centroid_3476	2.13E-05
centroid_3477	copper resistance D family protein	1	62	18	45	centroid_3477	15.8662076	6.80E-05	centroid_3477	2.13E-05
centroid_3478	copper resistance protein C	1	62	18	45	centroid_3478	15.8662076	6.80E-05	centroid_3478	2.13E-05
centroid_3479	copper resistance protein B	1	62	18	45	centroid_3479	15.8662076	6.80E-05	centroid_3479	2.13E-05
centroid_3480	copper resistance protein A	1	62	18	45	centroid_3480	15.8662076	6.80E-05	centroid_3480	2.13E-05
centroid_3481	putative copper resistant protein PcoE	1	62	18	45	centroid_3481	15.8662076	6.80E-05	centroid_3481	2.13E-05
centroid_3483	conserved hypothetical protein	1	62	18	45	centroid_3483	15.8662076	6.80E-05	centroid_3483	2.13E-05
centroid_3484	copper-translocating P-type ATPase	1	62	18	45	centroid_3484	15.8662076	6.80E-05	centroid_3484	2.13E-05
centroid_4049	tniQ family protein	1	62	18	45	centroid_4049	15.8662076	6.80E-05	centroid_4049	2.13E-05
centroid_3475	heavy metal sensor kinase family protein	1	61	18	46	centroid_3475	15.2775631	9.28E-05	centroid_3475	2.23E-05
centroid_3486	cation efflux system protein CusA	1	61	18	46	centroid_3486	15.2775631	9.28E-05	centroid_3486	2.23E-05
centroid_3487	efflux transporter, RND family, MFP subunit	1	61	18	46	centroid_3487	15.2775631	9.28E-05	centroid_3487	2.23E-05
centroid_3488	cation efflux system protein CusF	1	61	18	46	centroid_3488	15.2775631	9.28E-05	centroid_3488	2.23E-05
centroid_3489	ansporter, outer membrane factor (OMF) lipo, NodT family	1	61	18	46	centroid_3489	15.2775631	9.28E-05	centroid_3489	2.23E-05
centroid_3490	transcriptional regulatory protein CusR	1	61	18	46	centroid_3490	15.2775631	9.28E-05	centroid_3490	2.23E-05
centroid_3491	heavy metal sensor kinase family protein	1	61	18	46	centroid_3491	15.2775631	9.28E-05	centroid_3491	2.23E-05
centroid_5187	conserved hypothetical protein	1	61	18	46	centroid_5187	15.2775631	9.28E-05	centroid_5187	2.23E-05
centroid_1113	inner membrane protein YhaI	0	106	19	1	centroid_1113	111.278649	5.14E-26	centroid_1113	1.26E-21
centroid_1340	conserved hypothetical protein	0	106	19	1	centroid_1340	111.278649	5.14E-26	centroid_1340	1.26E-21
centroid_14713	conserved hypothetical protein	0	106	19	1	centroid_1471	111.278649	5.14E-26	centroid_1471	1.26E-21
centroid_14714	conserved hypothetical protein	0	106	19	1	centroid_1471	111.278649	5.14E-26	centroid_1471	1.26E-21
centroid_1035	2-keto-3-deoxy-L-rhamnonate aldolase	0	105	19	2	centroid_1035	104.915298	1.27E-24	centroid_1035	1.32E-20
centroid_1036	major Facilitator Superfamily protein	0	105	19	2	centroid_1036	104.915298	1.27E-24	centroid_1036	1.32E-20
centroid_1037	major Facilitator Superfamily protein	0	105	19	2	centroid_1037	104.915298	1.27E-24	centroid_1037	1.32E-20
centroid_1038	L-rhamnonate dehydratase	0	105	19	2	centroid_1038	104.915298	1.27E-24	centroid_1038	1.32E-20
centroid_1039	conserved hypothetical protein	0	105	19	2	centroid_1039	104.915298	1.27E-24	centroid_1039	1.32E-20
centroid_16153	bacterial transcriptional regulator family protein	0	105	19	2	centroid_1615	104.915298	1.27E-24	centroid_1615	1.32E-20
centroid_16154	iclR helix-turn-helix domain protein	0	105	19	2	centroid_1615	104.915298	1.27E-24	centroid_1615	1.32E-20
centroid_4273	inner membrane transport protein RhmT	0	105	19	2	centroid_4273	104.915298	1.27E-24	centroid_4273	1.32E-20
centroid_7695	bacterial transcriptional regulator family protein	0	105	19	2	centroid_7695	104.915298	1.27E-24	centroid_7695	1.32E-20
centroid_7696	iclR helix-turn-helix domain protein	0	105	19	2	centroid_7696	104.915298	1.27E-24	centroid_7696	1.32E-20
centroid_16783	hypothetical protein	0	104	19	3	centroid_1678	99.130452	2.36E-23	centroid_1678	9.69E-20
centroid_9217	conserved hypothetical protein	0	104	19	3	centroid_9217	99.130452	2.36E-23	centroid_9217	9.69E-20
centroid_16928	yiaA/B two helix domain protein	0	103	19	4	centroid_1692	93.8486555	3.41E-22	centroid_1692	5.57E-19
centroid_4665	conserved hypothetical protein	0	102	19	5	centroid_4665	89.0070274	3.93E-21	centroid_4665	2.67E-18
centroid_4710	gntP permease family protein	0	102	19	5	centroid_4710	89.0070274	3.93E-21	centroid_4710	2.67E-18
centroid_4814	conserved hypothetical protein	0	102	19	5	centroid_4814	89.0070274	3.93E-21	centroid_4814	2.67E-18
centroid_17796	outer membrane autotransporter barrel domain protein	0	101	19	6	centroid_1779	84.5527482	3.74E-20	centroid_1779	1.11E-17
centroid_4666	outer membrane autotransporter barrel domain protein	0	101	19	6	centroid_4666	84.5527482	3.74E-20	centroid_4666	1.11E-17
centroid_8894	outer membrane autotransporter barrel domain protein	0	101	19	6	centroid_8894	84.5527482	3.74E-20	centroid_8894	1.11E-17
centroid_13907	conserved hypothetical protein	0	99	19	8	centroid_1390	76.6340831	2.06E-18	centroid_1390	1.40E-16
centroid_1481	putative lipoprotein	0	99	19	8	centroid_1481	76.6340831	2.06E-18	centroid_1481	1.40E-16
centroid_1482	conserved hypothetical protein	0	99	19	8	centroid_1482	76.6340831	2.06E-18	centroid_1482	1.40E-16
centroid_8471	conserved hypothetical protein	0	99	19	8	centroid_8471	76.6340831	2.06E-18	centroid_8471	1.40E-16
centroid_9316	protein PhnP	0	97	19	10	centroid_9316	69.8077176	6.54E-17	centroid_9316	1.26E-15
centroid_4739	WGR domain protein	0	94	19	13	centroid_4739	61.1682266	5.24E-15	centroid_4739	2.18E-14
centroid_526	conserved hypothetical protein	0	94	19	13	centroid_526	61.1682266	5.24E-15	centroid_526	2.18E-14
centroid_15969	CRISPR-associated endonuclease/helicase Cas3	0	92	19	15	centroid_1596	56.2556611	6.36E-14	centroid_1596	1.17E-13
centroid_15998	SPR-associated endonuclease/helicase Cas3 domain pro	0	91	19	16	centroid_1599	54.0099436	1.99E-13	centroid_1599	2.55E-13
centroid_17627	fimbrial subunit ElfA	0	90	19	17	centroid_1762	51.8890064	5.87E-13	centroid_1762	5.41E-13
centroid_9313	CRISPR-associated endonuclease/helicase Cas3	0	90	19	17	centroid_9313	51.8890064	5.87E-13	centroid_9313	5.41E-13
centroid_12408	RISPR-associated protein Cas5/CasD, subtype I-E/ECO	0	89	19	18	centroid_1240	49.8827327	1.63E-12	centroid_1240	1.11E-12
centroid_13690	CT1975-like family protein	0	89	19	18	centroid_1369	49.8827327	1.63E-12	centroid_1369	1.11E-12
centroid_16204	CRISPR type I-E/ECOLI-associated protein CasA/Cse1	0	89	19	18	centroid_1620	49.8827327	1.63E-12	centroid_1620	1.11E-12
centroid_2335	CRISPR type I-E/ECOLI-associated protein CasB/Cse2	0	89	19	18	centroid_2335	49.8827327	1.63E-12	centroid_2335	1.11E-12
centroid_5348	CT1975-like family protein	0	89	19	18	centroid_5348	49.8827327	1.63E-12	centroid_5348	1.11E-12
centroid_5358	SPR-associated protein Cas7/Cse4/CasC, subtype I-E/E(	0	89	19	18	centroid_5358	49.8827327	1.63E-12	centroid_5358	1.11E-12
centroid_17647	CRISPR type I-E/ECOLI-associated protein CasA/Cse1	0	88	19	19	centroid_1764	47.9820707	4.30E-12	centroid_1764	2.22E-12
centroid_4613	CRISPR type I-E/ECOLI-associated protein CasA/Cse1	0	88	19	19	centroid_4613	47.9820707	4.30E-12	centroid_4613	2.22E-12
centroid_6137	CRISPR type I-E/ECOLI-associated protein CasA/Cse1	0	88	19	19	centroid_6137	47.9820707	4.30E-12	centroid_6137	2.22E-12
centroid_4612	RISPR-associated protein Cas5/CasD, subtype I-E/ECO	0	87	19	20	centroid_4612	46.178897	1.08E-11	centroid_4612	4.33E-12
centroid_12681	RISPR-associated protein Cas5/CasD, subtype I-E/ECO	0	85	19	22	centroid_1268	42.8364841	5.95E-11	centroid_1268	1.54E-11
centroid_9314	SPR-associated protein Cas6/Cse3/CasE, subtype I-E/E(	0	85	19	22	centroid_9314	42.8364841	5.95E-11	centroid_9314	1.54E-11
centroid_16849	hypothetical protein	0	83	19	24	centroid_1684	39.8050679	2.81E-10	centroid_1684	5.03E-11
centroid_2333	RISPR-associated protein Cas5/CasD, subtype I-E/ECO	0	83	19	24	centroid_2333	39.8050679	2.81E-10	centroid_2333	5.03E-11
centroid_5188	transposase DDE domain protein	0	80	19	27	centroid_5188	35.7523383	2.24E-09	centroid_5188	2.61E-10
centroid_580	biquinone/plastoquinone (complex I), various chains fami	0	78	19	29	centroid_580	33.3320571	7.77E-09	centroid_580	7.26E-10
centroid_2334	SPR-associated protein Cas7/Cse4/CasC, subtype I-E/E(	0	76	19	31	centroid_2334	31.1054814	2.44E-08	centroid_2334	1.91E-09
centroid_270	conserved hypothetical protein	0	76	19	31	centroid_270	31.1054814	2.44E-08	centroid_270	1.91E-09
centroid_4397	ftsK/SpoIIIE family protein	0	76	19	31	centroid_4397	31.1054814	2.44E-08	centroid_4397	1.91E-09
centroid_11294	porin, autotransporter (AT) family	0	75	19	32	centroid_1129	30.0577136	4.19E-08	centroid_1129	3.05E-09
centroid_13494	e ATP-binding component of a transport system domain p	0	75	19	32	centroid_1349	30.0577136	4.19E-08	centroid_1349	3.05E-09
centroid_14668	hypothetical protein	0	75	19	32	centroid_1466	30.0577136	4.19E-08	centroid_1466	3.05E-09
centroid_15981	H+ symporter family protein	0	75	19	32	centroid_1598	30.0577136	4.19E-08	centroid_1598	3.05E-09
centroid_16661	toxin YafO, type II toxin-antitoxin system family protein	0	75	19	32	centroid_1666	30.0577136	4.19E-08	centroid_1666	3.05E-09
centroid_17797	conserved hypothetical protein	0	75	19	32	centroid_1779	30.0577136	4.19E-08	centroid_1779	3.05E-09
centroid_1911	outer membrane autotransporter barrel domain protein	0	75	19	32	centroid_1911	30.0577136	4.19E-08	centroid_1911	3.05E-09
centroid_2822	antitoxin YafN	0	75	19	32	centroid_2822	30.0577136	4.19E-08	centroid_2822	3.05E-09
centroid_11286	D-serine deaminase transcriptional activator	0	74	19	33	centroid_1128	29.050267	7.05E-08	centroid_1128	4.80E-09
centroid_1251	dsdX permease	0	74	19	33	centroid_1251	29.050267	7.05E-08	centroid_1251	4.80E-09
centroid_2823	mRNA interferase YafO	0	74	19	33	centroid_2823	29.050267	7.05E-08	centroid_2823	4.80E-09
centroid_10353	orn/Lys/Arg decarboxylase, major domain protein	0	73	19	34	centroid_1035	28.0808602	1.16E-07	centroid_1035	7.49E-09
centroid_12500	ornithine decarboxylase, inducible domain protein	0	73	19	34	centroid_1250	28.0808602	1.16E-07	centroid_1250	7.49E-09
centroid_1484	conserved hypothetical protein	0	73	19	34	centroid_1484	28.0808602	1.16E-07	centroid_1484	7.49E-09
centroid_16491	orn/Lys/Arg decarboxylase, N-terminal domain protein	0	73	19	34	centroid_1649	28.0808602	1.16E-07	centroid_1649	7.49E-09
centroid_16443	D-serine deaminase transcriptional activator	0	72	19	35	centroid_1644	27.1473807	1.89E-07	centroid_1644	1.16E-08
centroid_2671	phosphodiesterase / nucleotide pyrophosphatase family p	0	72	19	35	centroid_2671	27.1473807	1.89E-07	centroid_2671	1.16E-08
centroid_1250	D-serine deaminase transcriptional activator	0	71	19	36	centroid_1250	26.2478699	3.00E-07	centroid_1250	1.76E-08
centroid_10799	ı-negative pili assembly chaperone, C-terminal domain pr	0	70	19	37	centroid_1079	25.3805091	4.71E-07	centroid_1079	2.67E-08
centroid_16913	putative membrane protein	0	70	19	37	centroid_1691	25.3805091	4.71E-07	centroid_1691	2.67E-08
centroid_1239	ı-negative pili assembly chaperone, N-terminal domain pr	0	69	19	38	centroid_1239	24.5436071	7.26E-07	centroid_1239	4.01E-08
centroid_16914	ı-negative pili assembly chaperone, N-terminal domain pr	0	69	19	38	centroid_1691	24.5436071	7.26E-07	centroid_1691	4.01E-08
centroid_4811	putative 4'-phosphopantetheinyl transferase EntD	0	69	19	38	centroid_4811	24.5436071	7.26E-07	centroid_4811	4.01E-08
centroid_1175	von Willebrand factor family protein	0	65	19	42	centroid_1175	21.4706503	3.59E-06	centroid_1175	1.87E-07
centroid_6930	conserved hypothetical protein	0	65	19	42	centroid_6930	21.4706503	3.59E-06	centroid_6930	1.87E-07
centroid_10249	conserved hypothetical protein	0	64	19	43	centroid_1024	20.764435	5.19E-06	centroid_1024	2.69E-07
centroid_1179	UBA/TS-N domain protein	0	64	19	43	centroid_1179	20.764435	5.19E-06	centroid_1179	2.69E-07
centroid_1180	conserved hypothetical protein	0	64	19	43	centroid_1180	20.764435	5.19E-06	centroid_1180	2.69E-07
centroid_1181	tetratricopeptide repeat family protein	0	64	19	43	centroid_1181	20.764435	5.19E-06	centroid_1181	2.69E-07
centroid_17103	CRISPR type I-E/ECOLI-associated protein CasA/Cse1	0	64	19	43	centroid_1710	20.764435	5.19E-06	centroid_1710	2.69E-07
centroid_17771	hypothetical protein	0	64	19	43	centroid_1777	20.764435	5.19E-06	centroid_1777	2.69E-07
centroid_2946	ı-negative pili assembly chaperone, C-terminal domain pr	0	64	19	43	centroid_2946	20.764435	5.19E-06	centroid_2946	2.69E-07
centroid_1235	conserved hypothetical protein	0	63	19	44	centroid_1235	20.080669	7.42E-06	centroid_1235	7.71E-07
centroid_1236	fimbrial family protein	0	63	19	44	centroid_1236	20.080669	7.42E-06	centroid_1236	7.71E-07
centroid_1237	fimbrial family protein	0	63	19	44	centroid_1237	20.080669	7.42E-06	centroid_1237	7.71E-07

centroid_1238	fimbrial family protein	0	63	19	44	centroid_1238	20.080669	7.42E-06	centroid_1238	7.71E-07
centroid_13865	fimbrial family protein	0	63	19	44	centroid_1386	20.080669	7.42E-06	centroid_1386	7.71E-07
centroid_14725	o-4-hydroxy-6-hydroxymethyldihydropteridine diphospho	0	61	19	46	centroid_1472	18.7763462	1.47E-05	centroid_1472	9.62E-07
centroid_16583	CRISPR type I-E/ECOLI-associated protein CasA/Cse1	0	61	19	46	centroid_1658	18.7763462	1.47E-05	centroid_1658	9.62E-07
centroid_17155	helix-turn-helix domain protein	0	61	19	46	centroid_1715	18.7763462	1.47E-05	centroid_1715	9.62E-07
centroid_1178	conserved hypothetical protein	0	60	19	47	centroid_1178	18.1538769	2.04E-05	centroid_1178	1.22E-06
centroid_2336	CRISPR type I-E/ECOLI-associated protein CasA/Cse1	0	60	19	47	centroid_2336	18.1538769	2.04E-05	centroid_2336	1.22E-06
centroid_3323	integrase core domain protein	0	60	19	47	centroid_3323	18.1538769	2.04E-05	centroid_3323	1.22E-06
centroid_4332	calcineurin-like phosphoesterase family protein	0	60	19	47	centroid_4332	18.1538769	2.04E-05	centroid_4332	1.22E-06
centroid_6506	hypothetical protein	0	60	19	47	centroid_6506	18.1538769	2.04E-05	centroid_6506	1.22E-06
centroid_7671	conserved hypothetical protein	0	60	19	47	centroid_7671	18.1538769	2.04E-05	centroid_7671	1.22E-06
centroid_4860	CFA/III pilin	0	59	19	48	centroid_4860	17.5500229	2.80E-05	centroid_4860	1.61E-06
centroid_864	putative membrane protein	0	59	19	48	centroid_864	17.5500229	2.80E-05	centroid_864	1.61E-06
centroid_10746	putative ybl54	0	58	19	49	centroid_1074	16.9639645	3.81E-05	centroid_1074	4.09E-06
centroid_15898	putative predicted protein	0	58	19	49	centroid_1589	16.9639645	3.81E-05	centroid_1589	4.09E-06
centroid_17655	conserved hypothetical protein	0	58	19	49	centroid_1765	16.9639645	3.81E-05	centroid_1765	4.09E-06
centroid_8703	putative ybl54	0	58	19	49	centroid_8703	16.9639645	3.81E-05	centroid_8703	4.09E-06
centroid_13614	putative membrane protein	0	57	19	50	centroid_1361	16.3949299	5.14E-05	centroid_1361	4.30E-06
centroid_16888	conserved hypothetical protein	0	57	19	50	centroid_1688	16.3949299	5.14E-05	centroid_1688	4.30E-06
centroid_7913	hypothetical protein	0	57	19	50	centroid_7913	16.3949299	5.14E-05	centroid_7913	4.30E-06
centroid_2647	sulfite exporter TauE/SafE family protein	0	55	19	52	centroid_2647	15.3050624	9.15E-05	centroid_2647	6.11E-06
centroid_2779	conserved hypothetical protein	0	55	19	52	centroid_2779	15.3050624	9.15E-05	centroid_2779	6.11E-06

Table S5. Distribution by Phylogroup E

Gene_ID	Annotation	Phylogroup E_present	Other_present	Phylogroup E_Absent	Other_Absent	Gene_ID	chisq-stats	pvalues	Gene_ID	pvalues
centroid_14354	conserved hypothetical protein	5	0	0	120	centroid_1435	100.31467	1.30E-23	centroid_1435	4.26E-09
centroid_14355	leucine rich repeat family protein	5	0	0	120	centroid_1435	100.31467	1.30E-23	centroid_1435	4.26E-09
centroid_17760	type III restriction enzyme, res subunit	5	0	0	120	centroid_1776	100.31467	1.30E-23	centroid_1776	4.26E-09
centroid_17761	helicase conserved C-terminal domain protein	5	0	0	120	centroid_1776	100.31467	1.30E-23	centroid_1776	4.26E-09
centroid_17762	TP-dependent DNA helicase, RecQ family domain protei	5	0	0	120	centroid_1776	100.31467	1.30E-23	centroid_1776	4.26E-09
centroid_17795	leucine rich repeat family protein	5	0	0	120	centroid_1779	100.31467	1.30E-23	centroid_1779	4.26E-09
centroid_17835	putative z1097 gene product	5	0	0	120	centroid_1783	100.31467	1.30E-23	centroid_1783	4.26E-09
centroid_8767	conserved hypothetical protein	5	0	0	120	centroid_8767	100.31467	1.30E-23	centroid_8767	4.26E-09
centroid_8785	conserved hypothetical protein	5	0	0	120	centroid_8785	100.31467	1.30E-23	centroid_8785	4.26E-09
centroid_8801	hypothetical protein	5	0	0	120	centroid_8801	100.31467	1.30E-23	centroid_8801	4.26E-09
centroid_8802	conserved hypothetical protein	5	0	0	120	centroid_8802	100.31467	1.30E-23	centroid_8802	4.26E-09
centroid_8834	ATP-dependent DNA helicase, RecQ family protein	5	0	0	120	centroid_8834	100.31467	1.30E-23	centroid_8834	4.26E-09
centroid_8835	DNA recombination-mediator A family protein	5	0	0	120	centroid_8835	100.31467	1.30E-23	centroid_8835	4.26E-09
centroid_8895	conserved hypothetical protein	5	0	0	120	centroid_8895	100.31467	1.30E-23	centroid_8895	4.26E-09
centroid_8896	tonB-dependent Receptor Plug domain protein	5	0	0	120	centroid_8896	100.31467	1.30E-23	centroid_8896	4.26E-09
centroid_8897	olinate phosphoribosyl transferase, C-terminal domain pr	5	0	0	120	centroid_8897	100.31467	1.30E-23	centroid_8897	4.26E-09
centroid_8898	methyltransferase domain protein	5	0	0	120	centroid_8898	100.31467	1.30E-23	centroid_8898	4.26E-09
centroid_8899	ABC transporter family protein	5	0	0	120	centroid_8899	100.31467	1.30E-23	centroid_8899	4.26E-09
centroid_8900	fecCD transport family protein	5	0	0	120	centroid_8900	100.31467	1.30E-23	centroid_8900	4.26E-09
centroid_8901	periplasmic binding family protein	5	0	0	120	centroid_8901	100.31467	1.30E-23	centroid_8901	4.26E-09
centroid_8958	type VII secretion system (T7SS), usher family protein	5	0	0	120	centroid_8958	100.31467	1.30E-23	centroid_8958	4.26E-09
centroid_8959	fimbrial family protein	5	0	0	120	centroid_8959	100.31467	1.30E-23	centroid_8959	4.26E-09
centroid_9082	fimbrial family protein	5	0	0	120	centroid_9082	100.31467	1.30E-23	centroid_9082	4.26E-09
centroid_9084	type VII secretion system (T7SS), usher family protein	5	0	0	120	centroid_9084	100.31467	1.30E-23	centroid_9084	4.26E-09
centroid_9085	fimbrial family protein	5	0	0	120	centroid_9085	100.31467	1.30E-23	centroid_9085	4.26E-09
centroid_9086	fimbrial family protein	5	0	0	120	centroid_9086	100.31467	1.30E-23	centroid_9086	4.26E-09
centroid_9252	major Facilitator Superfamily protein	5	0	0	120	centroid_9252	100.31467	1.30E-23	centroid_9252	4.26E-09
centroid_9262	type VII secretion system (T7SS), usher family protein	5	0	0	120	centroid_9262	100.31467	1.30E-23	centroid_9262	4.26E-09
centroid_9263	type VII secretion system (T7SS), usher family protein	5	0	0	120	centroid_9263	100.31467	1.30E-23	centroid_9263	4.26E-09
centroid_9457	-negative pili assembly chaperone, N-terminal domain pr	5	0	0	120	centroid_9457	100.31467	1.30E-23	centroid_9457	4.26E-09
centroid_12420	conserved hypothetical protein	5	2	0	118	centroid_1242	70.1816611	5.41E-17	centroid_1242	8.95E-08
centroid_8836	PBP superfamily domain protein	5	2	0	118	centroid_8836	70.1816611	5.41E-17	centroid_8836	8.95E-08
centroid_8961	conserved hypothetical protein	5	2	0	118	centroid_8961	70.1816611	5.41E-17	centroid_8961	8.95E-08
centroid_11114	vitamin B12 transporter BtuB	5	7	0	113	centroid_1111	38.7945935	4.71E-10	centroid_1111	3.38E-06
centroid_6297	maltoporin periplasmic N-terminal extension family protein	5	7	0	113	centroid_6297	38.7945935	4.71E-10	centroid_6297	3.38E-06
centroid_6298	tonB-dependent vitamin B12 receptor	5	7	0	113	centroid_6298	38.7945935	4.71E-10	centroid_6298	3.38E-06
centroid_7241	alpha/beta hydrolase family protein	5	7	0	113	centroid_7241	38.7945935	4.71E-10	centroid_7241	3.38E-06
centroid_7242	NAD dependent epimerase/dehydratase family protein	5	7	0	113	centroid_7242	38.7945935	4.71E-10	centroid_7242	3.38E-06
centroid_9337	putative carboxymethylenebutenolidase	5	7	0	113	centroid_9337	38.7945935	4.71E-10	centroid_9337	3.38E-06
centroid_9338	X-Pro dipeptidyl-peptidase family protein	5	7	0	113	centroid_9338	38.7945935	4.71E-10	centroid_9338	3.38E-06
centroid_9079	(Glycoside-Pentoside-Hexuronide) transporter domain p	5	8	0	112	centroid_9079	35.4146992	2.66E-09	centroid_9079	5.49E-06
centroid_9080	helix-turn-helix domain protein	5	8	0	112	centroid_9080	35.4146992	2.66E-09	centroid_9080	5.49E-06
centroid_9576	conserved hypothetical protein	5	8	0	112	centroid_9576	35.4146992	2.66E-09	centroid_9576	5.49E-06
centroid_10820	zinc-binding dehydrogenase family protein	5	10	0	110	centroid_1082	30.0071023	4.30E-08	centroid_1082	1.28E-05
centroid_5659	conserved hypothetical protein	5	10	0	110	centroid_5659	30.0071023	4.30E-08	centroid_5659	1.28E-05
centroid_8474	putative sor-operon regulator	5	10	0	110	centroid_8474	30.0071023	4.30E-08	centroid_8474	1.28E-05
centroid_8475	short chain dehydrogenase family protein	5	10	0	110	centroid_8475	30.0071023	4.30E-08	centroid_8475	1.28E-05
centroid_8476	m, mannose/fructose/sorbose family, IIA component dom	5	10	0	110	centroid_8476	30.0071023	4.30E-08	centroid_8476	1.28E-05
centroid_8477	rbose-specific phosphotransferase enzyme IIB compone	5	10	0	110	centroid_8477	30.0071023	4.30E-08	centroid_8477	1.28E-05
centroid_8478	stem, mannose/fructose/sorbose , IIC component family	5	10	0	110	centroid_8478	30.0071023	4.30E-08	centroid_8478	1.28E-05
centroid_8479	stem, mannose/fructose/sorbose , IID component family	5	10	0	110	centroid_8479	30.0071023	4.30E-08	centroid_8479	1.28E-05
centroid_8480	zinc-binding dehydrogenase family protein	5	10	0	110	centroid_8480	30.0071023	4.30E-08	centroid_8480	1.28E-05
centroid_8976	conserved hypothetical protein	5	10	0	110	centroid_8976	30.0071023	4.30E-08	centroid_8976	1.28E-05
centroid_9043	putative sugar-binding domain protein	5	10	0	110	centroid_9043	30.0071023	4.30E-08	centroid_9043	1.28E-05
centroid_9044	putative sugar-binding domain protein	5	10	0	110	centroid_9044	30.0071023	4.30E-08	centroid_9044	1.28E-05
centroid_16938	bacterial Ig-like domain family protein	5	12	0	108	centroid_1693	25.8721689	3.65E-07	centroid_1693	2.64E-05
centroid_17334	bacterial Ig-like domain family protein	5	13	0	107	centroid_1733	24.1493867	8.91E-07	centroid_1733	3.65E-05
centroid_6607	bacterial Ig-like domain family protein	5	13	0	107	centroid_6607	24.1493867	8.91E-07	centroid_6607	3.65E-05
centroid_8902	conserved hypothetical protein	5	15	0	105	centroid_8902	21.2208581	4.09E-06	centroid_8902	6.61E-05
centroid_13157	bacterial Ig-like domain family protein	5	16	0	104	centroid_1315	19.965874	7.88E-06	centroid_1315	8.68E-05
centroid_8095	shET2 enterotoxin, N-terminal region family protein	5	18	0	102	centroid_8095	17.7834834	2.48E-05	centroid_8095	0.00014347
centroid_9045	shET2 enterotoxin, N-terminal region family protein	5	18	0	102	centroid_9045	17.7834834	2.48E-05	centroid_9045	0.00014347
centroid_13367	type IV leader peptidase family protein	5	19	0	101	centroid_1336	16.8287825	4.09E-05	centroid_1336	0.00018123
centroid_13677	shET2 enterotoxin, N-terminal region family protein	5	19	0	101	centroid_1367	16.8287825	4.09E-05	centroid_1367	0.00018123
centroid_5812	conserved hypothetical protein	5	19	0	101	centroid_5812	16.8287825	4.09E-05	centroid_5812	0.00018123
centroid_5955	BFD-like [2Fe-2S] binding domain protein	5	19	0	101	centroid_5955	16.8287825	4.09E-05	centroid_5955	0.00018123
centroid_5956	bacterioferritin	5	19	0	101	centroid_5956	16.8287825	4.09E-05	centroid_5956	0.00018123
centroid_8094	ankyrin repeat A domain protein	5	19	0	101	centroid_8094	16.8287825	4.09E-05	centroid_8094	0.00018123
centroid_9373	putative ankyrin repeat A domain protein	5	19	0	101	centroid_9373	16.8287825	4.09E-05	centroid_9373	0.00018123
centroid_9374	putative type III effector domain protein	5	19	0	101	centroid_9374	16.8287825	4.09E-05	centroid_9374	0.00018123
centroid_11151	conserved hypothetical protein	5	21	0	99	centroid_1115	15.1398804	9.98E-05	centroid_1115	0.00028047
centroid_355	fimbrial family protein	5	21	0	99	centroid_355	15.1398804	9.98E-05	centroid_355	0.00028047
centroid_9480	conserved hypothetical protein	5	21	0	99	centroid_9480	15.1398804	9.98E-05	centroid_9480	0.00028047
centroid_13491	haemagglutinin family protein	4	0	1	120	centroid_1349	75.0285167	4.64E-18	centroid_1349	5.16E-07
centroid_14343	conserved hypothetical protein	4	0	1	120	centroid_1434	75.0285167	4.64E-18	centroid_1434	5.16E-07
centroid_17758	conserved hypothetical protein	4	0	1	120	centroid_1775	75.0285167	4.64E-18	centroid_1775	5.16E-07
centroid_17794	invasion plasmid antigen domain protein	4	0	1	120	centroid_1779	75.0285167	4.64E-18	centroid_1779	5.16E-07
centroid_17812	repair family protein	4	0	1	120	centroid_1781	75.0285167	4.64E-18	centroid_1781	5.16E-07
centroid_17813	conserved hypothetical protein	4	0	1	120	centroid_1781	75.0285167	4.64E-18	centroid_1781	5.16E-07
centroid_17969	marB family protein	4	0	1	120	centroid_1796	75.0285167	4.64E-18	centroid_1796	5.16E-07
centroid_8790	fimbrial family protein	4	0	1	120	centroid_8790	75.0285167	4.64E-18	centroid_8790	5.16E-07
centroid_8791	-negative pili assembly chaperone, N-terminal domain pr	4	0	1	120	centroid_8791	75.0285167	4.64E-18	centroid_8791	5.16E-07

centroid_8792	outer membrane usher protein HtrE	4	0	1	120	centroid_8792	75.0285167	4.64E-18	centroid_8792	5.16E-07
centroid_8793	fimbrial family protein	4	0	1	120	centroid_8793	75.0285167	4.64E-18	centroid_8793	5.16E-07
centroid_8795	conserved hypothetical protein	4	0	1	120	centroid_8795	75.0285167	4.64E-18	centroid_8795	5.16E-07
centroid_8845	repair family protein	4	0	1	120	centroid_8845	75.0285167	4.64E-18	centroid_8845	5.16E-07
centroid_8846	repair family protein	4	0	1	120	centroid_8846	75.0285167	4.64E-18	centroid_8846	5.16E-07
centroid_8847	repair family protein	4	0	1	120	centroid_8847	75.0285167	4.64E-18	centroid_8847	5.16E-07
centroid_8975	conserved hypothetical protein	4	0	1	120	centroid_8975	75.0285167	4.64E-18	centroid_8975	5.16E-07
centroid_8988	conserved hypothetical protein	4	0	1	120	centroid_8988	75.0285167	4.64E-18	centroid_8988	5.16E-07
centroid_8989	conserved hypothetical protein	4	0	1	120	centroid_8989	75.0285167	4.64E-18	centroid_8989	5.16E-07
centroid_9083	n-negative pili assembly chaperone, C-terminal domain pr	4	0	1	120	centroid_9083	75.0285167	4.64E-18	centroid_9083	5.16E-07
centroid_9243	pentapeptide repeats family protein	4	0	1	120	centroid_9243	75.0285167	4.64E-18	centroid_9243	5.16E-07
centroid_9253	bacterial regulatory helix-turn-helix , lysR family protein	4	0	1	120	centroid_9253	75.0285167	4.64E-18	centroid_9253	5.16E-07
centroid_9450	conserved hypothetical protein	4	0	1	120	centroid_9450	75.0285167	4.64E-18	centroid_9450	5.16E-07
centroid_9451	conserved hypothetical protein	4	0	1	120	centroid_9451	75.0285167	4.64E-18	centroid_9451	5.16E-07
centroid_9458	n-negative pili assembly chaperone, C-terminal domain pr	4	0	1	120	centroid_9458	75.0285167	4.64E-18	centroid_9458	5.16E-07
centroid_9467	haemagglutinin family protein	4	0	1	120	centroid_9467	75.0285167	4.64E-18	centroid_9467	5.16E-07
centroid_9556	tonB-dependent siderophore receptor family protein	4	0	1	120	centroid_9556	75.0285167	4.64E-18	centroid_9556	5.16E-07
centroid_9557	ABC transporter family protein	4	0	1	120	centroid_9557	75.0285167	4.64E-18	centroid_9557	5.16E-07
centroid_9558	fecCD transport family protein	4	0	1	120	centroid_9558	75.0285167	4.64E-18	centroid_9558	5.16E-07
centroid_9559	fecCD transport family protein	4	0	1	120	centroid_9559	75.0285167	4.64E-18	centroid_9559	5.16E-07
centroid_9560	periplasmic binding family protein	4	0	1	120	centroid_9560	75.0285167	4.64E-18	centroid_9560	5.16E-07
centroid_9561	SIS domain protein	4	0	1	120	centroid_9561	75.0285167	4.64E-18	centroid_9561	5.16E-07
centroid_8947	outer membrane protein C	4	1	1	119	centroid_8947	59.0820313	1.51E-14	centroid_8947	2.56E-06
centroid_8833	tetratricopeptide repeat family protein	4	2	1	118	centroid_8833	48.4524539	3.38E-12	centroid_8833	7.64E-06
centroid_9447	conserved hypothetical protein	4	2	1	118	centroid_9447	48.4524539	3.38E-12	centroid_9447	7.64E-06
centroid_14726	conserved hypothetical protein	4	6	1	114	centroid_1472	27.2022192	1.83E-07	centroid_1472	0.00010405
centroid_9033	conserved hypothetical protein	4	6	1	114	centroid_9033	27.2022192	1.83E-07	centroid_9033	0.00010405
centroid_9711	conserved hypothetical protein	4	6	1	114	centroid_9711	27.2022192	1.83E-07	centroid_9711	0.00010405
centroid_9739	transposase family protein	4	6	1	114	centroid_9739	27.2022192	1.83E-07	centroid_9739	0.00010405
centroid_18640	transposase family protein	4	7	1	113	centroid_1864	24.3065939	8.22E-07	centroid_1864	0.00016237
centroid_8361	putative transposase	4	7	1	113	centroid_8361	24.3065939	8.22E-07	centroid_8361	0.00016237
centroid_9246	conserved hypothetical protein	4	8	1	112	centroid_9246	21.8943968	2.88E-06	centroid_9246	0.00024187
centroid_18149	acetyltransferase domain protein	4	9	1	111	centroid_1814	19.854088	8.36E-06	centroid_1814	0.00034693
centroid_18234	lemA family protein	4	10	1	110	centroid_1823	18.1059966	2.09E-05	centroid_1823	0.00048229
centroid_7244	acyl transferase domain protein	4	10	1	110	centroid_7244	18.1059966	2.09E-05	centroid_7244	0.00048229
centroid_14456	conserved hypothetical protein	4	11	1	109	centroid_1445	16.5916982	4.64E-05	centroid_1445	0.00065302
centroid_15266	conserved hypothetical protein	4	11	1	109	centroid_1526	16.5916982	4.64E-05	centroid_1526	0.00065302
centroid_6478	nucleotide sugar dehydrogenase family protein	4	11	1	109	centroid_6478	16.5916982	4.64E-05	centroid_6478	0.00065302
centroid_6768	enterobacterial Ail/Lom family protein	4	11	1	109	centroid_6768	16.5916982	4.64E-05	centroid_6768	0.00065302
centroid_14323	caudovirales tail fibre assembly family protein	4	12	1	108	centroid_1432	15.267375	9.33E-05	centroid_1432	0.00086448
centroid_9413	bacterial Ig-like domain family protein	4	12	1	108	centroid_9413	15.267375	9.33E-05	centroid_9413	0.00086448
centroid_14327	fimbrial family protein	3	0	2	120	centroid_1432	50.3792407	1.27E-12	centroid_1432	3.15E-05
centroid_14367	conserved hypothetical protein	3	0	2	120	centroid_1436	50.3792407	1.27E-12	centroid_1436	3.15E-05
centroid_14415	conserved hypothetical protein	3	0	2	120	centroid_1441	50.3792407	1.27E-12	centroid_1441	3.15E-05
centroid_14416	hypothetical protein	3	0	2	120	centroid_1441	50.3792407	1.27E-12	centroid_1441	3.15E-05
centroid_14432	ype III secretion outer membrane pore, YscC/HrcC family	3	0	2	120	centroid_1443	50.3792407	1.27E-12	centroid_1443	3.15E-05
centroid_17747	HAMP domain protein	3	0	2	120	centroid_1774	50.3792407	1.27E-12	centroid_1774	3.15E-05
centroid_17757	conserved hypothetical protein	3	0	2	120	centroid_1775	50.3792407	1.27E-12	centroid_1775	3.15E-05
centroid_18012	ive non-LEE-encoded type III secreted effector domain pr	3	0	2	120	centroid_1801	50.3792407	1.27E-12	centroid_1801	3.15E-05
centroid_18123	conserved hypothetical protein	3	0	2	120	centroid_1812	50.3792407	1.27E-12	centroid_1812	3.15E-05
centroid_18144	sopA-like catalytic domain protein	3	0	2	120	centroid_1814	50.3792407	1.27E-12	centroid_1814	3.15E-05
centroid_18145	sopA-like central domain protein	3	0	2	120	centroid_1814	50.3792407	1.27E-12	centroid_1814	3.15E-05
centroid_18251	putative ipaH-like protein	3	0	2	120	centroid_1825	50.3792407	1.27E-12	centroid_1825	3.15E-05
centroid_18252	leucine Rich Repeat family protein	3	0	2	120	centroid_1825	50.3792407	1.27E-12	centroid_1825	3.15E-05
centroid_18253	on-LEE-encoded type III secreted effector domain protein	3	0	2	120	centroid_1825	50.3792407	1.27E-12	centroid_1825	3.15E-05
centroid_8797	(Glycoside-Pentoside-Hexuronide) transporter domain p	3	0	2	120	centroid_8797	50.3792407	1.27E-12	centroid_8797	3.15E-05
centroid_8798	glycosyl hydrolases 43 family protein	3	0	2	120	centroid_8798	50.3792407	1.27E-12	centroid_8798	3.15E-05
centroid_8807	hemin import ATP-binding protein HmuV	3	0	2	120	centroid_8807	50.3792407	1.27E-12	centroid_8807	3.15E-05
centroid_8808	fecCD transport family protein	3	0	2	120	centroid_8808	50.3792407	1.27E-12	centroid_8808	3.15E-05
centroid_8809	NADH(P)-binding family protein	3	0	2	120	centroid_8809	50.3792407	1.27E-12	centroid_8809	3.15E-05
centroid_8810	conserved hypothetical protein	3	0	2	120	centroid_8810	50.3792407	1.27E-12	centroid_8810	3.15E-05
centroid_8811	putative heme utilization radical SAM enzyme HutW	3	0	2	120	centroid_8811	50.3792407	1.27E-12	centroid_8811	3.15E-05
centroid_8812	periplasmic binding family protein	3	0	2	120	centroid_8812	50.3792407	1.27E-12	centroid_8812	3.15E-05
centroid_8813	tonB-dependent heme/hemoglobin receptor family protein	3	0	2	120	centroid_8813	50.3792407	1.27E-12	centroid_8813	3.15E-05
centroid_8814	hemin transport protein HemS	3	0	2	120	centroid_8814	50.3792407	1.27E-12	centroid_8814	3.15E-05
centroid_8857	ype III secretion outer membrane pore, YscC/HrcC family	3	0	2	120	centroid_8857	50.3792407	1.27E-12	centroid_8857	3.15E-05
centroid_8858	invasion protein InvF	3	0	2	120	centroid_8858	50.3792407	1.27E-12	centroid_8858	3.15E-05
centroid_8931	conserved hypothetical protein	3	0	2	120	centroid_8931	50.3792407	1.27E-12	centroid_8931	3.15E-05
centroid_8932	conserved hypothetical protein	3	0	2	120	centroid_8932	50.3792407	1.27E-12	centroid_8932	3.15E-05
centroid_8933	methylaspartate mutase, S subunit	3	0	2	120	centroid_8933	50.3792407	1.27E-12	centroid_8933	3.15E-05
centroid_8934	conserved hypothetical family protein	3	0	2	120	centroid_8934	50.3792407	1.27E-12	centroid_8934	3.15E-05
centroid_8935	methylaspartate mutase, E subunit	3	0	2	120	centroid_8935	50.3792407	1.27E-12	centroid_8935	3.15E-05
centroid_8936	methylaspartate ammonia-lyase	3	0	2	120	centroid_8936	50.3792407	1.27E-12	centroid_8936	3.15E-05
centroid_8937	conserved hypothetical protein	3	0	2	120	centroid_8937	50.3792407	1.27E-12	centroid_8937	3.15E-05
centroid_8957	n-negative pili assembly chaperone, N-terminal domain pr	3	0	2	120	centroid_8957	50.3792407	1.27E-12	centroid_8957	3.15E-05
centroid_8967	ABC transporter family protein	3	0	2	120	centroid_8967	50.3792407	1.27E-12	centroid_8967	3.15E-05
centroid_8968	-dependent transport system inner membrane componer	3	0	2	120	centroid_8968	50.3792407	1.27E-12	centroid_8968	3.15E-05
centroid_8969	conserved hypothetical protein	3	0	2	120	centroid_8969	50.3792407	1.27E-12	centroid_8969	3.15E-05
centroid_8970	major Facilitator Superfamily protein	3	0	2	120	centroid_8970	50.3792407	1.27E-12	centroid_8970	3.15E-05
centroid_8971	MASE1 family protein	3	0	2	120	centroid_8971	50.3792407	1.27E-12	centroid_8971	3.15E-05
centroid_8972	response regulator	3	0	2	120	centroid_8972	50.3792407	1.27E-12	centroid_8972	3.15E-05
centroid_8974	conserved hypothetical protein	3	0	2	120	centroid_8974	50.3792407	1.27E-12	centroid_8974	3.15E-05
centroid_8977	conserved hypothetical protein	3	0	2	120	centroid_8977	50.3792407	1.27E-12	centroid_8977	3.15E-05
centroid_8978	conserved hypothetical protein	3	0	2	120	centroid_8978	50.3792407	1.27E-12	centroid_8978	3.15E-05
centroid_8980	pentapeptide repeats family protein	3	0	2	120	centroid_8980	50.3792407	1.27E-12	centroid_8980	3.15E-05
centroid_8984	fimbrial family protein	3	0	2	120	centroid_8984	50.3792407	1.27E-12	centroid_8984	3.15E-05
centroid_8987	amidohydrolase family protein	3	0	2	120	centroid_8987	50.3792407	1.27E-12	centroid_8987	3.15E-05
centroid_8990	conserved hypothetical protein	3	0	2	120	centroid_8990	50.3792407	1.27E-12	centroid_8990	3.15E-05
centroid_9008	NADH:flavin oxidoreductase / NADH oxidase family protei	3	0	2	120	centroid_9008	50.3792407	1.27E-12	centroid_9008	3.15E-05
centroid_9009	alpha/beta hydrolase family protein	3	0	2	120	centroid_9009	50.3792407	1.27E-12	centroid_9009	3.15E-05
centroid_9010	lysR substrate binding domain protein	3	0	2	120	centroid_9010	50.3792407	1.27E-12	centroid_9010	3.15E-05
centroid_9011	lysR substrate binding domain protein	3	0	2	120	centroid_9011	50.3792407	1.27E-12	centroid_9011	3.15E-05
centroid_9012	aldo/keto reductase family protein	3	0	2	120	centroid_9012	50.3792407	1.27E-12	centroid_9012	3.15E-05
centroid_9013	aldo/keto reductase family protein	3	0	2	120	centroid_9013	50.3792407	1.27E-12	centroid_9013	3.15E-05
centroid_9107	type I secretion outer membrane , TolC family protein	3	0	2	120	centroid_9107	50.3792407	1.27E-12	centroid_9107	3.15E-05
centroid_9109	type I secretion system ATPase family protein	3	0	2	120	centroid_9109	50.3792407	1.27E-12	centroid_9109	3.15E-05
centroid_9110	type I secretion membrane fusion , HlyD family protein	3	0	2	120	centroid_9110	50.3792407	1.27E-12	centroid_9110	3.15E-05
centroid_9111	conserved hypothetical protein	3	0	2	120	centroid_9111	50.3792407	1.27E-12	centroid_9111	3.15E-05
centroid_9112	putative lipoprotein	3	0	2	120	centroid_9112	50.3792407	1.27E-12	centroid_9112	3.15E-05
centroid_9113	yadA-like C-terminal region family protein	3	0	2	120	centroid_9113	50.3792407	1.27E-12	centroid_9113	3.15E-05
centroid_9114	conserved hypothetical protein	3	0	2	120	centroid_9114	50.3792407	1.27E-12	centroid_9114	3.15E-05
centroid_9119	outer membrane porin protein OmpD	3	0	2	120	centroid_9119	50.3792407	1.27E-12	centroid_9119	3.15E-05
centroid_9120	leucine Rich Repeat family protein	3	0	2	120	centroid_9120	50.3792407	1.27E-12	centroid_9120	3.15E-05
centroid_9134	transcriptional regulatory , C terminal family protein	3	0	2	120	centroid_9134	50.3792407	1.27E-12	centroid_9134	3.15E-05
centroid_9135	pfkB carbohydrate kinase family protein	3	0	2	120	centroid_9135	50.3792407	1.27E-12	centroid_9135	3.15E-05
centroid_9136	ketose-bisphosphate aldolase family protein	3	0	2	120	centroid_9136	50.3792407	1.27E-12	centroid_9136	3.15E-05
centroid_9137	conserved hypothetical protein	3	0	2	120	centroid_9137	50.3792407	1.27E-12	centroid_9137	3.15E-05

centroid_9138	lasmic binding and sugar binding domain of LacI family pr	3	0	2	120	centroid_9138	50.3792407	1.27E-12	centroid_9138	3.15E-05
centroid_9139	ain amino acid transport system / permease component f	3	0	2	120	centroid_9139	50.3792407	1.27E-12	centroid_9139	3.15E-05
centroid_9140	ABC transporter family protein	3	0	2	120	centroid_9140	50.3792407	1.27E-12	centroid_9140	3.15E-05
centroid_9206	conserved hypothetical protein	3	0	2	120	centroid_9206	50.3792407	1.27E-12	centroid_9206	3.15E-05
centroid_9215	conserved hypothetical protein	3	0	2	120	centroid_9215	50.3792407	1.27E-12	centroid_9215	3.15E-05
centroid_9216	conserved hypothetical protein	3	0	2	120	centroid_9216	50.3792407	1.27E-12	centroid_9216	3.15E-05
centroid_9258	conserved hypothetical protein	3	0	2	120	centroid_9258	50.3792407	1.27E-12	centroid_9258	3.15E-05
centroid_9259	fimbrial family protein	3	0	2	120	centroid_9259	50.3792407	1.27E-12	centroid_9259	3.15E-05
centroid_9260	fimbrial family protein	3	0	2	120	centroid_9260	50.3792407	1.27E-12	centroid_9260	3.15E-05
centroid_9261	ı-negative pili assembly chaperone, N-terminal domain pr	3	0	2	120	centroid_9261	50.3792407	1.27E-12	centroid_9261	3.15E-05
centroid_9274	fimbrial family protein	3	0	2	120	centroid_9274	50.3792407	1.27E-12	centroid_9274	3.15E-05
centroid_9341	conserved hypothetical protein	3	0	2	120	centroid_9341	50.3792407	1.27E-12	centroid_9341	3.15E-05
centroid_9342	conserved hypothetical protein	3	0	2	120	centroid_9342	50.3792407	1.27E-12	centroid_9342	3.15E-05
centroid_9343	helix-turn-helix family protein	3	0	2	120	centroid_9343	50.3792407	1.27E-12	centroid_9343	3.15E-05
centroid_9416	glycosyl transferases group 1 family protein	3	0	2	120	centroid_9416	50.3792407	1.27E-12	centroid_9416	3.15E-05
centroid_9417	polysaccharide biosynthesis family protein	3	0	2	120	centroid_9417	50.3792407	1.27E-12	centroid_9417	3.15E-05
centroid_9419	transposase DDE domain protein	3	0	2	120	centroid_9419	50.3792407	1.27E-12	centroid_9419	3.15E-05
centroid_9420	putative h repeat-associated protein YhhI	3	0	2	120	centroid_9420	50.3792407	1.27E-12	centroid_9420	3.15E-05
centroid_9493	papC N-terminal domain protein	3	0	2	120	centroid_9493	50.3792407	1.27E-12	centroid_9493	3.15E-05
centroid_9494	papC N-terminal domain protein	3	0	2	120	centroid_9494	50.3792407	1.27E-12	centroid_9494	3.15E-05
centroid_9495	ı-negative pili assembly chaperone, N-terminal domain pr	3	0	2	120	centroid_9495	50.3792407	1.27E-12	centroid_9495	3.15E-05
centroid_9496	fimbrial family protein	3	0	2	120	centroid_9496	50.3792407	1.27E-12	centroid_9496	3.15E-05
centroid_9565	CFA/III pilin	3	0	2	120	centroid_9565	50.3792407	1.27E-12	centroid_9565	3.15E-05
centroid_9577	fimbrial family protein	3	0	2	120	centroid_9577	50.3792407	1.27E-12	centroid_9577	3.15E-05
centroid_9578	type VII secretion system (T7SS), usher family protein	3	0	2	120	centroid_9578	50.3792407	1.27E-12	centroid_9578	3.15E-05
centroid_9637	1-phosphate guanylyltransferase/mannose-6-phosphate	3	0	2	120	centroid_9637	50.3792407	1.27E-12	centroid_9637	3.15E-05
centroid_9638	glycosyl transferases group 1 family protein	3	0	2	120	centroid_9638	50.3792407	1.27E-12	centroid_9638	3.15E-05
centroid_9639	glycosyl transferases group 1 family protein	3	0	2	120	centroid_9639	50.3792407	1.27E-12	centroid_9639	3.15E-05
centroid_9640	putative membrane protein	3	0	2	120	centroid_9640	50.3792407	1.27E-12	centroid_9640	3.15E-05
centroid_9641	glycosyl transferases group 1 family protein	3	0	2	120	centroid_9641	50.3792407	1.27E-12	centroid_9641	3.15E-05
centroid_9642	glycosyl transferase 2 family protein	3	0	2	120	centroid_9642	50.3792407	1.27E-12	centroid_9642	3.15E-05
centroid_9464	conserved domain protein	3	1	2	119	centroid_9464	36.8268982	1.29E-09	centroid_9464	0.00012434
centroid_12550	conserved hypothetical protein	3	2	2	118	centroid_1255	28.7000868	8.45E-08	centroid_1255	0.000307
centroid_12631	transposase domain protein	3	2	2	118	centroid_1263	28.7000868	8.45E-08	centroid_1263	0.000307
centroid_12667	transposase IS66 family protein	3	2	2	118	centroid_1266	28.7000868	8.45E-08	centroid_1266	0.000307
centroid_13543	transposase domain protein	3	2	2	118	centroid_1354	28.7000868	8.45E-08	centroid_1354	0.000307
centroid_13549	transposase IS66 family protein	3	2	2	118	centroid_1354	28.7000868	8.45E-08	centroid_1354	0.000307
centroid_14473	putative minor structural subunit AalA	3	2	2	118	centroid_1447	28.7000868	8.45E-08	centroid_1447	0.000307
centroid_14475	helix-turn-helix domain protein	3	2	2	118	centroid_1447	28.7000868	8.45E-08	centroid_1447	0.000307
centroid_14506	transposase family protein	3	2	2	118	centroid_1450	28.7000868	8.45E-08	centroid_1450	0.000307
centroid_14507	transposase domain protein	3	2	2	118	centroid_1450	28.7000868	8.45E-08	centroid_1450	0.000307
centroid_8848	conserved hypothetical protein	3	2	2	118	centroid_8848	28.7000868	8.45E-08	centroid_8848	0.000307
centroid_9427	phage tail fibre repeat family protein	3	2	2	118	centroid_9427	28.7000868	8.45E-08	centroid_9427	0.000307
centroid_9562	ıdhesin biosynthesis transcription regulatory family protei	3	2	2	118	centroid_9562	28.7000868	8.45E-08	centroid_9562	0.000307
centroid_9563	bacterial regulatory helix-turn-helix, AraC family protein	3	2	2	118	centroid_9563	28.7000868	8.45E-08	centroid_9563	0.000307
centroid_9564	transglycosylase SLT domain protein	3	2	2	118	centroid_9564	28.7000868	8.45E-08	centroid_9564	0.000307
centroid_9566	repilin-type N-terminal cleavage/methylation domain prote	3	2	2	118	centroid_9566	28.7000868	8.45E-08	centroid_9566	0.000307
centroid_9598	hypothetical protein	3	2	2	118	centroid_9598	28.7000868	8.45E-08	centroid_9598	0.000307
centroid_9621	K88 fimbrial protein AD	3	2	2	118	centroid_9621	28.7000868	8.45E-08	centroid_9621	0.000307
centroid_9622	K88 fimbrial protein AB	3	2	2	118	centroid_9622	28.7000868	8.45E-08	centroid_9622	0.000307
centroid_9623	hypothetical protein	3	2	2	118	centroid_9623	28.7000868	8.45E-08	centroid_9623	0.000307
centroid_9624	fimbrial, major and minor subunit	3	2	2	118	centroid_9624	28.7000868	8.45E-08	centroid_9624	0.000307
centroid_9625	fimbrial, major and minor subunit	3	2	2	118	centroid_9625	28.7000868	8.45E-08	centroid_9625	0.000307
centroid_9626	putative k88 minor fimbrial subunit FaeF	3	2	2	118	centroid_9626	28.7000868	8.45E-08	centroid_9626	0.000307
centroid_9627	chaperone protein FaeE	3	2	2	118	centroid_9627	28.7000868	8.45E-08	centroid_9627	0.000307
centroid_9628	type VII secretion system (T7SS), usher family protein	3	2	2	118	centroid_9628	28.7000868	8.45E-08	centroid_9628	0.000307
centroid_9629	putative minor structural subunit AalA	3	2	2	118	centroid_9629	28.7000868	8.45E-08	centroid_9629	0.000307
centroid_9630	ıdhesin biosynthesis transcription regulatory family protei	3	2	2	118	centroid_9630	28.7000868	8.45E-08	centroid_9630	0.000307
centroid_9678	transposase family protein	3	2	2	118	centroid_9678	28.7000868	8.45E-08	centroid_9678	0.000307
centroid_9709	transposase family protein	3	2	2	118	centroid_9709	28.7000868	8.45E-08	centroid_9709	0.000307
centroid_9716	tn3 transposase DDE domain protein	3	2	2	118	centroid_9716	28.7000868	8.45E-08	centroid_9716	0.000307
centroid_9738	hypothetical protein	3	2	2	118	centroid_9738	28.7000868	8.45E-08	centroid_9738	0.000307
centroid_9747	putative transposase	3	2	2	118	centroid_9747	28.7000868	8.45E-08	centroid_9747	0.000307
centroid_9776	putative iS91 orf	3	2	2	118	centroid_9776	28.7000868	8.45E-08	centroid_9776	0.000307
centroid_10305	methylmalonyl-CoA mutase C-terminal domain protein	3	120	2	0	centroid_1030	26.6821223	2.40E-07	centroid_1030	0.00129032
centroid_10760	papC C-terminal domain protein	3	120	2	0	centroid_1076	26.6821223	2.40E-07	centroid_1076	0.00129032
centroid_10771	PTS system, glucose-like IIB component domain protein	3	120	2	0	centroid_1077	26.6821223	2.40E-07	centroid_1077	0.00129032
centroid_10772	PTS system beta-glucoside-specific EIIBCA component	3	120	2	0	centroid_1077	26.6821223	2.40E-07	centroid_1077	0.00129032
centroid_1151	fimbrial family protein	3	120	2	0	centroid_1151	26.6821223	2.40E-07	centroid_1151	0.00129032
centroid_11623	HokA domain protein	3	120	2	0	centroid_1162	26.6821223	2.40E-07	centroid_1162	0.00129032
centroid_12412	methylmalonyl-CoA mutase	3	120	2	0	centroid_1241	26.6821223	2.40E-07	centroid_1241	0.00129032
centroid_12547	mannosylglycerate hydrolase	3	120	2	0	centroid_1254	26.6821223	2.40E-07	centroid_1254	0.00129032
centroid_12682	ative phosphoethanolamine transferase YhbX domain pro	3	120	2	0	centroid_1268	26.6821223	2.40E-07	centroid_1268	0.00129032
centroid_1298	alpha amylase, catalytic domain protein	3	120	2	0	centroid_1298	26.6821223	2.40E-07	centroid_1298	0.00129032
centroid_13196	type VII secretion system (T7SS), usher family protein	3	120	2	0	centroid_1319	26.6821223	2.40E-07	centroid_1319	0.00129032
centroid_13615	type VII secretion system (T7SS), usher family protein	3	120	2	0	centroid_1361	26.6821223	2.40E-07	centroid_1361	0.00129032
centroid_13893	lamB porin family protein	3	120	2	0	centroid_1389	26.6821223	2.40E-07	centroid_1389	0.00129032
centroid_14001	6-phospho-beta-glucosidase BglB	3	120	2	0	centroid_1400	26.6821223	2.40E-07	centroid_1400	0.00129032
centroid_14201	transposase, YhgA-like family protein	3	120	2	0	centroid_1420	26.6821223	2.40E-07	centroid_1420	0.00129032
centroid_14261	inner membrane CbrB domain protein	3	120	2	0	centroid_1426	26.6821223	2.40E-07	centroid_1426	0.00129032
centroid_14270	type VII secretion system (T7SS), usher family protein	3	120	2	0	centroid_1427	26.6821223	2.40E-07	centroid_1427	0.00129032
centroid_14351	conserved hypothetical protein	3	120	2	0	centroid_1435	26.6821223	2.40E-07	centroid_1435	0.00129032
centroid_14624	SPFH domain / Band 7 family protein	3	120	2	0	centroid_1462	26.6821223	2.40E-07	centroid_1462	0.00129032
centroid_14723	hypothetical protein	3	120	2	0	centroid_1472	26.6821223	2.40E-07	centroid_1472	0.00129032
centroid_14738	conserved hypothetical protein	3	120	2	0	centroid_1473	26.6821223	2.40E-07	centroid_1473	0.00129032
centroid_14806	efflux transporter, RND family, MFP subunit	3	120	2	0	centroid_1480	26.6821223	2.40E-07	centroid_1480	0.00129032
centroid_16093	fimbrial family protein	3	120	2	0	centroid_1609	26.6821223	2.40E-07	centroid_1609	0.00129032
centroid_16439	leucine rich repeat family protein	3	120	2	0	centroid_1643	26.6821223	2.40E-07	centroid_1643	0.00129032
centroid_16929	conserved hypothetical protein	3	120	2	0	centroid_1692	26.6821223	2.40E-07	centroid_1692	0.00129032
centroid_16993	fimbrial family protein	3	120	2	0	centroid_1699	26.6821223	2.40E-07	centroid_1699	0.00129032
centroid_17220	gamma-glutamyltranspeptidase domain protein	3	120	2	0	centroid_1722	26.6821223	2.40E-07	centroid_1722	0.00129032
centroid_17323	bacterial regulatory, tetR family protein	3	120	2	0	centroid_1732	26.6821223	2.40E-07	centroid_1732	0.00129032
centroid_17324	3-type transcriptional repressor, C-terminal region family	3	120	2	0	centroid_1732	26.6821223	2.40E-07	centroid_1732	0.00129032
centroid_17427	beta galactosidase small chain family protein	3	120	2	0	centroid_1742	26.6821223	2.40E-07	centroid_1742	0.00129032
centroid_17456	S system, fructose subfamily, IIA component domain prot	3	120	2	0	centroid_1745	26.6821223	2.40E-07	centroid_1745	0.00129032
centroid_17457	PTS system, Fru family, IIC component domain protein	3	120	2	0	centroid_1745	26.6821223	2.40E-07	centroid_1745	0.00129032
centroid_1997	methylmalonyl-CoA mutase	3	120	2	0	centroid_1997	26.6821223	2.40E-07	centroid_1997	0.00129032
centroid_2003	arginine exporter protein ArgO	3	120	2	0	centroid_2003	26.6821223	2.40E-07	centroid_2003	0.00129032
centroid_2049	cryptic beta-glucoside bgl operon antiterminator	3	120	2	0	centroid_2049	26.6821223	2.40E-07	centroid_2049	0.00129032
centroid_2050	PTS system beta-glucoside-specific EIIBCA component	3	120	2	0	centroid_2050	26.6821223	2.40E-07	centroid_2050	0.00129032
centroid_2054	inner membrane protein CbrB	3	120	2	0	centroid_2054	26.6821223	2.40E-07	centroid_2054	0.00129032
centroid_2482	conserved hypothetical protein	3	120	2	0	centroid_2482	26.6821223	2.40E-07	centroid_2482	0.00129032
centroid_2483	putative acrEF/envCD operon repressor	3	120	2	0	centroid_2483	26.6821223	2.40E-07	centroid_2483	0.00129032
centroid_253	3-methyl-2-oxobutanoate hydroxymethyltransferase	3	120	2	0	centroid_253	26.6821223	2.40E-07	centroid_253	0.00129032
centroid_2543	protein HokA	3	120	2	0	centroid_2543	26.6821223	2.40E-07	centroid_2543	0.00129032
centroid_2858	serine transporter	3	120	2	0	centroid_2858	26.6821223	2.40E-07	centroid_2858	0.00129032
centroid_2959	osyl-D-glycerate transport/metabolism system repressor	3	120	2	0	centroid_2959	26.6821223	2.40E-07	centroid_2959	0.00129032

centroid_2960	heat-responsive suppressor HrsA	3	120	2	0	centroid_296	26.6821223	2.40E-07	centroid_296	0.00129032
centroid_2961	mannosylglycerate hydrolase	3	120	2	0	centroid_296	26.6821223	2.40E-07	centroid_296	0.00129032
centroid_3055	N-acetyltransferase family protein	3	120	2	0	centroid_305	26.6821223	2.40E-07	centroid_305	0.00129032
centroid_3410	ynbE-like lipofamily protein	3	120	2	0	centroid_341	26.6821223	2.40E-07	centroid_341	0.00129032
centroid_3632	regulatory protein SdiA	3	120	2	0	centroid_363	26.6821223	2.40E-07	centroid_363	0.00129032
centroid_3744	putative amino-acid metabolite efflux pump	3	120	2	0	centroid_374	26.6821223	2.40E-07	centroid_374	0.00129032
centroid_3745	marB family protein	3	120	2	0	centroid_374	26.6821223	2.40E-07	centroid_374	0.00129032
centroid_3822	conserved hypothetical protein	3	120	2	0	centroid_382	26.6821223	2.40E-07	centroid_382	0.00129032
centroid_4581	fimbrial family protein	3	120	2	0	centroid_458	26.6821223	2.40E-07	centroid_458	0.00129032
centroid_4655	HokA domain protein	3	120	2	0	centroid_465	26.6821223	2.40E-07	centroid_465	0.00129032
centroid_4810	cyanate hydratase	3	120	2	0	centroid_481	26.6821223	2.40E-07	centroid_481	0.00129032
centroid_511	cyanate transporter family protein	3	120	2	0	centroid_511	26.6821223	2.40E-07	centroid_511	0.00129032
centroid_512	cyanate hydratase	3	120	2	0	centroid_512	26.6821223	2.40E-07	centroid_512	0.00129032
centroid_513	carbonic anhydrase 1	3	120	2	0	centroid_513	26.6821223	2.40E-07	centroid_513	0.00129032
centroid_514	HTH-type transcriptional regulator CynR	3	120	2	0	centroid_514	26.6821223	2.40E-07	centroid_514	0.00129032
centroid_515	cytosine deaminase	3	120	2	0	centroid_515	26.6821223	2.40E-07	centroid_515	0.00129032
centroid_516	cytosine permease	3	120	2	0	centroid_516	26.6821223	2.40E-07	centroid_516	0.00129032
centroid_7318	putative acrEF/envCD operon repressor	3	120	2	0	centroid_731	26.6821223	2.40E-07	centroid_731	0.00129032
centroid_7642	6-phospho-beta-glucosidase BglB	3	120	2	0	centroid_764	26.6821223	2.40E-07	centroid_764	0.00129032
centroid_7716	S system, fructose subfamily, IIA component domain prot	3	120	2	0	centroid_771	26.6821223	2.40E-07	centroid_771	0.00129032
centroid_7717	heat-responsive suppressor HrsA domain protein	3	120	2	0	centroid_771	26.6821223	2.40E-07	centroid_771	0.00129032
centroid_7755	type VII secretion system (T7SS), usher family protein	3	120	2	0	centroid_775	26.6821223	2.40E-07	centroid_775	0.00129032
centroid_8060	PTS system sorbose-specific iic component family protei	3	120	2	0	centroid_806	26.6821223	2.40E-07	centroid_806	0.00129032
centroid_8062	ative phosphoethanolamine transferase YhbX domain pro	3	120	2	0	centroid_806	26.6821223	2.40E-07	centroid_806	0.00129032
centroid_8089	PRD domain protein	3	120	2	0	centroid_808	26.6821223	2.40E-07	centroid_808	0.00129032
centroid_8997	S system, glucose subfamily, IIA component domain prot	3	120	2	0	centroid_899	26.6821223	2.40E-07	centroid_899	0.00129032
centroid_8998	PTS system, glucose-like IIB component domain protein	3	120	2	0	centroid_899	26.6821223	2.40E-07	centroid_899	0.00129032
centroid_9024	DNA methylase family protein	3	120	2	0	centroid_902	26.6821223	2.40E-07	centroid_902	0.00129032
centroid_9028	yltransferase DNA adenine methyltransferase domain pr	3	120	2	0	centroid_902	26.6821223	2.40E-07	centroid_902	0.00129032
centroid_12596	proQ/FINO family protein	3	3	2	117	centroid_1259	23.2861374	1.40E-06	centroid_1259	0.00060636
centroid_14469	proQ/FINO family protein	3	3	2	117	centroid_1446	23.2861374	1.40E-06	centroid_1446	0.00060636
centroid_5722	conserved hypothetical protein	3	3	2	117	centroid_572	23.2861374	1.40E-06	centroid_572	0.00060636
centroid_8330	type IV leader peptidase family protein	3	3	2	117	centroid_833	23.2861374	1.40E-06	centroid_833	0.00060636
centroid_8331	putative cofJ	3	3	2	117	centroid_833	23.2861374	1.40E-06	centroid_833	0.00060636
centroid_8332	type II secretion system (T2SS), F family protein	3	3	2	117	centroid_833	23.2861374	1.40E-06	centroid_833	0.00060636
centroid_8333	type II/IV secretion system family protein	3	3	2	117	centroid_833	23.2861374	1.40E-06	centroid_833	0.00060636
centroid_8334	putative lngG	3	3	2	117	centroid_833	23.2861374	1.40E-06	centroid_833	0.00060636
centroid_8335	putative pilus biosynthesis protein	3	3	2	117	centroid_833	23.2861374	1.40E-06	centroid_833	0.00060636
centroid_8336	putative pilus biosynthesis transmembrane anchor protei	3	3	2	117	centroid_833	23.2861374	1.40E-06	centroid_833	0.00060636
centroid_8337	bacterial type II and III secretion system family protein	3	3	2	117	centroid_833	23.2861374	1.40E-06	centroid_833	0.00060636
centroid_8338	toxin co-regulated pilus biosynthesis Q family protein	3	3	2	117	centroid_833	23.2861374	1.40E-06	centroid_833	0.00060636
centroid_8346	hypothetical protein	3	3	2	117	centroid_834	23.2861374	1.40E-06	centroid_834	0.00060636
centroid_8349	resolvase, N terminal domain protein	3	3	2	117	centroid_834	23.2861374	1.40E-06	centroid_834	0.00060636
centroid_8859	conserved hypothetical protein	3	3	2	117	centroid_885	23.2861374	1.40E-06	centroid_885	0.00060636
centroid_9418	helix-turn-helix domain protein	3	3	2	117	centroid_941	23.2861374	1.40E-06	centroid_941	0.00060636
centroid_9567	proQ/FINO family protein	3	3	2	117	centroid_956	23.2861374	1.40E-06	centroid_956	0.00060636
centroid_9680	hypothetical protein	3	3	2	117	centroid_968	23.2861374	1.40E-06	centroid_968	0.00060636
centroid_9681	ABC transporter family protein	3	3	2	117	centroid_968	23.2861374	1.40E-06	centroid_968	0.00060636
centroid_9682	biotin-lipoyl like family protein	3	3	2	117	centroid_968	23.2861374	1.40E-06	centroid_968	0.00060636
centroid_9683	peptidase S24-like family protein	3	3	2	117	centroid_968	23.2861374	1.40E-06	centroid_968	0.00060636
centroid_9684	impB/mucB/samB family protein	3	3	2	117	centroid_968	23.2861374	1.40E-06	centroid_968	0.00060636
centroid_9686	conserved hypothetical protein	3	3	2	117	centroid_968	23.2861374	1.40E-06	centroid_968	0.00060636
centroid_9687	parB/RepB/Spo0J family partition domain protein	3	3	2	117	centroid_968	23.2861374	1.40E-06	centroid_968	0.00060636
centroid_9689	itoxin Phd_YefM, type II toxin-antitoxin system family prot	3	3	2	117	centroid_968	23.2861374	1.40E-06	centroid_968	0.00060636
centroid_9690	addiction module toxin, RelE/StbE family protein	3	3	2	117	centroid_969	23.2861374	1.40E-06	centroid_969	0.00060636
centroid_9732	conserved hypothetical protein	3	3	2	117	centroid_973	23.2861374	1.40E-06	centroid_973	0.00060636
centroid_9745	transposase family protein	3	3	2	117	centroid_974	23.2861374	1.40E-06	centroid_974	0.00060636
centroid_11096	putative type III secretion protein	3	4	2	116	centroid_1109	19.4224803	1.05E-05	centroid_1109	0.00104786
centroid_7198	UTRA domain protein	3	4	2	116	centroid_719	19.4224803	1.05E-05	centroid_719	0.00104786
centroid_7199	uvate-dependent sugar phosphotransferase system, EIIA	3	4	2	116	centroid_719	19.4224803	1.05E-05	centroid_719	0.00104786
centroid_7200	PTS system, Lactose/Cellobiose specific IIB subunit	3	4	2	116	centroid_720	19.4224803	1.05E-05	centroid_720	0.00104786
centroid_7201	S system sugar-specific permease component family pro	3	4	2	116	centroid_720	19.4224803	1.05E-05	centroid_720	0.00104786
centroid_7202	GY family of carbohydrate kinase, N-terminal domain pro	3	4	2	116	centroid_720	19.4224803	1.05E-05	centroid_720	0.00104786
centroid_7203	phosphotransferase system, HPr-related proteins	3	4	2	116	centroid_720	19.4224803	1.05E-05	centroid_720	0.00104786
centroid_7204	ketose-bisphosphate aldolase family protein	3	4	2	116	centroid_720	19.4224803	1.05E-05	centroid_720	0.00104786
centroid_8352	hypothetical protein	3	4	2	116	centroid_835	19.4224803	1.05E-05	centroid_835	0.00104786
centroid_8353	hypothetical protein	3	4	2	116	centroid_835	19.4224803	1.05E-05	centroid_835	0.00104786
centroid_8438	caudovirales tail fibre assembly family protein	3	4	2	116	centroid_843	19.4224803	1.05E-05	centroid_843	0.00104786
centroid_9444	PTS family galactitol porter, component IIC domain protei	3	4	2	116	centroid_944	19.4224803	1.05E-05	centroid_944	0.00104786
centroid_9445	S system sugar-specific permease component family pro	3	4	2	116	centroid_944	19.4224803	1.05E-05	centroid_944	0.00104786
centroid_9446	conserved hypothetical protein	3	4	2	116	centroid_944	19.4224803	1.05E-05	centroid_944	0.00104786
centroid_9685	conserved hypothetical protein	3	4	2	116	centroid_968	19.4224803	1.05E-05	centroid_968	0.00104786
centroid_9688	cobQ/CobB/MinD/ParA nucleotide binding domain protein	3	4	2	116	centroid_968	19.4224803	1.05E-05	centroid_968	0.00104786
centroid_9721	type I restriction enzyme R N terminus family protein	3	4	2	116	centroid_972	19.4224803	1.05E-05	centroid_972	0.00104786
centroid_9731	conserved hypothetical protein	3	4	2	116	centroid_973	19.4224803	1.05E-05	centroid_973	0.00104786
centroid_9740	HTH-like domain protein	3	4	2	116	centroid_974	19.4224803	1.05E-05	centroid_974	0.00104786
centroid_1085	evolved beta-galactosidase subunit alpha	3	119	2	1	centroid_108	16.9377561	3.86E-05	centroid_108	0.00380803
centroid_10980	type VII secretion system (T7SS), usher family protein	3	119	2	1	centroid_1098	16.9377561	3.86E-05	centroid_1098	0.00380803
centroid_1148	-negative pili assembly chaperone, N-terminal domain pr	3	119	2	1	centroid_114	16.9377561	3.86E-05	centroid_114	0.00380803
centroid_1150	conserved hypothetical protein	3	119	2	1	centroid_115	16.9377561	3.86E-05	centroid_115	0.00380803
centroid_11582	conserved hypothetical protein	3	119	2	1	centroid_1158	16.9377561	3.86E-05	centroid_1158	0.00380803
centroid_12428	inner membrane protein YqiK	3	119	2	1	centroid_1242	16.9377561	3.86E-05	centroid_1242	0.00380803
centroid_12573	fimbrial domain protein	3	119	2	1	centroid_1257	16.9377561	3.86E-05	centroid_1257	0.00380803
centroid_12605	fimbrial family protein	3	119	2	1	centroid_1260	16.9377561	3.86E-05	centroid_1260	0.00380803
centroid_12973	lysR substrate binding domain protein	3	119	2	1	centroid_1297	16.9377561	3.86E-05	centroid_1297	0.00380803
centroid_13350	cupin family protein	3	119	2	1	centroid_1335	16.9377561	3.86E-05	centroid_1335	0.00380803
centroid_14271	type VII secretion system (T7SS), usher family protein	3	119	2	1	centroid_1427	16.9377561	3.86E-05	centroid_1427	0.00380803
centroid_14282	putative membrane protein	3	119	2	1	centroid_1428	16.9377561	3.86E-05	centroid_1428	0.00380803
centroid_14718	conserved hypothetical protein	3	119	2	1	centroid_1471	16.9377561	3.86E-05	centroid_1471	0.00380803
centroid_15311	sdiA-regulated family protein	3	119	2	1	centroid_1531	16.9377561	3.86E-05	centroid_1531	0.00380803
centroid_15312	sdiA-regulated family protein	3	119	2	1	centroid_1531	16.9377561	3.86E-05	centroid_1531	0.00380803
centroid_17157	glucose inhibited division A family protein	3	119	2	1	centroid_1715	16.9377561	3.86E-05	centroid_1715	0.00380803
centroid_17169	pyridine nucleotide-disulfide oxidoreductase family protei	3	119	2	1	centroid_1716	16.9377561	3.86E-05	centroid_1716	0.00380803
centroid_18594	type VII secretion system (T7SS), usher family protein	3	119	2	1	centroid_1859	16.9377561	3.86E-05	centroid_1859	0.00380803
centroid_2051	cryptic outer membrane porin BglH	3	119	2	1	centroid_205	16.9377561	3.86E-05	centroid_205	0.00380803
centroid_2484	efflux transporter, RND family, MFP subunit	3	119	2	1	centroid_248	16.9377561	3.86E-05	centroid_248	0.00380803
centroid_3380	alpha amylase, catalytic domain protein	3	119	2	1	centroid_338	16.9377561	3.86E-05	centroid_338	0.00380803
centroid_3671	conserved hypothetical protein	3	119	2	1	centroid_367	16.9377561	3.86E-05	centroid_367	0.00380803
centroid_3672	conserved hypothetical protein	3	119	2	1	centroid_367	16.9377561	3.86E-05	centroid_367	0.00380803
centroid_3808	cupin family protein	3	119	2	1	centroid_380	16.9377561	3.86E-05	centroid_380	0.00380803
centroid_4723	conserved hypothetical protein	3	119	2	1	centroid_472	16.9377561	3.86E-05	centroid_472	0.00380803
centroid_5930	putative alpha amylase	3	119	2	1	centroid_593	16.9377561	3.86E-05	centroid_593	0.00380803
centroid_729	phosphonate metabolism protein PhnP	3	119	2	1	centroid_729	16.9377561	3.86E-05	centroid_729	0.00380803
centroid_7817	major Facilitator Superfamily protein	3	119	2	1	centroid_781	16.9377561	3.86E-05	centroid_781	0.00380803
centroid_8520	conserved hypothetical protein	3	119	2	1	centroid_852	16.9377561	3.86E-05	centroid_852	0.00380803
centroid_10630	prokaryotic metallothionein family protein	3	5	2	115	centroid_1063	16.5278334	4.79E-05	centroid_1063	0.00165548
centroid_12899	putative caudovirales tail fiber assembly protein	3	5	2	115	centroid_1289	16.5278334	4.79E-05	centroid_1289	0.00165548

centroid_13880	gram-negative porin family protein	3	5	2	115	centroid_1388	16.5278334	4.79E-05	centroid_1388	0.00165548
centroid_9473	outer membrane protein N	3	5	2	115	centroid_9473	16.5278334	4.79E-05	centroid_9473	0.00165548
centroid_9666	conserved hypothetical protein	3	5	2	115	centroid_9666	16.5278334	4.79E-05	centroid_9666	0.00165548
centroid_9713	putative domain protein	3	5	2	115	centroid_9713	16.5278334	4.79E-05	centroid_9713	0.00165548
centroid_9718	conserved hypothetical protein	3	5	2	115	centroid_9718	16.5278334	4.79E-05	centroid_9718	0.00165548
centroid_9719	conserved hypothetical protein	3	5	2	115	centroid_9719	16.5278334	4.79E-05	centroid_9719	0.00165548
centroid_9720	conserved hypothetical protein	3	5	2	115	centroid_9720	16.5278334	4.79E-05	centroid_9720	0.00165548
centroid_9790	putative domain protein	3	5	2	115	centroid_9790	16.5278334	4.79E-05	centroid_9790	0.00165548
centroid_10279	orn/Lys/Arg decarboxylase, major domain protein	2	120	3	0	centroid_1027	50.3792407	1.27E-12	centroid_1027	3.15E-05
centroid_1034	conserved hypothetical protein	2	120	3	0	centroid_1034	50.3792407	1.27E-12	centroid_1034	3.15E-05
centroid_11	swarming motility protein YbiA	2	120	3	0	centroid_11	50.3792407	1.27E-12	centroid_11	3.15E-05
centroid_11247	putrescine-ornithine antiporter	2	120	3	0	centroid_1124	50.3792407	1.27E-12	centroid_1124	3.15E-05
centroid_12471	putative acyl transferase domain protein	2	120	3	0	centroid_1247	50.3792407	1.27E-12	centroid_1247	3.15E-05
centroid_12472	putative acyl transferase domain protein	2	120	3	0	centroid_1247	50.3792407	1.27E-12	centroid_1247	3.15E-05
centroid_12548	glycosyl hydrolases family 38 C-terminal domain protein	2	120	3	0	centroid_1254	50.3792407	1.27E-12	centroid_1254	3.15E-05
centroid_12961	leucine Rich repeats family protein	2	120	3	0	centroid_1296	50.3792407	1.27E-12	centroid_1296	3.15E-05
centroid_13999	bacterial transferase hexapeptide family protein	2	120	3	0	centroid_1399	50.3792407	1.27E-12	centroid_1399	3.15E-05
centroid_15515	glcNAc-PI de-N-acetylase family protein	2	120	3	0	centroid_1551	50.3792407	1.27E-12	centroid_1551	3.15E-05
centroid_16360	eamA-like transporter family protein	2	120	3	0	centroid_1636	50.3792407	1.27E-12	centroid_1636	3.15E-05
centroid_16440	leucine Rich repeats family protein	2	120	3	0	centroid_1644	50.3792407	1.27E-12	centroid_1644	3.15E-05
centroid_16484	p-aminobenzoyl-glutamate transport protein	2	120	3	0	centroid_1648	50.3792407	1.27E-12	centroid_1648	3.15E-05
centroid_16885	orn/Lys/Arg decarboxylase, C-terminal domain protein	2	120	3	0	centroid_1688	50.3792407	1.27E-12	centroid_1688	3.15E-05
centroid_17768	ulp1 protease family, C-terminal catalytic domain protein	2	120	3	0	centroid_1776	50.3792407	1.27E-12	centroid_1776	3.15E-05
centroid_18273	ulp1 protease family, C-terminal catalytic domain protein	2	120	3	0	centroid_1827	50.3792407	1.27E-12	centroid_1827	3.15E-05
centroid_1878	conserved hypothetical protein	2	120	3	0	centroid_1878	50.3792407	1.27E-12	centroid_1878	3.15E-05
centroid_2481	DNA methylase family protein	2	120	3	0	centroid_2481	50.3792407	1.27E-12	centroid_2481	3.15E-05
centroid_3062	leucine Rich repeats family protein	2	120	3	0	centroid_3062	50.3792407	1.27E-12	centroid_3062	3.15E-05
centroid_3427	p-aminobenzoyl-glutamate transport protein	2	120	3	0	centroid_3427	50.3792407	1.27E-12	centroid_3427	3.15E-05
centroid_4270	conserved hypothetical protein	2	120	3	0	centroid_4270	50.3792407	1.27E-12	centroid_4270	3.15E-05
centroid_493	glcNAc-PI de-N-acetylase family protein	2	120	3	0	centroid_493	50.3792407	1.27E-12	centroid_493	3.15E-05
centroid_494	glycosyl transferase 2 family protein	2	120	3	0	centroid_494	50.3792407	1.27E-12	centroid_494	3.15E-05
centroid_495	putative acyl transferase	2	120	3	0	centroid_495	50.3792407	1.27E-12	centroid_495	3.15E-05
centroid_7341	conserved hypothetical protein	2	120	3	0	centroid_7341	50.3792407	1.27E-12	centroid_7341	3.15E-05
centroid_7342	conserved hypothetical domain protein	2	120	3	0	centroid_7342	50.3792407	1.27E-12	centroid_7342	3.15E-05
centroid_8142	putative acyl transferase	2	120	3	0	centroid_8142	50.3792407	1.27E-12	centroid_8142	3.15E-05
centroid_15215	-negative pili assembly chaperone, N-terminal domain pr	2	119	3	1	centroid_1521	36.8268982	1.29E-09	centroid_1521	0.00012434
centroid_18162	putative ubiquitin carboxyl-terminal hydrolase 45	2	119	3	1	centroid_1816	36.8268982	1.29E-09	centroid_1816	0.00012434
centroid_189	conserved hypothetical protein	2	119	3	1	centroid_189	36.8268982	1.29E-09	centroid_189	0.00012434
centroid_4299	-negative pili assembly chaperone, N-terminal domain pr	2	119	3	1	centroid_4299	36.8268982	1.29E-09	centroid_4299	0.00012434
centroid_6216	conserved hypothetical protein	2	119	3	1	centroid_6216	36.8268982	1.29E-09	centroid_6216	0.00012434
centroid_7652	orn/Lys/Arg decarboxylase, major domain protein	2	119	3	1	centroid_7652	36.8268982	1.29E-09	centroid_7652	0.00012434
centroid_10762	dnaJ domain protein	2	118	3	2	centroid_1076	28.7000868	8.45E-08	centroid_1076	0.000307
centroid_10763	putative heat shock DnaJ domain-containing protein	2	118	3	2	centroid_1076	28.7000868	8.45E-08	centroid_1076	0.000307
centroid_14212	hsp70 family protein	2	118	3	2	centroid_1421	28.7000868	8.45E-08	centroid_1421	0.000307
centroid_1447	sel1 repeat family protein	2	118	3	2	centroid_1447	28.7000868	8.45E-08	centroid_1447	0.000307
centroid_1448	conserved hypothetical protein	2	118	3	2	centroid_1448	28.7000868	8.45E-08	centroid_1448	0.000307
centroid_1449	dnaJ domain protein	2	118	3	2	centroid_1449	28.7000868	8.45E-08	centroid_1449	0.000307
centroid_1450	sel1 repeat family protein	2	118	3	2	centroid_1450	28.7000868	8.45E-08	centroid_1450	0.000307
centroid_1451	conserved hypothetical protein	2	118	3	2	centroid_1451	28.7000868	8.45E-08	centroid_1451	0.000307
centroid_1452	dnaJ domain protein	2	118	3	2	centroid_1452	28.7000868	8.45E-08	centroid_1452	0.000307
centroid_1453	hsp70 family protein	2	118	3	2	centroid_1453	28.7000868	8.45E-08	centroid_1453	0.000307
centroid_1544	fimbrial family protein	2	118	3	2	centroid_1544	28.7000868	8.45E-08	centroid_1544	0.000307
centroid_17321	hsp70 family protein	2	118	3	2	centroid_1732	28.7000868	8.45E-08	centroid_1732	0.000307
centroid_17322	putative truncated dnaK protein	2	118	3	2	centroid_1732	28.7000868	8.45E-08	centroid_1732	0.000307
centroid_484	protein SbmA	2	118	3	2	centroid_484	28.7000868	8.45E-08	centroid_484	0.000307
centroid_13488	hypothetical protein	2	0	3	120	centroid_1348	26.6821223	2.40E-07	centroid_1348	0.00129032
centroid_13492	conserved hypothetical protein	2	0	3	120	centroid_1349	26.6821223	2.40E-07	centroid_1349	0.00129032
centroid_13503	putative mobA/MobL protein	2	0	3	120	centroid_1350	26.6821223	2.40E-07	centroid_1350	0.00129032
centroid_14312	inner membrane protein YcfZ	2	0	3	120	centroid_1431	26.6821223	2.40E-07	centroid_1431	0.00129032
centroid_14320	conserved hypothetical protein	2	0	3	120	centroid_1432	26.6821223	2.40E-07	centroid_1432	0.00129032
centroid_14386	RNA ligase family protein	2	0	3	120	centroid_1438	26.6821223	2.40E-07	centroid_1438	0.00129032
centroid_14387	conserved hypothetical protein	2	0	3	120	centroid_1438	26.6821223	2.40E-07	centroid_1438	0.00129032
centroid_14388	AAA domain protein	2	0	3	120	centroid_1438	26.6821223	2.40E-07	centroid_1438	0.00129032
centroid_14406	conserved hypothetical protein	2	0	3	120	centroid_1440	26.6821223	2.40E-07	centroid_1440	0.00129032
centroid_14430	invasion protein InvA	2	0	3	120	centroid_1443	26.6821223	2.40E-07	centroid_1443	0.00129032
centroid_14431	bacterial type II and III secretion system family protein	2	0	3	120	centroid_1443	26.6821223	2.40E-07	centroid_1443	0.00129032
centroid_14464	hypothetical protein	2	0	3	120	centroid_1446	26.6821223	2.40E-07	centroid_1446	0.00129032
centroid_14472	conserved hypothetical protein	2	0	3	120	centroid_1447	26.6821223	2.40E-07	centroid_1447	0.00129032
centroid_14503	conserved hypothetical protein	2	0	3	120	centroid_1450	26.6821223	2.40E-07	centroid_1450	0.00129032
centroid_14521	oglucomutase/phosphomannomutase, C-terminal domain	2	0	3	120	centroid_1452	26.6821223	2.40E-07	centroid_1452	0.00129032
centroid_17822	bacterial regulatory helix-turn-helix, AraC family protein	2	0	3	120	centroid_1782	26.6821223	2.40E-07	centroid_1782	0.00129032
centroid_17823	amidotransferase family protein	2	0	3	120	centroid_1782	26.6821223	2.40E-07	centroid_1782	0.00129032
centroid_17824	conserved hypothetical protein	2	0	3	120	centroid_1782	26.6821223	2.40E-07	centroid_1782	0.00129032
centroid_17921	conserved hypothetical protein	2	0	3	120	centroid_1792	26.6821223	2.40E-07	centroid_1792	0.00129032
centroid_17972	AAA domain protein	2	0	3	120	centroid_1797	26.6821223	2.40E-07	centroid_1797	0.00129032
centroid_17975	on-LEE-encoded type III secreted effector domain protei	2	0	3	120	centroid_1797	26.6821223	2.40E-07	centroid_1797	0.00129032
centroid_17976	hypothetical protein	2	0	3	120	centroid_1797	26.6821223	2.40E-07	centroid_1797	0.00129032
centroid_18220	conserved hypothetical protein	2	0	3	120	centroid_1822	26.6821223	2.40E-07	centroid_1822	0.00129032
centroid_18231	conserved hypothetical protein	2	0	3	120	centroid_1823	26.6821223	2.40E-07	centroid_1823	0.00129032
centroid_18236	autotransporter beta-domain protein	2	0	3	120	centroid_1823	26.6821223	2.40E-07	centroid_1823	0.00129032
centroid_18261	conserved hypothetical protein	2	0	3	120	centroid_1826	26.6821223	2.40E-07	centroid_1826	0.00129032
centroid_8759	conserved hypothetical protein	2	0	3	120	centroid_8759	26.6821223	2.40E-07	centroid_8759	0.00129032
centroid_8760	conserved hypothetical protein	2	0	3	120	centroid_8760	26.6821223	2.40E-07	centroid_8760	0.00129032
centroid_8761	sopA-like catalytic domain protein	2	0	3	120	centroid_8761	26.6821223	2.40E-07	centroid_8761	0.00129032
centroid_8764	S system sugar-specific permease component family pro	2	0	3	120	centroid_8764	26.6821223	2.40E-07	centroid_8764	0.00129032
centroid_8765	PTS system, Lactose/Cellobiose specific IIB subunit	2	0	3	120	centroid_8765	26.6821223	2.40E-07	centroid_8765	0.00129032
centroid_8766	sulfatase family protein	2	0	3	120	centroid_8766	26.6821223	2.40E-07	centroid_8766	0.00129032
centroid_8803	pentapeptide repeats family protein	2	0	3	120	centroid_8803	26.6821223	2.40E-07	centroid_8803	0.00129032
centroid_8804	ccdB family protein	2	0	3	120	centroid_8804	26.6821223	2.40E-07	centroid_8804	0.00129032
centroid_8805	post-segregation antitoxin CcdA family protein	2	0	3	120	centroid_8805	26.6821223	2.40E-07	centroid_8805	0.00129032
centroid_8825	conserved hypothetical protein	2	0	3	120	centroid_8825	26.6821223	2.40E-07	centroid_8825	0.00129032
centroid_8826	AMP-binding enzyme family protein	2	0	3	120	centroid_8826	26.6821223	2.40E-07	centroid_8826	0.00129032
centroid_8827	putative membrane protein	2	0	3	120	centroid_8827	26.6821223	2.40E-07	centroid_8827	0.00129032
centroid_8832	putative lipoprotein	2	0	3	120	centroid_8832	26.6821223	2.40E-07	centroid_8832	0.00129032
centroid_8840	putative acetyltransferase	2	0	3	120	centroid_8840	26.6821223	2.40E-07	centroid_8840	0.00129032
centroid_8841	conserved hypothetical protein	2	0	3	120	centroid_8841	26.6821223	2.40E-07	centroid_8841	0.00129032
centroid_8856	type III secretion regulator YopN/LcrE/InvE/MxiC	2	0	3	120	centroid_8856	26.6821223	2.40E-07	centroid_8856	0.00129032
centroid_8890	conserved hypothetical protein	2	0	3	120	centroid_8890	26.6821223	2.40E-07	centroid_8890	0.00129032
centroid_8891	acyl transferase domain protein	2	0	3	120	centroid_8891	26.6821223	2.40E-07	centroid_8891	0.00129032
centroid_8911	bacterial regulatory, tetR family protein	2	0	3	120	centroid_8911	26.6821223	2.40E-07	centroid_8911	0.00129032
centroid_8912	efflux transporter, RND family, MFP subunit	2	0	3	120	centroid_8912	26.6821223	2.40E-07	centroid_8912	0.00129032
centroid_8913	D transporter, hydrophobe/amphiphile efflux-1 family prot	2	0	3	120	centroid_8913	26.6821223	2.40E-07	centroid_8913	0.00129032
centroid_8914	ansporter, outer membrane factor (OMF) lipo, NodT famil	2	0	3	120	centroid_8914	26.6821223	2.40E-07	centroid_8914	0.00129032
centroid_8915	drug resistance transporter, Bcr/CflA subfamily protein	2	0	3	120	centroid_8915	26.6821223	2.40E-07	centroid_8915	0.00129032
centroid_8938	conserved hypothetical protein	2	0	3	120	centroid_8938	26.6821223	2.40E-07	centroid_8938	0.00129032
centroid_8939	sodium:dicarboxylate symporter family protein	2	0	3	120	centroid_8939	26.6821223	2.40E-07	centroid_8939	0.00129032
centroid_8940	fumarate hydratase class I, aerobic	2	0	3	120	centroid_8940	26.6821223	2.40E-07	centroid_8940	0.00129032

centroid_8941	cob(I)yrinic acid a,c-diamide adenosyltransferase	2	0	3	120	centroid_894	26.6821223	2.40E-07	centroid_894	0.00129032
centroid_8942	bacterial regulatory helix-turn-helix , lysR family protein	2	0	3	120	centroid_894	26.6821223	2.40E-07	centroid_894	0.00129032
centroid_8943	fimbrial family protein	2	0	3	120	centroid_894	26.6821223	2.40E-07	centroid_894	0.00129032
centroid_8944	type VII secretion system (T7SS), usher family protein	2	0	3	120	centroid_894	26.6821223	2.40E-07	centroid_894	0.00129032
centroid_8945	-negative pili assembly chaperone, C-terminal domain pr	2	0	3	120	centroid_894	26.6821223	2.40E-07	centroid_894	0.00129032
centroid_8946	fimbrial family protein	2	0	3	120	centroid_894	26.6821223	2.40E-07	centroid_894	0.00129032
centroid_8951	conserved hypothetical protein	2	0	3	120	centroid_895	26.6821223	2.40E-07	centroid_895	0.00129032
centroid_8965	amidotransferase family protein	2	0	3	120	centroid_896	26.6821223	2.40E-07	centroid_896	0.00129032
centroid_8966	beta-lactamase superfamily domain protein	2	0	3	120	centroid_896	26.6821223	2.40E-07	centroid_896	0.00129032
centroid_8973	outer membrane autotransporter barrel domain protein	2	0	3	120	centroid_897	26.6821223	2.40E-07	centroid_897	0.00129032
centroid_8986	sugar (and other) transporter family protein	2	0	3	120	centroid_898	26.6821223	2.40E-07	centroid_898	0.00129032
centroid_9000	lysR substrate binding domain protein	2	0	3	120	centroid_900	26.6821223	2.40E-07	centroid_900	0.00129032
centroid_9001	oxidoreductase	2	0	3	120	centroid_900	26.6821223	2.40E-07	centroid_900	0.00129032
centroid_9002	putative ybiJ	2	0	3	120	centroid_900	26.6821223	2.40E-07	centroid_900	0.00129032
centroid_9003	bacterial regulatory helix-turn-helix , lysR family protein	2	0	3	120	centroid_900	26.6821223	2.40E-07	centroid_900	0.00129032
centroid_9004	conserved hypothetical protein	2	0	3	120	centroid_900	26.6821223	2.40E-07	centroid_900	0.00129032
centroid_9005	sugar (and other) transporter family protein	2	0	3	120	centroid_900	26.6821223	2.40E-07	centroid_900	0.00129032
centroid_9046	ynbE-like lipofamily protein	2	0	3	120	centroid_904	26.6821223	2.40E-07	centroid_904	0.00129032
centroid_9096	putative amino-acid metabolite efflux pump	2	0	3	120	centroid_909	26.6821223	2.40E-07	centroid_909	0.00129032
centroid_9118	molybdenum-pterin binding domain protein	2	0	3	120	centroid_911	26.6821223	2.40E-07	centroid_911	0.00129032
centroid_9121	leucine Rich repeats family protein	2	0	3	120	centroid_912	26.6821223	2.40E-07	centroid_912	0.00129032
centroid_9130	conserved hypothetical protein	2	0	3	120	centroid_913	26.6821223	2.40E-07	centroid_913	0.00129032
centroid_9131	conserved hypothetical protein	2	0	3	120	centroid_913	26.6821223	2.40E-07	centroid_913	0.00129032
centroid_9132	conserved hypothetical protein	2	0	3	120	centroid_913	26.6821223	2.40E-07	centroid_913	0.00129032
centroid_9133	type VI secretion system effector, Hcp1 family protein	2	0	3	120	centroid_913	26.6821223	2.40E-07	centroid_913	0.00129032
centroid_9235	conserved hypothetical protein	2	0	3	120	centroid_923	26.6821223	2.40E-07	centroid_923	0.00129032
centroid_9251	resolvase, N terminal domain protein	2	0	3	120	centroid_925	26.6821223	2.40E-07	centroid_925	0.00129032
centroid_9268	type VI secretion system effector, Hcp1 family protein	2	0	3	120	centroid_926	26.6821223	2.40E-07	centroid_926	0.00129032
centroid_9269	colicin-E7 immunity protein	2	0	3	120	centroid_926	26.6821223	2.40E-07	centroid_926	0.00129032
centroid_9270	colicin-E7 immunity protein	2	0	3	120	centroid_927	26.6821223	2.40E-07	centroid_927	0.00129032
centroid_9271	colicin-E7 immunity protein	2	0	3	120	centroid_927	26.6821223	2.40E-07	centroid_927	0.00129032
centroid_9298	evolved beta-galactosidase subunit alpha	2	0	3	120	centroid_929	26.6821223	2.40E-07	centroid_929	0.00129032
centroid_9312	conserved hypothetical protein	2	0	3	120	centroid_931	26.6821223	2.40E-07	centroid_931	0.00129032
centroid_9322	-negative pili assembly chaperone, C-terminal domain pr	2	0	3	120	centroid_932	26.6821223	2.40E-07	centroid_932	0.00129032
centroid_9323	type VII secretion system (T7SS), usher family protein	2	0	3	120	centroid_932	26.6821223	2.40E-07	centroid_932	0.00129032
centroid_9325	pe I restriction modification DNA specificity domain prote	2	0	3	120	centroid_932	26.6821223	2.40E-07	centroid_932	0.00129032
centroid_9326	helix-turn-helix domain protein	2	0	3	120	centroid_932	26.6821223	2.40E-07	centroid_932	0.00129032
centroid_9350	conserved hypothetical protein	2	0	3	120	centroid_935	26.6821223	2.40E-07	centroid_935	0.00129032
centroid_9356	poxvirus D5 protein-like family protein	2	0	3	120	centroid_935	26.6821223	2.40E-07	centroid_935	0.00129032
centroid_9357	conserved hypothetical protein	2	0	3	120	centroid_935	26.6821223	2.40E-07	centroid_935	0.00129032
centroid_9359	ash family protein	2	0	3	120	centroid_935	26.6821223	2.40E-07	centroid_935	0.00129032
centroid_9361	putative glyco3, capsid size determination protein Sid	2	0	3	120	centroid_936	26.6821223	2.40E-07	centroid_936	0.00129032
centroid_9364	conserved hypothetical protein	2	0	3	120	centroid_936	26.6821223	2.40E-07	centroid_936	0.00129032
centroid_9365	conserved hypothetical protein	2	0	3	120	centroid_936	26.6821223	2.40E-07	centroid_936	0.00129032
centroid_9366	integrase	2	0	3	120	centroid_936	26.6821223	2.40E-07	centroid_936	0.00129032
centroid_9380	PAAR motif family protein	2	0	3	120	centroid_938	26.6821223	2.40E-07	centroid_938	0.00129032
centroid_9381	rhs element Vgr family protein	2	0	3	120	centroid_938	26.6821223	2.40E-07	centroid_938	0.00129032
centroid_9382	phage-related baseplate assembly family protein	2	0	3	120	centroid_938	26.6821223	2.40E-07	centroid_938	0.00129032
centroid_9383	conserved hypothetical protein	2	0	3	120	centroid_938	26.6821223	2.40E-07	centroid_938	0.00129032
centroid_9384	conserved hypothetical protein	2	0	3	120	centroid_938	26.6821223	2.40E-07	centroid_938	0.00129032
centroid_9385	peptidase C39 family protein	2	0	3	120	centroid_938	26.6821223	2.40E-07	centroid_938	0.00129032
centroid_9390	conserved hypothetical protein	2	0	3	120	centroid_939	26.6821223	2.40E-07	centroid_939	0.00129032
centroid_9391	conserved hypothetical protein	2	0	3	120	centroid_939	26.6821223	2.40E-07	centroid_939	0.00129032
centroid_9395	conserved hypothetical protein	2	0	3	120	centroid_939	26.6821223	2.40E-07	centroid_939	0.00129032
centroid_9396	hypothetical protein	2	0	3	120	centroid_939	26.6821223	2.40E-07	centroid_939	0.00129032
centroid_9397	conserved hypothetical protein	2	0	3	120	centroid_939	26.6821223	2.40E-07	centroid_939	0.00129032
centroid_9398	conserved hypothetical protein	2	0	3	120	centroid_939	26.6821223	2.40E-07	centroid_939	0.00129032
centroid_9401	conserved hypothetical protein	2	0	3	120	centroid_940	26.6821223	2.40E-07	centroid_940	0.00129032
centroid_9403	DNA N-6-adenine-methyltransferase family protein	2	0	3	120	centroid_940	26.6821223	2.40E-07	centroid_940	0.00129032
centroid_9405	flagellin	2	0	3	120	centroid_940	26.6821223	2.40E-07	centroid_940	0.00129032
centroid_9407	conserved hypothetical protein	2	0	3	120	centroid_940	26.6821223	2.40E-07	centroid_940	0.00129032
centroid_9410	hypothetical protein	2	0	3	120	centroid_941	26.6821223	2.40E-07	centroid_941	0.00129032
centroid_9411	hypothetical protein	2	0	3	120	centroid_941	26.6821223	2.40E-07	centroid_941	0.00129032
centroid_9412	PAAR motif family protein	2	0	3	120	centroid_941	26.6821223	2.40E-07	centroid_941	0.00129032
centroid_9426	conserved hypothetical protein	2	0	3	120	centroid_942	26.6821223	2.40E-07	centroid_942	0.00129032
centroid_9428	conserved hypothetical protein	2	0	3	120	centroid_942	26.6821223	2.40E-07	centroid_942	0.00129032
centroid_9433	putative exported protein	2	0	3	120	centroid_943	26.6821223	2.40E-07	centroid_943	0.00129032
centroid_9434	conserved hypothetical protein	2	0	3	120	centroid_943	26.6821223	2.40E-07	centroid_943	0.00129032
centroid_9435	conserved hypothetical protein	2	0	3	120	centroid_943	26.6821223	2.40E-07	centroid_943	0.00129032
centroid_9436	conserved hypothetical protein	2	0	3	120	centroid_943	26.6821223	2.40E-07	centroid_943	0.00129032
centroid_9437	phage major capsid E family protein	2	0	3	120	centroid_943	26.6821223	2.40E-07	centroid_943	0.00129032
centroid_9438	conserved hypothetical protein	2	0	3	120	centroid_943	26.6821223	2.40E-07	centroid_943	0.00129032
centroid_9439	hypothetical protein	2	0	3	120	centroid_943	26.6821223	2.40E-07	centroid_943	0.00129032
centroid_9440	conserved hypothetical protein	2	0	3	120	centroid_944	26.6821223	2.40E-07	centroid_944	0.00129032
centroid_9441	conserved hypothetical protein	2	0	3	120	centroid_944	26.6821223	2.40E-07	centroid_944	0.00129032
centroid_9442	conserved hypothetical protein	2	0	3	120	centroid_944	26.6821223	2.40E-07	centroid_944	0.00129032
centroid_9443	conserved hypothetical protein	2	0	3	120	centroid_944	26.6821223	2.40E-07	centroid_944	0.00129032
centroid_9459	relaxase/Mobilisation nuclease domain protein	2	0	3	120	centroid_945	26.6821223	2.40E-07	centroid_945	0.00129032
centroid_9460	conserved hypothetical protein	2	0	3	120	centroid_946	26.6821223	2.40E-07	centroid_946	0.00129032
centroid_9461	tetratricopeptide repeat family protein	2	0	3	120	centroid_946	26.6821223	2.40E-07	centroid_946	0.00129032
centroid_9462	hypothetical protein	2	0	3	120	centroid_946	26.6821223	2.40E-07	centroid_946	0.00129032
centroid_9463	hypothetical protein	2	0	3	120	centroid_946	26.6821223	2.40E-07	centroid_946	0.00129032
centroid_9469	conserved hypothetical protein	2	0	3	120	centroid_946	26.6821223	2.40E-07	centroid_946	0.00129032
centroid_9470	putative predicted protein	2	0	3	120	centroid_947	26.6821223	2.40E-07	centroid_947	0.00129032
centroid_9476	hypothetical protein	2	0	3	120	centroid_947	26.6821223	2.40E-07	centroid_947	0.00129032
centroid_9477	conserved hypothetical protein	2	0	3	120	centroid_947	26.6821223	2.40E-07	centroid_947	0.00129032
centroid_9478	HNH endonuclease family protein	2	0	3	120	centroid_947	26.6821223	2.40E-07	centroid_947	0.00129032
centroid_9479	bacteriophage lysis family protein	2	0	3	120	centroid_947	26.6821223	2.40E-07	centroid_947	0.00129032
centroid_9484	hage tail tape measure protein, TP901 family, core regio	2	0	3	120	centroid_948	26.6821223	2.40E-07	centroid_948	0.00129032
centroid_9485	conserved hypothetical protein	2	0	3	120	centroid_948	26.6821223	2.40E-07	centroid_948	0.00129032
centroid_9486	conserved hypothetical protein	2	0	3	120	centroid_948	26.6821223	2.40E-07	centroid_948	0.00129032
centroid_9487	conserved hypothetical protein	2	0	3	120	centroid_948	26.6821223	2.40E-07	centroid_948	0.00129032
centroid_9488	conserved hypothetical protein	2	0	3	120	centroid_948	26.6821223	2.40E-07	centroid_948	0.00129032
centroid_9489	conserved hypothetical protein	2	0	3	120	centroid_948	26.6821223	2.40E-07	centroid_948	0.00129032
centroid_9490	conserved hypothetical protein	2	0	3	120	centroid_949	26.6821223	2.40E-07	centroid_949	0.00129032
centroid_9497	conserved hypothetical protein	2	0	3	120	centroid_949	26.6821223	2.40E-07	centroid_949	0.00129032
centroid_9498	reverse transcriptase family protein	2	0	3	120	centroid_949	26.6821223	2.40E-07	centroid_949	0.00129032
centroid_9499	reverse transcriptase family protein	2	0	3	120	centroid_949	26.6821223	2.40E-07	centroid_949	0.00129032
centroid_9500	phage integrase family protein	2	0	3	120	centroid_950	26.6821223	2.40E-07	centroid_950	0.00129032
centroid_9503	hypothetical protein	2	0	3	120	centroid_950	26.6821223	2.40E-07	centroid_950	0.00129032
centroid_9505	resolvase, N terminal domain protein	2	0	3	120	centroid_950	26.6821223	2.40E-07	centroid_950	0.00129032
centroid_9506	conserved hypothetical protein	2	0	3	120	centroid_950	26.6821223	2.40E-07	centroid_950	0.00129032
centroid_9507	phage-like baseplate assembly domain protein	2	0	3	120	centroid_950	26.6821223	2.40E-07	centroid_950	0.00129032
centroid_9508	hypothetical protein	2	0	3	120	centroid_950	26.6821223	2.40E-07	centroid_950	0.00129032
centroid_9509	putative zinc-dependent metallopeptidase	2	0	3	120	centroid_950	26.6821223	2.40E-07	centroid_950	0.00129032
centroid_9511	conserved hypothetical protein	2	0	3	120	centroid_951	26.6821223	2.40E-07	centroid_951	0.00129032
centroid_9512	conserved hypothetical protein	2	0	3	120	centroid_951	26.6821223	2.40E-07	centroid_951	0.00129032

centroid_9513	conserved hypothetical protein	2	0	3	120	centroid_9513	26.6821223	2.40E-07	centroid_9513	0.00129032
centroid_9514	rhs element Vgr family protein	2	0	3	120	centroid_9514	26.6821223	2.40E-07	centroid_9514	0.00129032
centroid_9515	evfW domain protein	2	0	3	120	centroid_9515	26.6821223	2.40E-07	centroid_9515	0.00129032
centroid_9516	conserved hypothetical protein	2	0	3	120	centroid_9516	26.6821223	2.40E-07	centroid_9516	0.00129032
centroid_9517	type VI secretion system effector, Hcp1 family protein	2	0	3	120	centroid_9517	26.6821223	2.40E-07	centroid_9517	0.00129032
centroid_9518	conserved hypothetical protein	2	0	3	120	centroid_9518	26.6821223	2.40E-07	centroid_9518	0.00129032
centroid_9519	conserved hypothetical protein	2	0	3	120	centroid_9519	26.6821223	2.40E-07	centroid_9519	0.00129032
centroid_9520	gene 25-like lysozyme family protein	2	0	3	120	centroid_9520	26.6821223	2.40E-07	centroid_9520	0.00129032
centroid_9521	conserved hypothetical protein	2	0	3	120	centroid_9521	26.6821223	2.40E-07	centroid_9521	0.00129032
centroid_9522	conserved hypothetical protein	2	0	3	120	centroid_9522	26.6821223	2.40E-07	centroid_9522	0.00129032
centroid_9523	FHA domain protein	2	0	3	120	centroid_9523	26.6821223	2.40E-07	centroid_9523	0.00129032
centroid_9524	type VI secretion lipofamily protein	2	0	3	120	centroid_9524	26.6821223	2.40E-07	centroid_9524	0.00129032
centroid_9525	conserved hypothetical protein	2	0	3	120	centroid_9525	26.6821223	2.40E-07	centroid_9525	0.00129032
centroid_9526	conserved hypothetical protein	2	0	3	120	centroid_9526	26.6821223	2.40E-07	centroid_9526	0.00129032
centroid_9527	impA domain family protein	2	0	3	120	centroid_9527	26.6821223	2.40E-07	centroid_9527	0.00129032
centroid_9528	type VI secretion system effector, Hcp1 family protein	2	0	3	120	centroid_9528	26.6821223	2.40E-07	centroid_9528	0.00129032
centroid_9529	putative aminopeptidase	2	0	3	120	centroid_9529	26.6821223	2.40E-07	centroid_9529	0.00129032
centroid_9535	conserved hypothetical protein	2	0	3	120	centroid_9535	26.6821223	2.40E-07	centroid_9535	0.00129032
centroid_9536	hypothetical protein	2	0	3	120	centroid_9536	26.6821223	2.40E-07	centroid_9536	0.00129032
centroid_9537	WYL domain protein	2	0	3	120	centroid_9537	26.6821223	2.40E-07	centroid_9537	0.00129032
centroid_9540	grating conjugative element , PFL_4695 family domain pro	2	0	3	120	centroid_9540	26.6821223	2.40E-07	centroid_9540	0.00129032
centroid_9541	conserved hypothetical protein	2	0	3	120	centroid_9541	26.6821223	2.40E-07	centroid_9541	0.00129032
centroid_9543	phage integrase family protein	2	0	3	120	centroid_9543	26.6821223	2.40E-07	centroid_9543	0.00129032
centroid_9546	conserved hypothetical protein	2	0	3	120	centroid_9546	26.6821223	2.40E-07	centroid_9546	0.00129032
centroid_9547	phage integrase family protein	2	0	3	120	centroid_9547	26.6821223	2.40E-07	centroid_9547	0.00129032
centroid_9548	conserved hypothetical protein	2	0	3	120	centroid_9548	26.6821223	2.40E-07	centroid_9548	0.00129032
centroid_9549	conserved hypothetical protein	2	0	3	120	centroid_9549	26.6821223	2.40E-07	centroid_9549	0.00129032
centroid_9574	conserved hypothetical protein	2	0	3	120	centroid_9574	26.6821223	2.40E-07	centroid_9574	0.00129032
centroid_9579	hypothetical protein	2	0	3	120	centroid_9579	26.6821223	2.40E-07	centroid_9579	0.00129032
centroid_9580	rhs element Vgr family protein	2	0	3	120	centroid_9580	26.6821223	2.40E-07	centroid_9580	0.00129032
centroid_9581	conserved hypothetical protein	2	0	3	120	centroid_9581	26.6821223	2.40E-07	centroid_9581	0.00129032
centroid_9582	conserved hypothetical protein	2	0	3	120	centroid_9582	26.6821223	2.40E-07	centroid_9582	0.00129032
centroid_9583	hypothetical protein	2	0	3	120	centroid_9583	26.6821223	2.40E-07	centroid_9583	0.00129032
centroid_9584	conserved hypothetical protein	2	0	3	120	centroid_9584	26.6821223	2.40E-07	centroid_9584	0.00129032
centroid_9585	winged helix-turn-helix DNA-binding family protein	2	0	3	120	centroid_9585	26.6821223	2.40E-07	centroid_9585	0.00129032
centroid_9586	addiction module antidote protein, HigA family	2	0	3	120	centroid_9586	26.6821223	2.40E-07	centroid_9586	0.00129032
centroid_9588	integrase family protein	2	0	3	120	centroid_9588	26.6821223	2.40E-07	centroid_9588	0.00129032
centroid_9602	conserved hypothetical protein	2	0	3	120	centroid_9602	26.6821223	2.40E-07	centroid_9602	0.00129032
centroid_9603	conserved hypothetical protein	2	0	3	120	centroid_9603	26.6821223	2.40E-07	centroid_9603	0.00129032
centroid_9604	conserved hypothetical protein	2	0	3	120	centroid_9604	26.6821223	2.40E-07	centroid_9604	0.00129032
centroid_9605	conserved hypothetical protein	2	0	3	120	centroid_9605	26.6821223	2.40E-07	centroid_9605	0.00129032
centroid_9606	conserved hypothetical protein	2	0	3	120	centroid_9606	26.6821223	2.40E-07	centroid_9606	0.00129032
centroid_9607	sel1 repeat family protein	2	0	3	120	centroid_9607	26.6821223	2.40E-07	centroid_9607	0.00129032
centroid_9608	conserved hypothetical protein	2	0	3	120	centroid_9608	26.6821223	2.40E-07	centroid_9608	0.00129032
centroid_9609	conserved hypothetical protein	2	0	3	120	centroid_9609	26.6821223	2.40E-07	centroid_9609	0.00129032
centroid_9610	conserved hypothetical protein	2	0	3	120	centroid_9610	26.6821223	2.40E-07	centroid_9610	0.00129032
centroid_9611	hypothetical protein	2	0	3	120	centroid_9611	26.6821223	2.40E-07	centroid_9611	0.00129032
centroid_9612	conserved hypothetical protein	2	0	3	120	centroid_9612	26.6821223	2.40E-07	centroid_9612	0.00129032
centroid_9616	divergent AAA domain protein	2	0	3	120	centroid_9616	26.6821223	2.40E-07	centroid_9616	0.00129032
centroid_9617	conserved hypothetical protein	2	0	3	120	centroid_9617	26.6821223	2.40E-07	centroid_9617	0.00129032
centroid_9634	hep_Hag family protein	2	0	3	120	centroid_9634	26.6821223	2.40E-07	centroid_9634	0.00129032
centroid_9644	putative dNA transfer protein p33	2	0	3	120	centroid_9644	26.6821223	2.40E-07	centroid_9644	0.00129032
centroid_9646	conserved hypothetical protein	2	0	3	120	centroid_9646	26.6821223	2.40E-07	centroid_9646	0.00129032
centroid_9647	conserved hypothetical protein	2	0	3	120	centroid_9647	26.6821223	2.40E-07	centroid_9647	0.00129032
centroid_9648	perC transcriptional activator family protein	2	0	3	120	centroid_9648	26.6821223	2.40E-07	centroid_9648	0.00129032
centroid_9649	conserved hypothetical protein	2	0	3	120	centroid_9649	26.6821223	2.40E-07	centroid_9649	0.00129032
centroid_9650	plasmid pRiA4b ORF-3-like family protein	2	0	3	120	centroid_9650	26.6821223	2.40E-07	centroid_9650	0.00129032
centroid_9651	conserved hypothetical protein	2	0	3	120	centroid_9651	26.6821223	2.40E-07	centroid_9651	0.00129032
centroid_9656	pentapeptide repeats family protein	2	0	3	120	centroid_9656	26.6821223	2.40E-07	centroid_9656	0.00129032
centroid_9668	phage integrase family protein	2	0	3	120	centroid_9668	26.6821223	2.40E-07	centroid_9668	0.00129032
centroid_9669	phage integrase family protein	2	0	3	120	centroid_9669	26.6821223	2.40E-07	centroid_9669	0.00129032
centroid_9670	phage integrase family protein	2	0	3	120	centroid_9670	26.6821223	2.40E-07	centroid_9670	0.00129032
centroid_9671	conserved hypothetical protein	2	0	3	120	centroid_9671	26.6821223	2.40E-07	centroid_9671	0.00129032
centroid_9672	hypothetical protein	2	0	3	120	centroid_9672	26.6821223	2.40E-07	centroid_9672	0.00129032
centroid_9673	conserved hypothetical protein	2	0	3	120	centroid_9673	26.6821223	2.40E-07	centroid_9673	0.00129032
centroid_9674	conserved hypothetical protein	2	0	3	120	centroid_9674	26.6821223	2.40E-07	centroid_9674	0.00129032
centroid_9675	conserved hypothetical protein	2	0	3	120	centroid_9675	26.6821223	2.40E-07	centroid_9675	0.00129032
centroid_9696	lysis S family protein	2	0	3	120	centroid_9696	26.6821223	2.40E-07	centroid_9696	0.00129032
centroid_9700	conserved hypothetical protein	2	0	3	120	centroid_9700	26.6821223	2.40E-07	centroid_9700	0.00129032
centroid_9701	conserved hypothetical protein	2	0	3	120	centroid_9701	26.6821223	2.40E-07	centroid_9701	0.00129032
centroid_9702	modification methylase PvuII	2	0	3	120	centroid_9702	26.6821223	2.40E-07	centroid_9702	0.00129032
centroid_9703	conserved hypothetical protein	2	0	3	120	centroid_9703	26.6821223	2.40E-07	centroid_9703	0.00129032
centroid_9704	conjugal transfer TraD family protein	2	0	3	120	centroid_9704	26.6821223	2.40E-07	centroid_9704	0.00129032
centroid_9726	phage integrase family protein	2	0	3	120	centroid_9726	26.6821223	2.40E-07	centroid_9726	0.00129032
centroid_9727	putative membrane protein	2	0	3	120	centroid_9727	26.6821223	2.40E-07	centroid_9727	0.00129032
centroid_9728	conserved hypothetical protein	2	0	3	120	centroid_9728	26.6821223	2.40E-07	centroid_9728	0.00129032
centroid_9729	hypothetical protein	2	0	3	120	centroid_9729	26.6821223	2.40E-07	centroid_9729	0.00129032
centroid_9743	hypothetical protein	2	0	3	120	centroid_9743	26.6821223	2.40E-07	centroid_9743	0.00129032
centroid_9760	conserved hypothetical protein	2	0	3	120	centroid_9760	26.6821223	2.40E-07	centroid_9760	0.00129032
centroid_9761	P22AR C-terminal domain protein	2	0	3	120	centroid_9761	26.6821223	2.40E-07	centroid_9761	0.00129032
centroid_9792	prophage P4 integrase domain protein	2	0	3	120	centroid_9792	26.6821223	2.40E-07	centroid_9792	0.00129032
centroid_9796	recombinase family protein	2	0	3	120	centroid_9796	26.6821223	2.40E-07	centroid_9796	0.00129032
centroid_14221	papC N-terminal domain protein	2	117	3	3	centroid_1422	23.2861374	1.40E-06	centroid_1422	0.00060636
centroid_1483	orn/Lys/Arg decarboxylase, major domain protein	2	117	3	3	centroid_1483	23.2861374	1.40E-06	centroid_1483	0.00060636
centroid_1548	conserved hypothetical protein	2	117	3	3	centroid_1548	23.2861374	1.40E-06	centroid_1548	0.00060636
centroid_10278	type VII secretion system (T7SS), usher family protein	2	116	3	4	centroid_1027	19.4224803	1.05E-05	centroid_1027	0.00104786
centroid_10331	type VII secretion system (T7SS), usher family protein	2	116	3	4	centroid_1033	19.4224803	1.05E-05	centroid_1033	0.00104786
centroid_11884	papC N-terminal domain protein	2	116	3	4	centroid_1188	19.4224803	1.05E-05	centroid_1188	0.00104786
centroid_15214	putative membrane protein	2	116	3	4	centroid_1521	19.4224803	1.05E-05	centroid_1521	0.00104786
centroid_1546	conserved hypothetical protein	2	116	3	4	centroid_1546	19.4224803	1.05E-05	centroid_1546	0.00104786
centroid_1547	fimbrial family protein	2	116	3	4	centroid_1547	19.4224803	1.05E-05	centroid_1547	0.00104786
centroid_6913	HEAT repeats family protein	2	116	3	4	centroid_6913	19.4224803	1.05E-05	centroid_6913	0.00104786
centroid_8444	n-negative pili assembly chaperone, N-terminal domain pr	2	116	3	4	centroid_8444	19.4224803	1.05E-05	centroid_8444	0.00104786
centroid_12933	single-stranded DNA-binding family protein	2	1	3	119	centroid_1293	16.9377561	3.86E-05	centroid_1293	0.00380803
centroid_15949	conserved hypothetical protein	2	1	3	119	centroid_1594	16.9377561	3.86E-05	centroid_1594	0.00380803
centroid_8320	peptidase C39 family protein	2	1	3	119	centroid_8320	16.9377561	3.86E-05	centroid_8320	0.00380803
centroid_8321	conserved hypothetical protein	2	1	3	119	centroid_8321	16.9377561	3.86E-05	centroid_8321	0.00380803
centroid_8322	putative zinc-dependent metallopeptidase domain protein	2	1	3	119	centroid_8322	16.9377561	3.86E-05	centroid_8322	0.00380803
centroid_8323	putative zinc-dependent metallopeptidase domain protein	2	1	3	119	centroid_8323	16.9377561	3.86E-05	centroid_8323	0.00380803
centroid_8324	conserved hypothetical protein	2	1	3	119	centroid_8324	16.9377561	3.86E-05	centroid_8324	0.00380803
centroid_8358	hypothetical protein	2	1	3	119	centroid_8358	16.9377561	3.86E-05	centroid_8358	0.00380803
centroid_8359	hypothetical protein	2	1	3	119	centroid_8359	16.9377561	3.86E-05	centroid_8359	0.00380803
centroid_9031	nosyl-l-methionine hydroxide adenosyltransferase family	2	1	3	119	centroid_9031	16.9377561	3.86E-05	centroid_9031	0.00380803
centroid_9161	putative membrane protein	2	1	3	119	centroid_9161	16.9377561	3.86E-05	centroid_9161	0.00380803
centroid_9181	putative yfdA	2	1	3	119	centroid_9181	16.9377561	3.86E-05	centroid_9181	0.00380803
centroid_9239	phage tail-collar fibre family protein	2	1	3	119	centroid_9239	16.9377561	3.86E-05	centroid_9239	0.00380803
centroid_9340	conserved hypothetical protein	2	1	3	119	centroid_9340	16.9377561	3.86E-05	centroid_9340	0.00380803

centroid_9358	conserved hypothetical protein	2	1	3	119	centroid_9358	16.9377561	3.86E-05	centroid_9358	0.00380803
centroid_9360	prophage CP4-57 regulatory family protein	2	1	3	119	centroid_9360	16.9377561	3.86E-05	centroid_9360	0.00380803
centroid_9362	ogr/Delta-like zinc finger family protein	2	1	3	119	centroid_9362	16.9377561	3.86E-05	centroid_9362	0.00380803
centroid_9363	polarity suppression protein	2	1	3	119	centroid_9363	16.9377561	3.86E-05	centroid_9363	0.00380803
centroid_9399	conserved hypothetical protein	2	1	3	119	centroid_9399	16.9377561	3.86E-05	centroid_9399	0.00380803
centroid_9400	conserved hypothetical protein	2	1	3	119	centroid_9400	16.9377561	3.86E-05	centroid_9400	0.00380803
centroid_9402	conserved hypothetical protein	2	1	3	119	centroid_9402	16.9377561	3.86E-05	centroid_9402	0.00380803
centroid_9550	putative prophage protein	2	1	3	119	centroid_9550	16.9377561	3.86E-05	centroid_9550	0.00380803
centroid_9554	conserved hypothetical protein	2	1	3	119	centroid_9554	16.9377561	3.86E-05	centroid_9554	0.00380803
centroid_9590	phage integrase family protein	2	1	3	119	centroid_9590	16.9377561	3.86E-05	centroid_9590	0.00380803
centroid_9591	conserved hypothetical protein	2	1	3	119	centroid_9591	16.9377561	3.86E-05	centroid_9591	0.00380803
centroid_9653	conserved hypothetical protein	2	1	3	119	centroid_9653	16.9377561	3.86E-05	centroid_9653	0.00380803
centroid_9654	major Facilitator Superfamily protein	2	1	3	119	centroid_9654	16.9377561	3.86E-05	centroid_9654	0.00380803
centroid_9655	conserved hypothetical protein	2	1	3	119	centroid_9655	16.9377561	3.86E-05	centroid_9655	0.00380803
centroid_9748	bacterial Ig-like domain family protein	2	1	3	119	centroid_9748	16.9377561	3.86E-05	centroid_9748	0.00380803
centroid_9793	conserved hypothetical protein	2	1	3	119	centroid_9793	16.9377561	3.86E-05	centroid_9793	0.00380803
centroid_17241	conserved hypothetical protein	2	115	3	5	centroid_1724	16.5278334	4.79E-05	centroid_1724	0.00165548
centroid_4046	conserved hypothetical protein	2	115	3	5	centroid_4046	16.5278334	4.79E-05	centroid_4046	0.00165548
centroid_4202	YibA domain protein	2	115	3	5	centroid_4202	16.5278334	4.79E-05	centroid_4202	0.00165548
centroid_1029	putative yfaH	1	120	4	0	centroid_1029	75.0285167	4.64E-18	centroid_1029	5.16E-07
centroid_12417	outer membrane protein C	1	120	4	0	centroid_1241	75.0285167	4.64E-18	centroid_1241	5.16E-07
centroid_14253	conserved hypothetical protein	1	120	4	0	centroid_1425	75.0285167	4.64E-18	centroid_1425	5.16E-07
centroid_14412	ribbon-helix-helix , copG family protein	1	120	4	0	centroid_1441	75.0285167	4.64E-18	centroid_1441	5.16E-07
centroid_18326	2-keto-3-deoxy-galactonokinase family protein	1	120	4	0	centroid_1832	75.0285167	4.64E-18	centroid_1832	5.16E-07
centroid_18327	2-keto-3-deoxy-galactonokinase family protein	1	120	4	0	centroid_1832	75.0285167	4.64E-18	centroid_1832	5.16E-07
centroid_2074	FCD domain protein	1	120	4	0	centroid_2074	75.0285167	4.64E-18	centroid_2074	5.16E-07
centroid_2075	2-keto-3-deoxy-galactonokinase family protein	1	120	4	0	centroid_2075	75.0285167	4.64E-18	centroid_2075	5.16E-07
centroid_2076	2-dehydro-3-deoxy-6-phosphogalactonate aldolase	1	120	4	0	centroid_2076	75.0285167	4.64E-18	centroid_2076	5.16E-07
centroid_2077	D-galactonate dehydratase	1	120	4	0	centroid_2077	75.0285167	4.64E-18	centroid_2077	5.16E-07
centroid_2078	D-galactonate transporter	1	120	4	0	centroid_2078	75.0285167	4.64E-18	centroid_2078	5.16E-07
centroid_311	sugar efflux transporter A	1	120	4	0	centroid_311	75.0285167	4.64E-18	centroid_311	5.16E-07
centroid_8456	2-keto-3-deoxy-galactonokinase family protein	1	120	4	0	centroid_8456	75.0285167	4.64E-18	centroid_8456	5.16E-07
centroid_10308	conserved hypothetical protein	1	119	4	1	centroid_1030	59.0820313	1.51E-14	centroid_1030	2.56E-06
centroid_13075	conserved hypothetical protein	1	119	4	1	centroid_1307	59.0820313	1.51E-14	centroid_1307	2.56E-06
centroid_15451	conserved hypothetical protein	1	119	4	1	centroid_1545	59.0820313	1.51E-14	centroid_1545	2.56E-06
centroid_1583	conserved hypothetical protein	1	119	4	1	centroid_1583	59.0820313	1.51E-14	centroid_1583	2.56E-06
centroid_1666	conserved hypothetical protein	1	119	4	1	centroid_1666	59.0820313	1.51E-14	centroid_1666	2.56E-06
centroid_2052	e-6-phosphate isomerases/6-phosphogluconolactonase f	1	119	4	1	centroid_2052	59.0820313	1.51E-14	centroid_2052	2.56E-06
centroid_7208	conserved hypothetical protein	1	119	4	1	centroid_7208	59.0820313	1.51E-14	centroid_7208	2.56E-06
centroid_9962	gamma-glutamyltranspeptidase domain protein	1	119	4	1	centroid_9962	59.0820313	1.51E-14	centroid_9962	2.56E-06
centroid_18532	type VI secretion system effector, Hcp1 family protein	1	116	4	4	centroid_1853	35.16877	3.02E-09	centroid_1853	3.52E-05
centroid_848	type VI secretion system effector, Hcp1 family protein	1	116	4	4	centroid_848	35.16877	3.02E-09	centroid_848	3.52E-05
centroid_11169	conserved hypothetical protein	1	115	4	5	centroid_1116	30.7423871	2.95E-08	centroid_1116	6.29E-05
centroid_7792	thanolamine utilization - propanediol utilization family prote	1	113	4	7	centroid_7792	24.3065939	8.22E-07	centroid_7792	0.00016237
centroid_8736	thanolamine utilization - propanediol utilization family prote	1	113	4	7	centroid_8736	24.3065939	8.22E-07	centroid_8736	0.00016237
centroid_13232	integrase core domain protein	1	112	4	8	centroid_1323	21.8943968	2.88E-06	centroid_1323	0.00024187
centroid_17674	conserved hypothetical protein	1	110	4	10	centroid_1767	18.1059966	2.09E-05	centroid_1767	0.00048229
centroid_2839	conserved hypothetical protein	1	110	4	10	centroid_2839	18.1059966	2.09E-05	centroid_2839	0.00048229
centroid_2840	repair family protein	1	110	4	10	centroid_2840	18.1059966	2.09E-05	centroid_2840	0.00048229
centroid_4254	hypothetical protein	1	110	4	10	centroid_4254	18.1059966	2.09E-05	centroid_4254	0.00048229
centroid_4506	conserved hypothetical protein	1	110	4	10	centroid_4506	18.1059966	2.09E-05	centroid_4506	0.00048229
centroid_5513	integrase core domain protein	1	110	4	10	centroid_5513	18.1059966	2.09E-05	centroid_5513	0.00048229
centroid_12444	uvate-dependent sugar phosphotransferase system, EIIA	1	109	4	11	centroid_1244	16.5916982	4.64E-05	centroid_1244	0.00065302
centroid_14010	alpha-glucosidase YihQ domain protein	1	109	4	11	centroid_1401	16.5916982	4.64E-05	centroid_1401	0.00065302
centroid_14132	integrase core domain protein	1	109	4	11	centroid_1413	16.5916982	4.64E-05	centroid_1413	0.00065302
centroid_17686	putative domain protein	1	109	4	11	centroid_1768	16.5916982	4.64E-05	centroid_1768	0.00065302
centroid_2249	corbate-specific phosphotransferase enzyme IIA compon	1	109	4	11	centroid_2249	16.5916982	4.64E-05	centroid_2249	0.00065302
centroid_1012	sensory box protein	1	108	4	12	centroid_1012	15.267375	9.33E-05	centroid_1012	0.00086448
centroid_1013	response regulator	1	108	4	12	centroid_1013	15.267375	9.33E-05	centroid_1013	0.00086448
centroid_1014	acetate CoA-transferase subunit alpha	1	108	4	12	centroid_1014	15.267375	9.33E-05	centroid_1014	0.00086448
centroid_1015	acetate CoA-transferase subunit beta	1	108	4	12	centroid_1015	15.267375	9.33E-05	centroid_1015	0.00086448
centroid_1016	short-chain fatty acids transporter	1	108	4	12	centroid_1016	15.267375	9.33E-05	centroid_1016	0.00086448
centroid_1017	acetyl-CoA C-acetyltransferase family protein	1	108	4	12	centroid_1017	15.267375	9.33E-05	centroid_1017	0.00086448
centroid_10599	putative predicted inner membrane protein	1	108	4	12	centroid_1059	15.267375	9.33E-05	centroid_1059	0.00086448
centroid_11719	integrase core domain protein	1	108	4	12	centroid_1171	15.267375	9.33E-05	centroid_1171	0.00086448
centroid_13637	integrase core domain protein	1	108	4	12	centroid_1363	15.267375	9.33E-05	centroid_1363	0.00086448
centroid_17355	conserved hypothetical protein	1	108	4	12	centroid_1735	15.267375	9.33E-05	centroid_1735	0.00086448
centroid_3651	conserved hypothetical protein	1	108	4	12	centroid_3651	15.267375	9.33E-05	centroid_3651	0.00086448
centroid_4246	integrase core domain protein	1	108	4	12	centroid_4246	15.267375	9.33E-05	centroid_4246	0.00086448
centroid_5438	conserved hypothetical protein	1	108	4	12	centroid_5438	15.267375	9.33E-05	centroid_5438	0.00086448
centroid_5520	integrase core domain protein	1	108	4	12	centroid_5520	15.267375	9.33E-05	centroid_5520	0.00086448
centroid_5995	integrase core domain protein	1	108	4	12	centroid_5995	15.267375	9.33E-05	centroid_5995	0.00086448
centroid_7693	ative signal transduction histidine-kinase atoS domain pro	1	108	4	12	centroid_7693	15.267375	9.33E-05	centroid_7693	0.00086448
centroid_7694	sensory box protein	1	108	4	12	centroid_7694	15.267375	9.33E-05	centroid_7694	0.00086448
centroid_8264	thiolase, N-terminal domain protein	1	108	4	12	centroid_8264	15.267375	9.33E-05	centroid_8264	0.00086448
centroid_8265	acetyl-CoA C-acetyltransferase family protein	1	108	4	12	centroid_8265	15.267375	9.33E-05	centroid_8265	0.00086448
centroid_1060	fimbrial family protein	0	120	5	0	centroid_1060	100.31467	1.30E-23	centroid_1060	4.26E-09
centroid_11948	GY family of carbohydrate kinase, N-terminal domain pro	0	120	5	0	centroid_1194	100.31467	1.30E-23	centroid_1194	4.26E-09
centroid_1240	type VII secretion system (T7SS), usher family protein	0	120	5	0	centroid_1240	100.31467	1.30E-23	centroid_1240	4.26E-09
centroid_1241	fimbrial family protein	0	120	5	0	centroid_1241	100.31467	1.30E-23	centroid_1241	4.26E-09
centroid_13867	type VII secretion system (T7SS), usher family protein	0	120	5	0	centroid_1386	100.31467	1.30E-23	centroid_1386	4.26E-09
centroid_14011	class II Aldolase and Adducin N-terminal domain protein	0	120	5	0	centroid_1401	100.31467	1.30E-23	centroid_1401	4.26E-09
centroid_1582	conserved hypothetical protein	0	120	5	0	centroid_1582	100.31467	1.30E-23	centroid_1582	4.26E-09
centroid_16905	major MR/P fimbria domain protein	0	120	5	0	centroid_1690	100.31467	1.30E-23	centroid_1690	4.26E-09
centroid_17640	conserved hypothetical protein	0	120	5	0	centroid_1764	100.31467	1.30E-23	centroid_1764	4.26E-09
centroid_1885	conserved hypothetical protein	0	120	5	0	centroid_1885	100.31467	1.30E-23	centroid_1885	4.26E-09
centroid_2767	conserved hypothetical protein	0	120	5	0	centroid_2767	100.31467	1.30E-23	centroid_2767	4.26E-09
centroid_3536	conserved hypothetical protein	0	120	5	0	centroid_3536	100.31467	1.30E-23	centroid_3536	4.26E-09
centroid_3537	2,3-diketo-L-gulonate reductase	0	120	5	0	centroid_3537	100.31467	1.30E-23	centroid_3537	4.26E-09
centroid_3538	HTH-type transcriptional regulator YiaJ	0	120	5	0	centroid_3538	100.31467	1.30E-23	centroid_3538	4.26E-09
centroid_3815	TRAP transporter solute receptor, DctP family protein	0	120	5	0	centroid_3815	100.31467	1.30E-23	centroid_3815	4.26E-09
centroid_3816	3-keto-L-gulonate-6-phosphate decarboxylase SgbH	0	120	5	0	centroid_3816	100.31467	1.30E-23	centroid_3816	4.26E-09
centroid_3817	xylose isomerase-like TIM barrel family protein	0	120	5	0	centroid_3817	100.31467	1.30E-23	centroid_3817	4.26E-09
centroid_6935	hypothetical protein	0	120	5	0	centroid_6935	100.31467	1.30E-23	centroid_6935	4.26E-09
centroid_7184	papC N-terminal domain protein	0	120	5	0	centroid_7184	100.31467	1.30E-23	centroid_7184	4.26E-09
centroid_7185	type VII secretion system (T7SS), usher family protein	0	120	5	0	centroid_7185	100.31467	1.30E-23	centroid_7185	4.26E-09
centroid_77	conserved hypothetical protein	0	120	5	0	centroid_77	100.31467	1.30E-23	centroid_77	4.26E-09
centroid_9912	type VII secretion system (T7SS), usher family protein	0	120	5	0	centroid_9912	100.31467	1.30E-23	centroid_9912	4.26E-09
centroid_9913	type VII secretion system (T7SS), usher family protein	0	120	5	0	centroid_9913	100.31467	1.30E-23	centroid_9913	4.26E-09
centroid_11173	carbohydrate kinase, FGGY family	0	119	5	1	centroid_1117	82.7370011	9.37E-20	centroid_1117	2.56E-08
centroid_17637	L-xylulose/3-keto-L-gulonate kinase	0	119	5	1	centroid_1763	82.7370011	9.37E-20	centroid_1763	2.56E-08
centroid_18274	gram-negative porin family protein	0	119	5	1	centroid_1827	82.7370011	9.37E-20	centroid_1827	2.56E-08
centroid_3818	L-ribulose-5-phosphate 4-epimerase	0	119	5	1	centroid_3818	82.7370011	9.37E-20	centroid_3818	2.56E-08
centroid_8454	fimbrial family protein	0	119	5	1	centroid_8454	82.7370011	9.37E-20	centroid_8454	2.56E-08
centroid_14943	inner membrane YcfZ domain protein	0	118	5	2	centroid_1494	70.1816611	5.41E-17	centroid_1494	8.95E-08
centroid_15256	conserved hypothetical protein	0	118	5	2	centroid_1525	70.1816611	5.41E-17	centroid_1525	8.95E-08
centroid_17545	conserved hypothetical protein	0	118	5	2	centroid_1754	70.1816611	5.41E-17	centroid_1754	8.95E-08

centroid_1782	inner membrane protein YcfZ	0	118	5	2	centroid_1782	70.1816611	5.41E-17	centroid_1782	8.95E-08
centroid_4537	inner membrane protein YcfZ	0	118	5	2	centroid_4537	70.1816611	5.41E-17	centroid_4537	8.95E-08
centroid_5240	conserved hypothetical protein	0	118	5	2	centroid_5240	70.1816611	5.41E-17	centroid_5240	8.95E-08
centroid_5924	conserved hypothetical protein	0	117	5	3	centroid_5924	60.76528	6.43E-15	centroid_5924	2.39E-07
centroid_13071	conserved hypothetical protein	0	116	5	4	centroid_1307	53.4415409	2.66E-13	centroid_1307	5.37E-07
centroid_16855	ia polymorphic membrane (Chlamydia_PMP) repeat famil	0	116	5	4	centroid_1685	53.4415409	2.66E-13	centroid_1685	5.37E-07
centroid_16930	phage late control gene D family protein	0	116	5	4	centroid_1693	53.4415409	2.66E-13	centroid_1693	5.37E-07
centroid_7097	conserved hypothetical protein	0	116	5	4	centroid_7097	53.4415409	2.66E-13	centroid_7097	5.37E-07
centroid_2768	putative type-1 fimbrial protein, A chain	0	115	5	5	centroid_2768	47.582654	5.27E-12	centroid_2768	1.07E-06
centroid_13866	type VII secretion system (T7SS), usher family protein	0	114	5	6	centroid_1386	42.7891165	6.10E-11	centroid_1386	1.97E-06
centroid_2145	tonB-dependent vitamin B12 receptor	0	114	5	6	centroid_2145	42.7891165	6.10E-11	centroid_2145	1.97E-06
centroid_6916	conserved hypothetical protein	0	114	5	6	centroid_6916	42.7891165	6.10E-11	centroid_6916	1.97E-06
centroid_14039	class II Aldolase and Adducin N-terminal domain protein	0	113	5	7	centroid_1403	38.7945935	4.71E-10	centroid_1403	3.38E-06
centroid_4431	conserved hypothetical protein	0	113	5	7	centroid_4431	38.7945935	4.71E-10	centroid_4431	3.38E-06
centroid_1247	conserved hypothetical protein	0	109	5	11	centroid_1247	27.8103796	1.34E-07	centroid_1247	1.86E-05
centroid_13565	putative colanic acid biosynthesis domain protein	0	109	5	11	centroid_1356	27.8103796	1.34E-07	centroid_1356	1.86E-05
centroid_14852	-independent periplasmic transporters, DctQ component	0	105	5	15	centroid_1485	21.2208581	4.09E-06	centroid_1485	6.61E-05
centroid_17471	conserved hypothetical protein	0	105	5	15	centroid_1747	21.2208581	4.09E-06	centroid_1747	6.61E-05
centroid_2637	conserved hypothetical protein	0	105	5	15	centroid_2637	21.2208581	4.09E-06	centroid_2637	6.61E-05
centroid_2747	antitoxin MqsA	0	105	5	15	centroid_2747	21.2208581	4.09E-06	centroid_2747	6.61E-05
centroid_4650	2,3-diketo-L-gulonate-binding periplasmic protein YiaO	0	105	5	15	centroid_4650	21.2208581	4.09E-06	centroid_4650	6.61E-05
centroid_4651	TRAP transporter, DctM subunit	0	105	5	15	centroid_4651	21.2208581	4.09E-06	centroid_4651	6.61E-05
centroid_4652	keto-L-gulonate TRAP transporter small permease protei	0	105	5	15	centroid_4652	21.2208581	4.09E-06	centroid_4652	6.61E-05
centroid_4653	conserved hypothetical protein	0	104	5	16	centroid_4653	19.965874	7.88E-06	centroid_4653	8.68E-05
centroid_17467	type II secretion system protein L	0	102	5	18	centroid_1746	17.7834834	2.48E-05	centroid_1746	0.00014347
centroid_3084	BFD-like [2Fe-2S] binding domain protein	0	102	5	18	centroid_3084	17.7834834	2.48E-05	centroid_3084	0.00014347
centroid_3085	bacterioferritin	0	102	5	18	centroid_3085	17.7834834	2.48E-05	centroid_3085	0.00014347
centroid_3086	be 4 prepilin-like proteins leader peptide-processing enzy	0	102	5	18	centroid_3086	17.7834834	2.48E-05	centroid_3086	0.00014347
centroid_3087	type II secretion system (T2SS), M family protein	0	102	5	18	centroid_3087	17.7834834	2.48E-05	centroid_3087	0.00014347
centroid_3098	AAA domain protein	0	102	5	18	centroid_3098	17.7834834	2.48E-05	centroid_3098	0.00014347
centroid_3099	putative peptidoglycan binding domain protein	0	102	5	18	centroid_3099	17.7834834	2.48E-05	centroid_3099	0.00014347
centroid_3100	lcium-binding protein required for initiation of chromosome	0	102	5	18	centroid_3100	17.7834834	2.48E-05	centroid_3100	0.00014347
centroid_3700	UDP-glucose 6-dehydrogenase	0	102	5	18	centroid_3700	17.7834834	2.48E-05	centroid_3700	0.00014347
centroid_5500	gspL periplasmic domain protein	0	102	5	18	centroid_5500	17.7834834	2.48E-05	centroid_5500	0.00014347
centroid_8581	putative general secretion pathway protein A	0	102	5	18	centroid_8581	17.7834834	2.48E-05	centroid_8581	0.00014347
centroid_10869	type II secretion system protein H	0	101	5	19	centroid_1086	16.8287825	4.09E-05	centroid_1086	0.00018123
centroid_12074	type II secretion system protein H	0	101	5	19	centroid_1207	16.8287825	4.09E-05	centroid_1207	0.00018123
centroid_17465	type II secretion system (T2SS), F family protein	0	101	5	19	centroid_1746	16.8287825	4.09E-05	centroid_1746	0.00018123
centroid_17466	type II secretion system (T2SS), F family protein	0	101	5	19	centroid_1746	16.8287825	4.09E-05	centroid_1746	0.00018123
centroid_3083	putative bifunctional chitinase/lysozyme	0	101	5	19	centroid_3083	16.8287825	4.09E-05	centroid_3083	0.00018123
centroid_3089	type II secretion system (T2SS), K family protein	0	101	5	19	centroid_3089	16.8287825	4.09E-05	centroid_3089	0.00018123
centroid_3090	repilin-type N-terminal cleavage/methylation domain prote	0	101	5	19	centroid_3090	16.8287825	4.09E-05	centroid_3090	0.00018123
centroid_3091	type II secretion system protein I	0	101	5	19	centroid_3091	16.8287825	4.09E-05	centroid_3091	0.00018123
centroid_3092	type II secretion system protein H	0	101	5	19	centroid_3092	16.8287825	4.09E-05	centroid_3092	0.00018123
centroid_3093	type II secretion system protein G	0	101	5	19	centroid_3093	16.8287825	4.09E-05	centroid_3093	0.00018123
centroid_3094	type II secretion system protein F	0	101	5	19	centroid_3094	16.8287825	4.09E-05	centroid_3094	0.00018123
centroid_3095	type II secretion system protein E	0	101	5	19	centroid_3095	16.8287825	4.09E-05	centroid_3095	0.00018123
centroid_4742	repilin-type N-terminal cleavage/methylation domain prote	0	101	5	19	centroid_4742	16.8287825	4.09E-05	centroid_4742	0.00018123
centroid_5294	shET2 enterotoxin, N-terminal region family protein	0	101	5	19	centroid_5294	16.8287825	4.09E-05	centroid_5294	0.00018123
centroid_6220	shET2 enterotoxin, N-terminal region family protein	0	101	5	19	centroid_6220	16.8287825	4.09E-05	centroid_6220	0.00018123
centroid_7722	type II secretion system protein F	0	101	5	19	centroid_7722	16.8287825	4.09E-05	centroid_7722	0.00018123
centroid_7723	putative type II secretion system F domain protein	0	101	5	19	centroid_7723	16.8287825	4.09E-05	centroid_7723	0.00018123
centroid_8069	type II secretion system protein D	0	101	5	19	centroid_8069	16.8287825	4.09E-05	centroid_8069	0.00018123
centroid_8070	type II secretion system protein L	0	101	5	19	centroid_8070	16.8287825	4.09E-05	centroid_8070	0.00018123
centroid_8580	carbohydrate binding domain protein	0	101	5	19	centroid_8580	16.8287825	4.09E-05	centroid_8580	0.00018123
centroid_4096	heat-stable enterotoxin A3/A4	0	100	5	20	centroid_4096	15.9505208	6.50E-05	centroid_4096	0.00022654
centroid_7923	heat-stable enterotoxin A3/A4	0	100	5	20	centroid_7923	15.9505208	6.50E-05	centroid_7923	0.00022654
centroid_13907	conserved hypothetical protein	0	99	5	21	centroid_1390	15.1398804	9.98E-05	centroid_1390	0.00028047
centroid_1481	putative lipoprotein	0	99	5	21	centroid_1481	15.1398804	9.98E-05	centroid_1481	0.00028047
centroid_1482	conserved hypothetical protein	0	99	5	21	centroid_1482	15.1398804	9.98E-05	centroid_1482	0.00028047
centroid_3096	type II secretion system protein D	0	99	5	21	centroid_3096	15.1398804	9.98E-05	centroid_3096	0.00028047
centroid_7749	hypothetical protein	0	99	5	21	centroid_7749	15.1398804	9.98E-05	centroid_7749	0.00028047
centroid_8068	type II secretion system D domain protein	0	99	5	21	centroid_8068	15.1398804	9.98E-05	centroid_8068	0.00028047
centroid_8471	conserved hypothetical protein	0	99	5	21	centroid_8471	15.1398804	9.98E-05	centroid_8471	0.00028047
